# Conversion of Helix 1 into a Loop in Prion Protein Misfolding

**DOI:** 10.1021/acsomega.3c00212

**Published:** 2023-02-10

**Authors:** Ayşenaz Tavşanlı, Bülent Balta

**Affiliations:** Department of Molecular Biology and Genetics, Istanbul Technical University, Maslak 34469 Istanbul, Turkey

## Abstract

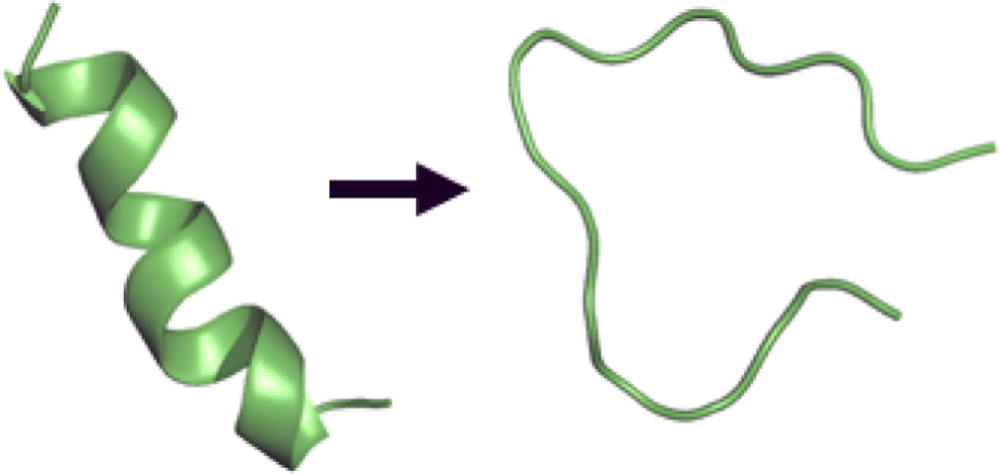

Cellular prion protein PrP^C^ consists of three α-helices, one β-sheet, and an unstructured N-terminal domain. Misfolding of this protein into the scrapie form (PrP^Sc^) increases dramatically the β-sheet content. H1 is the most stable helix on PrP^C^ and contains an unusual number of hydrophilic amino acids. Its fate in PrP^Sc^ is not clear. We performed replica exchange molecular dynamics simulations on H1 alone, H1 together with an N-terminally flanking H1B1 loop and H1 in complex with other hydrophilic regions of the prion protein. In the presence of the H^99^SQWNKPSKPKTNMK^113^ sequence, H1 is almost completely converted to a loop structure stabilized by a network of salt bridges. On the other hand, H1 retains its helical structure alone or together with the other sequences considered in this study. We carried out an additional simulation by restraining the distance between the two ends of H1, mimicking a possible geometric restriction by the rest of the protein. Even though the loop was the major conformation, a significant amount of helical structure was also observed. This suggests that the interaction with H^99^SQWNKPSKPKTNMK^113^ is necessary for complete helix-to-loop conversion.

## 1 Introduction

The conversion of the α-helix rich monomeric cellular form of the prion protein (PrP^C^) to β-sheet rich oligomers (PrP^Sc^) is the cause of transmissible spongiform encephalopathies (TSEs) such as Creutzfeldt–Jakob disease, Gerstmann–Sträussler–Scheinker disease (GSS), kuru, fatal familial insomnia, and scrapie.^1−3^ PrP^C^ contains three α-helices (H1: 146–158, H2: 174–196, and H3: 203–228 in sheep) and two short β-strands (B1: 129–134 and B2: 163–167 in sheep).^4^ The sequence comprising only H2 and H3 is capable to form misfolded aggregates.^5^ On the other hand, residues 23–144 flanking the N-terminal side of H1 are involved in GSS.^6^ Thus, both sides of H1 can form misfolded structures independent of each other without the assistance of H1. However, the structure and role of H1 in misfolded aggregates remain debated. In this study, we present a loop structure for the misfolded H1, stabilized by interactions with a particular hydrophilic PrP sequence, determined via molecular dynamics simulations (below, H1 will be used to refer to both the helix in PrP^C^ and the misfolded structure of this region in PrP^Sc^).

The structure of PrP^Sc^ is not known. Since the same protein can lead to more than one disease, it is likely that several misfolded structures exist. Nevertheless, experiments indicate that the helical content of PrP^C^ is completely lost^7^ in PrP^Sc^, while H2 and H3 are parts of the β-sheet core of PrP^Sc^.^5,7−9^ A number of different PrP^Sc^ structures were proposed in the literature. In the 4-rung solenoid model, H1 is incorporated into a β-strand.^10,11^ The β-hairpin model consisting of in-register β-strands also locates H1 in a β-strand, where it interacts with H1 of the adjacent monomers.^12^ In the left-handed β-helix model, H1 forms a loop.^13,14^ The spiral model conserves all three helices.^15,16^ In another model, H2 and H3 form an aggregate, but no structure for H1 is proposed.^17^ On the other hand, H/D exchange studies revealed that the region comprising B2, H2, and H3 and the loops connecting them were protected, suggesting that they are part of the β-sheet core of PrP^Sc^.^7−9^ However, a bimodal mass distribution was observed for B1 together with the preceding hydrophobic palindromic sequence^8,9^ and H1,^8^ indicating two different conformations of PrP^Sc^. H1 was mostly^8^ or completely^9^ unprotected. In the unprotected structure, H1 formed neither an α-helix nor a β-strand in PrP^Sc^.

Although experiments on the full-length prion or the C-terminal region do not detect any sign of helical structure on PrP^Sc^, the situation is different for the N-terminal region. There is experimental evidence about the presence of a helical H1 in the misfolded human PrP(23–159), which contains the N-terminal region of PrP truncated at the C-terminus of H1.^18^ The cryo-EM structure of human PrP(90–178) reveals an in-register β-sheet structure between residues 106 and 145.^19^ However, the conformation of H1 is not resolved.

H1 is mostly composed of hydrophilic amino acids and is the most soluble helix in the Brookhaven Protein Database.^20^ Simulations on the isolated helices indicated that H1 was stable, whereas H2 and H3 were partially unfolded.^21,22^ Two intrahelix salt bridges were proposed to stabilize H1. Disruption of intrahelix salt bridges and formation of intermolecular salt bridges with H1 of a neighboring PrP were suggested to be involved during PrP^Sc^ aggregation.^20^ A synthetic polypeptide corresponding to murine H1 retained the helical structure in different solutions.^23^ CD and NMR spectroscopies on human H1 alone and H1 with N-terminally or C-terminally flanking residues indicated a helical structure.^24^ However, another study on a peptide containing H1 and the C-terminally flanking residues observed a soluble β-hairpin structure.^25^ H1 maintained a helical structure under varying conditions such as high salt concentrations, pH variations, and the presence of organic co-solvents.^24^ Stability of H1 compared to the other PrP^C^ helices is also highlighted by the fact that conversion of H2 and H3 to a β-sheet structure precedes unfolding of H1 during PrP^Sc^ formation.^9^ Nevertheless, at pH = 2, where aspartic acid and glutamic acid residues on H1 were protonated, a decrease in helical content was observed,^24^ providing support to the idea that H1 was stabilized by intrahelix salt bridges. This idea was challenged by experiments showing that mutation of the residues involved in salt bridges did not destabilize the helical structure but increased the efficiency of unfolding via a different pathway.^26^ Another study where charged residues of H1 were mutated pointed out that most of the mutations involving the N-terminal residues D143, E145, and D146 of mouse PrP did not affect PrP^C^ to PrP^Sc^ conversion, whereas many mutations involving the C-terminal residues R147, R150, and E151 abolished PrP^Sc^ formation.^27^ Interestingly, mutants where residues in each salt bridge were exchanged such that the salt bridge was conserved but with the charges reversed did not convert to PrP^Sc^.^27^ Thus, not the salt bridges but the charge structure of H1 appeared to be critical for PrP^Sc^ generation.^27^

The fact that the regions N-terminal and C-terminal to H1 can misfold into PrP^Sc^ forms independent of H1 and of each other suggests that the interactions of these regions with each other and with H1 are limited at least in some PrP^Sc^ structures (note that there are probably more than one PrP^Sc^ structures, each leading to a different TSE). Thus, investigating the structure of different regions separately or in the presence of only short fragments of other regions can be a good strategy to divide the difficult problem of prion misfolding into smaller and more solvable ones. In this study, the misfolded structure of H1 was investigated through replica exchange molecular dynamics (REMD) simulations. In principle, the structure of a given sequence can be affected by the rest of the protein via several means. The backbone and side chains of the sequence can interact with the rest of the protein. The distance between the termini of the sequence can be restricted, or a hydrophobic environment can be imposed by the rest of the protein. In the case of H1, the possibility of a hydrophobic environment and interactions with the backbone of this sequence can be eliminated based on the experimental evidence that H1 is readily available for H/D exchange.^7−9^ The other factors, that is, the effect of the distance between the termini of H1 and the interactions of its side chains with several other sequences on the prion protein, were investigated in this study.

## 2 Methods

REMD simulations^28^ were performed using the Amber 16 program package^29−32^ with the ff10 force field.^33^ Solvent effects were taken into account using the Onufriev, Bashford, and Case generalized Born (model II) method^34^ together with mbondi2 atomic radii and a 0.1 M salt concentration. 12 replicas were used with temperatures 294, 310, 327, 344, 363, 382, 402, 423, 445, 468, 495, and 520 K. The temperature was regulated with the Langevin thermostat. The time step was taken as 1 fs. Heavy atom-hydrogen stretching movements were constrained via the SHAKE algorithm. Exchange attempts were performed every 5000 steps. The systems simulated in REMD calculations were H1 (residues 146–160), H1 + H1B1 (residues 136–160), H1 dimer, H1 in complex with H^99^SQWNKPSKPKTNMK^113^, and H1 in complex with K^197^GENFTETDIKIMER^211^. In addition, H1 was simulated with a distance restraint between the α-carbons of N146 and Y160 such that a quadratic energy penalty with a force constant of 100 kcal·mol^–1^·Å^–1^ was applied when this distance was above 9 Å. The simulation length was 200 ns for the H1 dimer and the H1–H^99^SQWNKPSKPKTNMK^113^ complex, 100 ns for the H1– K^197^GENFTETDIKIMER^211^ complex, and 265 ns for the isolated H1 and 255 ns for H1+H1B1 systems. Average exchange success rates (without counting that of the highest temperature, which is always 0) were 0.42 for isolated H1, 0.34 for H1 + H1B1, 0.24 for the H1 dimer, 0.24 for H1– K^197^GENFTETDIKIMER^211^, 0.26 for H1–H^99^SQWNKPSKPKTNMK^113^, and 0.41 for the restrained H1 simulations.

In the H1+H1B1 simulation, the initial geometry for all replicas was taken from the crystallographic structure (PDB ID: 1TQB).^4^ In the other REMD simulations, two different structures for H1 were given to different replicas in order to facilitate convergence. The initial H1 geometry for some replicas was the loop conformation truncated from the structure obtained in the H1+H1B1 simulation, whereas the initial H1 geometry for the other replicas was the crystallographic one (PDB ID: 1TQB).^4^ To construct the complexes, relevant sequences were truncated from the crystallographic structure (PDB ID: 1TQB)^4^ and put near H1 manually. The N- and C-termini of all peptides were capped by CH_3_CO– and −NHCH_3_ groups, respectively. In order to prevent H1 and the other sequences from moving infinitely away from each other, Y153^A^-Y153^B^, Y153-E203, and Y153-S106 distances were restrained at 20 Å. Additional restraints were used to avoid cis/trans isomerization of peptide bonds and reversal of the stereochemistry of the chiral atoms.

Trajectories were clustered based on the root mean square deviations of Cα atoms with a cluster radius of 4 Å via the hierarchical agglomerative approach using the average distance between members of two clusters. The appearance of clusters with respect to time is shown in Figure S1, and their fractions at different time intervals are given in Table S1. It can be seen from this figure and table that the populations of most clusters are sufficiently stable to draw conclusions after 60–70 ns. Even though the variation appears to be high for clusters 0 and 1 of the H1 dimer, H1-K^197^GENFTETDIKIMER^211^ and H1-H^99^SQWNKPSKPKTNMK^113^ systems, the structures in these clusters fall in the same category and the sum of their fractions remains the same throughout the trajectory. For instance, both clusters 0 and 1 from the H1-H^99^SQWNKPSKPKTNMK^113^ simulation contain loop conformations. Similarly, clusters 0 and 1 from the H1 dimer and H1-K^197^GENFTETDIKIMER^211^ simulations contain helical or distorted helical structures. The decrease in the fraction of one cluster is similar in magnitude to the increase in the fraction of the other cluster. Thus, the helix and loop populations are stable along the trajectory, and the selection of the time interval for analysis does not affect our conclusions for the H1+H1B1, H1 dimer, and H1–H^99^SQWNKPSKPKTNMK^113^ and H1–K^197^GENFTETDIKIMER^211^ simulations. Hence, clustering analysis was done on the whole trajectory for these simulations. On the other hand, in the isolated H1 simulation, Cluster 1, which is the loop structure, is quite abundant until ≈60th ns, whereas it is very rare afterward. Thus, we discarded the first 60 ns for the clustering analysis of the isolated H1 simulation.

**Figure 1:**
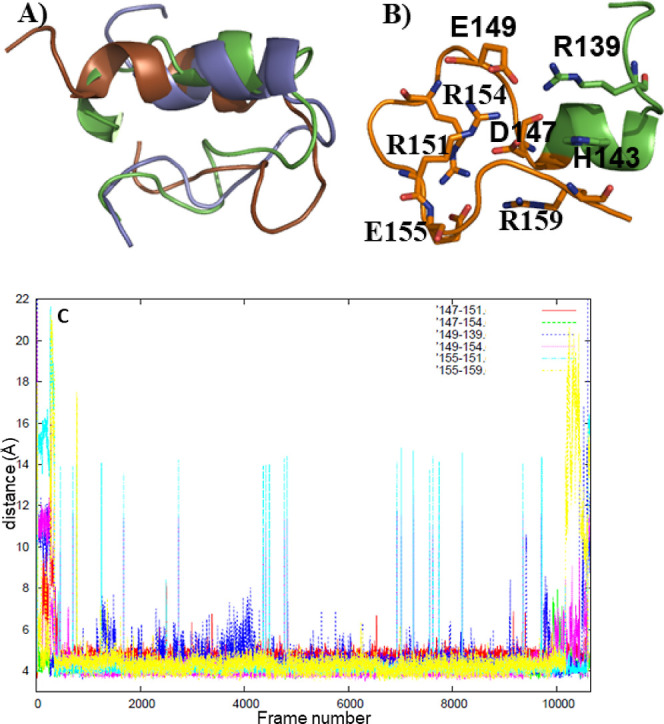
(A) Representatives of the three most abundant clusters of the H1 + H1B1 REMD simulation (green: 75%, blue: 12%, brown: 8%), (B) representative of the cluster (4%) containing loop structure of H1 (hydrogens are omitted), (C) salt bridge distances for the loop structures as measured with respect to Cζ of arginine, Cγ of aspartate, and Cδ of glutamate residues (values ≈ 4–5 Å correspond to salt bridges).

**Figure 2:**
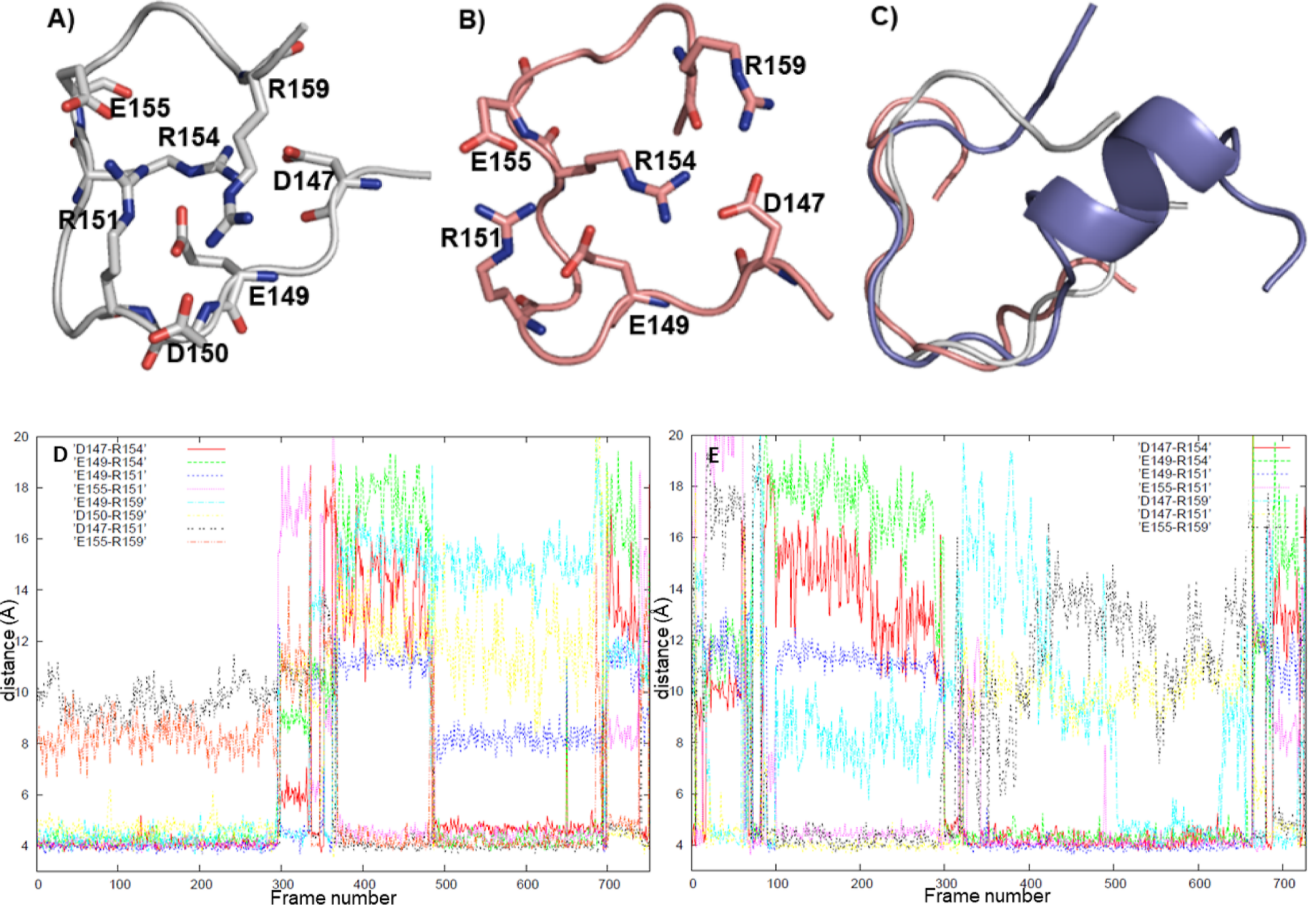
(A,B) Two conformers of the loop (0.4% each), (C) alignment of the central regions (D150-E155) of the loops obtained in H1 and H1+H1B1 simulations, (D,E) salt bridge distances for the loop structures in 2A and 2B, respectively, as measured with respect to Cζ of arginine, Cγ of aspartate, and Cδ of glutamate residues (values ≈ 4–5 Å correspond to salt bridges).

**Figure 3:**
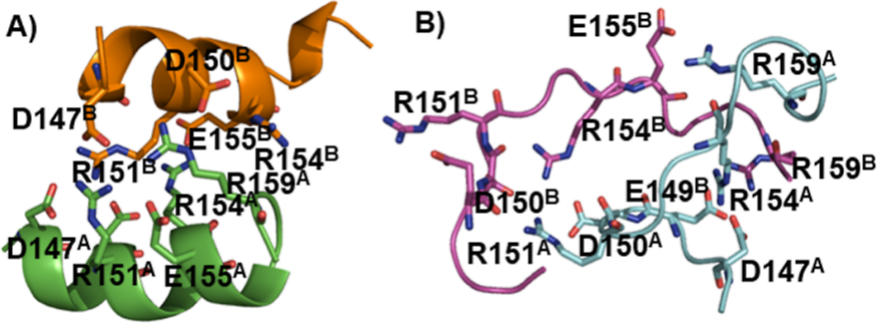
Complexes between two H1 sequences: (A) intact helices (78.4%) and (B) unfolded structures (3.6%).

**Figure 4:**
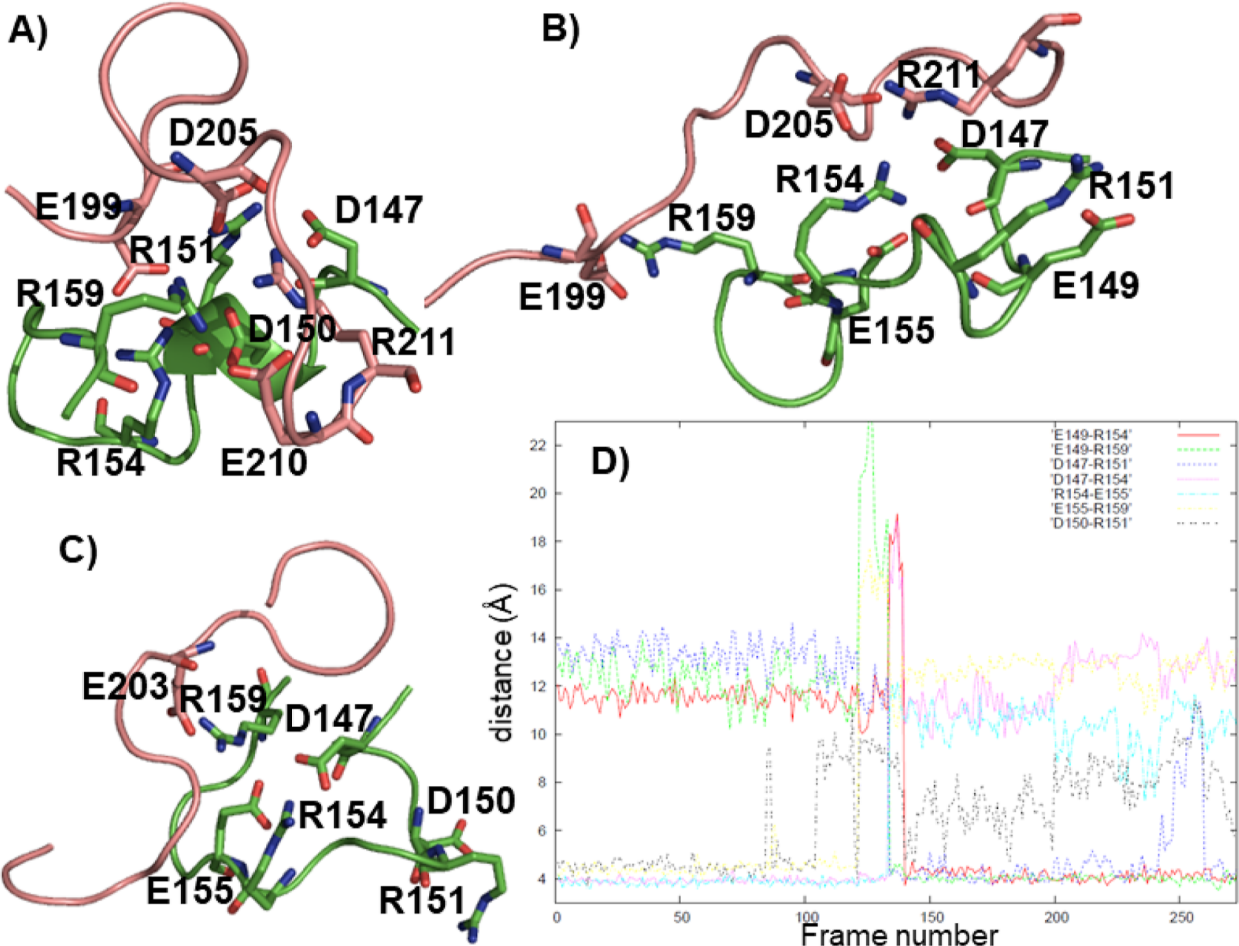
Complexes between the H1 (green) and K^197^GENFTETDIKIMER^211^ (pink) sequences: (A) helical H1 (86%), (B) unfolded H1 (4.2%), (C) loop (0.3%). (D) salt bridge distances for the loop structures as measured with respect to Cζ of arginine, Cγ of aspartate, and Cδ of glutamate residues (values ≈ 4–5 Å correspond to salt bridges).

**Figure 5:**
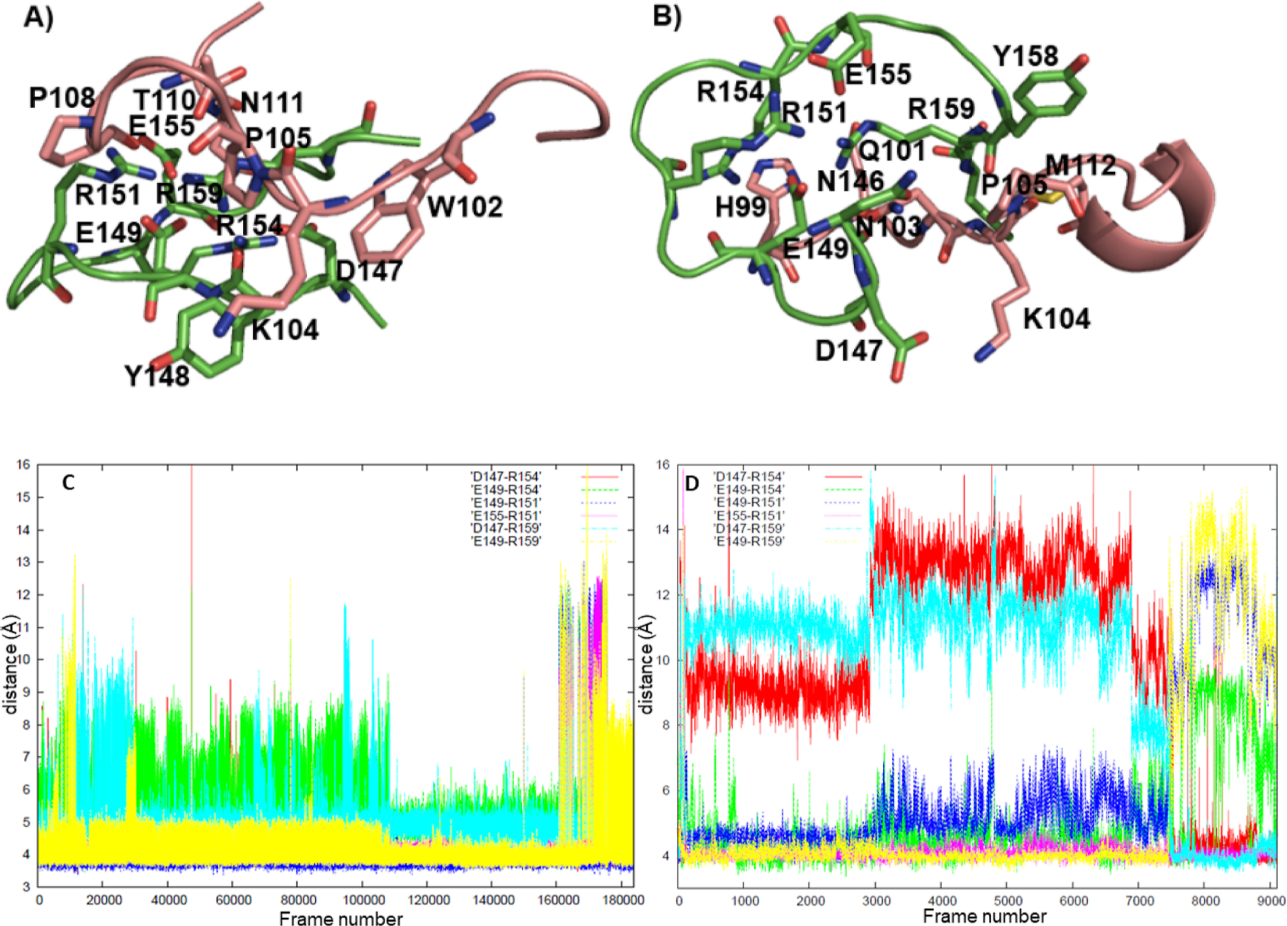
Complexes between the H1 (green) and H^99^SQWNKPSKPKTNMK^113^ (pink) sequences, (A) 92, (B) 4%. (C,D) salt bridge distances for the loop structures in 5A and 5B, respectively, as measured with respect to Cζ of arginine, Cγ of aspartate, and Cδ of glutamate residues (values ≈ 4–5 Å correspond to salt bridges).

The root mean square deviation of Cα atoms of cluster members with respect to the cluster representatives was calculated for the structures displayed in Figures 1–5. The results are shown in Figure S2. Although there are fluctuations in each cluster, the extent of these fluctuations is low in many clusters (Figure S2D,H,I,J,L,M). Visual inspection of the cluster members indicates that fluctuations consist of helix distortions, movements of loop termini, or loop to helix transition attempts (not shown).

Hydrogen bond and salt bridge occurrences were computed within each cluster containing loop structures shown in Figures 1–5. In the calculation of hydrogen bond occurrences, interactions with donor–acceptor distances less than 3 Å and donor-H-acceptor angles larger than 135° were considered as hydrogen bonds. Salt bridge distances were measured with respect to Cζ of arginine, Cγ of aspartate, and Cδ of glutamate residues. Interactions with distances shorter than 5 Å were considered as salt bridges.

Umbrella sampling simulations were performed in order to calculate the potential of mean force for different protonation states of H1. For each umbrella sampling calculation, one of D147, D150, E149, or E155 was protonated. The distance between the Cα atoms of N146 and Y160 was chosen as the reaction coordinate and scanned from 5 to 40 Å with 0.5 Å intervals. To restrain the distance between the Cα atoms of N146 and Y160, a quadratic restraint with a force constant of 1 kcal/mol was applied. Each window was simulated for 50 ns at 310 K with a time step of 2 ns using the SHAKE algorithm and the Langevin thermostat. For each protonation state, 71 windows were simulated. Thus, the total simulation length for each protonation state was 3550 ns. The N146 and Y160 distances were written to the disk at each 50 steps. The same solvent method as in the REMD simulations was used. Free energy profiles were constructed using the weighted histogram analysis method.^35−37^ Statistical uncertainties in the potentials of the mean force calculated using the bootstrap technique were on the order of 10^–2^ kcal/mol.

## 3 Results

### 3.1 Structure of H1 + H1B1

Several experimental studies showed that prion residues C-terminal to the H1 are part of the β-sheet core of PrP^Sc^.^7−9^ Also, it was observed that residues N-terminal to the loop connecting H1 to B1 (H1B1 loop) acquired two conformations,^8,9^ one of which contributes to the β-sheet. Since the residues flanking H1B1 and H1 are already known to form β-strands at least in some cases, we first focused on the intrinsic conformational behavior of H1B1 + H1 (residues 136–160). A 255 ns long REMD simulation was performed. The initial structure in all replicas was the conformation seen in PrP^C^. We clustered the trajectory using the Cα atoms. Most clusters (comprising 96% of the simulation) displayed a helix for H1, differing in the extent of distortion of the helix and the conformation of H1B1 (Figure 1A). Interestingly, a loop conformation of H1 appeared during 4% of the simulation (Figure 1B). This structure was stabilized via E149-R154, E149-R139, D147-R154, D147-R151, E155-R151 and E155-R159 salt bridges. A plot of the salt bridge distances within the cluster (Figure 1C) and the occurrences of salt bridges (Table S2) indicated that very little distortion of the salt bridge network occurred among the members of the cluster containing loop structures. The C-terminal half of H1B1 formed an α-helix. The interactions between H1 and H1B1 were limited to the E149-R139 salt bridge, two hydrogen bonds with low occurrences (Table S2), and hydrophobic contacts such as the one between hydrophobic parts of H143 and R159. The E149-R139 salt bridge was permanent (Figure 1C and Table S2), but a visual inspection of the cluster members showed that although there was at least one hydrophobic interaction between H1 and H1B1, the residues involved varied among different members of the cluster (not shown).

### 3.2 Structure of Isolated H1

We next investigated whether H1 alone was capable of folding into a loop. Both the loop and helix conformations were given to REMD as initial structures of different replicas. A clustering analysis showed the presence of a loop conformation during 9% of the simulation. However, the loop was mainly observed during the first 60 ns and rarely appeared in the rest of the simulation (Figure S1). Thus, the first 60 ns were excluded as the equilibration period, and a new clustering analysis was performed. 99.2% of the trajectory consisted of helical structures with different extents of distortion (not shown). The total abundance of two clusters containing a loop conformation was 0.8% (0.4% each). The two loop structures differed from each other in the salt bridge pattern (Figure 2). The E155-R151 salt bridge, which stabilized the central part of the loop, was observed in almost all members of both clusters (Figure 2D–E, Table S3). In the cluster represented by the structure in Figure 2A, the D147-R154 and E149-R154 interactions were present in most cluster members, whereas the E149-R151, E149-R159, and D150-R159 interactions were interchangeable with D147-R151 and E155-R159 salt bridges (Figure 2D, Table S3). On the other hand, in the cluster represented by the structure in Figure 2B, D147-R154, E149-R154, and E149-R151 salt bridges were interchangeable with D147-R151 and E155-R159 salt bridges (Figure 2E, Table S3). The E149-R159 and D150-R159 interactions were almost absent (Table S3). In addition to salt bridges, hydrogen bonds formed in some cluster members. However, these interactions were rare, and the residues involved varied within the clusters (Table S3). Thus, they are not discussed here.

The variation of salt bridge patterns within each cluster and between clusters is an indication of the flexibility of the loop termini. An alignment of the representatives of the clusters containing a loop in isolated H1 and H1 + H1B1 simulations shows that the central part of the loop is very similar in all cases, whereas the residues at both termini have different orientations (Figure 2C).

We computed MM/GBSA free energy using the trajectory of each cluster with the same GB method as the REMD simulations. Surprisingly, the MM/GBSA potential energy of the loop was 10 kcal/mol lower than that of the most populated cluster with a helical structure (−985.1 and −975.1 kcal/mol, respectively), although the helix was much more abundant in the REMD simulations. The electrostatic part of the solvation free energy cannot be at the origin of this discrepancy, as the same GB method is used in all calculations. The non-electrostatic part of the solvation free energy is not taken into account during REMD simulations. But it favors the loop only by 1.3 kcal/mol (14.7 and 13.4 kcal/mol for the helix and loop, respectively). One may expect that the helix is stabilized by entropy. Indeed, adding the entropy contributions using normal mode approximation reduces the free energy difference to 6 kcal/mol. The remaining error can be attributed to normal mode approximation. For instance, this method is known to underestimate the entropy of internal rotations. Such movements are expected to be less pronounced in the loop, which displays a network of salt bridges. Thus, the entropy contribution should be more underestimated for the helix. We also computed quasiharmonic entropies, but the results were unreliable as they depended largely on the number of frames used for the calculation.

### 3.3 Structure of H1 in Complex with Various Hydrophilic Prion Regions

We investigated whether the abundance of the loop structure depended on its interactions with other regions of PrP. Because of the unusually high hydrophilic nature of H1, we focused on its possible interactions with the hydrophilic regions, that is, the sequences K^197^GENFTETDIKIMER^211^, H^99^SQWNKPSKPKTNMK^113^_,_ and another H1. The sequence K^25^KRPK^29^ was not considered as it was not part of the misfolded PrP^Sc^ core.

In the simulation with two H1, a complex of two undistorted helices had an abundance of 78.4% (Figure 3A). Helices were arranged in a parallel manner, and D147^A^-R151^B^, D150^A^-R151^B^, R151^A^-D147^B^, R154^A^-E155^B^, R159^A^-D147^B^, and R159^A^-D150^B^ salt bridges were formed (A and B denote different H1s). Some intrahelix salt bridges were also maintained (D147^A^-R151^A^, D150^A^-R154^A^, R151^A^-E155^A^, E155^A^-R159^A^, D147^B^-R151^B^, and D150^B^-R154^B^). Several clusters with partially unfolded helices displayed a total abundance of 18% (not shown). Completely unfolded structures, different than the loop described above, were observed only in 3.6% of the simulation (Figure 3B). In all complexes, a network of intermolecular and intramolecular salt bridges was observed.

When H1 was allowed to form a complex with the sequence K^197^GENFTETDIKIMER^211^, the total abundance of the clusters containing helical H1 structures was 95%. The most abundant cluster (86%) shown in Figure 4A displayed several salt bridges between H1 and K^197^GENFTETDIKIMER^211^ (R154-E199, R151-D205, R159-E210, D147-R211). In addition, intrahelix D147-R151, D150-R154, and D150-R159 salt bridges were observed. The K^197^GENFTETDIKIMER^211^ moiety was also stabilized by internal salt bridges (E210-R211, D205-R211). The other clusters with a helical H1 also exhibited a high number of salt bridges within H1 and between H1 and K^197^GENFTETDIKIMER^211^, although the amino acids involved were different (not shown).

The remaining 5% consisted of several unfolded structures. Most of them were extended structures different from the loop presented above (Figure 4B). Only one cluster (0.3%) displayed a structure similar to this loop (Figure 4C). Residues D150-Y160 of H1 were aligned with the most stable loop conformation with an rmsd of 1.89 Å. This structure was unique in that only one salt bridge (R159-E203) was found between the H1 and K^197^GENFTETDIKIMER^211^ moieties, and its occurrence is only 43.5% (Table S4). Instead of salt bridges, hydrogen bonds and hydrophobic contacts held the two sequences together. However, a visual inspection of the cluster members and Table S4 indicated that the amino acids involved in these interactions varied within the cluster. D147-R154, R154-E155, E155-R159, and D150-R151 salt bridges stabilized the loop conformation interchangeably with E149-R154, E149-R159, and D147-R151 salt bridges (Figure 4D, Table S4).

The situation was completely different when H1 interacted with the H^99^SQWNKPSKPKTNMK^113^ sequence. The total abundance of the loop structures, distributed in two clusters, was 96%. In the most abundant structure (92%), the H1 moiety overlaps with the most stable loop conformation described above with an rmsd of 0.79 Å. No salt bridge was found between the two moieties. Several hydrogen bonds and hydrophobic contacts stabilized the complex; however, a visual inspection and occurrences in Table S5 showed that the amino acids involved in these interactions varied within the cluster. D147-R154, E149-R154, E149-R151, D147-R159, E149-R159, and E155-R151 salt bridges maintained the loop structure (Figure 5C, Table S5). The second most populated cluster (4%) contained a loop structure somewhat distorted at both ends. Nevertheless, the central part of H1 was similar in both complexes (sequence D150-E155 of the two complexes aligned with an rmsd 2 Å). E149-R154, E149-R151, E149-R159, and E155-R151 salt bridges, and to a lesser extent, D147-R154 and D147-R159 salt bridges, stabilized the loop structure (Figure 5D, Table S6). A variable set of hydrogen bonds and hydrophobic interactions between the two moieties was observed (Table S6). Unlike the most abundant complex, the K104-D147 salt bridge was formed. The remaining clusters contain helical H1 with limited or no interactions with H^99^SQWNKPSKPKTNMK^113^ (not shown).

The MM/GBSA free energy difference between the loop and helical structures using the trajectories of the most populated clusters containing a loop and a helix is 16.5 kcal/mol (*G*_loop_ = −1626.3 kcal/mol, *G*_helix_ = −1609.8 kcal/mol). Probably, this value is highly overestimated due to the inherent problems in normal mode entropy calculations. The entropy contribution is 8.7 kcal/mol in favor of the helix, in agreement with the results obtained for H1 alone. At least qualitatively, our results show that, both alone and in the complex, the loop is favored by the potential energy, whereas the helix is favored by the entropy. For H1 alone, the entropic contribution dominates, whereas the interactions in the complex lower the potential energy of the loop conformation to overcome the entropy effects.

Here, the interaction partners of H1 were taken as completely flexible. Of course, in PrP^Sc^, they acquire specific conformations. Nevertheless, one may assume that if H1s interact only with each other in an oligomer, they all fold in the same way, rather independent of the rest of the oligomer, like in our simulation. According to H/D exchange experiments, H^99^SQWNKPSKPKTNMK^113^ is in a highly accessible region of PrP^Sc^.^7−9^ Not being on a helix or β-strand, it is reasonable to hypothesize that this sequence would largely fold together with H1 if they interact with each other, as in our simulation. On the other hand, the sequence K^197^GENFTETDIKIMER^211^ is on a highly protected region of PrP^Sc^, most likely on a β-strand.^7−9^ None of the structures seen in our simulation display a β-strand. Thus, this particular simulation is informative only about the effect of salt bridges on the conformational preference of H1.

### 3.4 Effect of the Distance Between the N- and C-Termini of H1

The geometric restriction imposed on H1 by covalent bonds to the rest of the protein can be mimicked by putting a distance restraint between the residues at the termini of H1. In the simulations described above, the distance between the α-carbons of N146 and Y160 is ≈20 Å in the helix and ≈10 Å in the loop. When this distance is restrained to be ≤9 Å, the abundance of the loop rises to 76%. The remaining 24% is made of distorted helical conformations (structures not shown).

### 3.5 Comparison with Mutation Experiments

Norstrom and Mastrianni^27^ mutated charged amino acids on H1 to similarly charged, oppositely charged, or uncharged amino acids in mouse prion (for the sake of comparison, ovine PrP numbering will be used below). In these experiments, D147E, D147K, D147A, E149D, E149A, D150E, D150K, and D150R mutants retained their ability to convert into PrP^Sc^. D150A displayed a different pattern of proteinase K-resistance than the wild type. Among the mutations at the N-terminus of H1, only D147R, E149K, and E149R exhibited impaired ability of conversion. On the other hand, all mutations at the C-terminal part of H1 concerning R151, R154, and E155 led to impaired or completely blocked conversion, with the exception of R151K.

In our simulations, the R151-E155 salt bridge, a crucial interaction stabilizing the central part of the loop, was present in almost all loop structures (Figures 1C, 2D,E, 5C,D, Tables S2, S3, S5 and S6). The two acidic residues, D147 and E149, at the N-terminus of the loop can substitute each other in salt bridges. For instance, it can be seen from Figure 5D that the D147-R154 and D147-R159 interactions are interchangeable with the E149-R154 and E149-R159 interactions. Similarly, according to Figures 2D,E, the D147-R151 and E149-R151 salt bridges can replace each other. Figure 2D also shows that the E149-R159 and D150-R159 interactions are interchangeable with the E155-R159 interaction. In addition, the occurrence of the E149-R154 salt bridge is less than that of the others in Figure 5C (Table S5). All together, these observations suggest that the N-terminal region of the loop is relatively more flexible and negatively charged residues at this region can substitute each other in salt bridges. On the other hand, R151, R154, and E155 are more persistent in salt bridges and are not substituted by other residues. These observations are consistent with mutation experiments.

In the same study, positively and negatively charged residues in each salt bridge in the helical structure were exchanged. Although the salt bridges in the helical structure were conserved in these double mutants, they did not convert to PrP^Sc^.^27^ The authors concluded that not the salt bridges but the charge structure of H1 was critical for PrP^Sc^ generation.^27^ Our results show that the failure of the double mutants to convert to PrP^Sc^ arises from the fact that the salt bridges in the helix and loop structures are different. Therefore, while the double mutants maintain the salt bridges in the helix form, they cannot form the necessary salt bridges in the loop. For instance, double mutants have either D147R-R151D or D150R-R154D mutations or both. These mutants are not capable of forming crucial interactions in the loop such as the R151-E155 salt bridge.

### 3.6 Protonation of Aspartic and Glutamic Acids

**Figure 6:**
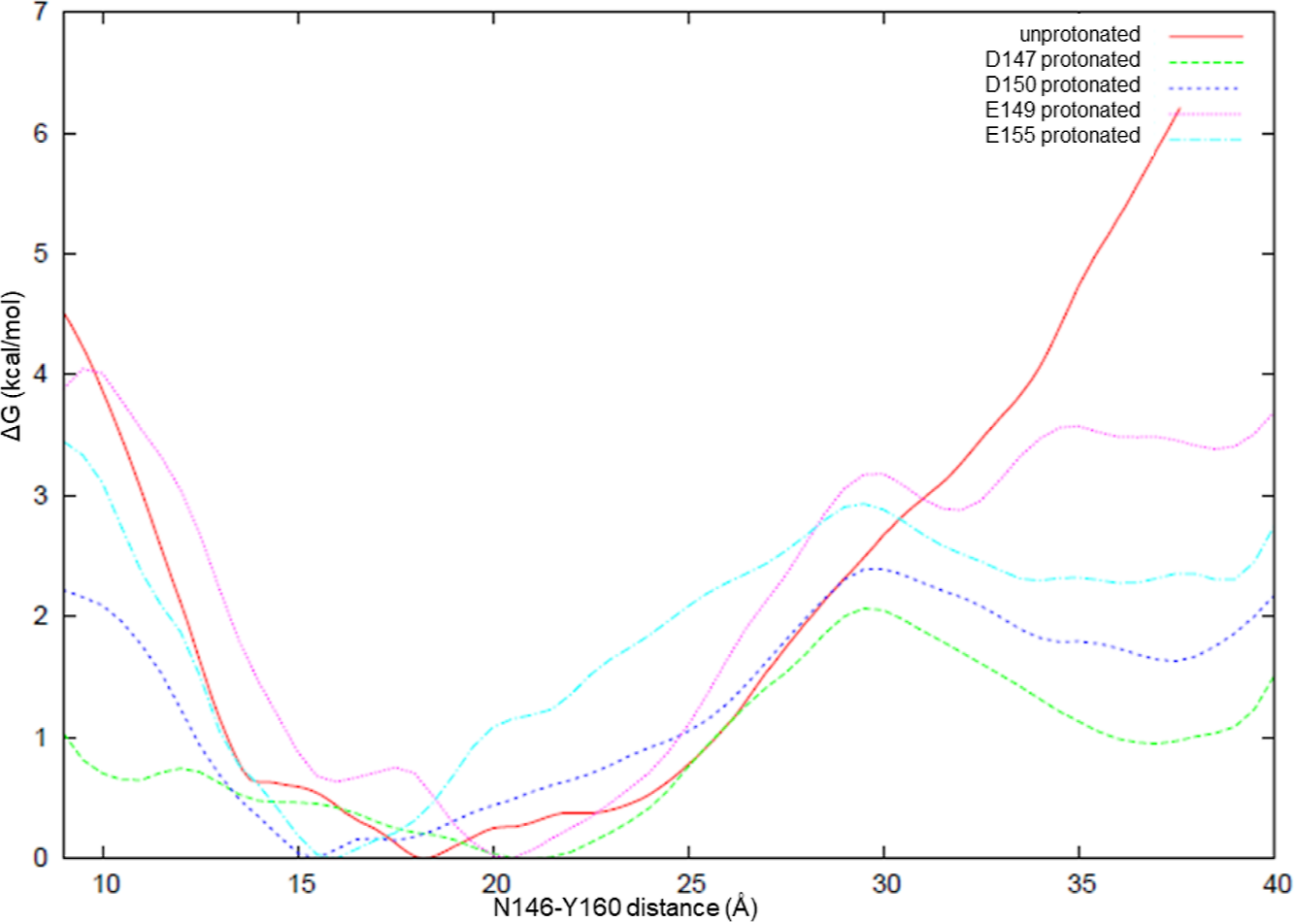
Free energy profiles of the unprotonated, D147 protonated, D150 protonated, E149 protonated, and E155 protonated H1.

Some studies in the literature considered the protonation of aspartic and glutamic acid residues on H1 as a sign that conversion to PrP^Sc^ was favored in the acidic medium of endosomes. Such a protonation is clearly incompatible with our results since it would break salt bridges. Nevertheless, it can be assumed that a temporary protonation of H1 may lower the free energy barrier for the unfolding of the helical structure. To investigate this hypothesis, we performed umbrella sampling simulations to calculate the potential of mean force on the structures protonated at D147, E149, D150, or E155, as well as the unprotonated H1. The distance between the two ends of H1, represented by the Cα atoms of N146 and Y160, was scanned. The results are given in Figure 6. Clearly, all protonated structures lie in shallow and broad free energy wells and can be stretched more easily than the unprotonated one.

## 4 Discussion

All simulations performed in this study and their outcomes are listed in Table 1.

**Table 1:** Simulations Performed in This Study, Simulation Methods, and Their Outcomes

simulated system	simulation method	outcome
H1 + H1B1	REMD	96% helix,4% loop
isolated H1	REMD	99.2% helix, 0.8% loop
H1 dimer	REMD	96.4% helix, 3.6% unfolded
H1 + K^197^GENFTETDIKIMER^211^	REMD	95% helix, 0.3% loop, 4.7% unfolded
H^1^ + H^99^SQWNKPSKPKTNMK^113^	REMD	4% helix, 96% loop
restrained H1	REMD	24% helix, 76% loop
H1 with ionized aspartates and glutamates	Umbrella sampling	deep free energy well
H1 with protonated D147	Umbrella sampling	shallow free energy well
H1 with protonated D150	Umbrella sampling	shallow free energy well
H1 with protonated E149	Umbrella sampling	shallow free energy well
H1 with protonated E155	Umbrella sampling	shallow free energy well

REMD simulations have been performed on the prion sequence 146–160, corresponding to helix 1 in the PrP^C^ form. This sequence (referred to as H1) adopts two conformations: a helical conformation (99.2%) as observed in PrP^C^ and a loop conformation (0.8%). The helical structure is stabilized by intrahelix salt bridges in addition to the usual backbone hydrogen bonds, as observed in PrP^C^ crystals. The loop is stabilized mainly by a network of salt bridges.

Although the loop is a negligible conformation in isolated H1, its abundance rises to 96% in the complex with the H^99^SQWNKPSKPKTNMK^113^ sequence. Conversely, the interaction of H1 with other highly hydrophilic sequences, that is, K^197^GENFTETDIKIMER^211^ or another H1 (representing H1 of an adjacent monomer) completely suppresses loop formation. While H^99^SQWNKPSKPKTNMK^113^ contains only positively charged or neutral amino acids, the other two sequences have both negatively and positively charged residues. Thus, when the charged residues on these two sequences interact with the pairs of residues forming a salt bridge on H1, they can interact with both negative and positive charges. However, the interaction of the positively charged H^99^SQWNKPSKPKTNMK^113^ with the negative charges on H1 is likely to be blocked by the positive charges on H1 salt bridges. As a result, the latter sequence binds to H1 mainly via hydrogen bonds, whereas the other two sequences form a network of salt bridges with H1. This difference in binding pattern seems to determine the helix/loop preference. In the helical form, charged amino acids are situated at the surface of H1. They can be reoriented to form salt bridges with another sequence without disturbing the intrahelix ones. However, the network of internal salt bridges responsible for the stability of the loop structure is broken upon salt bridge formation with another sequence, abolishing this conformation. This idea is further supported by the fact that the only H1-K^197^GENFTETDIKIMER^211^ complex with a loop conformation of H1 has just one salt bridge between the two moieties. Other hydrophilic sequences not considered in the present study are also close to oppositely charged amino acids. Hence, residues R167, D170, and R171 near the N-terminus of H2; residues K188, K197, and E199 near the C-terminus of H2; and residues R223 and E224 would lead to an interaction pattern with H1 that would favor a helical conformation.

Since the loop is stabilized by hydrogen bonds and hydrophobic interactions, one may think that the backbone of a mostly hydrophobic prion sequence can also provide these hydrogen bonds along with the side chains involved in hydrophobic contacts. However, the highly hydrophilic composition of H1 seems to prevent such interactions. For instance, the interactions between H1 and mostly hydrophobic H1B1 do not stabilize the loop significantly.

A significant amount of helical conformation exists even when the end-to-end distance of H1 is restrained to a small value. This indicates that anchoring the termini of H1 at a short distance to each other by covalent bonds to the rest of the protein is not enough to completely prevent helix formation in PrP^Sc^.

Interestingly, the average potential energy of the loop conformation is lower than that of the helix, although the helix is by far more abundant, suggesting that the helix is stabilized by the entropic contribution. In the helix, the salt bridges can be broken temporarily, making the side chains involved more mobile. On the other hand, the loop geometry strictly relies on the network of salt bridges. Thus, the positions and fluctuations of the charged side chains in the helix are significantly less restricted compared to the ones in the loop, probably leading to a higher entropy.

H1 is experimentally known to form a helix in the misfolded human PrP(23–159), which contains only the N-terminal region of prion together with H1.^18^ According to cryo-EM results, the region between residues 106 and 145 consists of an in-register β-sheet structure.^19^ Although the conformation of H1 is not resolved in this experiment, the in-register β-sheet arrangement should obviously locate H1 sequences of adjacent protomers next to each other, leading to helical structures consistent with our simulations.

On the other hand, H1 appears to be mostly^8^ or completely^9^ unprotected in H/D exchange experiments in full-length PrP^Sc^ oligomers. Our results indicate that when two H1s are allowed to interact with each other, helical structures form. Taking together, at least in the protomers where H1 is unprotected, this region must be located away from the H1 sequence of an adjacent protomer. Moreover, H1 must interact with the H^99^SQWNKPSKPKTNMK^113^ sequence from the same or a different protomer.

**Figure 7:**
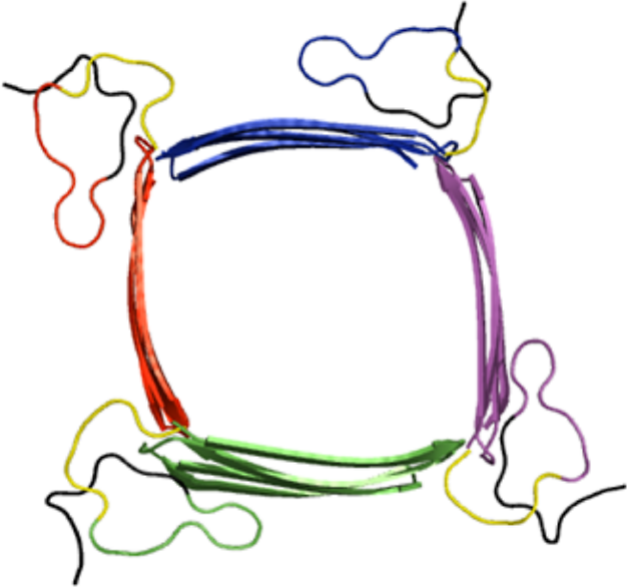
Putative structure of a PrP^Sc^ oligomer consistent with our results. The number of protomers and the arrangement of the β-sheet core are for illustration purposes only. Green, red, blue, and magenta indicate diferent protomers. H1 and H^99^SQWNKPSKPKTNMK^113^ are shown in yellow and black, respectively.

Since in different truncated prion proteins both the sequence N-terminal to H1^18,19^ and a sequence containing only H2 and H3^5^ are capable of forming β-rich misfolded structures, we hypothesize that both sequences can fold into β-sheets independently and H1 acts as a linker between them. A possible arrangement consistent with our results and experiments is given in Figure 7. The number of protomers and the arrangement of the β-sheet core are for illustration purposes only. Cryo-EM experiments suggest that prion fibrils consist of two protofilaments.^38^ In such a case, N-terminal regions of the protomers at the interface between two protofilaments may interact with each other and form a β-sheet. The N-terminal regions of the other protomers may form loops (or partially disordered structures) such that H^99^SQWNKPSKPKTNMK^113^ is positioned next to H1. This would explain why the N-terminal region and H1 are mostly unprotected in H/D exchange experiments, while a fraction of protected protomers exist. Alternatively, the N-terminal region and H1 of one protomer may be wound on the β-sheet core of several other protomers.

Protonation of glutamic acid and aspartic acid residues on H1 makes the helical conformation more flexible, thus more prone to unfold. But, in the loop form, these amino acids are not likely to be protonated.

## Supporting Information Available

Plots of the appearance of clusters with respect to time and root mean square deviations of Cα atoms of cluster members with respect to the cluster representatives; percentages of clusters at different time intervals and occurrences of salt bridges and hydrogen bonds in the clusters with a loop structure; examples of input files for REMD and umbrella sampling simulations and clustering analysis; and summary files of cluster analyzes and coordinates of the representatives of all clusters in pdb format (PDF)

## Notes

Data and Software Availability: Simulations were performed using Amber16, and results were analyzed using the cpptraj tool in AmberTools. The GPU version of the pmemd program which is part of the Amber16 package can be purchased from: https://ambermd.org/GetAmber.php. However, a CPU version (sander) that uses the same algorithms is part of AmberTools. AmberTools can be obtained free of charge from: https://ambermd.org/AmberTools.php. The WHAM code used for umbrella sampling calculations can be downloaded free of charge from Grossfield Lab website: http://membrane.urmc.rochester.edu/?page_id=126. Examples of input files for REMD and umbrella sampling simulations and clustering analysis are provided as Supporting Informations. Summary files of cluster analyses and coordinates of the representatives of all clusters are given as Supporting Informations.

## Abbreviations

PrP,: prion protein
PrP^C^,: cellular form of prion protein
PrP^Sc^,: scrapie form of prion protein
REMD,: replica exchange molecular dynamics
MM/GBSA,: molecular mechanics generalized Born surface area
