# Supplementary material for: Conversion of Helix 1 into a Loop in Prion Protein Misfolding
Source: ACS Omega. 2023 Feb 10;8(7):7191–200. doi: 10.1021/acsomega.3c00212 (PMC9948551; doi:10.1021/acsomega.3c00212)
Supplement: Supplementary file 1 — ao3c00212_si_001.pdf [file ao3c00212_si_001.pdf]

Supporting Informations

Conversion of Helix 1 into a Loop in Prion Protein Misfolding

*Ayşenaz Tavşanlı, Bülent Balta**

Department of Molecular Biology and Genetics, Istanbul Technical University, Maslak 34469, Istanbul, Turkey

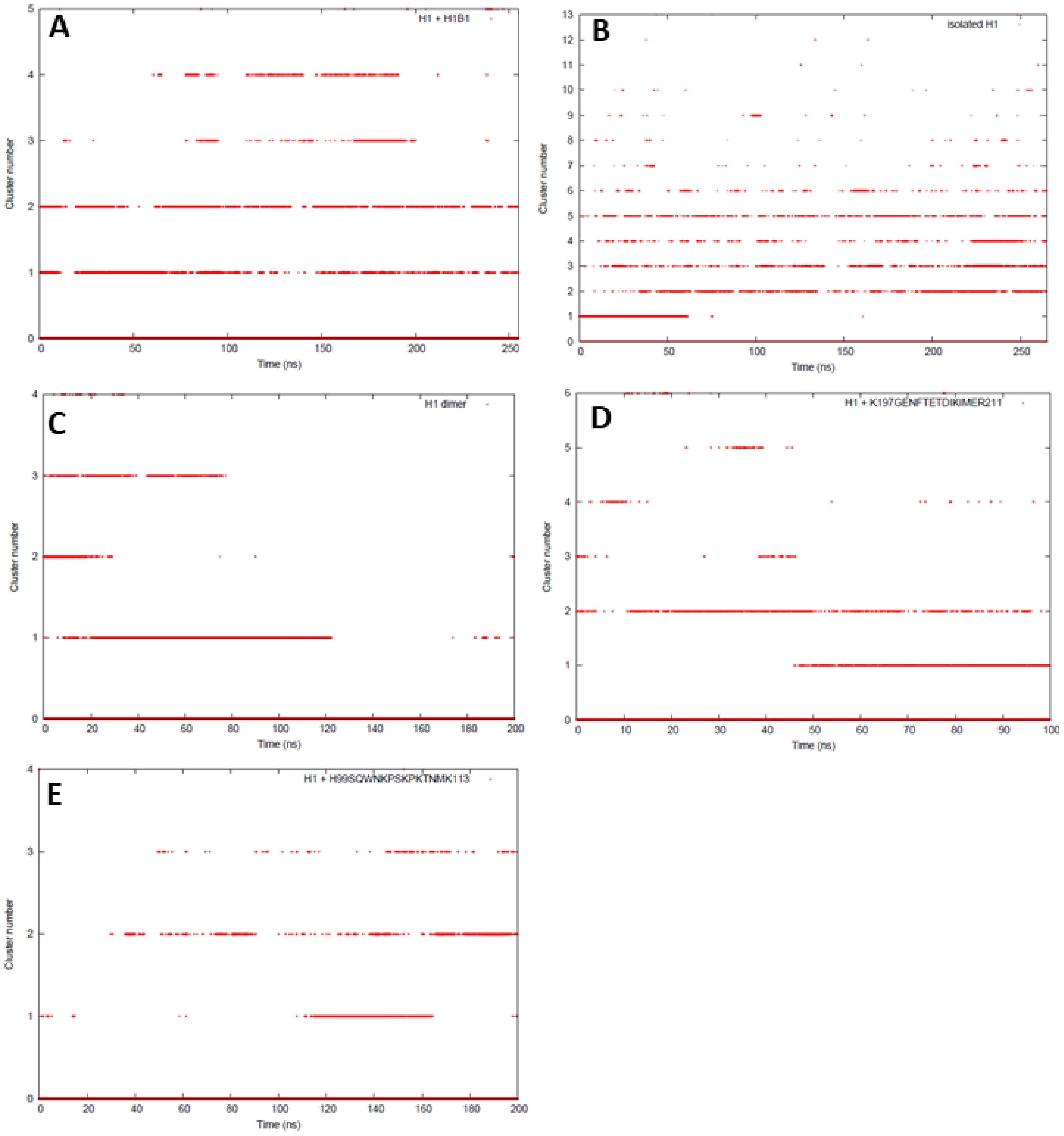

Figure S1: Appearance of clusters with respect to time. A) H1+H1B1, B) isolated H1, C) H1 dimer, D) H1- K^{197}GENFTETDIKIMER^{211}, E) H1-H^{99}SQWNKPSKPKTNMK^{113} simulations

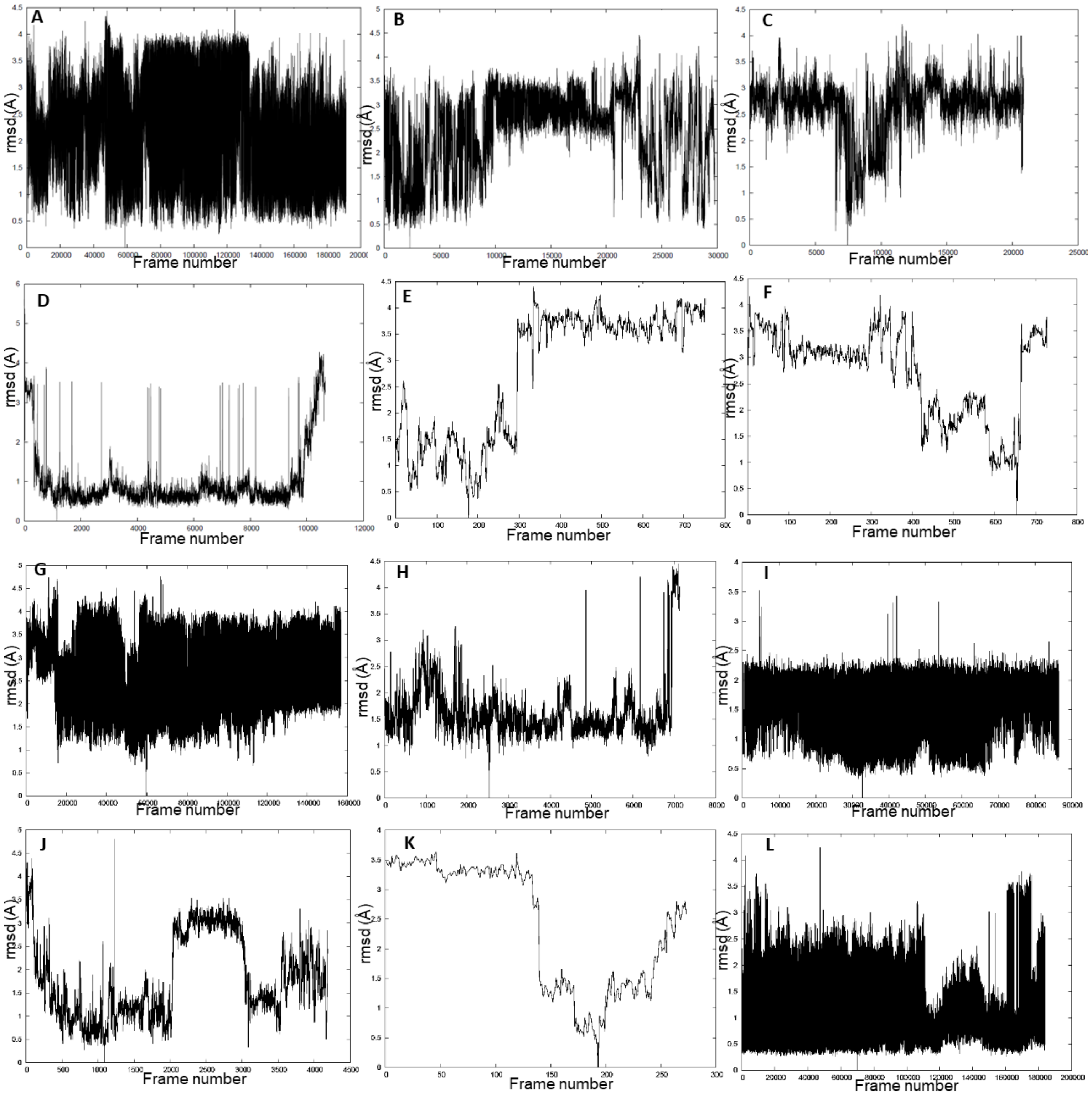
A
B
C
D
E
F
G
H
I
J
K
L
rmsd (Å)
Frame number

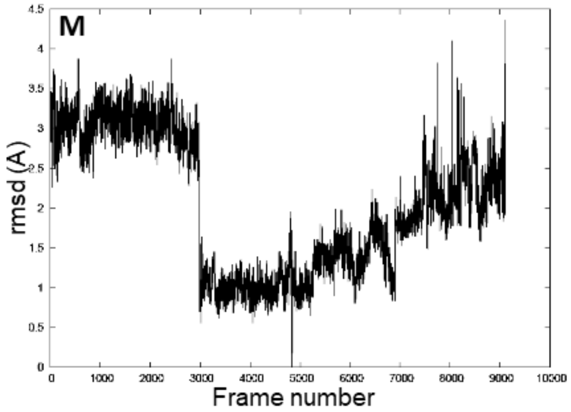

Figure S2: Root mean square deviations of Cα atoms of cluster members with respect to the cluster representatives. A-C) Clusters with representatives in Figure 1A (green, blue, brown, respectively), D) cluster with the representative in Figure 1B, E) cluster with the representative in Figure 2A, F) cluster with the representative in Figure 2B, G) cluster with the representative in Figure 3A, H) cluster with the representative in Figure 3B, I) cluster with the representative in Figure 4A, J) cluster with the representative in Figure 4B, K) cluster with the representative in Figure 4C, L) cluster with the representative in Figure 5A, M) cluster with the representative in Figure 5B

Table S1. Percentages of the clusters at different time intervals

	H1+H1B1			isolated H1			H1 dimer			H1+(197-211)			H1+(99-113)		
cluster number	0-255 ns	60-255 ns	155-255 ns	0-265 ns	60-265 ns	160-265 ns	0-200 ns	60-200 ns	130-200 ns	0-100 ns	60-100 ns	80-100 ns	0-200 ns	60-200 ns	130-200 ns
0	75.0	80.4	80.3	72.5	80.1	73.6	78.4	86.3	99.7	86.4	78.6	73.4	91.9	88.7	84.7
1	11.7	5.4	4.3	9.0	0.2	0	15.4	12.7	0.3	8.6	19.0	24.7	4.6	6.4	7
2	8.2	7.6	5.4	7.3	7.9	9.9	3.6	0	0	4.2	2.2	1.9	3.4	4.6	7.9
3	4.2	5.4	9.8	3.2	3.7	5.4	2.6	1	0	0.3	0	0	0.2	0.3	0.4
4	0.9	1.2	0.3	2.8	2.9	4.7	0.1	0	0	0.3	0.1	0.1	0	0	0
5	0	0	0	2.8	3.0	4.3				0.2	0	0			
6				1.5	1.5	1.2				0.1	0.1	0			
7				0.4	0.3	0.5									
8				0.2	0.2	0.4									
9				0.2	0.2	0									
10				0	0	0									
11				0	0	0									
12				0	0	0									
13				0	0	0									

Table S2. Occurrences of salt bridges and hydrogen bonds in the loop structure shown in Figure 1B (only hydrogen bond occurrences higher than 5% are listed)

	Interaction	Occurrence (%)
salt bridges within the loop	D147-R151<br>D147-R154<br>E149-R154<br>E155-R151<br>E155-R159	88.5<br>98.5<br>92.2<br>95.8<br>92.0
salt bridge between the loop and H1B1	E149-R139	83.9
hydrogen bonds within the loop	Y158CO-R159NηH<br>N156CO-R159NηH<br>E155Oε-E155NH<br>M157CO-R139NηH<br>E155Oε-N156NH<br>N156Oδ-Y158NH	36.2<br>32.7<br>29.7<br>16.1<br>11.9<br>10.6
hydrogen bonds between the loop and H1B1	R139NηH-D147CO<br>H143NδH-D147CO	26.4<br>5.8

Table S3. Occurrences of salt bridges and hydrogen bonds in the loop structures shown in Figure 2A and 2B (only hydrogen bond occurrences higher than 5% are listed)

	Interaction	Occurrence (%)
salt bridges in the structure in Figure 2A	D147-R151	52.5
	D147-R154	70.6
	D147-R159	13.3
	D150-R159	43.6
	E149-R151	46.5
	E149-R154	66.6
	E149-R159	44.9
	E155-R151	83.1
	E155-R159	47.7
hydrogen bonds in the structure in Figure 2A	E155Oε-E155NH	19.6
	N146CO-N156NδH	15.0
	D147Oδ-N156NH	12.9
	R151CO-E155NH	12.5
	R151CO-R154NH	10.5
	R151CO-R151NηH	8.6
	E149Oε-D150NH	7.3
	D147Oδ-E155NH	7.2
	D147Oδ-Y160OH	6.1
	D147Oδ-R159NH	6.1
	E155CO-M157NH	6.4
	Y153CO-E155NH	5.1
	E155Oε-N156NH	5.1
	N156CO-R159NH	5.1
salt bridges in the structure in Figure 2B	D147-R151	47.2
	D147-R154	54.5
	D147-R159	29.9
	D150-R159	11.7
	E149-R151	49.9
	E149-R154	50.6
	E149-R159	0.3
	E155-R151	80.7
	E155-R159	37.4
hydrogen bonds in the structure in Figure 2B	E155Oε-E155NH	20.1
	R151CO-E155NH	19.6
	R159CO-R154NηH	18.0
	R151CO-R154NH	16.6
	D147Oδ-Y160OH	13.5
	R151CO-Y153NH	12.6
	M157CO-Y160NH	8.8
	N156Oδ-Y158NH	8.8
	E155Oδ-N156NH	8.5
	Y148CO-R151NH	7.4
	E149Oε- R151NH	6.7
	Y152CO-Y160OH	5.8
	Y158CO-R159NεH	5.6
	E155CO-Y158NH	5.0

Table S4. Occurrences of salt bridges and hydrogen bonds in the loop structure shown in Figure 4C (only hydrogen bond occurrences higher than 5% are listed)

	Interaction	Occurrence (%)
salt bridges within the loop	D147-R151	43.9
	D147-R154	49.1
	D150-R151	38.8
	E149-R154	49.4
	E149-R159	51.6
	E155-R159	43.2
	R154-E155	49.1
salt bridge between the loop and (197-211)	R159-E203	43.5
hydrogen bonds within the loop	E149CO-Y152NH	36.6
	E149CO-Y153NH	35.2
	M157CO-Y153OH	34.1
	E155Oε-R151NH	32.2
	N156CO-R159NηH	23.8
	E155Oε-E155NH	22.0
	E149Oε-N146NδH	16.8
	N146CO-E149NH	15.4
	N146CO-D150NH	9.9
	Y148CO-Y152NH	9.5
	E155Oε-R154NH	9.2
	E155Oε-E155NH	7.3
	R151CO-M157NH	7.3
	N146Oδ-Y148NH	5.9
hydrogen bonds between the loop and (197-211)	T202Oγ-Y148OH	18.7
	E210Oε-Y160OH	16.5
	R211CO-N156NH	15.4
	D147Oδ-T202NH	5.9
	N200Oδ-N146NδH	14.3
	N200CO-D147NH	11.7

Table S5. Occurrences of salt bridges and hydrogen bonds in the loop structure shown in Figure 5A (only hydrogen bond occurrences higher than 5% are listed)

	Interaction	Occurrence (%)
salt bridges within the loop	D147-R154	99.3
	D147-R159	80.2
	E149-R151	99.2
	E149-R154	66.9
	E149-R159	95.1
	E155-R151	99.2
hydrogen bonds within the loop	E155Oε-E155NH	34.3
	E155CO-M157NH	31.4
	E155Oε-E155NH	31.1
	M157CO-R154NηH	28.9
	D147Oδ-R159NH	17.0
	E149Oε-D150NH	16.8
	E155CO-R159NεH	9.0
	E149Oε-R151NH	7.1
	N146Oδ-D147NH	5.3
hydrogen bonds between the loop and (99-113)	P108CO-R159NηH	15.7
	M157CO-N111NδH	13.9
	Y158CO-K113NH	13.2
	T110CO-R159NηH	13.1
	T110OH-R159NεH	8.6
	M157CO-Q101NεH	8.4
	N111Oδ-R151NηH	7.5
	E149Oε-T110OH	6.4
	P108CO-R159NεH	6.4
	N111CO-Y160NH	6.2
	K113CO-Y158NH	5.8
	K107CO-R159NηH	5.7
	Y158CO-W102NH	5.5

Table S6. Occurrences of salt bridges and hydrogen bonds in the loop structure shown in Figure 5B (only hydrogen bond occurrences higher than 5% are listed)

	Interaction	Occurrence (%)
salt bridges within the loop	D147-R154	18.4
	D147-R159	18.3
	E149-R151	49.8
	E149-R154	73.3
	E149-R159	81.9
	E155-R151	99.9
hydrogen bonds within the loop	E155CO-R159NηH	47.8
	Y148CO-R151NεH	37.0
	E155Oε-E155NH	27.2
	R151CO-R154NηH	25.5
	Y153CO-R154NηH	25.0
	E149CO-R154NηH	18.8
	E149CO-R151NH	17.6
	Y148CO-R151NηH	11.6
	D147CO-R151NεH	8.2
	Y148CO-R154NηH	7.0
	D147Oδ-E149NH	5.7
	M157CO-N146NδH	5.5
	R151CO-R154NεH	5.5
	Y153CO-R151NηH	5.4
hydrogen bonds between the loop and (99-113)	M157CO-N103NδH	20.6
	W102CO-Y160NH	14.4
	K107CO-Y160OH	10.4
	Y160CO-W102NH	9.1
	Q101CO-R159NηH	8.7
	H99CO-R154NηH	8.2
	S106CO-R151NηH	8.2
	N103Oδ-N146NδH	6.3
	E149Oδ-N103NδH	5.1
	H99CO-R154NεH	9.5
	N146CO-N103NδH	8.7

Input file for REMD simulation (the restraint file chirrest.dat was obtained by using makeCHIR_RST utility of AmberTools on the pdb file). All replicas use a similar input file with the exception of the temperature determined by the temp0 keyword.

```
 &cntrl
  irest=1, ntx=5,
  imin = 0, cut = 9999, ntb = 0,
  nstlim = 1000, dt = 0.001, ntt = 3,
  gamma_ln = 5.0, ig=-1, numexchg = 5000,
  temp0 = 294.0,
  ntc = 2, ntf = 2,
  ntp = 0,
  ntwx = 1000, ntwe = 1000, ntpr = 100,
  igb=5, saltcon=0.1,
  nmropt = 1,
 /
 &wt TYPE='END'
 /
DISANG=chirrest.dat
```

Example of a script used for clustering with the cpptraj utility:

```
trajin remd.mdcrd.001 remdtraj remdtrajtemp 310.0
rms first @CA
cluster h1remd rms @CA mass epsilon 4 out h1remdclus summary summary.dat info info.dat sieve 50 repout h1remdrep repfmt pdb
```

Example of an input file used for potential of mean force umbrella sampling calculations:

```
 &cntrl
  imin = 0, ntb = 0, cut = 30, igb = 5, saltcon = 0.1,
  nstlim = 25000000, dt = 0.002, ntc = 2, ntf = 2,
  ntt = 3, gamma_ln = 5.0, ig = -1, tempi = 20.0, temp0 = 310.0,
  ntp = 0,
  ntwx = 2500, ntwe = 2500, ntpr = 100,
  nmropt = 1,
 &end
 &wt
  type='DUMPFREQ', istep1=50,
 &end
 &wt
  type='END',
 &end
DISANG=1000.dat
DUMPAVE=h1-1000.dat
```

The restraint file 1000.dat:

```
 &rst
  iat=9,264,
  r1=1, r2=10.00, r3=10.00, r4=90,
  rk2=0.5, rk3=0.5,
 &end
```

Results of the clustering analyzes and coordinates of the representatives of all clusters in pdb format (the structures given in Figures 1-5 are indicated):

H1+H1B1 simulation:

#Cluster	Frames	Frac	AvgDist	Stdev	Centroid	AvgCDist
0	191332	0.750	2.883	0.992	213256	4.525
1	29787	0.117	3.076	1.156	6509	4.722
2	20851	0.082	2.578	0.798	207857	5.304
3	10649	0.042	1.188	0.678	178245	5.635
4	2258	0.009	1.062	0.219	82899	4.978
5	123	0.000	3.249	0.000	85891	4.714

Cluster 0, Figure 1A (green):

```
ATOM      1 HH31 ACE     1       3.117  14.794  -4.651  1.00  0.00           H
ATOM      2  CH3 ACE     1       3.052  13.795  -5.083  1.00  0.00           C
ATOM      3 HH32 ACE     1       3.531  13.959  -6.048  1.00  0.00           H
ATOM      4 HH33 ACE     1       2.018  13.479  -5.223  1.00  0.00           H
ATOM      5  C   ACE     1       3.769  12.899  -4.139  1.00  0.00           C
ATOM      6  O   ACE     1       3.340  12.777  -2.994  1.00  0.00           O
ATOM      7  N   VAL     2       4.947  12.374  -4.547  1.00  0.00           N
ATOM      8  H   VAL     2       5.115  12.450  -5.540  1.00  0.00           H
ATOM      9  CA  VAL     2       6.022  11.825  -3.711  1.00  0.00           C
ATOM     10  HA  VAL     2       5.683  10.878  -3.291  1.00  0.00           H
ATOM     11  CB  VAL     2       7.238  11.506  -4.654  1.00  0.00           C
ATOM     12  HB  VAL     2       7.966  10.934  -4.079  1.00  0.00           H
ATOM     13  CG1 VAL     2       6.794  10.415  -5.646  1.00  0.00           C
ATOM     14 HG11 VAL     2       7.630  10.280  -6.333  1.00  0.00           H
ATOM     15 HG12 VAL     2       6.611   9.459  -5.154  1.00  0.00           H
ATOM     16 HG13 VAL     2       5.933  10.690  -6.253  1.00  0.00           H
ATOM     17  CG2 VAL     2       7.861  12.785  -5.283  1.00  0.00           C
ATOM     18 HG21 VAL     2       8.085  12.609  -6.335  1.00  0.00           H
ATOM     19 HG22 VAL     2       7.045  13.504  -5.342  1.00  0.00           H
ATOM     20 HG23 VAL     2       8.725  13.115  -4.705  1.00  0.00           H
ATOM     21  C   VAL     2       6.578  12.676  -2.563  1.00  0.00           C
ATOM     22  O   VAL     2       7.149  12.178  -1.627  1.00  0.00           O
ATOM     23  N   MET     3       6.323  14.035  -2.538  1.00  0.00           N
ATOM     24  H   MET     3       5.854  14.387  -3.360  1.00  0.00           H
ATOM     25  CA  MET     3       6.671  14.932  -1.517  1.00  0.00           C
ATOM     26  HA  MET     3       7.680  14.735  -1.157  1.00  0.00           H
ATOM     27  CB  MET     3       6.762  16.316  -2.241  1.00  0.00           C
ATOM     28  HB2 MET     3       5.920  16.459  -2.918  1.00  0.00           H
ATOM     29  HB3 MET     3       6.648  17.125  -1.519  1.00  0.00           H
ATOM     30  CG  MET     3       8.118  16.474  -3.107  1.00  0.00           C
ATOM     31  HG2 MET     3       8.965  16.377  -2.427  1.00  0.00           H
ATOM     32  HG3 MET     3       8.116  15.643  -3.811  1.00  0.00           H
ATOM     33  SD  MET     3       8.137  17.992  -4.099  1.00  0.00           S
ATOM     34  CE  MET     3       7.262  17.271  -5.587  1.00  0.00           C
ATOM     35  HE1 MET     3       6.284  16.860  -5.338  1.00  0.00           H
ATOM     36  HE2 MET     3       7.216  17.995  -6.401  1.00  0.00           H
ATOM     37  HE3 MET     3       7.863  16.424  -5.918  1.00  0.00           H
ATOM     38  C   MET     3       5.660  14.951  -0.299  1.00  0.00           C
ATOM     39  O   MET     3       6.004  15.475   0.748  1.00  0.00           O
ATOM     40  N   SER     4       4.486  14.315  -0.413  1.00  0.00           N
ATOM     41  H   SER     4       4.238  13.909  -1.304  1.00  0.00           H
ATOM     42  CA  SER     4       3.590  14.168   0.778  1.00  0.00           C
ATOM     43  HA  SER     4       3.472  15.154   1.227  1.00  0.00           H
ATOM     44  CB  SER     4       2.227  13.801   0.403  1.00  0.00           C
ATOM     45  HB2 SER     4       2.213  12.863  -0.152  1.00  0.00           H
```

ATOM	46	HB3	SER	4	1.675	13.649	1.330	1.00	0.00	H
ATOM	47	OG	SER	4	1.634	14.894	-0.335	1.00	0.00	O
ATOM	48	HG	SER	4	0.924	14.546	-0.879	1.00	0.00	H
ATOM	49	C	SER	4	4.113	13.096	1.755	1.00	0.00	C
ATOM	50	O	SER	4	4.931	12.292	1.326	1.00	0.00	O
ATOM	51	N	ARG	5	3.660	13.051	3.049	1.00	0.00	N
ATOM	52	H	ARG	5	2.859	13.603	3.319	1.00	0.00	H
ATOM	53	CA	ARG	5	4.025	11.993	4.045	1.00	0.00	C
ATOM	54	HA	ARG	5	4.637	11.253	3.530	1.00	0.00	H
ATOM	55	CB	ARG	5	4.823	12.633	5.221	1.00	0.00	C
ATOM	56	HB2	ARG	5	4.210	13.356	5.757	1.00	0.00	H
ATOM	57	HB3	ARG	5	5.137	11.862	5.925	1.00	0.00	H
ATOM	58	CG	ARG	5	6.054	13.346	4.647	1.00	0.00	C
ATOM	59	HG2	ARG	5	6.588	12.646	4.005	1.00	0.00	H
ATOM	60	HG3	ARG	5	5.950	14.179	3.953	1.00	0.00	H
ATOM	61	CD	ARG	5	6.946	13.692	5.881	1.00	0.00	C
ATOM	62	HD2	ARG	5	6.415	14.390	6.527	1.00	0.00	H
ATOM	63	HD3	ARG	5	7.194	12.773	6.411	1.00	0.00	H
ATOM	64	NE	ARG	5	8.242	14.285	5.413	1.00	0.00	N
ATOM	65	HE	ARG	5	8.958	13.618	5.164	1.00	0.00	H
ATOM	66	CZ	ARG	5	8.710	15.519	5.469	1.00	0.00	C
ATOM	67	NH1	ARG	5	8.042	16.520	6.027	1.00	0.00	N
ATOM	68	HH11	ARG	5	8.318	17.479	5.871	1.00	0.00	H
ATOM	69	HH12	ARG	5	7.055	16.466	6.235	1.00	0.00	H
ATOM	70	NH2	ARG	5	9.925	15.779	4.907	1.00	0.00	N
ATOM	71	HH21	ARG	5	10.248	16.722	4.742	1.00	0.00	H
ATOM	72	HH22	ARG	5	10.574	15.014	4.786	1.00	0.00	H
ATOM	73	C	ARG	5	2.741	11.244	4.536	1.00	0.00	C
ATOM	74	O	ARG	5	1.694	11.936	4.605	1.00	0.00	O
ATOM	75	N	PRO	6	2.712	9.951	4.899	1.00	0.00	N
ATOM	76	CD	PRO	6	3.744	9.029	4.714	1.00	0.00	C
ATOM	77	HD2	PRO	6	4.684	9.357	5.158	1.00	0.00	H
ATOM	78	HD3	PRO	6	3.800	8.979	3.627	1.00	0.00	H
ATOM	79	CG	PRO	6	3.295	7.661	5.255	1.00	0.00	C
ATOM	80	HG2	PRO	6	4.091	7.242	5.871	1.00	0.00	H
ATOM	81	HG3	PRO	6	2.980	7.060	4.403	1.00	0.00	H
ATOM	82	CB	PRO	6	2.208	8.015	6.188	1.00	0.00	C
ATOM	83	HB2	PRO	6	2.573	8.143	7.208	1.00	0.00	H
ATOM	84	HB3	PRO	6	1.461	7.223	6.152	1.00	0.00	H
ATOM	85	CA	PRO	6	1.594	9.228	5.583	1.00	0.00	C
ATOM	86	HA	PRO	6	0.905	8.910	4.801	1.00	0.00	H
ATOM	87	C	PRO	6	0.728	10.046	6.617	1.00	0.00	C
ATOM	88	O	PRO	6	-0.504	9.870	6.708	1.00	0.00	O
ATOM	89	N	LEU	7	1.371	10.937	7.347	1.00	0.00	N
ATOM	90	H	LEU	7	2.379	10.946	7.288	1.00	0.00	H
ATOM	91	CA	LEU	7	0.731	11.783	8.403	1.00	0.00	C
ATOM	92	HA	LEU	7	0.196	11.122	9.086	1.00	0.00	H
ATOM	93	CB	LEU	7	1.810	12.521	9.188	1.00	0.00	C
ATOM	94	HB2	LEU	7	2.567	11.784	9.453	1.00	0.00	H
ATOM	95	HB3	LEU	7	2.355	13.312	8.676	1.00	0.00	H
ATOM	96	CG	LEU	7	1.368	13.210	10.516	1.00	0.00	C
ATOM	97	HG	LEU	7	0.513	13.882	10.440	1.00	0.00	H
ATOM	98	CD1	LEU	7	0.967	12.148	11.504	1.00	0.00	C
ATOM	99	HD11	LEU	7	1.791	11.435	11.489	1.00	0.00	H
ATOM	100	HD12	LEU	7	1.000	12.582	12.503	1.00	0.00	H
ATOM	101	HD13	LEU	7	0.021	11.629	11.359	1.00	0.00	H
ATOM	102	CD2	LEU	7	2.565	14.083	11.103	1.00	0.00	C
ATOM	103	HD21	LEU	7	2.335	14.574	12.048	1.00	0.00	H
ATOM	104	HD22	LEU	7	3.456	13.466	11.215	1.00	0.00	H
ATOM	105	HD23	LEU	7	2.639	14.853	10.334	1.00	0.00	H
ATOM	106	C	LEU	7	-0.267	12.807	7.867	1.00	0.00	C
ATOM	107	O	LEU	7	-1.248	13.087	8.582	1.00	0.00	O
ATOM	108	N	ILE	8	0.029	13.405	6.702	1.00	0.00	N

ATOM	109	H	ILE	8	0.855	13.011	6.273	1.00	0.00	H
ATOM	110	CA	ILE	8	-0.569	14.619	6.032	1.00	0.00	C
ATOM	111	HA	ILE	8	-1.453	14.828	6.635	1.00	0.00	H
ATOM	112	CB	ILE	8	0.347	15.848	6.108	1.00	0.00	C
ATOM	113	HB	ILE	8	-0.118	16.568	5.433	1.00	0.00	H
ATOM	114	CG2	ILE	8	0.335	16.393	7.544	1.00	0.00	C
ATOM	115	HG21	ILE	8	-0.704	16.523	7.842	1.00	0.00	H
ATOM	116	HG22	ILE	8	0.939	15.833	8.258	1.00	0.00	H
ATOM	117	HG23	ILE	8	0.861	17.345	7.623	1.00	0.00	H
ATOM	118	CG1	ILE	8	1.747	15.670	5.548	1.00	0.00	C
ATOM	119	HG12	ILE	8	2.288	16.510	5.982	1.00	0.00	H
ATOM	120	HG13	ILE	8	2.270	14.763	5.852	1.00	0.00	H
ATOM	121	CD1	ILE	8	1.899	15.765	4.014	1.00	0.00	C
ATOM	122	HD11	ILE	8	1.675	16.792	3.725	1.00	0.00	H
ATOM	123	HD12	ILE	8	2.955	15.597	3.798	1.00	0.00	H
ATOM	124	HD13	ILE	8	1.248	15.006	3.583	1.00	0.00	H
ATOM	125	C	ILE	8	-1.225	14.355	4.630	1.00	0.00	C
ATOM	126	O	ILE	8	-1.611	15.277	3.888	1.00	0.00	O
ATOM	127	N	HID	9	-1.419	13.095	4.207	1.00	0.00	N
ATOM	128	H	HID	9	-1.061	12.430	4.878	1.00	0.00	H
ATOM	129	CA	HID	9	-2.154	12.534	3.087	1.00	0.00	C
ATOM	130	HA	HID	9	-2.802	13.353	2.776	1.00	0.00	H
ATOM	131	CB	HID	9	-1.240	12.413	1.815	1.00	0.00	C
ATOM	132	HB2	HID	9	-0.839	13.394	1.558	1.00	0.00	H
ATOM	133	HB3	HID	9	-0.382	11.761	1.975	1.00	0.00	H
ATOM	134	CG	HID	9	-1.949	11.975	0.559	1.00	0.00	C
ATOM	135	ND1	HID	9	-3.160	12.521	0.078	1.00	0.00	N
ATOM	136	HD1	HID	9	-3.830	13.093	0.571	1.00	0.00	H
ATOM	137	CE1	HID	9	-3.393	11.936	-1.095	1.00	0.00	C
ATOM	138	HE1	HID	9	-4.247	12.132	-1.725	1.00	0.00	H
ATOM	139	NE2	HID	9	-2.352	11.073	-1.395	1.00	0.00	N
ATOM	140	CD2	HID	9	-1.475	11.096	-0.333	1.00	0.00	C
ATOM	141	HD2	HID	9	-0.457	10.735	-0.329	1.00	0.00	H
ATOM	142	C	HID	9	-3.178	11.426	3.401	1.00	0.00	C
ATOM	143	O	HID	9	-2.857	10.438	4.048	1.00	0.00	O
ATOM	144	N	PHE	10	-4.377	11.576	2.832	1.00	0.00	N
ATOM	145	H	PHE	10	-4.537	12.349	2.203	1.00	0.00	H
ATOM	146	CA	PHE	10	-5.641	10.778	3.052	1.00	0.00	C
ATOM	147	HA	PHE	10	-5.491	9.919	3.707	1.00	0.00	H
ATOM	148	CB	PHE	10	-6.606	11.561	3.947	1.00	0.00	C
ATOM	149	HB2	PHE	10	-6.847	12.572	3.617	1.00	0.00	H
ATOM	150	HB3	PHE	10	-7.555	11.025	3.984	1.00	0.00	H
ATOM	151	CG	PHE	10	-6.150	11.640	5.436	1.00	0.00	C
ATOM	152	CD1	PHE	10	-6.581	10.692	6.384	1.00	0.00	C
ATOM	153	HD1	PHE	10	-7.219	9.920	5.982	1.00	0.00	H
ATOM	154	CE1	PHE	10	-6.397	10.791	7.821	1.00	0.00	C
ATOM	155	HE1	PHE	10	-6.831	10.051	8.478	1.00	0.00	H
ATOM	156	CZ	PHE	10	-5.661	11.916	8.250	1.00	0.00	C
ATOM	157	HZ	PHE	10	-5.571	12.045	9.319	1.00	0.00	H
ATOM	158	CE2	PHE	10	-5.111	12.819	7.311	1.00	0.00	C
ATOM	159	HE2	PHE	10	-4.604	13.702	7.673	1.00	0.00	H
ATOM	160	CD2	PHE	10	-5.350	12.658	5.915	1.00	0.00	C
ATOM	161	HD2	PHE	10	-4.988	13.432	5.253	1.00	0.00	H
ATOM	162	C	PHE	10	-6.325	10.333	1.762	1.00	0.00	C
ATOM	163	O	PHE	10	-6.231	10.937	0.696	1.00	0.00	O
ATOM	164	N	GLY	11	-6.920	9.129	1.751	1.00	0.00	N
ATOM	165	H	GLY	11	-6.622	8.600	2.558	1.00	0.00	H
ATOM	166	CA	GLY	11	-7.522	8.405	0.602	1.00	0.00	C
ATOM	167	HA2	GLY	11	-6.953	8.828	-0.225	1.00	0.00	H
ATOM	168	HA3	GLY	11	-7.338	7.333	0.662	1.00	0.00	H
ATOM	169	C	GLY	11	-8.907	8.927	0.322	1.00	0.00	C
ATOM	170	O	GLY	11	-9.764	8.180	-0.085	1.00	0.00	O
ATOM	171	N	ASN	12	-9.229	10.165	0.668	1.00	0.00	N

ATOM	172	H	ASN	12	-8.565	10.638	1.264	1.00	0.00	H
ATOM	173	CA	ASN	12	-10.579	10.721	0.531	1.00	0.00	C
ATOM	174	HA	ASN	12	-10.941	10.319	-0.415	1.00	0.00	H
ATOM	175	CB	ASN	12	-11.437	10.362	1.806	1.00	0.00	C
ATOM	176	HB2	ASN	12	-11.596	9.291	1.921	1.00	0.00	H
ATOM	177	HB3	ASN	12	-10.957	10.675	2.733	1.00	0.00	H
ATOM	178	CG	ASN	12	-12.861	10.853	1.762	1.00	0.00	C
ATOM	179	OD1	ASN	12	-13.294	11.514	0.817	1.00	0.00	O
ATOM	180	ND2	ASN	12	-13.691	10.453	2.750	1.00	0.00	N
ATOM	181	HD21	ASN	12	-14.613	10.845	2.621	1.00	0.00	H
ATOM	182	HD22	ASN	12	-13.297	10.119	3.618	1.00	0.00	H
ATOM	183	C	ASN	12	-10.407	12.309	0.425	1.00	0.00	C
ATOM	184	O	ASN	12	-9.871	12.977	1.321	1.00	0.00	O
ATOM	185	N	ASP	13	-10.883	12.977	-0.663	1.00	0.00	N
ATOM	186	H	ASP	13	-11.367	12.416	-1.350	1.00	0.00	H
ATOM	187	CA	ASP	13	-10.728	14.391	-1.042	1.00	0.00	C
ATOM	188	HA	ASP	13	-9.677	14.674	-1.090	1.00	0.00	H
ATOM	189	CB	ASP	13	-11.331	14.624	-2.434	1.00	0.00	C
ATOM	190	HB2	ASP	13	-11.056	13.772	-3.056	1.00	0.00	H
ATOM	191	HB3	ASP	13	-12.422	14.608	-2.409	1.00	0.00	H
ATOM	192	CG	ASP	13	-10.857	15.908	-3.130	1.00	0.00	C
ATOM	193	OD1	ASP	13	-11.622	16.874	-3.053	1.00	0.00	O
ATOM	194	OD2	ASP	13	-9.696	16.064	-3.531	1.00	0.00	O
ATOM	195	C	ASP	13	-11.385	15.374	-0.023	1.00	0.00	C
ATOM	196	O	ASP	13	-10.981	16.556	0.082	1.00	0.00	O
ATOM	197	N	TYR	14	-12.317	14.922	0.785	1.00	0.00	N
ATOM	198	H	TYR	14	-12.644	13.998	0.540	1.00	0.00	H
ATOM	199	CA	TYR	14	-13.032	15.658	1.843	1.00	0.00	C
ATOM	200	HA	TYR	14	-13.117	16.712	1.579	1.00	0.00	H
ATOM	201	CB	TYR	14	-14.454	15.121	1.928	1.00	0.00	C
ATOM	202	HB2	TYR	14	-14.452	14.094	1.564	1.00	0.00	H
ATOM	203	HB3	TYR	14	-14.822	15.252	2.946	1.00	0.00	H
ATOM	204	CG	TYR	14	-15.408	15.808	0.943	1.00	0.00	C
ATOM	205	CD1	TYR	14	-15.274	15.658	-0.436	1.00	0.00	C
ATOM	206	HD1	TYR	14	-14.434	15.097	-0.820	1.00	0.00	H
ATOM	207	CE1	TYR	14	-16.218	16.271	-1.300	1.00	0.00	C
ATOM	208	HE1	TYR	14	-16.182	16.235	-2.379	1.00	0.00	H
ATOM	209	CZ	TYR	14	-17.168	17.230	-0.788	1.00	0.00	C
ATOM	210	OH	TYR	14	-18.043	17.940	-1.596	1.00	0.00	O
ATOM	211	HH	TYR	14	-18.670	18.492	-1.123	1.00	0.00	H
ATOM	212	CE2	TYR	14	-17.178	17.447	0.620	1.00	0.00	C
ATOM	213	HE2	TYR	14	-17.843	18.155	1.094	1.00	0.00	H
ATOM	214	CD2	TYR	14	-16.368	16.723	1.464	1.00	0.00	C
ATOM	215	HD2	TYR	14	-16.383	16.884	2.532	1.00	0.00	H
ATOM	216	C	TYR	14	-12.432	15.452	3.257	1.00	0.00	C
ATOM	217	O	TYR	14	-12.894	16.004	4.250	1.00	0.00	O
ATOM	218	N	GLU	15	-11.258	14.735	3.291	1.00	0.00	N
ATOM	219	H	GLU	15	-10.930	14.194	2.502	1.00	0.00	H
ATOM	220	CA	GLU	15	-10.336	14.775	4.407	1.00	0.00	C
ATOM	221	HA	GLU	15	-10.761	15.361	5.223	1.00	0.00	H
ATOM	222	CB	GLU	15	-9.977	13.370	4.926	1.00	0.00	C
ATOM	223	HB2	GLU	15	-9.628	12.749	4.101	1.00	0.00	H
ATOM	224	HB3	GLU	15	-9.084	13.371	5.552	1.00	0.00	H
ATOM	225	CG	GLU	15	-11.127	12.689	5.664	1.00	0.00	C
ATOM	226	HG2	GLU	15	-11.533	13.349	6.431	1.00	0.00	H
ATOM	227	HG3	GLU	15	-11.810	12.496	4.837	1.00	0.00	H
ATOM	228	CD	GLU	15	-10.563	11.422	6.297	1.00	0.00	C
ATOM	229	OE1	GLU	15	-10.301	10.399	5.608	1.00	0.00	O
ATOM	230	OE2	GLU	15	-10.371	11.503	7.535	1.00	0.00	O
ATOM	231	C	GLU	15	-9.021	15.430	3.958	1.00	0.00	C
ATOM	232	O	GLU	15	-8.436	16.173	4.709	1.00	0.00	O
ATOM	233	N	ASP	16	-8.668	15.226	2.669	1.00	0.00	N
ATOM	234	H	ASP	16	-9.232	14.659	2.053	1.00	0.00	H

ATOM	235	CA	ASP	16	-7.282	15.551	2.231	1.00	0.00	C
ATOM	236	HA	ASP	16	-6.607	15.185	3.005	1.00	0.00	H
ATOM	237	CB	ASP	16	-7.057	14.910	0.881	1.00	0.00	C
ATOM	238	HB2	ASP	16	-7.498	13.914	0.925	1.00	0.00	H
ATOM	239	HB3	ASP	16	-7.620	15.463	0.129	1.00	0.00	H
ATOM	240	CG	ASP	16	-5.619	14.818	0.527	1.00	0.00	C
ATOM	241	OD1	ASP	16	-5.205	15.239	-0.582	1.00	0.00	O
ATOM	242	OD2	ASP	16	-4.817	14.342	1.397	1.00	0.00	O
ATOM	243	C	ASP	16	-7.134	17.089	2.120	1.00	0.00	C
ATOM	244	O	ASP	16	-6.134	17.704	2.490	1.00	0.00	O
ATOM	245	N	ARG	17	-8.175	17.855	1.708	1.00	0.00	N
ATOM	246	H	ARG	17	-8.977	17.320	1.408	1.00	0.00	H
ATOM	247	CA	ARG	17	-8.188	19.296	1.480	1.00	0.00	C
ATOM	248	HA	ARG	17	-7.407	19.451	0.734	1.00	0.00	H
ATOM	249	CB	ARG	17	-9.603	19.651	0.938	1.00	0.00	C
ATOM	250	HB2	ARG	17	-9.676	20.691	0.621	1.00	0.00	H
ATOM	251	HB3	ARG	17	-9.643	19.109	-0.007	1.00	0.00	H
ATOM	252	CG	ARG	17	-10.781	19.291	1.844	1.00	0.00	C
ATOM	253	HG2	ARG	17	-10.765	18.217	2.032	1.00	0.00	H
ATOM	254	HG3	ARG	17	-10.615	19.889	2.740	1.00	0.00	H
ATOM	255	CD	ARG	17	-12.078	19.607	1.197	1.00	0.00	C
ATOM	256	HD2	ARG	17	-12.831	19.370	1.948	1.00	0.00	H
ATOM	257	HD3	ARG	17	-12.040	20.673	0.976	1.00	0.00	H
ATOM	258	NE	ARG	17	-12.375	18.973	-0.151	1.00	0.00	N
ATOM	259	HE	ARG	17	-11.587	18.428	-0.468	1.00	0.00	H
ATOM	260	CZ	ARG	17	-13.407	19.124	-0.946	1.00	0.00	C
ATOM	261	NH1	ARG	17	-14.416	19.907	-0.533	1.00	0.00	N
ATOM	262	HH11	ARG	17	-14.509	20.222	0.423	1.00	0.00	H
ATOM	263	HH12	ARG	17	-15.175	20.034	-1.186	1.00	0.00	H
ATOM	264	NH2	ARG	17	-13.466	18.587	-2.106	1.00	0.00	N
ATOM	265	HH21	ARG	17	-14.330	18.660	-2.624	1.00	0.00	H
ATOM	266	HH22	ARG	17	-12.644	18.057	-2.362	1.00	0.00	H
ATOM	267	C	ARG	17	-7.827	20.125	2.720	1.00	0.00	C
ATOM	268	O	ARG	17	-7.494	21.316	2.547	1.00	0.00	O
ATOM	269	N	TYR	18	-7.965	19.535	3.911	1.00	0.00	N
ATOM	270	H	TYR	18	-8.242	18.564	3.887	1.00	0.00	H
ATOM	271	CA	TYR	18	-7.495	20.217	5.160	1.00	0.00	C
ATOM	272	HA	TYR	18	-7.836	21.252	5.126	1.00	0.00	H
ATOM	273	CB	TYR	18	-8.076	19.481	6.325	1.00	0.00	C
ATOM	274	HB2	TYR	18	-7.803	18.438	6.164	1.00	0.00	H
ATOM	275	HB3	TYR	18	-7.542	19.870	7.192	1.00	0.00	H
ATOM	276	CG	TYR	18	-9.613	19.707	6.534	1.00	0.00	C
ATOM	277	CD1	TYR	18	-10.463	18.639	6.898	1.00	0.00	C
ATOM	278	HD1	TYR	18	-9.981	17.729	7.227	1.00	0.00	H
ATOM	279	CE1	TYR	18	-11.859	18.784	6.830	1.00	0.00	C
ATOM	280	HE1	TYR	18	-12.464	17.954	7.166	1.00	0.00	H
ATOM	281	CZ	TYR	18	-12.432	19.970	6.426	1.00	0.00	C
ATOM	282	OH	TYR	18	-13.779	20.145	6.174	1.00	0.00	O
ATOM	283	HH	TYR	18	-14.063	21.051	6.030	1.00	0.00	H
ATOM	284	CE2	TYR	18	-11.579	21.086	6.199	1.00	0.00	C
ATOM	285	HE2	TYR	18	-12.058	22.033	5.995	1.00	0.00	H
ATOM	286	CD2	TYR	18	-10.186	20.995	6.283	1.00	0.00	C
ATOM	287	HD2	TYR	18	-9.539	21.841	6.107	1.00	0.00	H
ATOM	288	C	TYR	18	-5.988	20.287	5.240	1.00	0.00	C
ATOM	289	O	TYR	18	-5.497	21.318	5.669	1.00	0.00	O
ATOM	290	N	TYR	19	-5.229	19.271	4.764	1.00	0.00	N
ATOM	291	H	TYR	19	-5.843	18.627	4.286	1.00	0.00	H
ATOM	292	CA	TYR	19	-3.784	18.978	4.805	1.00	0.00	C
ATOM	293	HA	TYR	19	-3.393	19.581	5.624	1.00	0.00	H
ATOM	294	CB	TYR	19	-3.468	17.596	5.291	1.00	0.00	C
ATOM	295	HB2	TYR	19	-3.571	17.016	4.375	1.00	0.00	H
ATOM	296	HB3	TYR	19	-2.434	17.738	5.611	1.00	0.00	H
ATOM	297	CG	TYR	19	-4.338	17.189	6.473	1.00	0.00	C

ATOM	298	CD1	TYR	19	-5.569	16.499	6.223	1.00	0.00	C
ATOM	299	HD1	TYR	19	-5.856	16.192	5.227	1.00	0.00	H
ATOM	300	CE1	TYR	19	-6.377	16.104	7.311	1.00	0.00	C
ATOM	301	HE1	TYR	19	-7.245	15.487	7.132	1.00	0.00	H
ATOM	302	CZ	TYR	19	-5.998	16.367	8.642	1.00	0.00	C
ATOM	303	OH	TYR	19	-6.839	15.857	9.609	1.00	0.00	O
ATOM	304	HH	TYR	19	-6.489	16.034	10.485	1.00	0.00	H
ATOM	305	CE2	TYR	19	-4.813	17.182	8.924	1.00	0.00	C
ATOM	306	HE2	TYR	19	-4.613	17.533	9.925	1.00	0.00	H
ATOM	307	CD2	TYR	19	-4.010	17.585	7.818	1.00	0.00	C
ATOM	308	HD2	TYR	19	-3.169	18.256	7.905	1.00	0.00	H
ATOM	309	C	TYR	19	-3.038	19.312	3.600	1.00	0.00	C
ATOM	310	O	TYR	19	-1.870	19.576	3.580	1.00	0.00	O
ATOM	311	N	ARG	20	-3.793	19.346	2.490	1.00	0.00	N
ATOM	312	H	ARG	20	-4.674	18.855	2.526	1.00	0.00	H
ATOM	313	CA	ARG	20	-3.292	19.705	1.161	1.00	0.00	C
ATOM	314	HA	ARG	20	-2.405	19.116	0.930	1.00	0.00	H
ATOM	315	CB	ARG	20	-4.518	19.479	0.174	1.00	0.00	C
ATOM	316	HB2	ARG	20	-4.987	18.512	0.354	1.00	0.00	H
ATOM	317	HB3	ARG	20	-5.321	20.179	0.411	1.00	0.00	H
ATOM	318	CG	ARG	20	-4.166	19.608	-1.302	1.00	0.00	C
ATOM	319	HG2	ARG	20	-3.757	20.610	-1.425	1.00	0.00	H
ATOM	320	HG3	ARG	20	-3.415	18.850	-1.526	1.00	0.00	H
ATOM	321	CD	ARG	20	-5.390	19.382	-2.131	1.00	0.00	C
ATOM	322	HD2	ARG	20	-6.175	20.056	-1.789	1.00	0.00	H
ATOM	323	HD3	ARG	20	-5.222	19.553	-3.195	1.00	0.00	H
ATOM	324	NE	ARG	20	-5.855	17.999	-1.998	1.00	0.00	N
ATOM	325	HE	ARG	20	-5.161	17.342	-1.672	1.00	0.00	H
ATOM	326	CZ	ARG	20	-7.028	17.521	-2.409	1.00	0.00	C
ATOM	327	NH1	ARG	20	-7.888	18.224	-3.020	1.00	0.00	N
ATOM	328	HH11	ARG	20	-8.713	17.729	-3.328	1.00	0.00	H
ATOM	329	HH12	ARG	20	-7.649	19.112	-3.437	1.00	0.00	H
ATOM	330	NH2	ARG	20	-7.233	16.240	-2.313	1.00	0.00	N
ATOM	331	HH21	ARG	20	-8.066	15.923	-2.787	1.00	0.00	H
ATOM	332	HH22	ARG	20	-6.527	15.608	-1.963	1.00	0.00	H
ATOM	333	C	ARG	20	-2.749	21.113	1.000	1.00	0.00	C
ATOM	334	O	ARG	20	-2.023	21.393	0.052	1.00	0.00	O
ATOM	335	N	GLU	21	-3.277	22.028	1.805	1.00	0.00	N
ATOM	336	H	GLU	21	-4.049	21.763	2.400	1.00	0.00	H
ATOM	337	CA	GLU	21	-2.925	23.423	1.803	1.00	0.00	C
ATOM	338	HA	GLU	21	-2.938	23.917	0.831	1.00	0.00	H
ATOM	339	CB	GLU	21	-4.012	24.293	2.506	1.00	0.00	C
ATOM	340	HB2	GLU	21	-3.689	25.333	2.507	1.00	0.00	H
ATOM	341	HB3	GLU	21	-4.990	24.090	2.070	1.00	0.00	H
ATOM	342	CG	GLU	21	-4.094	23.961	4.024	1.00	0.00	C
ATOM	343	HG2	GLU	21	-4.286	22.917	4.266	1.00	0.00	H
ATOM	344	HG3	GLU	21	-3.113	24.038	4.491	1.00	0.00	H
ATOM	345	CD	GLU	21	-5.144	24.849	4.735	1.00	0.00	C
ATOM	346	OE1	GLU	21	-6.358	24.455	4.762	1.00	0.00	O
ATOM	347	OE2	GLU	21	-4.810	25.885	5.398	1.00	0.00	O
ATOM	348	C	GLU	21	-1.455	23.624	2.428	1.00	0.00	C
ATOM	349	O	GLU	21	-0.918	24.743	2.297	1.00	0.00	O
ATOM	350	N	ASN	22	-0.803	22.548	2.968	1.00	0.00	N
ATOM	351	H	ASN	22	-1.142	21.602	2.861	1.00	0.00	H
ATOM	352	CA	ASN	22	0.648	22.713	3.310	1.00	0.00	C
ATOM	353	HA	ASN	22	0.914	23.693	3.706	1.00	0.00	H
ATOM	354	CB	ASN	22	1.095	21.709	4.316	1.00	0.00	C
ATOM	355	HB2	ASN	22	0.375	21.551	5.119	1.00	0.00	H
ATOM	356	HB3	ASN	22	1.107	20.717	3.864	1.00	0.00	H
ATOM	357	CG	ASN	22	2.424	21.945	4.985	1.00	0.00	C
ATOM	358	OD1	ASN	22	3.236	22.794	4.599	1.00	0.00	O
ATOM	359	ND2	ASN	22	2.813	21.285	6.106	1.00	0.00	N
ATOM	360	HD21	ASN	22	2.300	20.557	6.583	1.00	0.00	H

ATOM	361	HD22	ASN	22	3.758	21.402	6.443	1.00	0.00	H
ATOM	362	C	ASN	22	1.462	22.595	2.006	1.00	0.00	C
ATOM	363	O	ASN	22	1.707	21.489	1.528	1.00	0.00	O
ATOM	364	N	MET	23	1.882	23.736	1.480	1.00	0.00	N
ATOM	365	H	MET	23	1.782	24.598	1.996	1.00	0.00	H
ATOM	366	CA	MET	23	2.471	23.843	0.188	1.00	0.00	C
ATOM	367	HA	MET	23	1.703	23.476	-0.494	1.00	0.00	H
ATOM	368	CB	MET	23	2.763	25.345	-0.131	1.00	0.00	C
ATOM	369	HB2	MET	23	2.000	26.019	0.257	1.00	0.00	H
ATOM	370	HB3	MET	23	3.737	25.595	0.288	1.00	0.00	H
ATOM	371	CG	MET	23	2.897	25.504	-1.700	1.00	0.00	C
ATOM	372	HG2	MET	23	3.908	25.161	-1.912	1.00	0.00	H
ATOM	373	HG3	MET	23	2.238	24.796	-2.203	1.00	0.00	H
ATOM	374	SD	MET	23	2.748	27.230	-2.276	1.00	0.00	S
ATOM	375	CE	MET	23	4.385	27.783	-1.682	1.00	0.00	C
ATOM	376	HE1	MET	23	5.181	27.174	-2.109	1.00	0.00	H
ATOM	377	HE2	MET	23	4.501	28.827	-1.973	1.00	0.00	H
ATOM	378	HE3	MET	23	4.343	27.795	-0.593	1.00	0.00	H
ATOM	379	C	MET	23	3.653	22.949	-0.075	1.00	0.00	C
ATOM	380	O	MET	23	4.086	22.726	-1.233	1.00	0.00	O
ATOM	381	N	TYR	24	4.331	22.419	0.951	1.00	0.00	N
ATOM	382	H	TYR	24	3.843	22.502	1.831	1.00	0.00	H
ATOM	383	CA	TYR	24	5.406	21.463	0.953	1.00	0.00	C
ATOM	384	HA	TYR	24	6.172	22.073	0.475	1.00	0.00	H
ATOM	385	CB	TYR	24	5.800	21.204	2.431	1.00	0.00	C
ATOM	386	HB2	TYR	24	6.100	22.086	2.996	1.00	0.00	H
ATOM	387	HB3	TYR	24	4.893	20.859	2.930	1.00	0.00	H
ATOM	388	CG	TYR	24	6.956	20.246	2.473	1.00	0.00	C
ATOM	389	CD1	TYR	24	8.277	20.696	2.244	1.00	0.00	C
ATOM	390	HD1	TYR	24	8.390	21.766	2.151	1.00	0.00	H
ATOM	391	CE1	TYR	24	9.390	19.873	2.252	1.00	0.00	C
ATOM	392	HE1	TYR	24	10.374	20.307	2.154	1.00	0.00	H
ATOM	393	CZ	TYR	24	9.171	18.446	2.489	1.00	0.00	C
ATOM	394	OH	TYR	24	10.272	17.620	2.395	1.00	0.00	O
ATOM	395	HH	TYR	24	11.094	18.067	2.177	1.00	0.00	H
ATOM	396	CE2	TYR	24	7.835	17.972	2.653	1.00	0.00	C
ATOM	397	HE2	TYR	24	7.576	16.930	2.772	1.00	0.00	H
ATOM	398	CD2	TYR	24	6.797	18.878	2.677	1.00	0.00	C
ATOM	399	HD2	TYR	24	5.770	18.548	2.735	1.00	0.00	H
ATOM	400	C	TYR	24	5.045	20.232	0.112	1.00	0.00	C
ATOM	401	O	TYR	24	5.873	19.680	-0.646	1.00	0.00	O
ATOM	402	N	ARG	25	3.764	19.853	0.131	1.00	0.00	N
ATOM	403	H	ARG	25	3.036	20.440	0.511	1.00	0.00	H
ATOM	404	CA	ARG	25	3.242	18.660	-0.615	1.00	0.00	C
ATOM	405	HA	ARG	25	3.761	17.728	-0.392	1.00	0.00	H
ATOM	406	CB	ARG	25	1.774	18.295	-0.293	1.00	0.00	C
ATOM	407	HB2	ARG	25	1.166	19.194	-0.402	1.00	0.00	H
ATOM	408	HB3	ARG	25	1.434	17.477	-0.928	1.00	0.00	H
ATOM	409	CG	ARG	25	1.500	17.810	1.119	1.00	0.00	C
ATOM	410	HG2	ARG	25	1.980	16.832	1.177	1.00	0.00	H
ATOM	411	HG3	ARG	25	1.949	18.541	1.791	1.00	0.00	H
ATOM	412	CD	ARG	25	0.061	17.700	1.413	1.00	0.00	C
ATOM	413	HD2	ARG	25	-0.043	17.196	2.374	1.00	0.00	H
ATOM	414	HD3	ARG	25	-0.304	18.697	1.662	1.00	0.00	H
ATOM	415	NE	ARG	25	-0.822	17.077	0.339	1.00	0.00	N
ATOM	416	HE	ARG	25	-0.436	17.305	-0.566	1.00	0.00	H
ATOM	417	CZ	ARG	25	-1.993	16.579	0.405	1.00	0.00	C
ATOM	418	NH1	ARG	25	-2.671	16.318	1.482	1.00	0.00	N
ATOM	419	HH11	ARG	25	-3.534	15.793	1.499	1.00	0.00	H
ATOM	420	HH12	ARG	25	-2.212	16.436	2.374	1.00	0.00	H
ATOM	421	NH2	ARG	25	-2.581	16.257	-0.626	1.00	0.00	N
ATOM	422	HH21	ARG	25	-2.127	16.334	-1.525	1.00	0.00	H
ATOM	423	HH22	ARG	25	-3.403	15.673	-0.584	1.00	0.00	H

```
ATOM    424  C   ARG    25       3.467  18.819  -2.182  1.00  0.00           C
ATOM    425  O   ARG    25       3.665  17.828  -2.885  1.00  0.00           O
ATOM    426  N   TYR    26       3.591  20.093  -2.568  1.00  0.00           N
ATOM    427  H   TYR    26       3.730  20.783  -1.844  1.00  0.00           H
ATOM    428  CA  TYR    26       3.765  20.506  -3.945  1.00  0.00           C
ATOM    429  HA  TYR    26       3.909  19.584  -4.508  1.00  0.00           H
ATOM    430  CB  TYR    26       2.438  21.130  -4.368  1.00  0.00           C
ATOM    431  HB2 TYR    26       2.248  22.022  -3.770  1.00  0.00           H
ATOM    432  HB3 TYR    26       2.495  21.561  -5.368  1.00  0.00           H
ATOM    433  CG  TYR    26       1.293  20.173  -4.263  1.00  0.00           C
ATOM    434  CD1 TYR    26       1.440  19.029  -5.128  1.00  0.00           C
ATOM    435  HD1 TYR    26       2.193  18.968  -5.900  1.00  0.00           H
ATOM    436  CE1 TYR    26       0.604  17.944  -4.799  1.00  0.00           C
ATOM    437  HE1 TYR    26       0.559  17.049  -5.400  1.00  0.00           H
ATOM    438  CZ  TYR    26      -0.285  17.987  -3.735  1.00  0.00           C
ATOM    439  OH  TYR    26      -1.078  16.857  -3.502  1.00  0.00           O
ATOM    440  HH  TYR    26      -0.734  16.194  -4.104  1.00  0.00           H
ATOM    441  CE2 TYR    26      -0.398  19.080  -2.881  1.00  0.00           C
ATOM    442  HE2 TYR    26      -1.032  19.157  -2.012  1.00  0.00           H
ATOM    443  CD2 TYR    26       0.437  20.191  -3.143  1.00  0.00           C
ATOM    444  HD2 TYR    26       0.511  21.037  -2.474  1.00  0.00           H
ATOM    445  C   TYR    26       4.975  21.386  -4.297  1.00  0.00           C
ATOM    446  O   TYR    26       4.863  22.154  -5.286  1.00  0.00           O
ATOM    447  N   NME    27       6.030  21.375  -3.503  1.00  0.00           N
ATOM    448  H   NME    27       6.114  20.559  -2.914  1.00  0.00           H
ATOM    449  CH3 NME    27       7.192  22.269  -3.646  1.00  0.00           C
ATOM    450 HH31 NME    27       8.090  21.802  -3.243  1.00  0.00           H
ATOM    451 HH32 NME    27       7.446  22.520  -4.676  1.00  0.00           H
ATOM    452 HH33 NME    27       7.031  23.095  -2.954  1.00  0.00           H
TER     453      NME    27
END
```

Cluster1, Figure 1A (blue):

```
ATOM      1 HH31 ACE     1       4.525   7.115  -1.730  1.00  0.00           H
ATOM      2  CH3 ACE     1       3.963   7.152  -2.664  1.00  0.00           C
ATOM      3 HH32 ACE     1       3.059   7.735  -2.483  1.00  0.00           H
ATOM      4 HH33 ACE     1       3.568   6.158  -2.878  1.00  0.00           H
ATOM      5  C   ACE     1       4.878   7.624  -3.851  1.00  0.00           C
ATOM      6  O   ACE     1       6.097   7.605  -3.834  1.00  0.00           O
ATOM      7  N   VAL     2       4.156   8.002  -4.891  1.00  0.00           N
ATOM      8  H   VAL     2       3.176   8.125  -4.685  1.00  0.00           H
ATOM      9  CA  VAL     2       4.594   8.411  -6.245  1.00  0.00           C
ATOM     10  HA  VAL     2       5.275   7.658  -6.640  1.00  0.00           H
ATOM     11  CB  VAL     2       3.435   8.427  -7.299  1.00  0.00           C
ATOM     12  HB  VAL     2       3.753   8.596  -8.328  1.00  0.00           H
ATOM     13  CG1 VAL     2       2.694   7.116  -7.447  1.00  0.00           C
ATOM     14 HG11 VAL     2       3.354   6.254  -7.535  1.00  0.00           H
ATOM     15 HG12 VAL     2       2.030   6.938  -6.601  1.00  0.00           H
ATOM     16 HG13 VAL     2       2.154   7.222  -8.387  1.00  0.00           H
ATOM     17  CG2 VAL     2       2.414   9.573  -7.165  1.00  0.00           C
ATOM     18 HG21 VAL     2       1.919   9.450  -6.202  1.00  0.00           H
ATOM     19 HG22 VAL     2       2.825  10.555  -7.401  1.00  0.00           H
ATOM     20 HG23 VAL     2       1.682   9.477  -7.968  1.00  0.00           H
ATOM     21  C   VAL     2       5.400   9.760  -6.191  1.00  0.00           C
ATOM     22  O   VAL     2       6.451   9.941  -6.803  1.00  0.00           O
ATOM     23  N   MET     3       4.888  10.726  -5.396  1.00  0.00           N
ATOM     24  H   MET     3       4.044  10.506  -4.887  1.00  0.00           H
ATOM     25  CA  MET     3       5.378  12.106  -5.281  1.00  0.00           C
ATOM     26  HA  MET     3       6.404  12.184  -5.641  1.00  0.00           H
ATOM     27  CB  MET     3       4.490  13.150  -5.831  1.00  0.00           C
```

ATOM	28	HB2	MET	3	4.917	14.130	-5.618	1.00	0.00	H
ATOM	29	HB3	MET	3	4.357	13.026	-6.907	1.00	0.00	H
ATOM	30	CG	MET	3	3.113	13.191	-5.200	1.00	0.00	C
ATOM	31	HG2	MET	3	2.633	12.292	-5.587	1.00	0.00	H
ATOM	32	HG3	MET	3	3.176	13.165	-4.113	1.00	0.00	H
ATOM	33	SD	MET	3	1.975	14.544	-5.655	1.00	0.00	S
ATOM	34	CE	MET	3	0.485	13.774	-4.958	1.00	0.00	C
ATOM	35	HE1	MET	3	-0.294	14.195	-5.594	1.00	0.00	H
ATOM	36	HE2	MET	3	0.563	12.702	-5.138	1.00	0.00	H
ATOM	37	HE3	MET	3	0.352	13.977	-3.895	1.00	0.00	H
ATOM	38	C	MET	3	5.610	12.283	-3.755	1.00	0.00	C
ATOM	39	O	MET	3	4.868	11.663	-2.928	1.00	0.00	O
ATOM	40	N	SER	4	6.499	13.211	-3.315	1.00	0.00	N
ATOM	41	H	SER	4	7.017	13.791	-3.959	1.00	0.00	H
ATOM	42	CA	SER	4	6.918	13.338	-1.893	1.00	0.00	C
ATOM	43	HA	SER	4	6.977	12.363	-1.409	1.00	0.00	H
ATOM	44	CB	SER	4	8.331	13.944	-1.751	1.00	0.00	C
ATOM	45	HB2	SER	4	8.422	14.882	-2.299	1.00	0.00	H
ATOM	46	HB3	SER	4	8.644	13.999	-0.709	1.00	0.00	H
ATOM	47	OG	SER	4	9.180	13.023	-2.421	1.00	0.00	O
ATOM	48	HG	SER	4	10.054	13.420	-2.424	1.00	0.00	H
ATOM	49	C	SER	4	5.886	14.040	-0.991	1.00	0.00	C
ATOM	50	O	SER	4	6.354	14.817	-0.130	1.00	0.00	O
ATOM	51	N	ARG	5	4.586	13.812	-1.167	1.00	0.00	N
ATOM	52	H	ARG	5	4.348	13.247	-1.970	1.00	0.00	H
ATOM	53	CA	ARG	5	3.485	14.133	-0.238	1.00	0.00	C
ATOM	54	HA	ARG	5	3.497	15.207	-0.051	1.00	0.00	H
ATOM	55	CB	ARG	5	2.184	13.822	-1.054	1.00	0.00	C
ATOM	56	HB2	ARG	5	1.343	14.128	-0.431	1.00	0.00	H
ATOM	57	HB3	ARG	5	2.120	14.464	-1.933	1.00	0.00	H
ATOM	58	CG	ARG	5	1.924	12.356	-1.489	1.00	0.00	C
ATOM	59	HG2	ARG	5	2.314	12.110	-2.476	1.00	0.00	H
ATOM	60	HG3	ARG	5	2.443	11.639	-0.852	1.00	0.00	H
ATOM	61	CD	ARG	5	0.460	11.885	-1.404	1.00	0.00	C
ATOM	62	HD2	ARG	5	-0.139	12.627	-1.933	1.00	0.00	H
ATOM	63	HD3	ARG	5	0.415	10.916	-1.902	1.00	0.00	H
ATOM	64	NE	ARG	5	-0.087	11.720	-0.048	1.00	0.00	N
ATOM	65	HE	ARG	5	0.526	11.395	0.685	1.00	0.00	H
ATOM	66	CZ	ARG	5	-1.251	12.192	0.382	1.00	0.00	C
ATOM	67	NH1	ARG	5	-2.152	12.460	-0.468	1.00	0.00	N
ATOM	68	HH11	ARG	5	-1.910	12.188	-1.410	1.00	0.00	H
ATOM	69	HH12	ARG	5	-3.074	12.794	-0.227	1.00	0.00	H
ATOM	70	NH2	ARG	5	-1.499	12.303	1.623	1.00	0.00	N
ATOM	71	HH21	ARG	5	-0.941	11.831	2.319	1.00	0.00	H
ATOM	72	HH22	ARG	5	-2.386	12.668	1.941	1.00	0.00	H
ATOM	73	C	ARG	5	3.663	13.407	1.094	1.00	0.00	C
ATOM	74	O	ARG	5	4.412	12.470	1.240	1.00	0.00	O
ATOM	75	N	PRO	6	2.787	13.667	2.086	1.00	0.00	N
ATOM	76	CD	PRO	6	1.937	14.812	2.173	1.00	0.00	C
ATOM	77	HD2	PRO	6	1.052	14.681	1.550	1.00	0.00	H
ATOM	78	HD3	PRO	6	2.465	15.691	1.803	1.00	0.00	H
ATOM	79	CG	PRO	6	1.592	14.956	3.657	1.00	0.00	C
ATOM	80	HG2	PRO	6	0.615	15.397	3.858	1.00	0.00	H
ATOM	81	HG3	PRO	6	2.430	15.467	4.129	1.00	0.00	H
ATOM	82	CB	PRO	6	1.653	13.518	4.251	1.00	0.00	C
ATOM	83	HB2	PRO	6	0.697	12.995	4.202	1.00	0.00	H
ATOM	84	HB3	PRO	6	2.017	13.584	5.276	1.00	0.00	H
ATOM	85	CA	PRO	6	2.680	12.794	3.267	1.00	0.00	C
ATOM	86	HA	PRO	6	3.645	12.751	3.771	1.00	0.00	H
ATOM	87	C	PRO	6	2.349	11.347	3.013	1.00	0.00	C
ATOM	88	O	PRO	6	1.505	11.141	2.156	1.00	0.00	O
ATOM	89	N	LEU	7	2.854	10.393	3.725	1.00	0.00	N
ATOM	90	H	LEU	7	3.513	10.679	4.436	1.00	0.00	H

ATOM	91	CA	LEU	7	2.472	8.944	3.587	1.00	0.00	C
ATOM	92	HA	LEU	7	2.364	8.676	2.536	1.00	0.00	H
ATOM	93	CB	LEU	7	3.441	7.927	4.264	1.00	0.00	C
ATOM	94	HB2	LEU	7	4.456	8.093	3.904	1.00	0.00	H
ATOM	95	HB3	LEU	7	3.508	8.106	5.337	1.00	0.00	H
ATOM	96	CG	LEU	7	3.247	6.454	4.064	1.00	0.00	C
ATOM	97	HG	LEU	7	2.276	6.291	4.531	1.00	0.00	H
ATOM	98	CD1	LEU	7	3.242	5.928	2.659	1.00	0.00	C
ATOM	99	HD11	LEU	7	2.582	6.548	2.053	1.00	0.00	H
ATOM	100	HD12	LEU	7	4.279	6.073	2.357	1.00	0.00	H
ATOM	101	HD13	LEU	7	3.075	4.859	2.795	1.00	0.00	H
ATOM	102	CD2	LEU	7	4.339	5.724	4.893	1.00	0.00	C
ATOM	103	HD21	LEU	7	5.310	6.035	4.508	1.00	0.00	H
ATOM	104	HD22	LEU	7	4.450	6.148	5.892	1.00	0.00	H
ATOM	105	HD23	LEU	7	4.184	4.646	4.923	1.00	0.00	H
ATOM	106	C	LEU	7	1.059	8.701	4.145	1.00	0.00	C
ATOM	107	O	LEU	7	0.379	7.774	3.647	1.00	0.00	O
ATOM	108	N	ILE	8	0.686	9.520	5.127	1.00	0.00	N
ATOM	109	H	ILE	8	1.201	10.355	5.366	1.00	0.00	H
ATOM	110	CA	ILE	8	-0.628	9.332	5.726	1.00	0.00	C
ATOM	111	HA	ILE	8	-0.953	8.292	5.712	1.00	0.00	H
ATOM	112	CB	ILE	8	-0.655	9.894	7.168	1.00	0.00	C
ATOM	113	HB	ILE	8	-1.676	9.858	7.548	1.00	0.00	H
ATOM	114	CG2	ILE	8	0.250	8.969	7.947	1.00	0.00	C
ATOM	115	HG21	ILE	8	1.303	9.205	7.792	1.00	0.00	H
ATOM	116	HG22	ILE	8	0.062	8.997	9.021	1.00	0.00	H
ATOM	117	HG23	ILE	8	0.138	7.940	7.608	1.00	0.00	H
ATOM	118	CG1	ILE	8	-0.333	11.366	7.432	1.00	0.00	C
ATOM	119	HG12	ILE	8	0.746	11.422	7.577	1.00	0.00	H
ATOM	120	HG13	ILE	8	-0.664	12.099	6.696	1.00	0.00	H
ATOM	121	CD1	ILE	8	-0.943	11.863	8.796	1.00	0.00	C
ATOM	122	HD11	ILE	8	-0.837	12.946	8.866	1.00	0.00	H
ATOM	123	HD12	ILE	8	-2.006	11.654	8.902	1.00	0.00	H
ATOM	124	HD13	ILE	8	-0.322	11.339	9.523	1.00	0.00	H
ATOM	125	C	ILE	8	-1.748	9.925	4.831	1.00	0.00	C
ATOM	126	O	ILE	8	-1.508	10.700	3.867	1.00	0.00	O
ATOM	127	N	HID	9	-2.976	9.556	5.150	1.00	0.00	N
ATOM	128	H	HID	9	-3.047	8.968	5.968	1.00	0.00	H
ATOM	129	CA	HID	9	-4.222	9.981	4.520	1.00	0.00	C
ATOM	130	HA	HID	9	-4.135	10.914	3.964	1.00	0.00	H
ATOM	131	CB	HID	9	-4.616	8.783	3.646	1.00	0.00	C
ATOM	132	HB2	HID	9	-3.728	8.430	3.123	1.00	0.00	H
ATOM	133	HB3	HID	9	-4.861	7.950	4.305	1.00	0.00	H
ATOM	134	CG	HID	9	-5.675	8.985	2.638	1.00	0.00	C
ATOM	135	ND1	HID	9	-5.849	10.016	1.744	1.00	0.00	N
ATOM	136	HD1	HID	9	-5.089	10.624	1.476	1.00	0.00	H
ATOM	137	CE1	HID	9	-6.939	9.775	0.977	1.00	0.00	C
ATOM	138	HE1	HID	9	-7.241	10.405	0.153	1.00	0.00	H
ATOM	139	NE2	HID	9	-7.592	8.663	1.407	1.00	0.00	N
ATOM	140	CD2	HID	9	-6.728	8.144	2.393	1.00	0.00	C
ATOM	141	HD2	HID	9	-6.899	7.231	2.944	1.00	0.00	H
ATOM	142	C	HID	9	-5.399	10.075	5.492	1.00	0.00	C
ATOM	143	O	HID	9	-5.314	9.605	6.633	1.00	0.00	O
ATOM	144	N	PHE	10	-6.503	10.809	5.126	1.00	0.00	N
ATOM	145	H	PHE	10	-6.464	11.219	4.204	1.00	0.00	H
ATOM	146	CA	PHE	10	-7.616	11.196	6.088	1.00	0.00	C
ATOM	147	HA	PHE	10	-7.431	10.909	7.122	1.00	0.00	H
ATOM	148	CB	PHE	10	-7.544	12.773	6.179	1.00	0.00	C
ATOM	149	HB2	PHE	10	-7.502	13.211	5.182	1.00	0.00	H
ATOM	150	HB3	PHE	10	-8.522	12.999	6.603	1.00	0.00	H
ATOM	151	CG	PHE	10	-6.411	13.279	7.050	1.00	0.00	C
ATOM	152	CD1	PHE	10	-6.555	13.442	8.449	1.00	0.00	C
ATOM	153	HD1	PHE	10	-7.504	13.239	8.922	1.00	0.00	H

ATOM	154	CE1	PHE	10	-5.478	13.990	9.134	1.00	0.00	C
ATOM	155	HE1	PHE	10	-5.528	14.299	10.168	1.00	0.00	H
ATOM	156	CZ	PHE	10	-4.202	14.177	8.568	1.00	0.00	C
ATOM	157	HZ	PHE	10	-3.411	14.684	9.102	1.00	0.00	H
ATOM	158	CE2	PHE	10	-4.072	13.903	7.218	1.00	0.00	C
ATOM	159	HE2	PHE	10	-3.074	14.007	6.819	1.00	0.00	H
ATOM	160	CD2	PHE	10	-5.155	13.433	6.476	1.00	0.00	C
ATOM	161	HD2	PHE	10	-5.029	13.291	5.413	1.00	0.00	H
ATOM	162	C	PHE	10	-8.977	10.505	5.684	1.00	0.00	C
ATOM	163	O	PHE	10	-10.005	10.920	6.166	1.00	0.00	O
ATOM	164	N	GLY	11	-8.895	9.540	4.780	1.00	0.00	N
ATOM	165	H	GLY	11	-7.974	9.223	4.512	1.00	0.00	H
ATOM	166	CA	GLY	11	-9.988	8.867	4.238	1.00	0.00	C
ATOM	167	HA2	GLY	11	-9.530	8.129	3.581	1.00	0.00	H
ATOM	168	HA3	GLY	11	-10.530	8.484	5.102	1.00	0.00	H
ATOM	169	C	GLY	11	-10.947	9.671	3.398	1.00	0.00	C
ATOM	170	O	GLY	11	-12.043	9.271	3.201	1.00	0.00	O
ATOM	171	N	ASN	12	-10.558	10.847	2.951	1.00	0.00	N
ATOM	172	H	ASN	12	-9.597	11.105	3.124	1.00	0.00	H
ATOM	173	CA	ASN	12	-11.429	11.866	2.248	1.00	0.00	C
ATOM	174	HA	ASN	12	-12.223	11.342	1.716	1.00	0.00	H
ATOM	175	CB	ASN	12	-12.150	12.826	3.267	1.00	0.00	C
ATOM	176	HB2	ASN	12	-11.336	13.229	3.871	1.00	0.00	H
ATOM	177	HB3	ASN	12	-12.505	13.702	2.725	1.00	0.00	H
ATOM	178	CG	ASN	12	-13.245	12.087	4.064	1.00	0.00	C
ATOM	179	OD1	ASN	12	-14.159	11.481	3.467	1.00	0.00	O
ATOM	180	ND2	ASN	12	-13.283	12.006	5.369	1.00	0.00	N
ATOM	181	HD21	ASN	12	-14.016	11.494	5.838	1.00	0.00	H
ATOM	182	HD22	ASN	12	-12.472	12.344	5.867	1.00	0.00	H
ATOM	183	C	ASN	12	-10.582	12.762	1.302	1.00	0.00	C
ATOM	184	O	ASN	12	-9.678	13.439	1.781	1.00	0.00	O
ATOM	185	N	ASP	13	-10.956	12.910	0.022	1.00	0.00	N
ATOM	186	H	ASP	13	-11.559	12.207	-0.382	1.00	0.00	H
ATOM	187	CA	ASP	13	-10.256	13.834	-0.800	1.00	0.00	C
ATOM	188	HA	ASP	13	-9.222	13.512	-0.675	1.00	0.00	H
ATOM	189	CB	ASP	13	-10.616	13.596	-2.220	1.00	0.00	C
ATOM	190	HB2	ASP	13	-10.302	12.584	-2.478	1.00	0.00	H
ATOM	191	HB3	ASP	13	-11.669	13.736	-2.465	1.00	0.00	H
ATOM	192	CG	ASP	13	-10.018	14.559	-3.218	1.00	0.00	C
ATOM	193	OD1	ASP	13	-10.764	15.288	-3.907	1.00	0.00	O
ATOM	194	OD2	ASP	13	-8.783	14.490	-3.439	1.00	0.00	O
ATOM	195	C	ASP	13	-10.315	15.268	-0.387	1.00	0.00	C
ATOM	196	O	ASP	13	-9.327	15.986	-0.533	1.00	0.00	O
ATOM	197	N	TYR	14	-11.385	15.780	0.233	1.00	0.00	N
ATOM	198	H	TYR	14	-12.234	15.239	0.315	1.00	0.00	H
ATOM	199	CA	TYR	14	-11.396	17.212	0.546	1.00	0.00	C
ATOM	200	HA	TYR	14	-10.887	17.699	-0.285	1.00	0.00	H
ATOM	201	CB	TYR	14	-12.850	17.660	0.727	1.00	0.00	C
ATOM	202	HB2	TYR	14	-12.907	18.722	0.962	1.00	0.00	H
ATOM	203	HB3	TYR	14	-13.377	17.481	-0.209	1.00	0.00	H
ATOM	204	CG	TYR	14	-13.446	16.886	1.892	1.00	0.00	C
ATOM	205	CD1	TYR	14	-14.314	15.778	1.627	1.00	0.00	C
ATOM	206	HD1	TYR	14	-14.554	15.543	0.601	1.00	0.00	H
ATOM	207	CE1	TYR	14	-14.964	15.217	2.756	1.00	0.00	C
ATOM	208	HE1	TYR	14	-15.682	14.465	2.463	1.00	0.00	H
ATOM	209	CZ	TYR	14	-14.653	15.697	4.060	1.00	0.00	C
ATOM	210	OH	TYR	14	-15.217	15.073	5.131	1.00	0.00	O
ATOM	211	HH	TYR	14	-14.942	15.560	5.911	1.00	0.00	H
ATOM	212	CE2	TYR	14	-13.891	16.817	4.276	1.00	0.00	C
ATOM	213	HE2	TYR	14	-13.686	17.232	5.252	1.00	0.00	H
ATOM	214	CD2	TYR	14	-13.340	17.465	3.158	1.00	0.00	C
ATOM	215	HD2	TYR	14	-12.840	18.414	3.295	1.00	0.00	H
ATOM	216	C	TYR	14	-10.573	17.673	1.768	1.00	0.00	C

ATOM	217	O	TYR	14	-10.246	18.844	1.981	1.00	0.00	O
ATOM	218	N	GLU	15	-10.230	16.757	2.719	1.00	0.00	N
ATOM	219	H	GLU	15	-10.714	15.878	2.604	1.00	0.00	H
ATOM	220	CA	GLU	15	-9.418	16.974	3.900	1.00	0.00	C
ATOM	221	HA	GLU	15	-9.518	18.022	4.182	1.00	0.00	H
ATOM	222	CB	GLU	15	-9.993	16.190	5.064	1.00	0.00	C
ATOM	223	HB2	GLU	15	-11.067	16.010	5.027	1.00	0.00	H
ATOM	224	HB3	GLU	15	-9.404	15.274	5.082	1.00	0.00	H
ATOM	225	CG	GLU	15	-9.695	16.893	6.403	1.00	0.00	C
ATOM	226	HG2	GLU	15	-9.790	16.102	7.147	1.00	0.00	H
ATOM	227	HG3	GLU	15	-8.632	17.122	6.459	1.00	0.00	H
ATOM	228	CD	GLU	15	-10.625	18.090	6.749	1.00	0.00	C
ATOM	229	OE1	GLU	15	-10.376	19.146	6.153	1.00	0.00	O
ATOM	230	OE2	GLU	15	-11.486	18.101	7.623	1.00	0.00	O
ATOM	231	C	GLU	15	-7.993	16.591	3.665	1.00	0.00	C
ATOM	232	O	GLU	15	-7.132	17.391	3.932	1.00	0.00	O
ATOM	233	N	ASP	16	-7.730	15.466	2.955	1.00	0.00	N
ATOM	234	H	ASP	16	-8.505	14.827	2.848	1.00	0.00	H
ATOM	235	CA	ASP	16	-6.392	15.080	2.461	1.00	0.00	C
ATOM	236	HA	ASP	16	-5.764	14.841	3.319	1.00	0.00	H
ATOM	237	CB	ASP	16	-6.408	13.829	1.602	1.00	0.00	C
ATOM	238	HB2	ASP	16	-7.027	13.138	2.175	1.00	0.00	H
ATOM	239	HB3	ASP	16	-6.820	13.885	0.593	1.00	0.00	H
ATOM	240	CG	ASP	16	-5.026	13.271	1.173	1.00	0.00	C
ATOM	241	OD1	ASP	16	-4.103	13.204	2.009	1.00	0.00	O
ATOM	242	OD2	ASP	16	-4.818	13.045	-0.032	1.00	0.00	O
ATOM	243	C	ASP	16	-5.694	16.236	1.683	1.00	0.00	C
ATOM	244	O	ASP	16	-4.461	16.373	1.792	1.00	0.00	O
ATOM	245	N	ARG	17	-6.408	17.037	0.833	1.00	0.00	N
ATOM	246	H	ARG	17	-7.366	16.793	0.625	1.00	0.00	H
ATOM	247	CA	ARG	17	-5.852	18.106	-0.016	1.00	0.00	C
ATOM	248	HA	ARG	17	-5.240	17.573	-0.744	1.00	0.00	H
ATOM	249	CB	ARG	17	-7.008	18.649	-0.825	1.00	0.00	C
ATOM	250	HB2	ARG	17	-7.278	17.959	-1.624	1.00	0.00	H
ATOM	251	HB3	ARG	17	-7.860	18.780	-0.158	1.00	0.00	H
ATOM	252	CG	ARG	17	-6.793	19.971	-1.573	1.00	0.00	C
ATOM	253	HG2	ARG	17	-6.653	20.733	-0.806	1.00	0.00	H
ATOM	254	HG3	ARG	17	-5.878	20.029	-2.161	1.00	0.00	H
ATOM	255	CD	ARG	17	-7.983	20.377	-2.382	1.00	0.00	C
ATOM	256	HD2	ARG	17	-8.900	20.403	-1.794	1.00	0.00	H
ATOM	257	HD3	ARG	17	-7.793	21.404	-2.694	1.00	0.00	H
ATOM	258	NE	ARG	17	-8.172	19.436	-3.555	1.00	0.00	N
ATOM	259	HE	ARG	17	-7.730	19.736	-4.413	1.00	0.00	H
ATOM	260	CZ	ARG	17	-9.033	18.423	-3.739	1.00	0.00	C
ATOM	261	NH1	ARG	17	-9.727	18.010	-2.782	1.00	0.00	N
ATOM	262	HH11	ARG	17	-9.572	18.481	-1.902	1.00	0.00	H
ATOM	263	HH12	ARG	17	-9.929	17.021	-2.749	1.00	0.00	H
ATOM	264	NH2	ARG	17	-9.106	17.770	-4.800	1.00	0.00	N
ATOM	265	HH21	ARG	17	-8.552	17.920	-5.631	1.00	0.00	H
ATOM	266	HH22	ARG	17	-9.881	17.129	-4.895	1.00	0.00	H
ATOM	267	C	ARG	17	-5.046	19.204	0.749	1.00	0.00	C
ATOM	268	O	ARG	17	-4.015	19.704	0.337	1.00	0.00	O
ATOM	269	N	TYR	18	-5.506	19.553	1.940	1.00	0.00	N
ATOM	270	H	TYR	18	-6.289	19.031	2.307	1.00	0.00	H
ATOM	271	CA	TYR	18	-4.779	20.468	2.907	1.00	0.00	C
ATOM	272	HA	TYR	18	-4.791	21.516	2.609	1.00	0.00	H
ATOM	273	CB	TYR	18	-5.599	20.528	4.160	1.00	0.00	C
ATOM	274	HB2	TYR	18	-6.635	20.702	3.871	1.00	0.00	H
ATOM	275	HB3	TYR	18	-5.667	19.582	4.697	1.00	0.00	H
ATOM	276	CG	TYR	18	-5.224	21.582	5.219	1.00	0.00	C
ATOM	277	CD1	TYR	18	-5.649	22.939	5.149	1.00	0.00	C
ATOM	278	HD1	TYR	18	-6.209	23.313	4.305	1.00	0.00	H
ATOM	279	CE1	TYR	18	-5.254	23.782	6.218	1.00	0.00	C

ATOM	280	HE1	TYR	18	-5.474	24.839	6.213	1.00	0.00	H
ATOM	281	CZ	TYR	18	-4.424	23.356	7.290	1.00	0.00	C
ATOM	282	OH	TYR	18	-4.027	24.176	8.291	1.00	0.00	O
ATOM	283	HH	TYR	18	-4.267	25.090	8.119	1.00	0.00	H
ATOM	284	CE2	TYR	18	-4.135	21.993	7.356	1.00	0.00	C
ATOM	285	HE2	TYR	18	-3.563	21.616	8.192	1.00	0.00	H
ATOM	286	CD2	TYR	18	-4.530	21.101	6.363	1.00	0.00	C
ATOM	287	HD2	TYR	18	-4.223	20.070	6.455	1.00	0.00	H
ATOM	288	C	TYR	18	-3.442	19.955	3.189	1.00	0.00	C
ATOM	289	O	TYR	18	-2.406	20.672	3.133	1.00	0.00	O
ATOM	290	N	TYR	19	-3.303	18.626	3.388	1.00	0.00	N
ATOM	291	H	TYR	19	-4.079	18.009	3.193	1.00	0.00	H
ATOM	292	CA	TYR	19	-2.036	18.046	3.676	1.00	0.00	C
ATOM	293	HA	TYR	19	-1.388	18.613	4.344	1.00	0.00	H
ATOM	294	CB	TYR	19	-2.345	16.717	4.439	1.00	0.00	C
ATOM	295	HB2	TYR	19	-2.907	15.977	3.869	1.00	0.00	H
ATOM	296	HB3	TYR	19	-1.416	16.162	4.573	1.00	0.00	H
ATOM	297	CG	TYR	19	-3.061	16.953	5.753	1.00	0.00	C
ATOM	298	CD1	TYR	19	-4.418	16.700	5.855	1.00	0.00	C
ATOM	299	HD1	TYR	19	-4.927	16.299	4.990	1.00	0.00	H
ATOM	300	CE1	TYR	19	-5.107	16.980	7.032	1.00	0.00	C
ATOM	301	HE1	TYR	19	-6.151	16.761	7.199	1.00	0.00	H
ATOM	302	CZ	TYR	19	-4.334	17.489	8.084	1.00	0.00	C
ATOM	303	OH	TYR	19	-4.959	17.761	9.296	1.00	0.00	O
ATOM	304	HH	TYR	19	-5.914	17.694	9.227	1.00	0.00	H
ATOM	305	CE2	TYR	19	-2.969	17.788	8.038	1.00	0.00	C
ATOM	306	HE2	TYR	19	-2.474	18.148	8.928	1.00	0.00	H
ATOM	307	CD2	TYR	19	-2.313	17.457	6.842	1.00	0.00	C
ATOM	308	HD2	TYR	19	-1.235	17.523	6.806	1.00	0.00	H
ATOM	309	C	TYR	19	-1.157	17.729	2.451	1.00	0.00	C
ATOM	310	O	TYR	19	0.052	17.698	2.536	1.00	0.00	O
ATOM	311	N	ARG	20	-1.832	17.389	1.328	1.00	0.00	N
ATOM	312	H	ARG	20	-2.828	17.234	1.393	1.00	0.00	H
ATOM	313	CA	ARG	20	-1.342	17.223	-0.042	1.00	0.00	C
ATOM	314	HA	ARG	20	-0.685	16.357	-0.127	1.00	0.00	H
ATOM	315	CB	ARG	20	-2.503	16.923	-0.944	1.00	0.00	C
ATOM	316	HB2	ARG	20	-3.200	16.274	-0.414	1.00	0.00	H
ATOM	317	HB3	ARG	20	-3.026	17.833	-1.237	1.00	0.00	H
ATOM	318	CG	ARG	20	-2.137	16.235	-2.266	1.00	0.00	C
ATOM	319	HG2	ARG	20	-1.513	16.989	-2.746	1.00	0.00	H
ATOM	320	HG3	ARG	20	-1.568	15.320	-2.096	1.00	0.00	H
ATOM	321	CD	ARG	20	-3.391	15.936	-3.127	1.00	0.00	C
ATOM	322	HD2	ARG	20	-3.865	16.897	-3.328	1.00	0.00	H
ATOM	323	HD3	ARG	20	-2.912	15.578	-4.038	1.00	0.00	H
ATOM	324	NE	ARG	20	-4.382	15.001	-2.512	1.00	0.00	N
ATOM	325	HE	ARG	20	-4.052	14.382	-1.784	1.00	0.00	H
ATOM	326	CZ	ARG	20	-5.637	14.929	-2.793	1.00	0.00	C
ATOM	327	NH1	ARG	20	-6.251	15.479	-3.793	1.00	0.00	N
ATOM	328	HH11	ARG	20	-5.768	16.103	-4.426	1.00	0.00	H
ATOM	329	HH12	ARG	20	-7.191	15.176	-4.005	1.00	0.00	H
ATOM	330	NH2	ARG	20	-6.435	14.110	-2.140	1.00	0.00	N
ATOM	331	HH21	ARG	20	-6.122	13.600	-1.326	1.00	0.00	H
ATOM	332	HH22	ARG	20	-7.392	14.121	-2.463	1.00	0.00	H
ATOM	333	C	ARG	20	-0.571	18.466	-0.514	1.00	0.00	C
ATOM	334	O	ARG	20	0.513	18.477	-1.045	1.00	0.00	O
ATOM	335	N	GLU	21	-1.095	19.597	-0.144	1.00	0.00	N
ATOM	336	H	GLU	21	-2.014	19.656	0.269	1.00	0.00	H
ATOM	337	CA	GLU	21	-0.527	20.880	-0.522	1.00	0.00	C
ATOM	338	HA	GLU	21	-0.015	20.849	-1.483	1.00	0.00	H
ATOM	339	CB	GLU	21	-1.624	21.927	-0.569	1.00	0.00	C
ATOM	340	HB2	GLU	21	-2.242	21.694	0.299	1.00	0.00	H
ATOM	341	HB3	GLU	21	-1.231	22.944	-0.559	1.00	0.00	H
ATOM	342	CG	GLU	21	-2.456	21.839	-1.801	1.00	0.00	C

ATOM	343	HG2	GLU	21	-1.830	21.929	-2.688	1.00	0.00	H
ATOM	344	HG3	GLU	21	-2.869	20.830	-1.791	1.00	0.00	H
ATOM	345	CD	GLU	21	-3.555	22.965	-1.844	1.00	0.00	C
ATOM	346	OE1	GLU	21	-4.596	22.861	-1.207	1.00	0.00	O
ATOM	347	OE2	GLU	21	-3.219	24.142	-2.279	1.00	0.00	O
ATOM	348	C	GLU	21	0.575	21.241	0.548	1.00	0.00	C
ATOM	349	O	GLU	21	1.456	22.021	0.264	1.00	0.00	O
ATOM	350	N	ASN	22	0.650	20.588	1.710	1.00	0.00	N
ATOM	351	H	ASN	22	-0.006	19.839	1.883	1.00	0.00	H
ATOM	352	CA	ASN	22	1.598	20.570	2.796	1.00	0.00	C
ATOM	353	HA	ASN	22	1.322	20.018	3.695	1.00	0.00	H
ATOM	354	CB	ASN	22	2.797	19.805	2.237	1.00	0.00	C
ATOM	355	HB2	ASN	22	2.564	19.017	1.522	1.00	0.00	H
ATOM	356	HB3	ASN	22	3.382	20.392	1.530	1.00	0.00	H
ATOM	357	CG	ASN	22	3.662	19.201	3.268	1.00	0.00	C
ATOM	358	OD1	ASN	22	3.675	19.589	4.429	1.00	0.00	O
ATOM	359	ND2	ASN	22	4.496	18.243	2.919	1.00	0.00	N
ATOM	360	HD21	ASN	22	4.952	17.786	3.695	1.00	0.00	H
ATOM	361	HD22	ASN	22	4.522	17.971	1.947	1.00	0.00	H
ATOM	362	C	ASN	22	1.793	22.023	3.287	1.00	0.00	C
ATOM	363	O	ASN	22	0.887	22.814	3.300	1.00	0.00	O
ATOM	364	N	MET	23	3.001	22.317	3.765	1.00	0.00	N
ATOM	365	H	MET	23	3.561	21.491	3.920	1.00	0.00	H
ATOM	366	CA	MET	23	3.391	23.525	4.383	1.00	0.00	C
ATOM	367	HA	MET	23	2.829	23.493	5.316	1.00	0.00	H
ATOM	368	CB	MET	23	4.904	23.454	4.754	1.00	0.00	C
ATOM	369	HB2	MET	23	5.176	24.378	5.266	1.00	0.00	H
ATOM	370	HB3	MET	23	5.032	22.583	5.398	1.00	0.00	H
ATOM	371	CG	MET	23	5.692	23.271	3.452	1.00	0.00	C
ATOM	372	HG2	MET	23	5.418	22.354	2.931	1.00	0.00	H
ATOM	373	HG3	MET	23	5.424	24.120	2.824	1.00	0.00	H
ATOM	374	SD	MET	23	7.487	23.434	3.685	1.00	0.00	S
ATOM	375	CE	MET	23	8.071	21.939	4.499	1.00	0.00	C
ATOM	376	HE1	MET	23	9.141	22.041	4.678	1.00	0.00	H
ATOM	377	HE2	MET	23	7.565	21.846	5.460	1.00	0.00	H
ATOM	378	HE3	MET	23	7.871	21.025	3.939	1.00	0.00	H
ATOM	379	C	MET	23	3.050	24.871	3.750	1.00	0.00	C
ATOM	380	O	MET	23	3.104	25.845	4.489	1.00	0.00	O
ATOM	381	N	TYR	24	2.573	24.944	2.532	1.00	0.00	N
ATOM	382	H	TYR	24	2.635	24.079	2.015	1.00	0.00	H
ATOM	383	CA	TYR	24	1.924	26.142	1.893	1.00	0.00	C
ATOM	384	HA	TYR	24	2.539	27.006	2.142	1.00	0.00	H
ATOM	385	CB	TYR	24	1.670	26.034	0.355	1.00	0.00	C
ATOM	386	HB2	TYR	24	1.449	25.009	0.060	1.00	0.00	H
ATOM	387	HB3	TYR	24	0.807	26.639	0.077	1.00	0.00	H
ATOM	388	CG	TYR	24	2.922	26.499	-0.410	1.00	0.00	C
ATOM	389	CD1	TYR	24	4.050	25.723	-0.468	1.00	0.00	C
ATOM	390	HD1	TYR	24	4.035	24.773	0.043	1.00	0.00	H
ATOM	391	CE1	TYR	24	5.217	26.130	-1.154	1.00	0.00	C
ATOM	392	HE1	TYR	24	6.078	25.481	-1.095	1.00	0.00	H
ATOM	393	CZ	TYR	24	5.207	27.378	-1.752	1.00	0.00	C
ATOM	394	OH	TYR	24	6.297	27.862	-2.403	1.00	0.00	O
ATOM	395	HH	TYR	24	7.112	27.355	-2.439	1.00	0.00	H
ATOM	396	CE2	TYR	24	4.042	28.201	-1.775	1.00	0.00	C
ATOM	397	HE2	TYR	24	4.160	29.191	-2.190	1.00	0.00	H
ATOM	398	CD2	TYR	24	2.903	27.755	-1.044	1.00	0.00	C
ATOM	399	HD2	TYR	24	2.019	28.375	-1.025	1.00	0.00	H
ATOM	400	C	TYR	24	0.527	26.568	2.555	1.00	0.00	C
ATOM	401	O	TYR	24	0.063	27.751	2.404	1.00	0.00	O
ATOM	402	N	ARG	25	-0.094	25.728	3.346	1.00	0.00	N
ATOM	403	H	ARG	25	0.281	24.798	3.472	1.00	0.00	H
ATOM	404	CA	ARG	25	-1.471	25.849	3.843	1.00	0.00	C
ATOM	405	HA	ARG	25	-1.804	26.831	3.504	1.00	0.00	H

```
ATOM    406  CB  ARG    25      -2.401  24.800   3.345  1.00  0.00           C
ATOM    407  HB2 ARG    25      -1.959  23.809   3.442  1.00  0.00           H
ATOM    408  HB3 ARG    25      -3.304  24.868   3.952  1.00  0.00           H
ATOM    409  CG  ARG    25      -2.834  24.904   1.886  1.00  0.00           C
ATOM    410  HG2 ARG    25      -1.987  24.669   1.241  1.00  0.00           H
ATOM    411  HG3 ARG    25      -3.422  24.030   1.605  1.00  0.00           H
ATOM    412  CD  ARG    25      -3.462  26.190   1.357  1.00  0.00           C
ATOM    413  HD2 ARG    25      -4.224  26.528   2.060  1.00  0.00           H
ATOM    414  HD3 ARG    25      -2.732  27.000   1.321  1.00  0.00           H
ATOM    415  NE  ARG    25      -4.032  26.051   0.026  1.00  0.00           N
ATOM    416  HE  ARG    25      -3.541  25.520  -0.680  1.00  0.00           H
ATOM    417  CZ  ARG    25      -5.180  26.464  -0.453  1.00  0.00           C
ATOM    418  NH1 ARG    25      -5.883  27.321   0.241  1.00  0.00           N
ATOM    419 HH11 ARG    25      -5.540  27.597   1.150  1.00  0.00           H
ATOM    420 HH12 ARG    25      -6.826  27.502  -0.074  1.00  0.00           H
ATOM    421  NH2 ARG    25      -5.396  26.132  -1.669  1.00  0.00           N
ATOM    422 HH21 ARG    25      -4.911  25.369  -2.120  1.00  0.00           H
ATOM    423 HH22 ARG    25      -6.312  26.300  -2.061  1.00  0.00           H
ATOM    424  C   ARG    25      -1.501  25.965   5.367  1.00  0.00           C
ATOM    425  O   ARG    25      -2.533  26.461   5.890  1.00  0.00           O
ATOM    426  N   TYR    26      -0.455  25.484   6.090  1.00  0.00           N
ATOM    427  H   TYR    26       0.301  25.099   5.542  1.00  0.00           H
ATOM    428  CA  TYR    26      -0.450  25.288   7.581  1.00  0.00           C
ATOM    429  HA  TYR    26      -1.386  24.815   7.882  1.00  0.00           H
ATOM    430  CB  TYR    26       0.759  24.404   8.005  1.00  0.00           C
ATOM    431  HB2 TYR    26       1.698  24.915   7.792  1.00  0.00           H
ATOM    432  HB3 TYR    26       0.747  24.293   9.089  1.00  0.00           H
ATOM    433  CG  TYR    26       0.787  22.981   7.383  1.00  0.00           C
ATOM    434  CD1 TYR    26       1.938  22.126   7.580  1.00  0.00           C
ATOM    435  HD1 TYR    26       2.804  22.514   8.096  1.00  0.00           H
ATOM    436  CE1 TYR    26       1.974  20.772   7.122  1.00  0.00           C
ATOM    437  HE1 TYR    26       2.879  20.194   7.242  1.00  0.00           H
ATOM    438  CZ  TYR    26       0.901  20.234   6.378  1.00  0.00           C
ATOM    439  OH  TYR    26       0.920  18.927   6.032  1.00  0.00           O
ATOM    440  HH  TYR    26       1.829  18.618   6.071  1.00  0.00           H
ATOM    441  CE2 TYR    26      -0.273  21.004   6.305  1.00  0.00           C
ATOM    442  HE2 TYR    26      -1.188  20.594   5.904  1.00  0.00           H
ATOM    443  CD2 TYR    26      -0.338  22.336   6.786  1.00  0.00           C
ATOM    444  HD2 TYR    26      -1.301  22.823   6.818  1.00  0.00           H
ATOM    445  C   TYR    26      -0.467  26.652   8.337  1.00  0.00           C
ATOM    446  O   TYR    26      -0.855  26.733   9.507  1.00  0.00           O
ATOM    447  N   NME    27      -0.060  27.821   7.643  1.00  0.00           N
ATOM    448  H   NME    27       0.277  27.734   6.694  1.00  0.00           H
ATOM    449  CH3 NME    27      -0.226  29.160   8.157  1.00  0.00           C
ATOM    450 HH31 NME    27       0.035  29.837   7.344  1.00  0.00           H
ATOM    451 HH32 NME    27      -1.222  29.250   8.592  1.00  0.00           H
ATOM    452 HH33 NME    27       0.482  29.390   8.953  1.00  0.00           H
TER     453      NME    27
END
```

Cluster 2, Figure 1A (brown):

```
ATOM      1 HH31 ACE     1       4.041  17.070   8.078  1.00  0.00           H
ATOM      2  CH3 ACE     1       4.698  16.259   7.766  1.00  0.00           C
ATOM      3 HH32 ACE     1       4.493  15.326   8.292  1.00  0.00           H
ATOM      4 HH33 ACE     1       5.754  16.532   7.749  1.00  0.00           H
ATOM      5  C   ACE     1       4.102  16.061   6.403  1.00  0.00           C
ATOM      6  O   ACE     1       3.471  14.994   6.205  1.00  0.00           O
ATOM      7  N   VAL     2       4.398  16.890   5.409  1.00  0.00           N
ATOM      8  H   VAL     2       5.056  17.646   5.534  1.00  0.00           H
ATOM      9  CA  VAL     2       3.835  16.862   4.069  1.00  0.00           C
```

ATOM	10	HA	VAL	2	2.767	16.670	4.178	1.00	0.00	H
ATOM	11	CB	VAL	2	4.001	18.163	3.352	1.00	0.00	C
ATOM	12	HB	VAL	2	3.471	18.064	2.404	1.00	0.00	H
ATOM	13	CG1	VAL	2	3.375	19.323	4.074	1.00	0.00	C
ATOM	14	HG11	VAL	2	2.326	19.128	4.298	1.00	0.00	H
ATOM	15	HG12	VAL	2	3.799	19.730	4.992	1.00	0.00	H
ATOM	16	HG13	VAL	2	3.318	20.094	3.306	1.00	0.00	H
ATOM	17	CG2	VAL	2	5.430	18.598	2.954	1.00	0.00	C
ATOM	18	HG21	VAL	2	6.103	18.461	3.800	1.00	0.00	H
ATOM	19	HG22	VAL	2	5.838	17.950	2.177	1.00	0.00	H
ATOM	20	HG23	VAL	2	5.408	19.619	2.573	1.00	0.00	H
ATOM	21	C	VAL	2	4.337	15.698	3.203	1.00	0.00	C
ATOM	22	O	VAL	2	3.945	15.488	2.054	1.00	0.00	O
ATOM	23	N	MET	3	5.276	14.855	3.659	1.00	0.00	N
ATOM	24	H	MET	3	5.814	15.169	4.453	1.00	0.00	H
ATOM	25	CA	MET	3	5.635	13.556	2.968	1.00	0.00	C
ATOM	26	HA	MET	3	5.646	13.723	1.892	1.00	0.00	H
ATOM	27	CB	MET	3	7.140	13.055	3.200	1.00	0.00	C
ATOM	28	HB2	MET	3	7.208	12.045	2.793	1.00	0.00	H
ATOM	29	HB3	MET	3	7.781	13.727	2.631	1.00	0.00	H
ATOM	30	CG	MET	3	7.628	12.990	4.699	1.00	0.00	C
ATOM	31	HG2	MET	3	7.156	12.245	5.339	1.00	0.00	H
ATOM	32	HG3	MET	3	8.658	12.672	4.534	1.00	0.00	H
ATOM	33	SD	MET	3	7.759	14.457	5.658	1.00	0.00	S
ATOM	34	CE	MET	3	8.987	14.042	6.916	1.00	0.00	C
ATOM	35	HE1	MET	3	8.623	13.273	7.598	1.00	0.00	H
ATOM	36	HE2	MET	3	9.882	13.619	6.460	1.00	0.00	H
ATOM	37	HE3	MET	3	9.251	14.884	7.556	1.00	0.00	H
ATOM	38	C	MET	3	4.591	12.472	3.285	1.00	0.00	C
ATOM	39	O	MET	3	4.621	11.425	2.564	1.00	0.00	O
ATOM	40	N	SER	4	3.617	12.684	4.170	1.00	0.00	N
ATOM	41	H	SER	4	3.569	13.481	4.787	1.00	0.00	H
ATOM	42	CA	SER	4	2.510	11.789	4.376	1.00	0.00	C
ATOM	43	HA	SER	4	2.704	10.839	3.877	1.00	0.00	H
ATOM	44	CB	SER	4	2.279	11.516	5.855	1.00	0.00	C
ATOM	45	HB2	SER	4	2.146	12.481	6.345	1.00	0.00	H
ATOM	46	HB3	SER	4	1.402	10.878	5.966	1.00	0.00	H
ATOM	47	OG	SER	4	3.475	10.913	6.343	1.00	0.00	O
ATOM	48	HG	SER	4	3.506	11.024	7.297	1.00	0.00	H
ATOM	49	C	SER	4	1.174	12.342	3.728	1.00	0.00	C
ATOM	50	O	SER	4	0.833	13.531	3.774	1.00	0.00	O
ATOM	51	N	ARG	5	0.486	11.454	3.059	1.00	0.00	N
ATOM	52	H	ARG	5	0.848	10.514	3.138	1.00	0.00	H
ATOM	53	CA	ARG	5	-0.526	11.760	2.049	1.00	0.00	C
ATOM	54	HA	ARG	5	-0.977	12.684	2.415	1.00	0.00	H
ATOM	55	CB	ARG	5	0.201	12.010	0.682	1.00	0.00	C
ATOM	56	HB2	ARG	5	0.798	11.141	0.406	1.00	0.00	H
ATOM	57	HB3	ARG	5	-0.524	12.090	-0.128	1.00	0.00	H
ATOM	58	CG	ARG	5	1.100	13.321	0.530	1.00	0.00	C
ATOM	59	HG2	ARG	5	1.896	13.313	1.276	1.00	0.00	H
ATOM	60	HG3	ARG	5	1.456	13.122	-0.481	1.00	0.00	H
ATOM	61	CD	ARG	5	0.274	14.609	0.561	1.00	0.00	C
ATOM	62	HD2	ARG	5	0.061	14.892	1.592	1.00	0.00	H
ATOM	63	HD3	ARG	5	0.900	15.424	0.199	1.00	0.00	H
ATOM	64	NE	ARG	5	-0.976	14.512	-0.302	1.00	0.00	N
ATOM	65	HE	ARG	5	-1.835	14.194	0.123	1.00	0.00	H
ATOM	66	CZ	ARG	5	-1.013	14.589	-1.585	1.00	0.00	C
ATOM	67	NH1	ARG	5	0.005	15.001	-2.324	1.00	0.00	N
ATOM	68	HH11	ARG	5	0.743	15.420	-1.776	1.00	0.00	H
ATOM	69	HH12	ARG	5	-0.076	15.103	-3.325	1.00	0.00	H
ATOM	70	NH2	ARG	5	-2.144	14.238	-2.155	1.00	0.00	N
ATOM	71	HH21	ARG	5	-2.199	14.336	-3.159	1.00	0.00	H
ATOM	72	HH22	ARG	5	-3.010	14.003	-1.690	1.00	0.00	H

ATOM	73	C	ARG	5	-1.683	10.697	2.063	1.00	0.00	C
ATOM	74	O	ARG	5	-1.511	9.711	1.332	1.00	0.00	O
ATOM	75	N	PRO	6	-2.788	10.825	2.849	1.00	0.00	N
ATOM	76	CD	PRO	6	-3.034	11.907	3.812	1.00	0.00	C
ATOM	77	HD2	PRO	6	-3.615	12.730	3.396	1.00	0.00	H
ATOM	78	HD3	PRO	6	-2.120	12.259	4.292	1.00	0.00	H
ATOM	79	CG	PRO	6	-4.009	11.329	4.827	1.00	0.00	C
ATOM	80	HG2	PRO	6	-4.612	12.148	5.218	1.00	0.00	H
ATOM	81	HG3	PRO	6	-3.491	10.801	5.629	1.00	0.00	H
ATOM	82	CB	PRO	6	-4.837	10.341	3.888	1.00	0.00	C
ATOM	83	HB2	PRO	6	-5.618	10.821	3.298	1.00	0.00	H
ATOM	84	HB3	PRO	6	-5.315	9.549	4.465	1.00	0.00	H
ATOM	85	CA	PRO	6	-3.785	9.780	2.879	1.00	0.00	C
ATOM	86	HA	PRO	6	-3.353	8.941	3.427	1.00	0.00	H
ATOM	87	C	PRO	6	-4.346	9.356	1.477	1.00	0.00	C
ATOM	88	O	PRO	6	-4.831	8.287	1.325	1.00	0.00	O
ATOM	89	N	LEU	7	-4.274	10.256	0.507	1.00	0.00	N
ATOM	90	H	LEU	7	-4.103	11.221	0.755	1.00	0.00	H
ATOM	91	CA	LEU	7	-4.983	10.020	-0.823	1.00	0.00	C
ATOM	92	HA	LEU	7	-6.054	9.862	-0.692	1.00	0.00	H
ATOM	93	CB	LEU	7	-4.833	11.352	-1.676	1.00	0.00	C
ATOM	94	HB2	LEU	7	-5.218	12.264	-1.218	1.00	0.00	H
ATOM	95	HB3	LEU	7	-3.757	11.522	-1.684	1.00	0.00	H
ATOM	96	CG	LEU	7	-5.401	11.334	-3.124	1.00	0.00	C
ATOM	97	HG	LEU	7	-4.960	10.655	-3.856	1.00	0.00	H
ATOM	98	CD1	LEU	7	-6.874	10.988	-3.193	1.00	0.00	C
ATOM	99	HD11	LEU	7	-7.140	9.978	-2.882	1.00	0.00	H
ATOM	100	HD12	LEU	7	-7.559	11.662	-2.680	1.00	0.00	H
ATOM	101	HD13	LEU	7	-7.207	11.077	-4.228	1.00	0.00	H
ATOM	102	CD2	LEU	7	-5.201	12.706	-3.675	1.00	0.00	C
ATOM	103	HD21	LEU	7	-5.707	13.497	-3.122	1.00	0.00	H
ATOM	104	HD22	LEU	7	-4.140	12.938	-3.776	1.00	0.00	H
ATOM	105	HD23	LEU	7	-5.602	12.782	-4.685	1.00	0.00	H
ATOM	106	C	LEU	7	-4.495	8.832	-1.603	1.00	0.00	C
ATOM	107	O	LEU	7	-5.289	8.331	-2.420	1.00	0.00	O
ATOM	108	N	ILE	8	-3.190	8.433	-1.316	1.00	0.00	N
ATOM	109	H	ILE	8	-2.713	8.998	-0.628	1.00	0.00	H
ATOM	110	CA	ILE	8	-2.522	7.196	-1.797	1.00	0.00	C
ATOM	111	HA	ILE	8	-3.125	6.767	-2.598	1.00	0.00	H
ATOM	112	CB	ILE	8	-1.245	7.499	-2.587	1.00	0.00	C
ATOM	113	HB	ILE	8	-0.749	6.602	-2.958	1.00	0.00	H
ATOM	114	CG2	ILE	8	-1.670	8.305	-3.803	1.00	0.00	C
ATOM	115	HG21	ILE	8	-2.337	9.141	-3.594	1.00	0.00	H
ATOM	116	HG22	ILE	8	-0.768	8.710	-4.258	1.00	0.00	H
ATOM	117	HG23	ILE	8	-2.149	7.701	-4.575	1.00	0.00	H
ATOM	118	CG1	ILE	8	-0.173	8.276	-1.791	1.00	0.00	C
ATOM	119	HG12	ILE	8	-0.315	9.356	-1.848	1.00	0.00	H
ATOM	120	HG13	ILE	8	-0.202	7.850	-0.788	1.00	0.00	H
ATOM	121	CD1	ILE	8	1.230	7.873	-2.312	1.00	0.00	C
ATOM	122	HD11	ILE	8	1.270	7.783	-3.397	1.00	0.00	H
ATOM	123	HD12	ILE	8	2.021	8.579	-2.061	1.00	0.00	H
ATOM	124	HD13	ILE	8	1.514	6.873	-1.985	1.00	0.00	H
ATOM	125	C	ILE	8	-2.482	5.893	-0.907	1.00	0.00	C
ATOM	126	O	ILE	8	-1.758	4.934	-1.155	1.00	0.00	O
ATOM	127	N	HID	9	-3.327	5.888	0.168	1.00	0.00	N
ATOM	128	H	HID	9	-3.702	6.756	0.525	1.00	0.00	H
ATOM	129	CA	HID	9	-3.558	4.662	0.968	1.00	0.00	C
ATOM	130	HA	HID	9	-3.305	3.859	0.277	1.00	0.00	H
ATOM	131	CB	HID	9	-2.667	4.521	2.282	1.00	0.00	C
ATOM	132	HB2	HID	9	-2.824	5.354	2.968	1.00	0.00	H
ATOM	133	HB3	HID	9	-3.089	3.692	2.849	1.00	0.00	H
ATOM	134	CG	HID	9	-1.247	4.283	2.003	1.00	0.00	C
ATOM	135	ND1	HID	9	-0.615	3.123	1.555	1.00	0.00	N

ATOM	136	HD1	HID	9	-1.031	2.203	1.578	1.00	0.00	H
ATOM	137	CE1	HID	9	0.686	3.460	1.335	1.00	0.00	C
ATOM	138	HE1	HID	9	1.488	2.809	1.020	1.00	0.00	H
ATOM	139	NE2	HID	9	0.864	4.803	1.476	1.00	0.00	N
ATOM	140	CD2	HID	9	-0.351	5.342	1.978	1.00	0.00	C
ATOM	141	HD2	HID	9	-0.584	6.353	2.280	1.00	0.00	H
ATOM	142	C	HID	9	-5.014	4.467	1.294	1.00	0.00	C
ATOM	143	O	HID	9	-5.646	3.547	0.797	1.00	0.00	O
ATOM	144	N	PHE	10	-5.552	5.298	2.209	1.00	0.00	N
ATOM	145	H	PHE	10	-5.184	6.230	2.344	1.00	0.00	H
ATOM	146	CA	PHE	10	-6.993	5.268	2.628	1.00	0.00	C
ATOM	147	HA	PHE	10	-7.262	4.212	2.678	1.00	0.00	H
ATOM	148	CB	PHE	10	-6.990	5.915	4.076	1.00	0.00	C
ATOM	149	HB2	PHE	10	-6.816	6.981	3.928	1.00	0.00	H
ATOM	150	HB3	PHE	10	-7.989	5.757	4.482	1.00	0.00	H
ATOM	151	CG	PHE	10	-6.061	5.259	5.096	1.00	0.00	C
ATOM	152	CD1	PHE	10	-4.818	5.864	5.388	1.00	0.00	C
ATOM	153	HD1	PHE	10	-4.653	6.903	5.145	1.00	0.00	H
ATOM	154	CE1	PHE	10	-3.779	5.175	6.001	1.00	0.00	C
ATOM	155	HE1	PHE	10	-2.863	5.672	6.284	1.00	0.00	H
ATOM	156	CZ	PHE	10	-3.974	3.922	6.537	1.00	0.00	C
ATOM	157	HZ	PHE	10	-3.201	3.386	7.067	1.00	0.00	H
ATOM	158	CE2	PHE	10	-5.169	3.223	6.237	1.00	0.00	C
ATOM	159	HE2	PHE	10	-5.350	2.261	6.696	1.00	0.00	H
ATOM	160	CD2	PHE	10	-6.261	3.934	5.621	1.00	0.00	C
ATOM	161	HD2	PHE	10	-7.149	3.416	5.292	1.00	0.00	H
ATOM	162	C	PHE	10	-7.973	5.958	1.678	1.00	0.00	C
ATOM	163	O	PHE	10	-9.162	5.591	1.619	1.00	0.00	O
ATOM	164	N	GLY	11	-7.555	7.065	1.004	1.00	0.00	N
ATOM	165	H	GLY	11	-6.565	7.265	0.971	1.00	0.00	H
ATOM	166	CA	GLY	11	-8.522	7.858	0.218	1.00	0.00	C
ATOM	167	HA2	GLY	11	-7.895	8.368	-0.511	1.00	0.00	H
ATOM	168	HA3	GLY	11	-9.340	7.323	-0.265	1.00	0.00	H
ATOM	169	C	GLY	11	-9.180	8.918	1.095	1.00	0.00	C
ATOM	170	O	GLY	11	-10.131	9.529	0.664	1.00	0.00	O
ATOM	171	N	ASN	12	-8.717	9.217	2.345	1.00	0.00	N
ATOM	172	H	ASN	12	-7.889	8.753	2.690	1.00	0.00	H
ATOM	173	CA	ASN	12	-9.293	10.319	3.145	1.00	0.00	C
ATOM	174	HA	ASN	12	-10.382	10.339	3.099	1.00	0.00	H
ATOM	175	CB	ASN	12	-8.968	10.005	4.612	1.00	0.00	C
ATOM	176	HB2	ASN	12	-7.878	10.000	4.634	1.00	0.00	H
ATOM	177	HB3	ASN	12	-9.429	10.786	5.217	1.00	0.00	H
ATOM	178	CG	ASN	12	-9.341	8.733	5.316	1.00	0.00	C
ATOM	179	OD1	ASN	12	-9.612	7.674	4.754	1.00	0.00	O
ATOM	180	ND2	ASN	12	-9.366	8.770	6.632	1.00	0.00	N
ATOM	181	HD21	ASN	12	-9.662	7.990	7.203	1.00	0.00	H
ATOM	182	HD22	ASN	12	-9.160	9.590	7.183	1.00	0.00	H
ATOM	183	C	ASN	12	-8.803	11.720	2.672	1.00	0.00	C
ATOM	184	O	ASN	12	-7.835	12.234	3.207	1.00	0.00	O
ATOM	185	N	ASP	13	-9.609	12.314	1.833	1.00	0.00	N
ATOM	186	H	ASP	13	-10.398	11.760	1.532	1.00	0.00	H
ATOM	187	CA	ASP	13	-9.429	13.668	1.262	1.00	0.00	C
ATOM	188	HA	ASP	13	-8.397	13.675	0.913	1.00	0.00	H
ATOM	189	CB	ASP	13	-10.346	13.995	0.079	1.00	0.00	C
ATOM	190	HB2	ASP	13	-10.270	13.164	-0.623	1.00	0.00	H
ATOM	191	HB3	ASP	13	-11.388	14.041	0.400	1.00	0.00	H
ATOM	192	CG	ASP	13	-9.826	15.258	-0.604	1.00	0.00	C
ATOM	193	OD1	ASP	13	-10.339	16.366	-0.355	1.00	0.00	O
ATOM	194	OD2	ASP	13	-8.792	15.001	-1.309	1.00	0.00	O
ATOM	195	C	ASP	13	-9.562	14.768	2.317	1.00	0.00	C
ATOM	196	O	ASP	13	-8.980	15.817	2.143	1.00	0.00	O
ATOM	197	N	TYR	14	-10.230	14.457	3.399	1.00	0.00	N
ATOM	198	H	TYR	14	-10.714	13.573	3.465	1.00	0.00	H

ATOM	199	CA	TYR	14	-10.514	15.346	4.461	1.00	0.00	C
ATOM	200	HA	TYR	14	-10.476	16.365	4.079	1.00	0.00	H
ATOM	201	CB	TYR	14	-12.001	15.124	4.860	1.00	0.00	C
ATOM	202	HB2	TYR	14	-12.266	15.830	5.646	1.00	0.00	H
ATOM	203	HB3	TYR	14	-12.542	15.280	3.926	1.00	0.00	H
ATOM	204	CG	TYR	14	-12.203	13.691	5.335	1.00	0.00	C
ATOM	205	CD1	TYR	14	-12.846	12.721	4.504	1.00	0.00	C
ATOM	206	HD1	TYR	14	-13.204	13.068	3.546	1.00	0.00	H
ATOM	207	CE1	TYR	14	-13.110	11.420	4.824	1.00	0.00	C
ATOM	208	HE1	TYR	14	-13.561	10.795	4.067	1.00	0.00	H
ATOM	209	CZ	TYR	14	-12.711	11.059	6.113	1.00	0.00	C
ATOM	210	OH	TYR	14	-12.903	9.710	6.381	1.00	0.00	O
ATOM	211	HH	TYR	14	-13.507	9.366	5.718	1.00	0.00	H
ATOM	212	CE2	TYR	14	-12.105	11.939	7.012	1.00	0.00	C
ATOM	213	HE2	TYR	14	-11.664	11.493	7.892	1.00	0.00	H
ATOM	214	CD2	TYR	14	-11.920	13.319	6.621	1.00	0.00	C
ATOM	215	HD2	TYR	14	-11.558	14.022	7.357	1.00	0.00	H
ATOM	216	C	TYR	14	-9.479	15.386	5.610	1.00	0.00	C
ATOM	217	O	TYR	14	-9.661	16.194	6.495	1.00	0.00	O
ATOM	218	N	GLU	15	-8.486	14.575	5.420	1.00	0.00	N
ATOM	219	H	GLU	15	-8.613	14.030	4.580	1.00	0.00	H
ATOM	220	CA	GLU	15	-7.208	14.504	6.127	1.00	0.00	C
ATOM	221	HA	GLU	15	-7.188	15.179	6.983	1.00	0.00	H
ATOM	222	CB	GLU	15	-6.953	13.089	6.715	1.00	0.00	C
ATOM	223	HB2	GLU	15	-7.139	12.306	5.980	1.00	0.00	H
ATOM	224	HB3	GLU	15	-5.923	13.007	7.064	1.00	0.00	H
ATOM	225	CG	GLU	15	-7.785	13.017	8.040	1.00	0.00	C
ATOM	226	HG2	GLU	15	-7.337	13.648	8.807	1.00	0.00	H
ATOM	227	HG3	GLU	15	-8.833	13.292	7.929	1.00	0.00	H
ATOM	228	CD	GLU	15	-7.746	11.585	8.507	1.00	0.00	C
ATOM	229	OE1	GLU	15	-6.683	10.994	8.812	1.00	0.00	O
ATOM	230	OE2	GLU	15	-8.787	10.870	8.496	1.00	0.00	O
ATOM	231	C	GLU	15	-5.970	14.880	5.270	1.00	0.00	C
ATOM	232	O	GLU	15	-5.068	15.632	5.625	1.00	0.00	O
ATOM	233	N	ASP	16	-5.971	14.494	4.024	1.00	0.00	N
ATOM	234	H	ASP	16	-6.718	13.880	3.734	1.00	0.00	H
ATOM	235	CA	ASP	16	-4.942	14.919	2.994	1.00	0.00	C
ATOM	236	HA	ASP	16	-3.981	14.575	3.376	1.00	0.00	H
ATOM	237	CB	ASP	16	-5.281	14.066	1.718	1.00	0.00	C
ATOM	238	HB2	ASP	16	-5.520	13.030	1.953	1.00	0.00	H
ATOM	239	HB3	ASP	16	-6.198	14.474	1.292	1.00	0.00	H
ATOM	240	CG	ASP	16	-4.196	14.130	0.675	1.00	0.00	C
ATOM	241	OD1	ASP	16	-4.281	14.976	-0.297	1.00	0.00	O
ATOM	242	OD2	ASP	16	-3.275	13.324	0.733	1.00	0.00	O
ATOM	243	C	ASP	16	-5.005	16.459	2.721	1.00	0.00	C
ATOM	244	O	ASP	16	-3.989	17.037	2.363	1.00	0.00	O
ATOM	245	N	ARG	17	-6.181	17.022	2.798	1.00	0.00	N
ATOM	246	H	ARG	17	-6.943	16.447	3.129	1.00	0.00	H
ATOM	247	CA	ARG	17	-6.362	18.408	2.272	1.00	0.00	C
ATOM	248	HA	ARG	17	-6.001	18.484	1.247	1.00	0.00	H
ATOM	249	CB	ARG	17	-7.806	18.893	2.184	1.00	0.00	C
ATOM	250	HB2	ARG	17	-7.784	19.889	1.742	1.00	0.00	H
ATOM	251	HB3	ARG	17	-8.324	18.252	1.471	1.00	0.00	H
ATOM	252	CG	ARG	17	-8.570	18.840	3.496	1.00	0.00	C
ATOM	253	HG2	ARG	17	-8.458	17.898	4.033	1.00	0.00	H
ATOM	254	HG3	ARG	17	-8.244	19.698	4.085	1.00	0.00	H
ATOM	255	CD	ARG	17	-10.009	19.209	3.246	1.00	0.00	C
ATOM	256	HD2	ARG	17	-10.440	19.206	4.248	1.00	0.00	H
ATOM	257	HD3	ARG	17	-10.102	20.220	2.851	1.00	0.00	H
ATOM	258	NE	ARG	17	-10.660	18.350	2.273	1.00	0.00	N
ATOM	259	HE	ARG	17	-10.147	17.727	1.665	1.00	0.00	H
ATOM	260	CZ	ARG	17	-11.965	18.418	2.000	1.00	0.00	C
ATOM	261	NH1	ARG	17	-12.816	19.113	2.618	1.00	0.00	N

ATOM	262	HH11	ARG	17	-13.796	19.281	2.438	1.00	0.00	H
ATOM	263	HH12	ARG	17	-12.338	19.678	3.305	1.00	0.00	H
ATOM	264	NH2	ARG	17	-12.423	17.808	0.919	1.00	0.00	N
ATOM	265	HH21	ARG	17	-11.833	17.163	0.412	1.00	0.00	H
ATOM	266	HH22	ARG	17	-13.390	17.801	0.630	1.00	0.00	H
ATOM	267	C	ARG	17	-5.431	19.384	3.088	1.00	0.00	C
ATOM	268	O	ARG	17	-4.778	20.143	2.468	1.00	0.00	O
ATOM	269	N	TYR	18	-5.247	19.144	4.380	1.00	0.00	N
ATOM	270	H	TYR	18	-5.457	18.217	4.721	1.00	0.00	H
ATOM	271	CA	TYR	18	-4.487	20.036	5.248	1.00	0.00	C
ATOM	272	HA	TYR	18	-4.882	21.049	5.320	1.00	0.00	H
ATOM	273	CB	TYR	18	-4.693	19.521	6.684	1.00	0.00	C
ATOM	274	HB2	TYR	18	-4.158	18.582	6.822	1.00	0.00	H
ATOM	275	HB3	TYR	18	-4.320	20.299	7.350	1.00	0.00	H
ATOM	276	CG	TYR	18	-6.150	19.381	7.122	1.00	0.00	C
ATOM	277	CD1	TYR	18	-7.111	20.445	6.897	1.00	0.00	C
ATOM	278	HD1	TYR	18	-6.802	21.382	6.457	1.00	0.00	H
ATOM	279	CE1	TYR	18	-8.404	20.279	7.309	1.00	0.00	C
ATOM	280	HE1	TYR	18	-9.121	21.073	7.163	1.00	0.00	H
ATOM	281	CZ	TYR	18	-8.807	19.110	8.040	1.00	0.00	C
ATOM	282	OH	TYR	18	-10.127	18.994	8.400	1.00	0.00	O
ATOM	283	HH	TYR	18	-10.110	18.110	8.776	1.00	0.00	H
ATOM	284	CE2	TYR	18	-7.866	18.080	8.400	1.00	0.00	C
ATOM	285	HE2	TYR	18	-8.108	17.170	8.928	1.00	0.00	H
ATOM	286	CD2	TYR	18	-6.468	18.251	7.924	1.00	0.00	C
ATOM	287	HD2	TYR	18	-5.758	17.488	8.209	1.00	0.00	H
ATOM	288	C	TYR	18	-2.951	20.102	5.012	1.00	0.00	C
ATOM	289	O	TYR	18	-2.296	20.994	5.524	1.00	0.00	O
ATOM	290	N	TYR	19	-2.453	19.092	4.325	1.00	0.00	N
ATOM	291	H	TYR	19	-3.068	18.297	4.222	1.00	0.00	H
ATOM	292	CA	TYR	19	-1.043	19.011	3.949	1.00	0.00	C
ATOM	293	HA	TYR	19	-0.445	19.567	4.672	1.00	0.00	H
ATOM	294	CB	TYR	19	-0.587	17.512	4.006	1.00	0.00	C
ATOM	295	HB2	TYR	19	-1.182	16.932	3.302	1.00	0.00	H
ATOM	296	HB3	TYR	19	0.450	17.331	3.724	1.00	0.00	H
ATOM	297	CG	TYR	19	-0.774	16.806	5.359	1.00	0.00	C
ATOM	298	CD1	TYR	19	-2.001	16.212	5.725	1.00	0.00	C
ATOM	299	HD1	TYR	19	-2.767	16.205	4.963	1.00	0.00	H
ATOM	300	CE1	TYR	19	-2.132	15.542	6.952	1.00	0.00	C
ATOM	301	HE1	TYR	19	-3.090	15.145	7.254	1.00	0.00	H
ATOM	302	CZ	TYR	19	-1.066	15.628	7.894	1.00	0.00	C
ATOM	303	OH	TYR	19	-1.325	15.061	9.087	1.00	0.00	O
ATOM	304	HH	TYR	19	-0.585	15.179	9.688	1.00	0.00	H
ATOM	305	CE2	TYR	19	0.169	16.287	7.663	1.00	0.00	C
ATOM	306	HE2	TYR	19	0.883	16.319	8.472	1.00	0.00	H
ATOM	307	CD2	TYR	19	0.246	16.977	6.400	1.00	0.00	C
ATOM	308	HD2	TYR	19	1.121	17.603	6.297	1.00	0.00	H
ATOM	309	C	TYR	19	-0.677	19.622	2.607	1.00	0.00	C
ATOM	310	O	TYR	19	0.347	20.321	2.498	1.00	0.00	O
ATOM	311	N	ARG	20	-1.505	19.384	1.548	1.00	0.00	N
ATOM	312	H	ARG	20	-2.255	18.748	1.775	1.00	0.00	H
ATOM	313	CA	ARG	20	-1.176	19.742	0.191	1.00	0.00	C
ATOM	314	HA	ARG	20	-0.138	19.447	0.044	1.00	0.00	H
ATOM	315	CB	ARG	20	-2.018	18.896	-0.752	1.00	0.00	C
ATOM	316	HB2	ARG	20	-1.618	19.099	-1.745	1.00	0.00	H
ATOM	317	HB3	ARG	20	-1.694	17.871	-0.579	1.00	0.00	H
ATOM	318	CG	ARG	20	-3.535	19.196	-0.618	1.00	0.00	C
ATOM	319	HG2	ARG	20	-3.938	18.735	0.283	1.00	0.00	H
ATOM	320	HG3	ARG	20	-3.811	20.249	-0.647	1.00	0.00	H
ATOM	321	CD	ARG	20	-4.299	18.451	-1.737	1.00	0.00	C
ATOM	322	HD2	ARG	20	-3.948	18.831	-2.696	1.00	0.00	H
ATOM	323	HD3	ARG	20	-4.076	17.389	-1.642	1.00	0.00	H
ATOM	324	NE	ARG	20	-5.799	18.575	-1.658	1.00	0.00	N

ATOM	325	HE	ARG	20	-6.097	19.457	-2.048	1.00	0.00	H
ATOM	326	CZ	ARG	20	-6.753	17.629	-1.394	1.00	0.00	C
ATOM	327	NH1	ARG	20	-6.502	16.495	-0.871	1.00	0.00	N
ATOM	328	HH11	ARG	20	-5.565	16.156	-0.711	1.00	0.00	H
ATOM	329	HH12	ARG	20	-7.263	15.830	-0.862	1.00	0.00	H
ATOM	330	NH2	ARG	20	-7.920	17.758	-1.799	1.00	0.00	N
ATOM	331	HH21	ARG	20	-8.285	18.664	-2.058	1.00	0.00	H
ATOM	332	HH22	ARG	20	-8.621	17.078	-1.543	1.00	0.00	H
ATOM	333	C	ARG	20	-1.278	21.294	-0.086	1.00	0.00	C
ATOM	334	O	ARG	20	-0.726	21.706	-1.103	1.00	0.00	O
ATOM	335	N	GLU	21	-1.825	22.033	0.822	1.00	0.00	N
ATOM	336	H	GLU	21	-2.220	21.617	1.653	1.00	0.00	H
ATOM	337	CA	GLU	21	-1.844	23.506	0.762	1.00	0.00	C
ATOM	338	HA	GLU	21	-1.908	23.695	-0.309	1.00	0.00	H
ATOM	339	CB	GLU	21	-3.115	24.090	1.349	1.00	0.00	C
ATOM	340	HB2	GLU	21	-3.011	25.175	1.343	1.00	0.00	H
ATOM	341	HB3	GLU	21	-3.970	23.828	0.725	1.00	0.00	H
ATOM	342	CG	GLU	21	-3.218	23.548	2.816	1.00	0.00	C
ATOM	343	HG2	GLU	21	-3.768	22.608	2.750	1.00	0.00	H
ATOM	344	HG3	GLU	21	-2.292	23.407	3.373	1.00	0.00	H
ATOM	345	CD	GLU	21	-4.067	24.481	3.755	1.00	0.00	C
ATOM	346	OE1	GLU	21	-3.543	25.602	4.120	1.00	0.00	O
ATOM	347	OE2	GLU	21	-5.242	24.088	4.043	1.00	0.00	O
ATOM	348	C	GLU	21	-0.564	24.221	1.325	1.00	0.00	C
ATOM	349	O	GLU	21	-0.590	25.457	1.335	1.00	0.00	O
ATOM	350	N	ASN	22	0.458	23.456	1.761	1.00	0.00	N
ATOM	351	H	ASN	22	0.360	22.455	1.839	1.00	0.00	H
ATOM	352	CA	ASN	22	1.786	23.923	2.269	1.00	0.00	C
ATOM	353	HA	ASN	22	1.872	25.002	2.398	1.00	0.00	H
ATOM	354	CB	ASN	22	1.924	23.236	3.677	1.00	0.00	C
ATOM	355	HB2	ASN	22	1.580	22.205	3.603	1.00	0.00	H
ATOM	356	HB3	ASN	22	2.973	23.279	3.968	1.00	0.00	H
ATOM	357	CG	ASN	22	1.077	23.939	4.750	1.00	0.00	C
ATOM	358	OD1	ASN	22	1.370	25.050	5.032	1.00	0.00	O
ATOM	359	ND2	ASN	22	0.143	23.363	5.455	1.00	0.00	N
ATOM	360	HD21	ASN	22	-0.450	23.884	6.086	1.00	0.00	H
ATOM	361	HD22	ASN	22	-0.251	22.466	5.208	1.00	0.00	H
ATOM	362	C	ASN	22	2.971	23.679	1.326	1.00	0.00	C
ATOM	363	O	ASN	22	4.081	23.954	1.727	1.00	0.00	O
ATOM	364	N	MET	23	2.717	23.113	0.120	1.00	0.00	N
ATOM	365	H	MET	23	1.834	22.650	-0.045	1.00	0.00	H
ATOM	366	CA	MET	23	3.705	22.925	-0.897	1.00	0.00	C
ATOM	367	HA	MET	23	4.516	22.507	-0.302	1.00	0.00	H
ATOM	368	CB	MET	23	3.354	21.837	-2.006	1.00	0.00	C
ATOM	369	HB2	MET	23	2.386	22.002	-2.480	1.00	0.00	H
ATOM	370	HB3	MET	23	4.092	21.897	-2.804	1.00	0.00	H
ATOM	371	CG	MET	23	3.421	20.522	-1.206	1.00	0.00	C
ATOM	372	HG2	MET	23	4.449	20.382	-0.874	1.00	0.00	H
ATOM	373	HG3	MET	23	2.755	20.605	-0.348	1.00	0.00	H
ATOM	374	SD	MET	23	2.994	19.016	-2.203	1.00	0.00	S
ATOM	375	CE	MET	23	3.647	17.812	-0.992	1.00	0.00	C
ATOM	376	HE1	MET	23	3.488	16.869	-1.516	1.00	0.00	H
ATOM	377	HE2	MET	23	4.716	17.831	-0.782	1.00	0.00	H
ATOM	378	HE3	MET	23	3.106	17.774	-0.045	1.00	0.00	H
ATOM	379	C	MET	23	4.040	24.291	-1.601	1.00	0.00	C
ATOM	380	O	MET	23	5.041	24.493	-2.308	1.00	0.00	O
ATOM	381	N	TYR	24	3.234	25.368	-1.324	1.00	0.00	N
ATOM	382	H	TYR	24	2.492	25.231	-0.653	1.00	0.00	H
ATOM	383	CA	TYR	24	3.202	26.652	-2.014	1.00	0.00	C
ATOM	384	HA	TYR	24	4.009	26.555	-2.740	1.00	0.00	H
ATOM	385	CB	TYR	24	2.080	26.813	-2.978	1.00	0.00	C
ATOM	386	HB2	TYR	24	1.213	26.518	-2.386	1.00	0.00	H
ATOM	387	HB3	TYR	24	1.966	27.802	-3.421	1.00	0.00	H

ATOM	388	CG	TYR	24	2.295	25.850	-4.137	1.00	0.00	C
ATOM	389	CD1	TYR	24	3.114	26.173	-5.238	1.00	0.00	C
ATOM	390	HD1	TYR	24	3.567	27.139	-5.407	1.00	0.00	H
ATOM	391	CE1	TYR	24	3.199	25.399	-6.380	1.00	0.00	C
ATOM	392	HE1	TYR	24	3.629	25.781	-7.294	1.00	0.00	H
ATOM	393	CZ	TYR	24	2.500	24.207	-6.419	1.00	0.00	C
ATOM	394	OH	TYR	24	2.539	23.530	-7.614	1.00	0.00	O
ATOM	395	HH	TYR	24	3.031	24.022	-8.276	1.00	0.00	H
ATOM	396	CE2	TYR	24	1.727	23.801	-5.328	1.00	0.00	C
ATOM	397	HE2	TYR	24	1.257	22.828	-5.358	1.00	0.00	H
ATOM	398	CD2	TYR	24	1.604	24.625	-4.182	1.00	0.00	C
ATOM	399	HD2	TYR	24	0.826	24.394	-3.468	1.00	0.00	H
ATOM	400	C	TYR	24	3.531	27.883	-1.213	1.00	0.00	C
ATOM	401	O	TYR	24	3.831	28.909	-1.809	1.00	0.00	O
ATOM	402	N	ARG	25	3.647	27.866	0.114	1.00	0.00	N
ATOM	403	H	ARG	25	3.658	26.929	0.489	1.00	0.00	H
ATOM	404	CA	ARG	25	3.736	29.079	1.015	1.00	0.00	C
ATOM	405	HA	ARG	25	3.064	29.752	0.481	1.00	0.00	H
ATOM	406	CB	ARG	25	3.356	28.827	2.526	1.00	0.00	C
ATOM	407	HB2	ARG	25	3.646	27.790	2.699	1.00	0.00	H
ATOM	408	HB3	ARG	25	3.949	29.476	3.170	1.00	0.00	H
ATOM	409	CG	ARG	25	1.853	29.051	2.862	1.00	0.00	C
ATOM	410	HG2	ARG	25	1.650	30.121	2.918	1.00	0.00	H
ATOM	411	HG3	ARG	25	1.232	28.660	2.057	1.00	0.00	H
ATOM	412	CD	ARG	25	1.550	28.462	4.176	1.00	0.00	C
ATOM	413	HD2	ARG	25	1.677	27.385	4.291	1.00	0.00	H
ATOM	414	HD3	ARG	25	2.215	28.915	4.911	1.00	0.00	H
ATOM	415	NE	ARG	25	0.107	28.735	4.462	1.00	0.00	N
ATOM	416	HE	ARG	25	-0.136	29.711	4.373	1.00	0.00	H
ATOM	417	CZ	ARG	25	-0.871	27.888	4.673	1.00	0.00	C
ATOM	418	NH1	ARG	25	-0.801	26.593	4.812	1.00	0.00	N
ATOM	419	HH11	ARG	25	0.023	26.010	4.835	1.00	0.00	H
ATOM	420	HH12	ARG	25	-1.702	26.148	4.713	1.00	0.00	H
ATOM	421	NH2	ARG	25	-2.106	28.336	4.951	1.00	0.00	N
ATOM	422	HH21	ARG	25	-2.333	29.293	4.723	1.00	0.00	H
ATOM	423	HH22	ARG	25	-2.816	27.717	5.313	1.00	0.00	H
ATOM	424	C	ARG	25	5.100	29.657	0.833	1.00	0.00	C
ATOM	425	O	ARG	25	5.196	30.848	1.110	1.00	0.00	O
ATOM	426	N	TYR	26	6.058	28.909	0.388	1.00	0.00	N
ATOM	427	H	TYR	26	5.866	28.005	-0.020	1.00	0.00	H
ATOM	428	CA	TYR	26	7.418	29.380	0.304	1.00	0.00	C
ATOM	429	HA	TYR	26	7.516	30.454	0.460	1.00	0.00	H
ATOM	430	CB	TYR	26	8.200	28.768	1.483	1.00	0.00	C
ATOM	431	HB2	TYR	26	7.798	29.127	2.430	1.00	0.00	H
ATOM	432	HB3	TYR	26	8.068	27.690	1.564	1.00	0.00	H
ATOM	433	CG	TYR	26	9.685	29.072	1.514	1.00	0.00	C
ATOM	434	CD1	TYR	26	10.660	28.340	0.743	1.00	0.00	C
ATOM	435	HD1	TYR	26	10.392	27.632	-0.029	1.00	0.00	H
ATOM	436	CE1	TYR	26	12.023	28.671	0.767	1.00	0.00	C
ATOM	437	HE1	TYR	26	12.725	28.054	0.227	1.00	0.00	H
ATOM	438	CZ	TYR	26	12.444	29.770	1.572	1.00	0.00	C
ATOM	439	OH	TYR	26	13.746	30.155	1.532	1.00	0.00	O
ATOM	440	HH	TYR	26	14.205	29.510	0.990	1.00	0.00	H
ATOM	441	CE2	TYR	26	11.486	30.607	2.222	1.00	0.00	C
ATOM	442	HE2	TYR	26	11.798	31.475	2.783	1.00	0.00	H
ATOM	443	CD2	TYR	26	10.148	30.213	2.237	1.00	0.00	C
ATOM	444	HD2	TYR	26	9.447	30.806	2.805	1.00	0.00	H
ATOM	445	C	TYR	26	8.022	29.092	-1.106	1.00	0.00	C
ATOM	446	O	TYR	26	7.801	27.954	-1.655	1.00	0.00	O
ATOM	447	N	NME	27	8.824	30.052	-1.553	1.00	0.00	N
ATOM	448	H	NME	27	8.907	30.959	-1.115	1.00	0.00	H
ATOM	449	CH3	NME	27	9.874	29.712	-2.485	1.00	0.00	C
ATOM	450	HH31	NME	27	10.293	30.603	-2.953	1.00	0.00	H

```
ATOM    451 HH32 NME    27      10.756  29.262  -2.027  1.00  0.00           H
ATOM    452 HH33 NME    27       9.485  28.940  -3.148  1.00  0.00           H
TER     453      NME    27
END
```

Cluster 3, Figure 1B:

```
ATOM      1 HH31 ACE     1       1.524  10.046  -6.899  1.00  0.00           H
ATOM      2  CH3 ACE     1       1.652  10.688  -6.028  1.00  0.00           C
ATOM      3 HH32 ACE     1       0.631  11.046  -5.897  1.00  0.00           H
ATOM      4 HH33 ACE     1       2.419  11.411  -6.311  1.00  0.00           H
ATOM      5  C   ACE     1       2.105  10.045  -4.818  1.00  0.00           C
ATOM      6  O   ACE     1       2.537  10.654  -3.836  1.00  0.00           O
ATOM      7  N   VAL     2       1.922   8.715  -4.764  1.00  0.00           N
ATOM      8  H   VAL     2       1.449   8.330  -5.569  1.00  0.00           H
ATOM      9  CA  VAL     2       2.052   7.865  -3.589  1.00  0.00           C
ATOM     10  HA  VAL     2       1.316   8.208  -2.861  1.00  0.00           H
ATOM     11  CB  VAL     2       1.593   6.469  -4.025  1.00  0.00           C
ATOM     12  HB  VAL     2       1.512   5.842  -3.137  1.00  0.00           H
ATOM     13  CG1 VAL     2       0.188   6.497  -4.715  1.00  0.00           C
ATOM     14 HG11 VAL     2      -0.593   6.928  -4.090  1.00  0.00           H
ATOM     15 HG12 VAL     2       0.240   7.099  -5.623  1.00  0.00           H
ATOM     16 HG13 VAL     2      -0.048   5.435  -4.787  1.00  0.00           H
ATOM     17  CG2 VAL     2       2.415   5.630  -4.930  1.00  0.00           C
ATOM     18 HG21 VAL     2       1.944   4.649  -4.982  1.00  0.00           H
ATOM     19 HG22 VAL     2       2.621   6.060  -5.909  1.00  0.00           H
ATOM     20 HG23 VAL     2       3.340   5.313  -4.447  1.00  0.00           H
ATOM     21  C   VAL     2       3.447   7.824  -2.836  1.00  0.00           C
ATOM     22  O   VAL     2       3.552   7.195  -1.801  1.00  0.00           O
ATOM     23  N   MET     3       4.455   8.522  -3.418  1.00  0.00           N
ATOM     24  H   MET     3       4.276   8.898  -4.338  1.00  0.00           H
ATOM     25  CA  MET     3       5.805   8.607  -2.907  1.00  0.00           C
ATOM     26  HA  MET     3       5.919   8.049  -1.978  1.00  0.00           H
ATOM     27  CB  MET     3       6.821   8.008  -3.835  1.00  0.00           C
ATOM     28  HB2 MET     3       6.720   8.508  -4.798  1.00  0.00           H
ATOM     29  HB3 MET     3       7.812   8.247  -3.448  1.00  0.00           H
ATOM     30  CG  MET     3       6.711   6.491  -3.994  1.00  0.00           C
ATOM     31  HG2 MET     3       5.637   6.365  -4.122  1.00  0.00           H
ATOM     32  HG3 MET     3       7.217   6.269  -4.934  1.00  0.00           H
ATOM     33  SD  MET     3       7.412   5.649  -2.512  1.00  0.00           S
ATOM     34  CE  MET     3       7.557   3.987  -3.227  1.00  0.00           C
ATOM     35  HE1 MET     3       7.790   3.276  -2.434  1.00  0.00           H
ATOM     36  HE2 MET     3       6.548   3.712  -3.536  1.00  0.00           H
ATOM     37  HE3 MET     3       8.342   3.828  -3.966  1.00  0.00           H
ATOM     38  C   MET     3       6.147  10.043  -2.512  1.00  0.00           C
ATOM     39  O   MET     3       7.288  10.321  -2.021  1.00  0.00           O
ATOM     40  N   SER     4       5.178  11.003  -2.654  1.00  0.00           N
ATOM     41  H   SER     4       4.360  10.679  -3.151  1.00  0.00           H
ATOM     42  CA  SER     4       5.500  12.466  -2.468  1.00  0.00           C
ATOM     43  HA  SER     4       6.442  12.794  -2.908  1.00  0.00           H
ATOM     44  CB  SER     4       4.479  13.303  -3.206  1.00  0.00           C
ATOM     45  HB2 SER     4       4.734  14.351  -3.051  1.00  0.00           H
ATOM     46  HB3 SER     4       4.483  13.126  -4.281  1.00  0.00           H
ATOM     47  OG  SER     4       3.128  13.162  -2.648  1.00  0.00           O
ATOM     48  HG  SER     4       2.741  12.346  -2.972  1.00  0.00           H
ATOM     49  C   SER     4       5.518  12.850  -0.918  1.00  0.00           C
ATOM     50  O   SER     4       6.222  13.763  -0.551  1.00  0.00           O
ATOM     51  N   ARG     5       4.890  12.067  -0.063  1.00  0.00           N
ATOM     52  H   ARG     5       4.434  11.276  -0.495  1.00  0.00           H
ATOM     53  CA  ARG     5       4.573  12.395   1.322  1.00  0.00           C
ATOM     54  HA  ARG     5       5.385  13.021   1.690  1.00  0.00           H
```

ATOM	55	CB	ARG	5	3.272	13.239	1.391	1.00	0.00	C
ATOM	56	HB2	ARG	5	3.115	13.382	2.460	1.00	0.00	H
ATOM	57	HB3	ARG	5	3.556	14.103	0.791	1.00	0.00	H
ATOM	58	CG	ARG	5	2.050	12.567	0.804	1.00	0.00	C
ATOM	59	HG2	ARG	5	2.241	11.898	-0.035	1.00	0.00	H
ATOM	60	HG3	ARG	5	1.525	11.990	1.566	1.00	0.00	H
ATOM	61	CD	ARG	5	0.934	13.440	0.302	1.00	0.00	C
ATOM	62	HD2	ARG	5	0.157	12.865	-0.202	1.00	0.00	H
ATOM	63	HD3	ARG	5	0.575	14.019	1.153	1.00	0.00	H
ATOM	64	NE	ARG	5	1.355	14.419	-0.771	1.00	0.00	N
ATOM	65	HE	ARG	5	2.158	14.119	-1.304	1.00	0.00	H
ATOM	66	CZ	ARG	5	0.686	15.413	-1.300	1.00	0.00	C
ATOM	67	NH1	ARG	5	1.002	16.032	-2.415	1.00	0.00	N
ATOM	68	HH11	ARG	5	1.738	15.670	-3.004	1.00	0.00	H
ATOM	69	HH12	ARG	5	0.423	16.740	-2.843	1.00	0.00	H
ATOM	70	NH2	ARG	5	-0.447	15.739	-0.827	1.00	0.00	N
ATOM	71	HH21	ARG	5	-0.925	16.471	-1.334	1.00	0.00	H
ATOM	72	HH22	ARG	5	-0.846	15.272	-0.025	1.00	0.00	H
ATOM	73	C	ARG	5	4.424	11.115	2.248	1.00	0.00	C
ATOM	74	O	ARG	5	4.110	10.075	1.733	1.00	0.00	O
ATOM	75	N	PRO	6	4.745	11.183	3.563	1.00	0.00	N
ATOM	76	CD	PRO	6	5.428	12.303	4.262	1.00	0.00	C
ATOM	77	HD2	PRO	6	5.153	13.246	3.789	1.00	0.00	H
ATOM	78	HD3	PRO	6	6.501	12.117	4.239	1.00	0.00	H
ATOM	79	CG	PRO	6	4.943	12.232	5.724	1.00	0.00	C
ATOM	80	HG2	PRO	6	3.891	12.518	5.705	1.00	0.00	H
ATOM	81	HG3	PRO	6	5.568	12.749	6.453	1.00	0.00	H
ATOM	82	CB	PRO	6	4.995	10.729	5.921	1.00	0.00	C
ATOM	83	HB2	PRO	6	4.462	10.373	6.803	1.00	0.00	H
ATOM	84	HB3	PRO	6	5.998	10.313	6.016	1.00	0.00	H
ATOM	85	CA	PRO	6	4.467	10.194	4.585	1.00	0.00	C
ATOM	86	HA	PRO	6	5.071	9.329	4.311	1.00	0.00	H
ATOM	87	C	PRO	6	2.972	9.802	4.720	1.00	0.00	C
ATOM	88	O	PRO	6	2.086	10.437	4.167	1.00	0.00	O
ATOM	89	N	LEU	7	2.719	8.678	5.410	1.00	0.00	N
ATOM	90	H	LEU	7	3.546	8.233	5.783	1.00	0.00	H
ATOM	91	CA	LEU	7	1.427	8.028	5.550	1.00	0.00	C
ATOM	92	HA	LEU	7	1.064	7.915	4.529	1.00	0.00	H
ATOM	93	CB	LEU	7	1.820	6.560	6.056	1.00	0.00	C
ATOM	94	HB2	LEU	7	2.629	6.137	5.459	1.00	0.00	H
ATOM	95	HB3	LEU	7	2.147	6.641	7.093	1.00	0.00	H
ATOM	96	CG	LEU	7	0.572	5.550	5.981	1.00	0.00	C
ATOM	97	HG	LEU	7	-0.334	6.031	6.350	1.00	0.00	H
ATOM	98	CD1	LEU	7	0.225	5.117	4.625	1.00	0.00	C
ATOM	99	HD11	LEU	7	-0.451	4.267	4.713	1.00	0.00	H
ATOM	100	HD12	LEU	7	-0.229	5.962	4.108	1.00	0.00	H
ATOM	101	HD13	LEU	7	1.135	4.866	4.080	1.00	0.00	H
ATOM	102	CD2	LEU	7	0.911	4.378	6.932	1.00	0.00	C
ATOM	103	HD21	LEU	7	1.268	4.749	7.893	1.00	0.00	H
ATOM	104	HD22	LEU	7	0.049	3.781	7.230	1.00	0.00	H
ATOM	105	HD23	LEU	7	1.711	3.757	6.530	1.00	0.00	H
ATOM	106	C	LEU	7	0.518	8.886	6.443	1.00	0.00	C
ATOM	107	O	LEU	7	-0.680	8.916	6.318	1.00	0.00	O
ATOM	108	N	ILE	8	1.130	9.615	7.411	1.00	0.00	N
ATOM	109	H	ILE	8	2.130	9.515	7.517	1.00	0.00	H
ATOM	110	CA	ILE	8	0.527	10.569	8.311	1.00	0.00	C
ATOM	111	HA	ILE	8	-0.337	10.026	8.694	1.00	0.00	H
ATOM	112	CB	ILE	8	1.578	10.787	9.453	1.00	0.00	C
ATOM	113	HB	ILE	8	1.936	9.805	9.766	1.00	0.00	H
ATOM	114	CG2	ILE	8	2.826	11.621	9.133	1.00	0.00	C
ATOM	115	HG21	ILE	8	2.608	12.675	8.965	1.00	0.00	H
ATOM	116	HG22	ILE	8	3.549	11.462	9.933	1.00	0.00	H
ATOM	117	HG23	ILE	8	3.197	11.264	8.173	1.00	0.00	H

```
ATOM    118  CG1 ILE     8       0.920  11.254  10.769  1.00  0.00           C
ATOM    119 HG12 ILE     8       0.785  12.332  10.861  1.00  0.00           H
ATOM    120 HG13 ILE     8      -0.064  10.799  10.885  1.00  0.00           H
ATOM    121  CD1 ILE     8       1.671  10.683  11.977  1.00  0.00           C
ATOM    122 HD11 ILE     8       1.257  11.070  12.907  1.00  0.00           H
ATOM    123 HD12 ILE     8       1.659   9.593  11.960  1.00  0.00           H
ATOM    124 HD13 ILE     8       2.720  10.979  11.949  1.00  0.00           H
ATOM    125  C   ILE     8       0.077  11.864   7.608  1.00  0.00           C
ATOM    126  O   ILE     8      -0.830  12.536   8.095  1.00  0.00           O
ATOM    127  N   HID     9       0.654  12.167   6.446  1.00  0.00           N
ATOM    128  H   HID     9       1.443  11.606   6.161  1.00  0.00           H
ATOM    129  CA  HID     9       0.269  13.257   5.644  1.00  0.00           C
ATOM    130  HA  HID     9      -0.177  13.936   6.372  1.00  0.00           H
ATOM    131  CB  HID     9       1.450  13.882   4.919  1.00  0.00           C
ATOM    132  HB2 HID     9       2.251  14.012   5.647  1.00  0.00           H
ATOM    133  HB3 HID     9       1.795  13.088   4.258  1.00  0.00           H
ATOM    134  CG  HID     9       1.133  15.202   4.206  1.00  0.00           C
ATOM    135  ND1 HID     9      -0.082  15.695   3.739  1.00  0.00           N
ATOM    136  HD1 HID     9      -0.968  15.249   3.932  1.00  0.00           H
ATOM    137  CE1 HID     9       0.247  16.859   3.052  1.00  0.00           C
ATOM    138  HE1 HID     9      -0.435  17.450   2.458  1.00  0.00           H
ATOM    139  NE2 HID     9       1.570  17.102   3.041  1.00  0.00           N
ATOM    140  CD2 HID     9       2.168  16.074   3.829  1.00  0.00           C
ATOM    141  HD2 HID     9       3.194  15.859   4.090  1.00  0.00           H
ATOM    142  C   HID     9      -0.794  12.828   4.601  1.00  0.00           C
ATOM    143  O   HID     9      -1.870  13.482   4.500  1.00  0.00           O
ATOM    144  N   PHE    10      -0.619  11.667   3.941  1.00  0.00           N
ATOM    145  H   PHE    10       0.239  11.146   4.052  1.00  0.00           H
ATOM    146  CA  PHE    10      -1.472  11.176   2.895  1.00  0.00           C
ATOM    147  HA  PHE    10      -1.608  11.948   2.137  1.00  0.00           H
ATOM    148  CB  PHE    10      -0.826   9.939   2.283  1.00  0.00           C
ATOM    149  HB2 PHE    10       0.238  10.022   2.057  1.00  0.00           H
ATOM    150  HB3 PHE    10      -0.903   9.186   3.068  1.00  0.00           H
ATOM    151  CG  PHE    10      -1.548   9.356   1.074  1.00  0.00           C
ATOM    152  CD1 PHE    10      -1.532   9.924  -0.196  1.00  0.00           C
ATOM    153  HD1 PHE    10      -0.989  10.855  -0.275  1.00  0.00           H
ATOM    154  CE1 PHE    10      -1.994   9.286  -1.391  1.00  0.00           C
ATOM    155  HE1 PHE    10      -1.762   9.612  -2.394  1.00  0.00           H
ATOM    156  CZ  PHE    10      -2.640   7.983  -1.173  1.00  0.00           C
ATOM    157  HZ  PHE    10      -2.973   7.404  -2.022  1.00  0.00           H
ATOM    158  CE2 PHE    10      -2.718   7.453   0.124  1.00  0.00           C
ATOM    159  HE2 PHE    10      -3.178   6.491   0.289  1.00  0.00           H
ATOM    160  CD2 PHE    10      -2.100   8.098   1.218  1.00  0.00           C
ATOM    161  HD2 PHE    10      -1.984   7.566   2.150  1.00  0.00           H
ATOM    162  C   PHE    10      -2.921  10.829   3.311  1.00  0.00           C
ATOM    163  O   PHE    10      -3.858  10.965   2.511  1.00  0.00           O
ATOM    164  N   GLY    11      -3.044  10.285   4.521  1.00  0.00           N
ATOM    165  H   GLY    11      -2.208  10.265   5.088  1.00  0.00           H
ATOM    166  CA  GLY    11      -4.309  10.146   5.114  1.00  0.00           C
ATOM    167  HA2 GLY    11      -5.011   9.652   4.443  1.00  0.00           H
ATOM    168  HA3 GLY    11      -4.225   9.365   5.869  1.00  0.00           H
ATOM    169  C   GLY    11      -4.976  11.339   5.720  1.00  0.00           C
ATOM    170  O   GLY    11      -6.143  11.216   6.119  1.00  0.00           O
ATOM    171  N   ASN    12      -4.318  12.533   5.682  1.00  0.00           N
ATOM    172  H   ASN    12      -3.355  12.624   5.392  1.00  0.00           H
ATOM    173  CA  ASN    12      -4.926  13.752   6.259  1.00  0.00           C
ATOM    174  HA  ASN    12      -5.801  13.480   6.851  1.00  0.00           H
ATOM    175  CB  ASN    12      -3.811  14.298   7.230  1.00  0.00           C
ATOM    176  HB2 ASN    12      -3.510  13.510   7.920  1.00  0.00           H
ATOM    177  HB3 ASN    12      -2.919  14.566   6.663  1.00  0.00           H
ATOM    178  CG  ASN    12      -4.411  15.505   7.959  1.00  0.00           C
ATOM    179  OD1 ASN    12      -4.229  16.664   7.651  1.00  0.00           O
ATOM    180  ND2 ASN    12      -5.252  15.166   8.907  1.00  0.00           N
```

ATOM	181	HD21	ASN	12	-5.844	15.847	9.361	1.00	0.00	H
ATOM	182	HD22	ASN	12	-5.252	14.240	9.313	1.00	0.00	H
ATOM	183	C	ASN	12	-5.311	14.805	5.188	1.00	0.00	C
ATOM	184	O	ASN	12	-6.375	15.368	5.278	1.00	0.00	O
ATOM	185	N	ASP	13	-4.461	14.946	4.140	1.00	0.00	N
ATOM	186	H	ASP	13	-3.629	14.386	4.255	1.00	0.00	H
ATOM	187	CA	ASP	13	-4.452	15.940	3.027	1.00	0.00	C
ATOM	188	HA	ASP	13	-5.481	16.238	2.825	1.00	0.00	H
ATOM	189	CB	ASP	13	-3.685	17.193	3.529	1.00	0.00	C
ATOM	190	HB2	ASP	13	-4.191	17.598	4.406	1.00	0.00	H
ATOM	191	HB3	ASP	13	-2.722	16.897	3.945	1.00	0.00	H
ATOM	192	CG	ASP	13	-3.594	18.263	2.455	1.00	0.00	C
ATOM	193	OD1	ASP	13	-4.685	18.817	2.219	1.00	0.00	O
ATOM	194	OD2	ASP	13	-2.560	18.676	1.873	1.00	0.00	O
ATOM	195	C	ASP	13	-3.828	15.368	1.740	1.00	0.00	C
ATOM	196	O	ASP	13	-2.714	14.871	1.686	1.00	0.00	O
ATOM	197	N	TYR	14	-4.635	15.480	0.633	1.00	0.00	N
ATOM	198	H	TYR	14	-5.522	15.935	0.798	1.00	0.00	H
ATOM	199	CA	TYR	14	-4.329	15.008	-0.736	1.00	0.00	C
ATOM	200	HA	TYR	14	-3.264	14.788	-0.815	1.00	0.00	H
ATOM	201	CB	TYR	14	-5.102	13.613	-0.939	1.00	0.00	C
ATOM	202	HB2	TYR	14	-5.156	13.453	-2.016	1.00	0.00	H
ATOM	203	HB3	TYR	14	-4.542	12.770	-0.534	1.00	0.00	H
ATOM	204	CG	TYR	14	-6.504	13.585	-0.380	1.00	0.00	C
ATOM	205	CD1	TYR	14	-7.579	13.989	-1.225	1.00	0.00	C
ATOM	206	HD1	TYR	14	-7.332	14.191	-2.256	1.00	0.00	H
ATOM	207	CE1	TYR	14	-8.876	13.991	-0.793	1.00	0.00	C
ATOM	208	HE1	TYR	14	-9.641	14.252	-1.509	1.00	0.00	H
ATOM	209	CZ	TYR	14	-9.158	13.578	0.525	1.00	0.00	C
ATOM	210	OH	TYR	14	-10.423	13.453	1.028	1.00	0.00	O
ATOM	211	HH	TYR	14	-10.384	13.172	1.945	1.00	0.00	H
ATOM	212	CE2	TYR	14	-8.116	13.269	1.391	1.00	0.00	C
ATOM	213	HE2	TYR	14	-8.403	12.974	2.388	1.00	0.00	H
ATOM	214	CD2	TYR	14	-6.769	13.233	0.973	1.00	0.00	C
ATOM	215	HD2	TYR	14	-6.046	12.998	1.740	1.00	0.00	H
ATOM	216	C	TYR	14	-4.750	16.037	-1.709	1.00	0.00	C
ATOM	217	O	TYR	14	-5.517	16.928	-1.475	1.00	0.00	O
ATOM	218	N	GLU	15	-4.148	15.867	-2.943	1.00	0.00	N
ATOM	219	H	GLU	15	-3.603	15.047	-3.167	1.00	0.00	H
ATOM	220	CA	GLU	15	-4.292	16.927	-3.970	1.00	0.00	C
ATOM	221	HA	GLU	15	-4.083	17.906	-3.538	1.00	0.00	H
ATOM	222	CB	GLU	15	-3.335	16.780	-5.116	1.00	0.00	C
ATOM	223	HB2	GLU	15	-3.525	15.806	-5.568	1.00	0.00	H
ATOM	224	HB3	GLU	15	-3.585	17.491	-5.903	1.00	0.00	H
ATOM	225	CG	GLU	15	-1.891	16.794	-4.591	1.00	0.00	C
ATOM	226	HG2	GLU	15	-1.822	15.807	-4.132	1.00	0.00	H
ATOM	227	HG3	GLU	15	-1.123	16.683	-5.356	1.00	0.00	H
ATOM	228	CD	GLU	15	-1.616	17.956	-3.643	1.00	0.00	C
ATOM	229	OE1	GLU	15	-1.754	19.158	-4.066	1.00	0.00	O
ATOM	230	OE2	GLU	15	-1.415	17.645	-2.423	1.00	0.00	O
ATOM	231	C	GLU	15	-5.732	17.052	-4.525	1.00	0.00	C
ATOM	232	O	GLU	15	-6.059	18.068	-5.100	1.00	0.00	O
ATOM	233	N	ASP	16	-6.487	15.965	-4.453	1.00	0.00	N
ATOM	234	H	ASP	16	-6.054	15.158	-4.027	1.00	0.00	H
ATOM	235	CA	ASP	16	-7.785	15.885	-5.079	1.00	0.00	C
ATOM	236	HA	ASP	16	-7.696	16.507	-5.969	1.00	0.00	H
ATOM	237	CB	ASP	16	-7.988	14.532	-5.775	1.00	0.00	C
ATOM	238	HB2	ASP	16	-7.049	14.114	-6.136	1.00	0.00	H
ATOM	239	HB3	ASP	16	-8.220	13.818	-4.984	1.00	0.00	H
ATOM	240	CG	ASP	16	-8.944	14.472	-6.912	1.00	0.00	C
ATOM	241	OD1	ASP	16	-9.598	13.424	-6.980	1.00	0.00	O
ATOM	242	OD2	ASP	16	-8.959	15.298	-7.870	1.00	0.00	O
ATOM	243	C	ASP	16	-8.988	16.402	-4.334	1.00	0.00	C

ATOM	244	O	ASP	16	-10.155	16.207	-4.789	1.00	0.00	O
ATOM	245	N	ARG	17	-8.702	17.058	-3.188	1.00	0.00	N
ATOM	246	H	ARG	17	-7.751	16.984	-2.859	1.00	0.00	H
ATOM	247	CA	ARG	17	-9.768	17.672	-2.329	1.00	0.00	C
ATOM	248	HA	ARG	17	-10.489	16.895	-2.077	1.00	0.00	H
ATOM	249	CB	ARG	17	-9.073	18.332	-1.033	1.00	0.00	C
ATOM	250	HB2	ARG	17	-8.211	18.931	-1.326	1.00	0.00	H
ATOM	251	HB3	ARG	17	-9.817	18.907	-0.482	1.00	0.00	H
ATOM	252	CG	ARG	17	-8.587	17.243	-0.076	1.00	0.00	C
ATOM	253	HG2	ARG	17	-9.396	16.667	0.374	1.00	0.00	H
ATOM	254	HG3	ARG	17	-7.848	16.618	-0.574	1.00	0.00	H
ATOM	255	CD	ARG	17	-7.853	17.935	1.106	1.00	0.00	C
ATOM	256	HD2	ARG	17	-7.450	17.136	1.728	1.00	0.00	H
ATOM	257	HD3	ARG	17	-7.109	18.664	0.786	1.00	0.00	H
ATOM	258	NE	ARG	17	-8.813	18.590	1.938	1.00	0.00	N
ATOM	259	HE	ARG	17	-9.793	18.555	1.700	1.00	0.00	H
ATOM	260	CZ	ARG	17	-8.577	19.475	2.904	1.00	0.00	C
ATOM	261	NH1	ARG	17	-7.350	19.659	3.356	1.00	0.00	N
ATOM	262	HH11	ARG	17	-6.615	19.297	2.766	1.00	0.00	H
ATOM	263	HH12	ARG	17	-7.122	20.470	3.913	1.00	0.00	H
ATOM	264	NH2	ARG	17	-9.392	20.251	3.438	1.00	0.00	N
ATOM	265	HH21	ARG	17	-9.138	20.771	4.266	1.00	0.00	H
ATOM	266	HH22	ARG	17	-10.118	20.527	2.792	1.00	0.00	H
ATOM	267	C	ARG	17	-10.542	18.731	-3.080	1.00	0.00	C
ATOM	268	O	ARG	17	-10.019	19.505	-3.852	1.00	0.00	O
ATOM	269	N	TYR	18	-11.805	18.911	-2.627	1.00	0.00	N
ATOM	270	H	TYR	18	-12.155	18.171	-2.035	1.00	0.00	H
ATOM	271	CA	TYR	18	-12.720	19.980	-3.147	1.00	0.00	C
ATOM	272	HA	TYR	18	-12.520	20.037	-4.217	1.00	0.00	H
ATOM	273	CB	TYR	18	-14.180	19.533	-2.920	1.00	0.00	C
ATOM	274	HB2	TYR	18	-14.244	18.578	-3.440	1.00	0.00	H
ATOM	275	HB3	TYR	18	-14.504	19.529	-1.879	1.00	0.00	H
ATOM	276	CG	TYR	18	-15.198	20.412	-3.786	1.00	0.00	C
ATOM	277	CD1	TYR	18	-15.880	21.515	-3.301	1.00	0.00	C
ATOM	278	HD1	TYR	18	-15.964	21.712	-2.242	1.00	0.00	H
ATOM	279	CE1	TYR	18	-16.775	22.233	-4.092	1.00	0.00	C
ATOM	280	HE1	TYR	18	-17.109	23.199	-3.744	1.00	0.00	H
ATOM	281	CZ	TYR	18	-16.809	22.015	-5.469	1.00	0.00	C
ATOM	282	OH	TYR	18	-17.429	22.903	-6.255	1.00	0.00	O
ATOM	283	HH	TYR	18	-17.430	22.648	-7.181	1.00	0.00	H
ATOM	284	CE2	TYR	18	-16.148	20.906	-6.017	1.00	0.00	C
ATOM	285	HE2	TYR	18	-16.171	20.734	-7.083	1.00	0.00	H
ATOM	286	CD2	TYR	18	-15.371	20.089	-5.165	1.00	0.00	C
ATOM	287	HD2	TYR	18	-14.917	19.191	-5.559	1.00	0.00	H
ATOM	288	C	TYR	18	-12.401	21.374	-2.369	1.00	0.00	C
ATOM	289	O	TYR	18	-12.249	22.381	-2.992	1.00	0.00	O
ATOM	290	N	TYR	19	-12.283	21.315	-0.989	1.00	0.00	N
ATOM	291	H	TYR	19	-12.495	20.451	-0.512	1.00	0.00	H
ATOM	292	CA	TYR	19	-11.905	22.460	-0.166	1.00	0.00	C
ATOM	293	HA	TYR	19	-11.782	23.296	-0.853	1.00	0.00	H
ATOM	294	CB	TYR	19	-13.088	22.695	0.781	1.00	0.00	C
ATOM	295	HB2	TYR	19	-13.991	22.438	0.227	1.00	0.00	H
ATOM	296	HB3	TYR	19	-12.822	21.927	1.507	1.00	0.00	H
ATOM	297	CG	TYR	19	-13.065	24.108	1.361	1.00	0.00	C
ATOM	298	CD1	TYR	19	-12.638	24.347	2.706	1.00	0.00	C
ATOM	299	HD1	TYR	19	-12.245	23.466	3.190	1.00	0.00	H
ATOM	300	CE1	TYR	19	-12.694	25.640	3.198	1.00	0.00	C
ATOM	301	HE1	TYR	19	-12.315	25.699	4.208	1.00	0.00	H
ATOM	302	CZ	TYR	19	-13.092	26.702	2.414	1.00	0.00	C
ATOM	303	OH	TYR	19	-13.093	28.024	2.852	1.00	0.00	O
ATOM	304	HH	TYR	19	-13.105	28.053	3.812	1.00	0.00	H
ATOM	305	CE2	TYR	19	-13.636	26.486	1.066	1.00	0.00	C
ATOM	306	HE2	TYR	19	-14.118	27.278	0.512	1.00	0.00	H

ATOM	307	CD2	TYR	19	-13.559	25.191	0.494	1.00	0.00	C
ATOM	308	HD2	TYR	19	-13.952	24.958	-0.485	1.00	0.00	H
ATOM	309	C	TYR	19	-10.542	22.210	0.572	1.00	0.00	C
ATOM	310	O	TYR	19	-10.148	21.087	0.814	1.00	0.00	O
ATOM	311	N	ARG	20	-9.853	23.277	0.944	1.00	0.00	N
ATOM	312	H	ARG	20	-10.267	24.164	0.693	1.00	0.00	H
ATOM	313	CA	ARG	20	-8.530	23.227	1.532	1.00	0.00	C
ATOM	314	HA	ARG	20	-8.520	22.412	2.255	1.00	0.00	H
ATOM	315	CB	ARG	20	-7.571	22.841	0.362	1.00	0.00	C
ATOM	316	HB2	ARG	20	-8.029	22.210	-0.399	1.00	0.00	H
ATOM	317	HB3	ARG	20	-7.290	23.769	-0.136	1.00	0.00	H
ATOM	318	CG	ARG	20	-6.244	22.253	0.815	1.00	0.00	C
ATOM	319	HG2	ARG	20	-5.556	22.942	1.304	1.00	0.00	H
ATOM	320	HG3	ARG	20	-6.508	21.398	1.437	1.00	0.00	H
ATOM	321	CD	ARG	20	-5.462	21.743	-0.473	1.00	0.00	C
ATOM	322	HD2	ARG	20	-6.240	21.222	-1.032	1.00	0.00	H
ATOM	323	HD3	ARG	20	-4.968	22.617	-0.894	1.00	0.00	H
ATOM	324	NE	ARG	20	-4.517	20.634	-0.210	1.00	0.00	N
ATOM	325	HE	ARG	20	-4.330	20.418	0.759	1.00	0.00	H
ATOM	326	CZ	ARG	20	-3.654	20.194	-1.083	1.00	0.00	C
ATOM	327	NH1	ARG	20	-3.751	20.470	-2.367	1.00	0.00	N
ATOM	328	HH11	ARG	20	-4.459	21.155	-2.591	1.00	0.00	H
ATOM	329	HH12	ARG	20	-3.107	20.009	-2.994	1.00	0.00	H
ATOM	330	NH2	ARG	20	-2.753	19.345	-0.679	1.00	0.00	N
ATOM	331	HH21	ARG	20	-2.154	18.922	-1.374	1.00	0.00	H
ATOM	332	HH22	ARG	20	-2.844	18.918	0.233	1.00	0.00	H
ATOM	333	C	ARG	20	-8.243	24.373	2.401	1.00	0.00	C
ATOM	334	O	ARG	20	-8.504	25.475	1.912	1.00	0.00	O
ATOM	335	N	GLU	21	-7.794	24.195	3.626	1.00	0.00	N
ATOM	336	H	GLU	21	-7.591	23.271	3.981	1.00	0.00	H
ATOM	337	CA	GLU	21	-7.466	25.302	4.559	1.00	0.00	C
ATOM	338	HA	GLU	21	-8.022	26.190	4.256	1.00	0.00	H
ATOM	339	CB	GLU	21	-7.873	24.885	5.970	1.00	0.00	C
ATOM	340	HB2	GLU	21	-7.757	25.710	6.673	1.00	0.00	H
ATOM	341	HB3	GLU	21	-8.935	24.642	5.949	1.00	0.00	H
ATOM	342	CG	GLU	21	-7.162	23.648	6.516	1.00	0.00	C
ATOM	343	HG2	GLU	21	-6.100	23.864	6.629	1.00	0.00	H
ATOM	344	HG3	GLU	21	-7.373	23.481	7.572	1.00	0.00	H
ATOM	345	CD	GLU	21	-7.340	22.349	5.734	1.00	0.00	C
ATOM	346	OE1	GLU	21	-8.482	21.812	5.711	1.00	0.00	O
ATOM	347	OE2	GLU	21	-6.446	22.009	4.983	1.00	0.00	O
ATOM	348	C	GLU	21	-6.016	25.694	4.458	1.00	0.00	C
ATOM	349	O	GLU	21	-5.723	26.861	4.714	1.00	0.00	O
ATOM	350	N	ASN	22	-5.068	24.749	4.279	1.00	0.00	N
ATOM	351	H	ASN	22	-5.396	23.795	4.220	1.00	0.00	H
ATOM	352	CA	ASN	22	-3.692	24.886	4.413	1.00	0.00	C
ATOM	353	HA	ASN	22	-3.394	25.885	4.098	1.00	0.00	H
ATOM	354	CB	ASN	22	-3.207	24.697	5.922	1.00	0.00	C
ATOM	355	HB2	ASN	22	-3.907	25.278	6.523	1.00	0.00	H
ATOM	356	HB3	ASN	22	-3.433	23.682	6.248	1.00	0.00	H
ATOM	357	CG	ASN	22	-1.813	25.100	6.189	1.00	0.00	C
ATOM	358	OD1	ASN	22	-1.507	26.258	6.480	1.00	0.00	O
ATOM	359	ND2	ASN	22	-0.860	24.208	6.197	1.00	0.00	N
ATOM	360	HD21	ASN	22	0.097	24.487	6.027	1.00	0.00	H
ATOM	361	HD22	ASN	22	-1.075	23.222	6.159	1.00	0.00	H
ATOM	362	C	ASN	22	-2.945	23.886	3.470	1.00	0.00	C
ATOM	363	O	ASN	22	-2.969	22.672	3.683	1.00	0.00	O
ATOM	364	N	MET	23	-2.306	24.436	2.479	1.00	0.00	N
ATOM	365	H	MET	23	-2.162	25.432	2.390	1.00	0.00	H
ATOM	366	CA	MET	23	-1.791	23.590	1.354	1.00	0.00	C
ATOM	367	HA	MET	23	-2.554	23.005	0.842	1.00	0.00	H
ATOM	368	CB	MET	23	-1.012	24.565	0.439	1.00	0.00	C
ATOM	369	HB2	MET	23	-0.210	25.097	0.951	1.00	0.00	H

ATOM	370	HB3	MET	23	-0.464	24.022	-0.331	1.00	0.00	H
ATOM	371	CG	MET	23	-2.016	25.539	-0.160	1.00	0.00	C
ATOM	372	HG2	MET	23	-2.597	25.994	0.643	1.00	0.00	H
ATOM	373	HG3	MET	23	-1.297	26.172	-0.680	1.00	0.00	H
ATOM	374	SD	MET	23	-3.240	24.691	-1.201	1.00	0.00	S
ATOM	375	CE	MET	23	-3.940	25.986	-2.263	1.00	0.00	C
ATOM	376	HE1	MET	23	-3.184	26.187	-3.021	1.00	0.00	H
ATOM	377	HE2	MET	23	-4.884	25.635	-2.680	1.00	0.00	H
ATOM	378	HE3	MET	23	-4.298	26.837	-1.684	1.00	0.00	H
ATOM	379	C	MET	23	-0.744	22.575	1.783	1.00	0.00	C
ATOM	380	O	MET	23	-0.542	21.579	1.083	1.00	0.00	O
ATOM	381	N	TYR	24	-0.040	22.774	2.841	1.00	0.00	N
ATOM	382	H	TYR	24	-0.197	23.571	3.442	1.00	0.00	H
ATOM	383	CA	TYR	24	1.011	21.882	3.303	1.00	0.00	C
ATOM	384	HA	TYR	24	1.001	20.981	2.690	1.00	0.00	H
ATOM	385	CB	TYR	24	2.408	22.493	3.116	1.00	0.00	C
ATOM	386	HB2	TYR	24	3.111	21.661	3.153	1.00	0.00	H
ATOM	387	HB3	TYR	24	2.513	22.930	2.122	1.00	0.00	H
ATOM	388	CG	TYR	24	2.783	23.576	4.155	1.00	0.00	C
ATOM	389	CD1	TYR	24	3.450	23.229	5.302	1.00	0.00	C
ATOM	390	HD1	TYR	24	3.784	22.214	5.456	1.00	0.00	H
ATOM	391	CE1	TYR	24	3.757	24.116	6.316	1.00	0.00	C
ATOM	392	HE1	TYR	24	4.272	23.677	7.159	1.00	0.00	H
ATOM	393	CZ	TYR	24	3.317	25.452	6.264	1.00	0.00	C
ATOM	394	OH	TYR	24	3.576	26.412	7.220	1.00	0.00	O
ATOM	395	HH	TYR	24	4.303	26.096	7.761	1.00	0.00	H
ATOM	396	CE2	TYR	24	2.691	25.852	5.048	1.00	0.00	C
ATOM	397	HE2	TYR	24	2.522	26.905	4.879	1.00	0.00	H
ATOM	398	CD2	TYR	24	2.339	24.916	4.040	1.00	0.00	C
ATOM	399	HD2	TYR	24	2.002	25.137	3.038	1.00	0.00	H
ATOM	400	C	TYR	24	0.752	21.175	4.677	1.00	0.00	C
ATOM	401	O	TYR	24	-0.200	21.585	5.384	1.00	0.00	O
ATOM	402	N	ARG	25	1.615	20.253	5.076	1.00	0.00	N
ATOM	403	H	ARG	25	2.382	19.991	4.473	1.00	0.00	H
ATOM	404	CA	ARG	25	1.734	19.681	6.423	1.00	0.00	C
ATOM	405	HA	ARG	25	1.349	20.403	7.142	1.00	0.00	H
ATOM	406	CB	ARG	25	1.010	18.290	6.665	1.00	0.00	C
ATOM	407	HB2	ARG	25	1.465	17.525	6.035	1.00	0.00	H
ATOM	408	HB3	ARG	25	0.969	17.888	7.677	1.00	0.00	H
ATOM	409	CG	ARG	25	-0.433	18.230	6.271	1.00	0.00	C
ATOM	410	HG2	ARG	25	-0.589	18.724	5.312	1.00	0.00	H
ATOM	411	HG3	ARG	25	-0.602	17.167	6.104	1.00	0.00	H
ATOM	412	CD	ARG	25	-1.423	18.959	7.280	1.00	0.00	C
ATOM	413	HD2	ARG	25	-1.387	18.412	8.223	1.00	0.00	H
ATOM	414	HD3	ARG	25	-1.170	20.003	7.469	1.00	0.00	H
ATOM	415	NE	ARG	25	-2.852	19.003	6.876	1.00	0.00	N
ATOM	416	HE	ARG	25	-3.457	18.293	7.263	1.00	0.00	H
ATOM	417	CZ	ARG	25	-3.493	19.909	6.156	1.00	0.00	C
ATOM	418	NH1	ARG	25	-2.727	20.789	5.494	1.00	0.00	N
ATOM	419	HH11	ARG	25	-1.732	20.647	5.592	1.00	0.00	H
ATOM	420	HH12	ARG	25	-3.112	21.594	5.018	1.00	0.00	H
ATOM	421	NH2	ARG	25	-4.813	19.950	6.156	1.00	0.00	N
ATOM	422	HH21	ARG	25	-5.345	19.451	6.855	1.00	0.00	H
ATOM	423	HH22	ARG	25	-5.302	20.642	5.606	1.00	0.00	H
ATOM	424	C	ARG	25	3.231	19.445	6.773	1.00	0.00	C
ATOM	425	O	ARG	25	4.148	19.449	6.006	1.00	0.00	O
ATOM	426	N	TYR	26	3.451	19.288	8.087	1.00	0.00	N
ATOM	427	H	TYR	26	2.593	19.315	8.619	1.00	0.00	H
ATOM	428	CA	TYR	26	4.709	19.271	8.724	1.00	0.00	C
ATOM	429	HA	TYR	26	5.349	20.025	8.266	1.00	0.00	H
ATOM	430	CB	TYR	26	4.497	19.619	10.214	1.00	0.00	C
ATOM	431	HB2	TYR	26	3.872	18.835	10.642	1.00	0.00	H
ATOM	432	HB3	TYR	26	5.500	19.563	10.635	1.00	0.00	H

```
ATOM    433  CG  TYR    26       3.690  20.883  10.562  1.00  0.00           C
ATOM    434  CD1 TYR    26       3.810  22.040   9.770  1.00  0.00           C
ATOM    435  HD1 TYR    26       4.441  22.185   8.906  1.00  0.00           H
ATOM    436  CE1 TYR    26       3.123  23.220  10.145  1.00  0.00           C
ATOM    437  HE1 TYR    26       3.197  24.115   9.546  1.00  0.00           H
ATOM    438  CZ  TYR    26       2.329  23.230  11.323  1.00  0.00           C
ATOM    439  OH  TYR    26       1.670  24.401  11.674  1.00  0.00           O
ATOM    440  HH  TYR    26       1.146  24.376  12.479  1.00  0.00           H
ATOM    441  CE2 TYR    26       2.142  22.034  12.086  1.00  0.00           C
ATOM    442  HE2 TYR    26       1.509  22.091  12.959  1.00  0.00           H
ATOM    443  CD2 TYR    26       2.875  20.863  11.739  1.00  0.00           C
ATOM    444  HD2 TYR    26       2.855  19.996  12.383  1.00  0.00           H
ATOM    445  C   TYR    26       5.399  17.946   8.647  1.00  0.00           C
ATOM    446  O   TYR    26       4.904  16.935   9.194  1.00  0.00           O
ATOM    447  N   NME    27       6.479  17.916   7.905  1.00  0.00           N
ATOM    448  H   NME    27       6.820  18.812   7.590  1.00  0.00           H
ATOM    449  CH3 NME    27       7.177  16.654   7.651  1.00  0.00           C
ATOM    450 HH31 NME    27       7.674  16.350   8.572  1.00  0.00           H
ATOM    451 HH32 NME    27       6.488  15.887   7.298  1.00  0.00           H
ATOM    452 HH33 NME    27       7.850  16.817   6.808  1.00  0.00           H
TER     453      NME    27
END
```

Cluster 4:

```
ATOM      1 HH31 ACE     1       9.621  14.666   5.540  1.00  0.00           H
ATOM      2  CH3 ACE     1       9.527  13.582   5.584  1.00  0.00           C
ATOM      3 HH32 ACE     1       9.434  13.167   6.588  1.00  0.00           H
ATOM      4 HH33 ACE     1      10.430  13.157   5.143  1.00  0.00           H
ATOM      5  C   ACE     1       8.308  13.232   4.764  1.00  0.00           C
ATOM      6  O   ACE     1       8.245  12.157   4.066  1.00  0.00           O
ATOM      7  N   VAL     2       7.272  14.044   4.894  1.00  0.00           N
ATOM      8  H   VAL     2       7.393  14.955   5.313  1.00  0.00           H
ATOM      9  CA  VAL     2       5.985  13.807   4.320  1.00  0.00           C
ATOM     10  HA  VAL     2       5.904  12.732   4.485  1.00  0.00           H
ATOM     11  CB  VAL     2       4.781  14.350   5.128  1.00  0.00           C
ATOM     12  HB  VAL     2       3.829  14.081   4.672  1.00  0.00           H
ATOM     13  CG1 VAL     2       4.806  14.007   6.620  1.00  0.00           C
ATOM     14 HG11 VAL     2       4.831  12.948   6.879  1.00  0.00           H
ATOM     15 HG12 VAL     2       5.739  14.308   7.095  1.00  0.00           H
ATOM     16 HG13 VAL     2       3.988  14.535   7.112  1.00  0.00           H
ATOM     17  CG2 VAL     2       4.811  15.839   5.128  1.00  0.00           C
ATOM     18 HG21 VAL     2       5.784  16.264   5.376  1.00  0.00           H
ATOM     19 HG22 VAL     2       4.396  16.201   4.187  1.00  0.00           H
ATOM     20 HG23 VAL     2       4.224  16.376   5.872  1.00  0.00           H
ATOM     21  C   VAL     2       5.892  13.920   2.755  1.00  0.00           C
ATOM     22  O   VAL     2       4.792  13.846   2.240  1.00  0.00           O
ATOM     23  N   MET     3       6.936  14.205   2.039  1.00  0.00           N
ATOM     24  H   MET     3       7.784  14.293   2.581  1.00  0.00           H
ATOM     25  CA  MET     3       6.919  14.234   0.569  1.00  0.00           C
ATOM     26  HA  MET     3       6.095  14.856   0.221  1.00  0.00           H
ATOM     27  CB  MET     3       8.293  14.911   0.058  1.00  0.00           C
ATOM     28  HB2 MET     3       8.257  15.091  -1.017  1.00  0.00           H
ATOM     29  HB3 MET     3       8.309  15.863   0.589  1.00  0.00           H
ATOM     30  CG  MET     3       9.642  14.277   0.531  1.00  0.00           C
ATOM     31  HG2 MET     3      10.460  14.876   0.132  1.00  0.00           H
ATOM     32  HG3 MET     3       9.719  14.444   1.605  1.00  0.00           H
ATOM     33  SD  MET     3      10.028  12.567   0.185  1.00  0.00           S
ATOM     34  CE  MET     3      11.166  12.221   1.531  1.00  0.00           C
ATOM     35  HE1 MET     3      11.711  11.290   1.379  1.00  0.00           H
ATOM     36  HE2 MET     3      11.893  13.025   1.641  1.00  0.00           H
```

ATOM	37	HE3	MET	3	10.637	12.210	2.485	1.00	0.00	H
ATOM	38	C	MET	3	6.592	12.878	-0.186	1.00	0.00	C
ATOM	39	O	MET	3	6.237	12.970	-1.359	1.00	0.00	O
ATOM	40	N	SER	4	6.715	11.709	0.420	1.00	0.00	N
ATOM	41	H	SER	4	7.060	11.677	1.370	1.00	0.00	H
ATOM	42	CA	SER	4	6.380	10.389	-0.233	1.00	0.00	C
ATOM	43	HA	SER	4	5.873	10.566	-1.182	1.00	0.00	H
ATOM	44	CB	SER	4	7.668	9.662	-0.644	1.00	0.00	C
ATOM	45	HB2	SER	4	8.313	10.366	-1.170	1.00	0.00	H
ATOM	46	HB3	SER	4	8.136	9.536	0.332	1.00	0.00	H
ATOM	47	OG	SER	4	7.386	8.390	-1.295	1.00	0.00	O
ATOM	48	HG	SER	4	6.687	7.963	-0.795	1.00	0.00	H
ATOM	49	C	SER	4	5.370	9.629	0.708	1.00	0.00	C
ATOM	50	O	SER	4	5.332	8.402	0.582	1.00	0.00	O
ATOM	51	N	ARG	5	4.604	10.365	1.502	1.00	0.00	N
ATOM	52	H	ARG	5	4.888	11.323	1.648	1.00	0.00	H
ATOM	53	CA	ARG	5	3.638	9.779	2.539	1.00	0.00	C
ATOM	54	HA	ARG	5	4.180	9.244	3.319	1.00	0.00	H
ATOM	55	CB	ARG	5	2.803	10.968	3.217	1.00	0.00	C
ATOM	56	HB2	ARG	5	2.190	10.577	4.030	1.00	0.00	H
ATOM	57	HB3	ARG	5	3.524	11.603	3.731	1.00	0.00	H
ATOM	58	CG	ARG	5	1.971	11.829	2.330	1.00	0.00	C
ATOM	59	HG2	ARG	5	2.559	12.084	1.447	1.00	0.00	H
ATOM	60	HG3	ARG	5	1.133	11.254	1.937	1.00	0.00	H
ATOM	61	CD	ARG	5	1.497	13.153	2.899	1.00	0.00	C
ATOM	62	HD2	ARG	5	0.793	13.003	3.716	1.00	0.00	H
ATOM	63	HD3	ARG	5	2.363	13.753	3.177	1.00	0.00	H
ATOM	64	NE	ARG	5	0.833	13.991	2.001	1.00	0.00	N
ATOM	65	HE	ARG	5	-0.172	13.997	2.108	1.00	0.00	H
ATOM	66	CZ	ARG	5	1.251	14.736	1.026	1.00	0.00	C
ATOM	67	NH1	ARG	5	2.530	14.912	0.751	1.00	0.00	N
ATOM	68	HH11	ARG	5	3.239	14.379	1.234	1.00	0.00	H
ATOM	69	HH12	ARG	5	2.825	15.482	-0.030	1.00	0.00	H
ATOM	70	NH2	ARG	5	0.373	15.207	0.181	1.00	0.00	N
ATOM	71	HH21	ARG	5	-0.583	15.085	0.482	1.00	0.00	H
ATOM	72	HH22	ARG	5	0.568	15.693	-0.683	1.00	0.00	H
ATOM	73	C	ARG	5	2.672	8.760	1.837	1.00	0.00	C
ATOM	74	O	ARG	5	2.382	8.934	0.700	1.00	0.00	O
ATOM	75	N	PRO	6	2.121	7.654	2.413	1.00	0.00	N
ATOM	76	CD	PRO	6	2.339	7.312	3.800	1.00	0.00	C
ATOM	77	HD2	PRO	6	2.498	8.159	4.468	1.00	0.00	H
ATOM	78	HD3	PRO	6	3.206	6.666	3.938	1.00	0.00	H
ATOM	79	CG	PRO	6	1.145	6.452	4.280	1.00	0.00	C
ATOM	80	HG2	PRO	6	0.311	7.081	4.589	1.00	0.00	H
ATOM	81	HG3	PRO	6	1.498	5.771	5.054	1.00	0.00	H
ATOM	82	CB	PRO	6	0.858	5.671	3.009	1.00	0.00	C
ATOM	83	HB2	PRO	6	-0.174	5.330	2.923	1.00	0.00	H
ATOM	84	HB3	PRO	6	1.508	4.825	2.786	1.00	0.00	H
ATOM	85	CA	PRO	6	1.087	6.759	1.936	1.00	0.00	C
ATOM	86	HA	PRO	6	1.574	6.293	1.080	1.00	0.00	H
ATOM	87	C	PRO	6	-0.235	7.499	1.512	1.00	0.00	C
ATOM	88	O	PRO	6	-0.587	8.588	1.991	1.00	0.00	O
ATOM	89	N	LEU	7	-0.941	6.837	0.573	1.00	0.00	N
ATOM	90	H	LEU	7	-0.831	5.835	0.529	1.00	0.00	H
ATOM	91	CA	LEU	7	-2.088	7.343	-0.241	1.00	0.00	C
ATOM	92	HA	LEU	7	-1.737	8.217	-0.790	1.00	0.00	H
ATOM	93	CB	LEU	7	-2.614	6.297	-1.251	1.00	0.00	C
ATOM	94	HB2	LEU	7	-2.979	5.420	-0.716	1.00	0.00	H
ATOM	95	HB3	LEU	7	-3.508	6.640	-1.772	1.00	0.00	H
ATOM	96	CG	LEU	7	-1.528	5.824	-2.268	1.00	0.00	C
ATOM	97	HG	LEU	7	-0.680	5.285	-1.844	1.00	0.00	H
ATOM	98	CD1	LEU	7	-2.166	4.803	-3.240	1.00	0.00	C
ATOM	99	HD11	LEU	7	-2.598	3.970	-2.683	1.00	0.00	H

ATOM	100	HD12	LEU	7	-3.001	5.271	-3.763	1.00	0.00	H
ATOM	101	HD13	LEU	7	-1.435	4.483	-3.982	1.00	0.00	H
ATOM	102	CD2	LEU	7	-1.048	7.022	-3.062	1.00	0.00	C
ATOM	103	HD21	LEU	7	-1.873	7.729	-3.154	1.00	0.00	H
ATOM	104	HD22	LEU	7	-0.155	7.430	-2.589	1.00	0.00	H
ATOM	105	HD23	LEU	7	-0.778	6.769	-4.087	1.00	0.00	H
ATOM	106	C	LEU	7	-3.289	7.859	0.531	1.00	0.00	C
ATOM	107	O	LEU	7	-3.879	8.869	0.149	1.00	0.00	O
ATOM	108	N	ILE	8	-3.530	7.307	1.734	1.00	0.00	N
ATOM	109	H	ILE	8	-3.182	6.380	1.937	1.00	0.00	H
ATOM	110	CA	ILE	8	-4.490	7.832	2.703	1.00	0.00	C
ATOM	111	HA	ILE	8	-5.521	7.811	2.347	1.00	0.00	H
ATOM	112	CB	ILE	8	-4.552	6.940	3.968	1.00	0.00	C
ATOM	113	HB	ILE	8	-5.069	7.471	4.767	1.00	0.00	H
ATOM	114	CG2	ILE	8	-5.281	5.632	3.683	1.00	0.00	C
ATOM	115	HG21	ILE	8	-6.242	5.731	3.178	1.00	0.00	H
ATOM	116	HG22	ILE	8	-4.718	4.907	3.096	1.00	0.00	H
ATOM	117	HG23	ILE	8	-5.470	5.188	4.661	1.00	0.00	H
ATOM	118	CG1	ILE	8	-3.170	6.594	4.616	1.00	0.00	C
ATOM	119	HG12	ILE	8	-2.671	5.846	4.001	1.00	0.00	H
ATOM	120	HG13	ILE	8	-2.562	7.498	4.585	1.00	0.00	H
ATOM	121	CD1	ILE	8	-3.284	6.304	6.100	1.00	0.00	C
ATOM	122	HD11	ILE	8	-3.659	7.177	6.633	1.00	0.00	H
ATOM	123	HD12	ILE	8	-3.961	5.504	6.401	1.00	0.00	H
ATOM	124	HD13	ILE	8	-2.324	6.004	6.521	1.00	0.00	H
ATOM	125	C	ILE	8	-4.242	9.318	3.170	1.00	0.00	C
ATOM	126	O	ILE	8	-5.230	9.944	3.484	1.00	0.00	O
ATOM	127	N	HID	9	-2.987	9.809	3.232	1.00	0.00	N
ATOM	128	H	HID	9	-2.307	9.236	2.754	1.00	0.00	H
ATOM	129	CA	HID	9	-2.717	11.203	3.566	1.00	0.00	C
ATOM	130	HA	HID	9	-3.522	11.619	4.171	1.00	0.00	H
ATOM	131	CB	HID	9	-1.510	11.269	4.587	1.00	0.00	C
ATOM	132	HB2	HID	9	-0.553	11.207	4.067	1.00	0.00	H
ATOM	133	HB3	HID	9	-1.512	12.255	5.049	1.00	0.00	H
ATOM	134	CG	HID	9	-1.471	10.392	5.820	1.00	0.00	C
ATOM	135	ND1	HID	9	-2.465	10.112	6.723	1.00	0.00	N
ATOM	136	HD1	HID	9	-3.452	10.318	6.674	1.00	0.00	H
ATOM	137	CE1	HID	9	-1.904	9.337	7.700	1.00	0.00	C
ATOM	138	HE1	HID	9	-2.425	8.937	8.558	1.00	0.00	H
ATOM	139	NE2	HID	9	-0.566	9.179	7.452	1.00	0.00	N
ATOM	140	CD2	HID	9	-0.325	9.841	6.253	1.00	0.00	C
ATOM	141	HD2	HID	9	0.598	9.914	5.697	1.00	0.00	H
ATOM	142	C	HID	9	-2.452	12.057	2.341	1.00	0.00	C
ATOM	143	O	HID	9	-1.791	13.086	2.522	1.00	0.00	O
ATOM	144	N	PHE	10	-3.007	11.712	1.166	1.00	0.00	N
ATOM	145	H	PHE	10	-3.485	10.822	1.196	1.00	0.00	H
ATOM	146	CA	PHE	10	-2.864	12.417	-0.104	1.00	0.00	C
ATOM	147	HA	PHE	10	-2.599	13.456	0.089	1.00	0.00	H
ATOM	148	CB	PHE	10	-1.716	11.700	-0.887	1.00	0.00	C
ATOM	149	HB2	PHE	10	-1.086	11.353	-0.067	1.00	0.00	H
ATOM	150	HB3	PHE	10	-2.175	10.849	-1.391	1.00	0.00	H
ATOM	151	CG	PHE	10	-0.874	12.393	-1.975	1.00	0.00	C
ATOM	152	CD1	PHE	10	-1.514	13.055	-3.023	1.00	0.00	C
ATOM	153	HD1	PHE	10	-2.579	12.996	-3.196	1.00	0.00	H
ATOM	154	CE1	PHE	10	-0.751	13.692	-3.976	1.00	0.00	C
ATOM	155	HE1	PHE	10	-1.216	14.194	-4.811	1.00	0.00	H
ATOM	156	CZ	PHE	10	0.640	13.658	-3.845	1.00	0.00	C
ATOM	157	HZ	PHE	10	1.234	14.139	-4.609	1.00	0.00	H
ATOM	158	CE2	PHE	10	1.361	12.960	-2.876	1.00	0.00	C
ATOM	159	HE2	PHE	10	2.436	13.013	-2.789	1.00	0.00	H
ATOM	160	CD2	PHE	10	0.556	12.371	-1.842	1.00	0.00	C
ATOM	161	HD2	PHE	10	1.037	11.827	-1.043	1.00	0.00	H
ATOM	162	C	PHE	10	-4.298	12.471	-0.789	1.00	0.00	C

ATOM	163	O	PHE	10	-4.617	13.560	-1.099	1.00	0.00	O
ATOM	164	N	GLY	11	-5.075	11.344	-1.022	1.00	0.00	N
ATOM	165	H	GLY	11	-4.626	10.502	-0.690	1.00	0.00	H
ATOM	166	CA	GLY	11	-6.407	11.257	-1.604	1.00	0.00	C
ATOM	167	HA2	GLY	11	-6.319	11.892	-2.486	1.00	0.00	H
ATOM	168	HA3	GLY	11	-6.673	10.234	-1.873	1.00	0.00	H
ATOM	169	C	GLY	11	-7.589	11.739	-0.662	1.00	0.00	C
ATOM	170	O	GLY	11	-8.484	12.372	-1.171	1.00	0.00	O
ATOM	171	N	ASN	12	-7.514	11.516	0.710	1.00	0.00	N
ATOM	172	H	ASN	12	-6.889	10.842	1.130	1.00	0.00	H
ATOM	173	CA	ASN	12	-8.457	12.068	1.695	1.00	0.00	C
ATOM	174	HA	ASN	12	-9.417	12.119	1.182	1.00	0.00	H
ATOM	175	CB	ASN	12	-8.600	11.107	2.934	1.00	0.00	C
ATOM	176	HB2	ASN	12	-8.789	10.092	2.586	1.00	0.00	H
ATOM	177	HB3	ASN	12	-7.643	11.124	3.456	1.00	0.00	H
ATOM	178	CG	ASN	12	-9.665	11.440	3.942	1.00	0.00	C
ATOM	179	OD1	ASN	12	-10.739	11.989	3.557	1.00	0.00	O
ATOM	180	ND2	ASN	12	-9.508	11.103	5.208	1.00	0.00	N
ATOM	181	HD21	ASN	12	-10.165	11.505	5.862	1.00	0.00	H
ATOM	182	HD22	ASN	12	-8.811	10.428	5.491	1.00	0.00	H
ATOM	183	C	ASN	12	-8.022	13.459	2.119	1.00	0.00	C
ATOM	184	O	ASN	12	-7.237	13.610	3.121	1.00	0.00	O
ATOM	185	N	ASP	13	-8.847	14.462	1.806	1.00	0.00	N
ATOM	186	H	ASP	13	-9.479	14.206	1.063	1.00	0.00	H
ATOM	187	CA	ASP	13	-8.745	15.911	2.101	1.00	0.00	C
ATOM	188	HA	ASP	13	-7.770	16.223	1.726	1.00	0.00	H
ATOM	189	CB	ASP	13	-9.862	16.615	1.279	1.00	0.00	C
ATOM	190	HB2	ASP	13	-9.789	16.394	0.213	1.00	0.00	H
ATOM	191	HB3	ASP	13	-10.799	16.271	1.716	1.00	0.00	H
ATOM	192	CG	ASP	13	-9.671	18.159	1.471	1.00	0.00	C
ATOM	193	OD1	ASP	13	-10.496	18.612	2.221	1.00	0.00	O
ATOM	194	OD2	ASP	13	-8.913	18.885	0.859	1.00	0.00	O
ATOM	195	C	ASP	13	-8.875	16.238	3.561	1.00	0.00	C
ATOM	196	O	ASP	13	-8.343	17.176	4.043	1.00	0.00	O
ATOM	197	N	TYR	14	-9.496	15.396	4.312	1.00	0.00	N
ATOM	198	H	TYR	14	-9.869	14.622	3.780	1.00	0.00	H
ATOM	199	CA	TYR	14	-9.800	15.441	5.783	1.00	0.00	C
ATOM	200	HA	TYR	14	-10.162	16.416	6.109	1.00	0.00	H
ATOM	201	CB	TYR	14	-10.853	14.493	6.307	1.00	0.00	C
ATOM	202	HB2	TYR	14	-11.522	14.320	5.464	1.00	0.00	H
ATOM	203	HB3	TYR	14	-10.406	13.526	6.538	1.00	0.00	H
ATOM	204	CG	TYR	14	-11.721	14.977	7.481	1.00	0.00	C
ATOM	205	CD1	TYR	14	-12.717	15.897	7.266	1.00	0.00	C
ATOM	206	HD1	TYR	14	-12.869	16.377	6.311	1.00	0.00	H
ATOM	207	CE1	TYR	14	-13.567	16.256	8.313	1.00	0.00	C
ATOM	208	HE1	TYR	14	-14.377	16.959	8.192	1.00	0.00	H
ATOM	209	CZ	TYR	14	-13.446	15.799	9.590	1.00	0.00	C
ATOM	210	OH	TYR	14	-14.423	16.254	10.384	1.00	0.00	O
ATOM	211	HH	TYR	14	-14.255	16.057	11.308	1.00	0.00	H
ATOM	212	CE2	TYR	14	-12.475	14.809	9.886	1.00	0.00	C
ATOM	213	HE2	TYR	14	-12.383	14.512	10.919	1.00	0.00	H
ATOM	214	CD2	TYR	14	-11.605	14.361	8.783	1.00	0.00	C
ATOM	215	HD2	TYR	14	-10.779	13.682	8.932	1.00	0.00	H
ATOM	216	C	TYR	14	-8.460	15.200	6.517	1.00	0.00	C
ATOM	217	O	TYR	14	-8.309	15.509	7.728	1.00	0.00	O
ATOM	218	N	GLU	15	-7.482	14.611	5.855	1.00	0.00	N
ATOM	219	H	GLU	15	-7.707	14.509	4.876	1.00	0.00	H
ATOM	220	CA	GLU	15	-6.124	14.277	6.428	1.00	0.00	C
ATOM	221	HA	GLU	15	-5.994	14.531	7.481	1.00	0.00	H
ATOM	222	CB	GLU	15	-5.941	12.780	6.158	1.00	0.00	C
ATOM	223	HB2	GLU	15	-6.463	12.458	5.258	1.00	0.00	H
ATOM	224	HB3	GLU	15	-4.860	12.642	6.158	1.00	0.00	H
ATOM	225	CG	GLU	15	-6.573	11.906	7.316	1.00	0.00	C

ATOM	226	HG2	GLU	15	-6.073	12.224	8.231	1.00	0.00	H
ATOM	227	HG3	GLU	15	-7.623	12.134	7.502	1.00	0.00	H
ATOM	228	CD	GLU	15	-6.368	10.431	6.924	1.00	0.00	C
ATOM	229	OE1	GLU	15	-5.260	9.843	7.053	1.00	0.00	O
ATOM	230	OE2	GLU	15	-7.392	9.703	6.713	1.00	0.00	O
ATOM	231	C	GLU	15	-5.044	15.024	5.691	1.00	0.00	C
ATOM	232	O	GLU	15	-4.188	15.574	6.349	1.00	0.00	O
ATOM	233	N	ASP	16	-5.244	15.233	4.397	1.00	0.00	N
ATOM	234	H	ASP	16	-6.085	14.886	3.956	1.00	0.00	H
ATOM	235	CA	ASP	16	-4.136	15.716	3.528	1.00	0.00	C
ATOM	236	HA	ASP	16	-3.241	15.146	3.779	1.00	0.00	H
ATOM	237	CB	ASP	16	-4.374	15.439	2.086	1.00	0.00	C
ATOM	238	HB2	ASP	16	-4.194	14.385	1.878	1.00	0.00	H
ATOM	239	HB3	ASP	16	-5.415	15.659	1.854	1.00	0.00	H
ATOM	240	CG	ASP	16	-3.429	16.207	1.122	1.00	0.00	C
ATOM	241	OD1	ASP	16	-3.865	17.280	0.682	1.00	0.00	O
ATOM	242	OD2	ASP	16	-2.219	15.894	0.930	1.00	0.00	O
ATOM	243	C	ASP	16	-3.608	17.173	3.966	1.00	0.00	C
ATOM	244	O	ASP	16	-2.368	17.476	4.016	1.00	0.00	O
ATOM	245	N	ARG	17	-4.510	18.103	4.304	1.00	0.00	N
ATOM	246	H	ARG	17	-5.442	17.814	4.039	1.00	0.00	H
ATOM	247	CA	ARG	17	-4.389	19.411	4.851	1.00	0.00	C
ATOM	248	HA	ARG	17	-4.262	20.120	4.032	1.00	0.00	H
ATOM	249	CB	ARG	17	-5.717	19.812	5.598	1.00	0.00	C
ATOM	250	HB2	ARG	17	-5.527	20.796	6.024	1.00	0.00	H
ATOM	251	HB3	ARG	17	-6.570	19.853	4.920	1.00	0.00	H
ATOM	252	CG	ARG	17	-6.120	18.898	6.761	1.00	0.00	C
ATOM	253	HG2	ARG	17	-6.084	17.844	6.484	1.00	0.00	H
ATOM	254	HG3	ARG	17	-5.464	19.142	7.597	1.00	0.00	H
ATOM	255	CD	ARG	17	-7.540	19.119	7.345	1.00	0.00	C
ATOM	256	HD2	ARG	17	-7.617	18.486	8.229	1.00	0.00	H
ATOM	257	HD3	ARG	17	-7.708	20.178	7.542	1.00	0.00	H
ATOM	258	NE	ARG	17	-8.614	18.835	6.321	1.00	0.00	N
ATOM	259	HE	ARG	17	-8.341	18.161	5.620	1.00	0.00	H
ATOM	260	CZ	ARG	17	-9.795	19.371	6.212	1.00	0.00	C
ATOM	261	NH1	ARG	17	-10.262	20.205	7.047	1.00	0.00	N
ATOM	262	HH11	ARG	17	-9.554	20.505	7.702	1.00	0.00	H
ATOM	263	HH12	ARG	17	-11.266	20.325	7.046	1.00	0.00	H
ATOM	264	NH2	ARG	17	-10.644	19.177	5.165	1.00	0.00	N
ATOM	265	HH21	ARG	17	-10.457	18.617	4.346	1.00	0.00	H
ATOM	266	HH22	ARG	17	-11.554	19.613	5.228	1.00	0.00	H
ATOM	267	C	ARG	17	-3.247	19.585	5.809	1.00	0.00	C
ATOM	268	O	ARG	17	-2.581	20.640	5.747	1.00	0.00	O
ATOM	269	N	TYR	18	-2.933	18.560	6.648	1.00	0.00	N
ATOM	270	H	TYR	18	-3.412	17.673	6.580	1.00	0.00	H
ATOM	271	CA	TYR	18	-1.930	18.643	7.649	1.00	0.00	C
ATOM	272	HA	TYR	18	-2.065	19.577	8.192	1.00	0.00	H
ATOM	273	CB	TYR	18	-2.144	17.529	8.691	1.00	0.00	C
ATOM	274	HB2	TYR	18	-1.963	16.565	8.216	1.00	0.00	H
ATOM	275	HB3	TYR	18	-1.374	17.578	9.461	1.00	0.00	H
ATOM	276	CG	TYR	18	-3.473	17.568	9.406	1.00	0.00	C
ATOM	277	CD1	TYR	18	-4.333	16.493	9.285	1.00	0.00	C
ATOM	278	HD1	TYR	18	-4.005	15.598	8.778	1.00	0.00	H
ATOM	279	CE1	TYR	18	-5.611	16.625	9.898	1.00	0.00	C
ATOM	280	HE1	TYR	18	-6.338	15.847	9.717	1.00	0.00	H
ATOM	281	CZ	TYR	18	-6.005	17.817	10.421	1.00	0.00	C
ATOM	282	OH	TYR	18	-7.263	17.910	10.926	1.00	0.00	O
ATOM	283	HH	TYR	18	-7.733	17.075	10.861	1.00	0.00	H
ATOM	284	CE2	TYR	18	-5.089	18.885	10.539	1.00	0.00	C
ATOM	285	HE2	TYR	18	-5.373	19.785	11.063	1.00	0.00	H
ATOM	286	CD2	TYR	18	-3.782	18.786	10.101	1.00	0.00	C
ATOM	287	HD2	TYR	18	-3.115	19.632	10.185	1.00	0.00	H
ATOM	288	C	TYR	18	-0.518	18.532	7.011	1.00	0.00	C

ATOM	289	O	TYR	18	0.503	18.746	7.685	1.00	0.00	O
ATOM	290	N	TYR	19	-0.383	18.037	5.758	1.00	0.00	N
ATOM	291	H	TYR	19	-1.144	17.864	5.117	1.00	0.00	H
ATOM	292	CA	TYR	19	0.780	17.647	5.087	1.00	0.00	C
ATOM	293	HA	TYR	19	1.651	17.816	5.720	1.00	0.00	H
ATOM	294	CB	TYR	19	0.665	16.222	4.703	1.00	0.00	C
ATOM	295	HB2	TYR	19	-0.040	16.188	3.873	1.00	0.00	H
ATOM	296	HB3	TYR	19	1.592	15.916	4.219	1.00	0.00	H
ATOM	297	CG	TYR	19	0.296	15.361	5.890	1.00	0.00	C
ATOM	298	CD1	TYR	19	1.302	15.011	6.824	1.00	0.00	C
ATOM	299	HD1	TYR	19	2.261	15.506	6.869	1.00	0.00	H
ATOM	300	CE1	TYR	19	1.034	14.127	7.852	1.00	0.00	C
ATOM	301	HE1	TYR	19	1.818	13.861	8.544	1.00	0.00	H
ATOM	302	CZ	TYR	19	-0.298	13.657	8.079	1.00	0.00	C
ATOM	303	OH	TYR	19	-0.507	12.839	9.165	1.00	0.00	O
ATOM	304	HH	TYR	19	-1.407	12.507	9.183	1.00	0.00	H
ATOM	305	CE2	TYR	19	-1.284	13.988	7.086	1.00	0.00	C
ATOM	306	HE2	TYR	19	-2.305	13.637	7.120	1.00	0.00	H
ATOM	307	CD2	TYR	19	-0.973	14.770	5.981	1.00	0.00	C
ATOM	308	HD2	TYR	19	-1.758	15.122	5.326	1.00	0.00	H
ATOM	309	C	TYR	19	1.054	18.611	3.869	1.00	0.00	C
ATOM	310	O	TYR	19	2.218	18.833	3.631	1.00	0.00	O
ATOM	311	N	ARG	20	0.061	19.196	3.166	1.00	0.00	N
ATOM	312	H	ARG	20	-0.888	18.916	3.370	1.00	0.00	H
ATOM	313	CA	ARG	20	0.218	20.098	1.975	1.00	0.00	C
ATOM	314	HA	ARG	20	0.883	19.650	1.237	1.00	0.00	H
ATOM	315	CB	ARG	20	-1.157	20.327	1.198	1.00	0.00	C
ATOM	316	HB2	ARG	20	-0.921	20.839	0.266	1.00	0.00	H
ATOM	317	HB3	ARG	20	-1.405	19.297	0.943	1.00	0.00	H
ATOM	318	CG	ARG	20	-2.308	21.022	2.033	1.00	0.00	C
ATOM	319	HG2	ARG	20	-2.449	20.591	3.025	1.00	0.00	H
ATOM	320	HG3	ARG	20	-1.869	21.995	2.251	1.00	0.00	H
ATOM	321	CD	ARG	20	-3.578	21.406	1.338	1.00	0.00	C
ATOM	322	HD2	ARG	20	-4.152	22.111	1.940	1.00	0.00	H
ATOM	323	HD3	ARG	20	-3.342	21.968	0.435	1.00	0.00	H
ATOM	324	NE	ARG	20	-4.339	20.187	1.192	1.00	0.00	N
ATOM	325	HE	ARG	20	-3.790	19.345	1.287	1.00	0.00	H
ATOM	326	CZ	ARG	20	-5.569	20.066	0.845	1.00	0.00	C
ATOM	327	NH1	ARG	20	-6.470	21.021	0.759	1.00	0.00	N
ATOM	328	HH11	ARG	20	-6.139	21.948	0.984	1.00	0.00	H
ATOM	329	HH12	ARG	20	-7.459	20.836	0.672	1.00	0.00	H
ATOM	330	NH2	ARG	20	-5.988	18.846	0.642	1.00	0.00	N
ATOM	331	HH21	ARG	20	-5.319	18.109	0.471	1.00	0.00	H
ATOM	332	HH22	ARG	20	-6.946	18.691	0.358	1.00	0.00	H
ATOM	333	C	ARG	20	0.966	21.408	2.407	1.00	0.00	C
ATOM	334	O	ARG	20	0.997	21.852	3.565	1.00	0.00	O
ATOM	335	N	GLU	21	1.358	22.135	1.300	1.00	0.00	N
ATOM	336	H	GLU	21	1.235	21.831	0.345	1.00	0.00	H
ATOM	337	CA	GLU	21	2.192	23.379	1.481	1.00	0.00	C
ATOM	338	HA	GLU	21	2.723	23.173	2.410	1.00	0.00	H
ATOM	339	CB	GLU	21	3.281	23.391	0.343	1.00	0.00	C
ATOM	340	HB2	GLU	21	3.891	24.271	0.544	1.00	0.00	H
ATOM	341	HB3	GLU	21	3.993	22.581	0.500	1.00	0.00	H
ATOM	342	CG	GLU	21	2.634	23.409	-1.096	1.00	0.00	C
ATOM	343	HG2	GLU	21	1.836	22.668	-1.151	1.00	0.00	H
ATOM	344	HG3	GLU	21	2.194	24.384	-1.301	1.00	0.00	H
ATOM	345	CD	GLU	21	3.724	23.097	-2.103	1.00	0.00	C
ATOM	346	OE1	GLU	21	3.671	22.050	-2.734	1.00	0.00	O
ATOM	347	OE2	GLU	21	4.727	23.843	-2.124	1.00	0.00	O
ATOM	348	C	GLU	21	1.416	24.680	1.663	1.00	0.00	C
ATOM	349	O	GLU	21	1.997	25.700	2.182	1.00	0.00	O
ATOM	350	N	ASN	22	0.090	24.700	1.333	1.00	0.00	N
ATOM	351	H	ASN	22	-0.237	23.833	0.933	1.00	0.00	H

ATOM	352	CA	ASN	22	-0.813	25.823	1.543	1.00	0.00	C
ATOM	353	HA	ASN	22	-0.502	26.348	2.446	1.00	0.00	H
ATOM	354	CB	ASN	22	-0.603	26.712	0.313	1.00	0.00	C
ATOM	355	HB2	ASN	22	0.430	27.053	0.379	1.00	0.00	H
ATOM	356	HB3	ASN	22	-0.926	26.205	-0.597	1.00	0.00	H
ATOM	357	CG	ASN	22	-1.495	27.920	0.315	1.00	0.00	C
ATOM	358	OD1	ASN	22	-1.941	28.485	1.318	1.00	0.00	O
ATOM	359	ND2	ASN	22	-1.557	28.508	-0.817	1.00	0.00	N
ATOM	360	HD21	ASN	22	-2.034	29.394	-0.908	1.00	0.00	H
ATOM	361	HD22	ASN	22	-1.229	28.088	-1.674	1.00	0.00	H
ATOM	362	C	ASN	22	-2.256	25.216	1.581	1.00	0.00	C
ATOM	363	O	ASN	22	-2.461	24.161	0.982	1.00	0.00	O
ATOM	364	N	MET	23	-3.270	25.844	2.201	1.00	0.00	N
ATOM	365	H	MET	23	-3.166	26.741	2.654	1.00	0.00	H
ATOM	366	CA	MET	23	-4.540	25.203	2.365	1.00	0.00	C
ATOM	367	HA	MET	23	-4.290	24.200	2.711	1.00	0.00	H
ATOM	368	CB	MET	23	-5.312	25.975	3.372	1.00	0.00	C
ATOM	369	HB2	MET	23	-5.628	26.958	3.026	1.00	0.00	H
ATOM	370	HB3	MET	23	-6.225	25.411	3.562	1.00	0.00	H
ATOM	371	CG	MET	23	-4.693	26.280	4.724	1.00	0.00	C
ATOM	372	HG2	MET	23	-3.928	27.017	4.481	1.00	0.00	H
ATOM	373	HG3	MET	23	-5.430	26.745	5.378	1.00	0.00	H
ATOM	374	SD	MET	23	-3.949	24.928	5.692	1.00	0.00	S
ATOM	375	CE	MET	23	-3.056	25.921	6.979	1.00	0.00	C
ATOM	376	HE1	MET	23	-2.210	25.350	7.360	1.00	0.00	H
ATOM	377	HE2	MET	23	-2.686	26.808	6.467	1.00	0.00	H
ATOM	378	HE3	MET	23	-3.774	26.149	7.768	1.00	0.00	H
ATOM	379	C	MET	23	-5.397	25.082	1.113	1.00	0.00	C
ATOM	380	O	MET	23	-6.210	24.197	1.046	1.00	0.00	O
ATOM	381	N	TYR	24	-5.204	26.003	0.165	1.00	0.00	N
ATOM	382	H	TYR	24	-4.600	26.784	0.374	1.00	0.00	H
ATOM	383	CA	TYR	24	-6.161	26.096	-0.950	1.00	0.00	C
ATOM	384	HA	TYR	24	-7.018	25.483	-0.670	1.00	0.00	H
ATOM	385	CB	TYR	24	-6.645	27.544	-1.057	1.00	0.00	C
ATOM	386	HB2	TYR	24	-5.966	28.223	-1.571	1.00	0.00	H
ATOM	387	HB3	TYR	24	-7.531	27.452	-1.687	1.00	0.00	H
ATOM	388	CG	TYR	24	-7.126	28.080	0.298	1.00	0.00	C
ATOM	389	CD1	TYR	24	-7.992	27.432	1.112	1.00	0.00	C
ATOM	390	HD1	TYR	24	-8.556	26.599	0.720	1.00	0.00	H
ATOM	391	CE1	TYR	24	-8.292	27.934	2.364	1.00	0.00	C
ATOM	392	HE1	TYR	24	-9.051	27.404	2.921	1.00	0.00	H
ATOM	393	CZ	TYR	24	-7.645	29.113	2.896	1.00	0.00	C
ATOM	394	OH	TYR	24	-7.883	29.517	4.119	1.00	0.00	O
ATOM	395	HH	TYR	24	-7.396	30.333	4.261	1.00	0.00	H
ATOM	396	CE2	TYR	24	-6.942	29.885	1.980	1.00	0.00	C
ATOM	397	HE2	TYR	24	-6.519	30.800	2.366	1.00	0.00	H
ATOM	398	CD2	TYR	24	-6.556	29.352	0.733	1.00	0.00	C
ATOM	399	HD2	TYR	24	-5.872	29.923	0.122	1.00	0.00	H
ATOM	400	C	TYR	24	-5.657	25.560	-2.270	1.00	0.00	C
ATOM	401	O	TYR	24	-6.456	25.595	-3.283	1.00	0.00	O
ATOM	402	N	ARG	25	-4.335	25.128	-2.316	1.00	0.00	N
ATOM	403	H	ARG	25	-3.778	25.030	-1.479	1.00	0.00	H
ATOM	404	CA	ARG	25	-3.805	24.421	-3.506	1.00	0.00	C
ATOM	405	HA	ARG	25	-4.215	24.929	-4.378	1.00	0.00	H
ATOM	406	CB	ARG	25	-2.214	24.616	-3.490	1.00	0.00	C
ATOM	407	HB2	ARG	25	-2.051	25.684	-3.629	1.00	0.00	H
ATOM	408	HB3	ARG	25	-1.691	24.441	-2.550	1.00	0.00	H
ATOM	409	CG	ARG	25	-1.452	23.824	-4.614	1.00	0.00	C
ATOM	410	HG2	ARG	25	-0.416	24.164	-4.583	1.00	0.00	H
ATOM	411	HG3	ARG	25	-1.408	22.869	-4.089	1.00	0.00	H
ATOM	412	CD	ARG	25	-1.959	23.740	-6.035	1.00	0.00	C
ATOM	413	HD2	ARG	25	-1.106	23.339	-6.583	1.00	0.00	H
ATOM	414	HD3	ARG	25	-2.769	23.018	-6.143	1.00	0.00	H

```
ATOM    415  NE  ARG    25      -2.281  25.078  -6.527  1.00  0.00           N
ATOM    416  HE  ARG    25      -1.733  25.841  -6.156  1.00  0.00           H
ATOM    417  CZ  ARG    25      -3.069  25.298  -7.565  1.00  0.00           C
ATOM    418  NH1 ARG    25      -3.474  24.232  -8.286  1.00  0.00           N
ATOM    419 HH11 ARG    25      -3.729  23.354  -7.858  1.00  0.00           H
ATOM    420 HH12 ARG    25      -4.009  24.424  -9.119  1.00  0.00           H
ATOM    421  NH2 ARG    25      -3.342  26.442  -7.968  1.00  0.00           N
ATOM    422 HH21 ARG    25      -3.018  27.237  -7.435  1.00  0.00           H
ATOM    423 HH22 ARG    25      -3.934  26.487  -8.785  1.00  0.00           H
ATOM    424  C   ARG    25      -4.163  22.926  -3.547  1.00  0.00           C
ATOM    425  O   ARG    25      -3.685  22.255  -2.637  1.00  0.00           O
ATOM    426  N   TYR    26      -4.812  22.422  -4.574  1.00  0.00           N
ATOM    427  H   TYR    26      -5.022  23.109  -5.284  1.00  0.00           H
ATOM    428  CA  TYR    26      -5.093  21.014  -4.856  1.00  0.00           C
ATOM    429  HA  TYR    26      -4.284  20.501  -4.336  1.00  0.00           H
ATOM    430  CB  TYR    26      -6.501  20.658  -4.242  1.00  0.00           C
ATOM    431  HB2 TYR    26      -6.585  21.089  -3.245  1.00  0.00           H
ATOM    432  HB3 TYR    26      -7.246  21.092  -4.909  1.00  0.00           H
ATOM    433  CG  TYR    26      -6.744  19.147  -4.025  1.00  0.00           C
ATOM    434  CD1 TYR    26      -5.910  18.507  -3.075  1.00  0.00           C
ATOM    435  HD1 TYR    26      -5.168  19.070  -2.530  1.00  0.00           H
ATOM    436  CE1 TYR    26      -6.099  17.157  -2.774  1.00  0.00           C
ATOM    437  HE1 TYR    26      -5.536  16.566  -2.067  1.00  0.00           H
ATOM    438  CZ  TYR    26      -7.084  16.425  -3.530  1.00  0.00           C
ATOM    439  OH  TYR    26      -7.236  15.117  -3.242  1.00  0.00           O
ATOM    440  HH  TYR    26      -8.041  14.773  -3.639  1.00  0.00           H
ATOM    441  CE2 TYR    26      -7.905  17.034  -4.486  1.00  0.00           C
ATOM    442  HE2 TYR    26      -8.589  16.397  -5.028  1.00  0.00           H
ATOM    443  CD2 TYR    26      -7.707  18.418  -4.734  1.00  0.00           C
ATOM    444  HD2 TYR    26      -8.346  18.883  -5.469  1.00  0.00           H
ATOM    445  C   TYR    26      -4.790  20.751  -6.362  1.00  0.00           C
ATOM    446  O   TYR    26      -4.352  21.621  -7.105  1.00  0.00           O
ATOM    447  N   NME    27      -5.048  19.488  -6.728  1.00  0.00           N
ATOM    448  H   NME    27      -5.509  18.870  -6.076  1.00  0.00           H
ATOM    449  CH3 NME    27      -4.708  18.886  -7.965  1.00  0.00           C
ATOM    450 HH31 NME    27      -4.918  17.818  -7.892  1.00  0.00           H
ATOM    451 HH32 NME    27      -5.328  19.297  -8.762  1.00  0.00           H
ATOM    452 HH33 NME    27      -3.648  18.918  -8.223  1.00  0.00           H
TER     453      NME    27
END
```

Cluster 5:

```
ATOM      1 HH31 ACE     1      12.114   6.686  -0.015  1.00  0.00           H
ATOM      2  CH3 ACE     1      11.595   6.567  -0.967  1.00  0.00           C
ATOM      3 HH32 ACE     1      10.613   6.147  -0.752  1.00  0.00           H
ATOM      4 HH33 ACE     1      12.121   5.749  -1.461  1.00  0.00           H
ATOM      5  C   ACE     1      11.605   7.834  -1.897  1.00  0.00           C
ATOM      6  O   ACE     1      12.405   8.735  -1.639  1.00  0.00           O
ATOM      7  N   VAL     2      10.835   7.959  -2.977  1.00  0.00           N
ATOM      8  H   VAL     2      10.052   7.329  -3.072  1.00  0.00           H
ATOM      9  CA  VAL     2      11.021   8.997  -3.985  1.00  0.00           C
ATOM     10  HA  VAL     2      11.949   9.546  -3.825  1.00  0.00           H
ATOM     11  CB  VAL     2      11.133   8.458  -5.428  1.00  0.00           C
ATOM     12  HB  VAL     2      11.179   9.265  -6.160  1.00  0.00           H
ATOM     13  CG1 VAL     2      12.435   7.671  -5.601  1.00  0.00           C
ATOM     14 HG11 VAL     2      13.273   8.317  -5.340  1.00  0.00           H
ATOM     15 HG12 VAL     2      12.310   6.759  -5.017  1.00  0.00           H
ATOM     16 HG13 VAL     2      12.580   7.545  -6.674  1.00  0.00           H
ATOM     17  CG2 VAL     2      10.019   7.533  -5.714  1.00  0.00           C
ATOM     18 HG21 VAL     2       9.944   6.657  -5.069  1.00  0.00           H
```

```
ATOM     19 HG22 VAL     2       9.082   8.079  -5.614  1.00  0.00           H
ATOM     20 HG23 VAL     2      10.080   7.162  -6.737  1.00  0.00           H
ATOM     21  C   VAL     2       9.960  10.135  -3.935  1.00  0.00           C
ATOM     22  O   VAL     2      10.070  11.205  -4.514  1.00  0.00           O
ATOM     23  N   MET     3       9.036  10.030  -2.920  1.00  0.00           N
ATOM     24  H   MET     3       9.141   9.329  -2.201  1.00  0.00           H
ATOM     25  CA  MET     3       7.885  10.939  -2.759  1.00  0.00           C
ATOM     26  HA  MET     3       8.124  11.894  -3.229  1.00  0.00           H
ATOM     27  CB  MET     3       6.578  10.368  -3.327  1.00  0.00           C
ATOM     28  HB2 MET     3       6.497   9.481  -2.700  1.00  0.00           H
ATOM     29  HB3 MET     3       5.814  11.141  -3.243  1.00  0.00           H
ATOM     30  CG  MET     3       6.629   9.910  -4.821  1.00  0.00           C
ATOM     31  HG2 MET     3       6.306  10.722  -5.472  1.00  0.00           H
ATOM     32  HG3 MET     3       7.641   9.744  -5.191  1.00  0.00           H
ATOM     33  SD  MET     3       5.565   8.417  -5.240  1.00  0.00           S
ATOM     34  CE  MET     3       3.932   8.888  -4.657  1.00  0.00           C
ATOM     35  HE1 MET     3       3.629   9.834  -5.107  1.00  0.00           H
ATOM     36  HE2 MET     3       3.240   8.093  -4.932  1.00  0.00           H
ATOM     37  HE3 MET     3       3.954   9.111  -3.590  1.00  0.00           H
ATOM     38  C   MET     3       7.734  11.193  -1.284  1.00  0.00           C
ATOM     39  O   MET     3       8.079  10.412  -0.441  1.00  0.00           O
ATOM     40  N   SER     4       6.998  12.285  -0.974  1.00  0.00           N
ATOM     41  H   SER     4       6.570  12.825  -1.712  1.00  0.00           H
ATOM     42  CA  SER     4       6.968  12.830   0.412  1.00  0.00           C
ATOM     43  HA  SER     4       7.802  12.367   0.938  1.00  0.00           H
ATOM     44  CB  SER     4       7.240  14.373   0.426  1.00  0.00           C
ATOM     45  HB2 SER     4       6.419  14.797  -0.150  1.00  0.00           H
ATOM     46  HB3 SER     4       7.195  14.776   1.439  1.00  0.00           H
ATOM     47  OG  SER     4       8.528  14.695  -0.118  1.00  0.00           O
ATOM     48  HG  SER     4       8.652  15.626   0.082  1.00  0.00           H
ATOM     49  C   SER     4       5.732  12.429   1.108  1.00  0.00           C
ATOM     50  O   SER     4       5.664  12.790   2.261  1.00  0.00           O
ATOM     51  N   ARG     5       4.783  11.626   0.567  1.00  0.00           N
ATOM     52  H   ARG     5       5.010  11.369  -0.382  1.00  0.00           H
ATOM     53  CA  ARG     5       3.566  11.085   1.241  1.00  0.00           C
ATOM     54  HA  ARG     5       3.563  11.268   2.316  1.00  0.00           H
ATOM     55  CB  ARG     5       2.308  11.896   0.780  1.00  0.00           C
ATOM     56  HB2 ARG     5       1.436  11.785   1.424  1.00  0.00           H
ATOM     57  HB3 ARG     5       2.699  12.908   0.676  1.00  0.00           H
ATOM     58  CG  ARG     5       1.766  11.612  -0.626  1.00  0.00           C
ATOM     59  HG2 ARG     5       2.354  12.107  -1.398  1.00  0.00           H
ATOM     60  HG3 ARG     5       1.924  10.537  -0.707  1.00  0.00           H
ATOM     61  CD  ARG     5       0.268  11.998  -0.781  1.00  0.00           C
ATOM     62  HD2 ARG     5       0.212  13.050  -0.500  1.00  0.00           H
ATOM     63  HD3 ARG     5      -0.051  12.001  -1.823  1.00  0.00           H
ATOM     64  NE  ARG     5      -0.708  11.200   0.076  1.00  0.00           N
ATOM     65  HE  ARG     5      -0.551  10.221   0.267  1.00  0.00           H
ATOM     66  CZ  ARG     5      -1.906  11.614   0.541  1.00  0.00           C
ATOM     67  NH1 ARG     5      -2.385  12.721   0.239  1.00  0.00           N
ATOM     68 HH11 ARG     5      -1.805  13.345  -0.302  1.00  0.00           H
ATOM     69 HH12 ARG     5      -3.328  13.002   0.470  1.00  0.00           H
ATOM     70  NH2 ARG     5      -2.686  10.853   1.254  1.00  0.00           N
ATOM     71 HH21 ARG     5      -2.531   9.881   1.477  1.00  0.00           H
ATOM     72 HH22 ARG     5      -3.517  11.300   1.614  1.00  0.00           H
ATOM     73  C   ARG     5       3.454   9.577   1.069  1.00  0.00           C
ATOM     74  O   ARG     5       4.092   9.009   0.242  1.00  0.00           O
ATOM     75  N   PRO     6       2.586   8.885   1.816  1.00  0.00           N
ATOM     76  CD  PRO     6       1.966   9.266   3.064  1.00  0.00           C
ATOM     77  HD2 PRO     6       1.407  10.199   2.994  1.00  0.00           H
ATOM     78  HD3 PRO     6       2.748   9.401   3.811  1.00  0.00           H
ATOM     79  CG  PRO     6       1.015   8.139   3.459  1.00  0.00           C
ATOM     80  HG2 PRO     6       0.083   8.267   2.910  1.00  0.00           H
ATOM     81  HG3 PRO     6       0.886   8.127   4.542  1.00  0.00           H
```

ATOM	82	CB	PRO	6	1.730	6.903	2.906	1.00	0.00	C
ATOM	83	HB2	PRO	6	0.922	6.202	2.700	1.00	0.00	H
ATOM	84	HB3	PRO	6	2.537	6.454	3.485	1.00	0.00	H
ATOM	85	CA	PRO	6	2.305	7.472	1.577	1.00	0.00	C
ATOM	86	HA	PRO	6	3.238	6.926	1.436	1.00	0.00	H
ATOM	87	C	PRO	6	1.337	7.278	0.395	1.00	0.00	C
ATOM	88	O	PRO	6	0.774	8.232	-0.164	1.00	0.00	O
ATOM	89	N	LEU	7	1.240	6.025	-0.060	1.00	0.00	N
ATOM	90	H	LEU	7	1.735	5.313	0.457	1.00	0.00	H
ATOM	91	CA	LEU	7	0.273	5.541	-1.147	1.00	0.00	C
ATOM	92	HA	LEU	7	0.556	6.148	-2.006	1.00	0.00	H
ATOM	93	CB	LEU	7	0.576	4.035	-1.337	1.00	0.00	C
ATOM	94	HB2	LEU	7	1.632	3.766	-1.321	1.00	0.00	H
ATOM	95	HB3	LEU	7	0.312	3.538	-0.404	1.00	0.00	H
ATOM	96	CG	LEU	7	-0.172	3.235	-2.424	1.00	0.00	C
ATOM	97	HG	LEU	7	-1.253	3.318	-2.307	1.00	0.00	H
ATOM	98	CD1	LEU	7	0.336	3.846	-3.739	1.00	0.00	C
ATOM	99	HD11	LEU	7	0.246	3.214	-4.623	1.00	0.00	H
ATOM	100	HD12	LEU	7	-0.328	4.670	-3.997	1.00	0.00	H
ATOM	101	HD13	LEU	7	1.316	4.268	-3.513	1.00	0.00	H
ATOM	102	CD2	LEU	7	0.317	1.847	-2.486	1.00	0.00	C
ATOM	103	HD21	LEU	7	1.396	1.734	-2.380	1.00	0.00	H
ATOM	104	HD22	LEU	7	0.065	1.367	-1.541	1.00	0.00	H
ATOM	105	HD23	LEU	7	-0.091	1.212	-3.272	1.00	0.00	H
ATOM	106	C	LEU	7	-1.161	5.735	-0.841	1.00	0.00	C
ATOM	107	O	LEU	7	-1.972	5.929	-1.746	1.00	0.00	O
ATOM	108	N	ILE	8	-1.511	5.718	0.470	1.00	0.00	N
ATOM	109	H	ILE	8	-0.783	5.716	1.171	1.00	0.00	H
ATOM	110	CA	ILE	8	-2.909	5.682	0.876	1.00	0.00	C
ATOM	111	HA	ILE	8	-3.521	5.465	0.001	1.00	0.00	H
ATOM	112	CB	ILE	8	-3.347	4.515	1.901	1.00	0.00	C
ATOM	113	HB	ILE	8	-4.386	4.642	2.207	1.00	0.00	H
ATOM	114	CG2	ILE	8	-3.057	3.176	1.164	1.00	0.00	C
ATOM	115	HG21	ILE	8	-3.358	3.149	0.117	1.00	0.00	H
ATOM	116	HG22	ILE	8	-2.060	2.739	1.233	1.00	0.00	H
ATOM	117	HG23	ILE	8	-3.633	2.385	1.646	1.00	0.00	H
ATOM	118	CG1	ILE	8	-2.609	4.386	3.215	1.00	0.00	C
ATOM	119	HG12	ILE	8	-2.891	3.405	3.598	1.00	0.00	H
ATOM	120	HG13	ILE	8	-1.537	4.498	3.050	1.00	0.00	H
ATOM	121	CD1	ILE	8	-2.927	5.495	4.235	1.00	0.00	C
ATOM	122	HD11	ILE	8	-3.974	5.506	4.536	1.00	0.00	H
ATOM	123	HD12	ILE	8	-2.328	5.307	5.126	1.00	0.00	H
ATOM	124	HD13	ILE	8	-2.665	6.443	3.765	1.00	0.00	H
ATOM	125	C	ILE	8	-3.392	7.117	1.177	1.00	0.00	C
ATOM	126	O	ILE	8	-2.528	8.006	1.298	1.00	0.00	O
ATOM	127	N	HID	9	-4.711	7.293	1.320	1.00	0.00	N
ATOM	128	H	HID	9	-5.418	6.586	1.464	1.00	0.00	H
ATOM	129	CA	HID	9	-5.310	8.643	1.445	1.00	0.00	C
ATOM	130	HA	HID	9	-4.741	9.291	2.111	1.00	0.00	H
ATOM	131	CB	HID	9	-5.553	9.304	0.044	1.00	0.00	C
ATOM	132	HB2	HID	9	-5.990	10.273	0.285	1.00	0.00	H
ATOM	133	HB3	HID	9	-4.621	9.556	-0.460	1.00	0.00	H
ATOM	134	CG	HID	9	-6.585	8.575	-0.863	1.00	0.00	C
ATOM	135	ND1	HID	9	-7.870	9.039	-1.130	1.00	0.00	N
ATOM	136	HD1	HID	9	-8.411	9.716	-0.613	1.00	0.00	H
ATOM	137	CE1	HID	9	-8.567	8.083	-1.784	1.00	0.00	C
ATOM	138	HE1	HID	9	-9.576	8.161	-2.160	1.00	0.00	H
ATOM	139	NE2	HID	9	-7.721	7.038	-2.052	1.00	0.00	N
ATOM	140	CD2	HID	9	-6.539	7.330	-1.464	1.00	0.00	C
ATOM	141	HD2	HID	9	-5.836	6.511	-1.440	1.00	0.00	H
ATOM	142	C	HID	9	-6.707	8.531	2.067	1.00	0.00	C
ATOM	143	O	HID	9	-7.178	7.411	2.314	1.00	0.00	O
ATOM	144	N	PHE	10	-7.363	9.679	2.421	1.00	0.00	N

ATOM	145	H	PHE	10	-6.921	10.568	2.236	1.00	0.00	H
ATOM	146	CA	PHE	10	-8.755	9.765	3.001	1.00	0.00	C
ATOM	147	HA	PHE	10	-9.041	8.743	3.250	1.00	0.00	H
ATOM	148	CB	PHE	10	-8.679	10.440	4.353	1.00	0.00	C
ATOM	149	HB2	PHE	10	-8.540	11.514	4.234	1.00	0.00	H
ATOM	150	HB3	PHE	10	-9.648	10.299	4.833	1.00	0.00	H
ATOM	151	CG	PHE	10	-7.622	9.917	5.351	1.00	0.00	C
ATOM	152	CD1	PHE	10	-7.074	10.728	6.366	1.00	0.00	C
ATOM	153	HD1	PHE	10	-7.250	11.792	6.306	1.00	0.00	H
ATOM	154	CE1	PHE	10	-6.034	10.211	7.156	1.00	0.00	C
ATOM	155	HE1	PHE	10	-5.608	10.879	7.889	1.00	0.00	H
ATOM	156	CZ	PHE	10	-5.859	8.808	7.291	1.00	0.00	C
ATOM	157	HZ	PHE	10	-5.159	8.469	8.040	1.00	0.00	H
ATOM	158	CE2	PHE	10	-6.434	8.053	6.228	1.00	0.00	C
ATOM	159	HE2	PHE	10	-6.102	7.038	6.059	1.00	0.00	H
ATOM	160	CD2	PHE	10	-7.314	8.551	5.323	1.00	0.00	C
ATOM	161	HD2	PHE	10	-7.697	7.815	4.632	1.00	0.00	H
ATOM	162	C	PHE	10	-9.921	10.120	2.097	1.00	0.00	C
ATOM	163	O	PHE	10	-9.679	10.505	0.955	1.00	0.00	O
ATOM	164	N	GLY	11	-11.164	9.938	2.561	1.00	0.00	N
ATOM	165	H	GLY	11	-11.244	9.660	3.529	1.00	0.00	H
ATOM	166	CA	GLY	11	-12.406	10.148	1.782	1.00	0.00	C
ATOM	167	HA2	GLY	11	-12.329	9.758	0.768	1.00	0.00	H
ATOM	168	HA3	GLY	11	-13.274	9.732	2.294	1.00	0.00	H
ATOM	169	C	GLY	11	-12.740	11.669	1.611	1.00	0.00	C
ATOM	170	O	GLY	11	-12.819	12.152	0.493	1.00	0.00	O
ATOM	171	N	ASN	12	-12.868	12.433	2.646	1.00	0.00	N
ATOM	172	H	ASN	12	-12.741	12.089	3.586	1.00	0.00	H
ATOM	173	CA	ASN	12	-13.082	13.877	2.509	1.00	0.00	C
ATOM	174	HA	ASN	12	-13.760	14.039	1.672	1.00	0.00	H
ATOM	175	CB	ASN	12	-13.628	14.552	3.822	1.00	0.00	C
ATOM	176	HB2	ASN	12	-14.487	13.936	4.086	1.00	0.00	H
ATOM	177	HB3	ASN	12	-12.922	14.556	4.652	1.00	0.00	H
ATOM	178	CG	ASN	12	-14.186	15.978	3.733	1.00	0.00	C
ATOM	179	OD1	ASN	12	-13.616	16.779	3.017	1.00	0.00	O
ATOM	180	ND2	ASN	12	-15.131	16.365	4.605	1.00	0.00	N
ATOM	181	HD21	ASN	12	-15.608	17.247	4.488	1.00	0.00	H
ATOM	182	HD22	ASN	12	-15.416	15.712	5.320	1.00	0.00	H
ATOM	183	C	ASN	12	-11.745	14.589	2.119	1.00	0.00	C
ATOM	184	O	ASN	12	-10.729	14.432	2.879	1.00	0.00	O
ATOM	185	N	ASP	13	-11.780	15.458	1.095	1.00	0.00	N
ATOM	186	H	ASP	13	-12.705	15.683	0.760	1.00	0.00	H
ATOM	187	CA	ASP	13	-10.578	16.146	0.577	1.00	0.00	C
ATOM	188	HA	ASP	13	-9.803	15.459	0.236	1.00	0.00	H
ATOM	189	CB	ASP	13	-11.045	16.979	-0.634	1.00	0.00	C
ATOM	190	HB2	ASP	13	-11.359	16.314	-1.439	1.00	0.00	H
ATOM	191	HB3	ASP	13	-11.902	17.586	-0.344	1.00	0.00	H
ATOM	192	CG	ASP	13	-9.833	17.767	-1.202	1.00	0.00	C
ATOM	193	OD1	ASP	13	-9.907	18.999	-1.200	1.00	0.00	O
ATOM	194	OD2	ASP	13	-8.748	17.263	-1.652	1.00	0.00	O
ATOM	195	C	ASP	13	-10.019	17.100	1.635	1.00	0.00	C
ATOM	196	O	ASP	13	-8.752	17.120	1.752	1.00	0.00	O
ATOM	197	N	TYR	14	-10.814	17.633	2.542	1.00	0.00	N
ATOM	198	H	TYR	14	-11.812	17.471	2.531	1.00	0.00	H
ATOM	199	CA	TYR	14	-10.323	18.571	3.611	1.00	0.00	C
ATOM	200	HA	TYR	14	-9.547	19.219	3.203	1.00	0.00	H
ATOM	201	CB	TYR	14	-11.458	19.521	4.078	1.00	0.00	C
ATOM	202	HB2	TYR	14	-11.598	20.218	3.252	1.00	0.00	H
ATOM	203	HB3	TYR	14	-12.414	19.013	4.211	1.00	0.00	H
ATOM	204	CG	TYR	14	-11.149	20.384	5.246	1.00	0.00	C
ATOM	205	CD1	TYR	14	-10.207	21.445	5.083	1.00	0.00	C
ATOM	206	HD1	TYR	14	-9.827	21.708	4.108	1.00	0.00	H
ATOM	207	CE1	TYR	14	-9.828	22.277	6.104	1.00	0.00	C

ATOM	208	HE1	TYR	14	-9.233	23.159	5.918	1.00	0.00	H
ATOM	209	CZ	TYR	14	-10.277	21.887	7.404	1.00	0.00	C
ATOM	210	OH	TYR	14	-10.027	22.739	8.453	1.00	0.00	O
ATOM	211	HH	TYR	14	-9.511	23.520	8.242	1.00	0.00	H
ATOM	212	CE2	TYR	14	-11.187	20.829	7.620	1.00	0.00	C
ATOM	213	HE2	TYR	14	-11.567	20.649	8.615	1.00	0.00	H
ATOM	214	CD2	TYR	14	-11.611	20.053	6.542	1.00	0.00	C
ATOM	215	HD2	TYR	14	-12.288	19.212	6.577	1.00	0.00	H
ATOM	216	C	TYR	14	-9.621	17.804	4.746	1.00	0.00	C
ATOM	217	O	TYR	14	-8.699	18.246	5.456	1.00	0.00	O
ATOM	218	N	GLU	15	-10.000	16.491	4.866	1.00	0.00	N
ATOM	219	H	GLU	15	-10.504	16.090	4.088	1.00	0.00	H
ATOM	220	CA	GLU	15	-9.408	15.586	5.821	1.00	0.00	C
ATOM	221	HA	GLU	15	-9.066	16.203	6.651	1.00	0.00	H
ATOM	222	CB	GLU	15	-10.426	14.683	6.379	1.00	0.00	C
ATOM	223	HB2	GLU	15	-10.984	14.157	5.603	1.00	0.00	H
ATOM	224	HB3	GLU	15	-9.923	13.959	7.019	1.00	0.00	H
ATOM	225	CG	GLU	15	-11.349	15.507	7.296	1.00	0.00	C
ATOM	226	HG2	GLU	15	-11.760	16.365	6.763	1.00	0.00	H
ATOM	227	HG3	GLU	15	-12.153	14.802	7.502	1.00	0.00	H
ATOM	228	CD	GLU	15	-10.709	16.024	8.554	1.00	0.00	C
ATOM	229	OE1	GLU	15	-10.687	17.260	8.886	1.00	0.00	O
ATOM	230	OE2	GLU	15	-10.413	15.046	9.336	1.00	0.00	O
ATOM	231	C	GLU	15	-8.148	14.898	5.453	1.00	0.00	C
ATOM	232	O	GLU	15	-7.461	14.304	6.332	1.00	0.00	O
ATOM	233	N	ASP	16	-7.769	15.050	4.159	1.00	0.00	N
ATOM	234	H	ASP	16	-8.467	15.412	3.526	1.00	0.00	H
ATOM	235	CA	ASP	16	-6.490	14.462	3.638	1.00	0.00	C
ATOM	236	HA	ASP	16	-6.151	13.643	4.274	1.00	0.00	H
ATOM	237	CB	ASP	16	-6.738	14.068	2.192	1.00	0.00	C
ATOM	238	HB2	ASP	16	-7.527	13.316	2.154	1.00	0.00	H
ATOM	239	HB3	ASP	16	-7.212	14.921	1.706	1.00	0.00	H
ATOM	240	CG	ASP	16	-5.540	13.475	1.429	1.00	0.00	C
ATOM	241	OD1	ASP	16	-4.916	14.114	0.536	1.00	0.00	O
ATOM	242	OD2	ASP	16	-5.127	12.336	1.781	1.00	0.00	O
ATOM	243	C	ASP	16	-5.315	15.483	3.813	1.00	0.00	C
ATOM	244	O	ASP	16	-4.234	15.195	3.314	1.00	0.00	O
ATOM	245	N	ARG	17	-5.458	16.603	4.590	1.00	0.00	N
ATOM	246	H	ARG	17	-6.374	16.778	4.977	1.00	0.00	H
ATOM	247	CA	ARG	17	-4.396	17.627	4.760	1.00	0.00	C
ATOM	248	HA	ARG	17	-3.968	17.770	3.769	1.00	0.00	H
ATOM	249	CB	ARG	17	-4.983	19.005	5.046	1.00	0.00	C
ATOM	250	HB2	ARG	17	-4.144	19.701	5.073	1.00	0.00	H
ATOM	251	HB3	ARG	17	-5.583	19.258	4.172	1.00	0.00	H
ATOM	252	CG	ARG	17	-5.792	19.090	6.347	1.00	0.00	C
ATOM	253	HG2	ARG	17	-6.537	18.294	6.385	1.00	0.00	H
ATOM	254	HG3	ARG	17	-5.058	19.022	7.149	1.00	0.00	H
ATOM	255	CD	ARG	17	-6.586	20.354	6.639	1.00	0.00	C
ATOM	256	HD2	ARG	17	-5.930	21.180	6.364	1.00	0.00	H
ATOM	257	HD3	ARG	17	-7.423	20.456	5.947	1.00	0.00	H
ATOM	258	NE	ARG	17	-7.000	20.447	8.055	1.00	0.00	N
ATOM	259	HE	ARG	17	-6.512	21.185	8.540	1.00	0.00	H
ATOM	260	CZ	ARG	17	-7.890	19.738	8.722	1.00	0.00	C
ATOM	261	NH1	ARG	17	-8.525	18.808	8.057	1.00	0.00	N
ATOM	262	HH11	ARG	17	-8.414	18.852	7.055	1.00	0.00	H
ATOM	263	HH12	ARG	17	-9.139	18.195	8.575	1.00	0.00	H
ATOM	264	NH2	ARG	17	-8.116	19.894	9.949	1.00	0.00	N
ATOM	265	HH21	ARG	17	-7.406	20.340	10.512	1.00	0.00	H
ATOM	266	HH22	ARG	17	-8.895	19.459	10.422	1.00	0.00	H
ATOM	267	C	ARG	17	-3.254	17.241	5.657	1.00	0.00	C
ATOM	268	O	ARG	17	-2.270	17.964	5.690	1.00	0.00	O
ATOM	269	N	TYR	18	-3.347	16.045	6.306	1.00	0.00	N
ATOM	270	H	TYR	18	-4.248	15.590	6.254	1.00	0.00	H

ATOM	271	CA	TYR	18	-2.312	15.472	7.196	1.00	0.00	C
ATOM	272	HA	TYR	18	-1.968	16.286	7.835	1.00	0.00	H
ATOM	273	CB	TYR	18	-2.910	14.476	8.212	1.00	0.00	C
ATOM	274	HB2	TYR	18	-3.084	13.529	7.702	1.00	0.00	H
ATOM	275	HB3	TYR	18	-2.119	14.227	8.920	1.00	0.00	H
ATOM	276	CG	TYR	18	-4.059	15.067	9.038	1.00	0.00	C
ATOM	277	CD1	TYR	18	-4.012	16.358	9.592	1.00	0.00	C
ATOM	278	HD1	TYR	18	-3.033	16.810	9.618	1.00	0.00	H
ATOM	279	CE1	TYR	18	-5.141	16.932	10.280	1.00	0.00	C
ATOM	280	HE1	TYR	18	-5.036	17.903	10.740	1.00	0.00	H
ATOM	281	CZ	TYR	18	-6.325	16.264	10.389	1.00	0.00	C
ATOM	282	OH	TYR	18	-7.416	16.868	10.918	1.00	0.00	O
ATOM	283	HH	TYR	18	-8.114	16.210	10.911	1.00	0.00	H
ATOM	284	CE2	TYR	18	-6.409	14.963	9.781	1.00	0.00	C
ATOM	285	HE2	TYR	18	-7.362	14.456	9.779	1.00	0.00	H
ATOM	286	CD2	TYR	18	-5.229	14.358	9.256	1.00	0.00	C
ATOM	287	HD2	TYR	18	-5.337	13.346	8.894	1.00	0.00	H
ATOM	288	C	TYR	18	-1.039	14.945	6.551	1.00	0.00	C
ATOM	289	O	TYR	18	-0.043	14.795	7.252	1.00	0.00	O
ATOM	290	N	TYR	19	-1.124	14.709	5.226	1.00	0.00	N
ATOM	291	H	TYR	19	-2.066	14.589	4.881	1.00	0.00	H
ATOM	292	CA	TYR	19	-0.174	14.150	4.277	1.00	0.00	C
ATOM	293	HA	TYR	19	0.733	13.923	4.838	1.00	0.00	H
ATOM	294	CB	TYR	19	-0.681	12.918	3.666	1.00	0.00	C
ATOM	295	HB2	TYR	19	-1.530	13.063	2.996	1.00	0.00	H
ATOM	296	HB3	TYR	19	0.170	12.433	3.186	1.00	0.00	H
ATOM	297	CG	TYR	19	-1.327	11.964	4.707	1.00	0.00	C
ATOM	298	CD1	TYR	19	-2.688	11.620	4.715	1.00	0.00	C
ATOM	299	HD1	TYR	19	-3.388	12.239	4.174	1.00	0.00	H
ATOM	300	CE1	TYR	19	-3.048	10.426	5.352	1.00	0.00	C
ATOM	301	HE1	TYR	19	-4.073	10.094	5.425	1.00	0.00	H
ATOM	302	CZ	TYR	19	-2.108	9.613	6.007	1.00	0.00	C
ATOM	303	OH	TYR	19	-2.495	8.479	6.681	1.00	0.00	O
ATOM	304	HH	TYR	19	-3.360	8.149	6.429	1.00	0.00	H
ATOM	305	CE2	TYR	19	-0.730	10.005	6.069	1.00	0.00	C
ATOM	306	HE2	TYR	19	-0.115	9.455	6.767	1.00	0.00	H
ATOM	307	CD2	TYR	19	-0.403	11.233	5.425	1.00	0.00	C
ATOM	308	HD2	TYR	19	0.654	11.451	5.436	1.00	0.00	H
ATOM	309	C	TYR	19	0.271	15.249	3.310	1.00	0.00	C
ATOM	310	O	TYR	19	0.967	14.889	2.351	1.00	0.00	O
ATOM	311	N	ARG	20	-0.076	16.552	3.534	1.00	0.00	N
ATOM	312	H	ARG	20	-0.545	16.755	4.405	1.00	0.00	H
ATOM	313	CA	ARG	20	0.086	17.602	2.568	1.00	0.00	C
ATOM	314	HA	ARG	20	0.706	17.247	1.745	1.00	0.00	H
ATOM	315	CB	ARG	20	-1.216	18.176	2.040	1.00	0.00	C
ATOM	316	HB2	ARG	20	-1.738	18.721	2.827	1.00	0.00	H
ATOM	317	HB3	ARG	20	-0.906	18.946	1.333	1.00	0.00	H
ATOM	318	CG	ARG	20	-2.241	17.195	1.447	1.00	0.00	C
ATOM	319	HG2	ARG	20	-1.821	16.547	0.677	1.00	0.00	H
ATOM	320	HG3	ARG	20	-2.490	16.552	2.291	1.00	0.00	H
ATOM	321	CD	ARG	20	-3.456	17.885	0.793	1.00	0.00	C
ATOM	322	HD2	ARG	20	-4.050	18.392	1.554	1.00	0.00	H
ATOM	323	HD3	ARG	20	-3.117	18.674	0.121	1.00	0.00	H
ATOM	324	NE	ARG	20	-4.329	16.950	0.085	1.00	0.00	N
ATOM	325	HE	ARG	20	-4.218	15.968	0.291	1.00	0.00	H
ATOM	326	CZ	ARG	20	-5.366	17.282	-0.736	1.00	0.00	C
ATOM	327	NH1	ARG	20	-5.817	18.483	-0.881	1.00	0.00	N
ATOM	328	HH11	ARG	20	-5.317	19.243	-0.442	1.00	0.00	H
ATOM	329	HH12	ARG	20	-6.779	18.567	-1.175	1.00	0.00	H
ATOM	330	NH2	ARG	20	-6.050	16.350	-1.343	1.00	0.00	N
ATOM	331	HH21	ARG	20	-5.702	15.431	-1.109	1.00	0.00	H
ATOM	332	HH22	ARG	20	-7.022	16.476	-1.585	1.00	0.00	H
ATOM	333	C	ARG	20	0.945	18.765	3.139	1.00	0.00	C

ATOM	334	O	ARG	20	1.215	18.804	4.305	1.00	0.00	O
ATOM	335	N	GLU	21	1.246	19.720	2.229	1.00	0.00	N
ATOM	336	H	GLU	21	1.029	19.414	1.291	1.00	0.00	H
ATOM	337	CA	GLU	21	1.970	20.943	2.398	1.00	0.00	C
ATOM	338	HA	GLU	21	2.453	20.945	3.375	1.00	0.00	H
ATOM	339	CB	GLU	21	3.014	21.132	1.244	1.00	0.00	C
ATOM	340	HB2	GLU	21	3.754	20.380	1.520	1.00	0.00	H
ATOM	341	HB3	GLU	21	2.562	20.986	0.262	1.00	0.00	H
ATOM	342	CG	GLU	21	3.621	22.522	1.250	1.00	0.00	C
ATOM	343	HG2	GLU	21	2.983	23.195	0.676	1.00	0.00	H
ATOM	344	HG3	GLU	21	3.857	22.811	2.274	1.00	0.00	H
ATOM	345	CD	GLU	21	4.897	22.591	0.373	1.00	0.00	C
ATOM	346	OE1	GLU	21	5.509	23.629	0.408	1.00	0.00	O
ATOM	347	OE2	GLU	21	5.308	21.603	-0.296	1.00	0.00	O
ATOM	348	C	GLU	21	0.848	22.086	2.393	1.00	0.00	C
ATOM	349	O	GLU	21	-0.118	21.910	1.610	1.00	0.00	O
ATOM	350	N	ASN	22	1.101	23.184	3.125	1.00	0.00	N
ATOM	351	H	ASN	22	1.979	23.209	3.623	1.00	0.00	H
ATOM	352	CA	ASN	22	0.152	24.278	3.146	1.00	0.00	C
ATOM	353	HA	ASN	22	-0.824	23.925	3.480	1.00	0.00	H
ATOM	354	CB	ASN	22	0.516	25.195	4.424	1.00	0.00	C
ATOM	355	HB2	ASN	22	0.309	24.574	5.296	1.00	0.00	H
ATOM	356	HB3	ASN	22	1.521	25.618	4.439	1.00	0.00	H
ATOM	357	CG	ASN	22	-0.523	26.362	4.580	1.00	0.00	C
ATOM	358	OD1	ASN	22	-1.700	26.190	4.245	1.00	0.00	O
ATOM	359	ND2	ASN	22	-0.218	27.537	5.080	1.00	0.00	N
ATOM	360	HD21	ASN	22	-0.983	28.188	5.193	1.00	0.00	H
ATOM	361	HD22	ASN	22	0.730	27.789	5.318	1.00	0.00	H
ATOM	362	C	ASN	22	0.204	25.079	1.877	1.00	0.00	C
ATOM	363	O	ASN	22	1.221	25.794	1.658	1.00	0.00	O
ATOM	364	N	MET	23	-0.840	25.074	1.011	1.00	0.00	N
ATOM	365	H	MET	23	-1.686	24.602	1.299	1.00	0.00	H
ATOM	366	CA	MET	23	-0.779	25.712	-0.282	1.00	0.00	C
ATOM	367	HA	MET	23	0.252	25.888	-0.587	1.00	0.00	H
ATOM	368	CB	MET	23	-1.277	24.718	-1.374	1.00	0.00	C
ATOM	369	HB2	MET	23	-2.358	24.597	-1.319	1.00	0.00	H
ATOM	370	HB3	MET	23	-1.141	25.231	-2.326	1.00	0.00	H
ATOM	371	CG	MET	23	-0.504	23.398	-1.399	1.00	0.00	C
ATOM	372	HG2	MET	23	0.505	23.696	-1.115	1.00	0.00	H
ATOM	373	HG3	MET	23	-0.769	22.770	-0.549	1.00	0.00	H
ATOM	374	SD	MET	23	-0.435	22.377	-2.881	1.00	0.00	S
ATOM	375	CE	MET	23	1.351	22.251	-3.095	1.00	0.00	C
ATOM	376	HE1	MET	23	1.834	21.802	-2.227	1.00	0.00	H
ATOM	377	HE2	MET	23	1.531	21.757	-4.049	1.00	0.00	H
ATOM	378	HE3	MET	23	1.809	23.240	-3.110	1.00	0.00	H
ATOM	379	C	MET	23	-1.504	27.029	-0.387	1.00	0.00	C
ATOM	380	O	MET	23	-1.709	27.554	-1.487	1.00	0.00	O
ATOM	381	N	TYR	24	-1.816	27.617	0.733	1.00	0.00	N
ATOM	382	H	TYR	24	-1.400	27.213	1.560	1.00	0.00	H
ATOM	383	CA	TYR	24	-2.698	28.760	0.984	1.00	0.00	C
ATOM	384	HA	TYR	24	-2.919	29.087	-0.032	1.00	0.00	H
ATOM	385	CB	TYR	24	-4.039	28.305	1.620	1.00	0.00	C
ATOM	386	HB2	TYR	24	-3.867	27.918	2.626	1.00	0.00	H
ATOM	387	HB3	TYR	24	-4.745	29.132	1.550	1.00	0.00	H
ATOM	388	CG	TYR	24	-4.723	27.147	0.914	1.00	0.00	C
ATOM	389	CD1	TYR	24	-4.692	25.864	1.566	1.00	0.00	C
ATOM	390	HD1	TYR	24	-4.032	25.587	2.374	1.00	0.00	H
ATOM	391	CE1	TYR	24	-5.493	24.821	1.065	1.00	0.00	C
ATOM	392	HE1	TYR	24	-5.457	23.832	1.496	1.00	0.00	H
ATOM	393	CZ	TYR	24	-6.311	25.046	-0.087	1.00	0.00	C
ATOM	394	OH	TYR	24	-7.023	24.068	-0.677	1.00	0.00	O
ATOM	395	HH	TYR	24	-7.727	24.362	-1.259	1.00	0.00	H
ATOM	396	CE2	TYR	24	-6.519	26.321	-0.614	1.00	0.00	C

```
ATOM    397  HE2 TYR    24      -7.135  26.528  -1.476  1.00  0.00           H
ATOM    398  CD2 TYR    24      -5.739  27.338  -0.079  1.00  0.00           C
ATOM    399  HD2 TYR    24      -5.890  28.336  -0.462  1.00  0.00           H
ATOM    400  C   TYR    24      -2.027  29.891   1.770  1.00  0.00           C
ATOM    401  O   TYR    24      -2.729  30.582   2.470  1.00  0.00           O
ATOM    402  N   ARG    25      -0.730  30.123   1.535  1.00  0.00           N
ATOM    403  H   ARG    25      -0.317  29.721   0.704  1.00  0.00           H
ATOM    404  CA  ARG    25       0.152  31.050   2.261  1.00  0.00           C
ATOM    405  HA  ARG    25      -0.001  31.045   3.340  1.00  0.00           H
ATOM    406  CB  ARG    25       1.592  30.650   1.937  1.00  0.00           C
ATOM    407  HB2 ARG    25       1.641  30.653   0.848  1.00  0.00           H
ATOM    408  HB3 ARG    25       2.267  31.409   2.329  1.00  0.00           H
ATOM    409  CG  ARG    25       1.924  29.402   2.700  1.00  0.00           C
ATOM    410  HG2 ARG    25       2.145  29.615   3.746  1.00  0.00           H
ATOM    411  HG3 ARG    25       1.076  28.718   2.739  1.00  0.00           H
ATOM    412  CD  ARG    25       3.086  28.693   1.956  1.00  0.00           C
ATOM    413  HD2 ARG    25       2.591  28.270   1.082  1.00  0.00           H
ATOM    414  HD3 ARG    25       3.804  29.508   1.862  1.00  0.00           H
ATOM    415  NE  ARG    25       3.683  27.565   2.698  1.00  0.00           N
ATOM    416  HE  ARG    25       3.656  27.697   3.699  1.00  0.00           H
ATOM    417  CZ  ARG    25       4.214  26.449   2.218  1.00  0.00           C
ATOM    418  NH1 ARG    25       4.166  26.160   0.975  1.00  0.00           N
ATOM    419 HH11 ARG    25       3.672  26.811   0.381  1.00  0.00           H
ATOM    420 HH12 ARG    25       4.613  25.358   0.553  1.00  0.00           H
ATOM    421  NH2 ARG    25       4.801  25.546   2.970  1.00  0.00           N
ATOM    422 HH21 ARG    25       4.723  25.661   3.971  1.00  0.00           H
ATOM    423 HH22 ARG    25       5.243  24.747   2.538  1.00  0.00           H
ATOM    424  C   ARG    25      -0.117  32.524   1.872  1.00  0.00           C
ATOM    425  O   ARG    25      -0.631  32.806   0.792  1.00  0.00           O
ATOM    426  N   TYR    26       0.189  33.406   2.820  1.00  0.00           N
ATOM    427  H   TYR    26       0.576  32.964   3.642  1.00  0.00           H
ATOM    428  CA  TYR    26      -0.122  34.830   2.715  1.00  0.00           C
ATOM    429  HA  TYR    26      -0.276  34.977   1.646  1.00  0.00           H
ATOM    430  CB  TYR    26      -1.451  35.109   3.436  1.00  0.00           C
ATOM    431  HB2 TYR    26      -2.185  34.442   2.984  1.00  0.00           H
ATOM    432  HB3 TYR    26      -1.427  34.667   4.430  1.00  0.00           H
ATOM    433  CG  TYR    26      -1.903  36.549   3.369  1.00  0.00           C
ATOM    434  CD1 TYR    26      -2.457  37.059   2.168  1.00  0.00           C
ATOM    435  HD1 TYR    26      -2.610  36.403   1.323  1.00  0.00           H
ATOM    436  CE1 TYR    26      -2.869  38.355   2.042  1.00  0.00           C
ATOM    437  HE1 TYR    26      -3.324  38.704   1.126  1.00  0.00           H
ATOM    438  CZ  TYR    26      -2.737  39.150   3.186  1.00  0.00           C
ATOM    439  OH  TYR    26      -3.264  40.427   3.198  1.00  0.00           O
ATOM    440  HH  TYR    26      -3.762  40.560   2.387  1.00  0.00           H
ATOM    441  CE2 TYR    26      -2.124  38.682   4.300  1.00  0.00           C
ATOM    442  HE2 TYR    26      -1.922  39.418   5.064  1.00  0.00           H
ATOM    443  CD2 TYR    26      -1.827  37.335   4.491  1.00  0.00           C
ATOM    444  HD2 TYR    26      -1.479  36.927   5.428  1.00  0.00           H
ATOM    445  C   TYR    26       1.004  35.750   3.198  1.00  0.00           C
ATOM    446  O   TYR    26       2.003  35.303   3.724  1.00  0.00           O
ATOM    447  N   NME    27       0.953  36.995   2.793  1.00  0.00           N
ATOM    448  H   NME    27       0.143  37.336   2.293  1.00  0.00           H
ATOM    449  CH3 NME    27       1.981  37.973   3.150  1.00  0.00           C
ATOM    450 HH31 NME    27       1.619  38.966   2.885  1.00  0.00           H
ATOM    451 HH32 NME    27       2.122  37.966   4.230  1.00  0.00           H
ATOM    452 HH33 NME    27       2.982  37.808   2.753  1.00  0.00           H
TER     453      NME    27
END
```

Isolated H1 simulation:

#Cluster	Frames	Frac	AvgDist	Stdev	Centroid	AvgCDist
0	170823	0.833	3.483	0.970	203620	4.720
1	12981	0.063	3.122	0.000	51597	4.938
2	7560	0.037	3.276	1.000	166537	4.917
3	5933	0.029	3.383	0.662	174770	4.534
4	4977	0.024	3.247	0.827	125802	4.900
5	752	0.004	2.613	1.310	956	5.383
6	728	0.004	2.799	1.084	181681	4.515
7	715	0.003	3.575	0.000	188139	4.687
8	428	0.002	1.978	1.070	39264	4.578
9	99	0.000	0.000	0.000	199986	5.062
10	4	0.000	0.000	0.000	103764	5.052

Cluster 0:

ATOM	1	HH31	ACE	1	23.409	4.823	7.788	1.00	0.00	H
ATOM	2	CH3	ACE	1	23.168	4.865	8.850	1.00	0.00	C
ATOM	3	HH32	ACE	1	22.525	5.740	8.941	1.00	0.00	H
ATOM	4	HH33	ACE	1	24.014	5.061	9.508	1.00	0.00	H
ATOM	5	C	ACE	1	22.432	3.650	9.309	1.00	0.00	C
ATOM	6	O	ACE	1	22.949	2.503	9.294	1.00	0.00	O
ATOM	7	N	ASN	2	21.211	3.849	9.823	1.00	0.00	N
ATOM	8	H	ASN	2	20.771	4.757	9.780	1.00	0.00	H
ATOM	9	CA	ASN	2	20.292	2.791	10.218	1.00	0.00	C
ATOM	10	HA	ASN	2	20.777	1.836	10.016	1.00	0.00	H
ATOM	11	CB	ASN	2	20.089	2.844	11.726	1.00	0.00	C
ATOM	12	HB2	ASN	2	19.737	3.831	12.027	1.00	0.00	H
ATOM	13	HB3	ASN	2	21.120	2.800	12.077	1.00	0.00	H
ATOM	14	CG	ASN	2	19.428	1.739	12.423	1.00	0.00	C
ATOM	15	OD1	ASN	2	19.380	0.633	11.841	1.00	0.00	O
ATOM	16	ND2	ASN	2	18.848	1.936	13.568	1.00	0.00	N
ATOM	17	HD21	ASN	2	18.174	1.230	13.830	1.00	0.00	H
ATOM	18	HD22	ASN	2	18.850	2.911	13.828	1.00	0.00	H
ATOM	19	C	ASN	2	18.988	2.820	9.366	1.00	0.00	C
ATOM	20	O	ASN	2	18.651	3.828	8.820	1.00	0.00	O
ATOM	21	N	ASP	3	18.253	1.687	9.235	1.00	0.00	N
ATOM	22	H	ASP	3	18.548	0.988	9.902	1.00	0.00	H
ATOM	23	CA	ASP	3	17.051	1.597	8.413	1.00	0.00	C
ATOM	24	HA	ASP	3	16.997	2.499	7.803	1.00	0.00	H
ATOM	25	CB	ASP	3	17.261	0.455	7.456	1.00	0.00	C
ATOM	26	HB2	ASP	3	17.437	-0.534	7.879	1.00	0.00	H
ATOM	27	HB3	ASP	3	18.161	0.802	6.948	1.00	0.00	H
ATOM	28	CG	ASP	3	16.119	0.265	6.487	1.00	0.00	C
ATOM	29	OD1	ASP	3	15.184	-0.485	6.844	1.00	0.00	O
ATOM	30	OD2	ASP	3	16.149	0.720	5.306	1.00	0.00	O
ATOM	31	C	ASP	3	15.698	1.635	9.156	1.00	0.00	C
ATOM	32	O	ASP	3	14.753	2.175	8.584	1.00	0.00	O
ATOM	33	N	TYR	4	15.703	1.195	10.457	1.00	0.00	N
ATOM	34	H	TYR	4	16.482	0.642	10.785	1.00	0.00	H
ATOM	35	CA	TYR	4	14.494	1.158	11.207	1.00	0.00	C
ATOM	36	HA	TYR	4	13.721	0.575	10.707	1.00	0.00	H
ATOM	37	CB	TYR	4	14.786	0.414	12.497	1.00	0.00	C
ATOM	38	HB2	TYR	4	15.689	0.798	12.972	1.00	0.00	H
ATOM	39	HB3	TYR	4	14.900	-0.638	12.237	1.00	0.00	H
ATOM	40	CG	TYR	4	13.723	0.604	13.560	1.00	0.00	C
ATOM	41	CD1	TYR	4	12.523	-0.098	13.317	1.00	0.00	C
ATOM	42	HD1	TYR	4	12.501	-0.821	12.516	1.00	0.00	H
ATOM	43	CE1	TYR	4	11.370	0.211	14.149	1.00	0.00	C
ATOM	44	HE1	TYR	4	10.423	-0.242	13.896	1.00	0.00	H
ATOM	45	CZ	TYR	4	11.461	1.121	15.174	1.00	0.00	C
ATOM	46	OH	TYR	4	10.333	1.286	15.886	1.00	0.00	O
ATOM	47	HH	TYR	4	10.446	1.824	16.673	1.00	0.00	H

ATOM	48	CE2	TYR	4	12.733	1.758	15.437	1.00	0.00	C
ATOM	49	HE2	TYR	4	12.862	2.352	16.330	1.00	0.00	H
ATOM	50	CD2	TYR	4	13.823	1.442	14.727	1.00	0.00	C
ATOM	51	HD2	TYR	4	14.764	1.935	14.921	1.00	0.00	H
ATOM	52	C	TYR	4	13.967	2.573	11.428	1.00	0.00	C
ATOM	53	O	TYR	4	12.867	2.716	11.636	1.00	0.00	O
ATOM	54	N	GLU	5	14.809	3.658	11.378	1.00	0.00	N
ATOM	55	H	GLU	5	15.805	3.569	11.237	1.00	0.00	H
ATOM	56	CA	GLU	5	14.469	5.087	11.599	1.00	0.00	C
ATOM	57	HA	GLU	5	13.527	5.098	12.148	1.00	0.00	H
ATOM	58	CB	GLU	5	15.497	5.836	12.447	1.00	0.00	C
ATOM	59	HB2	GLU	5	15.164	6.838	12.718	1.00	0.00	H
ATOM	60	HB3	GLU	5	16.487	5.803	11.993	1.00	0.00	H
ATOM	61	CG	GLU	5	15.738	5.084	13.813	1.00	0.00	C
ATOM	62	HG2	GLU	5	15.782	4.001	13.696	1.00	0.00	H
ATOM	63	HG3	GLU	5	14.816	5.417	14.291	1.00	0.00	H
ATOM	64	CD	GLU	5	17.039	5.574	14.486	1.00	0.00	C
ATOM	65	OE1	GLU	5	17.057	6.682	15.111	1.00	0.00	O
ATOM	66	OE2	GLU	5	18.093	4.852	14.416	1.00	0.00	O
ATOM	67	C	GLU	5	14.157	5.868	10.286	1.00	0.00	C
ATOM	68	O	GLU	5	13.248	6.699	10.390	1.00	0.00	O
ATOM	69	N	ASP	6	14.769	5.625	9.159	1.00	0.00	N
ATOM	70	H	ASP	6	15.340	4.798	9.064	1.00	0.00	H
ATOM	71	CA	ASP	6	14.210	6.211	7.955	1.00	0.00	C
ATOM	72	HA	ASP	6	13.794	7.198	8.157	1.00	0.00	H
ATOM	73	CB	ASP	6	15.355	6.392	6.935	1.00	0.00	C
ATOM	74	HB2	ASP	6	15.949	5.480	6.885	1.00	0.00	H
ATOM	75	HB3	ASP	6	16.068	7.124	7.314	1.00	0.00	H
ATOM	76	CG	ASP	6	14.842	6.788	5.557	1.00	0.00	C
ATOM	77	OD1	ASP	6	14.182	7.905	5.408	1.00	0.00	O
ATOM	78	OD2	ASP	6	15.230	6.116	4.559	1.00	0.00	O
ATOM	79	C	ASP	6	12.915	5.530	7.377	1.00	0.00	C
ATOM	80	O	ASP	6	12.098	6.135	6.794	1.00	0.00	O
ATOM	81	N	ARG	7	12.762	4.176	7.662	1.00	0.00	N
ATOM	82	H	ARG	7	13.487	3.733	8.208	1.00	0.00	H
ATOM	83	CA	ARG	7	11.523	3.452	7.326	1.00	0.00	C
ATOM	84	HA	ARG	7	11.357	3.581	6.257	1.00	0.00	H
ATOM	85	CB	ARG	7	11.611	2.019	7.736	1.00	0.00	C
ATOM	86	HB2	ARG	7	11.702	1.994	8.822	1.00	0.00	H
ATOM	87	HB3	ARG	7	12.480	1.765	7.129	1.00	0.00	H
ATOM	88	CG	ARG	7	10.469	1.106	7.301	1.00	0.00	C
ATOM	89	HG2	ARG	7	10.409	1.158	6.214	1.00	0.00	H
ATOM	90	HG3	ARG	7	9.654	1.584	7.844	1.00	0.00	H
ATOM	91	CD	ARG	7	10.607	-0.360	7.638	1.00	0.00	C
ATOM	92	HD2	ARG	7	9.779	-0.938	7.227	1.00	0.00	H
ATOM	93	HD3	ARG	7	10.614	-0.526	8.716	1.00	0.00	H
ATOM	94	NE	ARG	7	11.745	-0.997	6.959	1.00	0.00	N
ATOM	95	HE	ARG	7	12.653	-0.845	7.373	1.00	0.00	H
ATOM	96	CZ	ARG	7	11.877	-1.686	5.789	1.00	0.00	C
ATOM	97	NH1	ARG	7	13.077	-2.121	5.366	1.00	0.00	N
ATOM	98	HH11	ARG	7	13.113	-2.412	4.400	1.00	0.00	H
ATOM	99	HH12	ARG	7	13.961	-2.002	5.840	1.00	0.00	H
ATOM	100	NH2	ARG	7	10.889	-1.764	4.910	1.00	0.00	N
ATOM	101	HH21	ARG	7	11.103	-1.950	3.940	1.00	0.00	H
ATOM	102	HH22	ARG	7	9.986	-1.409	5.192	1.00	0.00	H
ATOM	103	C	ARG	7	10.223	4.111	7.782	1.00	0.00	C
ATOM	104	O	ARG	7	9.237	4.153	7.116	1.00	0.00	O
ATOM	105	N	TYR	8	10.258	4.641	9.001	1.00	0.00	N
ATOM	106	H	TYR	8	11.103	4.777	9.537	1.00	0.00	H
ATOM	107	CA	TYR	8	9.052	5.163	9.690	1.00	0.00	C
ATOM	108	HA	TYR	8	8.271	4.403	9.689	1.00	0.00	H
ATOM	109	CB	TYR	8	9.597	5.429	11.170	1.00	0.00	C
ATOM	110	HB2	TYR	8	10.650	5.707	11.127	1.00	0.00	H

ATOM	111	HB3	TYR	8	9.465	4.461	11.653	1.00	0.00	H
ATOM	112	CG	TYR	8	8.783	6.206	12.149	1.00	0.00	C
ATOM	113	CD1	TYR	8	7.399	5.810	12.317	1.00	0.00	C
ATOM	114	HD1	TYR	8	6.939	5.013	11.751	1.00	0.00	H
ATOM	115	CE1	TYR	8	6.555	6.668	13.043	1.00	0.00	C
ATOM	116	HE1	TYR	8	5.487	6.517	13.006	1.00	0.00	H
ATOM	117	CZ	TYR	8	7.084	7.813	13.717	1.00	0.00	C
ATOM	118	OH	TYR	8	6.281	8.590	14.505	1.00	0.00	O
ATOM	119	HH	TYR	8	6.827	9.220	14.982	1.00	0.00	H
ATOM	120	CE2	TYR	8	8.494	8.051	13.736	1.00	0.00	C
ATOM	121	HE2	TYR	8	8.874	8.844	14.364	1.00	0.00	H
ATOM	122	CD2	TYR	8	9.340	7.272	12.901	1.00	0.00	C
ATOM	123	HD2	TYR	8	10.378	7.528	12.743	1.00	0.00	H
ATOM	124	C	TYR	8	8.398	6.413	9.070	1.00	0.00	C
ATOM	125	O	TYR	8	7.207	6.676	9.276	1.00	0.00	O
ATOM	126	N	TYR	9	9.249	7.111	8.272	1.00	0.00	N
ATOM	127	H	TYR	9	10.241	6.941	8.353	1.00	0.00	H
ATOM	128	CA	TYR	9	8.781	8.147	7.386	1.00	0.00	C
ATOM	129	HA	TYR	9	7.824	8.590	7.660	1.00	0.00	H
ATOM	130	CB	TYR	9	9.732	9.355	7.485	1.00	0.00	C
ATOM	131	HB2	TYR	9	9.430	10.101	6.749	1.00	0.00	H
ATOM	132	HB3	TYR	9	10.643	8.919	7.076	1.00	0.00	H
ATOM	133	CG	TYR	9	10.004	9.998	8.811	1.00	0.00	C
ATOM	134	CD1	TYR	9	9.125	10.963	9.356	1.00	0.00	C
ATOM	135	HD1	TYR	9	8.335	11.308	8.706	1.00	0.00	H
ATOM	136	CE1	TYR	9	9.326	11.478	10.635	1.00	0.00	C
ATOM	137	HE1	TYR	9	8.667	12.228	11.045	1.00	0.00	H
ATOM	138	CZ	TYR	9	10.410	11.131	11.355	1.00	0.00	C
ATOM	139	OH	TYR	9	10.710	11.673	12.584	1.00	0.00	O
ATOM	140	HH	TYR	9	11.652	11.537	12.709	1.00	0.00	H
ATOM	141	CE2	TYR	9	11.345	10.204	10.797	1.00	0.00	C
ATOM	142	HE2	TYR	9	12.221	9.944	11.373	1.00	0.00	H
ATOM	143	CD2	TYR	9	11.086	9.542	9.585	1.00	0.00	C
ATOM	144	HD2	TYR	9	11.673	8.778	9.098	1.00	0.00	H
ATOM	145	C	TYR	9	8.635	7.530	5.936	1.00	0.00	C
ATOM	146	O	TYR	9	7.605	7.810	5.285	1.00	0.00	O
ATOM	147	N	ARG	10	9.601	6.728	5.396	1.00	0.00	N
ATOM	148	H	ARG	10	10.404	6.692	6.008	1.00	0.00	H
ATOM	149	CA	ARG	10	9.532	6.076	4.065	1.00	0.00	C
ATOM	150	HA	ARG	10	9.779	6.810	3.297	1.00	0.00	H
ATOM	151	CB	ARG	10	10.568	4.931	3.972	1.00	0.00	C
ATOM	152	HB2	ARG	10	10.315	4.005	4.489	1.00	0.00	H
ATOM	153	HB3	ARG	10	11.441	5.398	4.428	1.00	0.00	H
ATOM	154	CG	ARG	10	10.973	4.531	2.591	1.00	0.00	C
ATOM	155	HG2	ARG	10	11.559	5.246	2.014	1.00	0.00	H
ATOM	156	HG3	ARG	10	10.100	4.332	1.969	1.00	0.00	H
ATOM	157	CD	ARG	10	11.944	3.309	2.626	1.00	0.00	C
ATOM	158	HD2	ARG	10	12.246	3.032	1.616	1.00	0.00	H
ATOM	159	HD3	ARG	10	11.480	2.411	3.033	1.00	0.00	H
ATOM	160	NE	ARG	10	13.207	3.641	3.311	1.00	0.00	N
ATOM	161	HE	ARG	10	13.384	4.631	3.408	1.00	0.00	H
ATOM	162	CZ	ARG	10	14.073	2.825	3.730	1.00	0.00	C
ATOM	163	NH1	ARG	10	14.069	1.542	3.578	1.00	0.00	N
ATOM	164	HH11	ARG	10	13.346	1.020	3.103	1.00	0.00	H
ATOM	165	HH12	ARG	10	14.855	1.053	3.982	1.00	0.00	H
ATOM	166	NH2	ARG	10	15.147	3.303	4.326	1.00	0.00	N
ATOM	167	HH21	ARG	10	15.268	4.303	4.400	1.00	0.00	H
ATOM	168	HH22	ARG	10	15.803	2.634	4.704	1.00	0.00	H
ATOM	169	C	ARG	10	8.121	5.424	3.775	1.00	0.00	C
ATOM	170	O	ARG	10	7.421	5.788	2.808	1.00	0.00	O
ATOM	171	N	GLU	11	7.662	4.575	4.635	1.00	0.00	N
ATOM	172	H	GLU	11	8.191	4.300	5.450	1.00	0.00	H
ATOM	173	CA	GLU	11	6.407	3.885	4.589	1.00	0.00	C

ATOM	174	HA	GLU	11	6.436	3.163	3.773	1.00	0.00	H
ATOM	175	CB	GLU	11	6.058	3.095	5.868	1.00	0.00	C
ATOM	176	HB2	GLU	11	5.376	3.721	6.443	1.00	0.00	H
ATOM	177	HB3	GLU	11	7.030	2.871	6.308	1.00	0.00	H
ATOM	178	CG	GLU	11	5.323	1.824	5.496	1.00	0.00	C
ATOM	179	HG2	GLU	11	5.917	1.144	4.884	1.00	0.00	H
ATOM	180	HG3	GLU	11	4.486	2.229	4.927	1.00	0.00	H
ATOM	181	CD	GLU	11	4.822	1.093	6.803	1.00	0.00	C
ATOM	182	OE1	GLU	11	3.799	1.616	7.277	1.00	0.00	O
ATOM	183	OE2	GLU	11	5.187	-0.050	7.133	1.00	0.00	O
ATOM	184	C	GLU	11	5.234	4.874	4.304	1.00	0.00	C
ATOM	185	O	GLU	11	4.329	4.481	3.562	1.00	0.00	O
ATOM	186	N	ASN	12	5.295	6.126	4.785	1.00	0.00	N
ATOM	187	H	ASN	12	6.203	6.439	5.099	1.00	0.00	H
ATOM	188	CA	ASN	12	4.260	7.176	4.821	1.00	0.00	C
ATOM	189	HA	ASN	12	3.334	6.608	4.913	1.00	0.00	H
ATOM	190	CB	ASN	12	4.248	7.936	6.150	1.00	0.00	C
ATOM	191	HB2	ASN	12	5.088	8.623	6.252	1.00	0.00	H
ATOM	192	HB3	ASN	12	4.270	7.169	6.924	1.00	0.00	H
ATOM	193	CG	ASN	12	2.983	8.757	6.402	1.00	0.00	C
ATOM	194	OD1	ASN	12	2.999	9.998	6.295	1.00	0.00	O
ATOM	195	ND2	ASN	12	1.916	8.077	6.849	1.00	0.00	N
ATOM	196	HD21	ASN	12	1.199	8.704	7.183	1.00	0.00	H
ATOM	197	HD22	ASN	12	1.976	7.173	7.296	1.00	0.00	H
ATOM	198	C	ASN	12	4.057	8.065	3.574	1.00	0.00	C
ATOM	199	O	ASN	12	3.161	8.916	3.419	1.00	0.00	O
ATOM	200	N	MET	13	4.916	7.862	2.626	1.00	0.00	N
ATOM	201	H	MET	13	5.658	7.198	2.792	1.00	0.00	H
ATOM	202	CA	MET	13	5.021	8.563	1.316	1.00	0.00	C
ATOM	203	HA	MET	13	4.146	9.151	1.040	1.00	0.00	H
ATOM	204	CB	MET	13	6.142	9.562	1.408	1.00	0.00	C
ATOM	205	HB2	MET	13	6.341	10.143	0.508	1.00	0.00	H
ATOM	206	HB3	MET	13	7.017	8.925	1.538	1.00	0.00	H
ATOM	207	CG	MET	13	6.039	10.591	2.504	1.00	0.00	C
ATOM	208	HG2	MET	13	5.962	10.310	3.554	1.00	0.00	H
ATOM	209	HG3	MET	13	5.219	11.212	2.144	1.00	0.00	H
ATOM	210	SD	MET	13	7.317	11.908	2.536	1.00	0.00	S
ATOM	211	CE	MET	13	8.312	11.280	3.988	1.00	0.00	C
ATOM	212	HE1	MET	13	8.181	10.208	4.137	1.00	0.00	H
ATOM	213	HE2	MET	13	9.375	11.431	3.804	1.00	0.00	H
ATOM	214	HE3	MET	13	7.999	11.788	4.900	1.00	0.00	H
ATOM	215	C	MET	13	5.317	7.588	0.185	1.00	0.00	C
ATOM	216	O	MET	13	5.420	6.377	0.318	1.00	0.00	O
ATOM	217	N	TYR	14	5.476	8.216	-1.000	1.00	0.00	N
ATOM	218	H	TYR	14	5.307	9.212	-1.000	1.00	0.00	H
ATOM	219	CA	TYR	14	5.530	7.561	-2.313	1.00	0.00	C
ATOM	220	HA	TYR	14	4.725	6.834	-2.419	1.00	0.00	H
ATOM	221	CB	TYR	14	5.416	8.609	-3.368	1.00	0.00	C
ATOM	222	HB2	TYR	14	5.261	8.227	-4.377	1.00	0.00	H
ATOM	223	HB3	TYR	14	6.374	9.130	-3.371	1.00	0.00	H
ATOM	224	CG	TYR	14	4.334	9.657	-3.163	1.00	0.00	C
ATOM	225	CD1	TYR	14	2.967	9.367	-3.396	1.00	0.00	C
ATOM	226	HD1	TYR	14	2.671	8.389	-3.745	1.00	0.00	H
ATOM	227	CE1	TYR	14	1.981	10.379	-3.236	1.00	0.00	C
ATOM	228	HE1	TYR	14	0.951	10.191	-3.501	1.00	0.00	H
ATOM	229	CZ	TYR	14	2.309	11.706	-2.840	1.00	0.00	C
ATOM	230	OH	TYR	14	1.289	12.606	-2.591	1.00	0.00	O
ATOM	231	HH	TYR	14	1.607	13.410	-2.174	1.00	0.00	H
ATOM	232	CE2	TYR	14	3.703	11.983	-2.507	1.00	0.00	C
ATOM	233	HE2	TYR	14	4.039	12.970	-2.226	1.00	0.00	H
ATOM	234	CD2	TYR	14	4.643	10.923	-2.628	1.00	0.00	C
ATOM	235	HD2	TYR	14	5.662	11.236	-2.454	1.00	0.00	H
ATOM	236	C	TYR	14	6.863	6.790	-2.589	1.00	0.00	C

```
ATOM    237  O   TYR    14       7.012   6.180  -3.621  1.00  0.00           O
ATOM    238  N   ARG    15       7.738   6.744  -1.550  1.00  0.00           N
ATOM    239  H   ARG    15       7.471   7.296  -0.747  1.00  0.00           H
ATOM    240  CA  ARG    15       8.966   5.974  -1.429  1.00  0.00           C
ATOM    241  HA  ARG    15       9.415   6.178  -2.401  1.00  0.00           H
ATOM    242  CB  ARG    15       9.976   6.589  -0.357  1.00  0.00           C
ATOM    243  HB2 ARG    15      10.972   6.149  -0.306  1.00  0.00           H
ATOM    244  HB3 ARG    15       9.479   6.364   0.587  1.00  0.00           H
ATOM    245  CG  ARG    15      10.392   8.015  -0.548  1.00  0.00           C
ATOM    246  HG2 ARG    15       9.578   8.731  -0.661  1.00  0.00           H
ATOM    247  HG3 ARG    15      10.932   8.068  -1.494  1.00  0.00           H
ATOM    248  CD  ARG    15      11.196   8.694   0.582  1.00  0.00           C
ATOM    249  HD2 ARG    15      10.599   8.773   1.491  1.00  0.00           H
ATOM    250  HD3 ARG    15      11.330   9.729   0.268  1.00  0.00           H
ATOM    251  NE  ARG    15      12.554   8.118   0.877  1.00  0.00           N
ATOM    252  HE  ARG    15      13.162   8.113   0.070  1.00  0.00           H
ATOM    253  CZ  ARG    15      13.075   7.809   2.004  1.00  0.00           C
ATOM    254  NH1 ARG    15      12.586   8.270   3.129  1.00  0.00           N
ATOM    255 HH11 ARG    15      11.663   8.680   3.135  1.00  0.00           H
ATOM    256 HH12 ARG    15      13.131   8.202   3.977  1.00  0.00           H
ATOM    257  NH2 ARG    15      14.275   7.415   2.140  1.00  0.00           N
ATOM    258 HH21 ARG    15      14.700   7.198   3.029  1.00  0.00           H
ATOM    259 HH22 ARG    15      14.740   7.321   1.248  1.00  0.00           H
ATOM    260  C   ARG    15       8.812   4.433  -1.241  1.00  0.00           C
ATOM    261  O   ARG    15       9.732   3.663  -1.454  1.00  0.00           O
ATOM    262  N   TYR    16       7.593   4.071  -0.804  1.00  0.00           N
ATOM    263  H   TYR    16       6.861   4.760  -0.910  1.00  0.00           H
ATOM    264  CA  TYR    16       7.182   2.591  -0.619  1.00  0.00           C
ATOM    265  HA  TYR    16       7.986   2.017  -1.080  1.00  0.00           H
ATOM    266  CB  TYR    16       7.140   2.191   0.846  1.00  0.00           C
ATOM    267  HB2 TYR    16       6.478   2.792   1.468  1.00  0.00           H
ATOM    268  HB3 TYR    16       8.124   2.254   1.311  1.00  0.00           H
ATOM    269  CG  TYR    16       6.634   0.748   1.034  1.00  0.00           C
ATOM    270  CD1 TYR    16       5.318   0.471   1.365  1.00  0.00           C
ATOM    271  HD1 TYR    16       4.617   1.261   1.591  1.00  0.00           H
ATOM    272  CE1 TYR    16       4.834  -0.850   1.373  1.00  0.00           C
ATOM    273  HE1 TYR    16       3.837  -1.044   1.742  1.00  0.00           H
ATOM    274  CZ  TYR    16       5.708  -1.884   0.922  1.00  0.00           C
ATOM    275  OH  TYR    16       5.267  -3.138   1.043  1.00  0.00           O
ATOM    276  HH  TYR    16       4.366  -3.167   1.375  1.00  0.00           H
ATOM    277  CE2 TYR    16       6.997  -1.579   0.548  1.00  0.00           C
ATOM    278  HE2 TYR    16       7.647  -2.395   0.267  1.00  0.00           H
ATOM    279  CD2 TYR    16       7.459  -0.267   0.627  1.00  0.00           C
ATOM    280  HD2 TYR    16       8.485  -0.002   0.420  1.00  0.00           H
ATOM    281  C   TYR    16       5.910   2.321  -1.361  1.00  0.00           C
ATOM    282  O   TYR    16       4.968   3.192  -1.361  1.00  0.00           O
ATOM    283  N   NME    17       5.709   1.149  -2.000  1.00  0.00           N
ATOM    284  H   NME    17       6.448   0.470  -2.112  1.00  0.00           H
ATOM    285  CH3 NME    17       4.447   0.868  -2.786  1.00  0.00           C
ATOM    286 HH31 NME    17       4.350  -0.043  -3.377  1.00  0.00           H
ATOM    287 HH32 NME    17       3.640   0.883  -2.054  1.00  0.00           H
ATOM    288 HH33 NME    17       4.110   1.725  -3.369  1.00  0.00           H
TER     289      NME    17
END
```

Cluster 1:

```
ATOM      1 HH31 ACE     1      20.814   0.621   8.660  1.00  0.00           H
ATOM      2  CH3 ACE     1      21.647   0.283   9.277  1.00  0.00           C
ATOM      3 HH32 ACE     1      22.587   0.746   8.975  1.00  0.00           H
ATOM      4 HH33 ACE     1      21.791  -0.785   9.117  1.00  0.00           H
```

ATOM	5	C	ACE	1	21.341	0.558	10.726	1.00	0.00	C
ATOM	6	O	ACE	1	21.898	0.005	11.669	1.00	0.00	O
ATOM	7	N	ASN	2	20.322	1.442	10.939	1.00	0.00	N
ATOM	8	H	ASN	2	19.948	1.940	10.145	1.00	0.00	H
ATOM	9	CA	ASN	2	19.540	1.592	12.204	1.00	0.00	C
ATOM	10	HA	ASN	2	19.761	0.752	12.863	1.00	0.00	H
ATOM	11	CB	ASN	2	20.067	2.918	12.819	1.00	0.00	C
ATOM	12	HB2	ASN	2	19.874	3.703	12.087	1.00	0.00	H
ATOM	13	HB3	ASN	2	21.130	2.699	12.721	1.00	0.00	H
ATOM	14	CG	ASN	2	19.659	3.283	14.251	1.00	0.00	C
ATOM	15	OD1	ASN	2	18.506	3.187	14.596	1.00	0.00	O
ATOM	16	ND2	ASN	2	20.555	3.772	15.079	1.00	0.00	N
ATOM	17	HD21	ASN	2	20.266	4.040	16.009	1.00	0.00	H
ATOM	18	HD22	ASN	2	21.523	3.862	14.804	1.00	0.00	H
ATOM	19	C	ASN	2	17.980	1.583	11.900	1.00	0.00	C
ATOM	20	O	ASN	2	17.553	2.189	10.927	1.00	0.00	O
ATOM	21	N	ASP	3	17.118	1.016	12.811	1.00	0.00	N
ATOM	22	H	ASP	3	17.457	0.343	13.484	1.00	0.00	H
ATOM	23	CA	ASP	3	15.638	1.021	12.591	1.00	0.00	C
ATOM	24	HA	ASP	3	15.306	0.497	11.695	1.00	0.00	H
ATOM	25	CB	ASP	3	14.958	0.497	13.852	1.00	0.00	C
ATOM	26	HB2	ASP	3	15.458	1.035	14.658	1.00	0.00	H
ATOM	27	HB3	ASP	3	15.122	-0.580	13.835	1.00	0.00	H
ATOM	28	CG	ASP	3	13.490	0.755	13.886	1.00	0.00	C
ATOM	29	OD1	ASP	3	13.147	1.573	14.802	1.00	0.00	O
ATOM	30	OD2	ASP	3	12.684	0.176	13.141	1.00	0.00	O
ATOM	31	C	ASP	3	15.168	2.555	12.432	1.00	0.00	C
ATOM	32	O	ASP	3	14.173	2.731	11.739	1.00	0.00	O
ATOM	33	N	TYR	4	15.774	3.446	13.253	1.00	0.00	N
ATOM	34	H	TYR	4	16.567	3.114	13.784	1.00	0.00	H
ATOM	35	CA	TYR	4	15.156	4.752	13.489	1.00	0.00	C
ATOM	36	HA	TYR	4	14.093	4.640	13.701	1.00	0.00	H
ATOM	37	CB	TYR	4	15.784	5.367	14.782	1.00	0.00	C
ATOM	38	HB2	TYR	4	15.661	6.449	14.827	1.00	0.00	H
ATOM	39	HB3	TYR	4	16.873	5.321	14.737	1.00	0.00	H
ATOM	40	CG	TYR	4	15.098	4.902	16.086	1.00	0.00	C
ATOM	41	CD1	TYR	4	14.136	5.659	16.689	1.00	0.00	C
ATOM	42	HD1	TYR	4	13.901	6.610	16.232	1.00	0.00	H
ATOM	43	CE1	TYR	4	13.490	5.234	17.876	1.00	0.00	C
ATOM	44	HE1	TYR	4	12.803	5.829	18.459	1.00	0.00	H
ATOM	45	CZ	TYR	4	13.751	3.937	18.397	1.00	0.00	C
ATOM	46	OH	TYR	4	13.162	3.562	19.558	1.00	0.00	O
ATOM	47	HH	TYR	4	13.281	2.630	19.757	1.00	0.00	H
ATOM	48	CE2	TYR	4	14.577	3.103	17.689	1.00	0.00	C
ATOM	49	HE2	TYR	4	14.714	2.166	18.208	1.00	0.00	H
ATOM	50	CD2	TYR	4	15.264	3.608	16.512	1.00	0.00	C
ATOM	51	HD2	TYR	4	15.971	2.983	15.987	1.00	0.00	H
ATOM	52	C	TYR	4	15.359	5.661	12.266	1.00	0.00	C
ATOM	53	O	TYR	4	14.592	6.638	12.047	1.00	0.00	O
ATOM	54	N	GLU	5	16.325	5.411	11.315	1.00	0.00	N
ATOM	55	H	GLU	5	16.996	4.691	11.542	1.00	0.00	H
ATOM	56	CA	GLU	5	16.371	6.047	10.019	1.00	0.00	C
ATOM	57	HA	GLU	5	15.886	7.023	10.020	1.00	0.00	H
ATOM	58	CB	GLU	5	17.824	6.044	9.521	1.00	0.00	C
ATOM	59	HB2	GLU	5	18.233	5.040	9.633	1.00	0.00	H
ATOM	60	HB3	GLU	5	18.319	6.783	10.152	1.00	0.00	H
ATOM	61	CG	GLU	5	18.007	6.454	8.086	1.00	0.00	C
ATOM	62	HG2	GLU	5	17.308	7.266	7.885	1.00	0.00	H
ATOM	63	HG3	GLU	5	17.730	5.526	7.585	1.00	0.00	H
ATOM	64	CD	GLU	5	19.435	6.863	7.780	1.00	0.00	C
ATOM	65	OE1	GLU	5	19.710	8.054	7.454	1.00	0.00	O
ATOM	66	OE2	GLU	5	20.368	6.051	7.968	1.00	0.00	O
ATOM	67	C	GLU	5	15.516	5.176	9.093	1.00	0.00	C

ATOM	68	O	GLU	5	14.633	5.745	8.430	1.00	0.00	O
ATOM	69	N	ASP	6	15.578	3.849	9.060	1.00	0.00	N
ATOM	70	H	ASP	6	16.019	3.364	9.829	1.00	0.00	H
ATOM	71	CA	ASP	6	14.849	3.049	8.085	1.00	0.00	C
ATOM	72	HA	ASP	6	15.138	3.372	7.084	1.00	0.00	H
ATOM	73	CB	ASP	6	15.256	1.573	8.170	1.00	0.00	C
ATOM	74	HB2	ASP	6	15.113	1.117	9.151	1.00	0.00	H
ATOM	75	HB3	ASP	6	16.338	1.593	8.039	1.00	0.00	H
ATOM	76	CG	ASP	6	14.648	0.649	7.098	1.00	0.00	C
ATOM	77	OD1	ASP	6	15.129	0.823	5.944	1.00	0.00	O
ATOM	78	OD2	ASP	6	13.747	-0.190	7.362	1.00	0.00	O
ATOM	79	C	ASP	6	13.276	3.237	8.168	1.00	0.00	C
ATOM	80	O	ASP	6	12.516	3.177	7.209	1.00	0.00	O
ATOM	81	N	ARG	7	12.753	3.597	9.342	1.00	0.00	N
ATOM	82	H	ARG	7	13.419	3.766	10.082	1.00	0.00	H
ATOM	83	CA	ARG	7	11.265	3.743	9.689	1.00	0.00	C
ATOM	84	HA	ARG	7	10.768	2.813	9.413	1.00	0.00	H
ATOM	85	CB	ARG	7	11.124	3.903	11.197	1.00	0.00	C
ATOM	86	HB2	ARG	7	11.562	3.036	11.692	1.00	0.00	H
ATOM	87	HB3	ARG	7	10.075	3.823	11.481	1.00	0.00	H
ATOM	88	CG	ARG	7	11.728	5.130	11.889	1.00	0.00	C
ATOM	89	HG2	ARG	7	11.539	6.055	11.344	1.00	0.00	H
ATOM	90	HG3	ARG	7	12.805	4.985	11.793	1.00	0.00	H
ATOM	91	CD	ARG	7	11.293	5.391	13.292	1.00	0.00	C
ATOM	92	HD2	ARG	7	10.230	5.621	13.357	1.00	0.00	H
ATOM	93	HD3	ARG	7	11.921	6.236	13.578	1.00	0.00	H
ATOM	94	NE	ARG	7	11.520	4.245	14.219	1.00	0.00	N
ATOM	95	HE	ARG	7	12.129	3.504	13.903	1.00	0.00	H
ATOM	96	CZ	ARG	7	11.007	4.187	15.462	1.00	0.00	C
ATOM	97	NH1	ARG	7	10.115	4.930	15.961	1.00	0.00	N
ATOM	98	HH11	ARG	7	9.828	4.832	16.924	1.00	0.00	H
ATOM	99	HH12	ARG	7	9.643	5.552	15.321	1.00	0.00	H
ATOM	100	NH2	ARG	7	11.369	3.055	16.040	1.00	0.00	N
ATOM	101	HH21	ARG	7	12.144	2.617	15.562	1.00	0.00	H
ATOM	102	HH22	ARG	7	11.309	2.983	17.046	1.00	0.00	H
ATOM	103	C	ARG	7	10.443	4.836	8.932	1.00	0.00	C
ATOM	104	O	ARG	7	9.268	4.619	8.711	1.00	0.00	O
ATOM	105	N	TYR	8	11.039	5.969	8.547	1.00	0.00	N
ATOM	106	H	TYR	8	12.029	6.089	8.708	1.00	0.00	H
ATOM	107	CA	TYR	8	10.439	6.911	7.629	1.00	0.00	C
ATOM	108	HA	TYR	8	9.465	7.288	7.940	1.00	0.00	H
ATOM	109	CB	TYR	8	11.386	8.129	7.546	1.00	0.00	C
ATOM	110	HB2	TYR	8	12.345	7.713	7.239	1.00	0.00	H
ATOM	111	HB3	TYR	8	11.372	8.513	8.566	1.00	0.00	H
ATOM	112	CG	TYR	8	10.949	9.166	6.574	1.00	0.00	C
ATOM	113	CD1	TYR	8	9.654	9.750	6.799	1.00	0.00	C
ATOM	114	HD1	TYR	8	9.233	9.514	7.765	1.00	0.00	H
ATOM	115	CE1	TYR	8	9.057	10.575	5.943	1.00	0.00	C
ATOM	116	HE1	TYR	8	8.132	11.060	6.217	1.00	0.00	H
ATOM	117	CZ	TYR	8	9.766	10.910	4.728	1.00	0.00	C
ATOM	118	OH	TYR	8	9.256	11.933	4.025	1.00	0.00	O
ATOM	119	HH	TYR	8	9.703	12.070	3.188	1.00	0.00	H
ATOM	120	CE2	TYR	8	10.989	10.238	4.412	1.00	0.00	C
ATOM	121	HE2	TYR	8	11.517	10.564	3.529	1.00	0.00	H
ATOM	122	CD2	TYR	8	11.579	9.373	5.339	1.00	0.00	C
ATOM	123	HD2	TYR	8	12.561	8.974	5.129	1.00	0.00	H
ATOM	124	C	TYR	8	10.193	6.303	6.208	1.00	0.00	C
ATOM	125	O	TYR	8	9.075	6.330	5.707	1.00	0.00	O
ATOM	126	N	TYR	9	11.164	5.633	5.702	1.00	0.00	N
ATOM	127	H	TYR	9	12.033	5.585	6.214	1.00	0.00	H
ATOM	128	CA	TYR	9	11.165	5.115	4.262	1.00	0.00	C
ATOM	129	HA	TYR	9	10.714	5.988	3.788	1.00	0.00	H
ATOM	130	CB	TYR	9	12.542	4.823	3.728	1.00	0.00	C

ATOM	131	HB2	TYR	9	12.370	4.719	2.656	1.00	0.00	H
ATOM	132	HB3	TYR	9	12.737	3.914	4.296	1.00	0.00	H
ATOM	133	CG	TYR	9	13.692	5.805	3.900	1.00	0.00	C
ATOM	134	CD1	TYR	9	13.825	6.948	3.023	1.00	0.00	C
ATOM	135	HD1	TYR	9	12.987	7.235	2.405	1.00	0.00	H
ATOM	136	CE1	TYR	9	14.957	7.762	3.049	1.00	0.00	C
ATOM	137	HE1	TYR	9	15.040	8.603	2.375	1.00	0.00	H
ATOM	138	CZ	TYR	9	15.971	7.597	4.055	1.00	0.00	C
ATOM	139	OH	TYR	9	16.944	8.508	4.175	1.00	0.00	O
ATOM	140	HH	TYR	9	16.845	9.212	3.529	1.00	0.00	H
ATOM	141	CE2	TYR	9	15.842	6.527	4.946	1.00	0.00	C
ATOM	142	HE2	TYR	9	16.570	6.348	5.723	1.00	0.00	H
ATOM	143	CD2	TYR	9	14.768	5.565	4.815	1.00	0.00	C
ATOM	144	HD2	TYR	9	14.696	4.664	5.407	1.00	0.00	H
ATOM	145	C	TYR	9	10.142	4.006	3.998	1.00	0.00	C
ATOM	146	O	TYR	9	9.908	3.620	2.840	1.00	0.00	O
ATOM	147	N	ARG	10	9.615	3.538	5.185	1.00	0.00	N
ATOM	148	H	ARG	10	10.043	3.821	6.054	1.00	0.00	H
ATOM	149	CA	ARG	10	8.691	2.520	5.115	1.00	0.00	C
ATOM	150	HA	ARG	10	9.001	1.794	4.364	1.00	0.00	H
ATOM	151	CB	ARG	10	8.630	1.741	6.401	1.00	0.00	C
ATOM	152	HB2	ARG	10	7.667	1.232	6.372	1.00	0.00	H
ATOM	153	HB3	ARG	10	8.728	2.540	7.135	1.00	0.00	H
ATOM	154	CG	ARG	10	9.781	0.764	6.744	1.00	0.00	C
ATOM	155	HG2	ARG	10	10.770	1.219	6.724	1.00	0.00	H
ATOM	156	HG3	ARG	10	9.534	-0.008	6.016	1.00	0.00	H
ATOM	157	CD	ARG	10	9.652	0.062	8.116	1.00	0.00	C
ATOM	158	HD2	ARG	10	8.942	-0.764	8.085	1.00	0.00	H
ATOM	159	HD3	ARG	10	9.096	0.825	8.662	1.00	0.00	H
ATOM	160	NE	ARG	10	10.931	-0.253	8.770	1.00	0.00	N
ATOM	161	HE	ARG	10	11.726	-0.515	8.205	1.00	0.00	H
ATOM	162	CZ	ARG	10	11.238	-0.088	10.004	1.00	0.00	C
ATOM	163	NH1	ARG	10	10.389	0.172	10.978	1.00	0.00	N
ATOM	164	HH11	ARG	10	9.412	0.212	10.723	1.00	0.00	H
ATOM	165	HH12	ARG	10	10.705	0.269	11.931	1.00	0.00	H
ATOM	166	NH2	ARG	10	12.475	-0.047	10.460	1.00	0.00	N
ATOM	167	HH21	ARG	10	12.652	0.200	11.423	1.00	0.00	H
ATOM	168	HH22	ARG	10	13.234	-0.392	9.890	1.00	0.00	H
ATOM	169	C	ARG	10	7.360	3.016	4.628	1.00	0.00	C
ATOM	170	O	ARG	10	6.628	2.221	3.969	1.00	0.00	O
ATOM	171	N	GLU	11	7.012	4.268	4.898	1.00	0.00	N
ATOM	172	H	GLU	11	7.541	4.811	5.565	1.00	0.00	H
ATOM	173	CA	GLU	11	5.732	4.953	4.399	1.00	0.00	C
ATOM	174	HA	GLU	11	5.004	4.228	4.036	1.00	0.00	H
ATOM	175	CB	GLU	11	5.096	5.708	5.591	1.00	0.00	C
ATOM	176	HB2	GLU	11	5.741	6.550	5.839	1.00	0.00	H
ATOM	177	HB3	GLU	11	5.112	5.161	6.534	1.00	0.00	H
ATOM	178	CG	GLU	11	3.730	6.294	5.285	1.00	0.00	C
ATOM	179	HG2	GLU	11	3.119	5.498	4.856	1.00	0.00	H
ATOM	180	HG3	GLU	11	3.852	7.094	4.555	1.00	0.00	H
ATOM	181	CD	GLU	11	3.088	6.978	6.454	1.00	0.00	C
ATOM	182	OE1	GLU	11	3.495	8.097	6.645	1.00	0.00	O
ATOM	183	OE2	GLU	11	2.306	6.341	7.174	1.00	0.00	O
ATOM	184	C	GLU	11	5.943	5.739	3.153	1.00	0.00	C
ATOM	185	O	GLU	11	5.014	6.072	2.465	1.00	0.00	O
ATOM	186	N	ASN	12	7.201	6.116	2.887	1.00	0.00	N
ATOM	187	H	ASN	12	7.789	5.862	3.668	1.00	0.00	H
ATOM	188	CA	ASN	12	7.639	7.076	1.785	1.00	0.00	C
ATOM	189	HA	ASN	12	6.956	7.924	1.837	1.00	0.00	H
ATOM	190	CB	ASN	12	9.040	7.495	1.949	1.00	0.00	C
ATOM	191	HB2	ASN	12	9.704	6.664	1.707	1.00	0.00	H
ATOM	192	HB3	ASN	12	9.171	7.811	2.983	1.00	0.00	H
ATOM	193	CG	ASN	12	9.360	8.642	1.024	1.00	0.00	C

ATOM	194	OD1	ASN	12	8.876	9.691	1.365	1.00	0.00	O
ATOM	195	ND2	ASN	12	10.025	8.579	-0.081	1.00	0.00	N
ATOM	196	HD21	ASN	12	10.290	9.479	-0.455	1.00	0.00	H
ATOM	197	HD22	ASN	12	10.371	7.696	-0.428	1.00	0.00	H
ATOM	198	C	ASN	12	7.417	6.485	0.373	1.00	0.00	C
ATOM	199	O	ASN	12	7.110	7.144	-0.623	1.00	0.00	O
ATOM	200	N	MET	13	7.410	5.174	0.362	1.00	0.00	N
ATOM	201	H	MET	13	7.589	4.698	1.234	1.00	0.00	H
ATOM	202	CA	MET	13	6.981	4.326	-0.763	1.00	0.00	C
ATOM	203	HA	MET	13	7.526	4.589	-1.669	1.00	0.00	H
ATOM	204	CB	MET	13	7.222	2.836	-0.449	1.00	0.00	C
ATOM	205	HB2	MET	13	6.714	2.115	-1.089	1.00	0.00	H
ATOM	206	HB3	MET	13	6.902	2.650	0.576	1.00	0.00	H
ATOM	207	CG	MET	13	8.696	2.626	-0.746	1.00	0.00	C
ATOM	208	HG2	MET	13	9.239	3.186	0.015	1.00	0.00	H
ATOM	209	HG3	MET	13	8.814	3.026	-1.753	1.00	0.00	H
ATOM	210	SD	MET	13	9.252	0.903	-0.659	1.00	0.00	S
ATOM	211	CE	MET	13	9.124	0.668	1.166	1.00	0.00	C
ATOM	212	HE1	MET	13	8.120	0.718	1.589	1.00	0.00	H
ATOM	213	HE2	MET	13	9.413	-0.347	1.436	1.00	0.00	H
ATOM	214	HE3	MET	13	9.749	1.450	1.596	1.00	0.00	H
ATOM	215	C	MET	13	5.511	4.533	-1.109	1.00	0.00	C
ATOM	216	O	MET	13	5.199	4.277	-2.226	1.00	0.00	O
ATOM	217	N	TYR	14	4.685	5.024	-0.200	1.00	0.00	N
ATOM	218	H	TYR	14	5.075	5.145	0.724	1.00	0.00	H
ATOM	219	CA	TYR	14	3.281	5.510	-0.487	1.00	0.00	C
ATOM	220	HA	TYR	14	2.968	5.189	-1.480	1.00	0.00	H
ATOM	221	CB	TYR	14	2.354	4.955	0.514	1.00	0.00	C
ATOM	222	HB2	TYR	14	1.306	5.236	0.407	1.00	0.00	H
ATOM	223	HB3	TYR	14	2.779	5.257	1.470	1.00	0.00	H
ATOM	224	CG	TYR	14	2.266	3.442	0.615	1.00	0.00	C
ATOM	225	CD1	TYR	14	3.158	2.789	1.494	1.00	0.00	C
ATOM	226	HD1	TYR	14	3.891	3.354	2.050	1.00	0.00	H
ATOM	227	CE1	TYR	14	3.129	1.436	1.666	1.00	0.00	C
ATOM	228	HE1	TYR	14	3.768	0.921	2.368	1.00	0.00	H
ATOM	229	CZ	TYR	14	2.258	0.666	0.946	1.00	0.00	C
ATOM	230	OH	TYR	14	2.369	-0.711	1.052	1.00	0.00	O
ATOM	231	HH	TYR	14	1.623	-1.165	0.654	1.00	0.00	H
ATOM	232	CE2	TYR	14	1.345	1.272	0.071	1.00	0.00	C
ATOM	233	HE2	TYR	14	0.785	0.626	-0.590	1.00	0.00	H
ATOM	234	CD2	TYR	14	1.319	2.663	-0.111	1.00	0.00	C
ATOM	235	HD2	TYR	14	0.579	3.020	-0.811	1.00	0.00	H
ATOM	236	C	TYR	14	3.144	7.026	-0.657	1.00	0.00	C
ATOM	237	O	TYR	14	2.098	7.462	-1.229	1.00	0.00	O
ATOM	238	N	ARG	15	4.109	7.800	-0.171	1.00	0.00	N
ATOM	239	H	ARG	15	4.943	7.367	0.199	1.00	0.00	H
ATOM	240	CA	ARG	15	4.105	9.340	-0.222	1.00	0.00	C
ATOM	241	HA	ARG	15	3.078	9.681	-0.094	1.00	0.00	H
ATOM	242	CB	ARG	15	5.159	9.875	0.798	1.00	0.00	C
ATOM	243	HB2	ARG	15	6.165	10.055	0.417	1.00	0.00	H
ATOM	244	HB3	ARG	15	5.210	9.199	1.653	1.00	0.00	H
ATOM	245	CG	ARG	15	4.579	11.094	1.447	1.00	0.00	C
ATOM	246	HG2	ARG	15	3.661	10.793	1.951	1.00	0.00	H
ATOM	247	HG3	ARG	15	4.338	11.776	0.632	1.00	0.00	H
ATOM	248	CD	ARG	15	5.478	11.656	2.515	1.00	0.00	C
ATOM	249	HD2	ARG	15	5.002	12.447	3.095	1.00	0.00	H
ATOM	250	HD3	ARG	15	6.226	12.181	1.920	1.00	0.00	H
ATOM	251	NE	ARG	15	6.131	10.743	3.516	1.00	0.00	N
ATOM	252	HE	ARG	15	7.140	10.706	3.536	1.00	0.00	H
ATOM	253	CZ	ARG	15	5.480	10.110	4.474	1.00	0.00	C
ATOM	254	NH1	ARG	15	4.223	10.295	4.746	1.00	0.00	N
ATOM	255	HH11	ARG	15	3.743	9.653	5.359	1.00	0.00	H
ATOM	256	HH12	ARG	15	3.594	10.828	4.162	1.00	0.00	H

```
ATOM    257  NH2 ARG    15        6.120   9.277   5.241  1.00  0.00             N
ATOM    258 HH21 ARG    15        5.570   8.742   5.899  1.00  0.00             H
ATOM    259 HH22 ARG    15        7.061   8.941   5.099  1.00  0.00             H
ATOM    260  C   ARG    15        4.441   9.840  -1.589  1.00  0.00             C
ATOM    261  O   ARG    15        3.823  10.753  -2.143  1.00  0.00             O
ATOM    262  N   TYR    16        5.501   9.235  -2.166  1.00  0.00             N
ATOM    263  H   TYR    16        6.143   8.604  -1.709  1.00  0.00             H
ATOM    264  CA  TYR    16        5.917   9.477  -3.570  1.00  0.00             C
ATOM    265  HA  TYR    16        5.246  10.108  -4.153  1.00  0.00             H
ATOM    266  CB  TYR    16        7.305  10.252  -3.616  1.00  0.00             C
ATOM    267  HB2 TYR    16        7.693  10.508  -4.602  1.00  0.00             H
ATOM    268  HB3 TYR    16        8.077   9.536  -3.335  1.00  0.00             H
ATOM    269  CG  TYR    16        7.399  11.492  -2.840  1.00  0.00             C
ATOM    270  CD1 TYR    16        7.153  12.664  -3.552  1.00  0.00             C
ATOM    271  HD1 TYR    16        6.941  12.578  -4.608  1.00  0.00             H
ATOM    272  CE1 TYR    16        7.048  13.905  -2.820  1.00  0.00             C
ATOM    273  HE1 TYR    16        6.818  14.854  -3.281  1.00  0.00             H
ATOM    274  CZ  TYR    16        7.356  13.977  -1.447  1.00  0.00             C
ATOM    275  OH  TYR    16        7.084  15.075  -0.683  1.00  0.00             O
ATOM    276  HH  TYR    16        6.843  15.782  -1.286  1.00  0.00             H
ATOM    277  CE2 TYR    16        7.727  12.752  -0.797  1.00  0.00             C
ATOM    278  HE2 TYR    16        7.965  12.753   0.257  1.00  0.00             H
ATOM    279  CD2 TYR    16        7.728  11.534  -1.464  1.00  0.00             C
ATOM    280  HD2 TYR    16        7.906  10.575  -1.001  1.00  0.00             H
ATOM    281  C   TYR    16        5.997   8.135  -4.372  1.00  0.00             C
ATOM    282  O   TYR    16        6.530   7.130  -3.944  1.00  0.00             O
ATOM    283  N   NME    17        5.538   8.204  -5.668  1.00  0.00             N
ATOM    284  H   NME    17        5.177   9.084  -6.009  1.00  0.00             H
ATOM    285  CH3 NME    17        5.467   7.121  -6.572  1.00  0.00             C
ATOM    286 HH31 NME    17        4.711   6.410  -6.240  1.00  0.00             H
ATOM    287 HH32 NME    17        5.121   7.589  -7.494  1.00  0.00             H
ATOM    288 HH33 NME    17        6.433   6.667  -6.794  1.00  0.00             H
TER     289      NME    17
END
```

Cluster 2:

```
ATOM      1 HH31 ACE     1       19.677   6.076  13.025  1.00  0.00             H
ATOM      2  CH3 ACE     1       20.283   6.126  12.120  1.00  0.00             C
ATOM      3 HH32 ACE     1       21.268   5.697  12.304  1.00  0.00             H
ATOM      4 HH33 ACE     1       20.366   7.179  11.849  1.00  0.00             H
ATOM      5  C   ACE     1       19.824   5.362  10.953  1.00  0.00             C
ATOM      6  O   ACE     1       19.734   5.860   9.837  1.00  0.00             O
ATOM      7  N   ASN     2       19.509   4.153  11.276  1.00  0.00             N
ATOM      8  H   ASN     2       19.611   3.814  12.222  1.00  0.00             H
ATOM      9  CA  ASN     2       18.883   3.242  10.333  1.00  0.00             C
ATOM     10  HA  ASN     2       19.361   3.451   9.376  1.00  0.00             H
ATOM     11  CB  ASN     2       19.102   1.728  10.781  1.00  0.00             C
ATOM     12  HB2 ASN     2       20.126   1.362  10.706  1.00  0.00             H
ATOM     13  HB3 ASN     2       18.494   1.219  10.033  1.00  0.00             H
ATOM     14  CG  ASN     2       18.617   1.327  12.172  1.00  0.00             C
ATOM     15  OD1 ASN     2       19.321   1.507  13.145  1.00  0.00             O
ATOM     16  ND2 ASN     2       17.461   0.738  12.392  1.00  0.00             N
ATOM     17 HD21 ASN     2       17.019   0.371  11.561  1.00  0.00             H
ATOM     18 HD22 ASN     2       17.096   0.563  13.318  1.00  0.00             H
ATOM     19  C   ASN     2       17.365   3.546  10.275  1.00  0.00             C
ATOM     20  O   ASN     2       16.548   2.901   9.555  1.00  0.00             O
ATOM     21  N   ASP     3       16.894   4.551  10.993  1.00  0.00             N
ATOM     22  H   ASP     3       17.422   4.842  11.804  1.00  0.00             H
ATOM     23  CA  ASP     3       15.481   5.000  10.878  1.00  0.00             C
ATOM     24  HA  ASP     3       14.873   4.096  10.879  1.00  0.00             H
```

ATOM	25	CB	ASP	3	15.041	5.783	12.163	1.00	0.00	C
ATOM	26	HB2	ASP	3	15.786	6.557	12.345	1.00	0.00	H
ATOM	27	HB3	ASP	3	15.210	5.037	12.939	1.00	0.00	H
ATOM	28	CG	ASP	3	13.596	6.295	12.121	1.00	0.00	C
ATOM	29	OD1	ASP	3	12.628	5.588	12.506	1.00	0.00	O
ATOM	30	OD2	ASP	3	13.496	7.509	11.748	1.00	0.00	O
ATOM	31	C	ASP	3	15.093	5.762	9.573	1.00	0.00	C
ATOM	32	O	ASP	3	13.961	5.544	9.083	1.00	0.00	O
ATOM	33	N	TYR	4	15.948	6.414	8.885	1.00	0.00	N
ATOM	34	H	TYR	4	16.807	6.570	9.391	1.00	0.00	H
ATOM	35	CA	TYR	4	15.826	6.912	7.524	1.00	0.00	C
ATOM	36	HA	TYR	4	14.794	7.259	7.484	1.00	0.00	H
ATOM	37	CB	TYR	4	16.810	8.022	7.244	1.00	0.00	C
ATOM	38	HB2	TYR	4	16.969	8.317	6.207	1.00	0.00	H
ATOM	39	HB3	TYR	4	17.818	7.692	7.497	1.00	0.00	H
ATOM	40	CG	TYR	4	16.645	9.421	7.968	1.00	0.00	C
ATOM	41	CD1	TYR	4	17.633	9.876	8.924	1.00	0.00	C
ATOM	42	HD1	TYR	4	18.432	9.260	9.311	1.00	0.00	H
ATOM	43	CE1	TYR	4	17.486	11.227	9.458	1.00	0.00	C
ATOM	44	HE1	TYR	4	18.167	11.532	10.239	1.00	0.00	H
ATOM	45	CZ	TYR	4	16.465	12.069	8.945	1.00	0.00	C
ATOM	46	OH	TYR	4	16.390	13.392	9.253	1.00	0.00	O
ATOM	47	HH	TYR	4	17.111	13.674	9.820	1.00	0.00	H
ATOM	48	CE2	TYR	4	15.644	11.629	7.887	1.00	0.00	C
ATOM	49	HE2	TYR	4	14.917	12.319	7.484	1.00	0.00	H
ATOM	50	CD2	TYR	4	15.674	10.301	7.368	1.00	0.00	C
ATOM	51	HD2	TYR	4	15.060	9.916	6.568	1.00	0.00	H
ATOM	52	C	TYR	4	15.748	5.806	6.457	1.00	0.00	C
ATOM	53	O	TYR	4	15.410	5.985	5.278	1.00	0.00	O
ATOM	54	N	GLU	5	16.073	4.600	6.867	1.00	0.00	N
ATOM	55	H	GLU	5	16.225	4.523	7.863	1.00	0.00	H
ATOM	56	CA	GLU	5	15.712	3.447	6.050	1.00	0.00	C
ATOM	57	HA	GLU	5	15.634	3.714	4.996	1.00	0.00	H
ATOM	58	CB	GLU	5	16.939	2.498	5.970	1.00	0.00	C
ATOM	59	HB2	GLU	5	16.739	1.529	5.512	1.00	0.00	H
ATOM	60	HB3	GLU	5	17.154	2.259	7.011	1.00	0.00	H
ATOM	61	CG	GLU	5	18.267	2.969	5.282	1.00	0.00	C
ATOM	62	HG2	GLU	5	19.014	2.191	5.441	1.00	0.00	H
ATOM	63	HG3	GLU	5	18.522	3.874	5.834	1.00	0.00	H
ATOM	64	CD	GLU	5	18.136	3.248	3.727	1.00	0.00	C
ATOM	65	OE1	GLU	5	17.365	2.605	2.980	1.00	0.00	O
ATOM	66	OE2	GLU	5	18.744	4.204	3.195	1.00	0.00	O
ATOM	67	C	GLU	5	14.478	2.723	6.533	1.00	0.00	C
ATOM	68	O	GLU	5	13.703	2.311	5.659	1.00	0.00	O
ATOM	69	N	ASP	6	14.212	2.667	7.805	1.00	0.00	N
ATOM	70	H	ASP	6	14.975	2.891	8.427	1.00	0.00	H
ATOM	71	CA	ASP	6	13.004	1.946	8.332	1.00	0.00	C
ATOM	72	HA	ASP	6	12.926	1.029	7.748	1.00	0.00	H
ATOM	73	CB	ASP	6	13.399	1.565	9.785	1.00	0.00	C
ATOM	74	HB2	ASP	6	13.267	2.502	10.326	1.00	0.00	H
ATOM	75	HB3	ASP	6	14.420	1.185	9.744	1.00	0.00	H
ATOM	76	CG	ASP	6	12.536	0.558	10.563	1.00	0.00	C
ATOM	77	OD1	ASP	6	12.848	-0.644	10.379	1.00	0.00	O
ATOM	78	OD2	ASP	6	11.550	0.856	11.303	1.00	0.00	O
ATOM	79	C	ASP	6	11.656	2.685	8.249	1.00	0.00	C
ATOM	80	O	ASP	6	10.600	2.079	8.001	1.00	0.00	O
ATOM	81	N	ARG	7	11.599	4.039	8.248	1.00	0.00	N
ATOM	82	H	ARG	7	12.474	4.541	8.305	1.00	0.00	H
ATOM	83	CA	ARG	7	10.361	4.788	8.342	1.00	0.00	C
ATOM	84	HA	ARG	7	9.881	4.302	9.191	1.00	0.00	H
ATOM	85	CB	ARG	7	10.735	6.281	8.643	1.00	0.00	C
ATOM	86	HB2	ARG	7	11.445	6.334	9.468	1.00	0.00	H
ATOM	87	HB3	ARG	7	9.811	6.785	8.925	1.00	0.00	H

ATOM	88	CG	ARG	7	11.450	7.033	7.501	1.00	0.00	C
ATOM	89	HG2	ARG	7	10.816	6.881	6.628	1.00	0.00	H
ATOM	90	HG3	ARG	7	12.374	6.476	7.345	1.00	0.00	H
ATOM	91	CD	ARG	7	11.664	8.592	7.708	1.00	0.00	C
ATOM	92	HD2	ARG	7	10.763	9.207	7.723	1.00	0.00	H
ATOM	93	HD3	ARG	7	12.205	8.867	6.803	1.00	0.00	H
ATOM	94	NE	ARG	7	12.476	8.924	8.925	1.00	0.00	N
ATOM	95	HE	ARG	7	12.477	8.202	9.630	1.00	0.00	H
ATOM	96	CZ	ARG	7	12.883	10.117	9.254	1.00	0.00	C
ATOM	97	NH1	ARG	7	12.628	11.166	8.424	1.00	0.00	N
ATOM	98	HH11	ARG	7	12.174	11.105	7.524	1.00	0.00	H
ATOM	99	HH12	ARG	7	13.035	12.038	8.730	1.00	0.00	H
ATOM	100	NH2	ARG	7	13.552	10.234	10.353	1.00	0.00	N
ATOM	101	HH21	ARG	7	13.732	11.142	10.758	1.00	0.00	H
ATOM	102	HH22	ARG	7	13.567	9.380	10.893	1.00	0.00	H
ATOM	103	C	ARG	7	9.501	4.631	7.143	1.00	0.00	C
ATOM	104	O	ARG	7	8.253	4.580	7.268	1.00	0.00	O
ATOM	105	N	TYR	8	10.136	4.467	5.984	1.00	0.00	N
ATOM	106	H	TYR	8	11.140	4.396	6.058	1.00	0.00	H
ATOM	107	CA	TYR	8	9.525	4.505	4.708	1.00	0.00	C
ATOM	108	HA	TYR	8	8.789	5.308	4.672	1.00	0.00	H
ATOM	109	CB	TYR	8	10.571	4.922	3.681	1.00	0.00	C
ATOM	110	HB2	TYR	8	10.048	4.824	2.729	1.00	0.00	H
ATOM	111	HB3	TYR	8	11.377	4.191	3.745	1.00	0.00	H
ATOM	112	CG	TYR	8	11.048	6.364	3.832	1.00	0.00	C
ATOM	113	CD1	TYR	8	12.379	6.555	4.225	1.00	0.00	C
ATOM	114	HD1	TYR	8	13.034	5.698	4.288	1.00	0.00	H
ATOM	115	CE1	TYR	8	12.822	7.862	4.545	1.00	0.00	C
ATOM	116	HE1	TYR	8	13.767	8.034	5.039	1.00	0.00	H
ATOM	117	CZ	TYR	8	12.006	8.977	4.324	1.00	0.00	C
ATOM	118	OH	TYR	8	12.446	10.260	4.401	1.00	0.00	O
ATOM	119	HH	TYR	8	13.391	10.347	4.543	1.00	0.00	H
ATOM	120	CE2	TYR	8	10.706	8.821	3.647	1.00	0.00	C
ATOM	121	HE2	TYR	8	10.131	9.719	3.472	1.00	0.00	H
ATOM	122	CD2	TYR	8	10.251	7.456	3.461	1.00	0.00	C
ATOM	123	HD2	TYR	8	9.271	7.298	3.035	1.00	0.00	H
ATOM	124	C	TYR	8	8.765	3.197	4.287	1.00	0.00	C
ATOM	125	O	TYR	8	8.030	3.245	3.318	1.00	0.00	O
ATOM	126	N	TYR	9	8.783	2.108	5.086	1.00	0.00	N
ATOM	127	H	TYR	9	9.250	2.215	5.976	1.00	0.00	H
ATOM	128	CA	TYR	9	7.818	1.011	4.998	1.00	0.00	C
ATOM	129	HA	TYR	9	7.027	1.245	4.285	1.00	0.00	H
ATOM	130	CB	TYR	9	8.595	-0.201	4.466	1.00	0.00	C
ATOM	131	HB2	TYR	9	8.944	-0.063	3.443	1.00	0.00	H
ATOM	132	HB3	TYR	9	7.802	-0.948	4.479	1.00	0.00	H
ATOM	133	CG	TYR	9	9.791	-0.681	5.271	1.00	0.00	C
ATOM	134	CD1	TYR	9	9.694	-1.700	6.172	1.00	0.00	C
ATOM	135	HD1	TYR	9	8.730	-2.186	6.196	1.00	0.00	H
ATOM	136	CE1	TYR	9	10.798	-2.134	6.911	1.00	0.00	C
ATOM	137	HE1	TYR	9	10.739	-2.797	7.761	1.00	0.00	H
ATOM	138	CZ	TYR	9	12.120	-1.622	6.660	1.00	0.00	C
ATOM	139	OH	TYR	9	13.212	-2.109	7.282	1.00	0.00	O
ATOM	140	HH	TYR	9	12.975	-2.911	7.754	1.00	0.00	H
ATOM	141	CE2	TYR	9	12.266	-0.562	5.799	1.00	0.00	C
ATOM	142	HE2	TYR	9	13.201	-0.027	5.728	1.00	0.00	H
ATOM	143	CD2	TYR	9	11.079	-0.108	5.092	1.00	0.00	C
ATOM	144	HD2	TYR	9	11.112	0.731	4.412	1.00	0.00	H
ATOM	145	C	TYR	9	7.125	0.641	6.356	1.00	0.00	C
ATOM	146	O	TYR	9	6.048	-0.019	6.431	1.00	0.00	O
ATOM	147	N	ARG	10	7.840	0.990	7.411	1.00	0.00	N
ATOM	148	H	ARG	10	8.791	1.289	7.246	1.00	0.00	H
ATOM	149	CA	ARG	10	7.157	1.000	8.742	1.00	0.00	C
ATOM	150	HA	ARG	10	6.864	-0.020	8.991	1.00	0.00	H

ATOM	151	CB	ARG	10	8.127	1.477	9.891	1.00	0.00	C
ATOM	152	HB2	ARG	10	8.473	2.511	9.900	1.00	0.00	H
ATOM	153	HB3	ARG	10	8.971	0.796	9.783	1.00	0.00	H
ATOM	154	CG	ARG	10	7.586	1.151	11.365	1.00	0.00	C
ATOM	155	HG2	ARG	10	7.525	0.085	11.583	1.00	0.00	H
ATOM	156	HG3	ARG	10	6.587	1.586	11.349	1.00	0.00	H
ATOM	157	CD	ARG	10	8.358	1.801	12.546	1.00	0.00	C
ATOM	158	HD2	ARG	10	9.363	1.384	12.484	1.00	0.00	H
ATOM	159	HD3	ARG	10	7.746	1.591	13.423	1.00	0.00	H
ATOM	160	NE	ARG	10	8.585	3.299	12.511	1.00	0.00	N
ATOM	161	HE	ARG	10	7.828	3.963	12.590	1.00	0.00	H
ATOM	162	CZ	ARG	10	9.778	3.959	12.474	1.00	0.00	C
ATOM	163	NH1	ARG	10	10.891	3.362	12.353	1.00	0.00	N
ATOM	164	HH11	ARG	10	10.972	2.388	12.098	1.00	0.00	H
ATOM	165	HH12	ARG	10	11.714	3.947	12.379	1.00	0.00	H
ATOM	166	NH2	ARG	10	9.831	5.204	12.625	1.00	0.00	N
ATOM	167	HH21	ARG	10	8.959	5.707	12.707	1.00	0.00	H
ATOM	168	HH22	ARG	10	10.690	5.736	12.620	1.00	0.00	H
ATOM	169	C	ARG	10	5.739	1.820	8.722	1.00	0.00	C
ATOM	170	O	ARG	10	4.702	1.159	8.945	1.00	0.00	O
ATOM	171	N	GLU	11	5.690	3.179	8.616	1.00	0.00	N
ATOM	172	H	GLU	11	6.557	3.644	8.386	1.00	0.00	H
ATOM	173	CA	GLU	11	4.474	4.021	8.746	1.00	0.00	C
ATOM	174	HA	GLU	11	3.649	3.316	8.841	1.00	0.00	H
ATOM	175	CB	GLU	11	4.538	4.997	9.992	1.00	0.00	C
ATOM	176	HB2	GLU	11	3.731	5.721	10.104	1.00	0.00	H
ATOM	177	HB3	GLU	11	5.425	5.587	9.760	1.00	0.00	H
ATOM	178	CG	GLU	11	4.706	4.256	11.344	1.00	0.00	C
ATOM	179	HG2	GLU	11	5.349	3.377	11.288	1.00	0.00	H
ATOM	180	HG3	GLU	11	3.679	3.914	11.470	1.00	0.00	H
ATOM	181	CD	GLU	11	5.207	5.198	12.446	1.00	0.00	C
ATOM	182	OE1	GLU	11	6.401	5.227	12.757	1.00	0.00	O
ATOM	183	OE2	GLU	11	4.386	5.975	12.959	1.00	0.00	O
ATOM	184	C	GLU	11	4.065	4.820	7.512	1.00	0.00	C
ATOM	185	O	GLU	11	2.931	4.717	7.053	1.00	0.00	O
ATOM	186	N	ASN	12	4.877	5.689	6.945	1.00	0.00	N
ATOM	187	H	ASN	12	5.807	5.769	7.330	1.00	0.00	H
ATOM	188	CA	ASN	12	4.670	6.407	5.689	1.00	0.00	C
ATOM	189	HA	ASN	12	3.612	6.535	5.459	1.00	0.00	H
ATOM	190	CB	ASN	12	5.114	7.857	5.836	1.00	0.00	C
ATOM	191	HB2	ASN	12	6.149	8.025	5.536	1.00	0.00	H
ATOM	192	HB3	ASN	12	5.142	8.035	6.912	1.00	0.00	H
ATOM	193	CG	ASN	12	4.275	8.700	4.946	1.00	0.00	C
ATOM	194	OD1	ASN	12	4.574	8.974	3.785	1.00	0.00	O
ATOM	195	ND2	ASN	12	3.133	9.092	5.441	1.00	0.00	N
ATOM	196	HD21	ASN	12	2.478	9.696	4.966	1.00	0.00	H
ATOM	197	HD22	ASN	12	2.938	8.843	6.401	1.00	0.00	H
ATOM	198	C	ASN	12	5.272	5.714	4.428	1.00	0.00	C
ATOM	199	O	ASN	12	6.112	4.780	4.592	1.00	0.00	O
ATOM	200	N	MET	13	4.769	6.090	3.252	1.00	0.00	N
ATOM	201	H	MET	13	4.167	6.901	3.266	1.00	0.00	H
ATOM	202	CA	MET	13	5.110	5.438	2.012	1.00	0.00	C
ATOM	203	HA	MET	13	5.923	4.746	2.233	1.00	0.00	H
ATOM	204	CB	MET	13	3.925	4.698	1.362	1.00	0.00	C
ATOM	205	HB2	MET	13	4.182	4.334	0.367	1.00	0.00	H
ATOM	206	HB3	MET	13	3.074	5.379	1.347	1.00	0.00	H
ATOM	207	CG	MET	13	3.553	3.371	2.100	1.00	0.00	C
ATOM	208	HG2	MET	13	4.492	2.963	2.475	1.00	0.00	H
ATOM	209	HG3	MET	13	3.089	2.747	1.336	1.00	0.00	H
ATOM	210	SD	MET	13	2.449	3.412	3.516	1.00	0.00	S
ATOM	211	CE	MET	13	2.805	1.741	4.255	1.00	0.00	C
ATOM	212	HE1	MET	13	3.885	1.670	4.380	1.00	0.00	H
ATOM	213	HE2	MET	13	2.347	1.640	5.239	1.00	0.00	H

ATOM	214	HE3	MET	13	2.519	0.974	3.535	1.00	0.00	H
ATOM	215	C	MET	13	5.813	6.391	0.968	1.00	0.00	C
ATOM	216	O	MET	13	6.505	5.906	0.023	1.00	0.00	O
ATOM	217	N	TYR	14	5.879	7.713	1.169	1.00	0.00	N
ATOM	218	H	TYR	14	5.356	8.030	1.973	1.00	0.00	H
ATOM	219	CA	TYR	14	6.552	8.648	0.251	1.00	0.00	C
ATOM	220	HA	TYR	14	6.632	8.220	-0.748	1.00	0.00	H
ATOM	221	CB	TYR	14	5.659	9.980	0.161	1.00	0.00	C
ATOM	222	HB2	TYR	14	5.638	10.406	1.164	1.00	0.00	H
ATOM	223	HB3	TYR	14	4.714	9.598	-0.226	1.00	0.00	H
ATOM	224	CG	TYR	14	6.221	11.036	-0.799	1.00	0.00	C
ATOM	225	CD1	TYR	14	5.757	10.954	-2.126	1.00	0.00	C
ATOM	226	HD1	TYR	14	5.054	10.199	-2.449	1.00	0.00	H
ATOM	227	CE1	TYR	14	6.101	11.959	-3.028	1.00	0.00	C
ATOM	228	HE1	TYR	14	5.572	11.997	-3.969	1.00	0.00	H
ATOM	229	CZ	TYR	14	6.950	13.042	-2.616	1.00	0.00	C
ATOM	230	OH	TYR	14	7.115	14.020	-3.522	1.00	0.00	O
ATOM	231	HH	TYR	14	7.645	14.728	-3.148	1.00	0.00	H
ATOM	232	CE2	TYR	14	7.349	13.156	-1.244	1.00	0.00	C
ATOM	233	HE2	TYR	14	7.966	13.968	-0.890	1.00	0.00	H
ATOM	234	CD2	TYR	14	7.062	12.106	-0.356	1.00	0.00	C
ATOM	235	HD2	TYR	14	7.382	12.177	0.673	1.00	0.00	H
ATOM	236	C	TYR	14	8.036	8.819	0.556	1.00	0.00	C
ATOM	237	O	TYR	14	8.374	9.829	1.204	1.00	0.00	O
ATOM	238	N	ARG	15	8.923	7.888	0.074	1.00	0.00	N
ATOM	239	H	ARG	15	8.598	7.116	-0.491	1.00	0.00	H
ATOM	240	CA	ARG	15	10.298	8.258	-0.050	1.00	0.00	C
ATOM	241	HA	ARG	15	10.580	8.887	0.794	1.00	0.00	H
ATOM	242	CB	ARG	15	11.023	6.951	-0.020	1.00	0.00	C
ATOM	243	HB2	ARG	15	10.916	6.348	-0.922	1.00	0.00	H
ATOM	244	HB3	ARG	15	10.578	6.360	0.781	1.00	0.00	H
ATOM	245	CG	ARG	15	12.547	7.008	0.273	1.00	0.00	C
ATOM	246	HG2	ARG	15	12.720	7.644	1.141	1.00	0.00	H
ATOM	247	HG3	ARG	15	12.916	7.615	-0.554	1.00	0.00	H
ATOM	248	CD	ARG	15	13.362	5.627	0.478	1.00	0.00	C
ATOM	249	HD2	ARG	15	13.078	4.923	-0.303	1.00	0.00	H
ATOM	250	HD3	ARG	15	12.950	5.284	1.427	1.00	0.00	H
ATOM	251	NE	ARG	15	14.765	5.795	0.638	1.00	0.00	N
ATOM	252	HE	ARG	15	15.133	6.701	0.386	1.00	0.00	H
ATOM	253	CZ	ARG	15	15.613	4.941	1.204	1.00	0.00	C
ATOM	254	NH1	ARG	15	15.299	3.712	1.484	1.00	0.00	N
ATOM	255	HH11	ARG	15	14.372	3.383	1.252	1.00	0.00	H
ATOM	256	HH12	ARG	15	16.008	3.109	1.877	1.00	0.00	H
ATOM	257	NH2	ARG	15	16.858	5.176	1.373	1.00	0.00	N
ATOM	258	HH21	ARG	15	17.180	6.063	1.015	1.00	0.00	H
ATOM	259	HH22	ARG	15	17.386	4.654	2.059	1.00	0.00	H
ATOM	260	C	ARG	15	10.582	8.982	-1.338	1.00	0.00	C
ATOM	261	O	ARG	15	10.319	8.456	-2.435	1.00	0.00	O
ATOM	262	N	TYR	16	11.239	10.159	-1.196	1.00	0.00	N
ATOM	263	H	TYR	16	11.381	10.601	-0.300	1.00	0.00	H
ATOM	264	CA	TYR	16	11.900	10.810	-2.328	1.00	0.00	C
ATOM	265	HA	TYR	16	11.275	10.821	-3.221	1.00	0.00	H
ATOM	266	CB	TYR	16	12.342	12.210	-1.913	1.00	0.00	C
ATOM	267	HB2	TYR	16	13.405	12.237	-1.676	1.00	0.00	H
ATOM	268	HB3	TYR	16	11.747	12.393	-1.018	1.00	0.00	H
ATOM	269	CG	TYR	16	12.303	13.425	-2.850	1.00	0.00	C
ATOM	270	CD1	TYR	16	11.376	14.410	-2.729	1.00	0.00	C
ATOM	271	HD1	TYR	16	10.593	14.187	-2.019	1.00	0.00	H
ATOM	272	CE1	TYR	16	11.353	15.580	-3.536	1.00	0.00	C
ATOM	273	HE1	TYR	16	10.599	16.351	-3.479	1.00	0.00	H
ATOM	274	CZ	TYR	16	12.378	15.740	-4.426	1.00	0.00	C
ATOM	275	OH	TYR	16	12.473	16.883	-5.076	1.00	0.00	O
ATOM	276	HH	TYR	16	13.250	16.958	-5.634	1.00	0.00	H

```
ATOM    277  CE2 TYR    16      13.353  14.725  -4.565  1.00  0.00           C
ATOM    278  HE2 TYR    16      14.200  14.999  -5.178  1.00  0.00           H
ATOM    279  CD2 TYR    16      13.347  13.540  -3.794  1.00  0.00           C
ATOM    280  HD2 TYR    16      14.042  12.734  -3.979  1.00  0.00           H
ATOM    281  C   TYR    16      13.149  10.039  -2.885  1.00  0.00           C
ATOM    282  O   TYR    16      13.865   9.441  -2.127  1.00  0.00           O
ATOM    283  N   NME    17      13.225  10.105  -4.237  1.00  0.00           N
ATOM    284  H   NME    17      12.396  10.518  -4.642  1.00  0.00           H
ATOM    285  CH3 NME    17      14.264   9.456  -4.988  1.00  0.00           C
ATOM    286 HH31 NME    17      14.017   8.457  -5.348  1.00  0.00           H
ATOM    287 HH32 NME    17      14.494  10.083  -5.849  1.00  0.00           H
ATOM    288 HH33 NME    17      15.231   9.316  -4.505  1.00  0.00           H
TER     289      NME    17
END
```

Cluster 3:

```
ATOM      1 HH31 ACE     1      19.105  -1.322   7.585  1.00  0.00           H
ATOM      2  CH3 ACE     1      19.092  -1.528   8.655  1.00  0.00           C
ATOM      3 HH32 ACE     1      19.998  -2.032   8.990  1.00  0.00           H
ATOM      4 HH33 ACE     1      18.245  -2.209   8.742  1.00  0.00           H
ATOM      5  C   ACE     1      18.774  -0.288   9.512  1.00  0.00           C
ATOM      6  O   ACE     1      18.365  -0.424  10.636  1.00  0.00           O
ATOM      7  N   ASN     2      19.064   0.916   9.056  1.00  0.00           N
ATOM      8  H   ASN     2      19.452   0.995   8.127  1.00  0.00           H
ATOM      9  CA  ASN     2      18.941   2.107   9.918  1.00  0.00           C
ATOM     10  HA  ASN     2      19.326   1.912  10.919  1.00  0.00           H
ATOM     11  CB  ASN     2      19.880   3.164   9.489  1.00  0.00           C
ATOM     12  HB2 ASN     2      19.534   3.715   8.614  1.00  0.00           H
ATOM     13  HB3 ASN     2      20.791   2.616   9.249  1.00  0.00           H
ATOM     14  CG  ASN     2      20.263   4.240  10.505  1.00  0.00           C
ATOM     15  OD1 ASN     2      19.751   5.355  10.359  1.00  0.00           O
ATOM     16  ND2 ASN     2      21.058   3.934  11.486  1.00  0.00           N
ATOM     17 HD21 ASN     2      21.342   2.988  11.694  1.00  0.00           H
ATOM     18 HD22 ASN     2      21.057   4.586  12.258  1.00  0.00           H
ATOM     19  C   ASN     2      17.510   2.640  10.070  1.00  0.00           C
ATOM     20  O   ASN     2      16.819   2.904   9.093  1.00  0.00           O
ATOM     21  N   ASP     3      17.047   2.862  11.270  1.00  0.00           N
ATOM     22  H   ASP     3      17.557   2.493  12.060  1.00  0.00           H
ATOM     23  CA  ASP     3      15.637   3.229  11.555  1.00  0.00           C
ATOM     24  HA  ASP     3      15.079   2.422  11.080  1.00  0.00           H
ATOM     25  CB  ASP     3      15.486   3.064  13.059  1.00  0.00           C
ATOM     26  HB2 ASP     3      16.072   3.781  13.635  1.00  0.00           H
ATOM     27  HB3 ASP     3      15.807   2.029  13.172  1.00  0.00           H
ATOM     28  CG  ASP     3      14.062   3.203  13.436  1.00  0.00           C
ATOM     29  OD1 ASP     3      13.565   4.239  13.978  1.00  0.00           O
ATOM     30  OD2 ASP     3      13.267   2.276  13.240  1.00  0.00           O
ATOM     31  C   ASP     3      15.122   4.562  10.952  1.00  0.00           C
ATOM     32  O   ASP     3      13.958   4.699  10.601  1.00  0.00           O
ATOM     33  N   TYR     4      16.054   5.502  10.852  1.00  0.00           N
ATOM     34  H   TYR     4      16.988   5.296  11.178  1.00  0.00           H
ATOM     35  CA  TYR     4      15.845   6.787  10.104  1.00  0.00           C
ATOM     36  HA  TYR     4      14.928   7.238  10.483  1.00  0.00           H
ATOM     37  CB  TYR     4      17.029   7.720  10.353  1.00  0.00           C
ATOM     38  HB2 TYR     4      16.950   8.568   9.672  1.00  0.00           H
ATOM     39  HB3 TYR     4      17.865   7.123   9.990  1.00  0.00           H
ATOM     40  CG  TYR     4      17.354   8.397  11.718  1.00  0.00           C
ATOM     41  CD1 TYR     4      16.329   8.897  12.550  1.00  0.00           C
ATOM     42  HD1 TYR     4      15.280   8.918  12.293  1.00  0.00           H
ATOM     43  CE1 TYR     4      16.648   9.481  13.731  1.00  0.00           C
ATOM     44  HE1 TYR     4      15.785   9.843  14.270  1.00  0.00           H
```

ATOM	45	CZ	TYR	4	18.009	9.692	14.088	1.00	0.00	C
ATOM	46	OH	TYR	4	18.238	10.063	15.348	1.00	0.00	O
ATOM	47	HH	TYR	4	17.477	10.297	15.885	1.00	0.00	H
ATOM	48	CE2	TYR	4	19.042	9.205	13.229	1.00	0.00	C
ATOM	49	HE2	TYR	4	20.062	9.278	13.577	1.00	0.00	H
ATOM	50	CD2	TYR	4	18.703	8.562	12.059	1.00	0.00	C
ATOM	51	HD2	TYR	4	19.505	8.151	11.464	1.00	0.00	H
ATOM	52	C	TYR	4	15.603	6.584	8.627	1.00	0.00	C
ATOM	53	O	TYR	4	14.937	7.436	8.047	1.00	0.00	O
ATOM	54	N	GLU	5	16.049	5.495	7.979	1.00	0.00	N
ATOM	55	H	GLU	5	16.475	4.740	8.496	1.00	0.00	H
ATOM	56	CA	GLU	5	15.669	5.231	6.591	1.00	0.00	C
ATOM	57	HA	GLU	5	15.297	6.158	6.154	1.00	0.00	H
ATOM	58	CB	GLU	5	16.943	4.825	5.817	1.00	0.00	C
ATOM	59	HB2	GLU	5	17.212	3.779	5.959	1.00	0.00	H
ATOM	60	HB3	GLU	5	17.702	5.417	6.329	1.00	0.00	H
ATOM	61	CG	GLU	5	16.900	5.045	4.314	1.00	0.00	C
ATOM	62	HG2	GLU	5	16.075	4.550	3.802	1.00	0.00	H
ATOM	63	HG3	GLU	5	17.870	4.640	4.027	1.00	0.00	H
ATOM	64	CD	GLU	5	16.753	6.558	3.938	1.00	0.00	C
ATOM	65	OE1	GLU	5	17.353	7.442	4.582	1.00	0.00	O
ATOM	66	OE2	GLU	5	15.885	6.884	3.090	1.00	0.00	O
ATOM	67	C	GLU	5	14.612	4.188	6.394	1.00	0.00	C
ATOM	68	O	GLU	5	13.747	4.351	5.520	1.00	0.00	O
ATOM	69	N	ASP	6	14.503	3.340	7.448	1.00	0.00	N
ATOM	70	H	ASP	6	15.183	3.258	8.191	1.00	0.00	H
ATOM	71	CA	ASP	6	13.565	2.284	7.295	1.00	0.00	C
ATOM	72	HA	ASP	6	13.675	2.050	6.237	1.00	0.00	H
ATOM	73	CB	ASP	6	13.987	1.041	8.141	1.00	0.00	C
ATOM	74	HB2	ASP	6	13.935	1.276	9.204	1.00	0.00	H
ATOM	75	HB3	ASP	6	15.068	0.940	8.048	1.00	0.00	H
ATOM	76	CG	ASP	6	13.300	-0.273	7.688	1.00	0.00	C
ATOM	77	OD1	ASP	6	13.771	-0.873	6.646	1.00	0.00	O
ATOM	78	OD2	ASP	6	12.390	-0.663	8.445	1.00	0.00	O
ATOM	79	C	ASP	6	12.087	2.721	7.436	1.00	0.00	C
ATOM	80	O	ASP	6	11.201	1.930	7.048	1.00	0.00	O
ATOM	81	N	ARG	7	11.853	3.947	7.930	1.00	0.00	N
ATOM	82	H	ARG	7	12.717	4.424	8.141	1.00	0.00	H
ATOM	83	CA	ARG	7	10.571	4.460	8.365	1.00	0.00	C
ATOM	84	HA	ARG	7	10.051	3.591	8.767	1.00	0.00	H
ATOM	85	CB	ARG	7	10.738	5.534	9.425	1.00	0.00	C
ATOM	86	HB2	ARG	7	11.241	5.102	10.291	1.00	0.00	H
ATOM	87	HB3	ARG	7	9.732	5.768	9.773	1.00	0.00	H
ATOM	88	CG	ARG	7	11.676	6.743	9.051	1.00	0.00	C
ATOM	89	HG2	ARG	7	11.204	7.307	8.246	1.00	0.00	H
ATOM	90	HG3	ARG	7	12.597	6.236	8.763	1.00	0.00	H
ATOM	91	CD	ARG	7	11.811	7.684	10.246	1.00	0.00	C
ATOM	92	HD2	ARG	7	10.799	7.956	10.547	1.00	0.00	H
ATOM	93	HD3	ARG	7	12.364	8.539	9.858	1.00	0.00	H
ATOM	94	NE	ARG	7	12.596	7.133	11.340	1.00	0.00	N
ATOM	95	HE	ARG	7	12.952	6.202	11.177	1.00	0.00	H
ATOM	96	CZ	ARG	7	12.691	7.658	12.595	1.00	0.00	C
ATOM	97	NH1	ARG	7	12.287	8.874	12.836	1.00	0.00	N
ATOM	98	HH11	ARG	7	11.876	9.405	12.082	1.00	0.00	H
ATOM	99	HH12	ARG	7	12.521	9.357	13.692	1.00	0.00	H
ATOM	100	NH2	ARG	7	13.140	6.972	13.593	1.00	0.00	N
ATOM	101	HH21	ARG	7	13.263	7.403	14.498	1.00	0.00	H
ATOM	102	HH22	ARG	7	13.442	6.015	13.477	1.00	0.00	H
ATOM	103	C	ARG	7	9.709	5.011	7.143	1.00	0.00	C
ATOM	104	O	ARG	7	8.498	4.900	7.218	1.00	0.00	O
ATOM	105	N	TYR	8	10.423	5.434	6.023	1.00	0.00	N
ATOM	106	H	TYR	8	11.429	5.482	5.939	1.00	0.00	H
ATOM	107	CA	TYR	8	9.646	5.807	4.815	1.00	0.00	C

ATOM	108	HA	TYR	8	8.949	6.611	5.052	1.00	0.00	H
ATOM	109	CB	TYR	8	10.595	6.497	3.772	1.00	0.00	C
ATOM	110	HB2	TYR	8	9.978	6.643	2.886	1.00	0.00	H
ATOM	111	HB3	TYR	8	11.380	5.757	3.614	1.00	0.00	H
ATOM	112	CG	TYR	8	11.269	7.790	4.300	1.00	0.00	C
ATOM	113	CD1	TYR	8	12.506	7.755	4.922	1.00	0.00	C
ATOM	114	HD1	TYR	8	12.984	6.798	5.067	1.00	0.00	H
ATOM	115	CE1	TYR	8	13.200	8.911	5.208	1.00	0.00	C
ATOM	116	HE1	TYR	8	14.216	8.906	5.573	1.00	0.00	H
ATOM	117	CZ	TYR	8	12.582	10.191	4.935	1.00	0.00	C
ATOM	118	OH	TYR	8	13.360	11.293	5.038	1.00	0.00	O
ATOM	119	HH	TYR	8	12.874	12.119	4.979	1.00	0.00	H
ATOM	120	CE2	TYR	8	11.267	10.238	4.481	1.00	0.00	C
ATOM	121	HE2	TYR	8	10.791	11.206	4.532	1.00	0.00	H
ATOM	122	CD2	TYR	8	10.658	9.049	4.024	1.00	0.00	C
ATOM	123	HD2	TYR	8	9.734	9.133	3.470	1.00	0.00	H
ATOM	124	C	TYR	8	8.914	4.643	4.053	1.00	0.00	C
ATOM	125	O	TYR	8	8.146	4.898	3.126	1.00	0.00	O
ATOM	126	N	TYR	9	9.240	3.385	4.418	1.00	0.00	N
ATOM	127	H	TYR	9	9.815	3.248	5.236	1.00	0.00	H
ATOM	128	CA	TYR	9	8.608	2.151	3.892	1.00	0.00	C
ATOM	129	HA	TYR	9	7.956	2.361	3.044	1.00	0.00	H
ATOM	130	CB	TYR	9	9.742	1.231	3.446	1.00	0.00	C
ATOM	131	HB2	TYR	9	9.277	0.427	2.876	1.00	0.00	H
ATOM	132	HB3	TYR	9	10.185	0.757	4.323	1.00	0.00	H
ATOM	133	CG	TYR	9	10.846	1.880	2.561	1.00	0.00	C
ATOM	134	CD1	TYR	9	10.524	2.129	1.187	1.00	0.00	C
ATOM	135	HD1	TYR	9	9.533	1.950	0.798	1.00	0.00	H
ATOM	136	CE1	TYR	9	11.494	2.616	0.353	1.00	0.00	C
ATOM	137	HE1	TYR	9	11.317	2.658	-0.712	1.00	0.00	H
ATOM	138	CZ	TYR	9	12.791	3.010	0.814	1.00	0.00	C
ATOM	139	OH	TYR	9	13.717	3.332	-0.114	1.00	0.00	O
ATOM	140	HH	TYR	9	14.598	3.235	0.255	1.00	0.00	H
ATOM	141	CE2	TYR	9	13.102	2.924	2.188	1.00	0.00	C
ATOM	142	HE2	TYR	9	14.063	3.249	2.558	1.00	0.00	H
ATOM	143	CD2	TYR	9	12.131	2.303	3.039	1.00	0.00	C
ATOM	144	HD2	TYR	9	12.433	2.143	4.064	1.00	0.00	H
ATOM	145	C	TYR	9	7.716	1.495	4.946	1.00	0.00	C
ATOM	146	O	TYR	9	7.198	0.433	4.658	1.00	0.00	O
ATOM	147	N	ARG	10	7.527	2.074	6.179	1.00	0.00	N
ATOM	148	H	ARG	10	7.919	2.994	6.319	1.00	0.00	H
ATOM	149	CA	ARG	10	6.833	1.499	7.326	1.00	0.00	C
ATOM	150	HA	ARG	10	6.524	0.522	6.953	1.00	0.00	H
ATOM	151	CB	ARG	10	7.864	1.281	8.436	1.00	0.00	C
ATOM	152	HB2	ARG	10	8.299	2.237	8.724	1.00	0.00	H
ATOM	153	HB3	ARG	10	8.627	0.764	7.853	1.00	0.00	H
ATOM	154	CG	ARG	10	7.364	0.468	9.642	1.00	0.00	C
ATOM	155	HG2	ARG	10	7.172	-0.566	9.355	1.00	0.00	H
ATOM	156	HG3	ARG	10	6.416	0.895	9.970	1.00	0.00	H
ATOM	157	CD	ARG	10	8.321	0.396	10.821	1.00	0.00	C
ATOM	158	HD2	ARG	10	9.111	-0.261	10.457	1.00	0.00	H
ATOM	159	HD3	ARG	10	7.692	-0.077	11.574	1.00	0.00	H
ATOM	160	NE	ARG	10	8.823	1.730	11.271	1.00	0.00	N
ATOM	161	HE	ARG	10	8.122	2.453	11.344	1.00	0.00	H
ATOM	162	CZ	ARG	10	9.896	2.141	11.830	1.00	0.00	C
ATOM	163	NH1	ARG	10	10.974	1.405	11.861	1.00	0.00	N
ATOM	164	HH11	ARG	10	10.949	0.457	11.514	1.00	0.00	H
ATOM	165	HH12	ARG	10	11.783	1.784	12.332	1.00	0.00	H
ATOM	166	NH2	ARG	10	9.937	3.270	12.450	1.00	0.00	N
ATOM	167	HH21	ARG	10	9.121	3.865	12.420	1.00	0.00	H
ATOM	168	HH22	ARG	10	10.819	3.538	12.862	1.00	0.00	H
ATOM	169	C	ARG	10	5.582	2.183	7.802	1.00	0.00	C
ATOM	170	O	ARG	10	4.489	1.657	7.938	1.00	0.00	O

ATOM	171	N	GLU	11	5.720	3.479	8.045	1.00	0.00	N
ATOM	172	H	GLU	11	6.622	3.923	7.946	1.00	0.00	H
ATOM	173	CA	GLU	11	4.599	4.341	8.447	1.00	0.00	C
ATOM	174	HA	GLU	11	3.647	3.833	8.605	1.00	0.00	H
ATOM	175	CB	GLU	11	4.943	5.109	9.723	1.00	0.00	C
ATOM	176	HB2	GLU	11	4.233	5.928	9.834	1.00	0.00	H
ATOM	177	HB3	GLU	11	5.897	5.588	9.502	1.00	0.00	H
ATOM	178	CG	GLU	11	5.116	4.278	10.993	1.00	0.00	C
ATOM	179	HG2	GLU	11	5.134	3.189	10.958	1.00	0.00	H
ATOM	180	HG3	GLU	11	4.231	4.587	11.549	1.00	0.00	H
ATOM	181	CD	GLU	11	6.325	4.576	11.847	1.00	0.00	C
ATOM	182	OE1	GLU	11	6.357	5.582	12.555	1.00	0.00	O
ATOM	183	OE2	GLU	11	7.401	3.989	11.626	1.00	0.00	O
ATOM	184	C	GLU	11	4.206	5.286	7.303	1.00	0.00	C
ATOM	185	O	GLU	11	3.066	5.490	7.021	1.00	0.00	O
ATOM	186	N	ASN	12	5.152	5.935	6.598	1.00	0.00	N
ATOM	187	H	ASN	12	6.105	5.600	6.630	1.00	0.00	H
ATOM	188	CA	ASN	12	4.859	6.568	5.349	1.00	0.00	C
ATOM	189	HA	ASN	12	3.974	7.204	5.374	1.00	0.00	H
ATOM	190	CB	ASN	12	5.880	7.689	5.036	1.00	0.00	C
ATOM	191	HB2	ASN	12	6.852	7.230	4.856	1.00	0.00	H
ATOM	192	HB3	ASN	12	5.937	8.295	5.940	1.00	0.00	H
ATOM	193	CG	ASN	12	5.602	8.742	3.902	1.00	0.00	C
ATOM	194	OD1	ASN	12	4.954	8.512	2.902	1.00	0.00	O
ATOM	195	ND2	ASN	12	6.161	9.944	3.969	1.00	0.00	N
ATOM	196	HD21	ASN	12	5.901	10.640	3.285	1.00	0.00	H
ATOM	197	HD22	ASN	12	6.631	10.202	4.825	1.00	0.00	H
ATOM	198	C	ASN	12	4.751	5.541	4.237	1.00	0.00	C
ATOM	199	O	ASN	12	5.197	4.360	4.382	1.00	0.00	O
ATOM	200	N	MET	13	4.168	5.908	3.095	1.00	0.00	N
ATOM	201	H	MET	13	3.918	6.878	2.966	1.00	0.00	H
ATOM	202	CA	MET	13	3.969	5.125	1.871	1.00	0.00	C
ATOM	203	HA	MET	13	3.937	4.075	2.162	1.00	0.00	H
ATOM	204	CB	MET	13	2.531	5.433	1.340	1.00	0.00	C
ATOM	205	HB2	MET	13	1.786	5.098	2.062	1.00	0.00	H
ATOM	206	HB3	MET	13	2.317	4.876	0.427	1.00	0.00	H
ATOM	207	CG	MET	13	2.265	6.902	1.035	1.00	0.00	C
ATOM	208	HG2	MET	13	1.588	6.934	0.181	1.00	0.00	H
ATOM	209	HG3	MET	13	3.230	7.201	0.627	1.00	0.00	H
ATOM	210	SD	MET	13	1.739	8.017	2.424	1.00	0.00	S
ATOM	211	CE	MET	13	0.826	9.332	1.472	1.00	0.00	C
ATOM	212	HE1	MET	13	1.422	9.774	0.674	1.00	0.00	H
ATOM	213	HE2	MET	13	0.035	8.803	0.939	1.00	0.00	H
ATOM	214	HE3	MET	13	0.285	10.047	2.092	1.00	0.00	H
ATOM	215	C	MET	13	4.977	5.277	0.719	1.00	0.00	C
ATOM	216	O	MET	13	4.987	4.403	-0.166	1.00	0.00	O
ATOM	217	N	TYR	14	5.800	6.346	0.860	1.00	0.00	N
ATOM	218	H	TYR	14	5.548	7.029	1.559	1.00	0.00	H
ATOM	219	CA	TYR	14	6.892	6.643	-0.100	1.00	0.00	C
ATOM	220	HA	TYR	14	7.212	5.743	-0.625	1.00	0.00	H
ATOM	221	CB	TYR	14	6.349	7.681	-1.153	1.00	0.00	C
ATOM	222	HB2	TYR	14	6.161	8.655	-0.703	1.00	0.00	H
ATOM	223	HB3	TYR	14	5.341	7.351	-1.405	1.00	0.00	H
ATOM	224	CG	TYR	14	7.037	7.825	-2.529	1.00	0.00	C
ATOM	225	CD1	TYR	14	7.537	6.598	-3.119	1.00	0.00	C
ATOM	226	HD1	TYR	14	7.396	5.615	-2.695	1.00	0.00	H
ATOM	227	CE1	TYR	14	8.131	6.666	-4.429	1.00	0.00	C
ATOM	228	HE1	TYR	14	8.255	5.708	-4.911	1.00	0.00	H
ATOM	229	CZ	TYR	14	8.343	7.953	-4.991	1.00	0.00	C
ATOM	230	OH	TYR	14	8.832	8.057	-6.251	1.00	0.00	O
ATOM	231	HH	TYR	14	8.767	7.152	-6.566	1.00	0.00	H
ATOM	232	CE2	TYR	14	7.952	9.135	-4.301	1.00	0.00	C
ATOM	233	HE2	TYR	14	8.244	10.082	-4.729	1.00	0.00	H

```
ATOM    234  CD2 TYR    14       7.190   9.072  -3.105  1.00  0.00           C
ATOM    235  HD2 TYR    14       6.872   9.911  -2.504  1.00  0.00           H
ATOM    236  C   TYR    14       8.146   7.359   0.528  1.00  0.00           C
ATOM    237  O   TYR    14       8.160   8.090   1.553  1.00  0.00           O
ATOM    238  N   ARG    15       9.184   7.178  -0.240  1.00  0.00           N
ATOM    239  H   ARG    15       9.154   6.571  -1.047  1.00  0.00           H
ATOM    240  CA  ARG    15      10.486   7.810  -0.060  1.00  0.00           C
ATOM    241  HA  ARG    15      10.520   8.211   0.953  1.00  0.00           H
ATOM    242  CB  ARG    15      11.608   6.671  -0.113  1.00  0.00           C
ATOM    243  HB2 ARG    15      11.825   6.459  -1.160  1.00  0.00           H
ATOM    244  HB3 ARG    15      11.079   5.793   0.258  1.00  0.00           H
ATOM    245  CG  ARG    15      12.829   6.971   0.661  1.00  0.00           C
ATOM    246  HG2 ARG    15      13.616   6.354   0.228  1.00  0.00           H
ATOM    247  HG3 ARG    15      12.614   6.657   1.683  1.00  0.00           H
ATOM    248  CD  ARG    15      13.361   8.399   0.597  1.00  0.00           C
ATOM    249  HD2 ARG    15      12.691   9.127   1.053  1.00  0.00           H
ATOM    250  HD3 ARG    15      13.558   8.519  -0.469  1.00  0.00           H
ATOM    251  NE  ARG    15      14.600   8.602   1.403  1.00  0.00           N
ATOM    252  HE  ARG    15      14.948   7.765   1.848  1.00  0.00           H
ATOM    253  CZ  ARG    15      15.368   9.657   1.561  1.00  0.00           C
ATOM    254  NH1 ARG    15      15.110  10.839   1.059  1.00  0.00           N
ATOM    255 HH11 ARG    15      14.385  10.910   0.360  1.00  0.00           H
ATOM    256 HH12 ARG    15      15.632  11.661   1.328  1.00  0.00           H
ATOM    257  NH2 ARG    15      16.401   9.620   2.349  1.00  0.00           N
ATOM    258 HH21 ARG    15      16.475   8.792   2.922  1.00  0.00           H
ATOM    259 HH22 ARG    15      17.060  10.356   2.560  1.00  0.00           H
ATOM    260  C   ARG    15      10.707   8.917  -1.055  1.00  0.00           C
ATOM    261  O   ARG    15      11.046   8.666  -2.218  1.00  0.00           O
ATOM    262  N   TYR    16      10.370  10.068  -0.532  1.00  0.00           N
ATOM    263  H   TYR    16       9.859  10.073   0.339  1.00  0.00           H
ATOM    264  CA  TYR    16      10.652  11.392  -1.142  1.00  0.00           C
ATOM    265  HA  TYR    16      10.165  11.323  -2.115  1.00  0.00           H
ATOM    266  CB  TYR    16      10.058  12.481  -0.274  1.00  0.00           C
ATOM    267  HB2 TYR    16      10.510  13.410  -0.623  1.00  0.00           H
ATOM    268  HB3 TYR    16      10.324  12.233   0.754  1.00  0.00           H
ATOM    269  CG  TYR    16       8.568  12.655  -0.389  1.00  0.00           C
ATOM    270  CD1 TYR    16       7.754  12.107   0.564  1.00  0.00           C
ATOM    271  HD1 TYR    16       8.159  11.410   1.284  1.00  0.00           H
ATOM    272  CE1 TYR    16       6.391  12.567   0.601  1.00  0.00           C
ATOM    273  HE1 TYR    16       5.740  12.256   1.405  1.00  0.00           H
ATOM    274  CZ  TYR    16       5.875  13.497  -0.376  1.00  0.00           C
ATOM    275  OH  TYR    16       4.535  13.860  -0.303  1.00  0.00           O
ATOM    276  HH  TYR    16       4.359  14.550  -0.946  1.00  0.00           H
ATOM    277  CE2 TYR    16       6.725  14.007  -1.326  1.00  0.00           C
ATOM    278  HE2 TYR    16       6.369  14.650  -2.118  1.00  0.00           H
ATOM    279  CD2 TYR    16       8.068  13.554  -1.374  1.00  0.00           C
ATOM    280  HD2 TYR    16       8.740  13.976  -2.106  1.00  0.00           H
ATOM    281  C   TYR    16      12.078  11.667  -1.496  1.00  0.00           C
ATOM    282  O   TYR    16      13.021  11.057  -0.998  1.00  0.00           O
ATOM    283  N   NME    17      12.272  12.749  -2.316  1.00  0.00           N
ATOM    284  H   NME    17      11.481  13.157  -2.793  1.00  0.00           H
ATOM    285  CH3 NME    17      13.594  13.090  -2.648  1.00  0.00           C
ATOM    286 HH31 NME    17      14.208  13.358  -1.787  1.00  0.00           H
ATOM    287 HH32 NME    17      14.054  12.225  -3.126  1.00  0.00           H
ATOM    288 HH33 NME    17      13.707  13.841  -3.430  1.00  0.00           H
TER     289      NME    17
END
```

Cluster 4:

```
ATOM      1 HH31 ACE     1      17.839  -1.284  11.080  1.00  0.00           H
```

ATOM	2	CH3	ACE	1	17.281	-1.626	11.952	1.00	0.00	C
ATOM	3	HH32	ACE	1	17.996	-2.227	12.515	1.00	0.00	H
ATOM	4	HH33	ACE	1	16.373	-2.205	11.786	1.00	0.00	H
ATOM	5	C	ACE	1	16.945	-0.400	12.812	1.00	0.00	C
ATOM	6	O	ACE	1	15.804	-0.387	13.279	1.00	0.00	O
ATOM	7	N	ASN	2	17.845	0.580	13.018	1.00	0.00	N
ATOM	8	H	ASN	2	18.725	0.580	12.522	1.00	0.00	H
ATOM	9	CA	ASN	2	17.534	1.877	13.710	1.00	0.00	C
ATOM	10	HA	ASN	2	17.128	1.697	14.705	1.00	0.00	H
ATOM	11	CB	ASN	2	18.857	2.721	13.892	1.00	0.00	C
ATOM	12	HB2	ASN	2	19.412	2.774	12.955	1.00	0.00	H
ATOM	13	HB3	ASN	2	19.240	2.133	14.726	1.00	0.00	H
ATOM	14	CG	ASN	2	18.554	4.167	14.402	1.00	0.00	C
ATOM	15	OD1	ASN	2	17.702	4.412	15.277	1.00	0.00	O
ATOM	16	ND2	ASN	2	19.499	5.090	14.153	1.00	0.00	N
ATOM	17	HD21	ASN	2	19.212	6.006	14.466	1.00	0.00	H
ATOM	18	HD22	ASN	2	20.290	4.937	13.544	1.00	0.00	H
ATOM	19	C	ASN	2	16.505	2.724	12.897	1.00	0.00	C
ATOM	20	O	ASN	2	16.734	3.033	11.721	1.00	0.00	O
ATOM	21	N	ASP	3	15.386	3.000	13.636	1.00	0.00	N
ATOM	22	H	ASP	3	15.307	2.671	14.588	1.00	0.00	H
ATOM	23	CA	ASP	3	14.145	3.584	13.003	1.00	0.00	C
ATOM	24	HA	ASP	3	13.715	2.812	12.366	1.00	0.00	H
ATOM	25	CB	ASP	3	13.178	4.005	14.174	1.00	0.00	C
ATOM	26	HB2	ASP	3	13.633	4.872	14.654	1.00	0.00	H
ATOM	27	HB3	ASP	3	13.190	3.119	14.808	1.00	0.00	H
ATOM	28	CG	ASP	3	11.807	4.314	13.805	1.00	0.00	C
ATOM	29	OD1	ASP	3	11.143	3.640	13.036	1.00	0.00	O
ATOM	30	OD2	ASP	3	11.404	5.447	14.186	1.00	0.00	O
ATOM	31	C	ASP	3	14.383	4.849	12.197	1.00	0.00	C
ATOM	32	O	ASP	3	13.796	5.025	11.093	1.00	0.00	O
ATOM	33	N	TYR	4	15.438	5.622	12.542	1.00	0.00	N
ATOM	34	H	TYR	4	15.960	5.472	13.394	1.00	0.00	H
ATOM	35	CA	TYR	4	15.761	6.851	11.834	1.00	0.00	C
ATOM	36	HA	TYR	4	14.803	7.345	11.669	1.00	0.00	H
ATOM	37	CB	TYR	4	16.540	7.779	12.667	1.00	0.00	C
ATOM	38	HB2	TYR	4	16.824	8.609	12.020	1.00	0.00	H
ATOM	39	HB3	TYR	4	17.454	7.254	12.943	1.00	0.00	H
ATOM	40	CG	TYR	4	15.948	8.348	13.920	1.00	0.00	C
ATOM	41	CD1	TYR	4	15.802	9.738	14.143	1.00	0.00	C
ATOM	42	HD1	TYR	4	15.921	10.475	13.363	1.00	0.00	H
ATOM	43	CE1	TYR	4	15.157	10.165	15.304	1.00	0.00	C
ATOM	44	HE1	TYR	4	15.129	11.234	15.455	1.00	0.00	H
ATOM	45	CZ	TYR	4	14.750	9.315	16.359	1.00	0.00	C
ATOM	46	OH	TYR	4	14.367	9.806	17.576	1.00	0.00	O
ATOM	47	HH	TYR	4	14.127	10.732	17.652	1.00	0.00	H
ATOM	48	CE2	TYR	4	15.138	8.000	16.266	1.00	0.00	C
ATOM	49	HE2	TYR	4	15.070	7.427	17.179	1.00	0.00	H
ATOM	50	CD2	TYR	4	15.705	7.468	15.044	1.00	0.00	C
ATOM	51	HD2	TYR	4	16.129	6.474	15.072	1.00	0.00	H
ATOM	52	C	TYR	4	16.442	6.640	10.467	1.00	0.00	C
ATOM	53	O	TYR	4	16.816	7.577	9.794	1.00	0.00	O
ATOM	54	N	GLU	5	16.618	5.365	10.008	1.00	0.00	N
ATOM	55	H	GLU	5	16.298	4.646	10.641	1.00	0.00	H
ATOM	56	CA	GLU	5	16.992	4.986	8.652	1.00	0.00	C
ATOM	57	HA	GLU	5	17.082	5.831	7.970	1.00	0.00	H
ATOM	58	CB	GLU	5	18.365	4.279	8.750	1.00	0.00	C
ATOM	59	HB2	GLU	5	18.387	3.392	9.383	1.00	0.00	H
ATOM	60	HB3	GLU	5	18.990	5.064	9.177	1.00	0.00	H
ATOM	61	CG	GLU	5	18.977	3.671	7.368	1.00	0.00	C
ATOM	62	HG2	GLU	5	18.560	4.178	6.497	1.00	0.00	H
ATOM	63	HG3	GLU	5	18.620	2.643	7.312	1.00	0.00	H
ATOM	64	CD	GLU	5	20.514	3.663	7.356	1.00	0.00	C

ATOM	65	OE1	GLU	5	21.082	4.520	6.663	1.00	0.00	O
ATOM	66	OE2	GLU	5	21.111	2.710	7.973	1.00	0.00	O
ATOM	67	C	GLU	5	15.960	4.172	7.983	1.00	0.00	C
ATOM	68	O	GLU	5	15.619	4.394	6.840	1.00	0.00	O
ATOM	69	N	ASP	6	15.317	3.258	8.725	1.00	0.00	N
ATOM	70	H	ASP	6	15.852	3.067	9.561	1.00	0.00	H
ATOM	71	CA	ASP	6	14.230	2.404	8.254	1.00	0.00	C
ATOM	72	HA	ASP	6	14.669	1.801	7.459	1.00	0.00	H
ATOM	73	CB	ASP	6	13.865	1.455	9.383	1.00	0.00	C
ATOM	74	HB2	ASP	6	13.603	2.060	10.252	1.00	0.00	H
ATOM	75	HB3	ASP	6	14.747	0.825	9.499	1.00	0.00	H
ATOM	76	CG	ASP	6	12.721	0.421	9.069	1.00	0.00	C
ATOM	77	OD1	ASP	6	12.830	-0.449	8.216	1.00	0.00	O
ATOM	78	OD2	ASP	6	11.842	0.353	10.012	1.00	0.00	O
ATOM	79	C	ASP	6	12.935	3.156	7.802	1.00	0.00	C
ATOM	80	O	ASP	6	12.262	2.650	6.919	1.00	0.00	O
ATOM	81	N	ARG	7	12.711	4.350	8.253	1.00	0.00	N
ATOM	82	H	ARG	7	13.384	4.683	8.928	1.00	0.00	H
ATOM	83	CA	ARG	7	11.477	5.145	8.039	1.00	0.00	C
ATOM	84	HA	ARG	7	10.634	4.462	8.142	1.00	0.00	H
ATOM	85	CB	ARG	7	11.256	6.151	9.208	1.00	0.00	C
ATOM	86	HB2	ARG	7	11.363	5.667	10.179	1.00	0.00	H
ATOM	87	HB3	ARG	7	10.267	6.581	9.052	1.00	0.00	H
ATOM	88	CG	ARG	7	12.195	7.332	9.174	1.00	0.00	C
ATOM	89	HG2	ARG	7	12.140	7.846	8.214	1.00	0.00	H
ATOM	90	HG3	ARG	7	13.247	7.061	9.267	1.00	0.00	H
ATOM	91	CD	ARG	7	12.096	8.554	10.066	1.00	0.00	C
ATOM	92	HD2	ARG	7	11.159	9.108	10.017	1.00	0.00	H
ATOM	93	HD3	ARG	7	12.925	9.157	9.695	1.00	0.00	H
ATOM	94	NE	ARG	7	12.114	8.112	11.491	1.00	0.00	N
ATOM	95	HE	ARG	7	11.928	7.128	11.624	1.00	0.00	H
ATOM	96	CZ	ARG	7	12.463	8.726	12.603	1.00	0.00	C
ATOM	97	NH1	ARG	7	12.837	10.004	12.625	1.00	0.00	N
ATOM	98	HH11	ARG	7	12.982	10.342	11.684	1.00	0.00	H
ATOM	99	HH12	ARG	7	12.789	10.640	13.408	1.00	0.00	H
ATOM	100	NH2	ARG	7	12.370	8.070	13.738	1.00	0.00	N
ATOM	101	HH21	ARG	7	12.650	8.505	14.606	1.00	0.00	H
ATOM	102	HH22	ARG	7	11.987	7.139	13.819	1.00	0.00	H
ATOM	103	C	ARG	7	11.387	5.582	6.574	1.00	0.00	C
ATOM	104	O	ARG	7	10.375	6.013	6.172	1.00	0.00	O
ATOM	105	N	TYR	8	12.468	5.535	5.794	1.00	0.00	N
ATOM	106	H	TYR	8	13.339	5.291	6.242	1.00	0.00	H
ATOM	107	CA	TYR	8	12.603	5.940	4.368	1.00	0.00	C
ATOM	108	HA	TYR	8	11.801	6.652	4.172	1.00	0.00	H
ATOM	109	CB	TYR	8	13.882	6.679	4.061	1.00	0.00	C
ATOM	110	HB2	TYR	8	13.890	7.050	3.036	1.00	0.00	H
ATOM	111	HB3	TYR	8	14.535	5.809	4.136	1.00	0.00	H
ATOM	112	CG	TYR	8	14.200	7.880	4.969	1.00	0.00	C
ATOM	113	CD1	TYR	8	13.408	9.080	4.861	1.00	0.00	C
ATOM	114	HD1	TYR	8	12.736	9.123	4.017	1.00	0.00	H
ATOM	115	CE1	TYR	8	13.489	10.090	5.824	1.00	0.00	C
ATOM	116	HE1	TYR	8	12.803	10.899	5.618	1.00	0.00	H
ATOM	117	CZ	TYR	8	14.561	10.096	6.753	1.00	0.00	C
ATOM	118	OH	TYR	8	14.760	11.091	7.679	1.00	0.00	O
ATOM	119	HH	TYR	8	14.134	11.806	7.541	1.00	0.00	H
ATOM	120	CE2	TYR	8	15.484	9.027	6.745	1.00	0.00	C
ATOM	121	HE2	TYR	8	16.340	9.095	7.399	1.00	0.00	H
ATOM	122	CD2	TYR	8	15.248	7.867	5.953	1.00	0.00	C
ATOM	123	HD2	TYR	8	15.828	6.961	6.054	1.00	0.00	H
ATOM	124	C	TYR	8	12.284	4.740	3.449	1.00	0.00	C
ATOM	125	O	TYR	8	11.745	4.911	2.406	1.00	0.00	O
ATOM	126	N	TYR	9	12.738	3.570	3.884	1.00	0.00	N
ATOM	127	H	TYR	9	13.183	3.533	4.790	1.00	0.00	H

ATOM	128	CA	TYR	9	12.677	2.318	3.050	1.00	0.00	C
ATOM	129	HA	TYR	9	12.745	2.598	1.999	1.00	0.00	H
ATOM	130	CB	TYR	9	13.938	1.454	3.222	1.00	0.00	C
ATOM	131	HB2	TYR	9	13.873	0.578	2.577	1.00	0.00	H
ATOM	132	HB3	TYR	9	13.784	1.117	4.247	1.00	0.00	H
ATOM	133	CG	TYR	9	15.293	2.100	2.947	1.00	0.00	C
ATOM	134	CD1	TYR	9	15.520	2.498	1.615	1.00	0.00	C
ATOM	135	HD1	TYR	9	14.773	2.303	0.860	1.00	0.00	H
ATOM	136	CE1	TYR	9	16.790	3.029	1.219	1.00	0.00	C
ATOM	137	HE1	TYR	9	16.983	3.187	0.169	1.00	0.00	H
ATOM	138	CZ	TYR	9	17.799	3.168	2.209	1.00	0.00	C
ATOM	139	OH	TYR	9	19.025	3.495	1.825	1.00	0.00	O
ATOM	140	HH	TYR	9	19.574	3.582	2.607	1.00	0.00	H
ATOM	141	CE2	TYR	9	17.483	2.953	3.546	1.00	0.00	C
ATOM	142	HE2	TYR	9	18.263	3.102	4.277	1.00	0.00	H
ATOM	143	CD2	TYR	9	16.243	2.391	3.935	1.00	0.00	C
ATOM	144	HD2	TYR	9	16.234	2.042	4.957	1.00	0.00	H
ATOM	145	C	TYR	9	11.448	1.474	3.103	1.00	0.00	C
ATOM	146	O	TYR	9	11.038	0.904	2.095	1.00	0.00	O
ATOM	147	N	ARG	10	10.768	1.384	4.301	1.00	0.00	N
ATOM	148	H	ARG	10	11.132	1.853	5.118	1.00	0.00	H
ATOM	149	CA	ARG	10	9.510	0.566	4.434	1.00	0.00	C
ATOM	150	HA	ARG	10	9.780	-0.446	4.133	1.00	0.00	H
ATOM	151	CB	ARG	10	9.284	0.301	5.976	1.00	0.00	C
ATOM	152	HB2	ARG	10	10.080	-0.211	6.515	1.00	0.00	H
ATOM	153	HB3	ARG	10	8.491	-0.441	6.069	1.00	0.00	H
ATOM	154	CG	ARG	10	8.793	1.541	6.813	1.00	0.00	C
ATOM	155	HG2	ARG	10	7.716	1.690	6.735	1.00	0.00	H
ATOM	156	HG3	ARG	10	9.290	2.443	6.456	1.00	0.00	H
ATOM	157	CD	ARG	10	9.060	1.538	8.276	1.00	0.00	C
ATOM	158	HD2	ARG	10	10.095	1.307	8.530	1.00	0.00	H
ATOM	159	HD3	ARG	10	8.346	0.807	8.654	1.00	0.00	H
ATOM	160	NE	ARG	10	8.682	2.808	8.921	1.00	0.00	N
ATOM	161	HE	ARG	10	7.827	3.233	8.593	1.00	0.00	H
ATOM	162	CZ	ARG	10	9.097	3.217	10.117	1.00	0.00	C
ATOM	163	NH1	ARG	10	10.063	2.661	10.746	1.00	0.00	N
ATOM	164	HH11	ARG	10	10.603	1.874	10.417	1.00	0.00	H
ATOM	165	HH12	ARG	10	10.365	3.057	11.625	1.00	0.00	H
ATOM	166	NH2	ARG	10	8.556	4.268	10.651	1.00	0.00	N
ATOM	167	HH21	ARG	10	7.805	4.855	10.317	1.00	0.00	H
ATOM	168	HH22	ARG	10	9.133	4.490	11.449	1.00	0.00	H
ATOM	169	C	ARG	10	8.273	1.165	3.757	1.00	0.00	C
ATOM	170	O	ARG	10	8.357	2.359	3.345	1.00	0.00	O
ATOM	171	N	GLU	11	7.144	0.485	3.806	1.00	0.00	N
ATOM	172	H	GLU	11	7.155	-0.464	4.153	1.00	0.00	H
ATOM	173	CA	GLU	11	5.965	1.086	3.181	1.00	0.00	C
ATOM	174	HA	GLU	11	6.202	1.374	2.157	1.00	0.00	H
ATOM	175	CB	GLU	11	4.901	0.017	3.129	1.00	0.00	C
ATOM	176	HB2	GLU	11	4.578	-0.327	4.112	1.00	0.00	H
ATOM	177	HB3	GLU	11	5.436	-0.792	2.631	1.00	0.00	H
ATOM	178	CG	GLU	11	3.665	0.333	2.248	1.00	0.00	C
ATOM	179	HG2	GLU	11	3.931	0.845	1.323	1.00	0.00	H
ATOM	180	HG3	GLU	11	3.159	1.017	2.929	1.00	0.00	H
ATOM	181	CD	GLU	11	2.740	-0.876	1.915	1.00	0.00	C
ATOM	182	OE1	GLU	11	3.005	-1.519	0.863	1.00	0.00	O
ATOM	183	OE2	GLU	11	1.705	-1.051	2.572	1.00	0.00	O
ATOM	184	C	GLU	11	5.454	2.371	3.979	1.00	0.00	C
ATOM	185	O	GLU	11	4.953	2.224	5.092	1.00	0.00	O
ATOM	186	N	ASN	12	5.521	3.552	3.385	1.00	0.00	N
ATOM	187	H	ASN	12	6.135	3.578	2.584	1.00	0.00	H
ATOM	188	CA	ASN	12	5.095	4.837	4.014	1.00	0.00	C
ATOM	189	HA	ASN	12	4.450	4.599	4.859	1.00	0.00	H
ATOM	190	CB	ASN	12	6.435	5.493	4.452	1.00	0.00	C

ATOM	191	HB2	ASN	12	7.054	4.777	4.992	1.00	0.00	H
ATOM	192	HB3	ASN	12	6.090	6.287	5.115	1.00	0.00	H
ATOM	193	CG	ASN	12	7.261	6.141	3.375	1.00	0.00	C
ATOM	194	OD1	ASN	12	6.892	7.207	2.861	1.00	0.00	O
ATOM	195	ND2	ASN	12	8.425	5.630	3.135	1.00	0.00	N
ATOM	196	HD21	ASN	12	9.058	6.075	2.486	1.00	0.00	H
ATOM	197	HD22	ASN	12	8.738	4.806	3.628	1.00	0.00	H
ATOM	198	C	ASN	12	4.281	5.809	3.117	1.00	0.00	C
ATOM	199	O	ASN	12	4.377	5.664	1.902	1.00	0.00	O
ATOM	200	N	MET	13	3.447	6.742	3.650	1.00	0.00	N
ATOM	201	H	MET	13	3.444	6.889	4.649	1.00	0.00	H
ATOM	202	CA	MET	13	2.802	7.743	2.814	1.00	0.00	C
ATOM	203	HA	MET	13	2.364	7.162	2.002	1.00	0.00	H
ATOM	204	CB	MET	13	1.666	8.409	3.629	1.00	0.00	C
ATOM	205	HB2	MET	13	0.963	8.793	2.890	1.00	0.00	H
ATOM	206	HB3	MET	13	2.174	9.133	4.266	1.00	0.00	H
ATOM	207	CG	MET	13	0.832	7.548	4.604	1.00	0.00	C
ATOM	208	HG2	MET	13	1.434	6.744	5.029	1.00	0.00	H
ATOM	209	HG3	MET	13	0.050	7.192	3.933	1.00	0.00	H
ATOM	210	SD	MET	13	-0.017	8.396	5.964	1.00	0.00	S
ATOM	211	CE	MET	13	-1.031	7.151	6.829	1.00	0.00	C
ATOM	212	HE1	MET	13	-0.460	6.418	7.398	1.00	0.00	H
ATOM	213	HE2	MET	13	-1.721	7.727	7.445	1.00	0.00	H
ATOM	214	HE3	MET	13	-1.518	6.594	6.028	1.00	0.00	H
ATOM	215	C	MET	13	3.608	8.885	2.144	1.00	0.00	C
ATOM	216	O	MET	13	3.198	9.399	1.096	1.00	0.00	O
ATOM	217	N	TYR	14	4.727	9.281	2.749	1.00	0.00	N
ATOM	218	H	TYR	14	4.957	8.872	3.643	1.00	0.00	H
ATOM	219	CA	TYR	14	5.527	10.475	2.387	1.00	0.00	C
ATOM	220	HA	TYR	14	4.844	11.304	2.199	1.00	0.00	H
ATOM	221	CB	TYR	14	6.324	10.721	3.608	1.00	0.00	C
ATOM	222	HB2	TYR	14	6.770	11.709	3.496	1.00	0.00	H
ATOM	223	HB3	TYR	14	7.126	9.984	3.569	1.00	0.00	H
ATOM	224	CG	TYR	14	5.747	10.602	4.955	1.00	0.00	C
ATOM	225	CD1	TYR	14	5.867	9.498	5.731	1.00	0.00	C
ATOM	226	HD1	TYR	14	6.536	8.707	5.423	1.00	0.00	H
ATOM	227	CE1	TYR	14	5.363	9.421	7.043	1.00	0.00	C
ATOM	228	HE1	TYR	14	5.656	8.600	7.681	1.00	0.00	H
ATOM	229	CZ	TYR	14	4.534	10.503	7.519	1.00	0.00	C
ATOM	230	OH	TYR	14	4.180	10.427	8.830	1.00	0.00	O
ATOM	231	HH	TYR	14	4.605	9.742	9.352	1.00	0.00	H
ATOM	232	CE2	TYR	14	4.237	11.584	6.651	1.00	0.00	C
ATOM	233	HE2	TYR	14	3.563	12.406	6.840	1.00	0.00	H
ATOM	234	CD2	TYR	14	4.851	11.637	5.384	1.00	0.00	C
ATOM	235	HD2	TYR	14	4.574	12.368	4.638	1.00	0.00	H
ATOM	236	C	TYR	14	6.333	10.309	1.062	1.00	0.00	C
ATOM	237	O	TYR	14	6.396	11.279	0.349	1.00	0.00	O
ATOM	238	N	ARG	15	6.780	9.084	0.727	1.00	0.00	N
ATOM	239	H	ARG	15	6.532	8.301	1.315	1.00	0.00	H
ATOM	240	CA	ARG	15	7.632	8.780	-0.396	1.00	0.00	C
ATOM	241	HA	ARG	15	8.534	9.380	-0.276	1.00	0.00	H
ATOM	242	CB	ARG	15	8.167	7.301	-0.326	1.00	0.00	C
ATOM	243	HB2	ARG	15	8.803	7.100	0.536	1.00	0.00	H
ATOM	244	HB3	ARG	15	8.768	7.171	-1.226	1.00	0.00	H
ATOM	245	CG	ARG	15	7.125	6.111	-0.424	1.00	0.00	C
ATOM	246	HG2	ARG	15	6.454	6.146	-1.282	1.00	0.00	H
ATOM	247	HG3	ARG	15	6.553	6.168	0.502	1.00	0.00	H
ATOM	248	CD	ARG	15	7.813	4.718	-0.538	1.00	0.00	C
ATOM	249	HD2	ARG	15	8.434	4.445	0.315	1.00	0.00	H
ATOM	250	HD3	ARG	15	8.440	4.775	-1.428	1.00	0.00	H
ATOM	251	NE	ARG	15	6.788	3.685	-0.715	1.00	0.00	N
ATOM	252	HE	ARG	15	5.867	4.015	-0.964	1.00	0.00	H
ATOM	253	CZ	ARG	15	6.869	2.372	-0.626	1.00	0.00	C

```
ATOM    254  NH1 ARG    15       7.965   1.773  -0.233  1.00  0.00           N
ATOM    255 HH11 ARG    15       8.007   0.766  -0.172  1.00  0.00           H
ATOM    256 HH12 ARG    15       8.816   2.311  -0.145  1.00  0.00           H
ATOM    257  NH2 ARG    15       5.828   1.647  -0.958  1.00  0.00           N
ATOM    258 HH21 ARG    15       5.178   2.196  -1.503  1.00  0.00           H
ATOM    259 HH22 ARG    15       6.024   0.693  -1.223  1.00  0.00           H
ATOM    260  C   ARG    15       6.961   9.063  -1.748  1.00  0.00           C
ATOM    261  O   ARG    15       7.619   9.520  -2.723  1.00  0.00           O
ATOM    262  N   TYR    16       5.664   8.727  -1.908  1.00  0.00           N
ATOM    263  H   TYR    16       5.294   8.199  -1.131  1.00  0.00           H
ATOM    264  CA  TYR    16       4.820   8.842  -3.119  1.00  0.00           C
ATOM    265  HA  TYR    16       5.326   8.313  -3.927  1.00  0.00           H
ATOM    266  CB  TYR    16       3.562   8.044  -3.003  1.00  0.00           C
ATOM    267  HB2 TYR    16       3.035   8.238  -3.937  1.00  0.00           H
ATOM    268  HB3 TYR    16       2.942   8.389  -2.175  1.00  0.00           H
ATOM    269  CG  TYR    16       3.631   6.524  -2.944  1.00  0.00           C
ATOM    270  CD1 TYR    16       4.053   5.861  -4.103  1.00  0.00           C
ATOM    271  HD1 TYR    16       4.210   6.374  -5.040  1.00  0.00           H
ATOM    272  CE1 TYR    16       3.983   4.455  -4.021  1.00  0.00           C
ATOM    273  HE1 TYR    16       4.299   3.836  -4.848  1.00  0.00           H
ATOM    274  CZ  TYR    16       3.624   3.815  -2.821  1.00  0.00           C
ATOM    275  OH  TYR    16       3.543   2.413  -2.731  1.00  0.00           O
ATOM    276  HH  TYR    16       3.372   2.053  -3.604  1.00  0.00           H
ATOM    277  CE2 TYR    16       3.200   4.502  -1.659  1.00  0.00           C
ATOM    278  HE2 TYR    16       2.760   3.961  -0.835  1.00  0.00           H
ATOM    279  CD2 TYR    16       3.242   5.902  -1.772  1.00  0.00           C
ATOM    280  HD2 TYR    16       2.783   6.487  -0.989  1.00  0.00           H
ATOM    281  C   TYR    16       4.539  10.322  -3.460  1.00  0.00           C
ATOM    282  O   TYR    16       4.386  10.617  -4.660  1.00  0.00           O
ATOM    283  N   NME    17       4.315  11.204  -2.495  1.00  0.00           N
ATOM    284  H   NME    17       4.197  10.867  -1.550  1.00  0.00           H
ATOM    285  CH3 NME    17       4.042  12.644  -2.634  1.00  0.00           C
ATOM    286 HH31 NME    17       3.004  12.747  -2.953  1.00  0.00           H
ATOM    287 HH32 NME    17       4.209  13.086  -1.652  1.00  0.00           H
ATOM    288 HH33 NME    17       4.684  13.141  -3.361  1.00  0.00           H
TER     289      NME    17
END
```

Cluster 5, Figure 2A:

```
ATOM      1 HH31 ACE     1      19.414  -4.695   6.674  1.00  0.00           H
ATOM      2  CH3 ACE     1      18.422  -4.257   6.564  1.00  0.00           C
ATOM      3 HH32 ACE     1      17.870  -4.165   7.499  1.00  0.00           H
ATOM      4 HH33 ACE     1      17.820  -4.964   5.993  1.00  0.00           H
ATOM      5  C   ACE     1      18.563  -3.012   5.754  1.00  0.00           C
ATOM      6  O   ACE     1      19.398  -2.229   6.143  1.00  0.00           O
ATOM      7  N   ASN     2      17.843  -2.841   4.697  1.00  0.00           N
ATOM      8  H   ASN     2      17.142  -3.525   4.449  1.00  0.00           H
ATOM      9  CA  ASN     2      18.046  -1.752   3.751  1.00  0.00           C
ATOM     10  HA  ASN     2      19.031  -1.353   3.997  1.00  0.00           H
ATOM     11  CB  ASN     2      18.152  -2.371   2.364  1.00  0.00           C
ATOM     12  HB2 ASN     2      17.216  -2.778   1.982  1.00  0.00           H
ATOM     13  HB3 ASN     2      18.901  -3.150   2.502  1.00  0.00           H
ATOM     14  CG  ASN     2      18.549  -1.329   1.357  1.00  0.00           C
ATOM     15  OD1 ASN     2      19.521  -0.656   1.603  1.00  0.00           O
ATOM     16  ND2 ASN     2      18.047  -1.290   0.124  1.00  0.00           N
ATOM     17 HD21 ASN     2      18.468  -0.681  -0.562  1.00  0.00           H
ATOM     18 HD22 ASN     2      17.454  -2.056  -0.162  1.00  0.00           H
ATOM     19  C   ASN     2      16.965  -0.681   3.938  1.00  0.00           C
ATOM     20  O   ASN     2      16.797   0.154   3.037  1.00  0.00           O
ATOM     21  N   ASP     3      16.146  -0.686   5.026  1.00  0.00           N
```

ATOM	22	H	ASP	3	16.269	-1.368	5.760	1.00	0.00	H
ATOM	23	CA	ASP	3	15.016	0.230	5.234	1.00	0.00	C
ATOM	24	HA	ASP	3	15.057	0.855	4.342	1.00	0.00	H
ATOM	25	CB	ASP	3	13.728	-0.671	5.292	1.00	0.00	C
ATOM	26	HB2	ASP	3	13.772	-1.296	6.186	1.00	0.00	H
ATOM	27	HB3	ASP	3	13.810	-1.253	4.374	1.00	0.00	H
ATOM	28	CG	ASP	3	12.491	0.188	5.341	1.00	0.00	C
ATOM	29	OD1	ASP	3	11.565	-0.085	6.119	1.00	0.00	O
ATOM	30	OD2	ASP	3	12.388	1.257	4.698	1.00	0.00	O
ATOM	31	C	ASP	3	15.166	1.078	6.448	1.00	0.00	C
ATOM	32	O	ASP	3	14.243	1.347	7.260	1.00	0.00	O
ATOM	33	N	TYR	4	16.370	1.624	6.658	1.00	0.00	N
ATOM	34	H	TYR	4	17.177	1.423	6.086	1.00	0.00	H
ATOM	35	CA	TYR	4	16.549	2.667	7.658	1.00	0.00	C
ATOM	36	HA	TYR	4	16.408	2.182	8.624	1.00	0.00	H
ATOM	37	CB	TYR	4	17.985	3.068	7.569	1.00	0.00	C
ATOM	38	HB2	TYR	4	18.314	3.258	6.547	1.00	0.00	H
ATOM	39	HB3	TYR	4	18.638	2.245	7.860	1.00	0.00	H
ATOM	40	CG	TYR	4	18.245	4.369	8.338	1.00	0.00	C
ATOM	41	CD1	TYR	4	18.359	4.262	9.749	1.00	0.00	C
ATOM	42	HD1	TYR	4	18.216	3.335	10.284	1.00	0.00	H
ATOM	43	CE1	TYR	4	18.820	5.316	10.550	1.00	0.00	C
ATOM	44	HE1	TYR	4	18.956	5.151	11.608	1.00	0.00	H
ATOM	45	CZ	TYR	4	19.065	6.520	9.893	1.00	0.00	C
ATOM	46	OH	TYR	4	19.358	7.705	10.530	1.00	0.00	O
ATOM	47	HH	TYR	4	19.220	7.499	11.458	1.00	0.00	H
ATOM	48	CE2	TYR	4	18.875	6.624	8.501	1.00	0.00	C
ATOM	49	HE2	TYR	4	19.052	7.601	8.076	1.00	0.00	H
ATOM	50	CD2	TYR	4	18.428	5.588	7.706	1.00	0.00	C
ATOM	51	HD2	TYR	4	18.430	5.735	6.636	1.00	0.00	H
ATOM	52	C	TYR	4	15.599	3.845	7.451	1.00	0.00	C
ATOM	53	O	TYR	4	15.100	4.041	6.297	1.00	0.00	O
ATOM	54	N	GLU	5	15.302	4.556	8.510	1.00	0.00	N
ATOM	55	H	GLU	5	15.826	4.259	9.322	1.00	0.00	H
ATOM	56	CA	GLU	5	14.278	5.578	8.640	1.00	0.00	C
ATOM	57	HA	GLU	5	14.164	5.953	7.622	1.00	0.00	H
ATOM	58	CB	GLU	5	12.928	4.961	9.169	1.00	0.00	C
ATOM	59	HB2	GLU	5	13.043	4.518	10.158	1.00	0.00	H
ATOM	60	HB3	GLU	5	12.786	4.197	8.403	1.00	0.00	H
ATOM	61	CG	GLU	5	11.818	6.020	9.252	1.00	0.00	C
ATOM	62	HG2	GLU	5	12.027	6.785	9.999	1.00	0.00	H
ATOM	63	HG3	GLU	5	10.924	5.480	9.561	1.00	0.00	H
ATOM	64	CD	GLU	5	11.555	6.707	7.910	1.00	0.00	C
ATOM	65	OE1	GLU	5	12.164	7.789	7.643	1.00	0.00	O
ATOM	66	OE2	GLU	5	10.808	6.167	7.020	1.00	0.00	O
ATOM	67	C	GLU	5	14.808	6.611	9.515	1.00	0.00	C
ATOM	68	O	GLU	5	15.508	6.333	10.518	1.00	0.00	O
ATOM	69	N	ASP	6	14.506	7.890	9.176	1.00	0.00	N
ATOM	70	H	ASP	6	13.859	7.983	8.406	1.00	0.00	H
ATOM	71	CA	ASP	6	14.947	9.088	9.904	1.00	0.00	C
ATOM	72	HA	ASP	6	15.414	8.782	10.841	1.00	0.00	H
ATOM	73	CB	ASP	6	15.964	9.848	9.053	1.00	0.00	C
ATOM	74	HB2	ASP	6	16.603	9.085	8.608	1.00	0.00	H
ATOM	75	HB3	ASP	6	16.476	10.390	9.847	1.00	0.00	H
ATOM	76	CG	ASP	6	15.403	10.792	8.036	1.00	0.00	C
ATOM	77	OD1	ASP	6	14.952	11.837	8.453	1.00	0.00	O
ATOM	78	OD2	ASP	6	15.173	10.420	6.869	1.00	0.00	O
ATOM	79	C	ASP	6	13.791	9.886	10.500	1.00	0.00	C
ATOM	80	O	ASP	6	13.991	10.647	11.422	1.00	0.00	O
ATOM	81	N	ARG	7	12.604	9.681	9.907	1.00	0.00	N
ATOM	82	H	ARG	7	12.493	9.052	9.123	1.00	0.00	H
ATOM	83	CA	ARG	7	11.445	10.541	10.146	1.00	0.00	C
ATOM	84	HA	ARG	7	11.718	11.580	10.337	1.00	0.00	H

ATOM	85	CB	ARG	7	10.679	10.444	8.814	1.00	0.00	C
ATOM	86	HB2	ARG	7	9.880	11.185	8.810	1.00	0.00	H
ATOM	87	HB3	ARG	7	10.368	9.399	8.799	1.00	0.00	H
ATOM	88	CG	ARG	7	11.492	10.923	7.630	1.00	0.00	C
ATOM	89	HG2	ARG	7	12.495	10.505	7.543	1.00	0.00	H
ATOM	90	HG3	ARG	7	11.611	11.997	7.779	1.00	0.00	H
ATOM	91	CD	ARG	7	10.860	10.689	6.246	1.00	0.00	C
ATOM	92	HD2	ARG	7	11.681	10.866	5.550	1.00	0.00	H
ATOM	93	HD3	ARG	7	10.138	11.504	6.213	1.00	0.00	H
ATOM	94	NE	ARG	7	10.389	9.299	6.094	1.00	0.00	N
ATOM	95	HE	ARG	7	10.761	8.661	6.782	1.00	0.00	H
ATOM	96	CZ	ARG	7	9.552	8.779	5.185	1.00	0.00	C
ATOM	97	NH1	ARG	7	9.002	9.461	4.199	1.00	0.00	N
ATOM	98	HH11	ARG	7	9.008	10.471	4.174	1.00	0.00	H
ATOM	99	HH12	ARG	7	8.317	8.956	3.656	1.00	0.00	H
ATOM	100	NH2	ARG	7	9.269	7.508	5.115	1.00	0.00	N
ATOM	101	HH21	ARG	7	9.726	6.849	5.729	1.00	0.00	H
ATOM	102	HH22	ARG	7	8.421	7.235	4.639	1.00	0.00	H
ATOM	103	C	ARG	7	10.537	10.127	11.327	1.00	0.00	C
ATOM	104	O	ARG	7	10.645	8.960	11.692	1.00	0.00	O
ATOM	105	N	TYR	8	9.701	10.983	11.922	1.00	0.00	N
ATOM	106	H	TYR	8	9.703	11.967	11.691	1.00	0.00	H
ATOM	107	CA	TYR	8	9.011	10.561	13.121	1.00	0.00	C
ATOM	108	HA	TYR	8	9.693	9.992	13.752	1.00	0.00	H
ATOM	109	CB	TYR	8	8.511	11.806	13.890	1.00	0.00	C
ATOM	110	HB2	TYR	8	7.865	11.415	14.676	1.00	0.00	H
ATOM	111	HB3	TYR	8	7.963	12.403	13.161	1.00	0.00	H
ATOM	112	CG	TYR	8	9.508	12.635	14.600	1.00	0.00	C
ATOM	113	CD1	TYR	8	10.299	12.090	15.645	1.00	0.00	C
ATOM	114	HD1	TYR	8	10.383	11.015	15.697	1.00	0.00	H
ATOM	115	CE1	TYR	8	11.072	12.993	16.402	1.00	0.00	C
ATOM	116	HE1	TYR	8	11.594	12.660	17.286	1.00	0.00	H
ATOM	117	CZ	TYR	8	11.096	14.398	16.167	1.00	0.00	C
ATOM	118	OH	TYR	8	11.882	15.226	16.993	1.00	0.00	O
ATOM	119	HH	TYR	8	11.984	16.116	16.649	1.00	0.00	H
ATOM	120	CE2	TYR	8	10.353	14.900	15.080	1.00	0.00	C
ATOM	121	HE2	TYR	8	10.361	15.969	14.928	1.00	0.00	H
ATOM	122	CD2	TYR	8	9.533	14.037	14.333	1.00	0.00	C
ATOM	123	HD2	TYR	8	8.929	14.444	13.537	1.00	0.00	H
ATOM	124	C	TYR	8	7.879	9.549	12.852	1.00	0.00	C
ATOM	125	O	TYR	8	7.403	8.837	13.751	1.00	0.00	O
ATOM	126	N	TYR	9	7.325	9.615	11.626	1.00	0.00	N
ATOM	127	H	TYR	9	7.706	10.308	10.999	1.00	0.00	H
ATOM	128	CA	TYR	9	6.341	8.661	11.132	1.00	0.00	C
ATOM	129	HA	TYR	9	6.576	7.734	11.653	1.00	0.00	H
ATOM	130	CB	TYR	9	4.888	9.031	11.552	1.00	0.00	C
ATOM	131	HB2	TYR	9	4.707	9.108	12.624	1.00	0.00	H
ATOM	132	HB3	TYR	9	4.340	8.124	11.300	1.00	0.00	H
ATOM	133	CG	TYR	9	4.293	10.288	10.964	1.00	0.00	C
ATOM	134	CD1	TYR	9	4.680	11.576	11.487	1.00	0.00	C
ATOM	135	HD1	TYR	9	5.145	11.639	12.460	1.00	0.00	H
ATOM	136	CE1	TYR	9	4.126	12.689	10.868	1.00	0.00	C
ATOM	137	HE1	TYR	9	4.458	13.692	11.089	1.00	0.00	H
ATOM	138	CZ	TYR	9	3.188	12.607	9.818	1.00	0.00	C
ATOM	139	OH	TYR	9	2.749	13.795	9.303	1.00	0.00	O
ATOM	140	HH	TYR	9	2.225	13.698	8.504	1.00	0.00	H
ATOM	141	CE2	TYR	9	2.884	11.329	9.254	1.00	0.00	C
ATOM	142	HE2	TYR	9	2.157	11.211	8.464	1.00	0.00	H
ATOM	143	CD2	TYR	9	3.454	10.176	9.832	1.00	0.00	C
ATOM	144	HD2	TYR	9	3.106	9.205	9.509	1.00	0.00	H
ATOM	145	C	TYR	9	6.575	8.524	9.608	1.00	0.00	C
ATOM	146	O	TYR	9	7.398	9.304	9.101	1.00	0.00	O
ATOM	147	N	ARG	10	5.850	7.620	8.913	1.00	0.00	N

ATOM	148	H	ARG	10	5.233	7.050	9.471	1.00	0.00	H
ATOM	149	CA	ARG	10	6.118	7.336	7.478	1.00	0.00	C
ATOM	150	HA	ARG	10	6.322	8.247	6.915	1.00	0.00	H
ATOM	151	CB	ARG	10	7.245	6.314	7.243	1.00	0.00	C
ATOM	152	HB2	ARG	10	8.216	6.693	7.566	1.00	0.00	H
ATOM	153	HB3	ARG	10	7.228	6.209	6.159	1.00	0.00	H
ATOM	154	CG	ARG	10	7.028	4.895	7.819	1.00	0.00	C
ATOM	155	HG2	ARG	10	5.966	4.682	7.942	1.00	0.00	H
ATOM	156	HG3	ARG	10	7.507	4.994	8.794	1.00	0.00	H
ATOM	157	CD	ARG	10	7.699	3.747	7.128	1.00	0.00	C
ATOM	158	HD2	ARG	10	7.136	3.640	6.201	1.00	0.00	H
ATOM	159	HD3	ARG	10	7.527	2.969	7.871	1.00	0.00	H
ATOM	160	NE	ARG	10	9.166	3.923	6.824	1.00	0.00	N
ATOM	161	HE	ARG	10	9.530	4.845	7.022	1.00	0.00	H
ATOM	162	CZ	ARG	10	9.979	3.005	6.335	1.00	0.00	C
ATOM	163	NH1	ARG	10	9.505	1.825	6.235	1.00	0.00	N
ATOM	164	HH11	ARG	10	8.529	1.567	6.268	1.00	0.00	H
ATOM	165	HH12	ARG	10	10.179	1.075	6.171	1.00	0.00	H
ATOM	166	NH2	ARG	10	11.215	3.284	6.082	1.00	0.00	N
ATOM	167	HH21	ARG	10	11.532	4.241	6.159	1.00	0.00	H
ATOM	168	HH22	ARG	10	11.761	2.556	5.645	1.00	0.00	H
ATOM	169	C	ARG	10	4.782	6.836	6.768	1.00	0.00	C
ATOM	170	O	ARG	10	3.944	6.250	7.415	1.00	0.00	O
ATOM	171	N	GLU	11	4.778	7.064	5.493	1.00	0.00	N
ATOM	172	H	GLU	11	5.689	7.291	5.121	1.00	0.00	H
ATOM	173	CA	GLU	11	3.774	6.535	4.632	1.00	0.00	C
ATOM	174	HA	GLU	11	2.851	6.588	5.208	1.00	0.00	H
ATOM	175	CB	GLU	11	3.550	7.417	3.372	1.00	0.00	C
ATOM	176	HB2	GLU	11	3.431	8.463	3.653	1.00	0.00	H
ATOM	177	HB3	GLU	11	2.732	6.888	2.883	1.00	0.00	H
ATOM	178	CG	GLU	11	4.720	7.562	2.408	1.00	0.00	C
ATOM	179	HG2	GLU	11	4.302	8.188	1.620	1.00	0.00	H
ATOM	180	HG3	GLU	11	4.952	6.581	1.994	1.00	0.00	H
ATOM	181	CD	GLU	11	5.997	8.282	2.922	1.00	0.00	C
ATOM	182	OE1	GLU	11	6.815	7.558	3.541	1.00	0.00	O
ATOM	183	OE2	GLU	11	6.107	9.529	2.823	1.00	0.00	O
ATOM	184	C	GLU	11	3.998	5.015	4.408	1.00	0.00	C
ATOM	185	O	GLU	11	5.108	4.511	4.553	1.00	0.00	O
ATOM	186	N	ASN	12	2.898	4.353	4.014	1.00	0.00	N
ATOM	187	H	ASN	12	2.023	4.857	4.048	1.00	0.00	H
ATOM	188	CA	ASN	12	2.767	2.869	3.896	1.00	0.00	C
ATOM	189	HA	ASN	12	3.372	2.510	4.728	1.00	0.00	H
ATOM	190	CB	ASN	12	1.311	2.413	4.139	1.00	0.00	C
ATOM	191	HB2	ASN	12	0.856	3.007	4.931	1.00	0.00	H
ATOM	192	HB3	ASN	12	1.221	1.388	4.501	1.00	0.00	H
ATOM	193	CG	ASN	12	0.477	2.617	2.931	1.00	0.00	C
ATOM	194	OD1	ASN	12	0.787	3.196	1.908	1.00	0.00	O
ATOM	195	ND2	ASN	12	-0.762	2.187	2.918	1.00	0.00	N
ATOM	196	HD21	ASN	12	-1.412	2.427	2.184	1.00	0.00	H
ATOM	197	HD22	ASN	12	-1.034	1.581	3.679	1.00	0.00	H
ATOM	198	C	ASN	12	3.473	2.254	2.683	1.00	0.00	C
ATOM	199	O	ASN	12	3.507	1.017	2.683	1.00	0.00	O
ATOM	200	N	MET	13	3.953	3.013	1.677	1.00	0.00	N
ATOM	201	H	MET	13	3.945	3.992	1.927	1.00	0.00	H
ATOM	202	CA	MET	13	4.854	2.572	0.604	1.00	0.00	C
ATOM	203	HA	MET	13	4.774	1.485	0.574	1.00	0.00	H
ATOM	204	CB	MET	13	4.282	3.136	-0.687	1.00	0.00	C
ATOM	205	HB2	MET	13	4.101	4.204	-0.569	1.00	0.00	H
ATOM	206	HB3	MET	13	3.364	2.586	-0.898	1.00	0.00	H
ATOM	207	CG	MET	13	5.090	2.907	-1.951	1.00	0.00	C
ATOM	208	HG2	MET	13	5.998	3.507	-1.888	1.00	0.00	H
ATOM	209	HG3	MET	13	4.459	3.301	-2.749	1.00	0.00	H
ATOM	210	SD	MET	13	5.717	1.268	-2.308	1.00	0.00	S

ATOM	211	CE	MET	13	7.071	1.761	-3.417	1.00	0.00	C
ATOM	212	HE1	MET	13	6.567	2.011	-4.350	1.00	0.00	H
ATOM	213	HE2	MET	13	7.627	2.649	-3.116	1.00	0.00	H
ATOM	214	HE3	MET	13	7.851	1.003	-3.494	1.00	0.00	H
ATOM	215	C	MET	13	6.310	3.032	0.969	1.00	0.00	C
ATOM	216	O	MET	13	6.648	4.224	1.074	1.00	0.00	O
ATOM	217	N	TYR	14	7.229	2.086	1.162	1.00	0.00	N
ATOM	218	H	TYR	14	6.952	1.130	0.990	1.00	0.00	H
ATOM	219	CA	TYR	14	8.659	2.311	1.450	1.00	0.00	C
ATOM	220	HA	TYR	14	8.647	3.074	2.228	1.00	0.00	H
ATOM	221	CB	TYR	14	9.194	1.029	1.948	1.00	0.00	C
ATOM	222	HB2	TYR	14	10.229	1.116	2.278	1.00	0.00	H
ATOM	223	HB3	TYR	14	9.388	0.359	1.110	1.00	0.00	H
ATOM	224	CG	TYR	14	8.348	0.325	3.069	1.00	0.00	C
ATOM	225	CD1	TYR	14	8.447	-1.066	3.243	1.00	0.00	C
ATOM	226	HD1	TYR	14	9.068	-1.716	2.646	1.00	0.00	H
ATOM	227	CE1	TYR	14	7.819	-1.730	4.266	1.00	0.00	C
ATOM	228	HE1	TYR	14	8.047	-2.780	4.372	1.00	0.00	H
ATOM	229	CZ	TYR	14	7.006	-1.058	5.201	1.00	0.00	C
ATOM	230	OH	TYR	14	6.434	-1.783	6.199	1.00	0.00	O
ATOM	231	HH	TYR	14	6.704	-2.696	6.080	1.00	0.00	H
ATOM	232	CE2	TYR	14	6.828	0.327	5.038	1.00	0.00	C
ATOM	233	HE2	TYR	14	6.151	0.886	5.667	1.00	0.00	H
ATOM	234	CD2	TYR	14	7.510	1.041	3.981	1.00	0.00	C
ATOM	235	HD2	TYR	14	7.424	2.112	3.870	1.00	0.00	H
ATOM	236	C	TYR	14	9.527	2.908	0.332	1.00	0.00	C
ATOM	237	O	TYR	14	9.189	2.852	-0.874	1.00	0.00	O
ATOM	238	N	ARG	15	10.663	3.532	0.783	1.00	0.00	N
ATOM	239	H	ARG	15	10.722	3.524	1.791	1.00	0.00	H
ATOM	240	CA	ARG	15	11.648	4.304	-0.038	1.00	0.00	C
ATOM	241	HA	ARG	15	11.203	5.253	-0.339	1.00	0.00	H
ATOM	242	CB	ARG	15	12.853	4.763	0.865	1.00	0.00	C
ATOM	243	HB2	ARG	15	13.573	5.328	0.273	1.00	0.00	H
ATOM	244	HB3	ARG	15	13.302	3.808	1.138	1.00	0.00	H
ATOM	245	CG	ARG	15	12.514	5.591	2.089	1.00	0.00	C
ATOM	246	HG2	ARG	15	11.554	5.350	2.545	1.00	0.00	H
ATOM	247	HG3	ARG	15	12.583	6.598	1.679	1.00	0.00	H
ATOM	248	CD	ARG	15	13.407	5.401	3.321	1.00	0.00	C
ATOM	249	HD2	ARG	15	14.444	5.330	2.993	1.00	0.00	H
ATOM	250	HD3	ARG	15	12.961	4.516	3.778	1.00	0.00	H
ATOM	251	NE	ARG	15	13.336	6.509	4.244	1.00	0.00	N
ATOM	252	HE	ARG	15	12.528	6.488	4.850	1.00	0.00	H
ATOM	253	CZ	ARG	15	14.296	7.394	4.567	1.00	0.00	C
ATOM	254	NH1	ARG	15	15.238	7.587	3.794	1.00	0.00	N
ATOM	255	HH11	ARG	15	15.235	7.191	2.865	1.00	0.00	H
ATOM	256	HH12	ARG	15	16.029	8.128	4.114	1.00	0.00	H
ATOM	257	NH2	ARG	15	14.096	8.099	5.628	1.00	0.00	N
ATOM	258	HH21	ARG	15	13.374	7.966	6.323	1.00	0.00	H
ATOM	259	HH22	ARG	15	14.558	8.985	5.773	1.00	0.00	H
ATOM	260	C	ARG	15	12.175	3.623	-1.242	1.00	0.00	C
ATOM	261	O	ARG	15	12.272	2.407	-1.280	1.00	0.00	O
ATOM	262	N	TYR	16	12.612	4.395	-2.223	1.00	0.00	N
ATOM	263	H	TYR	16	12.309	5.351	-2.103	1.00	0.00	H
ATOM	264	CA	TYR	16	13.318	3.932	-3.382	1.00	0.00	C
ATOM	265	HA	TYR	16	12.845	2.975	-3.601	1.00	0.00	H
ATOM	266	CB	TYR	16	13.001	4.822	-4.614	1.00	0.00	C
ATOM	267	HB2	TYR	16	13.577	4.598	-5.512	1.00	0.00	H
ATOM	268	HB3	TYR	16	13.354	5.809	-4.312	1.00	0.00	H
ATOM	269	CG	TYR	16	11.597	4.933	-5.184	1.00	0.00	C
ATOM	270	CD1	TYR	16	10.930	3.754	-5.668	1.00	0.00	C
ATOM	271	HD1	TYR	16	11.358	2.766	-5.766	1.00	0.00	H
ATOM	272	CE1	TYR	16	9.573	3.875	-6.061	1.00	0.00	C
ATOM	273	HE1	TYR	16	9.117	3.023	-6.543	1.00	0.00	H

```
ATOM    274  CZ  TYR    16       8.856   5.114  -5.924  1.00  0.00           C
ATOM    275  OH  TYR    16       7.573   5.184  -6.277  1.00  0.00           O
ATOM    276  HH  TYR    16       7.175   5.977  -5.909  1.00  0.00           H
ATOM    277  CE2 TYR    16       9.503   6.237  -5.265  1.00  0.00           C
ATOM    278  HE2 TYR    16       9.049   7.214  -5.201  1.00  0.00           H
ATOM    279  CD2 TYR    16      10.865   6.146  -4.946  1.00  0.00           C
ATOM    280  HD2 TYR    16      11.375   6.968  -4.467  1.00  0.00           H
ATOM    281  C   TYR    16      14.819   3.661  -3.172  1.00  0.00           C
ATOM    282  O   TYR    16      15.505   4.672  -3.023  1.00  0.00           O
ATOM    283  N   NME    17      15.314   2.415  -3.264  1.00  0.00           N
ATOM    284  H   NME    17      14.639   1.666  -3.329  1.00  0.00           H
ATOM    285  CH3 NME    17      16.757   2.016  -3.200  1.00  0.00           C
ATOM    286 HH31 NME    17      17.017   1.810  -4.238  1.00  0.00           H
ATOM    287 HH32 NME    17      17.312   2.909  -2.915  1.00  0.00           H
ATOM    288 HH33 NME    17      16.885   1.201  -2.488  1.00  0.00           H
TER     289      NME    17
END
```

Cluster 6, Figure 2B:

```
ATOM      1 HH31 ACE     1      25.519   5.760   7.795  1.00  0.00           H
ATOM      2  CH3 ACE     1      24.729   6.445   7.487  1.00  0.00           C
ATOM      3 HH32 ACE     1      25.208   7.350   7.112  1.00  0.00           H
ATOM      4 HH33 ACE     1      24.007   6.619   8.284  1.00  0.00           H
ATOM      5  C   ACE     1      23.920   5.663   6.447  1.00  0.00           C
ATOM      6  O   ACE     1      24.265   5.668   5.264  1.00  0.00           O
ATOM      7  N   ASN     2      22.846   5.011   6.922  1.00  0.00           N
ATOM      8  H   ASN     2      22.509   5.075   7.872  1.00  0.00           H
ATOM      9  CA  ASN     2      21.939   4.206   6.034  1.00  0.00           C
ATOM     10  HA  ASN     2      22.199   4.406   4.995  1.00  0.00           H
ATOM     11  CB  ASN     2      22.201   2.736   6.338  1.00  0.00           C
ATOM     12  HB2 ASN     2      21.644   2.413   7.218  1.00  0.00           H
ATOM     13  HB3 ASN     2      23.269   2.650   6.534  1.00  0.00           H
ATOM     14  CG  ASN     2      21.789   1.649   5.299  1.00  0.00           C
ATOM     15  OD1 ASN     2      21.517   2.002   4.123  1.00  0.00           O
ATOM     16  ND2 ASN     2      21.453   0.445   5.754  1.00  0.00           N
ATOM     17 HD21 ASN     2      20.941  -0.243   5.221  1.00  0.00           H
ATOM     18 HD22 ASN     2      21.557   0.274   6.744  1.00  0.00           H
ATOM     19  C   ASN     2      20.483   4.619   6.289  1.00  0.00           C
ATOM     20  O   ASN     2      20.190   5.050   7.428  1.00  0.00           O
ATOM     21  N   ASP     3      19.576   4.352   5.309  1.00  0.00           N
ATOM     22  H   ASP     3      19.928   4.024   4.422  1.00  0.00           H
ATOM     23  CA  ASP     3      18.121   4.837   5.262  1.00  0.00           C
ATOM     24  HA  ASP     3      18.094   5.691   5.939  1.00  0.00           H
ATOM     25  CB  ASP     3      17.870   5.302   3.808  1.00  0.00           C
ATOM     26  HB2 ASP     3      17.797   4.391   3.214  1.00  0.00           H
ATOM     27  HB3 ASP     3      18.745   5.907   3.569  1.00  0.00           H
ATOM     28  CG  ASP     3      16.596   6.246   3.720  1.00  0.00           C
ATOM     29  OD1 ASP     3      16.404   6.581   2.542  1.00  0.00           O
ATOM     30  OD2 ASP     3      16.035   6.742   4.697  1.00  0.00           O
ATOM     31  C   ASP     3      17.030   3.948   5.904  1.00  0.00           C
ATOM     32  O   ASP     3      16.130   3.498   5.206  1.00  0.00           O
ATOM     33  N   TYR     4      17.309   3.632   7.122  1.00  0.00           N
ATOM     34  H   TYR     4      18.214   3.905   7.478  1.00  0.00           H
ATOM     35  CA  TYR     4      16.429   2.872   7.956  1.00  0.00           C
ATOM     36  HA  TYR     4      16.043   1.987   7.449  1.00  0.00           H
ATOM     37  CB  TYR     4      17.249   2.404   9.114  1.00  0.00           C
ATOM     38  HB2 TYR     4      17.934   3.155   9.508  1.00  0.00           H
ATOM     39  HB3 TYR     4      17.952   1.701   8.668  1.00  0.00           H
ATOM     40  CG  TYR     4      16.449   1.713  10.215  1.00  0.00           C
ATOM     41  CD1 TYR     4      15.947   0.427  10.081  1.00  0.00           C
```

ATOM	42	HD1	TYR	4	16.061	-0.146	9.172	1.00	0.00	H
ATOM	43	CE1	TYR	4	15.394	-0.278	11.173	1.00	0.00	C
ATOM	44	HE1	TYR	4	15.098	-1.309	11.050	1.00	0.00	H
ATOM	45	CZ	TYR	4	15.296	0.395	12.387	1.00	0.00	C
ATOM	46	OH	TYR	4	14.749	-0.224	13.511	1.00	0.00	O
ATOM	47	HH	TYR	4	14.493	-1.125	13.299	1.00	0.00	H
ATOM	48	CE2	TYR	4	15.594	1.768	12.521	1.00	0.00	C
ATOM	49	HE2	TYR	4	15.250	2.257	13.420	1.00	0.00	H
ATOM	50	CD2	TYR	4	16.142	2.387	11.409	1.00	0.00	C
ATOM	51	HD2	TYR	4	16.514	3.388	11.571	1.00	0.00	H
ATOM	52	C	TYR	4	15.194	3.787	8.360	1.00	0.00	C
ATOM	53	O	TYR	4	15.222	5.009	8.392	1.00	0.00	O
ATOM	54	N	GLU	5	14.081	3.159	8.796	1.00	0.00	N
ATOM	55	H	GLU	5	14.105	2.152	8.866	1.00	0.00	H
ATOM	56	CA	GLU	5	12.960	3.968	9.374	1.00	0.00	C
ATOM	57	HA	GLU	5	13.276	4.995	9.555	1.00	0.00	H
ATOM	58	CB	GLU	5	11.759	4.029	8.378	1.00	0.00	C
ATOM	59	HB2	GLU	5	11.367	3.031	8.182	1.00	0.00	H
ATOM	60	HB3	GLU	5	12.131	4.381	7.416	1.00	0.00	H
ATOM	61	CG	GLU	5	10.512	4.783	8.953	1.00	0.00	C
ATOM	62	HG2	GLU	5	10.096	4.356	9.865	1.00	0.00	H
ATOM	63	HG3	GLU	5	9.774	4.755	8.151	1.00	0.00	H
ATOM	64	CD	GLU	5	10.797	6.290	9.229	1.00	0.00	C
ATOM	65	OE1	GLU	5	10.902	6.599	10.401	1.00	0.00	O
ATOM	66	OE2	GLU	5	10.980	7.078	8.281	1.00	0.00	O
ATOM	67	C	GLU	5	12.434	3.402	10.720	1.00	0.00	C
ATOM	68	O	GLU	5	11.858	2.351	10.722	1.00	0.00	O
ATOM	69	N	ASP	6	12.541	4.174	11.749	1.00	0.00	N
ATOM	70	H	ASP	6	12.942	5.090	11.608	1.00	0.00	H
ATOM	71	CA	ASP	6	12.308	3.781	13.160	1.00	0.00	C
ATOM	72	HA	ASP	6	12.908	2.899	13.384	1.00	0.00	H
ATOM	73	CB	ASP	6	12.772	5.041	14.039	1.00	0.00	C
ATOM	74	HB2	ASP	6	12.163	5.917	13.819	1.00	0.00	H
ATOM	75	HB3	ASP	6	13.812	5.130	13.723	1.00	0.00	H
ATOM	76	CG	ASP	6	12.659	4.751	15.510	1.00	0.00	C
ATOM	77	OD1	ASP	6	13.626	4.108	15.978	1.00	0.00	O
ATOM	78	OD2	ASP	6	11.619	5.118	16.117	1.00	0.00	O
ATOM	79	C	ASP	6	10.836	3.426	13.590	1.00	0.00	C
ATOM	80	O	ASP	6	10.595	2.578	14.427	1.00	0.00	O
ATOM	81	N	ARG	7	9.874	3.966	12.879	1.00	0.00	N
ATOM	82	H	ARG	7	10.158	4.688	12.232	1.00	0.00	H
ATOM	83	CA	ARG	7	8.426	3.743	12.849	1.00	0.00	C
ATOM	84	HA	ARG	7	8.056	3.478	13.839	1.00	0.00	H
ATOM	85	CB	ARG	7	7.751	5.034	12.366	1.00	0.00	C
ATOM	86	HB2	ARG	7	6.671	4.922	12.461	1.00	0.00	H
ATOM	87	HB3	ARG	7	8.050	5.107	11.320	1.00	0.00	H
ATOM	88	CG	ARG	7	8.098	6.388	13.040	1.00	0.00	C
ATOM	89	HG2	ARG	7	9.185	6.377	13.117	1.00	0.00	H
ATOM	90	HG3	ARG	7	7.675	6.320	14.042	1.00	0.00	H
ATOM	91	CD	ARG	7	7.721	7.675	12.304	1.00	0.00	C
ATOM	92	HD2	ARG	7	7.737	8.550	12.954	1.00	0.00	H
ATOM	93	HD3	ARG	7	6.645	7.610	12.141	1.00	0.00	H
ATOM	94	NE	ARG	7	8.514	7.911	11.047	1.00	0.00	N
ATOM	95	HE	ARG	7	9.404	7.443	10.954	1.00	0.00	H
ATOM	96	CZ	ARG	7	8.118	8.737	10.100	1.00	0.00	C
ATOM	97	NH1	ARG	7	7.065	9.488	10.272	1.00	0.00	N
ATOM	98	HH11	ARG	7	6.833	9.742	11.222	1.00	0.00	H
ATOM	99	HH12	ARG	7	6.728	10.007	9.474	1.00	0.00	H
ATOM	100	NH2	ARG	7	8.734	8.685	8.969	1.00	0.00	N
ATOM	101	HH21	ARG	7	8.254	9.040	8.154	1.00	0.00	H
ATOM	102	HH22	ARG	7	9.428	7.997	8.716	1.00	0.00	H
ATOM	103	C	ARG	7	8.041	2.588	11.939	1.00	0.00	C
ATOM	104	O	ARG	7	6.879	2.155	11.903	1.00	0.00	O

ATOM	105	N	TYR	8	8.927	2.156	11.014	1.00	0.00	N
ATOM	106	H	TYR	8	9.912	2.295	11.185	1.00	0.00	H
ATOM	107	CA	TYR	8	8.613	1.137	9.951	1.00	0.00	C
ATOM	108	HA	TYR	8	9.539	0.984	9.396	1.00	0.00	H
ATOM	109	CB	TYR	8	8.511	-0.251	10.495	1.00	0.00	C
ATOM	110	HB2	TYR	8	8.045	-0.878	9.736	1.00	0.00	H
ATOM	111	HB3	TYR	8	7.890	-0.243	11.390	1.00	0.00	H
ATOM	112	CG	TYR	8	9.876	-0.882	10.888	1.00	0.00	C
ATOM	113	CD1	TYR	8	10.196	-1.305	12.185	1.00	0.00	C
ATOM	114	HD1	TYR	8	9.561	-1.128	13.040	1.00	0.00	H
ATOM	115	CE1	TYR	8	11.354	-2.102	12.401	1.00	0.00	C
ATOM	116	HE1	TYR	8	11.614	-2.298	13.431	1.00	0.00	H
ATOM	117	CZ	TYR	8	12.220	-2.470	11.337	1.00	0.00	C
ATOM	118	OH	TYR	8	13.371	-3.076	11.559	1.00	0.00	O
ATOM	119	HH	TYR	8	13.654	-3.160	12.472	1.00	0.00	H
ATOM	120	CE2	TYR	8	11.962	-2.081	10.037	1.00	0.00	C
ATOM	121	HE2	TYR	8	12.657	-2.279	9.234	1.00	0.00	H
ATOM	122	CD2	TYR	8	10.721	-1.319	9.798	1.00	0.00	C
ATOM	123	HD2	TYR	8	10.456	-1.051	8.786	1.00	0.00	H
ATOM	124	C	TYR	8	7.523	1.403	8.915	1.00	0.00	C
ATOM	125	O	TYR	8	7.529	0.704	7.912	1.00	0.00	O
ATOM	126	N	TYR	9	6.664	2.402	9.112	1.00	0.00	N
ATOM	127	H	TYR	9	6.782	2.897	9.984	1.00	0.00	H
ATOM	128	CA	TYR	9	5.733	3.037	8.149	1.00	0.00	C
ATOM	129	HA	TYR	9	5.709	2.353	7.301	1.00	0.00	H
ATOM	130	CB	TYR	9	4.385	2.964	8.720	1.00	0.00	C
ATOM	131	HB2	TYR	9	4.397	3.448	9.696	1.00	0.00	H
ATOM	132	HB3	TYR	9	4.181	1.902	8.858	1.00	0.00	H
ATOM	133	CG	TYR	9	3.233	3.600	7.996	1.00	0.00	C
ATOM	134	CD1	TYR	9	2.607	4.639	8.650	1.00	0.00	C
ATOM	135	HD1	TYR	9	2.893	5.008	9.624	1.00	0.00	H
ATOM	136	CE1	TYR	9	1.533	5.322	8.059	1.00	0.00	C
ATOM	137	HE1	TYR	9	1.102	6.137	8.622	1.00	0.00	H
ATOM	138	CZ	TYR	9	1.042	5.006	6.802	1.00	0.00	C
ATOM	139	OH	TYR	9	0.191	5.841	6.047	1.00	0.00	O
ATOM	140	HH	TYR	9	0.022	5.529	5.155	1.00	0.00	H
ATOM	141	CE2	TYR	9	1.789	3.994	6.103	1.00	0.00	C
ATOM	142	HE2	TYR	9	1.586	3.863	5.051	1.00	0.00	H
ATOM	143	CD2	TYR	9	2.793	3.240	6.716	1.00	0.00	C
ATOM	144	HD2	TYR	9	3.328	2.527	6.107	1.00	0.00	H
ATOM	145	C	TYR	9	6.355	4.415	7.923	1.00	0.00	C
ATOM	146	O	TYR	9	6.473	5.206	8.887	1.00	0.00	O
ATOM	147	N	ARG	10	6.743	4.720	6.662	1.00	0.00	N
ATOM	148	H	ARG	10	6.467	4.070	5.940	1.00	0.00	H
ATOM	149	CA	ARG	10	7.175	6.037	6.381	1.00	0.00	C
ATOM	150	HA	ARG	10	7.236	6.652	7.279	1.00	0.00	H
ATOM	151	CB	ARG	10	8.473	6.038	5.464	1.00	0.00	C
ATOM	152	HB2	ARG	10	8.167	5.781	4.449	1.00	0.00	H
ATOM	153	HB3	ARG	10	9.134	5.388	6.037	1.00	0.00	H
ATOM	154	CG	ARG	10	9.063	7.456	5.468	1.00	0.00	C
ATOM	155	HG2	ARG	10	9.227	7.793	6.492	1.00	0.00	H
ATOM	156	HG3	ARG	10	8.289	8.049	4.981	1.00	0.00	H
ATOM	157	CD	ARG	10	10.368	7.552	4.623	1.00	0.00	C
ATOM	158	HD2	ARG	10	10.565	8.556	4.248	1.00	0.00	H
ATOM	159	HD3	ARG	10	10.266	6.849	3.797	1.00	0.00	H
ATOM	160	NE	ARG	10	11.563	7.137	5.481	1.00	0.00	N
ATOM	161	HE	ARG	10	11.388	7.171	6.476	1.00	0.00	H
ATOM	162	CZ	ARG	10	12.838	6.870	5.197	1.00	0.00	C
ATOM	163	NH1	ARG	10	13.343	6.777	3.973	1.00	0.00	N
ATOM	164	HH11	ARG	10	12.699	6.615	3.211	1.00	0.00	H
ATOM	165	HH12	ARG	10	14.341	6.688	3.845	1.00	0.00	H
ATOM	166	NH2	ARG	10	13.726	6.809	6.081	1.00	0.00	N
ATOM	167	HH21	ARG	10	13.552	6.853	7.075	1.00	0.00	H

ATOM	168	HH22	ARG	10	14.704	6.791	5.829	1.00	0.00	H
ATOM	169	C	ARG	10	6.007	6.677	5.553	1.00	0.00	C
ATOM	170	O	ARG	10	5.530	5.929	4.679	1.00	0.00	O
ATOM	171	N	GLU	11	5.531	7.878	5.835	1.00	0.00	N
ATOM	172	H	GLU	11	5.952	8.413	6.581	1.00	0.00	H
ATOM	173	CA	GLU	11	4.418	8.501	5.188	1.00	0.00	C
ATOM	174	HA	GLU	11	3.548	7.854	5.301	1.00	0.00	H
ATOM	175	CB	GLU	11	3.937	9.792	5.902	1.00	0.00	C
ATOM	176	HB2	GLU	11	3.533	9.621	6.900	1.00	0.00	H
ATOM	177	HB3	GLU	11	3.128	10.076	5.229	1.00	0.00	H
ATOM	178	CG	GLU	11	4.902	11.062	6.207	1.00	0.00	C
ATOM	179	HG2	GLU	11	4.433	12.029	6.388	1.00	0.00	H
ATOM	180	HG3	GLU	11	5.493	11.196	5.301	1.00	0.00	H
ATOM	181	CD	GLU	11	5.915	10.741	7.384	1.00	0.00	C
ATOM	182	OE1	GLU	11	5.717	11.251	8.493	1.00	0.00	O
ATOM	183	OE2	GLU	11	6.828	9.930	7.132	1.00	0.00	O
ATOM	184	C	GLU	11	4.557	8.696	3.688	1.00	0.00	C
ATOM	185	O	GLU	11	3.580	8.456	2.969	1.00	0.00	O
ATOM	186	N	ASN	12	5.794	8.922	3.207	1.00	0.00	N
ATOM	187	H	ASN	12	6.578	8.927	3.844	1.00	0.00	H
ATOM	188	CA	ASN	12	6.088	9.023	1.833	1.00	0.00	C
ATOM	189	HA	ASN	12	5.170	9.301	1.316	1.00	0.00	H
ATOM	190	CB	ASN	12	7.186	10.167	1.613	1.00	0.00	C
ATOM	191	HB2	ASN	12	8.176	9.720	1.695	1.00	0.00	H
ATOM	192	HB3	ASN	12	7.323	10.795	2.494	1.00	0.00	H
ATOM	193	CG	ASN	12	7.007	10.995	0.323	1.00	0.00	C
ATOM	194	OD1	ASN	12	6.274	10.703	-0.620	1.00	0.00	O
ATOM	195	ND2	ASN	12	7.564	12.173	0.286	1.00	0.00	N
ATOM	196	HD21	ASN	12	7.351	12.738	-0.524	1.00	0.00	H
ATOM	197	HD22	ASN	12	8.179	12.457	1.035	1.00	0.00	H
ATOM	198	C	ASN	12	6.572	7.676	1.209	1.00	0.00	C
ATOM	199	O	ASN	12	7.749	7.301	1.032	1.00	0.00	O
ATOM	200	N	MET	13	5.590	6.997	0.636	1.00	0.00	N
ATOM	201	H	MET	13	4.644	7.348	0.690	1.00	0.00	H
ATOM	202	CA	MET	13	5.736	5.730	-0.061	1.00	0.00	C
ATOM	203	HA	MET	13	6.162	5.025	0.653	1.00	0.00	H
ATOM	204	CB	MET	13	4.238	5.321	-0.478	1.00	0.00	C
ATOM	205	HB2	MET	13	4.261	4.403	-1.066	1.00	0.00	H
ATOM	206	HB3	MET	13	4.037	6.196	-1.096	1.00	0.00	H
ATOM	207	CG	MET	13	3.082	5.195	0.546	1.00	0.00	C
ATOM	208	HG2	MET	13	2.112	5.340	0.070	1.00	0.00	H
ATOM	209	HG3	MET	13	3.293	5.935	1.317	1.00	0.00	H
ATOM	210	SD	MET	13	2.776	3.606	1.318	1.00	0.00	S
ATOM	211	CE	MET	13	3.686	3.937	2.813	1.00	0.00	C
ATOM	212	HE1	MET	13	4.717	4.017	2.468	1.00	0.00	H
ATOM	213	HE2	MET	13	3.382	4.889	3.248	1.00	0.00	H
ATOM	214	HE3	MET	13	3.653	3.146	3.563	1.00	0.00	H
ATOM	215	C	MET	13	6.646	5.767	-1.349	1.00	0.00	C
ATOM	216	O	MET	13	7.211	4.777	-1.698	1.00	0.00	O
ATOM	217	N	TYR	14	6.849	6.989	-1.892	1.00	0.00	N
ATOM	218	H	TYR	14	6.332	7.776	-1.525	1.00	0.00	H
ATOM	219	CA	TYR	14	7.864	7.193	-2.937	1.00	0.00	C
ATOM	220	HA	TYR	14	7.768	6.380	-3.656	1.00	0.00	H
ATOM	221	CB	TYR	14	7.577	8.423	-3.767	1.00	0.00	C
ATOM	222	HB2	TYR	14	8.173	8.398	-4.679	1.00	0.00	H
ATOM	223	HB3	TYR	14	7.789	9.318	-3.183	1.00	0.00	H
ATOM	224	CG	TYR	14	6.110	8.479	-4.227	1.00	0.00	C
ATOM	225	CD1	TYR	14	5.319	9.550	-3.786	1.00	0.00	C
ATOM	226	HD1	TYR	14	5.635	10.260	-3.036	1.00	0.00	H
ATOM	227	CE1	TYR	14	4.092	9.776	-4.429	1.00	0.00	C
ATOM	228	HE1	TYR	14	3.444	10.568	-4.085	1.00	0.00	H
ATOM	229	CZ	TYR	14	3.651	8.954	-5.508	1.00	0.00	C
ATOM	230	OH	TYR	14	2.426	9.193	-6.071	1.00	0.00	O

```
ATOM    231  HH  TYR    14       2.071   9.997  -5.685  1.00  0.00           H
ATOM    232  CE2 TYR    14       4.327   7.807  -5.840  1.00  0.00           C
ATOM    233  HE2 TYR    14       3.976   7.245  -6.692  1.00  0.00           H
ATOM    234  CD2 TYR    14       5.598   7.601  -5.206  1.00  0.00           C
ATOM    235  HD2 TYR    14       6.275   6.866  -5.614  1.00  0.00           H
ATOM    236  C   TYR    14       9.318   7.168  -2.417  1.00  0.00           C
ATOM    237  O   TYR    14      10.174   7.012  -3.259  1.00  0.00           O
ATOM    238  N   ARG    15       9.571   7.189  -1.121  1.00  0.00           N
ATOM    239  H   ARG    15       8.824   7.493  -0.513  1.00  0.00           H
ATOM    240  CA  ARG    15      10.855   7.254  -0.486  1.00  0.00           C
ATOM    241  HA  ARG    15      11.591   6.836  -1.172  1.00  0.00           H
ATOM    242  CB  ARG    15      11.274   8.678  -0.094  1.00  0.00           C
ATOM    243  HB2 ARG    15      11.952   8.634   0.758  1.00  0.00           H
ATOM    244  HB3 ARG    15      10.330   9.154   0.172  1.00  0.00           H
ATOM    245  CG  ARG    15      11.809   9.573  -1.220  1.00  0.00           C
ATOM    246  HG2 ARG    15      11.734  10.632  -0.974  1.00  0.00           H
ATOM    247  HG3 ARG    15      11.074   9.428  -2.012  1.00  0.00           H
ATOM    248  CD  ARG    15      13.255   9.252  -1.682  1.00  0.00           C
ATOM    249  HD2 ARG    15      13.572  10.014  -2.394  1.00  0.00           H
ATOM    250  HD3 ARG    15      13.197   8.258  -2.124  1.00  0.00           H
ATOM    251  NE  ARG    15      14.236   9.365  -0.576  1.00  0.00           N
ATOM    252  HE  ARG    15      14.242  10.300  -0.194  1.00  0.00           H
ATOM    253  CZ  ARG    15      15.051   8.458  -0.015  1.00  0.00           C
ATOM    254  NH1 ARG    15      15.227   7.353  -0.605  1.00  0.00           N
ATOM    255 HH11 ARG    15      14.811   7.295  -1.524  1.00  0.00           H
ATOM    256 HH12 ARG    15      15.714   6.600  -0.140  1.00  0.00           H
ATOM    257  NH2 ARG    15      15.531   8.645   1.193  1.00  0.00           N
ATOM    258 HH21 ARG    15      15.792   9.554   1.548  1.00  0.00           H
ATOM    259 HH22 ARG    15      15.988   7.843   1.603  1.00  0.00           H
ATOM    260  C   ARG    15      11.062   6.181   0.636  1.00  0.00           C
ATOM    261  O   ARG    15      12.016   6.227   1.375  1.00  0.00           O
ATOM    262  N   TYR    16      10.136   5.234   0.691  1.00  0.00           N
ATOM    263  H   TYR    16       9.475   5.220  -0.073  1.00  0.00           H
ATOM    264  CA  TYR    16      10.105   4.115   1.573  1.00  0.00           C
ATOM    265  HA  TYR    16      10.512   4.292   2.569  1.00  0.00           H
ATOM    266  CB  TYR    16       8.597   3.779   1.748  1.00  0.00           C
ATOM    267  HB2 TYR    16       8.049   3.553   0.833  1.00  0.00           H
ATOM    268  HB3 TYR    16       8.180   4.683   2.192  1.00  0.00           H
ATOM    269  CG  TYR    16       8.195   2.637   2.730  1.00  0.00           C
ATOM    270  CD1 TYR    16       8.577   2.676   4.136  1.00  0.00           C
ATOM    271  HD1 TYR    16       9.309   3.352   4.553  1.00  0.00           H
ATOM    272  CE1 TYR    16       8.173   1.725   5.033  1.00  0.00           C
ATOM    273  HE1 TYR    16       8.570   1.754   6.037  1.00  0.00           H
ATOM    274  CZ  TYR    16       7.517   0.562   4.544  1.00  0.00           C
ATOM    275  OH  TYR    16       7.232  -0.458   5.440  1.00  0.00           O
ATOM    276  HH  TYR    16       7.517  -0.149   6.304  1.00  0.00           H
ATOM    277  CE2 TYR    16       7.271   0.423   3.167  1.00  0.00           C
ATOM    278  HE2 TYR    16       6.851  -0.481   2.751  1.00  0.00           H
ATOM    279  CD2 TYR    16       7.646   1.475   2.271  1.00  0.00           C
ATOM    280  HD2 TYR    16       7.458   1.413   1.209  1.00  0.00           H
ATOM    281  C   TYR    16      10.866   2.840   1.134  1.00  0.00           C
ATOM    282  O   TYR    16      11.272   2.670  -0.071  1.00  0.00           O
ATOM    283  N   NME    17      11.181   1.954   2.099  1.00  0.00           N
ATOM    284  H   NME    17      10.837   1.965   3.049  1.00  0.00           H
ATOM    285  CH3 NME    17      11.706   0.647   1.815  1.00  0.00           C
ATOM    286 HH31 NME    17      12.205   0.564   0.849  1.00  0.00           H
ATOM    287 HH32 NME    17      12.505   0.406   2.516  1.00  0.00           H
ATOM    288 HH33 NME    17      10.860  -0.022   1.970  1.00  0.00           H
TER     289      NME    17
END
```

Cluster 7:

ATOM	1	HH31	ACE	1	21.917	-2.565	12.479	1.00	0.00	H
ATOM	2	CH3	ACE	1	20.839	-2.475	12.618	1.00	0.00	C
ATOM	3	HH32	ACE	1	20.525	-1.913	13.497	1.00	0.00	H
ATOM	4	HH33	ACE	1	20.508	-3.500	12.788	1.00	0.00	H
ATOM	5	C	ACE	1	20.093	-1.872	11.458	1.00	0.00	C
ATOM	6	O	ACE	1	20.670	-1.755	10.386	1.00	0.00	O
ATOM	7	N	ASN	2	18.914	-1.422	11.742	1.00	0.00	N
ATOM	8	H	ASN	2	18.557	-1.613	12.668	1.00	0.00	H
ATOM	9	CA	ASN	2	17.861	-0.937	10.788	1.00	0.00	C
ATOM	10	HA	ASN	2	17.902	-1.616	9.937	1.00	0.00	H
ATOM	11	CB	ASN	2	16.485	-1.233	11.510	1.00	0.00	C
ATOM	12	HB2	ASN	2	16.419	-0.620	12.408	1.00	0.00	H
ATOM	13	HB3	ASN	2	16.577	-2.298	11.724	1.00	0.00	H
ATOM	14	CG	ASN	2	15.220	-1.009	10.736	1.00	0.00	C
ATOM	15	OD1	ASN	2	15.210	-0.451	9.634	1.00	0.00	O
ATOM	16	ND2	ASN	2	14.150	-1.529	11.300	1.00	0.00	N
ATOM	17	HD21	ASN	2	13.208	-1.472	10.938	1.00	0.00	H
ATOM	18	HD22	ASN	2	14.302	-1.933	12.214	1.00	0.00	H
ATOM	19	C	ASN	2	18.147	0.487	10.297	1.00	0.00	C
ATOM	20	O	ASN	2	18.492	1.384	11.072	1.00	0.00	O
ATOM	21	N	ASP	3	17.988	0.737	8.986	1.00	0.00	N
ATOM	22	H	ASP	3	17.801	-0.074	8.414	1.00	0.00	H
ATOM	23	CA	ASP	3	18.230	2.049	8.361	1.00	0.00	C
ATOM	24	HA	ASP	3	18.895	2.732	8.889	1.00	0.00	H
ATOM	25	CB	ASP	3	18.995	1.812	7.045	1.00	0.00	C
ATOM	26	HB2	ASP	3	18.390	1.160	6.415	1.00	0.00	H
ATOM	27	HB3	ASP	3	19.936	1.359	7.357	1.00	0.00	H
ATOM	28	CG	ASP	3	19.314	3.113	6.250	1.00	0.00	C
ATOM	29	OD1	ASP	3	20.328	3.764	6.564	1.00	0.00	O
ATOM	30	OD2	ASP	3	18.618	3.324	5.240	1.00	0.00	O
ATOM	31	C	ASP	3	16.881	2.674	8.165	1.00	0.00	C
ATOM	32	O	ASP	3	16.733	3.908	8.334	1.00	0.00	O
ATOM	33	N	TYR	4	15.774	1.922	7.860	1.00	0.00	N
ATOM	34	H	TYR	4	15.929	0.935	7.709	1.00	0.00	H
ATOM	35	CA	TYR	4	14.486	2.509	7.637	1.00	0.00	C
ATOM	36	HA	TYR	4	14.750	3.343	6.986	1.00	0.00	H
ATOM	37	CB	TYR	4	13.595	1.599	6.687	1.00	0.00	C
ATOM	38	HB2	TYR	4	13.323	0.699	7.238	1.00	0.00	H
ATOM	39	HB3	TYR	4	14.227	1.395	5.822	1.00	0.00	H
ATOM	40	CG	TYR	4	12.214	2.047	6.164	1.00	0.00	C
ATOM	41	CD1	TYR	4	12.069	3.385	5.699	1.00	0.00	C
ATOM	42	HD1	TYR	4	12.936	3.966	5.424	1.00	0.00	H
ATOM	43	CE1	TYR	4	10.850	3.973	5.329	1.00	0.00	C
ATOM	44	HE1	TYR	4	10.701	4.954	4.902	1.00	0.00	H
ATOM	45	CZ	TYR	4	9.712	3.161	5.532	1.00	0.00	C
ATOM	46	OH	TYR	4	8.456	3.655	5.266	1.00	0.00	O
ATOM	47	HH	TYR	4	8.510	4.573	4.989	1.00	0.00	H
ATOM	48	CE2	TYR	4	9.760	1.866	6.048	1.00	0.00	C
ATOM	49	HE2	TYR	4	8.839	1.314	6.167	1.00	0.00	H
ATOM	50	CD2	TYR	4	11.022	1.282	6.320	1.00	0.00	C
ATOM	51	HD2	TYR	4	11.173	0.240	6.560	1.00	0.00	H
ATOM	52	C	TYR	4	13.763	2.969	8.991	1.00	0.00	C
ATOM	53	O	TYR	4	12.749	3.611	8.947	1.00	0.00	O
ATOM	54	N	GLU	5	14.325	2.650	10.208	1.00	0.00	N
ATOM	55	H	GLU	5	15.108	2.012	10.200	1.00	0.00	H
ATOM	56	CA	GLU	5	13.644	2.826	11.470	1.00	0.00	C
ATOM	57	HA	GLU	5	12.772	2.174	11.416	1.00	0.00	H
ATOM	58	CB	GLU	5	14.589	2.350	12.514	1.00	0.00	C
ATOM	59	HB2	GLU	5	15.322	3.050	12.916	1.00	0.00	H
ATOM	60	HB3	GLU	5	15.145	1.547	12.031	1.00	0.00	H
ATOM	61	CG	GLU	5	13.801	1.838	13.723	1.00	0.00	C

ATOM	62	HG2	GLU	5	13.027	1.099	13.517	1.00	0.00	H
ATOM	63	HG3	GLU	5	13.431	2.800	14.078	1.00	0.00	H
ATOM	64	CD	GLU	5	14.736	1.151	14.771	1.00	0.00	C
ATOM	65	OE1	GLU	5	14.566	-0.090	14.977	1.00	0.00	O
ATOM	66	OE2	GLU	5	15.554	1.867	15.338	1.00	0.00	O
ATOM	67	C	GLU	5	13.212	4.304	11.735	1.00	0.00	C
ATOM	68	O	GLU	5	12.060	4.597	12.042	1.00	0.00	O
ATOM	69	N	ASP	6	14.173	5.246	11.458	1.00	0.00	N
ATOM	70	H	ASP	6	15.095	4.912	11.214	1.00	0.00	H
ATOM	71	CA	ASP	6	13.962	6.678	11.404	1.00	0.00	C
ATOM	72	HA	ASP	6	13.388	6.917	12.299	1.00	0.00	H
ATOM	73	CB	ASP	6	15.314	7.423	11.306	1.00	0.00	C
ATOM	74	HB2	ASP	6	15.910	7.024	10.485	1.00	0.00	H
ATOM	75	HB3	ASP	6	15.806	7.213	12.256	1.00	0.00	H
ATOM	76	CG	ASP	6	15.271	8.891	11.038	1.00	0.00	C
ATOM	77	OD1	ASP	6	14.868	9.578	12.003	1.00	0.00	O
ATOM	78	OD2	ASP	6	15.602	9.404	9.940	1.00	0.00	O
ATOM	79	C	ASP	6	13.125	7.204	10.204	1.00	0.00	C
ATOM	80	O	ASP	6	12.540	8.318	10.311	1.00	0.00	O
ATOM	81	N	ARG	7	13.340	6.515	9.060	1.00	0.00	N
ATOM	82	H	ARG	7	13.919	5.690	9.122	1.00	0.00	H
ATOM	83	CA	ARG	7	12.784	7.038	7.795	1.00	0.00	C
ATOM	84	HA	ARG	7	12.835	8.127	7.833	1.00	0.00	H
ATOM	85	CB	ARG	7	13.733	6.636	6.653	1.00	0.00	C
ATOM	86	HB2	ARG	7	13.798	5.560	6.491	1.00	0.00	H
ATOM	87	HB3	ARG	7	14.681	7.031	7.021	1.00	0.00	H
ATOM	88	CG	ARG	7	13.340	7.389	5.386	1.00	0.00	C
ATOM	89	HG2	ARG	7	13.142	8.445	5.572	1.00	0.00	H
ATOM	90	HG3	ARG	7	12.430	6.954	4.973	1.00	0.00	H
ATOM	91	CD	ARG	7	14.429	7.426	4.340	1.00	0.00	C
ATOM	92	HD2	ARG	7	15.282	8.069	4.557	1.00	0.00	H
ATOM	93	HD3	ARG	7	14.054	7.883	3.424	1.00	0.00	H
ATOM	94	NE	ARG	7	14.940	6.107	3.907	1.00	0.00	N
ATOM	95	HE	ARG	7	14.339	5.562	3.306	1.00	0.00	H
ATOM	96	CZ	ARG	7	16.043	5.526	4.260	1.00	0.00	C
ATOM	97	NH1	ARG	7	16.960	6.000	5.064	1.00	0.00	N
ATOM	98	HH11	ARG	7	16.701	6.820	5.594	1.00	0.00	H
ATOM	99	HH12	ARG	7	17.825	5.528	5.285	1.00	0.00	H
ATOM	100	NH2	ARG	7	16.344	4.328	3.894	1.00	0.00	N
ATOM	101	HH21	ARG	7	17.219	3.964	4.242	1.00	0.00	H
ATOM	102	HH22	ARG	7	15.653	3.831	3.351	1.00	0.00	H
ATOM	103	C	ARG	7	11.292	6.795	7.588	1.00	0.00	C
ATOM	104	O	ARG	7	10.641	7.539	6.841	1.00	0.00	O
ATOM	105	N	TYR	8	10.765	5.838	8.396	1.00	0.00	N
ATOM	106	H	TYR	8	11.429	5.272	8.904	1.00	0.00	H
ATOM	107	CA	TYR	8	9.334	5.431	8.400	1.00	0.00	C
ATOM	108	HA	TYR	8	9.071	5.011	7.429	1.00	0.00	H
ATOM	109	CB	TYR	8	9.113	4.289	9.449	1.00	0.00	C
ATOM	110	HB2	TYR	8	9.801	4.516	10.263	1.00	0.00	H
ATOM	111	HB3	TYR	8	9.476	3.386	8.959	1.00	0.00	H
ATOM	112	CG	TYR	8	7.746	4.051	10.055	1.00	0.00	C
ATOM	113	CD1	TYR	8	6.673	3.628	9.280	1.00	0.00	C
ATOM	114	HD1	TYR	8	6.882	3.375	8.252	1.00	0.00	H
ATOM	115	CE1	TYR	8	5.344	3.529	9.784	1.00	0.00	C
ATOM	116	HE1	TYR	8	4.513	3.161	9.201	1.00	0.00	H
ATOM	117	CZ	TYR	8	5.263	3.638	11.193	1.00	0.00	C
ATOM	118	OH	TYR	8	4.048	3.285	11.730	1.00	0.00	O
ATOM	119	HH	TYR	8	3.974	3.573	12.643	1.00	0.00	H
ATOM	120	CE2	TYR	8	6.359	4.084	12.033	1.00	0.00	C
ATOM	121	HE2	TYR	8	6.147	4.118	13.091	1.00	0.00	H
ATOM	122	CD2	TYR	8	7.597	4.372	11.395	1.00	0.00	C
ATOM	123	HD2	TYR	8	8.446	4.770	11.932	1.00	0.00	H
ATOM	124	C	TYR	8	8.435	6.593	8.710	1.00	0.00	C

ATOM	125	O	TYR	8	7.435	6.703	7.998	1.00	0.00	O
ATOM	126	N	TYR	9	8.756	7.479	9.649	1.00	0.00	N
ATOM	127	H	TYR	9	9.637	7.317	10.116	1.00	0.00	H
ATOM	128	CA	TYR	9	7.987	8.663	9.988	1.00	0.00	C
ATOM	129	HA	TYR	9	6.966	8.291	10.076	1.00	0.00	H
ATOM	130	CB	TYR	9	8.267	9.151	11.364	1.00	0.00	C
ATOM	131	HB2	TYR	9	7.641	10.000	11.636	1.00	0.00	H
ATOM	132	HB3	TYR	9	9.315	9.437	11.275	1.00	0.00	H
ATOM	133	CG	TYR	9	8.048	8.094	12.462	1.00	0.00	C
ATOM	134	CD1	TYR	9	9.086	7.866	13.382	1.00	0.00	C
ATOM	135	HD1	TYR	9	9.986	8.456	13.294	1.00	0.00	H
ATOM	136	CE1	TYR	9	8.828	7.136	14.559	1.00	0.00	C
ATOM	137	HE1	TYR	9	9.621	7.177	15.292	1.00	0.00	H
ATOM	138	CZ	TYR	9	7.573	6.477	14.712	1.00	0.00	C
ATOM	139	OH	TYR	9	7.269	5.786	15.843	1.00	0.00	O
ATOM	140	HH	TYR	9	8.083	5.660	16.336	1.00	0.00	H
ATOM	141	CE2	TYR	9	6.522	6.668	13.794	1.00	0.00	C
ATOM	142	HE2	TYR	9	5.540	6.234	13.908	1.00	0.00	H
ATOM	143	CD2	TYR	9	6.761	7.610	12.727	1.00	0.00	C
ATOM	144	HD2	TYR	9	5.968	7.765	12.010	1.00	0.00	H
ATOM	145	C	TYR	9	7.961	9.854	8.982	1.00	0.00	C
ATOM	146	O	TYR	9	7.402	10.930	9.294	1.00	0.00	O
ATOM	147	N	ARG	10	8.594	9.676	7.792	1.00	0.00	N
ATOM	148	H	ARG	10	9.080	8.799	7.671	1.00	0.00	H
ATOM	149	CA	ARG	10	8.996	10.794	6.894	1.00	0.00	C
ATOM	150	HA	ARG	10	8.524	11.745	7.139	1.00	0.00	H
ATOM	151	CB	ARG	10	10.535	11.001	7.006	1.00	0.00	C
ATOM	152	HB2	ARG	10	11.036	11.596	6.243	1.00	0.00	H
ATOM	153	HB3	ARG	10	10.876	9.970	6.909	1.00	0.00	H
ATOM	154	CG	ARG	10	10.986	11.514	8.445	1.00	0.00	C
ATOM	155	HG2	ARG	10	10.644	10.822	9.215	1.00	0.00	H
ATOM	156	HG3	ARG	10	10.580	12.523	8.518	1.00	0.00	H
ATOM	157	CD	ARG	10	12.525	11.529	8.623	1.00	0.00	C
ATOM	158	HD2	ARG	10	13.063	12.379	8.204	1.00	0.00	H
ATOM	159	HD3	ARG	10	12.906	10.639	8.122	1.00	0.00	H
ATOM	160	NE	ARG	10	12.907	11.390	10.107	1.00	0.00	N
ATOM	161	HE	ARG	10	13.447	10.583	10.387	1.00	0.00	H
ATOM	162	CZ	ARG	10	12.605	12.199	11.135	1.00	0.00	C
ATOM	163	NH1	ARG	10	13.069	11.886	12.291	1.00	0.00	N
ATOM	164	HH11	ARG	10	13.688	11.096	12.405	1.00	0.00	H
ATOM	165	HH12	ARG	10	12.900	12.499	13.076	1.00	0.00	H
ATOM	166	NH2	ARG	10	11.871	13.286	10.964	1.00	0.00	N
ATOM	167	HH21	ARG	10	11.393	13.384	10.079	1.00	0.00	H
ATOM	168	HH22	ARG	10	11.859	14.071	11.599	1.00	0.00	H
ATOM	169	C	ARG	10	8.639	10.474	5.498	1.00	0.00	C
ATOM	170	O	ARG	10	8.288	11.341	4.663	1.00	0.00	O
ATOM	171	N	GLU	11	8.734	9.147	5.157	1.00	0.00	N
ATOM	172	H	GLU	11	9.091	8.399	5.735	1.00	0.00	H
ATOM	173	CA	GLU	11	8.246	8.664	3.842	1.00	0.00	C
ATOM	174	HA	GLU	11	7.465	9.332	3.479	1.00	0.00	H
ATOM	175	CB	GLU	11	9.326	8.851	2.788	1.00	0.00	C
ATOM	176	HB2	GLU	11	10.122	8.257	3.237	1.00	0.00	H
ATOM	177	HB3	GLU	11	9.519	9.922	2.723	1.00	0.00	H
ATOM	178	CG	GLU	11	9.089	8.099	1.429	1.00	0.00	C
ATOM	179	HG2	GLU	11	8.046	8.141	1.117	1.00	0.00	H
ATOM	180	HG3	GLU	11	9.314	7.057	1.658	1.00	0.00	H
ATOM	181	CD	GLU	11	10.011	8.714	0.362	1.00	0.00	C
ATOM	182	OE1	GLU	11	9.561	9.683	-0.284	1.00	0.00	O
ATOM	183	OE2	GLU	11	11.181	8.242	0.236	1.00	0.00	O
ATOM	184	C	GLU	11	7.760	7.226	3.813	1.00	0.00	C
ATOM	185	O	GLU	11	8.435	6.335	4.261	1.00	0.00	O
ATOM	186	N	ASN	12	6.528	6.979	3.455	1.00	0.00	N
ATOM	187	H	ASN	12	5.819	7.648	3.189	1.00	0.00	H

ATOM	188	CA	ASN	12	5.961	5.609	3.521	1.00	0.00	C
ATOM	189	HA	ASN	12	6.273	5.111	4.439	1.00	0.00	H
ATOM	190	CB	ASN	12	4.491	5.693	3.758	1.00	0.00	C
ATOM	191	HB2	ASN	12	3.944	6.174	2.948	1.00	0.00	H
ATOM	192	HB3	ASN	12	4.391	6.373	4.605	1.00	0.00	H
ATOM	193	CG	ASN	12	3.714	4.437	4.013	1.00	0.00	C
ATOM	194	OD1	ASN	12	3.663	3.606	3.092	1.00	0.00	O
ATOM	195	ND2	ASN	12	3.073	4.239	5.183	1.00	0.00	N
ATOM	196	HD21	ASN	12	2.818	3.263	5.242	1.00	0.00	H
ATOM	197	HD22	ASN	12	2.957	5.002	5.834	1.00	0.00	H
ATOM	198	C	ASN	12	6.377	4.728	2.308	1.00	0.00	C
ATOM	199	O	ASN	12	6.725	5.289	1.284	1.00	0.00	O
ATOM	200	N	MET	13	6.516	3.492	2.486	1.00	0.00	N
ATOM	201	H	MET	13	6.221	3.105	3.371	1.00	0.00	H
ATOM	202	CA	MET	13	6.943	2.568	1.452	1.00	0.00	C
ATOM	203	HA	MET	13	7.948	2.912	1.207	1.00	0.00	H
ATOM	204	CB	MET	13	7.094	1.181	2.018	1.00	0.00	C
ATOM	205	HB2	MET	13	7.704	1.132	2.920	1.00	0.00	H
ATOM	206	HB3	MET	13	7.655	0.649	1.249	1.00	0.00	H
ATOM	207	CG	MET	13	5.744	0.512	2.400	1.00	0.00	C
ATOM	208	HG2	MET	13	5.118	0.261	1.543	1.00	0.00	H
ATOM	209	HG3	MET	13	5.201	1.117	3.127	1.00	0.00	H
ATOM	210	SD	MET	13	5.836	-1.151	3.162	1.00	0.00	S
ATOM	211	CE	MET	13	4.159	-1.036	4.012	1.00	0.00	C
ATOM	212	HE1	MET	13	4.333	-0.751	5.049	1.00	0.00	H
ATOM	213	HE2	MET	13	3.470	-0.430	3.424	1.00	0.00	H
ATOM	214	HE3	MET	13	3.757	-2.050	4.012	1.00	0.00	H
ATOM	215	C	MET	13	6.148	2.554	0.156	1.00	0.00	C
ATOM	216	O	MET	13	6.778	2.379	-0.917	1.00	0.00	O
ATOM	217	N	TYR	14	4.860	2.889	0.144	1.00	0.00	N
ATOM	218	H	TYR	14	4.414	3.122	1.020	1.00	0.00	H
ATOM	219	CA	TYR	14	4.100	2.836	-1.135	1.00	0.00	C
ATOM	220	HA	TYR	14	4.443	1.994	-1.736	1.00	0.00	H
ATOM	221	CB	TYR	14	2.644	2.574	-0.835	1.00	0.00	C
ATOM	222	HB2	TYR	14	1.978	2.613	-1.697	1.00	0.00	H
ATOM	223	HB3	TYR	14	2.359	3.424	-0.215	1.00	0.00	H
ATOM	224	CG	TYR	14	2.289	1.357	0.006	1.00	0.00	C
ATOM	225	CD1	TYR	14	1.678	1.548	1.247	1.00	0.00	C
ATOM	226	HD1	TYR	14	1.472	2.538	1.628	1.00	0.00	H
ATOM	227	CE1	TYR	14	1.282	0.420	2.007	1.00	0.00	C
ATOM	228	HE1	TYR	14	0.892	0.539	3.007	1.00	0.00	H
ATOM	229	CZ	TYR	14	1.505	-0.898	1.581	1.00	0.00	C
ATOM	230	OH	TYR	14	1.212	-1.899	2.395	1.00	0.00	O
ATOM	231	HH	TYR	14	1.293	-2.738	1.935	1.00	0.00	H
ATOM	232	CE2	TYR	14	2.074	-1.094	0.318	1.00	0.00	C
ATOM	233	HE2	TYR	14	2.381	-2.026	-0.133	1.00	0.00	H
ATOM	234	CD2	TYR	14	2.432	0.016	-0.526	1.00	0.00	C
ATOM	235	HD2	TYR	14	2.882	-0.190	-1.485	1.00	0.00	H
ATOM	236	C	TYR	14	4.324	4.042	-2.126	1.00	0.00	C
ATOM	237	O	TYR	14	3.893	3.972	-3.248	1.00	0.00	O
ATOM	238	N	ARG	15	5.092	5.095	-1.688	1.00	0.00	N
ATOM	239	H	ARG	15	5.481	4.984	-0.763	1.00	0.00	H
ATOM	240	CA	ARG	15	5.518	6.288	-2.510	1.00	0.00	C
ATOM	241	HA	ARG	15	4.815	6.362	-3.341	1.00	0.00	H
ATOM	242	CB	ARG	15	5.739	7.574	-1.707	1.00	0.00	C
ATOM	243	HB2	ARG	15	6.156	8.374	-2.318	1.00	0.00	H
ATOM	244	HB3	ARG	15	6.396	7.284	-0.887	1.00	0.00	H
ATOM	245	CG	ARG	15	4.419	8.109	-1.146	1.00	0.00	C
ATOM	246	HG2	ARG	15	4.700	8.795	-0.347	1.00	0.00	H
ATOM	247	HG3	ARG	15	4.008	7.216	-0.676	1.00	0.00	H
ATOM	248	CD	ARG	15	3.401	8.789	-2.160	1.00	0.00	C
ATOM	249	HD2	ARG	15	2.487	9.164	-1.700	1.00	0.00	H
ATOM	250	HD3	ARG	15	3.236	7.999	-2.893	1.00	0.00	H

```
ATOM    251  NE  ARG    15       3.939   9.946  -2.880  1.00  0.00           N
ATOM    252  HE  ARG    15       4.531  10.560  -2.339  1.00  0.00           H
ATOM    253  CZ  ARG    15       3.623  10.269  -4.119  1.00  0.00           C
ATOM    254  NH1 ARG    15       3.893  11.470  -4.572  1.00  0.00           N
ATOM    255 HH11 ARG    15       3.442  11.830  -5.401  1.00  0.00           H
ATOM    256 HH12 ARG    15       4.383  12.111  -3.964  1.00  0.00           H
ATOM    257  NH2 ARG    15       2.980   9.493  -4.906  1.00  0.00           N
ATOM    258 HH21 ARG    15       2.828   9.658  -5.891  1.00  0.00           H
ATOM    259 HH22 ARG    15       2.671   8.595  -4.562  1.00  0.00           H
ATOM    260  C   ARG    15       6.803   6.029  -3.307  1.00  0.00           C
ATOM    261  O   ARG    15       7.203   6.926  -4.097  1.00  0.00           O
ATOM    262  N   TYR    16       7.401   4.846  -3.128  1.00  0.00           N
ATOM    263  H   TYR    16       6.847   4.253  -2.526  1.00  0.00           H
ATOM    264  CA  TYR    16       8.719   4.417  -3.682  1.00  0.00           C
ATOM    265  HA  TYR    16       9.141   5.312  -4.138  1.00  0.00           H
ATOM    266  CB  TYR    16       9.578   3.850  -2.558  1.00  0.00           C
ATOM    267  HB2 TYR    16       9.293   3.127  -1.793  1.00  0.00           H
ATOM    268  HB3 TYR    16       9.726   4.744  -1.952  1.00  0.00           H
ATOM    269  CG  TYR    16      10.946   3.497  -3.057  1.00  0.00           C
ATOM    270  CD1 TYR    16      11.291   2.101  -3.268  1.00  0.00           C
ATOM    271  HD1 TYR    16      10.628   1.279  -3.045  1.00  0.00           H
ATOM    272  CE1 TYR    16      12.604   1.782  -3.638  1.00  0.00           C
ATOM    273  HE1 TYR    16      12.844   0.730  -3.681  1.00  0.00           H
ATOM    274  CZ  TYR    16      13.500   2.845  -3.920  1.00  0.00           C
ATOM    275  OH  TYR    16      14.745   2.542  -4.426  1.00  0.00           O
ATOM    276  HH  TYR    16      15.234   3.296  -4.763  1.00  0.00           H
ATOM    277  CE2 TYR    16      13.143   4.190  -3.775  1.00  0.00           C
ATOM    278  HE2 TYR    16      13.864   4.980  -3.928  1.00  0.00           H
ATOM    279  CD2 TYR    16      11.885   4.546  -3.328  1.00  0.00           C
ATOM    280  HD2 TYR    16      11.699   5.571  -3.042  1.00  0.00           H
ATOM    281  C   TYR    16       8.496   3.358  -4.769  1.00  0.00           C
ATOM    282  O   TYR    16       7.693   2.436  -4.611  1.00  0.00           O
ATOM    283  N   NME    17       9.183   3.545  -5.884  1.00  0.00           N
ATOM    284  H   NME    17       9.887   4.269  -5.913  1.00  0.00           H
ATOM    285  CH3 NME    17       9.124   2.628  -6.991  1.00  0.00           C
ATOM    286 HH31 NME    17       9.492   3.175  -7.858  1.00  0.00           H
ATOM    287 HH32 NME    17       9.786   1.769  -6.880  1.00  0.00           H
ATOM    288 HH33 NME    17       8.108   2.281  -7.180  1.00  0.00           H
TER     289      NME    17
END
```

Cluster 8:

```
ATOM      1 HH31 ACE     1      18.749  10.074   6.378  1.00  0.00           H
ATOM      2  CH3 ACE     1      19.746   9.952   5.955  1.00  0.00           C
ATOM      3 HH32 ACE     1      20.372  10.249   6.797  1.00  0.00           H
ATOM      4 HH33 ACE     1      19.859  10.659   5.134  1.00  0.00           H
ATOM      5  C   ACE     1      20.046   8.541   5.603  1.00  0.00           C
ATOM      6  O   ACE     1      20.540   8.349   4.510  1.00  0.00           O
ATOM      7  N   ASN     2      19.695   7.589   6.469  1.00  0.00           N
ATOM      8  H   ASN     2      19.356   7.925   7.360  1.00  0.00           H
ATOM      9  CA  ASN     2      19.754   6.151   6.169  1.00  0.00           C
ATOM     10  HA  ASN     2      20.052   5.996   5.132  1.00  0.00           H
ATOM     11  CB  ASN     2      20.873   5.482   7.029  1.00  0.00           C
ATOM     12  HB2 ASN     2      20.631   5.309   8.078  1.00  0.00           H
ATOM     13  HB3 ASN     2      21.744   6.136   6.987  1.00  0.00           H
ATOM     14  CG  ASN     2      21.334   4.084   6.603  1.00  0.00           C
ATOM     15  OD1 ASN     2      21.013   3.608   5.507  1.00  0.00           O
ATOM     16  ND2 ASN     2      22.321   3.512   7.303  1.00  0.00           N
ATOM     17 HD21 ASN     2      22.694   2.618   7.014  1.00  0.00           H
ATOM     18 HD22 ASN     2      22.521   3.844   8.235  1.00  0.00           H
```

ATOM	19	C	ASN	2	18.429	5.347	6.336	1.00	0.00	C
ATOM	20	O	ASN	2	17.484	5.852	6.961	1.00	0.00	O
ATOM	21	N	ASP	3	18.342	4.134	5.771	1.00	0.00	N
ATOM	22	H	ASP	3	19.083	3.824	5.158	1.00	0.00	H
ATOM	23	CA	ASP	3	17.112	3.331	5.651	1.00	0.00	C
ATOM	24	HA	ASP	3	16.305	4.031	5.428	1.00	0.00	H
ATOM	25	CB	ASP	3	17.219	2.454	4.363	1.00	0.00	C
ATOM	26	HB2	ASP	3	18.103	1.842	4.182	1.00	0.00	H
ATOM	27	HB3	ASP	3	17.258	3.326	3.711	1.00	0.00	H
ATOM	28	CG	ASP	3	15.976	1.577	4.071	1.00	0.00	C
ATOM	29	OD1	ASP	3	14.871	1.968	4.436	1.00	0.00	O
ATOM	30	OD2	ASP	3	16.103	0.592	3.357	1.00	0.00	O
ATOM	31	C	ASP	3	16.797	2.554	6.938	1.00	0.00	C
ATOM	32	O	ASP	3	16.715	1.323	6.859	1.00	0.00	O
ATOM	33	N	TYR	4	16.669	3.219	8.069	1.00	0.00	N
ATOM	34	H	TYR	4	16.668	4.228	8.018	1.00	0.00	H
ATOM	35	CA	TYR	4	16.114	2.697	9.282	1.00	0.00	C
ATOM	36	HA	TYR	4	16.735	1.878	9.645	1.00	0.00	H
ATOM	37	CB	TYR	4	16.122	3.788	10.356	1.00	0.00	C
ATOM	38	HB2	TYR	4	15.818	3.374	11.317	1.00	0.00	H
ATOM	39	HB3	TYR	4	15.258	4.409	10.123	1.00	0.00	H
ATOM	40	CG	TYR	4	17.392	4.569	10.524	1.00	0.00	C
ATOM	41	CD1	TYR	4	17.322	5.913	11.034	1.00	0.00	C
ATOM	42	HD1	TYR	4	16.373	6.377	11.258	1.00	0.00	H
ATOM	43	CE1	TYR	4	18.496	6.586	11.360	1.00	0.00	C
ATOM	44	HE1	TYR	4	18.420	7.534	11.873	1.00	0.00	H
ATOM	45	CZ	TYR	4	19.789	5.984	11.143	1.00	0.00	C
ATOM	46	OH	TYR	4	21.004	6.598	11.468	1.00	0.00	O
ATOM	47	HH	TYR	4	21.760	6.091	11.160	1.00	0.00	H
ATOM	48	CE2	TYR	4	19.848	4.650	10.657	1.00	0.00	C
ATOM	49	HE2	TYR	4	20.797	4.231	10.358	1.00	0.00	H
ATOM	50	CD2	TYR	4	18.659	3.945	10.303	1.00	0.00	C
ATOM	51	HD2	TYR	4	18.821	3.089	9.665	1.00	0.00	H
ATOM	52	C	TYR	4	14.629	2.233	9.047	1.00	0.00	C
ATOM	53	O	TYR	4	13.915	2.664	8.144	1.00	0.00	O
ATOM	54	N	GLU	5	14.162	1.510	10.070	1.00	0.00	N
ATOM	55	H	GLU	5	14.851	1.369	10.795	1.00	0.00	H
ATOM	56	CA	GLU	5	12.841	0.785	10.254	1.00	0.00	C
ATOM	57	HA	GLU	5	12.406	0.569	9.278	1.00	0.00	H
ATOM	58	CB	GLU	5	13.052	-0.545	10.998	1.00	0.00	C
ATOM	59	HB2	GLU	5	12.100	-1.074	11.062	1.00	0.00	H
ATOM	60	HB3	GLU	5	13.284	-0.362	12.047	1.00	0.00	H
ATOM	61	CG	GLU	5	14.038	-1.451	10.353	1.00	0.00	C
ATOM	62	HG2	GLU	5	14.061	-2.393	10.901	1.00	0.00	H
ATOM	63	HG3	GLU	5	15.027	-0.993	10.395	1.00	0.00	H
ATOM	64	CD	GLU	5	13.593	-1.819	8.898	1.00	0.00	C
ATOM	65	OE1	GLU	5	14.449	-1.959	7.973	1.00	0.00	O
ATOM	66	OE2	GLU	5	12.388	-1.945	8.628	1.00	0.00	O
ATOM	67	C	GLU	5	11.769	1.692	10.865	1.00	0.00	C
ATOM	68	O	GLU	5	10.675	1.249	11.164	1.00	0.00	O
ATOM	69	N	ASP	6	12.054	2.961	11.125	1.00	0.00	N
ATOM	70	H	ASP	6	12.989	3.318	10.984	1.00	0.00	H
ATOM	71	CA	ASP	6	11.019	3.860	11.722	1.00	0.00	C
ATOM	72	HA	ASP	6	10.625	3.354	12.604	1.00	0.00	H
ATOM	73	CB	ASP	6	11.586	5.106	12.353	1.00	0.00	C
ATOM	74	HB2	ASP	6	11.990	5.786	11.604	1.00	0.00	H
ATOM	75	HB3	ASP	6	12.438	4.766	12.940	1.00	0.00	H
ATOM	76	CG	ASP	6	10.629	5.805	13.354	1.00	0.00	C
ATOM	77	OD1	ASP	6	9.939	5.049	14.128	1.00	0.00	O
ATOM	78	OD2	ASP	6	10.707	7.084	13.412	1.00	0.00	O
ATOM	79	C	ASP	6	9.844	4.133	10.845	1.00	0.00	C
ATOM	80	O	ASP	6	10.119	4.476	9.697	1.00	0.00	O
ATOM	81	N	ARG	7	8.595	4.056	11.328	1.00	0.00	N

ATOM	82	H	ARG	7	8.318	3.727	12.242	1.00	0.00	H
ATOM	83	CA	ARG	7	7.380	4.360	10.540	1.00	0.00	C
ATOM	84	HA	ARG	7	7.427	3.680	9.690	1.00	0.00	H
ATOM	85	CB	ARG	7	6.095	4.014	11.347	1.00	0.00	C
ATOM	86	HB2	ARG	7	5.196	4.147	10.745	1.00	0.00	H
ATOM	87	HB3	ARG	7	6.078	4.660	12.225	1.00	0.00	H
ATOM	88	CG	ARG	7	6.035	2.466	11.605	1.00	0.00	C
ATOM	89	HG2	ARG	7	6.976	2.180	12.074	1.00	0.00	H
ATOM	90	HG3	ARG	7	5.939	1.968	10.640	1.00	0.00	H
ATOM	91	CD	ARG	7	4.928	2.056	12.555	1.00	0.00	C
ATOM	92	HD2	ARG	7	5.093	2.634	13.465	1.00	0.00	H
ATOM	93	HD3	ARG	7	5.163	0.991	12.565	1.00	0.00	H
ATOM	94	NE	ARG	7	3.619	2.348	12.017	1.00	0.00	N
ATOM	95	HE	ARG	7	3.552	2.285	11.012	1.00	0.00	H
ATOM	96	CZ	ARG	7	2.511	2.592	12.681	1.00	0.00	C
ATOM	97	NH1	ARG	7	2.380	2.640	13.962	1.00	0.00	N
ATOM	98	HH11	ARG	7	3.177	2.622	14.582	1.00	0.00	H
ATOM	99	HH12	ARG	7	1.488	3.019	14.245	1.00	0.00	H
ATOM	100	NH2	ARG	7	1.482	2.707	11.963	1.00	0.00	N
ATOM	101	HH21	ARG	7	0.583	2.855	12.400	1.00	0.00	H
ATOM	102	HH22	ARG	7	1.650	2.665	10.969	1.00	0.00	H
ATOM	103	C	ARG	7	7.329	5.784	9.910	1.00	0.00	C
ATOM	104	O	ARG	7	6.527	6.001	9.026	1.00	0.00	O
ATOM	105	N	TYR	8	8.295	6.636	10.209	1.00	0.00	N
ATOM	106	H	TYR	8	8.833	6.444	11.041	1.00	0.00	H
ATOM	107	CA	TYR	8	8.404	7.965	9.543	1.00	0.00	C
ATOM	108	HA	TYR	8	7.370	8.271	9.385	1.00	0.00	H
ATOM	109	CB	TYR	8	8.999	9.076	10.486	1.00	0.00	C
ATOM	110	HB2	TYR	8	10.066	8.957	10.674	1.00	0.00	H
ATOM	111	HB3	TYR	8	8.453	9.018	11.428	1.00	0.00	H
ATOM	112	CG	TYR	8	9.045	10.387	9.733	1.00	0.00	C
ATOM	113	CD1	TYR	8	10.188	10.763	9.082	1.00	0.00	C
ATOM	114	HD1	TYR	8	11.045	10.120	8.951	1.00	0.00	H
ATOM	115	CE1	TYR	8	10.267	12.123	8.567	1.00	0.00	C
ATOM	116	HE1	TYR	8	11.152	12.530	8.100	1.00	0.00	H
ATOM	117	CZ	TYR	8	9.125	12.896	8.714	1.00	0.00	C
ATOM	118	OH	TYR	8	9.202	14.122	8.184	1.00	0.00	O
ATOM	119	HH	TYR	8	8.432	14.655	8.396	1.00	0.00	H
ATOM	120	CE2	TYR	8	7.955	12.508	9.314	1.00	0.00	C
ATOM	121	HE2	TYR	8	7.129	13.187	9.467	1.00	0.00	H
ATOM	122	CD2	TYR	8	7.960	11.275	9.975	1.00	0.00	C
ATOM	123	HD2	TYR	8	7.161	10.918	10.608	1.00	0.00	H
ATOM	124	C	TYR	8	9.062	7.788	8.215	1.00	0.00	C
ATOM	125	O	TYR	8	9.005	8.678	7.337	1.00	0.00	O
ATOM	126	N	TYR	9	9.728	6.613	7.943	1.00	0.00	N
ATOM	127	H	TYR	9	9.731	6.028	8.766	1.00	0.00	H
ATOM	128	CA	TYR	9	10.346	6.225	6.628	1.00	0.00	C
ATOM	129	HA	TYR	9	10.243	7.067	5.944	1.00	0.00	H
ATOM	130	CB	TYR	9	11.898	6.156	6.728	1.00	0.00	C
ATOM	131	HB2	TYR	9	12.443	5.725	5.887	1.00	0.00	H
ATOM	132	HB3	TYR	9	11.851	5.416	7.527	1.00	0.00	H
ATOM	133	CG	TYR	9	12.544	7.408	7.234	1.00	0.00	C
ATOM	134	CD1	TYR	9	12.733	7.684	8.633	1.00	0.00	C
ATOM	135	HD1	TYR	9	12.421	6.920	9.331	1.00	0.00	H
ATOM	136	CE1	TYR	9	13.547	8.751	9.105	1.00	0.00	C
ATOM	137	HE1	TYR	9	13.743	8.851	10.162	1.00	0.00	H
ATOM	138	CZ	TYR	9	14.092	9.643	8.083	1.00	0.00	C
ATOM	139	OH	TYR	9	14.891	10.702	8.395	1.00	0.00	O
ATOM	140	HH	TYR	9	14.938	10.729	9.353	1.00	0.00	H
ATOM	141	CE2	TYR	9	13.827	9.390	6.754	1.00	0.00	C
ATOM	142	HE2	TYR	9	14.256	9.986	5.962	1.00	0.00	H
ATOM	143	CD2	TYR	9	12.957	8.366	6.332	1.00	0.00	C
ATOM	144	HD2	TYR	9	12.666	8.309	5.293	1.00	0.00	H

ATOM	145	C	TYR	9	9.831	4.949	5.959	1.00	0.00	C
ATOM	146	O	TYR	9	9.296	4.965	4.855	1.00	0.00	O
ATOM	147	N	ARG	10	9.821	3.887	6.767	1.00	0.00	N
ATOM	148	H	ARG	10	10.214	3.957	7.695	1.00	0.00	H
ATOM	149	CA	ARG	10	9.375	2.508	6.322	1.00	0.00	C
ATOM	150	HA	ARG	10	9.953	2.253	5.434	1.00	0.00	H
ATOM	151	CB	ARG	10	9.679	1.431	7.344	1.00	0.00	C
ATOM	152	HB2	ARG	10	9.061	1.664	8.213	1.00	0.00	H
ATOM	153	HB3	ARG	10	10.732	1.572	7.585	1.00	0.00	H
ATOM	154	CG	ARG	10	9.346	-0.072	7.006	1.00	0.00	C
ATOM	155	HG2	ARG	10	8.288	-0.202	6.773	1.00	0.00	H
ATOM	156	HG3	ARG	10	9.533	-0.603	7.939	1.00	0.00	H
ATOM	157	CD	ARG	10	10.144	-0.854	5.897	1.00	0.00	C
ATOM	158	HD2	ARG	10	9.819	-0.522	4.911	1.00	0.00	H
ATOM	159	HD3	ARG	10	9.936	-1.909	6.073	1.00	0.00	H
ATOM	160	NE	ARG	10	11.596	-0.817	6.032	1.00	0.00	N
ATOM	161	HE	ARG	10	11.857	-1.393	6.820	1.00	0.00	H
ATOM	162	CZ	ARG	10	12.569	-0.149	5.449	1.00	0.00	C
ATOM	163	NH1	ARG	10	12.261	0.571	4.416	1.00	0.00	N
ATOM	164	HH11	ARG	10	11.326	0.533	4.037	1.00	0.00	H
ATOM	165	HH12	ARG	10	13.079	1.050	4.067	1.00	0.00	H
ATOM	166	NH2	ARG	10	13.778	-0.093	5.908	1.00	0.00	N
ATOM	167	HH21	ARG	10	14.474	0.480	5.455	1.00	0.00	H
ATOM	168	HH22	ARG	10	13.987	-0.631	6.737	1.00	0.00	H
ATOM	169	C	ARG	10	7.913	2.458	5.879	1.00	0.00	C
ATOM	170	O	ARG	10	7.590	1.544	5.072	1.00	0.00	O
ATOM	171	N	GLU	11	6.964	3.255	6.384	1.00	0.00	N
ATOM	172	H	GLU	11	7.215	3.712	7.250	1.00	0.00	H
ATOM	173	CA	GLU	11	5.538	3.287	5.932	1.00	0.00	C
ATOM	174	HA	GLU	11	5.390	2.404	5.310	1.00	0.00	H
ATOM	175	CB	GLU	11	4.698	3.312	7.279	1.00	0.00	C
ATOM	176	HB2	GLU	11	4.945	4.196	7.867	1.00	0.00	H
ATOM	177	HB3	GLU	11	5.001	2.399	7.793	1.00	0.00	H
ATOM	178	CG	GLU	11	3.177	3.362	7.110	1.00	0.00	C
ATOM	179	HG2	GLU	11	2.907	2.565	6.418	1.00	0.00	H
ATOM	180	HG3	GLU	11	2.906	4.266	6.565	1.00	0.00	H
ATOM	181	CD	GLU	11	2.383	3.165	8.422	1.00	0.00	C
ATOM	182	OE1	GLU	11	1.268	3.756	8.550	1.00	0.00	O
ATOM	183	OE2	GLU	11	2.720	2.357	9.374	1.00	0.00	O
ATOM	184	C	GLU	11	5.266	4.525	5.055	1.00	0.00	C
ATOM	185	O	GLU	11	4.161	4.652	4.688	1.00	0.00	O
ATOM	186	N	ASN	12	6.227	5.385	4.750	1.00	0.00	N
ATOM	187	H	ASN	12	7.177	5.068	4.882	1.00	0.00	H
ATOM	188	CA	ASN	12	5.987	6.666	4.115	1.00	0.00	C
ATOM	189	HA	ASN	12	5.101	7.158	4.518	1.00	0.00	H
ATOM	190	CB	ASN	12	7.238	7.580	4.356	1.00	0.00	C
ATOM	191	HB2	ASN	12	8.073	7.386	3.682	1.00	0.00	H
ATOM	192	HB3	ASN	12	7.628	7.372	5.352	1.00	0.00	H
ATOM	193	CG	ASN	12	6.844	9.025	4.384	1.00	0.00	C
ATOM	194	OD1	ASN	12	6.212	9.438	3.442	1.00	0.00	O
ATOM	195	ND2	ASN	12	7.357	9.837	5.339	1.00	0.00	N
ATOM	196	HD21	ASN	12	7.221	10.821	5.155	1.00	0.00	H
ATOM	197	HD22	ASN	12	8.056	9.518	5.995	1.00	0.00	H
ATOM	198	C	ASN	12	5.834	6.520	2.579	1.00	0.00	C
ATOM	199	O	ASN	12	6.586	5.788	1.957	1.00	0.00	O
ATOM	200	N	MET	13	5.043	7.385	2.014	1.00	0.00	N
ATOM	201	H	MET	13	4.398	7.942	2.557	1.00	0.00	H
ATOM	202	CA	MET	13	4.872	7.529	0.568	1.00	0.00	C
ATOM	203	HA	MET	13	4.554	6.613	0.069	1.00	0.00	H
ATOM	204	CB	MET	13	3.818	8.616	0.447	1.00	0.00	C
ATOM	205	HB2	MET	13	4.029	9.502	1.046	1.00	0.00	H
ATOM	206	HB3	MET	13	2.981	8.061	0.871	1.00	0.00	H
ATOM	207	CG	MET	13	3.414	8.981	-0.950	1.00	0.00	C

ATOM	208	HG2	MET	13	3.586	8.177	-1.666	1.00	0.00	H
ATOM	209	HG3	MET	13	4.007	9.875	-1.141	1.00	0.00	H
ATOM	210	SD	MET	13	1.553	9.332	-0.992	1.00	0.00	S
ATOM	211	CE	MET	13	1.523	10.871	0.040	1.00	0.00	C
ATOM	212	HE1	MET	13	2.260	11.625	-0.239	1.00	0.00	H
ATOM	213	HE2	MET	13	0.613	11.447	-0.125	1.00	0.00	H
ATOM	214	HE3	MET	13	1.747	10.487	1.035	1.00	0.00	H
ATOM	215	C	MET	13	6.220	7.950	-0.157	1.00	0.00	C
ATOM	216	O	MET	13	6.413	7.435	-1.264	1.00	0.00	O
ATOM	217	N	TYR	14	7.071	8.744	0.564	1.00	0.00	N
ATOM	218	H	TYR	14	6.774	9.178	1.426	1.00	0.00	H
ATOM	219	CA	TYR	14	8.240	9.328	-0.093	1.00	0.00	C
ATOM	220	HA	TYR	14	7.869	9.486	-1.105	1.00	0.00	H
ATOM	221	CB	TYR	14	8.556	10.672	0.589	1.00	0.00	C
ATOM	222	HB2	TYR	14	9.456	11.133	0.180	1.00	0.00	H
ATOM	223	HB3	TYR	14	8.664	10.420	1.643	1.00	0.00	H
ATOM	224	CG	TYR	14	7.482	11.696	0.410	1.00	0.00	C
ATOM	225	CD1	TYR	14	6.856	12.279	1.579	1.00	0.00	C
ATOM	226	HD1	TYR	14	7.151	12.155	2.610	1.00	0.00	H
ATOM	227	CE1	TYR	14	5.912	13.350	1.362	1.00	0.00	C
ATOM	228	HE1	TYR	14	5.447	13.863	2.190	1.00	0.00	H
ATOM	229	CZ	TYR	14	5.559	13.753	0.046	1.00	0.00	C
ATOM	230	OH	TYR	14	4.669	14.835	-0.129	1.00	0.00	O
ATOM	231	HH	TYR	14	4.486	14.868	-1.070	1.00	0.00	H
ATOM	232	CE2	TYR	14	6.242	13.235	-1.097	1.00	0.00	C
ATOM	233	HE2	TYR	14	6.006	13.447	-2.130	1.00	0.00	H
ATOM	234	CD2	TYR	14	7.197	12.195	-0.897	1.00	0.00	C
ATOM	235	HD2	TYR	14	7.635	11.754	-1.780	1.00	0.00	H
ATOM	236	C	TYR	14	9.501	8.345	-0.182	1.00	0.00	C
ATOM	237	O	TYR	14	10.479	8.609	-0.889	1.00	0.00	O
ATOM	238	N	ARG	15	9.476	7.193	0.476	1.00	0.00	N
ATOM	239	H	ARG	15	8.615	7.081	0.993	1.00	0.00	H
ATOM	240	CA	ARG	15	10.505	6.127	0.469	1.00	0.00	C
ATOM	241	HA	ARG	15	11.482	6.609	0.498	1.00	0.00	H
ATOM	242	CB	ARG	15	10.339	5.285	1.808	1.00	0.00	C
ATOM	243	HB2	ARG	15	9.334	4.864	1.807	1.00	0.00	H
ATOM	244	HB3	ARG	15	10.539	5.952	2.647	1.00	0.00	H
ATOM	245	CG	ARG	15	11.236	4.039	2.058	1.00	0.00	C
ATOM	246	HG2	ARG	15	10.931	3.238	1.384	1.00	0.00	H
ATOM	247	HG3	ARG	15	10.957	3.748	3.070	1.00	0.00	H
ATOM	248	CD	ARG	15	12.692	4.345	1.953	1.00	0.00	C
ATOM	249	HD2	ARG	15	12.936	5.086	2.715	1.00	0.00	H
ATOM	250	HD3	ARG	15	12.852	4.747	0.953	1.00	0.00	H
ATOM	251	NE	ARG	15	13.538	3.168	1.920	1.00	0.00	N
ATOM	252	HE	ARG	15	14.093	3.056	2.755	1.00	0.00	H
ATOM	253	CZ	ARG	15	13.849	2.336	0.921	1.00	0.00	C
ATOM	254	NH1	ARG	15	13.477	2.462	-0.358	1.00	0.00	N
ATOM	255	HH11	ARG	15	12.885	3.257	-0.553	1.00	0.00	H
ATOM	256	HH12	ARG	15	13.802	1.845	-1.089	1.00	0.00	H
ATOM	257	NH2	ARG	15	14.712	1.310	1.052	1.00	0.00	N
ATOM	258	HH21	ARG	15	15.148	1.163	1.952	1.00	0.00	H
ATOM	259	HH22	ARG	15	14.771	0.547	0.394	1.00	0.00	H
ATOM	260	C	ARG	15	10.519	5.212	-0.768	1.00	0.00	C
ATOM	261	O	ARG	15	11.477	4.515	-1.063	1.00	0.00	O
ATOM	262	N	TYR	16	9.553	5.394	-1.675	1.00	0.00	N
ATOM	263	H	TYR	16	8.971	6.214	-1.583	1.00	0.00	H
ATOM	264	CA	TYR	16	9.283	4.615	-2.879	1.00	0.00	C
ATOM	265	HA	TYR	16	10.162	4.014	-3.108	1.00	0.00	H
ATOM	266	CB	TYR	16	8.216	3.591	-2.565	1.00	0.00	C
ATOM	267	HB2	TYR	16	8.173	2.886	-3.395	1.00	0.00	H
ATOM	268	HB3	TYR	16	7.373	4.281	-2.601	1.00	0.00	H
ATOM	269	CG	TYR	16	8.259	2.809	-1.207	1.00	0.00	C
ATOM	270	CD1	TYR	16	7.362	3.245	-0.196	1.00	0.00	C

```
ATOM    271  HD1 TYR    16       6.594   3.968  -0.431  1.00  0.00           H
ATOM    272  CE1 TYR    16       7.469   2.634   1.104  1.00  0.00           C
ATOM    273  HE1 TYR    16       6.726   2.889   1.845  1.00  0.00           H
ATOM    274  CZ  TYR    16       8.463   1.614   1.383  1.00  0.00           C
ATOM    275  OH  TYR    16       8.488   1.004   2.637  1.00  0.00           O
ATOM    276  HH  TYR    16       7.770   1.382   3.150  1.00  0.00           H
ATOM    277  CE2 TYR    16       9.238   1.154   0.321  1.00  0.00           C
ATOM    278  HE2 TYR    16       9.917   0.341   0.533  1.00  0.00           H
ATOM    279  CD2 TYR    16       9.204   1.735  -1.010  1.00  0.00           C
ATOM    280  HD2 TYR    16       9.819   1.450  -1.850  1.00  0.00           H
ATOM    281  C   TYR    16       9.114   5.342  -4.205  1.00  0.00           C
ATOM    282  O   TYR    16       8.582   4.818  -5.201  1.00  0.00           O
ATOM    283  N   NME    17       9.621   6.598  -4.284  1.00  0.00           N
ATOM    284  H   NME    17       9.798   7.067  -3.407  1.00  0.00           H
ATOM    285  CH3 NME    17       9.730   7.366  -5.609  1.00  0.00           C
ATOM    286 HH31 NME    17       8.742   7.529  -6.040  1.00  0.00           H
ATOM    287 HH32 NME    17      10.130   8.365  -5.435  1.00  0.00           H
ATOM    288 HH33 NME    17      10.378   6.941  -6.377  1.00  0.00           H
TER     289      NME    17
END
```

Cluster 9:

```
ATOM      1 HH31 ACE     1      16.009   1.087   0.224  1.00  0.00           H
ATOM      2  CH3 ACE     1      16.866   1.759   0.267  1.00  0.00           C
ATOM      3 HH32 ACE     1      16.593   2.814   0.235  1.00  0.00           H
ATOM      4 HH33 ACE     1      17.439   1.503  -0.624  1.00  0.00           H
ATOM      5  C   ACE     1      17.635   1.408   1.527  1.00  0.00           C
ATOM      6  O   ACE     1      17.513   0.325   2.099  1.00  0.00           O
ATOM      7  N   ASN     2      18.544   2.351   1.888  1.00  0.00           N
ATOM      8  H   ASN     2      18.454   3.234   1.405  1.00  0.00           H
ATOM      9  CA  ASN     2      19.304   2.246   3.171  1.00  0.00           C
ATOM     10  HA  ASN     2      19.631   1.206   3.186  1.00  0.00           H
ATOM     11  CB  ASN     2      20.657   3.025   3.126  1.00  0.00           C
ATOM     12  HB2 ASN     2      20.569   4.105   3.009  1.00  0.00           H
ATOM     13  HB3 ASN     2      21.092   2.778   2.157  1.00  0.00           H
ATOM     14  CG  ASN     2      21.768   2.732   4.154  1.00  0.00           C
ATOM     15  OD1 ASN     2      21.588   2.020   5.132  1.00  0.00           O
ATOM     16  ND2 ASN     2      22.944   3.084   3.822  1.00  0.00           N
ATOM     17 HD21 ASN     2      23.696   2.769   4.419  1.00  0.00           H
ATOM     18 HD22 ASN     2      23.133   3.516   2.929  1.00  0.00           H
ATOM     19  C   ASN     2      18.329   2.648   4.301  1.00  0.00           C
ATOM     20  O   ASN     2      17.475   3.527   4.203  1.00  0.00           O
ATOM     21  N   ASP     3      18.436   1.941   5.449  1.00  0.00           N
ATOM     22  H   ASP     3      19.221   1.309   5.517  1.00  0.00           H
ATOM     23  CA  ASP     3      17.520   2.072   6.603  1.00  0.00           C
ATOM     24  HA  ASP     3      16.508   2.200   6.218  1.00  0.00           H
ATOM     25  CB  ASP     3      17.622   0.794   7.491  1.00  0.00           C
ATOM     26  HB2 ASP     3      18.452   0.741   8.196  1.00  0.00           H
ATOM     27  HB3 ASP     3      17.846  -0.015   6.795  1.00  0.00           H
ATOM     28  CG  ASP     3      16.328   0.509   8.317  1.00  0.00           C
ATOM     29  OD1 ASP     3      16.537  -0.208   9.335  1.00  0.00           O
ATOM     30  OD2 ASP     3      15.223   0.926   7.988  1.00  0.00           O
ATOM     31  C   ASP     3      17.797   3.375   7.406  1.00  0.00           C
ATOM     32  O   ASP     3      18.924   3.832   7.620  1.00  0.00           O
ATOM     33  N   TYR     4      16.713   3.922   8.010  1.00  0.00           N
ATOM     34  H   TYR     4      15.803   3.500   7.890  1.00  0.00           H
ATOM     35  CA  TYR     4      16.743   5.058   8.913  1.00  0.00           C
ATOM     36  HA  TYR     4      17.688   4.948   9.445  1.00  0.00           H
ATOM     37  CB  TYR     4      16.683   6.371   8.146  1.00  0.00           C
ATOM     38  HB2 TYR     4      15.673   6.447   7.744  1.00  0.00           H
```

ATOM	39	HB3	TYR	4	17.349	6.125	7.319	1.00	0.00	H
ATOM	40	CG	TYR	4	17.181	7.630	8.827	1.00	0.00	C
ATOM	41	CD1	TYR	4	16.286	8.752	8.819	1.00	0.00	C
ATOM	42	HD1	TYR	4	15.366	8.733	8.253	1.00	0.00	H
ATOM	43	CE1	TYR	4	16.634	9.859	9.627	1.00	0.00	C
ATOM	44	HE1	TYR	4	15.948	10.693	9.654	1.00	0.00	H
ATOM	45	CZ	TYR	4	17.851	9.904	10.399	1.00	0.00	C
ATOM	46	OH	TYR	4	18.099	10.993	11.198	1.00	0.00	O
ATOM	47	HH	TYR	4	18.939	10.887	11.648	1.00	0.00	H
ATOM	48	CE2	TYR	4	18.773	8.848	10.260	1.00	0.00	C
ATOM	49	HE2	TYR	4	19.749	8.851	10.722	1.00	0.00	H
ATOM	50	CD2	TYR	4	18.412	7.686	9.555	1.00	0.00	C
ATOM	51	HD2	TYR	4	19.077	6.835	9.555	1.00	0.00	H
ATOM	52	C	TYR	4	15.573	4.842	9.910	1.00	0.00	C
ATOM	53	O	TYR	4	14.734	3.939	9.858	1.00	0.00	O
ATOM	54	N	GLU	5	15.510	5.797	10.822	1.00	0.00	N
ATOM	55	H	GLU	5	16.101	6.614	10.889	1.00	0.00	H
ATOM	56	CA	GLU	5	14.558	5.749	11.945	1.00	0.00	C
ATOM	57	HA	GLU	5	14.469	4.739	12.346	1.00	0.00	H
ATOM	58	CB	GLU	5	15.157	6.465	13.167	1.00	0.00	C
ATOM	59	HB2	GLU	5	16.199	6.171	13.291	1.00	0.00	H
ATOM	60	HB3	GLU	5	14.549	6.089	13.990	1.00	0.00	H
ATOM	61	CG	GLU	5	15.235	8.046	13.144	1.00	0.00	C
ATOM	62	HG2	GLU	5	14.252	8.446	13.391	1.00	0.00	H
ATOM	63	HG3	GLU	5	15.651	8.386	12.195	1.00	0.00	H
ATOM	64	CD	GLU	5	16.119	8.595	14.274	1.00	0.00	C
ATOM	65	OE1	GLU	5	15.571	8.818	15.402	1.00	0.00	O
ATOM	66	OE2	GLU	5	17.334	8.732	14.063	1.00	0.00	O
ATOM	67	C	GLU	5	13.137	6.173	11.492	1.00	0.00	C
ATOM	68	O	GLU	5	12.162	6.115	12.258	1.00	0.00	O
ATOM	69	N	ASP	6	12.893	6.422	10.175	1.00	0.00	N
ATOM	70	H	ASP	6	13.671	6.406	9.531	1.00	0.00	H
ATOM	71	CA	ASP	6	11.615	6.794	9.581	1.00	0.00	C
ATOM	72	HA	ASP	6	11.096	7.521	10.207	1.00	0.00	H
ATOM	73	CB	ASP	6	11.908	7.595	8.303	1.00	0.00	C
ATOM	74	HB2	ASP	6	12.623	8.405	8.452	1.00	0.00	H
ATOM	75	HB3	ASP	6	10.954	8.072	8.080	1.00	0.00	H
ATOM	76	CG	ASP	6	12.400	6.483	7.214	1.00	0.00	C
ATOM	77	OD1	ASP	6	13.216	5.648	7.582	1.00	0.00	O
ATOM	78	OD2	ASP	6	11.990	6.469	6.051	1.00	0.00	O
ATOM	79	C	ASP	6	10.616	5.584	9.308	1.00	0.00	C
ATOM	80	O	ASP	6	9.588	5.663	8.626	1.00	0.00	O
ATOM	81	N	ARG	7	11.028	4.404	9.712	1.00	0.00	N
ATOM	82	H	ARG	7	11.909	4.395	10.206	1.00	0.00	H
ATOM	83	CA	ARG	7	10.357	3.077	9.582	1.00	0.00	C
ATOM	84	HA	ARG	7	10.482	2.673	8.578	1.00	0.00	H
ATOM	85	CB	ARG	7	10.895	2.150	10.761	1.00	0.00	C
ATOM	86	HB2	ARG	7	10.277	1.307	11.068	1.00	0.00	H
ATOM	87	HB3	ARG	7	10.945	2.887	11.563	1.00	0.00	H
ATOM	88	CG	ARG	7	12.307	1.595	10.462	1.00	0.00	C
ATOM	89	HG2	ARG	7	13.082	2.361	10.508	1.00	0.00	H
ATOM	90	HG3	ARG	7	12.148	1.195	9.460	1.00	0.00	H
ATOM	91	CD	ARG	7	12.739	0.518	11.377	1.00	0.00	C
ATOM	92	HD2	ARG	7	11.976	-0.247	11.517	1.00	0.00	H
ATOM	93	HD3	ARG	7	12.897	1.003	12.340	1.00	0.00	H
ATOM	94	NE	ARG	7	13.969	-0.115	11.129	1.00	0.00	N
ATOM	95	HE	ARG	7	14.543	0.250	10.382	1.00	0.00	H
ATOM	96	CZ	ARG	7	14.296	-1.281	11.641	1.00	0.00	C
ATOM	97	NH1	ARG	7	13.452	-1.971	12.387	1.00	0.00	N
ATOM	98	HH11	ARG	7	12.554	-1.667	12.734	1.00	0.00	H
ATOM	99	HH12	ARG	7	13.869	-2.775	12.834	1.00	0.00	H
ATOM	100	NH2	ARG	7	15.480	-1.726	11.371	1.00	0.00	N
ATOM	101	HH21	ARG	7	16.071	-1.255	10.700	1.00	0.00	H

ATOM	102	HH22	ARG	7	15.741	-2.674	11.604	1.00	0.00	H
ATOM	103	C	ARG	7	8.826	3.217	9.722	1.00	0.00	C
ATOM	104	O	ARG	7	8.012	2.616	8.927	1.00	0.00	O
ATOM	105	N	TYR	8	8.386	3.860	10.829	1.00	0.00	N
ATOM	106	H	TYR	8	9.117	4.271	11.393	1.00	0.00	H
ATOM	107	CA	TYR	8	6.951	4.100	11.070	1.00	0.00	C
ATOM	108	HA	TYR	8	6.430	3.173	10.831	1.00	0.00	H
ATOM	109	CB	TYR	8	6.724	4.535	12.532	1.00	0.00	C
ATOM	110	HB2	TYR	8	5.714	4.766	12.870	1.00	0.00	H
ATOM	111	HB3	TYR	8	7.385	5.397	12.440	1.00	0.00	H
ATOM	112	CG	TYR	8	7.179	3.469	13.520	1.00	0.00	C
ATOM	113	CD1	TYR	8	6.230	2.473	13.950	1.00	0.00	C
ATOM	114	HD1	TYR	8	5.198	2.477	13.632	1.00	0.00	H
ATOM	115	CE1	TYR	8	6.575	1.467	14.801	1.00	0.00	C
ATOM	116	HE1	TYR	8	5.825	0.755	15.112	1.00	0.00	H
ATOM	117	CZ	TYR	8	7.888	1.516	15.398	1.00	0.00	C
ATOM	118	OH	TYR	8	8.220	0.628	16.353	1.00	0.00	O
ATOM	119	HH	TYR	8	7.595	-0.058	16.599	1.00	0.00	H
ATOM	120	CE2	TYR	8	8.816	2.440	14.889	1.00	0.00	C
ATOM	121	HE2	TYR	8	9.775	2.544	15.375	1.00	0.00	H
ATOM	122	CD2	TYR	8	8.442	3.461	14.032	1.00	0.00	C
ATOM	123	HD2	TYR	8	9.126	4.253	13.764	1.00	0.00	H
ATOM	124	C	TYR	8	6.128	5.025	10.186	1.00	0.00	C
ATOM	125	O	TYR	8	4.913	4.837	10.175	1.00	0.00	O
ATOM	126	N	TYR	9	6.781	5.795	9.275	1.00	0.00	N
ATOM	127	H	TYR	9	7.766	5.876	9.482	1.00	0.00	H
ATOM	128	CA	TYR	9	6.205	6.775	8.342	1.00	0.00	C
ATOM	129	HA	TYR	9	5.124	6.676	8.437	1.00	0.00	H
ATOM	130	CB	TYR	9	6.627	8.205	8.673	1.00	0.00	C
ATOM	131	HB2	TYR	9	6.002	8.859	8.065	1.00	0.00	H
ATOM	132	HB3	TYR	9	7.682	8.297	8.416	1.00	0.00	H
ATOM	133	CG	TYR	9	6.439	8.690	10.124	1.00	0.00	C
ATOM	134	CD1	TYR	9	5.190	9.115	10.614	1.00	0.00	C
ATOM	135	HD1	TYR	9	4.399	9.286	9.899	1.00	0.00	H
ATOM	136	CE1	TYR	9	4.990	9.419	11.988	1.00	0.00	C
ATOM	137	HE1	TYR	9	4.036	9.740	12.380	1.00	0.00	H
ATOM	138	CZ	TYR	9	6.085	9.395	12.867	1.00	0.00	C
ATOM	139	OH	TYR	9	5.951	9.930	14.101	1.00	0.00	O
ATOM	140	HH	TYR	9	5.062	10.200	14.346	1.00	0.00	H
ATOM	141	CE2	TYR	9	7.347	9.032	12.352	1.00	0.00	C
ATOM	142	HE2	TYR	9	8.218	8.894	12.976	1.00	0.00	H
ATOM	143	CD2	TYR	9	7.512	8.570	11.037	1.00	0.00	C
ATOM	144	HD2	TYR	9	8.497	8.227	10.754	1.00	0.00	H
ATOM	145	C	TYR	9	6.534	6.436	6.886	1.00	0.00	C
ATOM	146	O	TYR	9	6.321	7.305	5.983	1.00	0.00	O
ATOM	147	N	ARG	10	7.145	5.300	6.642	1.00	0.00	N
ATOM	148	H	ARG	10	7.313	4.578	7.329	1.00	0.00	H
ATOM	149	CA	ARG	10	7.704	4.916	5.317	1.00	0.00	C
ATOM	150	HA	ARG	10	8.268	5.783	4.974	1.00	0.00	H
ATOM	151	CB	ARG	10	8.560	3.625	5.498	1.00	0.00	C
ATOM	152	HB2	ARG	10	7.957	2.730	5.343	1.00	0.00	H
ATOM	153	HB3	ARG	10	8.951	3.657	6.515	1.00	0.00	H
ATOM	154	CG	ARG	10	9.700	3.567	4.431	1.00	0.00	C
ATOM	155	HG2	ARG	10	10.050	4.569	4.183	1.00	0.00	H
ATOM	156	HG3	ARG	10	9.159	3.139	3.587	1.00	0.00	H
ATOM	157	CD	ARG	10	10.885	2.637	4.744	1.00	0.00	C
ATOM	158	HD2	ARG	10	11.441	2.448	3.826	1.00	0.00	H
ATOM	159	HD3	ARG	10	10.476	1.652	4.969	1.00	0.00	H
ATOM	160	NE	ARG	10	11.736	3.018	5.866	1.00	0.00	N
ATOM	161	HE	ARG	10	11.769	3.970	6.203	1.00	0.00	H
ATOM	162	CZ	ARG	10	12.734	2.386	6.417	1.00	0.00	C
ATOM	163	NH1	ARG	10	13.309	3.001	7.420	1.00	0.00	N
ATOM	164	HH11	ARG	10	13.750	2.353	8.057	1.00	0.00	H

ATOM	165	HH12	ARG	10	13.235	3.994	7.586	1.00	0.00	H
ATOM	166	NH2	ARG	10	13.064	1.144	6.214	1.00	0.00	N
ATOM	167	HH21	ARG	10	12.752	0.634	5.400	1.00	0.00	H
ATOM	168	HH22	ARG	10	13.821	0.751	6.755	1.00	0.00	H
ATOM	169	C	ARG	10	6.608	4.694	4.241	1.00	0.00	C
ATOM	170	O	ARG	10	6.861	4.897	3.046	1.00	0.00	O
ATOM	171	N	GLU	11	5.402	4.270	4.617	1.00	0.00	N
ATOM	172	H	GLU	11	5.198	4.201	5.604	1.00	0.00	H
ATOM	173	CA	GLU	11	4.283	4.041	3.667	1.00	0.00	C
ATOM	174	HA	GLU	11	4.488	3.353	2.847	1.00	0.00	H
ATOM	175	CB	GLU	11	3.048	3.439	4.380	1.00	0.00	C
ATOM	176	HB2	GLU	11	2.187	3.477	3.713	1.00	0.00	H
ATOM	177	HB3	GLU	11	2.922	4.209	5.142	1.00	0.00	H
ATOM	178	CG	GLU	11	2.999	2.057	5.010	1.00	0.00	C
ATOM	179	HG2	GLU	11	2.016	1.766	5.379	1.00	0.00	H
ATOM	180	HG3	GLU	11	3.719	2.006	5.827	1.00	0.00	H
ATOM	181	CD	GLU	11	3.419	1.035	3.907	1.00	0.00	C
ATOM	182	OE1	GLU	11	2.556	0.479	3.221	1.00	0.00	O
ATOM	183	OE2	GLU	11	4.622	0.839	3.705	1.00	0.00	O
ATOM	184	C	GLU	11	3.873	5.279	2.879	1.00	0.00	C
ATOM	185	O	GLU	11	3.690	5.198	1.671	1.00	0.00	O
ATOM	186	N	ASN	12	3.857	6.398	3.634	1.00	0.00	N
ATOM	187	H	ASN	12	4.307	6.305	4.533	1.00	0.00	H
ATOM	188	CA	ASN	12	3.415	7.766	3.317	1.00	0.00	C
ATOM	189	HA	ASN	12	2.494	7.601	2.759	1.00	0.00	H
ATOM	190	CB	ASN	12	3.175	8.524	4.616	1.00	0.00	C
ATOM	191	HB2	ASN	12	4.014	9.073	5.044	1.00	0.00	H
ATOM	192	HB3	ASN	12	2.950	7.773	5.373	1.00	0.00	H
ATOM	193	CG	ASN	12	2.003	9.354	4.459	1.00	0.00	C
ATOM	194	OD1	ASN	12	0.951	8.916	3.965	1.00	0.00	O
ATOM	195	ND2	ASN	12	2.041	10.589	4.908	1.00	0.00	N
ATOM	196	HD21	ASN	12	1.150	11.065	4.897	1.00	0.00	H
ATOM	197	HD22	ASN	12	2.800	10.880	5.507	1.00	0.00	H
ATOM	198	C	ASN	12	4.519	8.490	2.498	1.00	0.00	C
ATOM	199	O	ASN	12	4.209	9.233	1.567	1.00	0.00	O
ATOM	200	N	MET	13	5.770	8.230	2.925	1.00	0.00	N
ATOM	201	H	MET	13	6.003	7.529	3.614	1.00	0.00	H
ATOM	202	CA	MET	13	6.960	8.857	2.350	1.00	0.00	C
ATOM	203	HA	MET	13	6.786	9.917	2.168	1.00	0.00	H
ATOM	204	CB	MET	13	8.218	8.815	3.294	1.00	0.00	C
ATOM	205	HB2	MET	13	9.134	8.988	2.730	1.00	0.00	H
ATOM	206	HB3	MET	13	8.298	7.785	3.643	1.00	0.00	H
ATOM	207	CG	MET	13	8.197	9.647	4.553	1.00	0.00	C
ATOM	208	HG2	MET	13	7.712	9.151	5.394	1.00	0.00	H
ATOM	209	HG3	MET	13	7.653	10.517	4.186	1.00	0.00	H
ATOM	210	SD	MET	13	9.769	10.103	5.216	1.00	0.00	S
ATOM	211	CE	MET	13	9.176	11.402	6.347	1.00	0.00	C
ATOM	212	HE1	MET	13	10.012	11.980	6.740	1.00	0.00	H
ATOM	213	HE2	MET	13	8.518	12.116	5.852	1.00	0.00	H
ATOM	214	HE3	MET	13	8.601	10.874	7.109	1.00	0.00	H
ATOM	215	C	MET	13	7.433	8.195	1.034	1.00	0.00	C
ATOM	216	O	MET	13	7.885	8.909	0.179	1.00	0.00	O
ATOM	217	N	TYR	14	7.226	6.877	0.868	1.00	0.00	N
ATOM	218	H	TYR	14	6.915	6.367	1.682	1.00	0.00	H
ATOM	219	CA	TYR	14	7.833	6.066	-0.240	1.00	0.00	C
ATOM	220	HA	TYR	14	7.572	5.035	-0.002	1.00	0.00	H
ATOM	221	CB	TYR	14	7.093	6.266	-1.601	1.00	0.00	C
ATOM	222	HB2	TYR	14	7.527	5.573	-2.322	1.00	0.00	H
ATOM	223	HB3	TYR	14	7.492	7.236	-1.897	1.00	0.00	H
ATOM	224	CG	TYR	14	5.592	6.240	-1.567	1.00	0.00	C
ATOM	225	CD1	TYR	14	4.871	7.381	-1.768	1.00	0.00	C
ATOM	226	HD1	TYR	14	5.420	8.276	-2.022	1.00	0.00	H
ATOM	227	CE1	TYR	14	3.466	7.353	-1.675	1.00	0.00	C

```
ATOM    228  HE1 TYR    14       2.879   8.222  -1.936  1.00  0.00           H
ATOM    229  CZ  TYR    14       2.781   6.114  -1.375  1.00  0.00           C
ATOM    230  OH  TYR    14       1.414   5.986  -1.339  1.00  0.00           O
ATOM    231  HH  TYR    14       1.078   5.100  -1.186  1.00  0.00           H
ATOM    232  CE2 TYR    14       3.516   4.934  -1.176  1.00  0.00           C
ATOM    233  HE2 TYR    14       3.092   3.990  -0.867  1.00  0.00           H
ATOM    234  CD2 TYR    14       4.936   5.044  -1.271  1.00  0.00           C
ATOM    235  HD2 TYR    14       5.537   4.159  -1.125  1.00  0.00           H
ATOM    236  C   TYR    14       9.358   6.160  -0.405  1.00  0.00           C
ATOM    237  O   TYR    14       9.849   6.536  -1.479  1.00  0.00           O
ATOM    238  N   ARG    15      10.076   5.897   0.729  1.00  0.00           N
ATOM    239  H   ARG    15       9.583   5.732   1.595  1.00  0.00           H
ATOM    240  CA  ARG    15      11.526   5.863   0.745  1.00  0.00           C
ATOM    241  HA  ARG    15      11.879   6.526  -0.045  1.00  0.00           H
ATOM    242  CB  ARG    15      12.062   6.590   1.996  1.00  0.00           C
ATOM    243  HB2 ARG    15      11.869   5.982   2.880  1.00  0.00           H
ATOM    244  HB3 ARG    15      11.348   7.412   1.956  1.00  0.00           H
ATOM    245  CG  ARG    15      13.443   7.125   1.992  1.00  0.00           C
ATOM    246  HG2 ARG    15      13.578   7.802   1.149  1.00  0.00           H
ATOM    247  HG3 ARG    15      14.088   6.251   1.897  1.00  0.00           H
ATOM    248  CD  ARG    15      13.867   7.854   3.243  1.00  0.00           C
ATOM    249  HD2 ARG    15      13.059   8.444   3.675  1.00  0.00           H
ATOM    250  HD3 ARG    15      14.696   8.474   2.904  1.00  0.00           H
ATOM    251  NE  ARG    15      14.123   6.905   4.353  1.00  0.00           N
ATOM    252  HE  ARG    15      13.325   6.774   4.959  1.00  0.00           H
ATOM    253  CZ  ARG    15      15.115   6.040   4.452  1.00  0.00           C
ATOM    254  NH1 ARG    15      14.949   5.190   5.398  1.00  0.00           N
ATOM    255 HH11 ARG    15      15.687   4.506   5.480  1.00  0.00           H
ATOM    256 HH12 ARG    15      14.312   5.359   6.164  1.00  0.00           H
ATOM    257  NH2 ARG    15      16.056   5.931   3.601  1.00  0.00           N
ATOM    258 HH21 ARG    15      16.629   5.108   3.726  1.00  0.00           H
ATOM    259 HH22 ARG    15      16.230   6.740   3.023  1.00  0.00           H
ATOM    260  C   ARG    15      12.207   4.438   0.470  1.00  0.00           C
ATOM    261  O   ARG    15      13.396   4.334   0.677  1.00  0.00           O
ATOM    262  N   TYR    16      11.422   3.417   0.134  1.00  0.00           N
ATOM    263  H   TYR    16      10.462   3.615  -0.111  1.00  0.00           H
ATOM    264  CA  TYR    16      11.795   2.056  -0.138  1.00  0.00           C
ATOM    265  HA  TYR    16      12.436   1.663   0.651  1.00  0.00           H
ATOM    266  CB  TYR    16      10.538   1.171  -0.200  1.00  0.00           C
ATOM    267  HB2 TYR    16      10.928   0.213  -0.543  1.00  0.00           H
ATOM    268  HB3 TYR    16       9.924   1.619  -0.981  1.00  0.00           H
ATOM    269  CG  TYR    16       9.605   0.928   0.968  1.00  0.00           C
ATOM    270  CD1 TYR    16      10.071   0.137   2.026  1.00  0.00           C
ATOM    271  HD1 TYR    16      11.054  -0.297   1.917  1.00  0.00           H
ATOM    272  CE1 TYR    16       9.242  -0.009   3.178  1.00  0.00           C
ATOM    273  HE1 TYR    16       9.523  -0.609   4.031  1.00  0.00           H
ATOM    274  CZ  TYR    16       7.927   0.495   3.191  1.00  0.00           C
ATOM    275  OH  TYR    16       7.178   0.360   4.320  1.00  0.00           O
ATOM    276  HH  TYR    16       6.268   0.591   4.118  1.00  0.00           H
ATOM    277  CE2 TYR    16       7.508   1.275   2.118  1.00  0.00           C
ATOM    278  HE2 TYR    16       6.500   1.652   2.202  1.00  0.00           H
ATOM    279  CD2 TYR    16       8.268   1.474   0.971  1.00  0.00           C
ATOM    280  HD2 TYR    16       7.918   1.992   0.091  1.00  0.00           H
ATOM    281  C   TYR    16      12.590   1.951  -1.482  1.00  0.00           C
ATOM    282  O   TYR    16      12.538   2.830  -2.363  1.00  0.00           O
ATOM    283  N   NME    17      13.331   0.809  -1.608  1.00  0.00           N
ATOM    284  H   NME    17      13.278   0.069  -0.923  1.00  0.00           H
ATOM    285  CH3 NME    17      14.059   0.617  -2.873  1.00  0.00           C
ATOM    286 HH31 NME    17      14.565   1.528  -3.193  1.00  0.00           H
ATOM    287 HH32 NME    17      14.767  -0.189  -2.676  1.00  0.00           H
ATOM    288 HH33 NME    17      13.342   0.352  -3.650  1.00  0.00           H
TER     289      NME    17
END
```

Cluster 10:

```
ATOM      1 HH31 ACE     1      11.872  -2.212   8.846  1.00  0.00           H
ATOM      2  CH3 ACE     1      11.424  -1.833   7.929  1.00  0.00           C
ATOM      3 HH32 ACE     1      10.828  -2.631   7.486  1.00  0.00           H
ATOM      4 HH33 ACE     1      12.157  -1.514   7.187  1.00  0.00           H
ATOM      5  C   ACE     1      10.469  -0.721   8.283  1.00  0.00           C
ATOM      6  O   ACE     1      10.641   0.464   8.000  1.00  0.00           O
ATOM      7  N   ASN     2       9.460  -1.165   8.969  1.00  0.00           N
ATOM      8  H   ASN     2       9.382  -2.147   9.191  1.00  0.00           H
ATOM      9  CA  ASN     2       8.415  -0.292   9.534  1.00  0.00           C
ATOM     10  HA  ASN     2       8.281   0.461   8.758  1.00  0.00           H
ATOM     11  CB  ASN     2       7.193  -1.125   9.840  1.00  0.00           C
ATOM     12  HB2 ASN     2       7.266  -1.412  10.889  1.00  0.00           H
ATOM     13  HB3 ASN     2       7.118  -1.966   9.152  1.00  0.00           H
ATOM     14  CG  ASN     2       5.857  -0.416   9.616  1.00  0.00           C
ATOM     15  OD1 ASN     2       5.722   0.757   9.308  1.00  0.00           O
ATOM     16  ND2 ASN     2       4.772  -1.052   9.770  1.00  0.00           N
ATOM     17 HD21 ASN     2       3.908  -0.569   9.970  1.00  0.00           H
ATOM     18 HD22 ASN     2       4.803  -2.011  10.084  1.00  0.00           H
ATOM     19  C   ASN     2       8.895   0.545  10.750  1.00  0.00           C
ATOM     20  O   ASN     2       8.027   0.987  11.419  1.00  0.00           O
ATOM     21  N   ASP     3      10.190   0.768  11.004  1.00  0.00           N
ATOM     22  H   ASP     3      10.860   0.408  10.341  1.00  0.00           H
ATOM     23  CA  ASP     3      10.846   1.429  12.089  1.00  0.00           C
ATOM     24  HA  ASP     3      10.176   2.114  12.610  1.00  0.00           H
ATOM     25  CB  ASP     3      11.196   0.456  13.159  1.00  0.00           C
ATOM     26  HB2 ASP     3      11.486   1.119  13.974  1.00  0.00           H
ATOM     27  HB3 ASP     3      12.047  -0.142  12.829  1.00  0.00           H
ATOM     28  CG  ASP     3      10.106  -0.505  13.609  1.00  0.00           C
ATOM     29  OD1 ASP     3      10.168  -1.666  13.097  1.00  0.00           O
ATOM     30  OD2 ASP     3       9.309  -0.231  14.581  1.00  0.00           O
ATOM     31  C   ASP     3      12.113   2.124  11.566  1.00  0.00           C
ATOM     32  O   ASP     3      12.927   2.578  12.379  1.00  0.00           O
ATOM     33  N   TYR     4      12.339   2.209  10.185  1.00  0.00           N
ATOM     34  H   TYR     4      11.628   1.927   9.525  1.00  0.00           H
ATOM     35  CA  TYR     4      13.511   2.954   9.693  1.00  0.00           C
ATOM     36  HA  TYR     4      14.416   2.685  10.237  1.00  0.00           H
ATOM     37  CB  TYR     4      13.824   2.612   8.238  1.00  0.00           C
ATOM     38  HB2 TYR     4      14.461   3.356   7.758  1.00  0.00           H
ATOM     39  HB3 TYR     4      12.851   2.488   7.763  1.00  0.00           H
ATOM     40  CG  TYR     4      14.600   1.332   8.021  1.00  0.00           C
ATOM     41  CD1 TYR     4      14.159   0.407   7.123  1.00  0.00           C
ATOM     42  HD1 TYR     4      13.187   0.559   6.674  1.00  0.00           H
ATOM     43  CE1 TYR     4      15.003  -0.674   6.753  1.00  0.00           C
ATOM     44  HE1 TYR     4      14.675  -1.302   5.937  1.00  0.00           H
ATOM     45  CZ  TYR     4      16.202  -0.883   7.439  1.00  0.00           C
ATOM     46  OH  TYR     4      17.059  -1.931   7.035  1.00  0.00           O
ATOM     47  HH  TYR     4      16.758  -2.396   6.251  1.00  0.00           H
ATOM     48  CE2 TYR     4      16.634   0.057   8.389  1.00  0.00           C
ATOM     49  HE2 TYR     4      17.583  -0.135   8.866  1.00  0.00           H
ATOM     50  CD2 TYR     4      15.813   1.166   8.735  1.00  0.00           C
ATOM     51  HD2 TYR     4      16.178   1.878   9.460  1.00  0.00           H
ATOM     52  C   TYR     4      13.339   4.496   9.841  1.00  0.00           C
ATOM     53  O   TYR     4      12.524   5.123   9.205  1.00  0.00           O
ATOM     54  N   GLU     5      14.266   5.063  10.617  1.00  0.00           N
ATOM     55  H   GLU     5      14.915   4.557  11.202  1.00  0.00           H
ATOM     56  CA  GLU     5      14.348   6.501  10.615  1.00  0.00           C
ATOM     57  HA  GLU     5      13.331   6.889  10.677  1.00  0.00           H
ATOM     58  CB  GLU     5      15.154   6.977  11.851  1.00  0.00           C
```

ATOM	59	HB2	GLU	5	15.259	8.061	11.832	1.00	0.00	H
ATOM	60	HB3	GLU	5	16.091	6.441	11.695	1.00	0.00	H
ATOM	61	CG	GLU	5	14.478	6.514	13.186	1.00	0.00	C
ATOM	62	HG2	GLU	5	14.637	5.451	13.369	1.00	0.00	H
ATOM	63	HG3	GLU	5	13.405	6.651	13.050	1.00	0.00	H
ATOM	64	CD	GLU	5	15.100	7.375	14.235	1.00	0.00	C
ATOM	65	OE1	GLU	5	16.309	7.314	14.457	1.00	0.00	O
ATOM	66	OE2	GLU	5	14.392	8.174	14.919	1.00	0.00	O
ATOM	67	C	GLU	5	15.082	7.203	9.365	1.00	0.00	C
ATOM	68	O	GLU	5	15.795	8.200	9.504	1.00	0.00	O
ATOM	69	N	ASP	6	14.949	6.569	8.182	1.00	0.00	N
ATOM	70	H	ASP	6	14.341	5.762	8.191	1.00	0.00	H
ATOM	71	CA	ASP	6	15.574	6.936	6.932	1.00	0.00	C
ATOM	72	HA	ASP	6	16.646	6.946	7.131	1.00	0.00	H
ATOM	73	CB	ASP	6	15.455	5.796	5.854	1.00	0.00	C
ATOM	74	HB2	ASP	6	14.416	5.484	5.749	1.00	0.00	H
ATOM	75	HB3	ASP	6	16.087	5.019	6.282	1.00	0.00	H
ATOM	76	CG	ASP	6	16.063	6.058	4.433	1.00	0.00	C
ATOM	77	OD1	ASP	6	17.177	5.590	4.101	1.00	0.00	O
ATOM	78	OD2	ASP	6	15.256	6.595	3.611	1.00	0.00	O
ATOM	79	C	ASP	6	15.178	8.304	6.446	1.00	0.00	C
ATOM	80	O	ASP	6	14.055	8.797	6.467	1.00	0.00	O
ATOM	81	N	ARG	7	16.129	9.011	5.797	1.00	0.00	N
ATOM	82	H	ARG	7	17.045	8.609	5.940	1.00	0.00	H
ATOM	83	CA	ARG	7	15.956	10.340	5.218	1.00	0.00	C
ATOM	84	HA	ARG	7	15.606	11.020	5.995	1.00	0.00	H
ATOM	85	CB	ARG	7	17.267	10.934	4.836	1.00	0.00	C
ATOM	86	HB2	ARG	7	17.923	11.030	5.701	1.00	0.00	H
ATOM	87	HB3	ARG	7	16.933	11.916	4.498	1.00	0.00	H
ATOM	88	CG	ARG	7	18.115	10.375	3.673	1.00	0.00	C
ATOM	89	HG2	ARG	7	18.904	11.103	3.485	1.00	0.00	H
ATOM	90	HG3	ARG	7	17.471	10.157	2.821	1.00	0.00	H
ATOM	91	CD	ARG	7	18.996	9.183	3.937	1.00	0.00	C
ATOM	92	HD2	ARG	7	18.306	8.350	4.067	1.00	0.00	H
ATOM	93	HD3	ARG	7	19.457	9.473	4.880	1.00	0.00	H
ATOM	94	NE	ARG	7	20.047	8.986	2.995	1.00	0.00	N
ATOM	95	HE	ARG	7	20.186	9.747	2.345	1.00	0.00	H
ATOM	96	CZ	ARG	7	21.029	8.093	3.111	1.00	0.00	C
ATOM	97	NH1	ARG	7	21.940	7.977	2.206	1.00	0.00	N
ATOM	98	HH11	ARG	7	21.898	8.521	1.356	1.00	0.00	H
ATOM	99	HH12	ARG	7	22.586	7.213	2.347	1.00	0.00	H
ATOM	100	NH2	ARG	7	21.043	7.242	4.095	1.00	0.00	N
ATOM	101	HH21	ARG	7	20.204	7.203	4.655	1.00	0.00	H
ATOM	102	HH22	ARG	7	21.785	6.573	4.247	1.00	0.00	H
ATOM	103	C	ARG	7	14.842	10.541	4.183	1.00	0.00	C
ATOM	104	O	ARG	7	14.317	11.618	4.051	1.00	0.00	O
ATOM	105	N	TYR	8	14.622	9.493	3.368	1.00	0.00	N
ATOM	106	H	TYR	8	15.209	8.674	3.412	1.00	0.00	H
ATOM	107	CA	TYR	8	13.514	9.473	2.410	1.00	0.00	C
ATOM	108	HA	TYR	8	12.967	10.415	2.399	1.00	0.00	H
ATOM	109	CB	TYR	8	14.135	9.246	1.083	1.00	0.00	C
ATOM	110	HB2	TYR	8	13.449	9.246	0.235	1.00	0.00	H
ATOM	111	HB3	TYR	8	14.574	8.253	1.175	1.00	0.00	H
ATOM	112	CG	TYR	8	15.184	10.305	0.729	1.00	0.00	C
ATOM	113	CD1	TYR	8	16.559	9.841	0.557	1.00	0.00	C
ATOM	114	HD1	TYR	8	16.894	8.854	0.843	1.00	0.00	H
ATOM	115	CE1	TYR	8	17.644	10.677	0.235	1.00	0.00	C
ATOM	116	HE1	TYR	8	18.606	10.188	0.264	1.00	0.00	H
ATOM	117	CZ	TYR	8	17.358	12.047	0.087	1.00	0.00	C
ATOM	118	OH	TYR	8	18.420	12.883	-0.061	1.00	0.00	O
ATOM	119	HH	TYR	8	19.268	12.441	-0.153	1.00	0.00	H
ATOM	120	CE2	TYR	8	16.083	12.562	0.323	1.00	0.00	C
ATOM	121	HE2	TYR	8	15.920	13.630	0.315	1.00	0.00	H

ATOM	122	CD2	TYR	8	14.950	11.647	0.614	1.00	0.00	C
ATOM	123	HD2	TYR	8	13.990	12.139	0.664	1.00	0.00	H
ATOM	124	C	TYR	8	12.433	8.366	2.710	1.00	0.00	C
ATOM	125	O	TYR	8	11.853	7.727	1.929	1.00	0.00	O
ATOM	126	N	TYR	9	12.225	8.098	4.040	1.00	0.00	N
ATOM	127	H	TYR	9	12.961	8.515	4.592	1.00	0.00	H
ATOM	128	CA	TYR	9	11.167	7.358	4.694	1.00	0.00	C
ATOM	129	HA	TYR	9	11.401	7.377	5.759	1.00	0.00	H
ATOM	130	CB	TYR	9	9.857	8.088	4.529	1.00	0.00	C
ATOM	131	HB2	TYR	9	9.112	7.547	5.112	1.00	0.00	H
ATOM	132	HB3	TYR	9	9.483	8.051	3.505	1.00	0.00	H
ATOM	133	CG	TYR	9	9.820	9.515	5.020	1.00	0.00	C
ATOM	134	CD1	TYR	9	10.318	9.727	6.306	1.00	0.00	C
ATOM	135	HD1	TYR	9	10.628	8.912	6.944	1.00	0.00	H
ATOM	136	CE1	TYR	9	10.575	11.031	6.720	1.00	0.00	C
ATOM	137	HE1	TYR	9	10.839	11.340	7.721	1.00	0.00	H
ATOM	138	CZ	TYR	9	10.284	12.087	5.834	1.00	0.00	C
ATOM	139	OH	TYR	9	10.334	13.328	6.270	1.00	0.00	O
ATOM	140	HH	TYR	9	9.924	13.863	5.587	1.00	0.00	H
ATOM	141	CE2	TYR	9	9.719	11.861	4.544	1.00	0.00	C
ATOM	142	HE2	TYR	9	9.446	12.661	3.872	1.00	0.00	H
ATOM	143	CD2	TYR	9	9.455	10.550	4.092	1.00	0.00	C
ATOM	144	HD2	TYR	9	9.056	10.368	3.105	1.00	0.00	H
ATOM	145	C	TYR	9	11.154	5.875	4.210	1.00	0.00	C
ATOM	146	O	TYR	9	10.105	5.209	4.313	1.00	0.00	O
ATOM	147	N	ARG	10	12.259	5.322	3.651	1.00	0.00	N
ATOM	148	H	ARG	10	13.109	5.864	3.722	1.00	0.00	H
ATOM	149	CA	ARG	10	12.274	4.047	2.885	1.00	0.00	C
ATOM	150	HA	ARG	10	11.484	4.130	2.139	1.00	0.00	H
ATOM	151	CB	ARG	10	13.557	3.869	2.077	1.00	0.00	C
ATOM	152	HB2	ARG	10	13.505	2.942	1.507	1.00	0.00	H
ATOM	153	HB3	ARG	10	14.315	3.859	2.860	1.00	0.00	H
ATOM	154	CG	ARG	10	13.863	4.903	1.010	1.00	0.00	C
ATOM	155	HG2	ARG	10	13.876	5.907	1.433	1.00	0.00	H
ATOM	156	HG3	ARG	10	13.020	4.732	0.339	1.00	0.00	H
ATOM	157	CD	ARG	10	15.246	4.813	0.331	1.00	0.00	C
ATOM	158	HD2	ARG	10	15.454	5.663	-0.318	1.00	0.00	H
ATOM	159	HD3	ARG	10	15.010	3.944	-0.283	1.00	0.00	H
ATOM	160	NE	ARG	10	16.383	4.670	1.199	1.00	0.00	N
ATOM	161	HE	ARG	10	16.447	5.311	1.976	1.00	0.00	H
ATOM	162	CZ	ARG	10	17.502	3.986	0.999	1.00	0.00	C
ATOM	163	NH1	ARG	10	18.373	4.160	1.930	1.00	0.00	N
ATOM	164	HH11	ARG	10	18.098	4.828	2.637	1.00	0.00	H
ATOM	165	HH12	ARG	10	19.293	3.743	1.957	1.00	0.00	H
ATOM	166	NH2	ARG	10	17.753	3.110	0.064	1.00	0.00	N
ATOM	167	HH21	ARG	10	17.176	3.058	-0.763	1.00	0.00	H
ATOM	168	HH22	ARG	10	18.563	2.510	0.002	1.00	0.00	H
ATOM	169	C	ARG	10	11.818	2.806	3.664	1.00	0.00	C
ATOM	170	O	ARG	10	12.446	2.352	4.598	1.00	0.00	O
ATOM	171	N	GLU	11	10.718	2.213	3.222	1.00	0.00	N
ATOM	172	H	GLU	11	10.304	2.733	2.461	1.00	0.00	H
ATOM	173	CA	GLU	11	9.900	1.179	3.835	1.00	0.00	C
ATOM	174	HA	GLU	11	9.133	0.968	3.089	1.00	0.00	H
ATOM	175	CB	GLU	11	10.548	-0.232	3.922	1.00	0.00	C
ATOM	176	HB2	GLU	11	9.859	-0.939	4.383	1.00	0.00	H
ATOM	177	HB3	GLU	11	11.363	-0.054	4.624	1.00	0.00	H
ATOM	178	CG	GLU	11	11.026	-0.991	2.661	1.00	0.00	C
ATOM	179	HG2	GLU	11	11.590	-0.318	2.016	1.00	0.00	H
ATOM	180	HG3	GLU	11	10.061	-1.270	2.239	1.00	0.00	H
ATOM	181	CD	GLU	11	11.868	-2.204	2.997	1.00	0.00	C
ATOM	182	OE1	GLU	11	13.050	-2.269	2.676	1.00	0.00	O
ATOM	183	OE2	GLU	11	11.289	-3.205	3.582	1.00	0.00	O
ATOM	184	C	GLU	11	9.084	1.644	5.089	1.00	0.00	C

ATOM	185	O	GLU	11	8.103	0.975	5.483	1.00	0.00	O
ATOM	186	N	ASN	12	9.470	2.748	5.741	1.00	0.00	N
ATOM	187	H	ASN	12	10.241	3.316	5.419	1.00	0.00	H
ATOM	188	CA	ASN	12	8.913	3.307	7.024	1.00	0.00	C
ATOM	189	HA	ASN	12	8.999	2.465	7.710	1.00	0.00	H
ATOM	190	CB	ASN	12	9.769	4.416	7.585	1.00	0.00	C
ATOM	191	HB2	ASN	12	9.800	5.298	6.945	1.00	0.00	H
ATOM	192	HB3	ASN	12	10.751	3.981	7.768	1.00	0.00	H
ATOM	193	CG	ASN	12	9.373	4.902	8.994	1.00	0.00	C
ATOM	194	OD1	ASN	12	9.030	4.107	9.855	1.00	0.00	O
ATOM	195	ND2	ASN	12	9.279	6.222	9.206	1.00	0.00	N
ATOM	196	HD21	ASN	12	8.898	6.317	10.136	1.00	0.00	H
ATOM	197	HD22	ASN	12	9.561	6.888	8.501	1.00	0.00	H
ATOM	198	C	ASN	12	7.542	3.942	6.935	1.00	0.00	C
ATOM	199	O	ASN	12	6.757	3.617	7.785	1.00	0.00	O
ATOM	200	N	MET	13	7.175	4.645	5.876	1.00	0.00	N
ATOM	201	H	MET	13	7.878	4.807	5.169	1.00	0.00	H
ATOM	202	CA	MET	13	5.843	5.149	5.605	1.00	0.00	C
ATOM	203	HA	MET	13	5.223	4.827	6.441	1.00	0.00	H
ATOM	204	CB	MET	13	5.874	6.682	5.693	1.00	0.00	C
ATOM	205	HB2	MET	13	4.864	7.061	5.542	1.00	0.00	H
ATOM	206	HB3	MET	13	6.516	6.966	4.860	1.00	0.00	H
ATOM	207	CG	MET	13	6.397	7.321	7.008	1.00	0.00	C
ATOM	208	HG2	MET	13	7.279	6.795	7.376	1.00	0.00	H
ATOM	209	HG3	MET	13	5.472	7.210	7.575	1.00	0.00	H
ATOM	210	SD	MET	13	6.742	9.090	7.069	1.00	0.00	S
ATOM	211	CE	MET	13	5.309	9.834	6.309	1.00	0.00	C
ATOM	212	HE1	MET	13	4.427	9.783	6.947	1.00	0.00	H
ATOM	213	HE2	MET	13	5.546	10.887	6.159	1.00	0.00	H
ATOM	214	HE3	MET	13	5.127	9.410	5.321	1.00	0.00	H
ATOM	215	C	MET	13	5.162	4.559	4.331	1.00	0.00	C
ATOM	216	O	MET	13	5.860	3.951	3.544	1.00	0.00	O
ATOM	217	N	TYR	14	3.939	4.902	4.081	1.00	0.00	N
ATOM	218	H	TYR	14	3.452	5.489	4.744	1.00	0.00	H
ATOM	219	CA	TYR	14	3.150	4.159	3.102	1.00	0.00	C
ATOM	220	HA	TYR	14	3.088	3.103	3.364	1.00	0.00	H
ATOM	221	CB	TYR	14	1.648	4.650	3.284	1.00	0.00	C
ATOM	222	HB2	TYR	14	1.384	4.305	4.284	1.00	0.00	H
ATOM	223	HB3	TYR	14	1.160	3.954	2.602	1.00	0.00	H
ATOM	224	CG	TYR	14	1.219	6.037	2.929	1.00	0.00	C
ATOM	225	CD1	TYR	14	1.076	6.988	3.967	1.00	0.00	C
ATOM	226	HD1	TYR	14	1.325	6.719	4.983	1.00	0.00	H
ATOM	227	CE1	TYR	14	0.839	8.352	3.587	1.00	0.00	C
ATOM	228	HE1	TYR	14	0.799	9.118	4.347	1.00	0.00	H
ATOM	229	CZ	TYR	14	0.731	8.734	2.281	1.00	0.00	C
ATOM	230	OH	TYR	14	0.582	10.066	2.054	1.00	0.00	O
ATOM	231	HH	TYR	14	0.573	10.457	2.931	1.00	0.00	H
ATOM	232	CE2	TYR	14	0.778	7.746	1.217	1.00	0.00	C
ATOM	233	HE2	TYR	14	0.709	8.072	0.189	1.00	0.00	H
ATOM	234	CD2	TYR	14	1.054	6.431	1.514	1.00	0.00	C
ATOM	235	HD2	TYR	14	1.071	5.628	0.792	1.00	0.00	H
ATOM	236	C	TYR	14	3.481	4.462	1.605	1.00	0.00	C
ATOM	237	O	TYR	14	3.108	3.711	0.735	1.00	0.00	O
ATOM	238	N	ARG	15	4.059	5.704	1.491	1.00	0.00	N
ATOM	239	H	ARG	15	4.167	6.113	2.407	1.00	0.00	H
ATOM	240	CA	ARG	15	4.748	6.327	0.303	1.00	0.00	C
ATOM	241	HA	ARG	15	4.728	5.575	-0.486	1.00	0.00	H
ATOM	242	CB	ARG	15	3.897	7.501	-0.197	1.00	0.00	C
ATOM	243	HB2	ARG	15	3.601	8.205	0.582	1.00	0.00	H
ATOM	244	HB3	ARG	15	2.915	7.171	-0.535	1.00	0.00	H
ATOM	245	CG	ARG	15	4.545	8.142	-1.467	1.00	0.00	C
ATOM	246	HG2	ARG	15	4.708	7.454	-2.296	1.00	0.00	H
ATOM	247	HG3	ARG	15	5.496	8.488	-1.064	1.00	0.00	H

```
ATOM    248  CD  ARG    15       3.873   9.420  -1.970  1.00  0.00           C
ATOM    249  HD2 ARG    15       3.477   9.994  -1.132  1.00  0.00           H
ATOM    250  HD3 ARG    15       3.033   9.153  -2.609  1.00  0.00           H
ATOM    251  NE  ARG    15       4.806  10.215  -2.804  1.00  0.00           N
ATOM    252  HE  ARG    15       5.701  10.464  -2.409  1.00  0.00           H
ATOM    253  CZ  ARG    15       4.481  10.860  -3.902  1.00  0.00           C
ATOM    254  NH1 ARG    15       3.459  10.522  -4.649  1.00  0.00           N
ATOM    255 HH11 ARG    15       2.978   9.653  -4.467  1.00  0.00           H
ATOM    256 HH12 ARG    15       3.384  10.801  -5.617  1.00  0.00           H
ATOM    257  NH2 ARG    15       5.145  11.890  -4.316  1.00  0.00           N
ATOM    258 HH21 ARG    15       4.890  12.386  -5.157  1.00  0.00           H
ATOM    259 HH22 ARG    15       5.787  12.378  -3.709  1.00  0.00           H
ATOM    260  C   ARG    15       6.183   6.679   0.734  1.00  0.00           C
ATOM    261  O   ARG    15       6.318   7.275   1.773  1.00  0.00           O
ATOM    262  N   TYR    16       7.224   6.273  -0.018  1.00  0.00           N
ATOM    263  H   TYR    16       6.961   5.835  -0.889  1.00  0.00           H
ATOM    264  CA  TYR    16       8.613   6.567   0.286  1.00  0.00           C
ATOM    265  HA  TYR    16       8.695   7.505   0.833  1.00  0.00           H
ATOM    266  CB  TYR    16       9.037   5.419   1.074  1.00  0.00           C
ATOM    267  HB2 TYR    16       8.378   5.279   1.930  1.00  0.00           H
ATOM    268  HB3 TYR    16      10.007   5.691   1.491  1.00  0.00           H
ATOM    269  CG  TYR    16       9.156   4.000   0.415  1.00  0.00           C
ATOM    270  CD1 TYR    16       8.100   3.093   0.717  1.00  0.00           C
ATOM    271  HD1 TYR    16       7.326   3.541   1.322  1.00  0.00           H
ATOM    272  CE1 TYR    16       8.183   1.797   0.233  1.00  0.00           C
ATOM    273  HE1 TYR    16       7.367   1.119   0.436  1.00  0.00           H
ATOM    274  CZ  TYR    16       9.300   1.374  -0.488  1.00  0.00           C
ATOM    275  OH  TYR    16       9.269   0.050  -1.005  1.00  0.00           O
ATOM    276  HH  TYR    16       9.921  -0.100  -1.695  1.00  0.00           H
ATOM    277  CE2 TYR    16      10.372   2.228  -0.809  1.00  0.00           C
ATOM    278  HE2 TYR    16      11.197   1.995  -1.465  1.00  0.00           H
ATOM    279  CD2 TYR    16      10.304   3.561  -0.291  1.00  0.00           C
ATOM    280  HD2 TYR    16      11.127   4.198  -0.578  1.00  0.00           H
ATOM    281  C   TYR    16       9.463   6.979  -0.972  1.00  0.00           C
ATOM    282  O   TYR    16       9.178   6.735  -2.148  1.00  0.00           O
ATOM    283  N   NME    17      10.640   7.516  -0.657  1.00  0.00           N
ATOM    284  H   NME    17      10.988   7.653   0.281  1.00  0.00           H
ATOM    285  CH3 NME    17      11.708   7.715  -1.632  1.00  0.00           C
ATOM    286 HH31 NME    17      11.581   7.211  -2.590  1.00  0.00           H
ATOM    287 HH32 NME    17      11.762   8.784  -1.836  1.00  0.00           H
ATOM    288 HH33 NME    17      12.731   7.615  -1.269  1.00  0.00           H
TER     289      NME    17
END
```

H1 dimer simulation:

```
#Cluster   Frames      Frac  AvgDist    Stdev Centroid AvgCDist
       0   156799     0.784    3.156    1.025    96112    4.735
       1    30747     0.154    1.265    0.640    77157    5.266
       2     7133     0.036    1.998    0.752     3249    5.098
       3     5142     0.026    2.692    1.006    58827    4.513
       4      179     0.001    0.000    0.000    21711    5.336
```

Cluster 0, Figure 3A:

```
ATOM      1 HH31 ACE     1      16.002   3.050 -11.564  1.00  0.00           H
ATOM      2  CH3 ACE     1      16.474   3.155 -10.588  1.00  0.00           C
ATOM      3 HH32 ACE     1      16.905   4.156 -10.581  1.00  0.00           H
```

ATOM	4	HH33	ACE	1	17.249	2.405	-10.427	1.00	0.00	H
ATOM	5	C	ACE	1	15.572	3.085	-9.345	1.00	0.00	C
ATOM	6	O	ACE	1	15.555	4.100	-8.604	1.00	0.00	O
ATOM	7	N	ASN	2	14.893	1.968	-9.129	1.00	0.00	N
ATOM	8	H	ASN	2	14.851	1.415	-9.974	1.00	0.00	H
ATOM	9	CA	ASN	2	13.896	1.709	-8.038	1.00	0.00	C
ATOM	10	HA	ASN	2	13.297	2.615	-7.942	1.00	0.00	H
ATOM	11	CB	ASN	2	12.906	0.657	-8.631	1.00	0.00	C
ATOM	12	HB2	ASN	2	13.361	-0.307	-8.859	1.00	0.00	H
ATOM	13	HB3	ASN	2	12.491	1.068	-9.552	1.00	0.00	H
ATOM	14	CG	ASN	2	11.638	0.325	-7.824	1.00	0.00	C
ATOM	15	OD1	ASN	2	11.473	0.689	-6.644	1.00	0.00	O
ATOM	16	ND2	ASN	2	10.739	-0.422	-8.410	1.00	0.00	N
ATOM	17	HD21	ASN	2	10.117	-0.914	-7.785	1.00	0.00	H
ATOM	18	HD22	ASN	2	10.856	-0.672	-9.381	1.00	0.00	H
ATOM	19	C	ASN	2	14.562	1.416	-6.690	1.00	0.00	C
ATOM	20	O	ASN	2	15.613	0.725	-6.681	1.00	0.00	O
ATOM	21	N	ASP	3	14.124	1.970	-5.568	1.00	0.00	N
ATOM	22	H	ASP	3	13.363	2.634	-5.556	1.00	0.00	H
ATOM	23	CA	ASP	3	14.802	1.932	-4.271	1.00	0.00	C
ATOM	24	HA	ASP	3	15.637	2.634	-4.284	1.00	0.00	H
ATOM	25	CB	ASP	3	13.786	2.354	-3.150	1.00	0.00	C
ATOM	26	HB2	ASP	3	12.941	1.669	-3.091	1.00	0.00	H
ATOM	27	HB3	ASP	3	13.478	3.369	-3.401	1.00	0.00	H
ATOM	28	CG	ASP	3	14.405	2.493	-1.745	1.00	0.00	C
ATOM	29	OD1	ASP	3	14.490	1.542	-0.910	1.00	0.00	O
ATOM	30	OD2	ASP	3	14.700	3.651	-1.320	1.00	0.00	O
ATOM	31	C	ASP	3	15.425	0.627	-3.916	1.00	0.00	C
ATOM	32	O	ASP	3	16.601	0.643	-3.476	1.00	0.00	O
ATOM	33	N	TYR	4	14.922	-0.591	-4.283	1.00	0.00	N
ATOM	34	H	TYR	4	14.023	-0.559	-4.743	1.00	0.00	H
ATOM	35	CA	TYR	4	15.364	-1.937	-3.904	1.00	0.00	C
ATOM	36	HA	TYR	4	15.318	-1.933	-2.815	1.00	0.00	H
ATOM	37	CB	TYR	4	14.353	-3.054	-4.343	1.00	0.00	C
ATOM	38	HB2	TYR	4	14.264	-3.046	-5.429	1.00	0.00	H
ATOM	39	HB3	TYR	4	13.388	-2.650	-4.037	1.00	0.00	H
ATOM	40	CG	TYR	4	14.563	-4.382	-3.739	1.00	0.00	C
ATOM	41	CD1	TYR	4	15.189	-5.366	-4.562	1.00	0.00	C
ATOM	42	HD1	TYR	4	15.430	-5.139	-5.590	1.00	0.00	H
ATOM	43	CE1	TYR	4	15.551	-6.587	-3.921	1.00	0.00	C
ATOM	44	HE1	TYR	4	16.040	-7.316	-4.551	1.00	0.00	H
ATOM	45	CZ	TYR	4	15.181	-6.883	-2.598	1.00	0.00	C
ATOM	46	OH	TYR	4	15.576	-8.105	-2.085	1.00	0.00	O
ATOM	47	HH	TYR	4	15.095	-8.331	-1.285	1.00	0.00	H
ATOM	48	CE2	TYR	4	14.535	-5.911	-1.768	1.00	0.00	C
ATOM	49	HE2	TYR	4	14.401	-6.099	-0.713	1.00	0.00	H
ATOM	50	CD2	TYR	4	14.223	-4.657	-2.400	1.00	0.00	C
ATOM	51	HD2	TYR	4	13.773	-3.950	-1.719	1.00	0.00	H
ATOM	52	C	TYR	4	16.743	-2.282	-4.524	1.00	0.00	C
ATOM	53	O	TYR	4	17.578	-2.990	-3.916	1.00	0.00	O
ATOM	54	N	GLU	5	17.054	-1.718	-5.730	1.00	0.00	N
ATOM	55	H	GLU	5	16.263	-1.222	-6.115	1.00	0.00	H
ATOM	56	CA	GLU	5	18.382	-1.849	-6.337	1.00	0.00	C
ATOM	57	HA	GLU	5	18.558	-2.924	-6.301	1.00	0.00	H
ATOM	58	CB	GLU	5	18.461	-1.306	-7.754	1.00	0.00	C
ATOM	59	HB2	GLU	5	19.524	-1.366	-7.986	1.00	0.00	H
ATOM	60	HB3	GLU	5	18.183	-0.252	-7.746	1.00	0.00	H
ATOM	61	CG	GLU	5	17.789	-2.099	-8.852	1.00	0.00	C
ATOM	62	HG2	GLU	5	16.718	-2.143	-8.656	1.00	0.00	H
ATOM	63	HG3	GLU	5	18.156	-3.120	-8.745	1.00	0.00	H
ATOM	64	CD	GLU	5	18.014	-1.515	-10.286	1.00	0.00	C
ATOM	65	OE1	GLU	5	17.293	-1.872	-11.255	1.00	0.00	O
ATOM	66	OE2	GLU	5	19.050	-0.824	-10.469	1.00	0.00	O

ATOM	67	C	GLU	5	19.526	-1.189	-5.587	1.00	0.00	C
ATOM	68	O	GLU	5	20.662	-1.596	-5.661	1.00	0.00	O
ATOM	69	N	ASP	6	19.232	-0.109	-4.845	1.00	0.00	N
ATOM	70	H	ASP	6	18.270	0.197	-4.830	1.00	0.00	H
ATOM	71	CA	ASP	6	20.083	0.659	-3.955	1.00	0.00	C
ATOM	72	HA	ASP	6	21.109	0.583	-4.316	1.00	0.00	H
ATOM	73	CB	ASP	6	19.632	2.108	-4.097	1.00	0.00	C
ATOM	74	HB2	ASP	6	18.549	2.223	-4.044	1.00	0.00	H
ATOM	75	HB3	ASP	6	19.980	2.360	-5.098	1.00	0.00	H
ATOM	76	CG	ASP	6	20.226	3.082	-2.989	1.00	0.00	C
ATOM	77	OD1	ASP	6	21.413	3.381	-2.970	1.00	0.00	O
ATOM	78	OD2	ASP	6	19.341	3.546	-2.193	1.00	0.00	O
ATOM	79	C	ASP	6	19.975	0.168	-2.516	1.00	0.00	C
ATOM	80	O	ASP	6	20.981	0.185	-1.800	1.00	0.00	O
ATOM	81	N	ARG	7	18.831	-0.330	-2.072	1.00	0.00	N
ATOM	82	H	ARG	7	18.004	-0.144	-2.621	1.00	0.00	H
ATOM	83	CA	ARG	7	18.625	-0.875	-0.733	1.00	0.00	C
ATOM	84	HA	ARG	7	18.836	-0.088	-0.009	1.00	0.00	H
ATOM	85	CB	ARG	7	17.142	-1.407	-0.684	1.00	0.00	C
ATOM	86	HB2	ARG	7	17.044	-2.299	-1.302	1.00	0.00	H
ATOM	87	HB3	ARG	7	16.511	-0.648	-1.147	1.00	0.00	H
ATOM	88	CG	ARG	7	16.772	-1.757	0.785	1.00	0.00	C
ATOM	89	HG2	ARG	7	17.158	-2.724	1.108	1.00	0.00	H
ATOM	90	HG3	ARG	7	15.709	-1.993	0.736	1.00	0.00	H
ATOM	91	CD	ARG	7	16.848	-0.616	1.866	1.00	0.00	C
ATOM	92	HD2	ARG	7	17.846	-0.213	2.037	1.00	0.00	H
ATOM	93	HD3	ARG	7	16.395	-1.053	2.756	1.00	0.00	H
ATOM	94	NE	ARG	7	16.061	0.573	1.538	1.00	0.00	N
ATOM	95	HE	ARG	7	15.623	0.594	0.628	1.00	0.00	H
ATOM	96	CZ	ARG	7	15.847	1.660	2.232	1.00	0.00	C
ATOM	97	NH1	ARG	7	16.345	1.846	3.402	1.00	0.00	N
ATOM	98	HH11	ARG	7	16.990	1.144	3.736	1.00	0.00	H
ATOM	99	HH12	ARG	7	16.140	2.634	4.000	1.00	0.00	H
ATOM	100	NH2	ARG	7	15.183	2.631	1.734	1.00	0.00	N
ATOM	101	HH21	ARG	7	14.570	2.458	0.950	1.00	0.00	H
ATOM	102	HH22	ARG	7	15.227	3.505	2.237	1.00	0.00	H
ATOM	103	C	ARG	7	19.616	-1.999	-0.365	1.00	0.00	C
ATOM	104	O	ARG	7	20.119	-1.999	0.735	1.00	0.00	O
ATOM	105	N	TYR	8	19.834	-2.942	-1.255	1.00	0.00	N
ATOM	106	H	TYR	8	19.303	-2.855	-2.109	1.00	0.00	H
ATOM	107	CA	TYR	8	20.591	-4.071	-0.960	1.00	0.00	C
ATOM	108	HA	TYR	8	20.088	-4.774	-0.296	1.00	0.00	H
ATOM	109	CB	TYR	8	20.697	-4.929	-2.236	1.00	0.00	C
ATOM	110	HB2	TYR	8	20.997	-4.450	-3.168	1.00	0.00	H
ATOM	111	HB3	TYR	8	19.706	-5.371	-2.332	1.00	0.00	H
ATOM	112	CG	TYR	8	21.620	-6.169	-2.243	1.00	0.00	C
ATOM	113	CD1	TYR	8	22.962	-6.007	-2.728	1.00	0.00	C
ATOM	114	HD1	TYR	8	23.382	-5.040	-2.962	1.00	0.00	H
ATOM	115	CE1	TYR	8	23.806	-7.113	-2.776	1.00	0.00	C
ATOM	116	HE1	TYR	8	24.785	-7.039	-3.225	1.00	0.00	H
ATOM	117	CZ	TYR	8	23.464	-8.413	-2.318	1.00	0.00	C
ATOM	118	OH	TYR	8	24.410	-9.452	-2.177	1.00	0.00	O
ATOM	119	HH	TYR	8	25.283	-9.059	-2.249	1.00	0.00	H
ATOM	120	CE2	TYR	8	22.175	-8.502	-1.692	1.00	0.00	C
ATOM	121	HE2	TYR	8	21.916	-9.467	-1.280	1.00	0.00	H
ATOM	122	CD2	TYR	8	21.266	-7.414	-1.645	1.00	0.00	C
ATOM	123	HD2	TYR	8	20.247	-7.601	-1.339	1.00	0.00	H
ATOM	124	C	TYR	8	22.072	-3.766	-0.537	1.00	0.00	C
ATOM	125	O	TYR	8	22.645	-4.497	0.243	1.00	0.00	O
ATOM	126	N	TYR	9	22.573	-2.628	-1.075	1.00	0.00	N
ATOM	127	H	TYR	9	22.061	-2.103	-1.770	1.00	0.00	H
ATOM	128	CA	TYR	9	23.878	-2.147	-0.737	1.00	0.00	C
ATOM	129	HA	TYR	9	24.438	-3.049	-0.491	1.00	0.00	H

ATOM	130	CB	TYR	9	24.547	-1.373	-1.932	1.00	0.00	C
ATOM	131	HB2	TYR	9	25.436	-0.874	-1.547	1.00	0.00	H
ATOM	132	HB3	TYR	9	23.791	-0.644	-2.224	1.00	0.00	H
ATOM	133	CG	TYR	9	24.793	-2.238	-3.130	1.00	0.00	C
ATOM	134	CD1	TYR	9	25.837	-3.185	-3.051	1.00	0.00	C
ATOM	135	HD1	TYR	9	26.476	-3.239	-2.182	1.00	0.00	H
ATOM	136	CE1	TYR	9	26.164	-3.956	-4.217	1.00	0.00	C
ATOM	137	HE1	TYR	9	27.005	-4.630	-4.293	1.00	0.00	H
ATOM	138	CZ	TYR	9	25.309	-3.933	-5.346	1.00	0.00	C
ATOM	139	OH	TYR	9	25.642	-4.511	-6.495	1.00	0.00	O
ATOM	140	HH	TYR	9	25.105	-4.194	-7.225	1.00	0.00	H
ATOM	141	CE2	TYR	9	24.117	-3.154	-5.248	1.00	0.00	C
ATOM	142	HE2	TYR	9	23.430	-3.123	-6.081	1.00	0.00	H
ATOM	143	CD2	TYR	9	23.912	-2.227	-4.202	1.00	0.00	C
ATOM	144	HD2	TYR	9	23.097	-1.529	-4.070	1.00	0.00	H
ATOM	145	C	TYR	9	23.832	-1.374	0.575	1.00	0.00	C
ATOM	146	O	TYR	9	24.573	-1.772	1.434	1.00	0.00	O
ATOM	147	N	ARG	10	22.953	-0.400	0.641	1.00	0.00	N
ATOM	148	H	ARG	10	22.325	-0.280	-0.140	1.00	0.00	H
ATOM	149	CA	ARG	10	22.911	0.518	1.763	1.00	0.00	C
ATOM	150	HA	ARG	10	23.855	1.063	1.738	1.00	0.00	H
ATOM	151	CB	ARG	10	21.769	1.559	1.416	1.00	0.00	C
ATOM	152	HB2	ARG	10	21.589	2.210	2.272	1.00	0.00	H
ATOM	153	HB3	ARG	10	20.897	0.956	1.163	1.00	0.00	H
ATOM	154	CG	ARG	10	22.051	2.442	0.150	1.00	0.00	C
ATOM	155	HG2	ARG	10	21.019	2.704	-0.083	1.00	0.00	H
ATOM	156	HG3	ARG	10	22.719	1.865	-0.490	1.00	0.00	H
ATOM	157	CD	ARG	10	22.802	3.684	0.456	1.00	0.00	C
ATOM	158	HD2	ARG	10	23.843	3.387	0.585	1.00	0.00	H
ATOM	159	HD3	ARG	10	22.285	4.232	1.244	1.00	0.00	H
ATOM	160	NE	ARG	10	22.711	4.662	-0.624	1.00	0.00	N
ATOM	161	HE	ARG	10	22.261	4.385	-1.485	1.00	0.00	H
ATOM	162	CZ	ARG	10	23.141	5.889	-0.535	1.00	0.00	C
ATOM	163	NH1	ARG	10	23.831	6.285	0.439	1.00	0.00	N
ATOM	164	HH11	ARG	10	23.966	5.642	1.206	1.00	0.00	H
ATOM	165	HH12	ARG	10	24.310	7.174	0.416	1.00	0.00	H
ATOM	166	NH2	ARG	10	22.961	6.692	-1.515	1.00	0.00	N
ATOM	167	HH21	ARG	10	22.535	6.435	-2.394	1.00	0.00	H
ATOM	168	HH22	ARG	10	23.601	7.473	-1.531	1.00	0.00	H
ATOM	169	C	ARG	10	22.711	-0.149	3.144	1.00	0.00	C
ATOM	170	O	ARG	10	23.186	0.368	4.127	1.00	0.00	O
ATOM	171	N	GLU	11	21.907	-1.200	3.169	1.00	0.00	N
ATOM	172	H	GLU	11	21.430	-1.501	2.330	1.00	0.00	H
ATOM	173	CA	GLU	11	21.579	-1.890	4.431	1.00	0.00	C
ATOM	174	HA	GLU	11	21.537	-1.201	5.273	1.00	0.00	H
ATOM	175	CB	GLU	11	20.164	-2.531	4.301	1.00	0.00	C
ATOM	176	HB2	GLU	11	19.867	-3.155	5.144	1.00	0.00	H
ATOM	177	HB3	GLU	11	20.384	-3.237	3.500	1.00	0.00	H
ATOM	178	CG	GLU	11	19.022	-1.583	3.971	1.00	0.00	C
ATOM	179	HG2	GLU	11	18.055	-2.070	3.850	1.00	0.00	H
ATOM	180	HG3	GLU	11	19.404	-1.023	3.117	1.00	0.00	H
ATOM	181	CD	GLU	11	18.736	-0.593	5.069	1.00	0.00	C
ATOM	182	OE1	GLU	11	18.393	0.560	4.770	1.00	0.00	O
ATOM	183	OE2	GLU	11	18.612	-0.970	6.206	1.00	0.00	O
ATOM	184	C	GLU	11	22.655	-2.949	4.804	1.00	0.00	C
ATOM	185	O	GLU	11	23.090	-2.964	6.000	1.00	0.00	O
ATOM	186	N	ASN	12	23.304	-3.611	3.848	1.00	0.00	N
ATOM	187	H	ASN	12	23.096	-3.429	2.877	1.00	0.00	H
ATOM	188	CA	ASN	12	24.322	-4.547	4.192	1.00	0.00	C
ATOM	189	HA	ASN	12	24.020	-5.167	5.037	1.00	0.00	H
ATOM	190	CB	ASN	12	24.502	-5.466	2.943	1.00	0.00	C
ATOM	191	HB2	ASN	12	24.725	-4.859	2.065	1.00	0.00	H
ATOM	192	HB3	ASN	12	23.476	-5.832	2.901	1.00	0.00	H

ATOM	193	CG	ASN	12	25.554	-6.600	3.107	1.00	0.00	C
ATOM	194	OD1	ASN	12	25.197	-7.738	3.433	1.00	0.00	O
ATOM	195	ND2	ASN	12	26.847	-6.400	2.852	1.00	0.00	N
ATOM	196	HD21	ASN	12	27.448	-7.210	2.891	1.00	0.00	H
ATOM	197	HD22	ASN	12	27.016	-5.561	2.316	1.00	0.00	H
ATOM	198	C	ASN	12	25.645	-3.843	4.578	1.00	0.00	C
ATOM	199	O	ASN	12	26.344	-4.295	5.511	1.00	0.00	O
ATOM	200	N	MET	13	25.986	-2.675	3.919	1.00	0.00	N
ATOM	201	H	MET	13	25.394	-2.395	3.150	1.00	0.00	H
ATOM	202	CA	MET	13	27.273	-1.955	3.993	1.00	0.00	C
ATOM	203	HA	MET	13	28.000	-2.758	4.114	1.00	0.00	H
ATOM	204	CB	MET	13	27.563	-1.204	2.674	1.00	0.00	C
ATOM	205	HB2	MET	13	28.448	-0.590	2.836	1.00	0.00	H
ATOM	206	HB3	MET	13	26.634	-0.726	2.361	1.00	0.00	H
ATOM	207	CG	MET	13	27.890	-2.101	1.462	1.00	0.00	C
ATOM	208	HG2	MET	13	28.157	-1.492	0.598	1.00	0.00	H
ATOM	209	HG3	MET	13	26.925	-2.553	1.237	1.00	0.00	H
ATOM	210	SD	MET	13	29.108	-3.421	1.674	1.00	0.00	S
ATOM	211	CE	MET	13	30.691	-2.429	1.765	1.00	0.00	C
ATOM	212	HE1	MET	13	30.754	-1.881	2.705	1.00	0.00	H
ATOM	213	HE2	MET	13	31.575	-3.067	1.791	1.00	0.00	H
ATOM	214	HE3	MET	13	30.732	-1.732	0.928	1.00	0.00	H
ATOM	215	C	MET	13	27.389	-0.960	5.101	1.00	0.00	C
ATOM	216	O	MET	13	28.475	-0.614	5.596	1.00	0.00	O
ATOM	217	N	TYR	14	26.307	-0.350	5.613	1.00	0.00	N
ATOM	218	H	TYR	14	25.470	-0.730	5.195	1.00	0.00	H
ATOM	219	CA	TYR	14	26.323	0.816	6.504	1.00	0.00	C
ATOM	220	HA	TYR	14	27.269	0.826	7.045	1.00	0.00	H
ATOM	221	CB	TYR	14	26.267	2.162	5.723	1.00	0.00	C
ATOM	222	HB2	TYR	14	26.290	2.984	6.439	1.00	0.00	H
ATOM	223	HB3	TYR	14	25.286	2.162	5.249	1.00	0.00	H
ATOM	224	CG	TYR	14	27.348	2.444	4.741	1.00	0.00	C
ATOM	225	CD1	TYR	14	28.735	2.473	5.055	1.00	0.00	C
ATOM	226	HD1	TYR	14	29.081	2.267	6.057	1.00	0.00	H
ATOM	227	CE1	TYR	14	29.681	2.724	4.044	1.00	0.00	C
ATOM	228	HE1	TYR	14	30.740	2.583	4.199	1.00	0.00	H
ATOM	229	CZ	TYR	14	29.309	2.971	2.709	1.00	0.00	C
ATOM	230	OH	TYR	14	30.271	3.104	1.748	1.00	0.00	O
ATOM	231	HH	TYR	14	31.129	3.304	2.129	1.00	0.00	H
ATOM	232	CE2	TYR	14	27.927	2.920	2.398	1.00	0.00	C
ATOM	233	HE2	TYR	14	27.596	3.070	1.380	1.00	0.00	H
ATOM	234	CD2	TYR	14	26.985	2.748	3.401	1.00	0.00	C
ATOM	235	HD2	TYR	14	25.936	2.841	3.161	1.00	0.00	H
ATOM	236	C	TYR	14	25.300	0.862	7.584	1.00	0.00	C
ATOM	237	O	TYR	14	25.571	1.568	8.570	1.00	0.00	O
ATOM	238	N	ARG	15	24.210	0.106	7.547	1.00	0.00	N
ATOM	239	H	ARG	15	23.971	-0.372	6.691	1.00	0.00	H
ATOM	240	CA	ARG	15	23.232	-0.027	8.603	1.00	0.00	C
ATOM	241	HA	ARG	15	23.464	0.675	9.405	1.00	0.00	H
ATOM	242	CB	ARG	15	21.846	0.407	8.059	1.00	0.00	C
ATOM	243	HB2	ARG	15	20.991	0.167	8.692	1.00	0.00	H
ATOM	244	HB3	ARG	15	21.723	-0.306	7.244	1.00	0.00	H
ATOM	245	CG	ARG	15	21.768	1.827	7.563	1.00	0.00	C
ATOM	246	HG2	ARG	15	22.473	2.054	6.764	1.00	0.00	H
ATOM	247	HG3	ARG	15	21.935	2.456	8.438	1.00	0.00	H
ATOM	248	CD	ARG	15	20.432	2.100	6.905	1.00	0.00	C
ATOM	249	HD2	ARG	15	19.608	1.430	7.148	1.00	0.00	H
ATOM	250	HD3	ARG	15	20.668	1.877	5.865	1.00	0.00	H
ATOM	251	NE	ARG	15	20.018	3.511	6.828	1.00	0.00	N
ATOM	252	HE	ARG	15	20.618	4.220	7.224	1.00	0.00	H
ATOM	253	CZ	ARG	15	19.006	3.960	6.168	1.00	0.00	C
ATOM	254	NH1	ARG	15	18.126	3.169	5.597	1.00	0.00	N
ATOM	255	HH11	ARG	15	18.354	2.217	5.348	1.00	0.00	H

ATOM	256	HH12	ARG	15	17.187	3.540	5.563	1.00	0.00	H
ATOM	257	NH2	ARG	15	18.722	5.205	6.198	1.00	0.00	N
ATOM	258	HH21	ARG	15	19.435	5.737	6.677	1.00	0.00	H
ATOM	259	HH22	ARG	15	18.001	5.702	5.696	1.00	0.00	H
ATOM	260	C	ARG	15	23.218	-1.427	9.228	1.00	0.00	C
ATOM	261	O	ARG	15	22.616	-1.552	10.241	1.00	0.00	O
ATOM	262	N	TYR	16	23.950	-2.394	8.612	1.00	0.00	N
ATOM	263	H	TYR	16	24.402	-2.147	7.744	1.00	0.00	H
ATOM	264	CA	TYR	16	24.131	-3.729	9.077	1.00	0.00	C
ATOM	265	HA	TYR	16	24.615	-4.270	8.264	1.00	0.00	H
ATOM	266	CB	TYR	16	25.230	-3.727	10.191	1.00	0.00	C
ATOM	267	HB2	TYR	16	25.561	-4.743	10.405	1.00	0.00	H
ATOM	268	HB3	TYR	16	24.629	-3.340	11.014	1.00	0.00	H
ATOM	269	CG	TYR	16	26.399	-2.737	10.047	1.00	0.00	C
ATOM	270	CD1	TYR	16	27.331	-2.932	8.955	1.00	0.00	C
ATOM	271	HD1	TYR	16	27.338	-3.804	8.317	1.00	0.00	H
ATOM	272	CE1	TYR	16	28.355	-1.975	8.751	1.00	0.00	C
ATOM	273	HE1	TYR	16	29.163	-2.097	8.046	1.00	0.00	H
ATOM	274	CZ	TYR	16	28.457	-0.891	9.628	1.00	0.00	C
ATOM	275	OH	TYR	16	29.391	-0.058	9.392	1.00	0.00	O
ATOM	276	HH	TYR	16	29.793	-0.224	8.536	1.00	0.00	H
ATOM	277	CE2	TYR	16	27.533	-0.658	10.662	1.00	0.00	C
ATOM	278	HE2	TYR	16	27.563	0.228	11.278	1.00	0.00	H
ATOM	279	CD2	TYR	16	26.475	-1.563	10.859	1.00	0.00	C
ATOM	280	HD2	TYR	16	25.811	-1.391	11.694	1.00	0.00	H
ATOM	281	C	TYR	16	22.815	-4.471	9.227	1.00	0.00	C
ATOM	282	O	TYR	16	22.726	-5.213	10.207	1.00	0.00	O
ATOM	283	N	NME	17	21.821	-4.302	8.350	1.00	0.00	N
ATOM	284	H	NME	17	22.135	-3.798	7.533	1.00	0.00	H
ATOM	285	CH3	NME	17	20.573	-5.069	8.323	1.00	0.00	C
ATOM	286	HH31	NME	17	20.050	-4.784	7.410	1.00	0.00	H
ATOM	287	HH32	NME	17	20.788	-6.138	8.344	1.00	0.00	H
ATOM	288	HH33	NME	17	19.867	-4.961	9.147	1.00	0.00	H
TER	289		NME	17						
ATOM	289	HH31	ACE	18	14.353	12.635	11.058	1.00	0.00	H
ATOM	290	CH3	ACE	18	14.456	11.582	11.318	1.00	0.00	C
ATOM	291	HH32	ACE	18	13.955	11.406	12.270	1.00	0.00	H
ATOM	292	HH33	ACE	18	15.467	11.216	11.499	1.00	0.00	H
ATOM	293	C	ACE	18	13.848	10.683	10.294	1.00	0.00	C
ATOM	294	O	ACE	18	13.000	9.858	10.678	1.00	0.00	O
ATOM	295	N	ASN	19	14.315	10.710	9.028	1.00	0.00	N
ATOM	296	H	ASN	19	14.974	11.471	8.943	1.00	0.00	H
ATOM	297	CA	ASN	19	13.837	10.031	7.819	1.00	0.00	C
ATOM	298	HA	ASN	19	13.070	9.347	8.182	1.00	0.00	H
ATOM	299	CB	ASN	19	13.178	11.152	6.989	1.00	0.00	C
ATOM	300	HB2	ASN	19	13.944	11.741	6.485	1.00	0.00	H
ATOM	301	HB3	ASN	19	12.580	11.719	7.703	1.00	0.00	H
ATOM	302	CG	ASN	19	12.125	10.791	5.948	1.00	0.00	C
ATOM	303	OD1	ASN	19	11.668	9.616	5.820	1.00	0.00	O
ATOM	304	ND2	ASN	19	11.714	11.700	5.092	1.00	0.00	N
ATOM	305	HD21	ASN	19	10.886	11.465	4.563	1.00	0.00	H
ATOM	306	HD22	ASN	19	11.836	12.681	5.299	1.00	0.00	H
ATOM	307	C	ASN	19	14.794	9.122	7.137	1.00	0.00	C
ATOM	308	O	ASN	19	15.999	9.296	7.234	1.00	0.00	O
ATOM	309	N	ASP	20	14.272	8.162	6.398	1.00	0.00	N
ATOM	310	H	ASP	20	13.272	8.015	6.400	1.00	0.00	H
ATOM	311	CA	ASP	20	15.147	7.200	5.675	1.00	0.00	C
ATOM	312	HA	ASP	20	15.844	6.703	6.350	1.00	0.00	H
ATOM	313	CB	ASP	20	14.243	6.095	5.127	1.00	0.00	C
ATOM	314	HB2	ASP	20	13.423	6.457	4.507	1.00	0.00	H
ATOM	315	HB3	ASP	20	13.847	5.661	6.045	1.00	0.00	H
ATOM	316	CG	ASP	20	14.881	5.007	4.288	1.00	0.00	C
ATOM	317	OD1	ASP	20	15.059	5.224	3.091	1.00	0.00	O

ATOM	318	OD2	ASP	20	15.586	4.104	4.795	1.00	0.00	O
ATOM	319	C	ASP	20	16.018	7.871	4.568	1.00	0.00	C
ATOM	320	O	ASP	20	17.178	7.480	4.358	1.00	0.00	O
ATOM	321	N	TYR	21	15.508	8.896	3.843	1.00	0.00	N
ATOM	322	H	TYR	21	14.545	9.058	4.103	1.00	0.00	H
ATOM	323	CA	TYR	21	16.301	9.716	2.873	1.00	0.00	C
ATOM	324	HA	TYR	21	16.782	9.106	2.108	1.00	0.00	H
ATOM	325	CB	TYR	21	15.292	10.686	2.147	1.00	0.00	C
ATOM	326	HB2	TYR	21	14.826	11.275	2.937	1.00	0.00	H
ATOM	327	HB3	TYR	21	14.526	10.102	1.637	1.00	0.00	H
ATOM	328	CG	TYR	21	16.008	11.615	1.149	1.00	0.00	C
ATOM	329	CD1	TYR	21	16.961	11.172	0.221	1.00	0.00	C
ATOM	330	HD1	TYR	21	17.186	10.116	0.252	1.00	0.00	H
ATOM	331	CE1	TYR	21	17.501	12.069	-0.727	1.00	0.00	C
ATOM	332	HE1	TYR	21	18.361	11.682	-1.254	1.00	0.00	H
ATOM	333	CZ	TYR	21	17.100	13.446	-0.738	1.00	0.00	C
ATOM	334	OH	TYR	21	17.640	14.342	-1.539	1.00	0.00	O
ATOM	335	HH	TYR	21	17.367	15.245	-1.361	1.00	0.00	H
ATOM	336	CE2	TYR	21	16.043	13.774	0.157	1.00	0.00	C
ATOM	337	HE2	TYR	21	15.591	14.755	0.141	1.00	0.00	H
ATOM	338	CD2	TYR	21	15.670	12.970	1.236	1.00	0.00	C
ATOM	339	HD2	TYR	21	14.991	13.310	2.003	1.00	0.00	H
ATOM	340	C	TYR	21	17.449	10.557	3.578	1.00	0.00	C
ATOM	341	O	TYR	21	18.534	10.754	3.036	1.00	0.00	O
ATOM	342	N	GLU	22	17.161	11.122	4.719	1.00	0.00	N
ATOM	343	H	GLU	22	16.276	10.832	5.109	1.00	0.00	H
ATOM	344	CA	GLU	22	18.005	12.090	5.477	1.00	0.00	C
ATOM	345	HA	GLU	22	18.300	12.941	4.863	1.00	0.00	H
ATOM	346	CB	GLU	22	17.243	12.648	6.671	1.00	0.00	C
ATOM	347	HB2	GLU	22	16.786	11.821	7.215	1.00	0.00	H
ATOM	348	HB3	GLU	22	16.475	13.266	6.206	1.00	0.00	H
ATOM	349	CG	GLU	22	18.075	13.547	7.549	1.00	0.00	C
ATOM	350	HG2	GLU	22	18.658	14.218	6.918	1.00	0.00	H
ATOM	351	HG3	GLU	22	18.771	12.928	8.115	1.00	0.00	H
ATOM	352	CD	GLU	22	17.119	14.475	8.396	1.00	0.00	C
ATOM	353	OE1	GLU	22	16.764	15.604	7.984	1.00	0.00	O
ATOM	354	OE2	GLU	22	16.882	14.254	9.567	1.00	0.00	O
ATOM	355	C	GLU	22	19.261	11.416	5.972	1.00	0.00	C
ATOM	356	O	GLU	22	20.383	11.910	5.879	1.00	0.00	O
ATOM	357	N	ASP	23	19.227	10.173	6.480	1.00	0.00	N
ATOM	358	H	ASP	23	18.318	9.784	6.685	1.00	0.00	H
ATOM	359	CA	ASP	23	20.376	9.498	6.958	1.00	0.00	C
ATOM	360	HA	ASP	23	20.968	10.280	7.433	1.00	0.00	H
ATOM	361	CB	ASP	23	19.905	8.472	7.931	1.00	0.00	C
ATOM	362	HB2	ASP	23	18.931	8.090	7.626	1.00	0.00	H
ATOM	363	HB3	ASP	23	19.711	9.090	8.808	1.00	0.00	H
ATOM	364	CG	ASP	23	20.833	7.254	8.201	1.00	0.00	C
ATOM	365	OD1	ASP	23	21.620	7.319	9.167	1.00	0.00	O
ATOM	366	OD2	ASP	23	20.801	6.267	7.407	1.00	0.00	O
ATOM	367	C	ASP	23	21.276	9.027	5.756	1.00	0.00	C
ATOM	368	O	ASP	23	22.537	8.984	5.957	1.00	0.00	O
ATOM	369	N	ARG	24	20.682	8.707	4.623	1.00	0.00	N
ATOM	370	H	ARG	24	19.705	8.946	4.708	1.00	0.00	H
ATOM	371	CA	ARG	24	21.404	8.401	3.403	1.00	0.00	C
ATOM	372	HA	ARG	24	22.248	7.781	3.708	1.00	0.00	H
ATOM	373	CB	ARG	24	20.552	7.674	2.263	1.00	0.00	C
ATOM	374	HB2	ARG	24	21.091	7.573	1.321	1.00	0.00	H
ATOM	375	HB3	ARG	24	19.721	8.353	2.066	1.00	0.00	H
ATOM	376	CG	ARG	24	20.086	6.238	2.519	1.00	0.00	C
ATOM	377	HG2	ARG	24	19.426	6.165	3.383	1.00	0.00	H
ATOM	378	HG3	ARG	24	21.002	5.687	2.732	1.00	0.00	H
ATOM	379	CD	ARG	24	19.493	5.516	1.296	1.00	0.00	C
ATOM	380	HD2	ARG	24	19.445	4.432	1.394	1.00	0.00	H

ATOM	381	HD3	ARG	24	20.132	5.807	0.462	1.00	0.00	H
ATOM	382	NE	ARG	24	18.048	5.933	1.161	1.00	0.00	N
ATOM	383	HE	ARG	24	17.754	6.706	1.741	1.00	0.00	H
ATOM	384	CZ	ARG	24	17.062	5.401	0.434	1.00	0.00	C
ATOM	385	NH1	ARG	24	17.250	4.503	-0.462	1.00	0.00	N
ATOM	386	HH11	ARG	24	18.165	4.145	-0.694	1.00	0.00	H
ATOM	387	HH12	ARG	24	16.389	4.239	-0.920	1.00	0.00	H
ATOM	388	NH2	ARG	24	15.782	5.609	0.534	1.00	0.00	N
ATOM	389	HH21	ARG	24	15.421	5.772	1.463	1.00	0.00	H
ATOM	390	HH22	ARG	24	15.114	5.032	0.043	1.00	0.00	H
ATOM	391	C	ARG	24	22.071	9.670	2.761	1.00	0.00	C
ATOM	392	O	ARG	24	23.262	9.551	2.383	1.00	0.00	O
ATOM	393	N	TYR	25	21.432	10.850	2.803	1.00	0.00	N
ATOM	394	H	TYR	25	20.441	10.911	2.987	1.00	0.00	H
ATOM	395	CA	TYR	25	21.965	12.130	2.403	1.00	0.00	C
ATOM	396	HA	TYR	25	22.269	12.137	1.356	1.00	0.00	H
ATOM	397	CB	TYR	25	20.854	13.147	2.498	1.00	0.00	C
ATOM	398	HB2	TYR	25	20.246	12.932	3.377	1.00	0.00	H
ATOM	399	HB3	TYR	25	20.233	12.838	1.657	1.00	0.00	H
ATOM	400	CG	TYR	25	21.276	14.581	2.405	1.00	0.00	C
ATOM	401	CD1	TYR	25	21.479	15.368	3.572	1.00	0.00	C
ATOM	402	HD1	TYR	25	21.298	14.902	4.529	1.00	0.00	H
ATOM	403	CE1	TYR	25	21.881	16.735	3.499	1.00	0.00	C
ATOM	404	HE1	TYR	25	22.044	17.279	4.417	1.00	0.00	H
ATOM	405	CZ	TYR	25	22.065	17.368	2.233	1.00	0.00	C
ATOM	406	OH	TYR	25	22.585	18.620	2.099	1.00	0.00	O
ATOM	407	HH	TYR	25	22.682	19.011	2.971	1.00	0.00	H
ATOM	408	CE2	TYR	25	21.850	16.649	1.029	1.00	0.00	C
ATOM	409	HE2	TYR	25	22.146	17.018	0.058	1.00	0.00	H
ATOM	410	CD2	TYR	25	21.433	15.282	1.162	1.00	0.00	C
ATOM	411	HD2	TYR	25	21.225	14.714	0.267	1.00	0.00	H
ATOM	412	C	TYR	25	23.178	12.525	3.249	1.00	0.00	C
ATOM	413	O	TYR	25	24.125	13.070	2.692	1.00	0.00	O
ATOM	414	N	TYR	26	23.206	12.305	4.611	1.00	0.00	N
ATOM	415	H	TYR	26	22.355	11.940	5.014	1.00	0.00	H
ATOM	416	CA	TYR	26	24.254	12.712	5.506	1.00	0.00	C
ATOM	417	HA	TYR	26	24.604	13.737	5.382	1.00	0.00	H
ATOM	418	CB	TYR	26	23.632	12.614	6.949	1.00	0.00	C
ATOM	419	HB2	TYR	26	23.428	11.581	7.229	1.00	0.00	H
ATOM	420	HB3	TYR	26	22.657	13.102	6.962	1.00	0.00	H
ATOM	421	CG	TYR	26	24.556	13.031	8.136	1.00	0.00	C
ATOM	422	CD1	TYR	26	24.436	14.329	8.661	1.00	0.00	C
ATOM	423	HD1	TYR	26	23.801	15.111	8.274	1.00	0.00	H
ATOM	424	CE1	TYR	26	25.212	14.652	9.825	1.00	0.00	C
ATOM	425	HE1	TYR	26	25.130	15.589	10.356	1.00	0.00	H
ATOM	426	CZ	TYR	26	26.136	13.721	10.378	1.00	0.00	C
ATOM	427	OH	TYR	26	26.992	14.071	11.416	1.00	0.00	O
ATOM	428	HH	TYR	26	26.969	15.021	11.549	1.00	0.00	H
ATOM	429	CE2	TYR	26	26.223	12.398	9.864	1.00	0.00	C
ATOM	430	HE2	TYR	26	26.826	11.674	10.393	1.00	0.00	H
ATOM	431	CD2	TYR	26	25.471	12.093	8.715	1.00	0.00	C
ATOM	432	HD2	TYR	26	25.481	11.078	8.347	1.00	0.00	H
ATOM	433	C	TYR	26	25.437	11.773	5.316	1.00	0.00	C
ATOM	434	O	TYR	26	26.499	12.267	4.940	1.00	0.00	O
ATOM	435	N	ARG	27	25.248	10.422	5.336	1.00	0.00	N
ATOM	436	H	ARG	27	24.295	10.145	5.525	1.00	0.00	H
ATOM	437	CA	ARG	27	26.353	9.503	5.423	1.00	0.00	C
ATOM	438	HA	ARG	27	26.827	9.729	6.378	1.00	0.00	H
ATOM	439	CB	ARG	27	25.752	8.089	5.501	1.00	0.00	C
ATOM	440	HB2	ARG	27	26.419	7.365	5.034	1.00	0.00	H
ATOM	441	HB3	ARG	27	24.789	8.161	4.996	1.00	0.00	H
ATOM	442	CG	ARG	27	25.467	7.615	6.971	1.00	0.00	C
ATOM	443	HG2	ARG	27	24.870	8.294	7.580	1.00	0.00	H

ATOM	444	HG3	ARG	27	26.422	7.521	7.487	1.00	0.00	H
ATOM	445	CD	ARG	27	24.634	6.389	6.912	1.00	0.00	C
ATOM	446	HD2	ARG	27	25.093	5.627	6.283	1.00	0.00	H
ATOM	447	HD3	ARG	27	23.759	6.798	6.405	1.00	0.00	H
ATOM	448	NE	ARG	27	24.219	5.925	8.252	1.00	0.00	N
ATOM	449	HE	ARG	27	23.283	6.232	8.477	1.00	0.00	H
ATOM	450	CZ	ARG	27	24.734	4.960	8.932	1.00	0.00	C
ATOM	451	NH1	ARG	27	25.863	4.318	8.635	1.00	0.00	N
ATOM	452	HH11	ARG	27	26.377	4.518	7.789	1.00	0.00	H
ATOM	453	HH12	ARG	27	26.116	3.529	9.213	1.00	0.00	H
ATOM	454	NH2	ARG	27	24.109	4.457	9.950	1.00	0.00	N
ATOM	455	HH21	ARG	27	24.636	3.865	10.576	1.00	0.00	H
ATOM	456	HH22	ARG	27	23.215	4.837	10.228	1.00	0.00	H
ATOM	457	C	ARG	27	27.385	9.608	4.233	1.00	0.00	C
ATOM	458	O	ARG	27	28.540	9.447	4.402	1.00	0.00	O
ATOM	459	N	GLU	28	26.842	9.885	3.084	1.00	0.00	N
ATOM	460	H	GLU	28	25.836	9.810	3.040	1.00	0.00	H
ATOM	461	CA	GLU	28	27.628	9.943	1.815	1.00	0.00	C
ATOM	462	HA	GLU	28	28.378	9.187	2.047	1.00	0.00	H
ATOM	463	CB	GLU	28	26.843	9.500	0.585	1.00	0.00	C
ATOM	464	HB2	GLU	28	26.374	8.545	0.824	1.00	0.00	H
ATOM	465	HB3	GLU	28	27.605	9.186	-0.128	1.00	0.00	H
ATOM	466	CG	GLU	28	25.878	10.561	-0.034	1.00	0.00	C
ATOM	467	HG2	GLU	28	26.449	11.380	-0.471	1.00	0.00	H
ATOM	468	HG3	GLU	28	25.279	10.864	0.825	1.00	0.00	H
ATOM	469	CD	GLU	28	24.988	10.005	-1.176	1.00	0.00	C
ATOM	470	OE1	GLU	28	24.694	8.759	-1.201	1.00	0.00	O
ATOM	471	OE2	GLU	28	24.624	10.813	-2.042	1.00	0.00	O
ATOM	472	C	GLU	28	28.340	11.265	1.606	1.00	0.00	C
ATOM	473	O	GLU	28	29.240	11.410	0.775	1.00	0.00	O
ATOM	474	N	ASN	29	27.962	12.296	2.414	1.00	0.00	N
ATOM	475	H	ASN	29	27.196	12.075	3.035	1.00	0.00	H
ATOM	476	CA	ASN	29	28.480	13.692	2.345	1.00	0.00	C
ATOM	477	HA	ASN	29	28.536	13.848	1.268	1.00	0.00	H
ATOM	478	CB	ASN	29	27.439	14.642	3.062	1.00	0.00	C
ATOM	479	HB2	ASN	29	27.217	14.536	4.124	1.00	0.00	H
ATOM	480	HB3	ASN	29	26.500	14.432	2.549	1.00	0.00	H
ATOM	481	CG	ASN	29	27.842	16.133	3.020	1.00	0.00	C
ATOM	482	OD1	ASN	29	29.018	16.428	3.069	1.00	0.00	O
ATOM	483	ND2	ASN	29	26.871	16.965	2.963	1.00	0.00	N
ATOM	484	HD21	ASN	29	27.121	17.931	2.808	1.00	0.00	H
ATOM	485	HD22	ASN	29	25.984	16.651	2.596	1.00	0.00	H
ATOM	486	C	ASN	29	29.903	13.730	2.904	1.00	0.00	C
ATOM	487	O	ASN	29	30.102	13.601	4.117	1.00	0.00	O
ATOM	488	N	MET	30	30.924	13.720	2.043	1.00	0.00	N
ATOM	489	H	MET	30	30.766	13.616	1.051	1.00	0.00	H
ATOM	490	CA	MET	30	32.353	13.535	2.449	1.00	0.00	C
ATOM	491	HA	MET	30	32.398	12.744	3.198	1.00	0.00	H
ATOM	492	CB	MET	30	33.158	13.074	1.151	1.00	0.00	C
ATOM	493	HB2	MET	30	34.171	12.788	1.435	1.00	0.00	H
ATOM	494	HB3	MET	30	33.269	14.025	0.629	1.00	0.00	H
ATOM	495	CG	MET	30	32.566	12.035	0.265	1.00	0.00	C
ATOM	496	HG2	MET	30	31.498	12.165	0.088	1.00	0.00	H
ATOM	497	HG3	MET	30	32.614	11.181	0.941	1.00	0.00	H
ATOM	498	SD	MET	30	33.314	11.784	-1.264	1.00	0.00	S
ATOM	499	CE	MET	30	32.592	10.145	-1.602	1.00	0.00	C
ATOM	500	HE1	MET	30	33.110	9.401	-0.997	1.00	0.00	H
ATOM	501	HE2	MET	30	31.516	10.036	-1.470	1.00	0.00	H
ATOM	502	HE3	MET	30	32.833	9.938	-2.645	1.00	0.00	H
ATOM	503	C	MET	30	32.936	14.819	3.062	1.00	0.00	C
ATOM	504	O	MET	30	33.901	14.749	3.910	1.00	0.00	O
ATOM	505	N	TYR	31	32.426	15.988	2.668	1.00	0.00	N
ATOM	506	H	TYR	31	31.712	16.092	1.962	1.00	0.00	H

ATOM	507	CA	TYR	31	32.913	17.287	3.191	1.00	0.00	C
ATOM	508	HA	TYR	31	33.923	17.450	2.815	1.00	0.00	H
ATOM	509	CB	TYR	31	32.032	18.462	2.601	1.00	0.00	C
ATOM	510	HB2	TYR	31	32.129	19.388	3.167	1.00	0.00	H
ATOM	511	HB3	TYR	31	31.012	18.105	2.746	1.00	0.00	H
ATOM	512	CG	TYR	31	32.266	18.753	1.109	1.00	0.00	C
ATOM	513	CD1	TYR	31	33.348	19.556	0.730	1.00	0.00	C
ATOM	514	HD1	TYR	31	33.950	20.074	1.463	1.00	0.00	H
ATOM	515	CE1	TYR	31	33.601	19.674	-0.639	1.00	0.00	C
ATOM	516	HE1	TYR	31	34.428	20.278	-0.980	1.00	0.00	H
ATOM	517	CZ	TYR	31	32.884	18.935	-1.618	1.00	0.00	C
ATOM	518	OH	TYR	31	33.202	18.997	-2.921	1.00	0.00	O
ATOM	519	HH	TYR	31	32.668	18.336	-3.367	1.00	0.00	H
ATOM	520	CE2	TYR	31	31.786	18.100	-1.171	1.00	0.00	C
ATOM	521	HE2	TYR	31	31.360	17.438	-1.910	1.00	0.00	H
ATOM	522	CD2	TYR	31	31.463	18.027	0.173	1.00	0.00	C
ATOM	523	HD2	TYR	31	30.712	17.352	0.554	1.00	0.00	H
ATOM	524	C	TYR	31	32.891	17.307	4.706	1.00	0.00	C
ATOM	525	O	TYR	31	33.794	17.889	5.318	1.00	0.00	O
ATOM	526	N	ARG	32	32.056	16.484	5.420	1.00	0.00	N
ATOM	527	H	ARG	32	31.464	15.850	4.902	1.00	0.00	H
ATOM	528	CA	ARG	32	32.041	16.363	6.880	1.00	0.00	C
ATOM	529	HA	ARG	32	31.868	17.358	7.289	1.00	0.00	H
ATOM	530	CB	ARG	32	30.959	15.375	7.198	1.00	0.00	C
ATOM	531	HB2	ARG	32	30.936	15.158	8.266	1.00	0.00	H
ATOM	532	HB3	ARG	32	31.335	14.439	6.783	1.00	0.00	H
ATOM	533	CG	ARG	32	29.537	15.710	6.826	1.00	0.00	C
ATOM	534	HG2	ARG	32	29.412	15.909	5.762	1.00	0.00	H
ATOM	535	HG3	ARG	32	29.494	16.625	7.418	1.00	0.00	H
ATOM	536	CD	ARG	32	28.497	14.602	7.184	1.00	0.00	C
ATOM	537	HD2	ARG	32	27.609	14.902	6.627	1.00	0.00	H
ATOM	538	HD3	ARG	32	28.376	14.620	8.267	1.00	0.00	H
ATOM	539	NE	ARG	32	28.952	13.274	6.756	1.00	0.00	N
ATOM	540	HE	ARG	32	28.922	13.172	5.751	1.00	0.00	H
ATOM	541	CZ	ARG	32	29.753	12.445	7.360	1.00	0.00	C
ATOM	542	NH1	ARG	32	29.988	12.432	8.639	1.00	0.00	N
ATOM	543	HH11	ARG	32	29.499	13.124	9.189	1.00	0.00	H
ATOM	544	HH12	ARG	32	30.702	11.842	9.043	1.00	0.00	H
ATOM	545	NH2	ARG	32	30.318	11.517	6.636	1.00	0.00	N
ATOM	546	HH21	ARG	32	30.140	11.484	5.642	1.00	0.00	H
ATOM	547	HH22	ARG	32	30.728	10.759	7.163	1.00	0.00	H
ATOM	548	C	ARG	32	33.361	15.898	7.458	1.00	0.00	C
ATOM	549	O	ARG	32	33.567	16.038	8.622	1.00	0.00	O
ATOM	550	N	TYR	33	34.252	15.252	6.661	1.00	0.00	N
ATOM	551	H	TYR	33	34.116	15.287	5.661	1.00	0.00	H
ATOM	552	CA	TYR	33	35.559	14.777	6.986	1.00	0.00	C
ATOM	553	HA	TYR	33	35.745	15.184	7.979	1.00	0.00	H
ATOM	554	CB	TYR	33	35.506	13.264	7.035	1.00	0.00	C
ATOM	555	HB2	TYR	33	35.051	12.849	6.135	1.00	0.00	H
ATOM	556	HB3	TYR	33	34.790	12.974	7.803	1.00	0.00	H
ATOM	557	CG	TYR	33	36.836	12.599	7.184	1.00	0.00	C
ATOM	558	CD1	TYR	33	37.455	12.418	8.458	1.00	0.00	C
ATOM	559	HD1	TYR	33	36.956	12.967	9.242	1.00	0.00	H
ATOM	560	CE1	TYR	33	38.668	11.703	8.529	1.00	0.00	C
ATOM	561	HE1	TYR	33	39.207	11.521	9.448	1.00	0.00	H
ATOM	562	CZ	TYR	33	39.247	11.283	7.351	1.00	0.00	C
ATOM	563	OH	TYR	33	40.521	10.683	7.465	1.00	0.00	O
ATOM	564	HH	TYR	33	40.800	10.261	6.649	1.00	0.00	H
ATOM	565	CE2	TYR	33	38.669	11.465	6.084	1.00	0.00	C
ATOM	566	HE2	TYR	33	39.169	11.114	5.194	1.00	0.00	H
ATOM	567	CD2	TYR	33	37.419	12.065	6.026	1.00	0.00	C
ATOM	568	HD2	TYR	33	36.889	12.047	5.085	1.00	0.00	H
ATOM	569	C	TYR	33	36.716	15.257	6.073	1.00	0.00	C

```
ATOM    570  O   TYR    33      37.780  15.632   6.648  1.00  0.00           O
ATOM    571  N   NME    34      36.532  15.227   4.742  1.00  0.00           N
ATOM    572  H   NME    34      35.626  14.861   4.485  1.00  0.00           H
ATOM    573  CH3 NME    34      37.496  15.779   3.785  1.00  0.00           C
ATOM    574 HH31 NME    34      37.261  15.543   2.748  1.00  0.00           H
ATOM    575 HH32 NME    34      37.449  16.868   3.781  1.00  0.00           H
ATOM    576 HH33 NME    34      38.487  15.354   3.948  1.00  0.00           H
TER     577      NME    34
END
```

Cluster 1:

```
ATOM      1 HH31 ACE     1      18.148  11.491  -3.707  1.00  0.00           H
ATOM      2  CH3 ACE     1      18.676  12.188  -3.056  1.00  0.00           C
ATOM      3 HH32 ACE     1      18.132  12.362  -2.127  1.00  0.00           H
ATOM      4 HH33 ACE     1      18.789  13.090  -3.657  1.00  0.00           H
ATOM      5  C   ACE     1      19.928  11.389  -2.669  1.00  0.00           C
ATOM      6  O   ACE     1      21.022  11.863  -2.917  1.00  0.00           O
ATOM      7  N   ASN     2      19.683  10.185  -2.189  1.00  0.00           N
ATOM      8  H   ASN     2      18.727   9.889  -2.327  1.00  0.00           H
ATOM      9  CA  ASN     2      20.756   9.280  -1.672  1.00  0.00           C
ATOM     10  HA  ASN     2      21.423   9.933  -1.110  1.00  0.00           H
ATOM     11  CB  ASN     2      20.090   8.246  -0.767  1.00  0.00           C
ATOM     12  HB2 ASN     2      19.600   7.427  -1.294  1.00  0.00           H
ATOM     13  HB3 ASN     2      19.270   8.762  -0.269  1.00  0.00           H
ATOM     14  CG  ASN     2      20.841   7.558   0.318  1.00  0.00           C
ATOM     15  OD1 ASN     2      20.737   7.849   1.550  1.00  0.00           O
ATOM     16  ND2 ASN     2      21.747   6.611   0.041  1.00  0.00           N
ATOM     17 HD21 ASN     2      22.220   6.095   0.769  1.00  0.00           H
ATOM     18 HD22 ASN     2      21.905   6.303  -0.908  1.00  0.00           H
ATOM     19  C   ASN     2      21.724   8.708  -2.772  1.00  0.00           C
ATOM     20  O   ASN     2      21.391   8.814  -3.902  1.00  0.00           O
ATOM     21  N   ASP     3      22.839   8.052  -2.413  1.00  0.00           N
ATOM     22  H   ASP     3      23.023   7.939  -1.426  1.00  0.00           H
ATOM     23  CA  ASP     3      23.695   7.314  -3.353  1.00  0.00           C
ATOM     24  HA  ASP     3      23.954   8.057  -4.107  1.00  0.00           H
ATOM     25  CB  ASP     3      25.092   7.025  -2.735  1.00  0.00           C
ATOM     26  HB2 ASP     3      25.004   6.589  -1.740  1.00  0.00           H
ATOM     27  HB3 ASP     3      25.467   8.046  -2.670  1.00  0.00           H
ATOM     28  CG  ASP     3      25.989   6.132  -3.543  1.00  0.00           C
ATOM     29  OD1 ASP     3      25.928   4.885  -3.473  1.00  0.00           O
ATOM     30  OD2 ASP     3      26.681   6.726  -4.443  1.00  0.00           O
ATOM     31  C   ASP     3      22.989   6.081  -3.893  1.00  0.00           C
ATOM     32  O   ASP     3      22.374   5.459  -3.007  1.00  0.00           O
ATOM     33  N   TYR     4      23.130   5.642  -5.162  1.00  0.00           N
ATOM     34  H   TYR     4      23.727   6.222  -5.736  1.00  0.00           H
ATOM     35  CA  TYR     4      22.408   4.527  -5.819  1.00  0.00           C
ATOM     36  HA  TYR     4      21.333   4.588  -5.644  1.00  0.00           H
ATOM     37  CB  TYR     4      22.848   4.379  -7.225  1.00  0.00           C
ATOM     38  HB2 TYR     4      22.422   3.489  -7.689  1.00  0.00           H
ATOM     39  HB3 TYR     4      23.935   4.317  -7.270  1.00  0.00           H
ATOM     40  CG  TYR     4      22.568   5.474  -8.237  1.00  0.00           C
ATOM     41  CD1 TYR     4      21.375   6.180  -8.091  1.00  0.00           C
ATOM     42  HD1 TYR     4      20.656   6.073  -7.292  1.00  0.00           H
ATOM     43  CE1 TYR     4      21.047   7.246  -8.978  1.00  0.00           C
ATOM     44  HE1 TYR     4      20.135   7.814  -8.871  1.00  0.00           H
ATOM     45  CZ  TYR     4      21.823   7.463 -10.134  1.00  0.00           C
ATOM     46  OH  TYR     4      21.423   8.426 -11.076  1.00  0.00           O
ATOM     47  HH  TYR     4      22.023   8.506 -11.821  1.00  0.00           H
ATOM     48  CE2 TYR     4      22.986   6.741 -10.361  1.00  0.00           C
ATOM     49  HE2 TYR     4      23.678   7.052 -11.129  1.00  0.00           H
```

ATOM	50	CD2	TYR	4	23.341	5.733	-9.422	1.00	0.00	C
ATOM	51	HD2	TYR	4	24.273	5.190	-9.475	1.00	0.00	H
ATOM	52	C	TYR	4	22.825	3.235	-5.089	1.00	0.00	C
ATOM	53	O	TYR	4	21.954	2.467	-4.663	1.00	0.00	O
ATOM	54	N	GLU	5	24.068	2.972	-4.661	1.00	0.00	N
ATOM	55	H	GLU	5	24.716	3.747	-4.665	1.00	0.00	H
ATOM	56	CA	GLU	5	24.444	1.772	-4.084	1.00	0.00	C
ATOM	57	HA	GLU	5	23.895	0.963	-4.566	1.00	0.00	H
ATOM	58	CB	GLU	5	25.999	1.481	-4.276	1.00	0.00	C
ATOM	59	HB2	GLU	5	26.301	0.740	-3.535	1.00	0.00	H
ATOM	60	HB3	GLU	5	26.461	2.434	-4.022	1.00	0.00	H
ATOM	61	CG	GLU	5	26.467	1.026	-5.620	1.00	0.00	C
ATOM	62	HG2	GLU	5	26.223	1.771	-6.377	1.00	0.00	H
ATOM	63	HG3	GLU	5	26.051	0.027	-5.752	1.00	0.00	H
ATOM	64	CD	GLU	5	27.976	0.956	-5.638	1.00	0.00	C
ATOM	65	OE1	GLU	5	28.713	1.985	-5.855	1.00	0.00	O
ATOM	66	OE2	GLU	5	28.447	-0.200	-5.452	1.00	0.00	O
ATOM	67	C	GLU	5	24.134	1.730	-2.612	1.00	0.00	C
ATOM	68	O	GLU	5	23.796	0.635	-2.132	1.00	0.00	O
ATOM	69	N	ASP	6	24.252	2.801	-1.830	1.00	0.00	N
ATOM	70	H	ASP	6	24.667	3.657	-2.171	1.00	0.00	H
ATOM	71	CA	ASP	6	23.803	2.909	-0.487	1.00	0.00	C
ATOM	72	HA	ASP	6	24.248	2.095	0.086	1.00	0.00	H
ATOM	73	CB	ASP	6	24.368	4.183	0.183	1.00	0.00	C
ATOM	74	HB2	ASP	6	24.036	5.079	-0.341	1.00	0.00	H
ATOM	75	HB3	ASP	6	25.423	3.961	0.022	1.00	0.00	H
ATOM	76	CG	ASP	6	24.047	4.257	1.715	1.00	0.00	C
ATOM	77	OD1	ASP	6	24.686	3.474	2.491	1.00	0.00	O
ATOM	78	OD2	ASP	6	23.129	5.062	2.098	1.00	0.00	O
ATOM	79	C	ASP	6	22.262	2.777	-0.369	1.00	0.00	C
ATOM	80	O	ASP	6	21.846	2.476	0.735	1.00	0.00	O
ATOM	81	N	ARG	7	21.437	3.093	-1.411	1.00	0.00	N
ATOM	82	H	ARG	7	21.830	3.448	-2.271	1.00	0.00	H
ATOM	83	CA	ARG	7	19.955	3.089	-1.154	1.00	0.00	C
ATOM	84	HA	ARG	7	19.906	3.599	-0.192	1.00	0.00	H
ATOM	85	CB	ARG	7	19.172	3.937	-2.193	1.00	0.00	C
ATOM	86	HB2	ARG	7	19.519	3.735	-3.206	1.00	0.00	H
ATOM	87	HB3	ARG	7	19.574	4.943	-2.074	1.00	0.00	H
ATOM	88	CG	ARG	7	17.647	4.048	-2.003	1.00	0.00	C
ATOM	89	HG2	ARG	7	17.218	3.064	-2.197	1.00	0.00	H
ATOM	90	HG3	ARG	7	17.324	4.651	-2.852	1.00	0.00	H
ATOM	91	CD	ARG	7	17.140	4.708	-0.637	1.00	0.00	C
ATOM	92	HD2	ARG	7	16.054	4.778	-0.679	1.00	0.00	H
ATOM	93	HD3	ARG	7	17.512	5.729	-0.720	1.00	0.00	H
ATOM	94	NE	ARG	7	17.466	3.966	0.627	1.00	0.00	N
ATOM	95	HE	ARG	7	17.208	2.991	0.568	1.00	0.00	H
ATOM	96	CZ	ARG	7	18.087	4.396	1.714	1.00	0.00	C
ATOM	97	NH1	ARG	7	18.246	5.656	1.997	1.00	0.00	N
ATOM	98	HH11	ARG	7	18.017	6.433	1.394	1.00	0.00	H
ATOM	99	HH12	ARG	7	18.796	5.850	2.822	1.00	0.00	H
ATOM	100	NH2	ARG	7	18.667	3.612	2.526	1.00	0.00	N
ATOM	101	HH21	ARG	7	18.751	2.611	2.420	1.00	0.00	H
ATOM	102	HH22	ARG	7	19.149	4.110	3.261	1.00	0.00	H
ATOM	103	C	ARG	7	19.408	1.635	-1.004	1.00	0.00	C
ATOM	104	O	ARG	7	18.353	1.451	-0.415	1.00	0.00	O
ATOM	105	N	TYR	8	20.196	0.648	-1.509	1.00	0.00	N
ATOM	106	H	TYR	8	21.016	0.902	-2.041	1.00	0.00	H
ATOM	107	CA	TYR	8	19.873	-0.814	-1.403	1.00	0.00	C
ATOM	108	HA	TYR	8	18.888	-0.850	-1.869	1.00	0.00	H
ATOM	109	CB	TYR	8	20.760	-1.600	-2.293	1.00	0.00	C
ATOM	110	HB2	TYR	8	21.699	-1.855	-1.801	1.00	0.00	H
ATOM	111	HB3	TYR	8	20.804	-0.970	-3.181	1.00	0.00	H
ATOM	112	CG	TYR	8	20.029	-2.943	-2.596	1.00	0.00	C

ATOM	113	CD1	TYR	8	20.651	-4.156	-2.124	1.00	0.00	C
ATOM	114	HD1	TYR	8	21.542	-4.036	-1.525	1.00	0.00	H
ATOM	115	CE1	TYR	8	20.129	-5.378	-2.496	1.00	0.00	C
ATOM	116	HE1	TYR	8	20.686	-6.290	-2.335	1.00	0.00	H
ATOM	117	CZ	TYR	8	18.924	-5.377	-3.166	1.00	0.00	C
ATOM	118	OH	TYR	8	18.372	-6.566	-3.591	1.00	0.00	O
ATOM	119	HH	TYR	8	18.871	-7.284	-3.196	1.00	0.00	H
ATOM	120	CE2	TYR	8	18.311	-4.227	-3.559	1.00	0.00	C
ATOM	121	HE2	TYR	8	17.419	-4.307	-4.163	1.00	0.00	H
ATOM	122	CD2	TYR	8	18.863	-2.952	-3.320	1.00	0.00	C
ATOM	123	HD2	TYR	8	18.450	-2.077	-3.800	1.00	0.00	H
ATOM	124	C	TYR	8	19.760	-1.359	0.053	1.00	0.00	C
ATOM	125	O	TYR	8	19.148	-2.418	0.287	1.00	0.00	O
ATOM	126	N	TYR	9	20.412	-0.656	1.029	1.00	0.00	N
ATOM	127	H	TYR	9	20.840	0.228	0.793	1.00	0.00	H
ATOM	128	CA	TYR	9	20.366	-1.024	2.475	1.00	0.00	C
ATOM	129	HA	TYR	9	20.141	-2.078	2.641	1.00	0.00	H
ATOM	130	CB	TYR	9	21.756	-0.771	3.159	1.00	0.00	C
ATOM	131	HB2	TYR	9	21.863	-1.257	4.129	1.00	0.00	H
ATOM	132	HB3	TYR	9	21.786	0.315	3.244	1.00	0.00	H
ATOM	133	CG	TYR	9	22.978	-1.293	2.406	1.00	0.00	C
ATOM	134	CD1	TYR	9	23.665	-0.513	1.468	1.00	0.00	C
ATOM	135	HD1	TYR	9	23.462	0.544	1.379	1.00	0.00	H
ATOM	136	CE1	TYR	9	24.724	-1.006	0.724	1.00	0.00	C
ATOM	137	HE1	TYR	9	25.298	-0.413	0.028	1.00	0.00	H
ATOM	138	CZ	TYR	9	25.016	-2.371	0.758	1.00	0.00	C
ATOM	139	OH	TYR	9	26.005	-2.972	-0.052	1.00	0.00	O
ATOM	140	HH	TYR	9	26.219	-3.895	0.099	1.00	0.00	H
ATOM	141	CE2	TYR	9	24.324	-3.195	1.687	1.00	0.00	C
ATOM	142	HE2	TYR	9	24.481	-4.263	1.653	1.00	0.00	H
ATOM	143	CD2	TYR	9	23.291	-2.645	2.484	1.00	0.00	C
ATOM	144	HD2	TYR	9	22.738	-3.313	3.129	1.00	0.00	H
ATOM	145	C	TYR	9	19.292	-0.290	3.275	1.00	0.00	C
ATOM	146	O	TYR	9	18.738	0.743	2.809	1.00	0.00	O
ATOM	147	N	ARG	10	18.958	-0.791	4.466	1.00	0.00	N
ATOM	148	H	ARG	10	19.453	-1.572	4.872	1.00	0.00	H
ATOM	149	CA	ARG	10	17.950	-0.150	5.399	1.00	0.00	C
ATOM	150	HA	ARG	10	17.177	0.353	4.818	1.00	0.00	H
ATOM	151	CB	ARG	10	17.171	-1.311	6.199	1.00	0.00	C
ATOM	152	HB2	ARG	10	16.679	-0.842	7.052	1.00	0.00	H
ATOM	153	HB3	ARG	10	17.928	-1.976	6.613	1.00	0.00	H
ATOM	154	CG	ARG	10	16.200	-2.130	5.371	1.00	0.00	C
ATOM	155	HG2	ARG	10	16.118	-3.185	5.633	1.00	0.00	H
ATOM	156	HG3	ARG	10	16.617	-2.195	4.366	1.00	0.00	H
ATOM	157	CD	ARG	10	14.732	-1.583	5.109	1.00	0.00	C
ATOM	158	HD2	ARG	10	14.060	-2.290	4.622	1.00	0.00	H
ATOM	159	HD3	ARG	10	14.903	-0.722	4.462	1.00	0.00	H
ATOM	160	NE	ARG	10	14.054	-1.223	6.294	1.00	0.00	N
ATOM	161	HE	ARG	10	14.231	-0.326	6.723	1.00	0.00	H
ATOM	162	CZ	ARG	10	13.072	-1.948	6.883	1.00	0.00	C
ATOM	163	NH1	ARG	10	12.627	-3.062	6.423	1.00	0.00	N
ATOM	164	HH11	ARG	10	13.039	-3.346	5.546	1.00	0.00	H
ATOM	165	HH12	ARG	10	11.942	-3.569	6.966	1.00	0.00	H
ATOM	166	NH2	ARG	10	12.443	-1.451	7.931	1.00	0.00	N
ATOM	167	HH21	ARG	10	12.739	-0.540	8.250	1.00	0.00	H
ATOM	168	HH22	ARG	10	11.514	-1.730	8.212	1.00	0.00	H
ATOM	169	C	ARG	10	18.580	0.851	6.373	1.00	0.00	C
ATOM	170	O	ARG	10	19.802	1.030	6.488	1.00	0.00	O
ATOM	171	N	GLU	11	17.691	1.588	7.009	1.00	0.00	N
ATOM	172	H	GLU	11	16.764	1.204	6.893	1.00	0.00	H
ATOM	173	CA	GLU	11	17.935	2.598	8.094	1.00	0.00	C
ATOM	174	HA	GLU	11	18.688	3.205	7.592	1.00	0.00	H
ATOM	175	CB	GLU	11	16.660	3.423	8.204	1.00	0.00	C

ATOM	176	HB2	GLU	11	16.293	3.847	7.269	1.00	0.00	H
ATOM	177	HB3	GLU	11	16.982	4.246	8.841	1.00	0.00	H
ATOM	178	CG	GLU	11	15.426	2.908	8.913	1.00	0.00	C
ATOM	179	HG2	GLU	11	14.740	3.751	8.992	1.00	0.00	H
ATOM	180	HG3	GLU	11	15.759	2.675	9.925	1.00	0.00	H
ATOM	181	CD	GLU	11	14.668	1.682	8.352	1.00	0.00	C
ATOM	182	OE1	GLU	11	14.821	1.340	7.152	1.00	0.00	O
ATOM	183	OE2	GLU	11	13.957	1.022	9.151	1.00	0.00	O
ATOM	184	C	GLU	11	18.566	2.161	9.364	1.00	0.00	C
ATOM	185	O	GLU	11	19.257	2.938	10.021	1.00	0.00	O
ATOM	186	N	ASN	12	18.328	0.850	9.681	1.00	0.00	N
ATOM	187	H	ASN	12	17.818	0.230	9.069	1.00	0.00	H
ATOM	188	CA	ASN	12	18.790	0.230	10.967	1.00	0.00	C
ATOM	189	HA	ASN	12	19.005	1.019	11.688	1.00	0.00	H
ATOM	190	CB	ASN	12	17.625	-0.650	11.450	1.00	0.00	C
ATOM	191	HB2	ASN	12	17.585	-1.643	11.001	1.00	0.00	H
ATOM	192	HB3	ASN	12	16.697	-0.198	11.100	1.00	0.00	H
ATOM	193	CG	ASN	12	17.689	-0.963	12.939	1.00	0.00	C
ATOM	194	OD1	ASN	12	18.316	-0.324	13.779	1.00	0.00	O
ATOM	195	ND2	ASN	12	17.028	-2.018	13.414	1.00	0.00	N
ATOM	196	HD21	ASN	12	17.267	-2.179	14.381	1.00	0.00	H
ATOM	197	HD22	ASN	12	16.438	-2.680	12.930	1.00	0.00	H
ATOM	198	C	ASN	12	20.067	-0.689	10.834	1.00	0.00	C
ATOM	199	O	ASN	12	20.288	-1.548	11.682	1.00	0.00	O
ATOM	200	N	MET	13	20.615	-0.662	9.631	1.00	0.00	N
ATOM	201	H	MET	13	20.234	0.022	8.993	1.00	0.00	H
ATOM	202	CA	MET	13	21.781	-1.418	9.178	1.00	0.00	C
ATOM	203	HA	MET	13	21.986	-2.092	10.010	1.00	0.00	H
ATOM	204	CB	MET	13	21.350	-2.273	7.890	1.00	0.00	C
ATOM	205	HB2	MET	13	21.271	-1.654	6.996	1.00	0.00	H
ATOM	206	HB3	MET	13	20.369	-2.683	8.131	1.00	0.00	H
ATOM	207	CG	MET	13	22.176	-3.468	7.491	1.00	0.00	C
ATOM	208	HG2	MET	13	22.379	-4.117	8.343	1.00	0.00	H
ATOM	209	HG3	MET	13	23.153	-3.031	7.283	1.00	0.00	H
ATOM	210	SD	MET	13	21.769	-4.542	6.158	1.00	0.00	S
ATOM	211	CE	MET	13	23.439	-4.786	5.525	1.00	0.00	C
ATOM	212	HE1	MET	13	23.932	-5.279	6.362	1.00	0.00	H
ATOM	213	HE2	MET	13	23.426	-5.381	4.612	1.00	0.00	H
ATOM	214	HE3	MET	13	23.934	-3.853	5.255	1.00	0.00	H
ATOM	215	C	MET	13	22.975	-0.500	8.875	1.00	0.00	C
ATOM	216	O	MET	13	22.855	0.553	8.271	1.00	0.00	O
ATOM	217	N	TYR	14	24.123	-0.881	9.418	1.00	0.00	N
ATOM	218	H	TYR	14	24.285	-1.830	9.722	1.00	0.00	H
ATOM	219	CA	TYR	14	25.368	-0.159	9.046	1.00	0.00	C
ATOM	220	HA	TYR	14	25.111	0.883	9.236	1.00	0.00	H
ATOM	221	CB	TYR	14	26.405	-0.557	10.152	1.00	0.00	C
ATOM	222	HB2	TYR	14	26.808	-1.562	10.027	1.00	0.00	H
ATOM	223	HB3	TYR	14	25.829	-0.498	11.075	1.00	0.00	H
ATOM	224	CG	TYR	14	27.693	0.297	10.089	1.00	0.00	C
ATOM	225	CD1	TYR	14	27.667	1.684	10.239	1.00	0.00	C
ATOM	226	HD1	TYR	14	26.673	2.091	10.352	1.00	0.00	H
ATOM	227	CE1	TYR	14	28.809	2.454	10.045	1.00	0.00	C
ATOM	228	HE1	TYR	14	28.724	3.528	10.113	1.00	0.00	H
ATOM	229	CZ	TYR	14	30.140	1.836	9.952	1.00	0.00	C
ATOM	230	OH	TYR	14	31.314	2.563	10.185	1.00	0.00	O
ATOM	231	HH	TYR	14	31.987	1.934	10.455	1.00	0.00	H
ATOM	232	CE2	TYR	14	30.101	0.473	9.697	1.00	0.00	C
ATOM	233	HE2	TYR	14	31.025	-0.038	9.468	1.00	0.00	H
ATOM	234	CD2	TYR	14	28.946	-0.286	9.721	1.00	0.00	C
ATOM	235	HD2	TYR	14	29.021	-1.354	9.577	1.00	0.00	H
ATOM	236	C	TYR	14	26.036	-0.484	7.721	1.00	0.00	C
ATOM	237	O	TYR	14	25.909	-1.635	7.239	1.00	0.00	O
ATOM	238	N	ARG	15	26.639	0.518	7.096	1.00	0.00	N

ATOM	239	H	ARG	15	26.704	1.458	7.460	1.00	0.00	H
ATOM	240	CA	ARG	15	27.349	0.350	5.843	1.00	0.00	C
ATOM	241	HA	ARG	15	27.384	-0.713	5.604	1.00	0.00	H
ATOM	242	CB	ARG	15	26.562	0.959	4.666	1.00	0.00	C
ATOM	243	HB2	ARG	15	27.000	0.651	3.717	1.00	0.00	H
ATOM	244	HB3	ARG	15	26.529	2.035	4.839	1.00	0.00	H
ATOM	245	CG	ARG	15	25.161	0.391	4.506	1.00	0.00	C
ATOM	246	HG2	ARG	15	25.162	-0.687	4.670	1.00	0.00	H
ATOM	247	HG3	ARG	15	25.043	0.471	3.425	1.00	0.00	H
ATOM	248	CD	ARG	15	24.048	0.948	5.303	1.00	0.00	C
ATOM	249	HD2	ARG	15	24.305	1.170	6.339	1.00	0.00	H
ATOM	250	HD3	ARG	15	23.330	0.129	5.333	1.00	0.00	H
ATOM	251	NE	ARG	15	23.545	2.181	4.672	1.00	0.00	N
ATOM	252	HE	ARG	15	23.995	2.497	3.825	1.00	0.00	H
ATOM	253	CZ	ARG	15	22.596	2.914	5.092	1.00	0.00	C
ATOM	254	NH1	ARG	15	22.115	2.773	6.302	1.00	0.00	N
ATOM	255	HH11	ARG	15	22.432	2.064	6.948	1.00	0.00	H
ATOM	256	HH12	ARG	15	21.404	3.440	6.563	1.00	0.00	H
ATOM	257	NH2	ARG	15	22.157	3.851	4.375	1.00	0.00	N
ATOM	258	HH21	ARG	15	22.732	4.124	3.591	1.00	0.00	H
ATOM	259	HH22	ARG	15	21.461	4.440	4.811	1.00	0.00	H
ATOM	260	C	ARG	15	28.786	0.903	5.932	1.00	0.00	C
ATOM	261	O	ARG	15	29.121	2.033	5.649	1.00	0.00	O
ATOM	262	N	TYR	16	29.663	-0.056	6.149	1.00	0.00	N
ATOM	263	H	TYR	16	29.443	-1.002	6.428	1.00	0.00	H
ATOM	264	CA	TYR	16	31.128	0.271	6.232	1.00	0.00	C
ATOM	265	HA	TYR	16	31.277	0.913	7.100	1.00	0.00	H
ATOM	266	CB	TYR	16	31.894	-1.034	6.590	1.00	0.00	C
ATOM	267	HB2	TYR	16	31.626	-1.848	5.916	1.00	0.00	H
ATOM	268	HB3	TYR	16	31.447	-1.355	7.531	1.00	0.00	H
ATOM	269	CG	TYR	16	33.412	-0.814	6.764	1.00	0.00	C
ATOM	270	CD1	TYR	16	34.300	-1.057	5.662	1.00	0.00	C
ATOM	271	HD1	TYR	16	33.949	-1.632	4.818	1.00	0.00	H
ATOM	272	CE1	TYR	16	35.641	-0.599	5.765	1.00	0.00	C
ATOM	273	HE1	TYR	16	36.321	-0.979	5.017	1.00	0.00	H
ATOM	274	CZ	TYR	16	36.106	0.165	6.906	1.00	0.00	C
ATOM	275	OH	TYR	16	37.368	0.603	6.908	1.00	0.00	O
ATOM	276	HH	TYR	16	37.831	0.118	6.221	1.00	0.00	H
ATOM	277	CE2	TYR	16	35.232	0.392	7.978	1.00	0.00	C
ATOM	278	HE2	TYR	16	35.628	0.951	8.814	1.00	0.00	H
ATOM	279	CD2	TYR	16	33.865	-0.107	7.923	1.00	0.00	C
ATOM	280	HD2	TYR	16	33.271	0.144	8.789	1.00	0.00	H
ATOM	281	C	TYR	16	31.662	0.828	4.908	1.00	0.00	C
ATOM	282	O	TYR	16	31.360	0.219	3.856	1.00	0.00	O
ATOM	283	N	NME	17	32.440	1.920	5.009	1.00	0.00	N
ATOM	284	H	NME	17	32.409	2.445	5.872	1.00	0.00	H
ATOM	285	CH3	NME	17	33.163	2.499	3.940	1.00	0.00	C
ATOM	286	HH31	NME	17	33.476	1.800	3.164	1.00	0.00	H
ATOM	287	HH32	NME	17	32.474	3.223	3.506	1.00	0.00	H
ATOM	288	HH33	NME	17	34.024	3.047	4.322	1.00	0.00	H
TER	289		NME	17						
ATOM	289	HH31	ACE	18	8.701	5.958	4.524	1.00	0.00	H
ATOM	290	CH3	ACE	18	9.532	6.652	4.404	1.00	0.00	C
ATOM	291	HH32	ACE	18	9.221	7.678	4.603	1.00	0.00	H
ATOM	292	HH33	ACE	18	9.932	6.684	3.390	1.00	0.00	H
ATOM	293	C	ACE	18	10.651	6.324	5.315	1.00	0.00	C
ATOM	294	O	ACE	18	10.975	7.083	6.200	1.00	0.00	O
ATOM	295	N	ASN	19	11.380	5.271	5.093	1.00	0.00	N
ATOM	296	H	ASN	19	10.949	4.578	4.497	1.00	0.00	H
ATOM	297	CA	ASN	19	12.704	4.963	5.688	1.00	0.00	C
ATOM	298	HA	ASN	19	12.677	5.170	6.758	1.00	0.00	H
ATOM	299	CB	ASN	19	13.016	3.432	5.629	1.00	0.00	C
ATOM	300	HB2	ASN	19	12.161	2.757	5.601	1.00	0.00	H

ATOM	301	HB3	ASN	19	13.579	3.347	6.558	1.00	0.00	H
ATOM	302	CG	ASN	19	13.735	3.053	4.355	1.00	0.00	C
ATOM	303	OD1	ASN	19	13.257	3.278	3.257	1.00	0.00	O
ATOM	304	ND2	ASN	19	14.782	2.253	4.374	1.00	0.00	N
ATOM	305	HD21	ASN	19	15.251	2.007	3.514	1.00	0.00	H
ATOM	306	HD22	ASN	19	14.956	1.745	5.230	1.00	0.00	H
ATOM	307	C	ASN	19	13.908	5.826	5.224	1.00	0.00	C
ATOM	308	O	ASN	19	14.837	5.940	5.991	1.00	0.00	O
ATOM	309	N	ASP	20	13.974	6.294	3.962	1.00	0.00	N
ATOM	310	H	ASP	20	13.340	5.877	3.296	1.00	0.00	H
ATOM	311	CA	ASP	20	15.137	7.020	3.398	1.00	0.00	C
ATOM	312	HA	ASP	20	16.054	6.447	3.534	1.00	0.00	H
ATOM	313	CB	ASP	20	14.893	7.057	1.907	1.00	0.00	C
ATOM	314	HB2	ASP	20	13.856	7.378	1.813	1.00	0.00	H
ATOM	315	HB3	ASP	20	15.117	6.076	1.489	1.00	0.00	H
ATOM	316	CG	ASP	20	15.822	8.068	1.093	1.00	0.00	C
ATOM	317	OD1	ASP	20	16.960	7.727	0.749	1.00	0.00	O
ATOM	318	OD2	ASP	20	15.299	9.113	0.591	1.00	0.00	O
ATOM	319	C	ASP	20	15.382	8.478	3.917	1.00	0.00	C
ATOM	320	O	ASP	20	16.556	8.879	4.009	1.00	0.00	O
ATOM	321	N	TYR	21	14.315	9.242	4.286	1.00	0.00	N
ATOM	322	H	TYR	21	13.431	8.935	3.907	1.00	0.00	H
ATOM	323	CA	TYR	21	14.521	10.495	4.983	1.00	0.00	C
ATOM	324	HA	TYR	21	14.963	11.267	4.354	1.00	0.00	H
ATOM	325	CB	TYR	21	13.083	11.144	5.292	1.00	0.00	C
ATOM	326	HB2	TYR	21	12.460	10.424	5.823	1.00	0.00	H
ATOM	327	HB3	TYR	21	12.751	11.426	4.293	1.00	0.00	H
ATOM	328	CG	TYR	21	12.940	12.396	6.207	1.00	0.00	C
ATOM	329	CD1	TYR	21	12.954	12.259	7.626	1.00	0.00	C
ATOM	330	HD1	TYR	21	12.899	11.249	8.005	1.00	0.00	H
ATOM	331	CE1	TYR	21	13.018	13.322	8.454	1.00	0.00	C
ATOM	332	HE1	TYR	21	13.039	13.184	9.525	1.00	0.00	H
ATOM	333	CZ	TYR	21	12.953	14.577	7.895	1.00	0.00	C
ATOM	334	OH	TYR	21	12.863	15.635	8.783	1.00	0.00	O
ATOM	335	HH	TYR	21	12.613	15.364	9.670	1.00	0.00	H
ATOM	336	CE2	TYR	21	12.946	14.763	6.444	1.00	0.00	C
ATOM	337	HE2	TYR	21	12.763	15.740	6.022	1.00	0.00	H
ATOM	338	CD2	TYR	21	12.955	13.621	5.654	1.00	0.00	C
ATOM	339	HD2	TYR	21	12.857	13.826	4.598	1.00	0.00	H
ATOM	340	C	TYR	21	15.291	10.316	6.265	1.00	0.00	C
ATOM	341	O	TYR	21	16.173	11.148	6.529	1.00	0.00	O
ATOM	342	N	GLU	22	14.997	9.294	7.070	1.00	0.00	N
ATOM	343	H	GLU	22	14.337	8.618	6.714	1.00	0.00	H
ATOM	344	CA	GLU	22	15.599	9.139	8.356	1.00	0.00	C
ATOM	345	HA	GLU	22	15.565	10.136	8.795	1.00	0.00	H
ATOM	346	CB	GLU	22	14.639	8.327	9.247	1.00	0.00	C
ATOM	347	HB2	GLU	22	14.534	7.332	8.813	1.00	0.00	H
ATOM	348	HB3	GLU	22	13.689	8.857	9.188	1.00	0.00	H
ATOM	349	CG	GLU	22	15.201	8.249	10.744	1.00	0.00	C
ATOM	350	HG2	GLU	22	15.305	9.261	11.134	1.00	0.00	H
ATOM	351	HG3	GLU	22	16.191	7.796	10.704	1.00	0.00	H
ATOM	352	CD	GLU	22	14.271	7.424	11.682	1.00	0.00	C
ATOM	353	OE1	GLU	22	13.151	7.920	12.054	1.00	0.00	O
ATOM	354	OE2	GLU	22	14.594	6.282	11.912	1.00	0.00	O
ATOM	355	C	GLU	22	17.054	8.575	8.259	1.00	0.00	C
ATOM	356	O	GLU	22	17.856	8.681	9.143	1.00	0.00	O
ATOM	357	N	ASP	23	17.356	8.083	7.054	1.00	0.00	N
ATOM	358	H	ASP	23	16.532	8.022	6.473	1.00	0.00	H
ATOM	359	CA	ASP	23	18.604	7.483	6.618	1.00	0.00	C
ATOM	360	HA	ASP	23	19.202	7.280	7.507	1.00	0.00	H
ATOM	361	CB	ASP	23	18.365	6.216	5.866	1.00	0.00	C
ATOM	362	HB2	ASP	23	17.685	6.316	5.020	1.00	0.00	H
ATOM	363	HB3	ASP	23	17.898	5.654	6.673	1.00	0.00	H

ATOM	364	CG	ASP	23	19.593	5.448	5.536	1.00	0.00	C
ATOM	365	OD1	ASP	23	20.350	5.033	6.471	1.00	0.00	O
ATOM	366	OD2	ASP	23	19.924	5.345	4.340	1.00	0.00	O
ATOM	367	C	ASP	23	19.555	8.453	5.838	1.00	0.00	C
ATOM	368	O	ASP	23	20.732	8.123	5.630	1.00	0.00	O
ATOM	369	N	ARG	24	19.039	9.647	5.498	1.00	0.00	N
ATOM	370	H	ARG	24	18.074	9.876	5.690	1.00	0.00	H
ATOM	371	CA	ARG	24	19.691	10.708	4.617	1.00	0.00	C
ATOM	372	HA	ARG	24	19.934	10.230	3.668	1.00	0.00	H
ATOM	373	CB	ARG	24	18.687	11.767	4.101	1.00	0.00	C
ATOM	374	HB2	ARG	24	18.671	12.533	4.877	1.00	0.00	H
ATOM	375	HB3	ARG	24	17.768	11.184	4.055	1.00	0.00	H
ATOM	376	CG	ARG	24	19.052	12.424	2.701	1.00	0.00	C
ATOM	377	HG2	ARG	24	19.541	11.664	2.092	1.00	0.00	H
ATOM	378	HG3	ARG	24	19.750	13.153	3.112	1.00	0.00	H
ATOM	379	CD	ARG	24	17.873	13.149	2.101	1.00	0.00	C
ATOM	380	HD2	ARG	24	18.169	13.672	1.193	1.00	0.00	H
ATOM	381	HD3	ARG	24	17.522	13.858	2.851	1.00	0.00	H
ATOM	382	NE	ARG	24	16.811	12.220	1.632	1.00	0.00	N
ATOM	383	HE	ARG	24	17.061	11.274	1.381	1.00	0.00	H
ATOM	384	CZ	ARG	24	15.624	12.623	1.277	1.00	0.00	C
ATOM	385	NH1	ARG	24	15.150	13.832	1.559	1.00	0.00	N
ATOM	386	HH11	ARG	24	15.805	14.424	2.050	1.00	0.00	H
ATOM	387	HH12	ARG	24	14.259	14.213	1.272	1.00	0.00	H
ATOM	388	NH2	ARG	24	14.911	11.829	0.542	1.00	0.00	N
ATOM	389	HH21	ARG	24	15.211	10.889	0.324	1.00	0.00	H
ATOM	390	HH22	ARG	24	13.971	12.065	0.258	1.00	0.00	H
ATOM	391	C	ARG	24	21.043	11.384	5.026	1.00	0.00	C
ATOM	392	O	ARG	24	21.684	11.992	4.162	1.00	0.00	O
ATOM	393	N	TYR	25	21.485	11.215	6.267	1.00	0.00	N
ATOM	394	H	TYR	25	20.976	10.619	6.905	1.00	0.00	H
ATOM	395	CA	TYR	25	22.830	11.651	6.719	1.00	0.00	C
ATOM	396	HA	TYR	25	22.772	12.711	6.473	1.00	0.00	H
ATOM	397	CB	TYR	25	23.035	11.461	8.220	1.00	0.00	C
ATOM	398	HB2	TYR	25	22.330	12.094	8.761	1.00	0.00	H
ATOM	399	HB3	TYR	25	24.035	11.865	8.378	1.00	0.00	H
ATOM	400	CG	TYR	25	23.002	10.037	8.683	1.00	0.00	C
ATOM	401	CD1	TYR	25	21.647	9.556	8.963	1.00	0.00	C
ATOM	402	HD1	TYR	25	20.770	10.179	9.066	1.00	0.00	H
ATOM	403	CE1	TYR	25	21.484	8.182	9.405	1.00	0.00	C
ATOM	404	HE1	TYR	25	20.533	7.747	9.672	1.00	0.00	H
ATOM	405	CZ	TYR	25	22.635	7.290	9.466	1.00	0.00	C
ATOM	406	OH	TYR	25	22.453	5.994	9.820	1.00	0.00	O
ATOM	407	HH	TYR	25	21.588	5.825	10.201	1.00	0.00	H
ATOM	408	CE2	TYR	25	23.915	7.749	9.101	1.00	0.00	C
ATOM	409	HE2	TYR	25	24.760	7.077	9.153	1.00	0.00	H
ATOM	410	CD2	TYR	25	24.086	9.145	8.795	1.00	0.00	C
ATOM	411	HD2	TYR	25	25.105	9.463	8.633	1.00	0.00	H
ATOM	412	C	TYR	25	23.847	10.916	5.883	1.00	0.00	C
ATOM	413	O	TYR	25	23.613	9.876	5.228	1.00	0.00	O
ATOM	414	N	TYR	26	25.044	11.528	5.840	1.00	0.00	N
ATOM	415	H	TYR	26	25.115	12.382	6.375	1.00	0.00	H
ATOM	416	CA	TYR	26	26.219	11.163	5.050	1.00	0.00	C
ATOM	417	HA	TYR	26	25.834	10.869	4.074	1.00	0.00	H
ATOM	418	CB	TYR	26	27.064	12.380	4.842	1.00	0.00	C
ATOM	419	HB2	TYR	26	27.603	12.778	5.702	1.00	0.00	H
ATOM	420	HB3	TYR	26	26.378	13.152	4.494	1.00	0.00	H
ATOM	421	CG	TYR	26	28.147	12.226	3.795	1.00	0.00	C
ATOM	422	CD1	TYR	26	29.473	11.719	4.142	1.00	0.00	C
ATOM	423	HD1	TYR	26	29.665	11.358	5.142	1.00	0.00	H
ATOM	424	CE1	TYR	26	30.480	11.505	3.154	1.00	0.00	C
ATOM	425	HE1	TYR	26	31.319	10.873	3.407	1.00	0.00	H
ATOM	426	CZ	TYR	26	30.146	11.797	1.782	1.00	0.00	C

ATOM	427	OH	TYR	26	31.042	11.580	0.784	1.00	0.00	O
ATOM	428	HH	TYR	26	30.799	11.906	-0.085	1.00	0.00	H
ATOM	429	CE2	TYR	26	28.901	12.292	1.426	1.00	0.00	C
ATOM	430	HE2	TYR	26	28.722	12.618	0.412	1.00	0.00	H
ATOM	431	CD2	TYR	26	27.935	12.491	2.436	1.00	0.00	C
ATOM	432	HD2	TYR	26	26.985	12.925	2.161	1.00	0.00	H
ATOM	433	C	TYR	26	26.919	9.926	5.540	1.00	0.00	C
ATOM	434	O	TYR	26	26.816	9.686	6.750	1.00	0.00	O
ATOM	435	N	ARG	27	27.661	9.248	4.644	1.00	0.00	N
ATOM	436	H	ARG	27	27.774	9.649	3.724	1.00	0.00	H
ATOM	437	CA	ARG	27	28.415	8.010	4.916	1.00	0.00	C
ATOM	438	HA	ARG	27	28.776	8.164	5.933	1.00	0.00	H
ATOM	439	CB	ARG	27	27.599	6.707	4.855	1.00	0.00	C
ATOM	440	HB2	ARG	27	28.350	5.924	4.745	1.00	0.00	H
ATOM	441	HB3	ARG	27	27.001	6.805	3.949	1.00	0.00	H
ATOM	442	CG	ARG	27	26.739	6.335	6.068	1.00	0.00	C
ATOM	443	HG2	ARG	27	26.235	7.229	6.435	1.00	0.00	H
ATOM	444	HG3	ARG	27	27.521	6.022	6.760	1.00	0.00	H
ATOM	445	CD	ARG	27	25.725	5.205	5.805	1.00	0.00	C
ATOM	446	HD2	ARG	27	25.355	4.891	6.782	1.00	0.00	H
ATOM	447	HD3	ARG	27	26.282	4.399	5.328	1.00	0.00	H
ATOM	448	NE	ARG	27	24.663	5.547	4.907	1.00	0.00	N
ATOM	449	HE	ARG	27	24.681	5.091	4.006	1.00	0.00	H
ATOM	450	CZ	ARG	27	23.672	6.434	5.136	1.00	0.00	C
ATOM	451	NH1	ARG	27	23.671	7.245	6.165	1.00	0.00	N
ATOM	452	HH11	ARG	27	24.401	7.401	6.845	1.00	0.00	H
ATOM	453	HH12	ARG	27	23.099	8.072	6.071	1.00	0.00	H
ATOM	454	NH2	ARG	27	22.712	6.646	4.290	1.00	0.00	N
ATOM	455	HH21	ARG	27	22.661	6.130	3.423	1.00	0.00	H
ATOM	456	HH22	ARG	27	21.905	7.166	4.606	1.00	0.00	H
ATOM	457	C	ARG	27	29.581	7.917	3.931	1.00	0.00	C
ATOM	458	O	ARG	27	29.511	8.482	2.835	1.00	0.00	O
ATOM	459	N	GLU	28	30.552	7.130	4.370	1.00	0.00	N
ATOM	460	H	GLU	28	30.515	6.789	5.320	1.00	0.00	H
ATOM	461	CA	GLU	28	31.779	7.014	3.634	1.00	0.00	C
ATOM	462	HA	GLU	28	31.994	8.025	3.289	1.00	0.00	H
ATOM	463	CB	GLU	28	32.874	6.771	4.619	1.00	0.00	C
ATOM	464	HB2	GLU	28	33.790	6.548	4.072	1.00	0.00	H
ATOM	465	HB3	GLU	28	32.552	5.847	5.100	1.00	0.00	H
ATOM	466	CG	GLU	28	33.165	8.008	5.497	1.00	0.00	C
ATOM	467	HG2	GLU	28	32.457	8.360	6.247	1.00	0.00	H
ATOM	468	HG3	GLU	28	33.366	8.748	4.723	1.00	0.00	H
ATOM	469	CD	GLU	28	34.650	7.934	6.022	1.00	0.00	C
ATOM	470	OE1	GLU	28	34.945	6.985	6.834	1.00	0.00	O
ATOM	471	OE2	GLU	28	35.531	8.660	5.534	1.00	0.00	O
ATOM	472	C	GLU	28	31.764	6.065	2.468	1.00	0.00	C
ATOM	473	O	GLU	28	32.749	5.750	1.807	1.00	0.00	O
ATOM	474	N	ASN	29	30.565	5.697	2.021	1.00	0.00	N
ATOM	475	H	ASN	29	29.756	5.989	2.551	1.00	0.00	H
ATOM	476	CA	ASN	29	30.372	4.817	0.851	1.00	0.00	C
ATOM	477	HA	ASN	29	31.342	4.399	0.580	1.00	0.00	H
ATOM	478	CB	ASN	29	29.517	3.706	1.491	1.00	0.00	C
ATOM	479	HB2	ASN	29	29.978	3.261	2.373	1.00	0.00	H
ATOM	480	HB3	ASN	29	29.483	2.939	0.718	1.00	0.00	H
ATOM	481	CG	ASN	29	28.058	4.025	1.803	1.00	0.00	C
ATOM	482	OD1	ASN	29	27.577	5.130	2.080	1.00	0.00	O
ATOM	483	ND2	ASN	29	27.285	2.973	1.732	1.00	0.00	N
ATOM	484	HD21	ASN	29	26.304	3.159	1.889	1.00	0.00	H
ATOM	485	HD22	ASN	29	27.667	2.146	1.296	1.00	0.00	H
ATOM	486	C	ASN	29	29.744	5.482	-0.374	1.00	0.00	C
ATOM	487	O	ASN	29	29.395	4.874	-1.403	1.00	0.00	O
ATOM	488	N	MET	30	29.390	6.797	-0.193	1.00	0.00	N
ATOM	489	H	MET	30	29.480	7.190	0.733	1.00	0.00	H

ATOM	490	CA	MET	30	28.675	7.638	-1.198	1.00	0.00	C
ATOM	491	HA	MET	30	27.963	6.991	-1.711	1.00	0.00	H
ATOM	492	CB	MET	30	27.814	8.663	-0.398	1.00	0.00	C
ATOM	493	HB2	MET	30	27.267	9.382	-1.008	1.00	0.00	H
ATOM	494	HB3	MET	30	28.515	9.215	0.228	1.00	0.00	H
ATOM	495	CG	MET	30	26.727	8.089	0.457	1.00	0.00	C
ATOM	496	HG2	MET	30	27.018	7.171	0.967	1.00	0.00	H
ATOM	497	HG3	MET	30	26.042	7.735	-0.313	1.00	0.00	H
ATOM	498	SD	MET	30	26.137	9.332	1.612	1.00	0.00	S
ATOM	499	CE	MET	30	24.484	8.582	1.922	1.00	0.00	C
ATOM	500	HE1	MET	30	24.425	7.503	1.785	1.00	0.00	H
ATOM	501	HE2	MET	30	24.157	8.709	2.954	1.00	0.00	H
ATOM	502	HE3	MET	30	23.707	9.029	1.301	1.00	0.00	H
ATOM	503	C	MET	30	29.645	8.223	-2.173	1.00	0.00	C
ATOM	504	O	MET	30	30.698	8.826	-1.769	1.00	0.00	O
ATOM	505	N	TYR	31	29.156	7.991	-3.430	1.00	0.00	N
ATOM	506	H	TYR	31	28.430	7.317	-3.630	1.00	0.00	H
ATOM	507	CA	TYR	31	29.777	8.541	-4.647	1.00	0.00	C
ATOM	508	HA	TYR	31	30.479	9.338	-4.400	1.00	0.00	H
ATOM	509	CB	TYR	31	30.500	7.418	-5.326	1.00	0.00	C
ATOM	510	HB2	TYR	31	29.825	6.607	-5.598	1.00	0.00	H
ATOM	511	HB3	TYR	31	31.104	7.277	-4.429	1.00	0.00	H
ATOM	512	CG	TYR	31	31.423	7.488	-6.495	1.00	0.00	C
ATOM	513	CD1	TYR	31	32.757	7.116	-6.345	1.00	0.00	C
ATOM	514	HD1	TYR	31	33.058	6.812	-5.353	1.00	0.00	H
ATOM	515	CE1	TYR	31	33.600	7.076	-7.446	1.00	0.00	C
ATOM	516	HE1	TYR	31	34.643	6.812	-7.347	1.00	0.00	H
ATOM	517	CZ	TYR	31	33.077	7.218	-8.774	1.00	0.00	C
ATOM	518	OH	TYR	31	33.846	7.070	-9.881	1.00	0.00	O
ATOM	519	HH	TYR	31	33.460	7.301	-10.729	1.00	0.00	H
ATOM	520	CE2	TYR	31	31.718	7.622	-8.942	1.00	0.00	C
ATOM	521	HE2	TYR	31	31.307	7.897	-9.901	1.00	0.00	H
ATOM	522	CD2	TYR	31	30.873	7.666	-7.778	1.00	0.00	C
ATOM	523	HD2	TYR	31	29.811	7.839	-7.870	1.00	0.00	H
ATOM	524	C	TYR	31	28.858	9.383	-5.548	1.00	0.00	C
ATOM	525	O	TYR	31	29.209	10.408	-6.165	1.00	0.00	O
ATOM	526	N	ARG	32	27.500	9.081	-5.500	1.00	0.00	N
ATOM	527	H	ARG	32	27.259	8.266	-4.954	1.00	0.00	H
ATOM	528	CA	ARG	32	26.418	9.804	-6.216	1.00	0.00	C
ATOM	529	HA	ARG	32	26.893	10.367	-7.020	1.00	0.00	H
ATOM	530	CB	ARG	32	25.416	8.848	-6.858	1.00	0.00	C
ATOM	531	HB2	ARG	32	24.485	9.377	-7.062	1.00	0.00	H
ATOM	532	HB3	ARG	32	25.208	8.106	-6.088	1.00	0.00	H
ATOM	533	CG	ARG	32	25.791	8.163	-8.240	1.00	0.00	C
ATOM	534	HG2	ARG	32	26.254	8.868	-8.930	1.00	0.00	H
ATOM	535	HG3	ARG	32	24.868	7.717	-8.609	1.00	0.00	H
ATOM	536	CD	ARG	32	26.828	7.041	-8.292	1.00	0.00	C
ATOM	537	HD2	ARG	32	27.788	7.364	-8.696	1.00	0.00	H
ATOM	538	HD3	ARG	32	26.345	6.354	-8.987	1.00	0.00	H
ATOM	539	NE	ARG	32	27.054	6.305	-7.058	1.00	0.00	N
ATOM	540	HE	ARG	32	26.708	6.674	-6.184	1.00	0.00	H
ATOM	541	CZ	ARG	32	27.789	5.229	-6.969	1.00	0.00	C
ATOM	542	NH1	ARG	32	28.486	4.726	-7.921	1.00	0.00	N
ATOM	543	HH11	ARG	32	28.444	5.181	-8.821	1.00	0.00	H
ATOM	544	HH12	ARG	32	29.237	4.068	-7.765	1.00	0.00	H
ATOM	545	NH2	ARG	32	27.964	4.568	-5.857	1.00	0.00	N
ATOM	546	HH21	ARG	32	27.495	4.925	-5.037	1.00	0.00	H
ATOM	547	HH22	ARG	32	28.441	3.678	-5.884	1.00	0.00	H
ATOM	548	C	ARG	32	25.736	10.947	-5.352	1.00	0.00	C
ATOM	549	O	ARG	32	24.822	11.526	-5.884	1.00	0.00	O
ATOM	550	N	TYR	33	26.133	11.181	-4.058	1.00	0.00	N
ATOM	551	H	TYR	33	26.947	10.683	-3.729	1.00	0.00	H
ATOM	552	CA	TYR	33	25.669	12.266	-3.200	1.00	0.00	C

```
ATOM    553  HA  TYR    33      25.125  12.989  -3.808  1.00  0.00           H
ATOM    554  CB  TYR    33      24.600  11.700  -2.243  1.00  0.00           C
ATOM    555  HB2 TYR    33      24.862  10.712  -1.863  1.00  0.00           H
ATOM    556  HB3 TYR    33      23.765  11.621  -2.939  1.00  0.00           H
ATOM    557  CG  TYR    33      24.219  12.652  -1.192  1.00  0.00           C
ATOM    558  CD1 TYR    33      24.596  12.545   0.143  1.00  0.00           C
ATOM    559  HD1 TYR    33      25.300  11.788   0.454  1.00  0.00           H
ATOM    560  CE1 TYR    33      24.103  13.441   1.141  1.00  0.00           C
ATOM    561  HE1 TYR    33      24.308  13.412   2.201  1.00  0.00           H
ATOM    562  CZ  TYR    33      23.268  14.564   0.676  1.00  0.00           C
ATOM    563  OH  TYR    33      23.062  15.667   1.496  1.00  0.00           O
ATOM    564  HH  TYR    33      22.425  16.229   1.049  1.00  0.00           H
ATOM    565  CE2 TYR    33      22.919  14.699  -0.666  1.00  0.00           C
ATOM    566  HE2 TYR    33      22.217  15.389  -1.112  1.00  0.00           H
ATOM    567  CD2 TYR    33      23.413  13.737  -1.537  1.00  0.00           C
ATOM    568  HD2 TYR    33      23.072  13.691  -2.561  1.00  0.00           H
ATOM    569  C   TYR    33      26.862  12.975  -2.503  1.00  0.00           C
ATOM    570  O   TYR    33      27.893  12.368  -2.293  1.00  0.00           O
ATOM    571  N   NME    34      26.714  14.280  -2.163  1.00  0.00           N
ATOM    572  H   NME    34      25.892  14.845  -2.326  1.00  0.00           H
ATOM    573  CH3 NME    34      27.995  14.938  -1.668  1.00  0.00           C
ATOM    574 HH31 NME    34      27.686  15.788  -1.061  1.00  0.00           H
ATOM    575 HH32 NME    34      28.639  14.353  -1.010  1.00  0.00           H
ATOM    576 HH33 NME    34      28.581  15.418  -2.453  1.00  0.00           H
TER     577      NME    34
END
```

Cluster 2, Figure 3B:

```
ATOM      1 HH31 ACE     1      13.700  -0.779  -3.487  1.00  0.00           H
ATOM      2  CH3 ACE     1      14.137  -0.458  -4.432  1.00  0.00           C
ATOM      3 HH32 ACE     1      14.231   0.627  -4.399  1.00  0.00           H
ATOM      4 HH33 ACE     1      13.401  -0.657  -5.211  1.00  0.00           H
ATOM      5  C   ACE     1      15.403  -1.118  -4.875  1.00  0.00           C
ATOM      6  O   ACE     1      15.407  -2.303  -5.308  1.00  0.00           O
ATOM      7  N   ASN     2      16.429  -0.386  -4.635  1.00  0.00           N
ATOM      8  H   ASN     2      16.297   0.534  -4.237  1.00  0.00           H
ATOM      9  CA  ASN     2      17.843  -0.660  -5.011  1.00  0.00           C
ATOM     10  HA  ASN     2      18.093  -1.613  -4.545  1.00  0.00           H
ATOM     11  CB  ASN     2      18.048  -0.747  -6.512  1.00  0.00           C
ATOM     12  HB2 ASN     2      17.566  -1.621  -6.948  1.00  0.00           H
ATOM     13  HB3 ASN     2      19.115  -0.971  -6.494  1.00  0.00           H
ATOM     14  CG  ASN     2      17.627   0.439  -7.371  1.00  0.00           C
ATOM     15  OD1 ASN     2      18.213   1.495  -7.312  1.00  0.00           O
ATOM     16  ND2 ASN     2      16.745   0.276  -8.300  1.00  0.00           N
ATOM     17 HD21 ASN     2      16.575   1.075  -8.894  1.00  0.00           H
ATOM     18 HD22 ASN     2      16.165  -0.548  -8.368  1.00  0.00           H
ATOM     19  C   ASN     2      18.804   0.406  -4.438  1.00  0.00           C
ATOM     20  O   ASN     2      18.445   1.455  -3.917  1.00  0.00           O
ATOM     21  N   ASP     3      20.119   0.115  -4.469  1.00  0.00           N
ATOM     22  H   ASP     3      20.293  -0.848  -4.717  1.00  0.00           H
ATOM     23  CA  ASP     3      21.148   0.988  -4.056  1.00  0.00           C
ATOM     24  HA  ASP     3      21.058   1.215  -2.994  1.00  0.00           H
ATOM     25  CB  ASP     3      22.590   0.452  -4.052  1.00  0.00           C
ATOM     26  HB2 ASP     3      22.760   0.112  -5.074  1.00  0.00           H
ATOM     27  HB3 ASP     3      22.539  -0.370  -3.336  1.00  0.00           H
ATOM     28  CG  ASP     3      23.704   1.547  -3.713  1.00  0.00           C
ATOM     29  OD1 ASP     3      24.380   2.078  -4.631  1.00  0.00           O
ATOM     30  OD2 ASP     3      23.887   1.818  -2.503  1.00  0.00           O
ATOM     31  C   ASP     3      21.183   2.295  -4.905  1.00  0.00           C
ATOM     32  O   ASP     3      20.959   2.316  -6.161  1.00  0.00           O
```

ATOM	33	N	TYR	4	21.296	3.457	-4.280	1.00	0.00	N
ATOM	34	H	TYR	4	21.468	3.363	-3.288	1.00	0.00	H
ATOM	35	CA	TYR	4	21.402	4.810	-4.749	1.00	0.00	C
ATOM	36	HA	TYR	4	21.896	4.852	-5.719	1.00	0.00	H
ATOM	37	CB	TYR	4	20.031	5.409	-5.032	1.00	0.00	C
ATOM	38	HB2	TYR	4	19.506	5.522	-4.084	1.00	0.00	H
ATOM	39	HB3	TYR	4	19.527	4.684	-5.672	1.00	0.00	H
ATOM	40	CG	TYR	4	19.991	6.757	-5.694	1.00	0.00	C
ATOM	41	CD1	TYR	4	20.546	6.797	-6.980	1.00	0.00	C
ATOM	42	HD1	TYR	4	20.900	5.858	-7.376	1.00	0.00	H
ATOM	43	CE1	TYR	4	20.471	7.979	-7.725	1.00	0.00	C
ATOM	44	HE1	TYR	4	20.825	8.046	-8.744	1.00	0.00	H
ATOM	45	CZ	TYR	4	19.867	9.117	-7.139	1.00	0.00	C
ATOM	46	OH	TYR	4	19.831	10.274	-7.829	1.00	0.00	O
ATOM	47	HH	TYR	4	19.465	10.957	-7.263	1.00	0.00	H
ATOM	48	CE2	TYR	4	19.479	9.105	-5.809	1.00	0.00	C
ATOM	49	HE2	TYR	4	19.232	10.091	-5.447	1.00	0.00	H
ATOM	50	CD2	TYR	4	19.510	7.900	-4.990	1.00	0.00	C
ATOM	51	HD2	TYR	4	19.197	7.854	-3.957	1.00	0.00	H
ATOM	52	C	TYR	4	22.196	5.613	-3.657	1.00	0.00	C
ATOM	53	O	TYR	4	22.270	5.133	-2.513	1.00	0.00	O
ATOM	54	N	GLU	5	22.676	6.803	-3.973	1.00	0.00	N
ATOM	55	H	GLU	5	22.528	7.053	-4.940	1.00	0.00	H
ATOM	56	CA	GLU	5	23.491	7.744	-3.209	1.00	0.00	C
ATOM	57	HA	GLU	5	23.976	7.122	-2.456	1.00	0.00	H
ATOM	58	CB	GLU	5	24.557	8.496	-4.129	1.00	0.00	C
ATOM	59	HB2	GLU	5	25.264	8.980	-3.455	1.00	0.00	H
ATOM	60	HB3	GLU	5	23.972	9.236	-4.675	1.00	0.00	H
ATOM	61	CG	GLU	5	25.362	7.753	-5.179	1.00	0.00	C
ATOM	62	HG2	GLU	5	26.117	8.409	-5.611	1.00	0.00	H
ATOM	63	HG3	GLU	5	24.598	7.333	-5.834	1.00	0.00	H
ATOM	64	CD	GLU	5	26.216	6.671	-4.510	1.00	0.00	C
ATOM	65	OE1	GLU	5	26.786	6.837	-3.424	1.00	0.00	O
ATOM	66	OE2	GLU	5	26.369	5.618	-5.140	1.00	0.00	O
ATOM	67	C	GLU	5	22.738	8.794	-2.300	1.00	0.00	C
ATOM	68	O	GLU	5	23.457	9.569	-1.724	1.00	0.00	O
ATOM	69	N	ASP	6	21.430	8.733	-2.033	1.00	0.00	N
ATOM	70	H	ASP	6	20.901	8.051	-2.558	1.00	0.00	H
ATOM	71	CA	ASP	6	20.785	9.637	-1.081	1.00	0.00	C
ATOM	72	HA	ASP	6	21.531	10.010	-0.378	1.00	0.00	H
ATOM	73	CB	ASP	6	20.156	10.840	-1.775	1.00	0.00	C
ATOM	74	HB2	ASP	6	19.689	10.723	-2.753	1.00	0.00	H
ATOM	75	HB3	ASP	6	21.070	11.422	-1.897	1.00	0.00	H
ATOM	76	CG	ASP	6	19.189	11.694	-0.892	1.00	0.00	C
ATOM	77	OD1	ASP	6	19.672	12.148	0.164	1.00	0.00	O
ATOM	78	OD2	ASP	6	18.061	12.003	-1.287	1.00	0.00	O
ATOM	79	C	ASP	6	19.698	8.967	-0.147	1.00	0.00	C
ATOM	80	O	ASP	6	19.758	9.246	1.050	1.00	0.00	O
ATOM	81	N	ARG	7	18.801	8.095	-0.598	1.00	0.00	N
ATOM	82	H	ARG	7	18.759	8.062	-1.606	1.00	0.00	H
ATOM	83	CA	ARG	7	17.735	7.450	0.122	1.00	0.00	C
ATOM	84	HA	ARG	7	17.498	8.090	0.971	1.00	0.00	H
ATOM	85	CB	ARG	7	16.427	7.419	-0.757	1.00	0.00	C
ATOM	86	HB2	ARG	7	15.669	6.938	-0.141	1.00	0.00	H
ATOM	87	HB3	ARG	7	16.702	6.793	-1.605	1.00	0.00	H
ATOM	88	CG	ARG	7	15.774	8.717	-1.182	1.00	0.00	C
ATOM	89	HG2	ARG	7	14.930	8.601	-1.860	1.00	0.00	H
ATOM	90	HG3	ARG	7	16.626	9.165	-1.694	1.00	0.00	H
ATOM	91	CD	ARG	7	15.276	9.545	0.019	1.00	0.00	C
ATOM	92	HD2	ARG	7	15.583	9.105	0.968	1.00	0.00	H
ATOM	93	HD3	ARG	7	14.217	9.660	-0.214	1.00	0.00	H
ATOM	94	NE	ARG	7	15.906	10.831	0.037	1.00	0.00	N
ATOM	95	HE	ARG	7	16.696	11.005	-0.568	1.00	0.00	H

ATOM	96	CZ	ARG	7	15.715	11.769	0.918	1.00	0.00	C
ATOM	97	NH1	ARG	7	14.829	11.645	1.899	1.00	0.00	N
ATOM	98	HH11	ARG	7	14.176	10.874	1.895	1.00	0.00	H
ATOM	99	HH12	ARG	7	14.815	12.214	2.733	1.00	0.00	H
ATOM	100	NH2	ARG	7	16.560	12.788	1.045	1.00	0.00	N
ATOM	101	HH21	ARG	7	17.297	12.962	0.377	1.00	0.00	H
ATOM	102	HH22	ARG	7	16.407	13.468	1.776	1.00	0.00	H
ATOM	103	C	ARG	7	18.055	6.011	0.660	1.00	0.00	C
ATOM	104	O	ARG	7	17.420	5.555	1.645	1.00	0.00	O
ATOM	105	N	TYR	8	18.972	5.240	0.157	1.00	0.00	N
ATOM	106	H	TYR	8	19.586	5.600	-0.559	1.00	0.00	H
ATOM	107	CA	TYR	8	19.202	3.820	0.519	1.00	0.00	C
ATOM	108	HA	TYR	8	18.337	3.442	1.064	1.00	0.00	H
ATOM	109	CB	TYR	8	19.451	2.902	-0.642	1.00	0.00	C
ATOM	110	HB2	TYR	8	20.375	3.161	-1.159	1.00	0.00	H
ATOM	111	HB3	TYR	8	18.576	3.106	-1.261	1.00	0.00	H
ATOM	112	CG	TYR	8	19.484	1.407	-0.272	1.00	0.00	C
ATOM	113	CD1	TYR	8	20.733	0.734	-0.438	1.00	0.00	C
ATOM	114	HD1	TYR	8	21.577	1.335	-0.743	1.00	0.00	H
ATOM	115	CE1	TYR	8	20.912	-0.662	-0.159	1.00	0.00	C
ATOM	116	HE1	TYR	8	21.881	-1.126	-0.264	1.00	0.00	H
ATOM	117	CZ	TYR	8	19.798	-1.402	0.139	1.00	0.00	C
ATOM	118	OH	TYR	8	19.818	-2.733	0.293	1.00	0.00	O
ATOM	119	HH	TYR	8	20.686	-3.094	0.096	1.00	0.00	H
ATOM	120	CE2	TYR	8	18.574	-0.767	0.390	1.00	0.00	C
ATOM	121	HE2	TYR	8	17.698	-1.372	0.575	1.00	0.00	H
ATOM	122	CD2	TYR	8	18.374	0.658	0.179	1.00	0.00	C
ATOM	123	HD2	TYR	8	17.419	1.159	0.243	1.00	0.00	H
ATOM	124	C	TYR	8	20.332	3.800	1.561	1.00	0.00	C
ATOM	125	O	TYR	8	21.234	4.641	1.526	1.00	0.00	O
ATOM	126	N	TYR	9	20.321	2.960	2.600	1.00	0.00	N
ATOM	127	H	TYR	9	19.601	2.251	2.592	1.00	0.00	H
ATOM	128	CA	TYR	9	21.256	2.826	3.711	1.00	0.00	C
ATOM	129	HA	TYR	9	21.853	3.738	3.712	1.00	0.00	H
ATOM	130	CB	TYR	9	20.526	2.782	5.058	1.00	0.00	C
ATOM	131	HB2	TYR	9	21.180	2.675	5.924	1.00	0.00	H
ATOM	132	HB3	TYR	9	19.919	1.877	5.016	1.00	0.00	H
ATOM	133	CG	TYR	9	19.667	3.929	5.389	1.00	0.00	C
ATOM	134	CD1	TYR	9	18.264	3.766	5.503	1.00	0.00	C
ATOM	135	HD1	TYR	9	17.851	2.828	5.161	1.00	0.00	H
ATOM	136	CE1	TYR	9	17.426	4.800	5.925	1.00	0.00	C
ATOM	137	HE1	TYR	9	16.364	4.620	5.848	1.00	0.00	H
ATOM	138	CZ	TYR	9	17.957	6.080	6.153	1.00	0.00	C
ATOM	139	OH	TYR	9	17.146	7.126	6.539	1.00	0.00	O
ATOM	140	HH	TYR	9	17.625	7.952	6.435	1.00	0.00	H
ATOM	141	CE2	TYR	9	19.335	6.260	6.148	1.00	0.00	C
ATOM	142	HE2	TYR	9	19.702	7.248	6.382	1.00	0.00	H
ATOM	143	CD2	TYR	9	20.216	5.173	5.803	1.00	0.00	C
ATOM	144	HD2	TYR	9	21.289	5.294	5.796	1.00	0.00	H
ATOM	145	C	TYR	9	22.360	1.711	3.524	1.00	0.00	C
ATOM	146	O	TYR	9	21.970	0.540	3.180	1.00	0.00	O
ATOM	147	N	ARG	10	23.636	2.064	3.842	1.00	0.00	N
ATOM	148	H	ARG	10	23.806	2.995	4.196	1.00	0.00	H
ATOM	149	CA	ARG	10	24.770	1.106	3.678	1.00	0.00	C
ATOM	150	HA	ARG	10	24.472	0.121	3.317	1.00	0.00	H
ATOM	151	CB	ARG	10	25.718	1.798	2.727	1.00	0.00	C
ATOM	152	HB2	ARG	10	26.554	1.114	2.586	1.00	0.00	H
ATOM	153	HB3	ARG	10	26.130	2.680	3.218	1.00	0.00	H
ATOM	154	CG	ARG	10	25.202	2.109	1.347	1.00	0.00	C
ATOM	155	HG2	ARG	10	24.295	2.713	1.353	1.00	0.00	H
ATOM	156	HG3	ARG	10	24.931	1.164	0.877	1.00	0.00	H
ATOM	157	CD	ARG	10	26.218	2.785	0.464	1.00	0.00	C
ATOM	158	HD2	ARG	10	27.209	2.334	0.439	1.00	0.00	H

ATOM	159	HD3	ARG	10	26.402	3.786	0.853	1.00	0.00	H
ATOM	160	NE	ARG	10	25.671	3.096	-0.867	1.00	0.00	N
ATOM	161	HE	ARG	10	24.910	2.585	-1.289	1.00	0.00	H
ATOM	162	CZ	ARG	10	25.977	4.136	-1.594	1.00	0.00	C
ATOM	163	NH1	ARG	10	26.800	5.053	-1.275	1.00	0.00	N
ATOM	164	HH11	ARG	10	27.284	5.015	-0.390	1.00	0.00	H
ATOM	165	HH12	ARG	10	26.925	5.897	-1.815	1.00	0.00	H
ATOM	166	NH2	ARG	10	25.397	4.216	-2.754	1.00	0.00	N
ATOM	167	HH21	ARG	10	24.874	3.392	-3.011	1.00	0.00	H
ATOM	168	HH22	ARG	10	25.845	4.818	-3.430	1.00	0.00	H
ATOM	169	C	ARG	10	25.267	0.795	5.073	1.00	0.00	C
ATOM	170	O	ARG	10	25.899	1.573	5.788	1.00	0.00	O
ATOM	171	N	GLU	11	25.003	-0.491	5.440	1.00	0.00	N
ATOM	172	H	GLU	11	24.603	-1.174	4.811	1.00	0.00	H
ATOM	173	CA	GLU	11	25.338	-1.027	6.809	1.00	0.00	C
ATOM	174	HA	GLU	11	24.906	-0.360	7.554	1.00	0.00	H
ATOM	175	CB	GLU	11	24.650	-2.414	7.010	1.00	0.00	C
ATOM	176	HB2	GLU	11	25.015	-2.968	6.145	1.00	0.00	H
ATOM	177	HB3	GLU	11	23.647	-1.991	6.953	1.00	0.00	H
ATOM	178	CG	GLU	11	24.772	-3.138	8.377	1.00	0.00	C
ATOM	179	HG2	GLU	11	25.801	-3.334	8.676	1.00	0.00	H
ATOM	180	HG3	GLU	11	24.192	-4.050	8.229	1.00	0.00	H
ATOM	181	CD	GLU	11	24.173	-2.319	9.494	1.00	0.00	C
ATOM	182	OE1	GLU	11	22.942	-2.288	9.583	1.00	0.00	O
ATOM	183	OE2	GLU	11	24.885	-1.663	10.289	1.00	0.00	O
ATOM	184	C	GLU	11	26.822	-1.204	7.049	1.00	0.00	C
ATOM	185	O	GLU	11	27.682	-1.227	6.155	1.00	0.00	O
ATOM	186	N	ASN	12	27.162	-1.104	8.318	1.00	0.00	N
ATOM	187	H	ASN	12	26.439	-1.043	9.022	1.00	0.00	H
ATOM	188	CA	ASN	12	28.536	-1.236	8.808	1.00	0.00	C
ATOM	189	HA	ASN	12	28.967	-0.235	8.793	1.00	0.00	H
ATOM	190	CB	ASN	12	28.412	-1.536	10.271	1.00	0.00	C
ATOM	191	HB2	ASN	12	27.897	-2.488	10.402	1.00	0.00	H
ATOM	192	HB3	ASN	12	27.808	-0.689	10.597	1.00	0.00	H
ATOM	193	CG	ASN	12	29.651	-1.578	11.101	1.00	0.00	C
ATOM	194	OD1	ASN	12	30.315	-2.606	11.113	1.00	0.00	O
ATOM	195	ND2	ASN	12	29.987	-0.472	11.785	1.00	0.00	N
ATOM	196	HD21	ASN	12	30.833	-0.469	12.336	1.00	0.00	H
ATOM	197	HD22	ASN	12	29.318	0.285	11.759	1.00	0.00	H
ATOM	198	C	ASN	12	29.471	-2.301	8.154	1.00	0.00	C
ATOM	199	O	ASN	12	30.639	-1.972	7.938	1.00	0.00	O
ATOM	200	N	MET	13	28.916	-3.430	7.653	1.00	0.00	N
ATOM	201	H	MET	13	27.962	-3.667	7.884	1.00	0.00	H
ATOM	202	CA	MET	13	29.761	-4.505	7.050	1.00	0.00	C
ATOM	203	HA	MET	13	30.644	-4.477	7.688	1.00	0.00	H
ATOM	204	CB	MET	13	29.272	-5.922	7.296	1.00	0.00	C
ATOM	205	HB2	MET	13	28.267	-6.097	6.912	1.00	0.00	H
ATOM	206	HB3	MET	13	29.187	-5.942	8.383	1.00	0.00	H
ATOM	207	CG	MET	13	30.285	-7.035	6.889	1.00	0.00	C
ATOM	208	HG2	MET	13	31.284	-6.693	7.156	1.00	0.00	H
ATOM	209	HG3	MET	13	30.149	-6.989	5.808	1.00	0.00	H
ATOM	210	SD	MET	13	29.880	-8.682	7.489	1.00	0.00	S
ATOM	211	CE	MET	13	28.436	-9.056	6.350	1.00	0.00	C
ATOM	212	HE1	MET	13	28.677	-9.266	5.308	1.00	0.00	H
ATOM	213	HE2	MET	13	27.938	-9.931	6.765	1.00	0.00	H
ATOM	214	HE3	MET	13	27.733	-8.231	6.465	1.00	0.00	H
ATOM	215	C	MET	13	29.994	-4.344	5.517	1.00	0.00	C
ATOM	216	O	MET	13	31.082	-4.619	5.019	1.00	0.00	O
ATOM	217	N	TYR	14	28.965	-3.826	4.816	1.00	0.00	N
ATOM	218	H	TYR	14	28.062	-3.709	5.254	1.00	0.00	H
ATOM	219	CA	TYR	14	28.946	-3.870	3.298	1.00	0.00	C
ATOM	220	HA	TYR	14	29.367	-4.848	3.063	1.00	0.00	H
ATOM	221	CB	TYR	14	27.441	-3.928	2.715	1.00	0.00	C

ATOM	222	HB2	TYR	14	27.411	-4.147	1.647	1.00	0.00	H
ATOM	223	HB3	TYR	14	27.036	-2.923	2.837	1.00	0.00	H
ATOM	224	CG	TYR	14	26.453	-4.917	3.238	1.00	0.00	C
ATOM	225	CD1	TYR	14	25.202	-4.492	3.753	1.00	0.00	C
ATOM	226	HD1	TYR	14	25.026	-3.428	3.815	1.00	0.00	H
ATOM	227	CE1	TYR	14	24.164	-5.399	4.148	1.00	0.00	C
ATOM	228	HE1	TYR	14	23.243	-5.012	4.558	1.00	0.00	H
ATOM	229	CZ	TYR	14	24.402	-6.826	4.066	1.00	0.00	C
ATOM	230	OH	TYR	14	23.365	-7.576	4.380	1.00	0.00	O
ATOM	231	HH	TYR	14	22.780	-7.094	4.968	1.00	0.00	H
ATOM	232	CE2	TYR	14	25.606	-7.209	3.563	1.00	0.00	C
ATOM	233	HE2	TYR	14	25.650	-8.289	3.555	1.00	0.00	H
ATOM	234	CD2	TYR	14	26.690	-6.309	3.148	1.00	0.00	C
ATOM	235	HD2	TYR	14	27.620	-6.635	2.706	1.00	0.00	H
ATOM	236	C	TYR	14	29.774	-2.766	2.616	1.00	0.00	C
ATOM	237	O	TYR	14	30.439	-2.988	1.610	1.00	0.00	O
ATOM	238	N	ARG	15	29.635	-1.662	3.322	1.00	0.00	N
ATOM	239	H	ARG	15	29.037	-1.543	4.128	1.00	0.00	H
ATOM	240	CA	ARG	15	30.491	-0.465	3.150	1.00	0.00	C
ATOM	241	HA	ARG	15	30.055	0.229	2.432	1.00	0.00	H
ATOM	242	CB	ARG	15	30.499	0.301	4.390	1.00	0.00	C
ATOM	243	HB2	ARG	15	31.309	1.023	4.489	1.00	0.00	H
ATOM	244	HB3	ARG	15	30.534	-0.462	5.169	1.00	0.00	H
ATOM	245	CG	ARG	15	29.276	1.190	4.772	1.00	0.00	C
ATOM	246	HG2	ARG	15	28.314	0.701	4.630	1.00	0.00	H
ATOM	247	HG3	ARG	15	29.215	2.148	4.254	1.00	0.00	H
ATOM	248	CD	ARG	15	29.299	1.499	6.312	1.00	0.00	C
ATOM	249	HD2	ARG	15	30.303	1.664	6.705	1.00	0.00	H
ATOM	250	HD3	ARG	15	28.872	0.643	6.835	1.00	0.00	H
ATOM	251	NE	ARG	15	28.400	2.592	6.596	1.00	0.00	N
ATOM	252	HE	ARG	15	27.445	2.337	6.386	1.00	0.00	H
ATOM	253	CZ	ARG	15	28.669	3.794	7.100	1.00	0.00	C
ATOM	254	NH1	ARG	15	29.796	4.305	7.305	1.00	0.00	N
ATOM	255	HH11	ARG	15	30.632	3.817	7.016	1.00	0.00	H
ATOM	256	HH12	ARG	15	29.774	5.010	8.028	1.00	0.00	H
ATOM	257	NH2	ARG	15	27.652	4.449	7.553	1.00	0.00	N
ATOM	258	HH21	ARG	15	26.823	3.898	7.727	1.00	0.00	H
ATOM	259	HH22	ARG	15	27.830	5.311	8.047	1.00	0.00	H
ATOM	260	C	ARG	15	31.956	-0.575	2.633	1.00	0.00	C
ATOM	261	O	ARG	15	32.441	0.170	1.758	1.00	0.00	O
ATOM	262	N	TYR	16	32.623	-1.544	3.204	1.00	0.00	N
ATOM	263	H	TYR	16	32.283	-1.918	4.079	1.00	0.00	H
ATOM	264	CA	TYR	16	33.985	-1.989	2.674	1.00	0.00	C
ATOM	265	HA	TYR	16	34.219	-1.345	1.828	1.00	0.00	H
ATOM	266	CB	TYR	16	35.006	-1.523	3.717	1.00	0.00	C
ATOM	267	HB2	TYR	16	34.797	-1.977	4.685	1.00	0.00	H
ATOM	268	HB3	TYR	16	34.776	-0.460	3.800	1.00	0.00	H
ATOM	269	CG	TYR	16	36.425	-1.726	3.518	1.00	0.00	C
ATOM	270	CD1	TYR	16	37.065	-2.825	4.138	1.00	0.00	C
ATOM	271	HD1	TYR	16	36.475	-3.550	4.681	1.00	0.00	H
ATOM	272	CE1	TYR	16	38.458	-2.919	4.108	1.00	0.00	C
ATOM	273	HE1	TYR	16	38.897	-3.797	4.557	1.00	0.00	H
ATOM	274	CZ	TYR	16	39.262	-2.034	3.313	1.00	0.00	C
ATOM	275	OH	TYR	16	40.602	-2.088	3.156	1.00	0.00	O
ATOM	276	HH	TYR	16	40.890	-1.247	2.794	1.00	0.00	H
ATOM	277	CE2	TYR	16	38.572	-0.870	2.721	1.00	0.00	C
ATOM	278	HE2	TYR	16	39.132	-0.085	2.235	1.00	0.00	H
ATOM	279	CD2	TYR	16	37.210	-0.742	2.831	1.00	0.00	C
ATOM	280	HD2	TYR	16	36.795	0.224	2.584	1.00	0.00	H
ATOM	281	C	TYR	16	34.174	-3.450	2.300	1.00	0.00	C
ATOM	282	O	TYR	16	35.011	-3.790	1.464	1.00	0.00	O
ATOM	283	N	NME	17	33.426	-4.319	2.977	1.00	0.00	N
ATOM	284	H	NME	17	32.795	-3.947	3.671	1.00	0.00	H

```
ATOM    285  CH3 NME    17      33.527  -5.752   2.714  1.00  0.00           C
ATOM    286 HH31 NME    17      34.522  -6.042   2.372  1.00  0.00           H
ATOM    287 HH32 NME    17      33.424  -6.286   3.659  1.00  0.00           H
ATOM    288 HH33 NME    17      32.790  -6.119   2.000  1.00  0.00           H
TER     289      NME    17
ATOM    289 HH31 ACE    18      10.959   6.220  -1.986  1.00  0.00           H
ATOM    290  CH3 ACE    18      10.663   5.510  -1.214  1.00  0.00           C
ATOM    291 HH32 ACE    18      11.416   4.723  -1.162  1.00  0.00           H
ATOM    292 HH33 ACE    18       9.689   5.096  -1.475  1.00  0.00           H
ATOM    293  C   ACE    18      10.643   6.256   0.117  1.00  0.00           C
ATOM    294  O   ACE    18       9.659   6.394   0.793  1.00  0.00           O
ATOM    295  N   ASN    19      11.810   6.770   0.491  1.00  0.00           N
ATOM    296  H   ASN    19      12.568   6.656  -0.166  1.00  0.00           H
ATOM    297  CA  ASN    19      12.168   7.396   1.730  1.00  0.00           C
ATOM    298  HA  ASN    19      11.519   7.025   2.525  1.00  0.00           H
ATOM    299  CB  ASN    19      13.561   6.850   2.148  1.00  0.00           C
ATOM    300  HB2 ASN    19      14.354   7.123   1.452  1.00  0.00           H
ATOM    301  HB3 ASN    19      13.376   5.777   2.174  1.00  0.00           H
ATOM    302  CG  ASN    19      13.977   7.169   3.595  1.00  0.00           C
ATOM    303  OD1 ASN    19      13.245   7.624   4.429  1.00  0.00           O
ATOM    304  ND2 ASN    19      15.234   6.992   3.938  1.00  0.00           N
ATOM    305 HD21 ASN    19      15.632   7.236   4.835  1.00  0.00           H
ATOM    306 HD22 ASN    19      15.796   6.513   3.250  1.00  0.00           H
ATOM    307  C   ASN    19      11.935   8.907   1.600  1.00  0.00           C
ATOM    308  O   ASN    19      12.732   9.646   2.136  1.00  0.00           O
ATOM    309  N   ASP    20      10.786   9.310   1.052  1.00  0.00           N
ATOM    310  H   ASP    20      10.069   8.624   0.871  1.00  0.00           H
ATOM    311  CA  ASP    20      10.305  10.629   0.636  1.00  0.00           C
ATOM    312  HA  ASP    20      11.133  11.316   0.456  1.00  0.00           H
ATOM    313  CB  ASP    20       9.410  10.384  -0.586  1.00  0.00           C
ATOM    314  HB2 ASP    20       8.438   9.935  -0.381  1.00  0.00           H
ATOM    315  HB3 ASP    20       9.969   9.701  -1.226  1.00  0.00           H
ATOM    316  CG  ASP    20       9.075  11.679  -1.387  1.00  0.00           C
ATOM    317  OD1 ASP    20       7.929  11.970  -1.795  1.00  0.00           O
ATOM    318  OD2 ASP    20      10.040  12.486  -1.468  1.00  0.00           O
ATOM    319  C   ASP    20       9.552  11.310   1.838  1.00  0.00           C
ATOM    320  O   ASP    20       8.783  12.234   1.587  1.00  0.00           O
ATOM    321  N   TYR    21       9.618  10.781   3.102  1.00  0.00           N
ATOM    322  H   TYR    21      10.078   9.890   3.232  1.00  0.00           H
ATOM    323  CA  TYR    21       8.941  11.347   4.282  1.00  0.00           C
ATOM    324  HA  TYR    21       8.256  12.117   3.929  1.00  0.00           H
ATOM    325  CB  TYR    21       8.022  10.187   4.751  1.00  0.00           C
ATOM    326  HB2 TYR    21       7.360   9.941   3.922  1.00  0.00           H
ATOM    327  HB3 TYR    21       7.409  10.599   5.553  1.00  0.00           H
ATOM    328  CG  TYR    21       8.769   8.966   5.328  1.00  0.00           C
ATOM    329  CD1 TYR    21       9.157   8.796   6.687  1.00  0.00           C
ATOM    330  HD1 TYR    21       8.973   9.638   7.337  1.00  0.00           H
ATOM    331  CE1 TYR    21       9.831   7.688   7.127  1.00  0.00           C
ATOM    332  HE1 TYR    21      10.173   7.661   8.150  1.00  0.00           H
ATOM    333  CZ  TYR    21      10.099   6.609   6.216  1.00  0.00           C
ATOM    334  OH  TYR    21      10.731   5.431   6.636  1.00  0.00           O
ATOM    335  HH  TYR    21      10.919   5.483   7.576  1.00  0.00           H
ATOM    336  CE2 TYR    21       9.685   6.729   4.881  1.00  0.00           C
ATOM    337  HE2 TYR    21       9.943   5.949   4.180  1.00  0.00           H
ATOM    338  CD2 TYR    21       8.992   7.931   4.436  1.00  0.00           C
ATOM    339  HD2 TYR    21       8.742   7.918   3.386  1.00  0.00           H
ATOM    340  C   TYR    21       9.825  11.865   5.392  1.00  0.00           C
ATOM    341  O   TYR    21       9.395  12.611   6.239  1.00  0.00           O
ATOM    342  N   GLU    22      11.124  11.658   5.323  1.00  0.00           N
ATOM    343  H   GLU    22      11.362  10.978   4.615  1.00  0.00           H
ATOM    344  CA  GLU    22      12.152  12.267   6.244  1.00  0.00           C
ATOM    345  HA  GLU    22      11.639  13.203   6.463  1.00  0.00           H
ATOM    346  CB  GLU    22      12.284  11.319   7.497  1.00  0.00           C
```

ATOM	347	HB2	GLU	22	12.573	10.366	7.056	1.00	0.00	H
ATOM	348	HB3	GLU	22	11.245	11.196	7.801	1.00	0.00	H
ATOM	349	CG	GLU	22	13.257	11.702	8.654	1.00	0.00	C
ATOM	350	HG2	GLU	22	14.293	11.833	8.340	1.00	0.00	H
ATOM	351	HG3	GLU	22	13.190	10.938	9.428	1.00	0.00	H
ATOM	352	CD	GLU	22	12.876	13.095	9.220	1.00	0.00	C
ATOM	353	OE1	GLU	22	12.189	13.073	10.248	1.00	0.00	O
ATOM	354	OE2	GLU	22	13.248	14.107	8.611	1.00	0.00	O
ATOM	355	C	GLU	22	13.507	12.541	5.527	1.00	0.00	C
ATOM	356	O	GLU	22	13.759	12.175	4.362	1.00	0.00	O
ATOM	357	N	ASP	23	14.304	13.446	6.075	1.00	0.00	N
ATOM	358	H	ASP	23	13.954	13.792	6.956	1.00	0.00	H
ATOM	359	CA	ASP	23	15.533	13.980	5.461	1.00	0.00	C
ATOM	360	HA	ASP	23	15.928	13.222	4.785	1.00	0.00	H
ATOM	361	CB	ASP	23	15.332	15.298	4.716	1.00	0.00	C
ATOM	362	HB2	ASP	23	15.018	16.031	5.457	1.00	0.00	H
ATOM	363	HB3	ASP	23	14.479	15.076	4.074	1.00	0.00	H
ATOM	364	CG	ASP	23	16.524	15.708	3.823	1.00	0.00	C
ATOM	365	OD1	ASP	23	16.852	14.908	2.921	1.00	0.00	O
ATOM	366	OD2	ASP	23	17.032	16.836	3.901	1.00	0.00	O
ATOM	367	C	ASP	23	16.630	14.201	6.583	1.00	0.00	C
ATOM	368	O	ASP	23	17.756	14.593	6.267	1.00	0.00	O
ATOM	369	N	ARG	24	16.340	13.909	7.838	1.00	0.00	N
ATOM	370	H	ARG	24	15.414	13.570	8.058	1.00	0.00	H
ATOM	371	CA	ARG	24	17.160	14.165	9.072	1.00	0.00	C
ATOM	372	HA	ARG	24	18.021	14.724	8.706	1.00	0.00	H
ATOM	373	CB	ARG	24	16.448	14.943	10.125	1.00	0.00	C
ATOM	374	HB2	ARG	24	16.951	15.026	11.088	1.00	0.00	H
ATOM	375	HB3	ARG	24	15.591	14.292	10.295	1.00	0.00	H
ATOM	376	CG	ARG	24	16.013	16.331	9.699	1.00	0.00	C
ATOM	377	HG2	ARG	24	15.553	16.361	8.711	1.00	0.00	H
ATOM	378	HG3	ARG	24	16.837	17.045	9.677	1.00	0.00	H
ATOM	379	CD	ARG	24	14.900	16.859	10.581	1.00	0.00	C
ATOM	380	HD2	ARG	24	14.542	17.838	10.259	1.00	0.00	H
ATOM	381	HD3	ARG	24	15.299	17.007	11.585	1.00	0.00	H
ATOM	382	NE	ARG	24	13.732	15.952	10.732	1.00	0.00	N
ATOM	383	HE	ARG	24	13.588	15.208	10.065	1.00	0.00	H
ATOM	384	CZ	ARG	24	12.636	16.200	11.480	1.00	0.00	C
ATOM	385	NH1	ARG	24	12.523	17.315	12.209	1.00	0.00	N
ATOM	386	HH11	ARG	24	13.203	18.015	11.951	1.00	0.00	H
ATOM	387	HH12	ARG	24	11.607	17.580	12.542	1.00	0.00	H
ATOM	388	NH2	ARG	24	11.656	15.400	11.460	1.00	0.00	N
ATOM	389	HH21	ARG	24	11.726	14.597	10.853	1.00	0.00	H
ATOM	390	HH22	ARG	24	10.850	15.519	12.058	1.00	0.00	H
ATOM	391	C	ARG	24	17.951	12.933	9.510	1.00	0.00	C
ATOM	392	O	ARG	24	18.408	13.044	10.661	1.00	0.00	O
ATOM	393	N	TYR	25	18.215	11.948	8.616	1.00	0.00	N
ATOM	394	H	TYR	25	17.996	12.065	7.637	1.00	0.00	H
ATOM	395	CA	TYR	25	19.191	10.948	8.924	1.00	0.00	C
ATOM	396	HA	TYR	25	19.026	10.670	9.965	1.00	0.00	H
ATOM	397	CB	TYR	25	18.921	9.715	8.113	1.00	0.00	C
ATOM	398	HB2	TYR	25	18.056	9.146	8.455	1.00	0.00	H
ATOM	399	HB3	TYR	25	19.728	9.035	8.387	1.00	0.00	H
ATOM	400	CG	TYR	25	18.740	9.766	6.616	1.00	0.00	C
ATOM	401	CD1	TYR	25	19.804	9.789	5.727	1.00	0.00	C
ATOM	402	HD1	TYR	25	20.739	9.294	5.946	1.00	0.00	H
ATOM	403	CE1	TYR	25	19.629	10.185	4.404	1.00	0.00	C
ATOM	404	HE1	TYR	25	20.480	10.271	3.745	1.00	0.00	H
ATOM	405	CZ	TYR	25	18.324	10.524	4.032	1.00	0.00	C
ATOM	406	OH	TYR	25	18.165	10.761	2.697	1.00	0.00	O
ATOM	407	HH	TYR	25	18.829	10.434	2.085	1.00	0.00	H
ATOM	408	CE2	TYR	25	17.185	10.520	4.856	1.00	0.00	C
ATOM	409	HE2	TYR	25	16.224	10.833	4.472	1.00	0.00	H

ATOM	410	CD2	TYR	25	17.390	10.114	6.167	1.00	0.00	C
ATOM	411	HD2	TYR	25	16.560	9.937	6.835	1.00	0.00	H
ATOM	412	C	TYR	25	20.620	11.562	8.753	1.00	0.00	C
ATOM	413	O	TYR	25	20.810	12.519	7.942	1.00	0.00	O
ATOM	414	N	TYR	26	21.590	10.990	9.506	1.00	0.00	N
ATOM	415	H	TYR	26	21.342	10.221	10.111	1.00	0.00	H
ATOM	416	CA	TYR	26	22.965	11.457	9.410	1.00	0.00	C
ATOM	417	HA	TYR	26	22.887	12.544	9.445	1.00	0.00	H
ATOM	418	CB	TYR	26	23.707	11.054	10.672	1.00	0.00	C
ATOM	419	HB2	TYR	26	23.553	9.977	10.726	1.00	0.00	H
ATOM	420	HB3	TYR	26	23.319	11.583	11.543	1.00	0.00	H
ATOM	421	CG	TYR	26	25.189	11.369	10.666	1.00	0.00	C
ATOM	422	CD1	TYR	26	26.101	10.289	10.840	1.00	0.00	C
ATOM	423	HD1	TYR	26	25.725	9.281	10.743	1.00	0.00	H
ATOM	424	CE1	TYR	26	27.512	10.522	11.075	1.00	0.00	C
ATOM	425	HE1	TYR	26	28.193	9.692	11.193	1.00	0.00	H
ATOM	426	CZ	TYR	26	27.897	11.823	11.255	1.00	0.00	C
ATOM	427	OH	TYR	26	29.210	12.123	11.298	1.00	0.00	O
ATOM	428	HH	TYR	26	29.318	13.055	11.098	1.00	0.00	H
ATOM	429	CE2	TYR	26	26.954	12.895	11.230	1.00	0.00	C
ATOM	430	HE2	TYR	26	27.310	13.891	11.447	1.00	0.00	H
ATOM	431	CD2	TYR	26	25.638	12.680	10.899	1.00	0.00	C
ATOM	432	HD2	TYR	26	24.968	13.526	10.855	1.00	0.00	H
ATOM	433	C	TYR	26	23.558	10.871	8.086	1.00	0.00	C
ATOM	434	O	TYR	26	23.323	9.665	7.739	1.00	0.00	O
ATOM	435	N	ARG	27	24.325	11.658	7.278	1.00	0.00	N
ATOM	436	H	ARG	27	24.384	12.630	7.544	1.00	0.00	H
ATOM	437	CA	ARG	27	24.818	11.209	6.003	1.00	0.00	C
ATOM	438	HA	ARG	27	24.203	10.366	5.686	1.00	0.00	H
ATOM	439	CB	ARG	27	24.623	12.346	4.965	1.00	0.00	C
ATOM	440	HB2	ARG	27	25.095	12.291	3.984	1.00	0.00	H
ATOM	441	HB3	ARG	27	24.987	13.213	5.516	1.00	0.00	H
ATOM	442	CG	ARG	27	23.079	12.650	4.578	1.00	0.00	C
ATOM	443	HG2	ARG	27	22.617	13.052	5.480	1.00	0.00	H
ATOM	444	HG3	ARG	27	22.672	11.668	4.335	1.00	0.00	H
ATOM	445	CD	ARG	27	22.921	13.657	3.469	1.00	0.00	C
ATOM	446	HD2	ARG	27	23.617	13.327	2.699	1.00	0.00	H
ATOM	447	HD3	ARG	27	23.051	14.656	3.886	1.00	0.00	H
ATOM	448	NE	ARG	27	21.549	13.368	2.931	1.00	0.00	N
ATOM	449	HE	ARG	27	21.551	12.864	2.056	1.00	0.00	H
ATOM	450	CZ	ARG	27	20.329	13.764	3.442	1.00	0.00	C
ATOM	451	NH1	ARG	27	20.094	14.311	4.621	1.00	0.00	N
ATOM	452	HH11	ARG	27	20.869	14.408	5.262	1.00	0.00	H
ATOM	453	HH12	ARG	27	19.172	14.501	4.987	1.00	0.00	H
ATOM	454	NH2	ARG	27	19.281	13.612	2.750	1.00	0.00	N
ATOM	455	HH21	ARG	27	19.328	13.025	1.930	1.00	0.00	H
ATOM	456	HH22	ARG	27	18.425	14.118	2.927	1.00	0.00	H
ATOM	457	C	ARG	27	26.296	10.798	6.107	1.00	0.00	C
ATOM	458	O	ARG	27	27.242	11.458	6.514	1.00	0.00	O
ATOM	459	N	GLU	28	26.499	9.565	5.595	1.00	0.00	N
ATOM	460	H	GLU	28	25.672	8.985	5.548	1.00	0.00	H
ATOM	461	CA	GLU	28	27.870	9.015	5.367	1.00	0.00	C
ATOM	462	HA	GLU	28	28.569	9.852	5.337	1.00	0.00	H
ATOM	463	CB	GLU	28	28.383	8.180	6.448	1.00	0.00	C
ATOM	464	HB2	GLU	28	29.377	7.821	6.181	1.00	0.00	H
ATOM	465	HB3	GLU	28	27.620	7.413	6.574	1.00	0.00	H
ATOM	466	CG	GLU	28	28.715	8.820	7.787	1.00	0.00	C
ATOM	467	HG2	GLU	28	27.772	9.100	8.258	1.00	0.00	H
ATOM	468	HG3	GLU	28	29.260	9.741	7.578	1.00	0.00	H
ATOM	469	CD	GLU	28	29.495	7.902	8.758	1.00	0.00	C
ATOM	470	OE1	GLU	28	30.362	8.258	9.613	1.00	0.00	O
ATOM	471	OE2	GLU	28	29.243	6.676	8.764	1.00	0.00	O
ATOM	472	C	GLU	28	27.907	8.249	4.024	1.00	0.00	C

ATOM	473	O	GLU	28	28.517	7.202	3.870	1.00	0.00	O
ATOM	474	N	ASN	29	27.141	8.755	3.030	1.00	0.00	N
ATOM	475	H	ASN	29	26.587	9.590	3.153	1.00	0.00	H
ATOM	476	CA	ASN	29	27.002	8.054	1.744	1.00	0.00	C
ATOM	477	HA	ASN	29	26.740	7.012	1.923	1.00	0.00	H
ATOM	478	CB	ASN	29	25.929	8.777	0.867	1.00	0.00	C
ATOM	479	HB2	ASN	29	24.935	8.975	1.269	1.00	0.00	H
ATOM	480	HB3	ASN	29	25.857	8.119	0.002	1.00	0.00	H
ATOM	481	CG	ASN	29	26.400	10.128	0.239	1.00	0.00	C
ATOM	482	OD1	ASN	29	27.342	10.791	0.597	1.00	0.00	O
ATOM	483	ND2	ASN	29	25.616	10.677	-0.717	1.00	0.00	N
ATOM	484	HD21	ASN	29	25.844	11.609	-1.033	1.00	0.00	H
ATOM	485	HD22	ASN	29	24.721	10.249	-0.907	1.00	0.00	H
ATOM	486	C	ASN	29	28.238	7.990	0.808	1.00	0.00	C
ATOM	487	O	ASN	29	28.115	7.449	-0.307	1.00	0.00	O
ATOM	488	N	MET	30	29.416	8.531	1.196	1.00	0.00	N
ATOM	489	H	MET	30	29.508	9.086	2.034	1.00	0.00	H
ATOM	490	CA	MET	30	30.609	8.350	0.361	1.00	0.00	C
ATOM	491	HA	MET	30	30.401	8.748	-0.632	1.00	0.00	H
ATOM	492	CB	MET	30	31.686	9.348	0.844	1.00	0.00	C
ATOM	493	HB2	MET	30	31.298	10.368	0.848	1.00	0.00	H
ATOM	494	HB3	MET	30	32.402	9.217	0.033	1.00	0.00	H
ATOM	495	CG	MET	30	32.362	8.971	2.174	1.00	0.00	C
ATOM	496	HG2	MET	30	33.224	9.602	2.389	1.00	0.00	H
ATOM	497	HG3	MET	30	32.681	7.949	1.969	1.00	0.00	H
ATOM	498	SD	MET	30	31.401	8.710	3.756	1.00	0.00	S
ATOM	499	CE	MET	30	30.940	10.427	3.999	1.00	0.00	C
ATOM	500	HE1	MET	30	30.276	10.812	3.224	1.00	0.00	H
ATOM	501	HE2	MET	30	30.548	10.559	5.007	1.00	0.00	H
ATOM	502	HE3	MET	30	31.835	11.015	3.797	1.00	0.00	H
ATOM	503	C	MET	30	31.053	6.852	0.284	1.00	0.00	C
ATOM	504	O	MET	30	31.347	6.463	-0.843	1.00	0.00	O
ATOM	505	N	TYR	31	30.890	6.039	1.302	1.00	0.00	N
ATOM	506	H	TYR	31	30.522	6.429	2.157	1.00	0.00	H
ATOM	507	CA	TYR	31	31.077	4.614	1.225	1.00	0.00	C
ATOM	508	HA	TYR	31	32.105	4.402	0.931	1.00	0.00	H
ATOM	509	CB	TYR	31	30.880	3.874	2.526	1.00	0.00	C
ATOM	510	HB2	TYR	31	30.752	2.802	2.375	1.00	0.00	H
ATOM	511	HB3	TYR	31	29.910	4.250	2.851	1.00	0.00	H
ATOM	512	CG	TYR	31	32.010	4.157	3.432	1.00	0.00	C
ATOM	513	CD1	TYR	31	31.814	5.069	4.469	1.00	0.00	C
ATOM	514	HD1	TYR	31	30.830	5.511	4.500	1.00	0.00	H
ATOM	515	CE1	TYR	31	32.847	5.344	5.361	1.00	0.00	C
ATOM	516	HE1	TYR	31	32.748	6.211	5.997	1.00	0.00	H
ATOM	517	CZ	TYR	31	34.051	4.574	5.291	1.00	0.00	C
ATOM	518	OH	TYR	31	35.067	4.713	6.158	1.00	0.00	O
ATOM	519	HH	TYR	31	35.782	4.126	5.903	1.00	0.00	H
ATOM	520	CE2	TYR	31	34.155	3.727	4.185	1.00	0.00	C
ATOM	521	HE2	TYR	31	35.139	3.378	3.908	1.00	0.00	H
ATOM	522	CD2	TYR	31	33.223	3.433	3.275	1.00	0.00	C
ATOM	523	HD2	TYR	31	33.288	2.684	2.501	1.00	0.00	H
ATOM	524	C	TYR	31	30.124	3.937	0.224	1.00	0.00	C
ATOM	525	O	TYR	31	28.959	4.123	0.210	1.00	0.00	O
ATOM	526	N	ARG	32	30.636	3.097	-0.718	1.00	0.00	N
ATOM	527	H	ARG	32	31.607	2.830	-0.650	1.00	0.00	H
ATOM	528	CA	ARG	32	29.869	2.294	-1.756	1.00	0.00	C
ATOM	529	HA	ARG	32	29.077	2.915	-2.175	1.00	0.00	H
ATOM	530	CB	ARG	32	30.806	1.692	-2.902	1.00	0.00	C
ATOM	531	HB2	ARG	32	30.400	1.492	-3.893	1.00	0.00	H
ATOM	532	HB3	ARG	32	31.206	0.743	-2.547	1.00	0.00	H
ATOM	533	CG	ARG	32	32.009	2.545	-3.283	1.00	0.00	C
ATOM	534	HG2	ARG	32	32.674	2.026	-3.973	1.00	0.00	H
ATOM	535	HG3	ARG	32	32.531	2.605	-2.327	1.00	0.00	H

```
ATOM    536  CD  ARG    32      31.913   3.916  -3.852  1.00  0.00           C
ATOM    537  HD2 ARG    32      31.675   3.940  -4.916  1.00  0.00           H
ATOM    538  HD3 ARG    32      32.898   4.332  -3.641  1.00  0.00           H
ATOM    539  NE  ARG    32      31.010   4.909  -3.235  1.00  0.00           N
ATOM    540  HE  ARG    32      31.357   5.242  -2.346  1.00  0.00           H
ATOM    541  CZ  ARG    32      29.736   5.243  -3.469  1.00  0.00           C
ATOM    542  NH1 ARG    32      29.071   4.762  -4.469  1.00  0.00           N
ATOM    543 HH11 ARG    32      29.574   4.126  -5.071  1.00  0.00           H
ATOM    544 HH12 ARG    32      28.171   5.180  -4.657  1.00  0.00           H
ATOM    545  NH2 ARG    32      29.203   6.204  -2.735  1.00  0.00           N
ATOM    546 HH21 ARG    32      29.780   6.593  -2.003  1.00  0.00           H
ATOM    547 HH22 ARG    32      28.372   6.705  -3.016  1.00  0.00           H
ATOM    548  C   ARG    32      29.127   1.136  -1.183  1.00  0.00           C
ATOM    549  O   ARG    32      29.263   0.928   0.012  1.00  0.00           O
ATOM    550  N   TYR    33      28.324   0.402  -2.003  1.00  0.00           N
ATOM    551  H   TYR    33      28.072   0.787  -2.901  1.00  0.00           H
ATOM    552  CA  TYR    33      27.662  -0.865  -1.583  1.00  0.00           C
ATOM    553  HA  TYR    33      27.830  -1.036  -0.520  1.00  0.00           H
ATOM    554  CB  TYR    33      26.180  -0.651  -1.776  1.00  0.00           C
ATOM    555  HB2 TYR    33      25.957  -0.318  -2.790  1.00  0.00           H
ATOM    556  HB3 TYR    33      25.878   0.153  -1.106  1.00  0.00           H
ATOM    557  CG  TYR    33      25.286  -1.827  -1.664  1.00  0.00           C
ATOM    558  CD1 TYR    33      24.823  -2.526  -2.745  1.00  0.00           C
ATOM    559  HD1 TYR    33      25.139  -2.303  -3.754  1.00  0.00           H
ATOM    560  CE1 TYR    33      24.009  -3.622  -2.664  1.00  0.00           C
ATOM    561  HE1 TYR    33      23.755  -4.067  -3.615  1.00  0.00           H
ATOM    562  CZ  TYR    33      23.513  -4.027  -1.429  1.00  0.00           C
ATOM    563  OH  TYR    33      22.648  -5.078  -1.374  1.00  0.00           O
ATOM    564  HH  TYR    33      22.661  -5.586  -2.189  1.00  0.00           H
ATOM    565  CE2 TYR    33      23.996  -3.352  -0.261  1.00  0.00           C
ATOM    566  HE2 TYR    33      23.686  -3.728   0.703  1.00  0.00           H
ATOM    567  CD2 TYR    33      24.836  -2.234  -0.398  1.00  0.00           C
ATOM    568  HD2 TYR    33      25.090  -1.577   0.421  1.00  0.00           H
ATOM    569  C   TYR    33      28.248  -2.020  -2.440  1.00  0.00           C
ATOM    570  O   TYR    33      28.409  -2.048  -3.717  1.00  0.00           O
ATOM    571  N   NME    34      28.579  -3.154  -1.734  1.00  0.00           N
ATOM    572  H   NME    34      28.239  -3.177  -0.783  1.00  0.00           H
ATOM    573  CH3 NME    34      28.992  -4.481  -2.252  1.00  0.00           C
ATOM    574 HH31 NME    34      29.043  -5.184  -1.421  1.00  0.00           H
ATOM    575 HH32 NME    34      28.166  -4.813  -2.881  1.00  0.00           H
ATOM    576 HH33 NME    34      29.908  -4.355  -2.828  1.00  0.00           H
TER     577      NME    34
END
```

Cluster 3:

```
ATOM      1 HH31 ACE     1      19.735   1.605 -13.135  1.00  0.00           H
ATOM      2  CH3 ACE     1      19.748   2.634 -12.777  1.00  0.00           C
ATOM      3 HH32 ACE     1      19.792   3.187 -13.715  1.00  0.00           H
ATOM      4 HH33 ACE     1      20.576   2.885 -12.113  1.00  0.00           H
ATOM      5  C   ACE     1      18.389   3.049 -12.210  1.00  0.00           C
ATOM      6  O   ACE     1      17.566   3.513 -13.010  1.00  0.00           O
ATOM      7  N   ASN     2      18.151   2.729 -10.888  1.00  0.00           N
ATOM      8  H   ASN     2      18.944   2.506 -10.304  1.00  0.00           H
ATOM      9  CA  ASN     2      16.989   2.979 -10.090  1.00  0.00           C
ATOM     10  HA  ASN     2      16.391   3.824 -10.430  1.00  0.00           H
ATOM     11  CB  ASN     2      16.072   1.774 -10.002  1.00  0.00           C
ATOM     12  HB2 ASN     2      15.705   1.528 -10.998  1.00  0.00           H
ATOM     13  HB3 ASN     2      15.300   2.117  -9.312  1.00  0.00           H
ATOM     14  CG  ASN     2      16.744   0.506  -9.414  1.00  0.00           C
ATOM     15  OD1 ASN     2      16.718   0.224  -8.200  1.00  0.00           O
```

ATOM	16	ND2	ASN	2	17.481	-0.277	-10.164	1.00	0.00	N
ATOM	17	HD21	ASN	2	18.074	-1.022	-9.829	1.00	0.00	H
ATOM	18	HD22	ASN	2	17.706	0.085	-11.080	1.00	0.00	H
ATOM	19	C	ASN	2	17.398	3.482	-8.717	1.00	0.00	C
ATOM	20	O	ASN	2	18.555	3.307	-8.285	1.00	0.00	O
ATOM	21	N	ASP	3	16.394	4.003	-7.952	1.00	0.00	N
ATOM	22	H	ASP	3	15.578	4.312	-8.459	1.00	0.00	H
ATOM	23	CA	ASP	3	16.604	4.539	-6.568	1.00	0.00	C
ATOM	24	HA	ASP	3	17.629	4.908	-6.590	1.00	0.00	H
ATOM	25	CB	ASP	3	15.562	5.687	-6.479	1.00	0.00	C
ATOM	26	HB2	ASP	3	14.543	5.305	-6.541	1.00	0.00	H
ATOM	27	HB3	ASP	3	15.784	6.412	-7.262	1.00	0.00	H
ATOM	28	CG	ASP	3	15.685	6.430	-5.177	1.00	0.00	C
ATOM	29	OD1	ASP	3	15.030	6.070	-4.154	1.00	0.00	O
ATOM	30	OD2	ASP	3	16.499	7.368	-5.092	1.00	0.00	O
ATOM	31	C	ASP	3	16.442	3.465	-5.486	1.00	0.00	C
ATOM	32	O	ASP	3	16.863	3.620	-4.318	1.00	0.00	O
ATOM	33	N	TYR	4	15.834	2.345	-5.943	1.00	0.00	N
ATOM	34	H	TYR	4	15.703	2.328	-6.944	1.00	0.00	H
ATOM	35	CA	TYR	4	15.324	1.285	-5.094	1.00	0.00	C
ATOM	36	HA	TYR	4	14.878	1.773	-4.228	1.00	0.00	H
ATOM	37	CB	TYR	4	14.310	0.462	-5.866	1.00	0.00	C
ATOM	38	HB2	TYR	4	13.794	-0.254	-5.225	1.00	0.00	H
ATOM	39	HB3	TYR	4	14.935	-0.122	-6.541	1.00	0.00	H
ATOM	40	CG	TYR	4	13.301	1.248	-6.701	1.00	0.00	C
ATOM	41	CD1	TYR	4	13.184	1.043	-8.111	1.00	0.00	C
ATOM	42	HD1	TYR	4	13.716	0.226	-8.577	1.00	0.00	H
ATOM	43	CE1	TYR	4	12.306	1.880	-8.876	1.00	0.00	C
ATOM	44	HE1	TYR	4	12.096	1.628	-9.905	1.00	0.00	H
ATOM	45	CZ	TYR	4	11.432	2.733	-8.127	1.00	0.00	C
ATOM	46	OH	TYR	4	10.553	3.581	-8.762	1.00	0.00	O
ATOM	47	HH	TYR	4	10.063	4.127	-8.143	1.00	0.00	H
ATOM	48	CE2	TYR	4	11.685	2.931	-6.748	1.00	0.00	C
ATOM	49	HE2	TYR	4	11.141	3.743	-6.289	1.00	0.00	H
ATOM	50	CD2	TYR	4	12.644	2.260	-6.047	1.00	0.00	C
ATOM	51	HD2	TYR	4	12.671	2.353	-4.972	1.00	0.00	H
ATOM	52	C	TYR	4	16.449	0.477	-4.434	1.00	0.00	C
ATOM	53	O	TYR	4	16.331	0.162	-3.207	1.00	0.00	O
ATOM	54	N	GLU	5	17.611	0.310	-5.050	1.00	0.00	N
ATOM	55	H	GLU	5	17.685	0.559	-6.026	1.00	0.00	H
ATOM	56	CA	GLU	5	18.770	-0.378	-4.450	1.00	0.00	C
ATOM	57	HA	GLU	5	18.288	-1.253	-4.013	1.00	0.00	H
ATOM	58	CB	GLU	5	19.733	-0.739	-5.583	1.00	0.00	C
ATOM	59	HB2	GLU	5	19.077	-1.254	-6.286	1.00	0.00	H
ATOM	60	HB3	GLU	5	20.490	-1.455	-5.263	1.00	0.00	H
ATOM	61	CG	GLU	5	20.481	0.504	-6.204	1.00	0.00	C
ATOM	62	HG2	GLU	5	21.038	0.976	-5.395	1.00	0.00	H
ATOM	63	HG3	GLU	5	19.715	1.186	-6.573	1.00	0.00	H
ATOM	64	CD	GLU	5	21.461	0.249	-7.329	1.00	0.00	C
ATOM	65	OE1	GLU	5	21.046	-0.028	-8.476	1.00	0.00	O
ATOM	66	OE2	GLU	5	22.685	0.230	-7.025	1.00	0.00	O
ATOM	67	C	GLU	5	19.405	0.402	-3.232	1.00	0.00	C
ATOM	68	O	GLU	5	19.786	-0.140	-2.240	1.00	0.00	O
ATOM	69	N	ASP	6	19.417	1.704	-3.299	1.00	0.00	N
ATOM	70	H	ASP	6	19.018	2.148	-4.113	1.00	0.00	H
ATOM	71	CA	ASP	6	19.995	2.611	-2.271	1.00	0.00	C
ATOM	72	HA	ASP	6	21.007	2.281	-2.036	1.00	0.00	H
ATOM	73	CB	ASP	6	20.024	4.075	-2.773	1.00	0.00	C
ATOM	74	HB2	ASP	6	19.076	4.341	-3.241	1.00	0.00	H
ATOM	75	HB3	ASP	6	20.723	3.930	-3.596	1.00	0.00	H
ATOM	76	CG	ASP	6	20.520	5.176	-1.830	1.00	0.00	C
ATOM	77	OD1	ASP	6	19.786	5.466	-0.843	1.00	0.00	O
ATOM	78	OD2	ASP	6	21.590	5.850	-2.127	1.00	0.00	O

ATOM	79	C	ASP	6	19.333	2.419	-0.933	1.00	0.00	C
ATOM	80	O	ASP	6	19.860	2.470	0.141	1.00	0.00	O
ATOM	81	N	ARG	7	18.001	2.118	-0.963	1.00	0.00	N
ATOM	82	H	ARG	7	17.582	2.043	-1.879	1.00	0.00	H
ATOM	83	CA	ARG	7	17.102	1.793	0.186	1.00	0.00	C
ATOM	84	HA	ARG	7	17.331	2.483	0.998	1.00	0.00	H
ATOM	85	CB	ARG	7	15.730	1.939	-0.337	1.00	0.00	C
ATOM	86	HB2	ARG	7	14.956	1.744	0.405	1.00	0.00	H
ATOM	87	HB3	ARG	7	15.653	1.118	-1.050	1.00	0.00	H
ATOM	88	CG	ARG	7	15.369	3.211	-1.120	1.00	0.00	C
ATOM	89	HG2	ARG	7	15.893	3.417	-2.053	1.00	0.00	H
ATOM	90	HG3	ARG	7	15.604	3.939	-0.343	1.00	0.00	H
ATOM	91	CD	ARG	7	13.962	3.248	-1.488	1.00	0.00	C
ATOM	92	HD2	ARG	7	13.261	3.222	-0.655	1.00	0.00	H
ATOM	93	HD3	ARG	7	13.821	2.360	-2.105	1.00	0.00	H
ATOM	94	NE	ARG	7	13.661	4.427	-2.334	1.00	0.00	N
ATOM	95	HE	ARG	7	14.392	4.911	-2.836	1.00	0.00	H
ATOM	96	CZ	ARG	7	12.520	5.012	-2.434	1.00	0.00	C
ATOM	97	NH1	ARG	7	11.528	4.575	-1.820	1.00	0.00	N
ATOM	98	HH11	ARG	7	11.670	3.759	-1.243	1.00	0.00	H
ATOM	99	HH12	ARG	7	10.626	4.981	-2.023	1.00	0.00	H
ATOM	100	NH2	ARG	7	12.317	6.015	-3.224	1.00	0.00	N
ATOM	101	HH21	ARG	7	13.152	6.418	-3.626	1.00	0.00	H
ATOM	102	HH22	ARG	7	11.493	6.595	-3.163	1.00	0.00	H
ATOM	103	C	ARG	7	17.487	0.492	0.839	1.00	0.00	C
ATOM	104	O	ARG	7	17.108	0.391	2.023	1.00	0.00	O
ATOM	105	N	TYR	8	18.330	-0.375	0.255	1.00	0.00	N
ATOM	106	H	TYR	8	18.602	-0.227	-0.706	1.00	0.00	H
ATOM	107	CA	TYR	8	18.823	-1.568	0.838	1.00	0.00	C
ATOM	108	HA	TYR	8	18.226	-1.841	1.708	1.00	0.00	H
ATOM	109	CB	TYR	8	18.854	-2.729	-0.149	1.00	0.00	C
ATOM	110	HB2	TYR	8	19.465	-2.516	-1.026	1.00	0.00	H
ATOM	111	HB3	TYR	8	17.867	-2.633	-0.600	1.00	0.00	H
ATOM	112	CG	TYR	8	19.116	-4.100	0.361	1.00	0.00	C
ATOM	113	CD1	TYR	8	20.379	-4.384	0.756	1.00	0.00	C
ATOM	114	HD1	TYR	8	21.218	-3.727	0.579	1.00	0.00	H
ATOM	115	CE1	TYR	8	20.574	-5.648	1.371	1.00	0.00	C
ATOM	116	HE1	TYR	8	21.587	-5.913	1.637	1.00	0.00	H
ATOM	117	CZ	TYR	8	19.530	-6.586	1.556	1.00	0.00	C
ATOM	118	OH	TYR	8	19.785	-7.813	2.006	1.00	0.00	O
ATOM	119	HH	TYR	8	20.735	-7.850	2.139	1.00	0.00	H
ATOM	120	CE2	TYR	8	18.268	-6.305	1.046	1.00	0.00	C
ATOM	121	HE2	TYR	8	17.503	-7.060	1.143	1.00	0.00	H
ATOM	122	CD2	TYR	8	17.997	-4.989	0.505	1.00	0.00	C
ATOM	123	HD2	TYR	8	17.022	-4.721	0.124	1.00	0.00	H
ATOM	124	C	TYR	8	20.226	-1.303	1.368	1.00	0.00	C
ATOM	125	O	TYR	8	20.497	-1.603	2.523	1.00	0.00	O
ATOM	126	N	TYR	9	21.230	-0.779	0.655	1.00	0.00	N
ATOM	127	H	TYR	9	21.068	-0.688	-0.338	1.00	0.00	H
ATOM	128	CA	TYR	9	22.633	-0.544	1.063	1.00	0.00	C
ATOM	129	HA	TYR	9	22.829	-1.443	1.647	1.00	0.00	H
ATOM	130	CB	TYR	9	23.588	-0.620	-0.118	1.00	0.00	C
ATOM	131	HB2	TYR	9	24.589	-0.273	0.138	1.00	0.00	H
ATOM	132	HB3	TYR	9	23.160	0.114	-0.801	1.00	0.00	H
ATOM	133	CG	TYR	9	23.697	-2.040	-0.647	1.00	0.00	C
ATOM	134	CD1	TYR	9	22.895	-2.575	-1.636	1.00	0.00	C
ATOM	135	HD1	TYR	9	22.148	-1.991	-2.152	1.00	0.00	H
ATOM	136	CE1	TYR	9	23.126	-3.904	-2.112	1.00	0.00	C
ATOM	137	HE1	TYR	9	22.481	-4.235	-2.913	1.00	0.00	H
ATOM	138	CZ	TYR	9	24.142	-4.754	-1.635	1.00	0.00	C
ATOM	139	OH	TYR	9	24.408	-5.947	-2.162	1.00	0.00	O
ATOM	140	HH	TYR	9	23.879	-6.268	-2.896	1.00	0.00	H
ATOM	141	CE2	TYR	9	24.911	-4.230	-0.564	1.00	0.00	C

ATOM	142	HE2	TYR	9	25.640	-4.857	-0.072	1.00	0.00	H
ATOM	143	CD2	TYR	9	24.737	-2.902	-0.151	1.00	0.00	C
ATOM	144	HD2	TYR	9	25.397	-2.529	0.619	1.00	0.00	H
ATOM	145	C	TYR	9	22.780	0.752	1.717	1.00	0.00	C
ATOM	146	O	TYR	9	23.420	1.680	1.179	1.00	0.00	O
ATOM	147	N	ARG	10	22.120	0.861	2.874	1.00	0.00	N
ATOM	148	H	ARG	10	21.670	0.041	3.255	1.00	0.00	H
ATOM	149	CA	ARG	10	22.192	2.074	3.665	1.00	0.00	C
ATOM	150	HA	ARG	10	22.467	2.890	2.996	1.00	0.00	H
ATOM	151	CB	ARG	10	20.785	2.327	4.124	1.00	0.00	C
ATOM	152	HB2	ARG	10	20.455	1.596	4.863	1.00	0.00	H
ATOM	153	HB3	ARG	10	20.026	2.362	3.343	1.00	0.00	H
ATOM	154	CG	ARG	10	20.677	3.681	4.838	1.00	0.00	C
ATOM	155	HG2	ARG	10	21.385	3.773	5.661	1.00	0.00	H
ATOM	156	HG3	ARG	10	19.664	3.642	5.240	1.00	0.00	H
ATOM	157	CD	ARG	10	20.847	4.869	3.826	1.00	0.00	C
ATOM	158	HD2	ARG	10	21.879	4.919	3.481	1.00	0.00	H
ATOM	159	HD3	ARG	10	20.748	5.795	4.392	1.00	0.00	H
ATOM	160	NE	ARG	10	19.929	4.858	2.663	1.00	0.00	N
ATOM	161	HE	ARG	10	20.397	4.822	1.769	1.00	0.00	H
ATOM	162	CZ	ARG	10	18.663	5.111	2.611	1.00	0.00	C
ATOM	163	NH1	ARG	10	17.933	5.311	3.634	1.00	0.00	N
ATOM	164	HH11	ARG	10	18.437	5.363	4.508	1.00	0.00	H
ATOM	165	HH12	ARG	10	16.948	5.484	3.490	1.00	0.00	H
ATOM	166	NH2	ARG	10	18.038	5.183	1.473	1.00	0.00	N
ATOM	167	HH21	ARG	10	18.472	5.338	0.574	1.00	0.00	H
ATOM	168	HH22	ARG	10	17.131	5.620	1.559	1.00	0.00	H
ATOM	169	C	ARG	10	23.246	2.024	4.762	1.00	0.00	C
ATOM	170	O	ARG	10	24.028	2.950	4.891	1.00	0.00	O
ATOM	171	N	GLU	11	23.313	0.994	5.587	1.00	0.00	N
ATOM	172	H	GLU	11	22.650	0.233	5.624	1.00	0.00	H
ATOM	173	CA	GLU	11	24.300	0.791	6.728	1.00	0.00	C
ATOM	174	HA	GLU	11	24.194	1.648	7.393	1.00	0.00	H
ATOM	175	CB	GLU	11	23.999	-0.429	7.547	1.00	0.00	C
ATOM	176	HB2	GLU	11	24.310	-0.241	8.575	1.00	0.00	H
ATOM	177	HB3	GLU	11	24.625	-1.190	7.082	1.00	0.00	H
ATOM	178	CG	GLU	11	22.501	-0.847	7.733	1.00	0.00	C
ATOM	179	HG2	GLU	11	22.342	-1.630	8.475	1.00	0.00	H
ATOM	180	HG3	GLU	11	22.067	-1.210	6.801	1.00	0.00	H
ATOM	181	CD	GLU	11	21.665	0.344	8.252	1.00	0.00	C
ATOM	182	OE1	GLU	11	20.558	0.522	7.701	1.00	0.00	O
ATOM	183	OE2	GLU	11	22.090	1.095	9.180	1.00	0.00	O
ATOM	184	C	GLU	11	25.750	0.769	6.238	1.00	0.00	C
ATOM	185	O	GLU	11	26.086	0.329	5.073	1.00	0.00	O
ATOM	186	N	ASN	12	26.708	1.061	7.112	1.00	0.00	N
ATOM	187	H	ASN	12	26.469	1.562	7.955	1.00	0.00	H
ATOM	188	CA	ASN	12	28.148	1.191	6.724	1.00	0.00	C
ATOM	189	HA	ASN	12	28.218	1.776	5.807	1.00	0.00	H
ATOM	190	CB	ASN	12	28.859	1.909	7.914	1.00	0.00	C
ATOM	191	HB2	ASN	12	28.543	1.499	8.873	1.00	0.00	H
ATOM	192	HB3	ASN	12	28.566	2.948	7.765	1.00	0.00	H
ATOM	193	CG	ASN	12	30.349	1.860	7.775	1.00	0.00	C
ATOM	194	OD1	ASN	12	30.994	2.321	6.904	1.00	0.00	O
ATOM	195	ND2	ASN	12	30.994	1.273	8.727	1.00	0.00	N
ATOM	196	HD21	ASN	12	32.003	1.265	8.700	1.00	0.00	H
ATOM	197	HD22	ASN	12	30.536	0.934	9.561	1.00	0.00	H
ATOM	198	C	ASN	12	28.784	-0.190	6.598	1.00	0.00	C
ATOM	199	O	ASN	12	29.754	-0.283	5.862	1.00	0.00	O
ATOM	200	N	MET	13	28.299	-1.244	7.240	1.00	0.00	N
ATOM	201	H	MET	13	27.450	-1.014	7.736	1.00	0.00	H
ATOM	202	CA	MET	13	28.860	-2.598	7.353	1.00	0.00	C
ATOM	203	HA	MET	13	29.950	-2.587	7.390	1.00	0.00	H
ATOM	204	CB	MET	13	28.428	-3.329	8.611	1.00	0.00	C

ATOM	205	HB2	MET	13	28.598	-4.393	8.442	1.00	0.00	H
ATOM	206	HB3	MET	13	27.359	-3.114	8.627	1.00	0.00	H
ATOM	207	CG	MET	13	29.184	-2.888	9.898	1.00	0.00	C
ATOM	208	HG2	MET	13	30.256	-3.034	10.028	1.00	0.00	H
ATOM	209	HG3	MET	13	28.615	-3.487	10.608	1.00	0.00	H
ATOM	210	SD	MET	13	28.898	-1.144	10.448	1.00	0.00	S
ATOM	211	CE	MET	13	30.086	-0.985	11.818	1.00	0.00	C
ATOM	212	HE1	MET	13	30.070	0.018	12.244	1.00	0.00	H
ATOM	213	HE2	MET	13	29.804	-1.798	12.487	1.00	0.00	H
ATOM	214	HE3	MET	13	31.073	-1.170	11.395	1.00	0.00	H
ATOM	215	C	MET	13	28.486	-3.451	6.117	1.00	0.00	C
ATOM	216	O	MET	13	29.044	-4.530	5.916	1.00	0.00	O
ATOM	217	N	TYR	14	27.501	-2.980	5.314	1.00	0.00	N
ATOM	218	H	TYR	14	27.123	-2.104	5.645	1.00	0.00	H
ATOM	219	CA	TYR	14	26.853	-3.761	4.203	1.00	0.00	C
ATOM	220	HA	TYR	14	26.956	-4.842	4.299	1.00	0.00	H
ATOM	221	CB	TYR	14	25.376	-3.472	4.153	1.00	0.00	C
ATOM	222	HB2	TYR	14	24.833	-3.732	3.244	1.00	0.00	H
ATOM	223	HB3	TYR	14	25.353	-2.383	4.177	1.00	0.00	H
ATOM	224	CG	TYR	14	24.292	-4.028	5.228	1.00	0.00	C
ATOM	225	CD1	TYR	14	24.829	-4.593	6.449	1.00	0.00	C
ATOM	226	HD1	TYR	14	25.901	-4.705	6.515	1.00	0.00	H
ATOM	227	CE1	TYR	14	23.935	-5.022	7.475	1.00	0.00	C
ATOM	228	HE1	TYR	14	24.361	-5.435	8.377	1.00	0.00	H
ATOM	229	CZ	TYR	14	22.570	-4.946	7.282	1.00	0.00	C
ATOM	230	OH	TYR	14	21.790	-5.429	8.260	1.00	0.00	O
ATOM	231	HH	TYR	14	20.855	-5.422	8.041	1.00	0.00	H
ATOM	232	CE2	TYR	14	22.056	-4.311	6.208	1.00	0.00	C
ATOM	233	HE2	TYR	14	21.008	-4.144	6.009	1.00	0.00	H
ATOM	234	CD2	TYR	14	22.899	-3.827	5.229	1.00	0.00	C
ATOM	235	HD2	TYR	14	22.490	-3.227	4.429	1.00	0.00	H
ATOM	236	C	TYR	14	27.471	-3.406	2.836	1.00	0.00	C
ATOM	237	O	TYR	14	27.158	-4.089	1.862	1.00	0.00	O
ATOM	238	N	ARG	15	28.335	-2.366	2.796	1.00	0.00	N
ATOM	239	H	ARG	15	28.474	-1.819	3.633	1.00	0.00	H
ATOM	240	CA	ARG	15	29.042	-1.926	1.575	1.00	0.00	C
ATOM	241	HA	ARG	15	28.825	-2.452	0.645	1.00	0.00	H
ATOM	242	CB	ARG	15	28.821	-0.412	1.419	1.00	0.00	C
ATOM	243	HB2	ARG	15	29.081	-0.074	0.416	1.00	0.00	H
ATOM	244	HB3	ARG	15	29.470	0.119	2.115	1.00	0.00	H
ATOM	245	CG	ARG	15	27.452	0.165	1.586	1.00	0.00	C
ATOM	246	HG2	ARG	15	26.871	-0.205	2.431	1.00	0.00	H
ATOM	247	HG3	ARG	15	26.888	0.012	0.666	1.00	0.00	H
ATOM	248	CD	ARG	15	27.708	1.668	1.869	1.00	0.00	C
ATOM	249	HD2	ARG	15	28.601	2.116	1.433	1.00	0.00	H
ATOM	250	HD3	ARG	15	27.923	1.764	2.933	1.00	0.00	H
ATOM	251	NE	ARG	15	26.481	2.379	1.451	1.00	0.00	N
ATOM	252	HE	ARG	15	25.685	1.902	1.051	1.00	0.00	H
ATOM	253	CZ	ARG	15	26.210	3.696	1.633	1.00	0.00	C
ATOM	254	NH1	ARG	15	27.074	4.552	2.061	1.00	0.00	N
ATOM	255	HH11	ARG	15	28.066	4.363	2.068	1.00	0.00	H
ATOM	256	HH12	ARG	15	26.716	5.496	2.042	1.00	0.00	H
ATOM	257	NH2	ARG	15	24.996	4.083	1.569	1.00	0.00	N
ATOM	258	HH21	ARG	15	24.219	3.575	1.171	1.00	0.00	H
ATOM	259	HH22	ARG	15	24.782	5.016	1.892	1.00	0.00	H
ATOM	260	C	ARG	15	30.575	-2.241	1.691	1.00	0.00	C
ATOM	261	O	ARG	15	31.354	-1.724	0.883	1.00	0.00	O
ATOM	262	N	TYR	16	30.951	-3.023	2.703	1.00	0.00	N
ATOM	263	H	TYR	16	30.287	-3.316	3.405	1.00	0.00	H
ATOM	264	CA	TYR	16	32.357	-3.333	2.821	1.00	0.00	C
ATOM	265	HA	TYR	16	32.832	-3.255	1.843	1.00	0.00	H
ATOM	266	CB	TYR	16	32.911	-2.353	3.868	1.00	0.00	C
ATOM	267	HB2	TYR	16	32.484	-2.644	4.828	1.00	0.00	H

ATOM	268	HB3	TYR	16	32.396	-1.448	3.546	1.00	0.00	H
ATOM	269	CG	TYR	16	34.426	-2.127	3.908	1.00	0.00	C
ATOM	270	CD1	TYR	16	35.033	-1.161	3.013	1.00	0.00	C
ATOM	271	HD1	TYR	16	34.460	-0.531	2.349	1.00	0.00	H
ATOM	272	CE1	TYR	16	36.416	-1.016	2.984	1.00	0.00	C
ATOM	273	HE1	TYR	16	36.856	-0.252	2.360	1.00	0.00	H
ATOM	274	CZ	TYR	16	37.255	-1.750	3.854	1.00	0.00	C
ATOM	275	OH	TYR	16	38.571	-1.430	3.923	1.00	0.00	O
ATOM	276	HH	TYR	16	38.853	-0.758	3.298	1.00	0.00	H
ATOM	277	CE2	TYR	16	36.654	-2.827	4.650	1.00	0.00	C
ATOM	278	HE2	TYR	16	37.326	-3.421	5.252	1.00	0.00	H
ATOM	279	CD2	TYR	16	35.257	-2.904	4.680	1.00	0.00	C
ATOM	280	HD2	TYR	16	34.808	-3.696	5.262	1.00	0.00	H
ATOM	281	C	TYR	16	32.637	-4.833	3.192	1.00	0.00	C
ATOM	282	O	TYR	16	31.769	-5.407	3.838	1.00	0.00	O
ATOM	283	N	NME	17	33.876	-5.291	2.904	1.00	0.00	N
ATOM	284	H	NME	17	34.544	-4.654	2.493	1.00	0.00	H
ATOM	285	CH3	NME	17	34.309	-6.650	3.145	1.00	0.00	C
ATOM	286	HH31	NME	17	35.151	-6.569	3.832	1.00	0.00	H
ATOM	287	HH32	NME	17	33.525	-7.189	3.678	1.00	0.00	H
ATOM	288	HH33	NME	17	34.567	-7.240	2.266	1.00	0.00	H
TER	289		NME	17						
ATOM	289	HH31	ACE	18	12.680	9.233	9.656	1.00	0.00	H
ATOM	290	CH3	ACE	18	11.725	9.507	9.209	1.00	0.00	C
ATOM	291	HH32	ACE	18	11.466	10.505	9.561	1.00	0.00	H
ATOM	292	HH33	ACE	18	10.936	8.806	9.483	1.00	0.00	H
ATOM	293	C	ACE	18	11.845	9.397	7.753	1.00	0.00	C
ATOM	294	O	ACE	18	11.465	8.373	7.190	1.00	0.00	O
ATOM	295	N	ASN	19	12.345	10.387	7.029	1.00	0.00	N
ATOM	296	H	ASN	19	12.663	11.173	7.577	1.00	0.00	H
ATOM	297	CA	ASN	19	12.407	10.599	5.554	1.00	0.00	C
ATOM	298	HA	ASN	19	11.580	10.050	5.105	1.00	0.00	H
ATOM	299	CB	ASN	19	12.424	12.074	5.276	1.00	0.00	C
ATOM	300	HB2	ASN	19	13.308	12.489	5.760	1.00	0.00	H
ATOM	301	HB3	ASN	19	11.446	12.448	5.581	1.00	0.00	H
ATOM	302	CG	ASN	19	12.482	12.416	3.790	1.00	0.00	C
ATOM	303	OD1	ASN	19	12.257	11.649	2.933	1.00	0.00	O
ATOM	304	ND2	ASN	19	12.819	13.662	3.449	1.00	0.00	N
ATOM	305	HD21	ASN	19	12.710	14.017	2.509	1.00	0.00	H
ATOM	306	HD22	ASN	19	13.051	14.392	4.107	1.00	0.00	H
ATOM	307	C	ASN	19	13.682	9.937	4.932	1.00	0.00	C
ATOM	308	O	ASN	19	14.794	10.359	5.258	1.00	0.00	O
ATOM	309	N	ASP	20	13.492	8.953	4.039	1.00	0.00	N
ATOM	310	H	ASP	20	12.593	8.534	3.851	1.00	0.00	H
ATOM	311	CA	ASP	20	14.601	8.164	3.422	1.00	0.00	C
ATOM	312	HA	ASP	20	14.918	7.458	4.190	1.00	0.00	H
ATOM	313	CB	ASP	20	14.060	7.465	2.126	1.00	0.00	C
ATOM	314	HB2	ASP	20	13.845	8.117	1.280	1.00	0.00	H
ATOM	315	HB3	ASP	20	13.154	6.926	2.406	1.00	0.00	H
ATOM	316	CG	ASP	20	15.103	6.493	1.633	1.00	0.00	C
ATOM	317	OD1	ASP	20	15.516	5.553	2.377	1.00	0.00	O
ATOM	318	OD2	ASP	20	15.727	6.715	0.517	1.00	0.00	O
ATOM	319	C	ASP	20	15.644	9.068	2.844	1.00	0.00	C
ATOM	320	O	ASP	20	16.835	8.740	2.971	1.00	0.00	O
ATOM	321	N	TYR	21	15.299	10.230	2.294	1.00	0.00	N
ATOM	322	H	TYR	21	14.308	10.373	2.159	1.00	0.00	H
ATOM	323	CA	TYR	21	16.162	11.155	1.631	1.00	0.00	C
ATOM	324	HA	TYR	21	16.795	10.551	0.980	1.00	0.00	H
ATOM	325	CB	TYR	21	15.309	12.087	0.782	1.00	0.00	C
ATOM	326	HB2	TYR	21	15.911	12.819	0.244	1.00	0.00	H
ATOM	327	HB3	TYR	21	14.763	12.559	1.598	1.00	0.00	H
ATOM	328	CG	TYR	21	14.385	11.358	-0.197	1.00	0.00	C
ATOM	329	CD1	TYR	21	13.000	11.545	-0.092	1.00	0.00	C

ATOM	330	HD1	TYR	21	12.659	12.315	0.583	1.00	0.00	H
ATOM	331	CE1	TYR	21	12.091	10.799	-0.876	1.00	0.00	C
ATOM	332	HE1	TYR	21	11.021	10.919	-0.796	1.00	0.00	H
ATOM	333	CZ	TYR	21	12.541	9.733	-1.566	1.00	0.00	C
ATOM	334	OH	TYR	21	11.632	8.828	-1.967	1.00	0.00	O
ATOM	335	HH	TYR	21	10.734	8.956	-1.652	1.00	0.00	H
ATOM	336	CE2	TYR	21	13.901	9.556	-1.721	1.00	0.00	C
ATOM	337	HE2	TYR	21	14.171	8.816	-2.460	1.00	0.00	H
ATOM	338	CD2	TYR	21	14.827	10.338	-1.027	1.00	0.00	C
ATOM	339	HD2	TYR	21	15.883	10.215	-1.213	1.00	0.00	H
ATOM	340	C	TYR	21	17.088	11.903	2.570	1.00	0.00	C
ATOM	341	O	TYR	21	18.182	12.340	2.229	1.00	0.00	O
ATOM	342	N	GLU	22	16.690	12.064	3.822	1.00	0.00	N
ATOM	343	H	GLU	22	15.861	11.567	4.115	1.00	0.00	H
ATOM	344	CA	GLU	22	17.458	12.604	4.894	1.00	0.00	C
ATOM	345	HA	GLU	22	18.330	13.227	4.700	1.00	0.00	H
ATOM	346	CB	GLU	22	16.510	13.362	5.864	1.00	0.00	C
ATOM	347	HB2	GLU	22	16.901	13.792	6.786	1.00	0.00	H
ATOM	348	HB3	GLU	22	15.720	12.672	6.157	1.00	0.00	H
ATOM	349	CG	GLU	22	15.903	14.635	5.176	1.00	0.00	C
ATOM	350	HG2	GLU	22	15.695	14.669	4.106	1.00	0.00	H
ATOM	351	HG3	GLU	22	16.817	15.227	5.219	1.00	0.00	H
ATOM	352	CD	GLU	22	14.778	15.419	5.974	1.00	0.00	C
ATOM	353	OE1	GLU	22	13.632	15.543	5.462	1.00	0.00	O
ATOM	354	OE2	GLU	22	15.083	15.979	7.102	1.00	0.00	O
ATOM	355	C	GLU	22	18.236	11.487	5.646	1.00	0.00	C
ATOM	356	O	GLU	22	19.166	11.794	6.378	1.00	0.00	O
ATOM	357	N	ASP	23	17.745	10.269	5.585	1.00	0.00	N
ATOM	358	H	ASP	23	16.931	9.997	5.054	1.00	0.00	H
ATOM	359	CA	ASP	23	18.468	9.099	6.135	1.00	0.00	C
ATOM	360	HA	ASP	23	18.844	9.329	7.132	1.00	0.00	H
ATOM	361	CB	ASP	23	17.483	7.942	6.283	1.00	0.00	C
ATOM	362	HB2	ASP	23	17.034	7.700	5.320	1.00	0.00	H
ATOM	363	HB3	ASP	23	16.633	8.210	6.909	1.00	0.00	H
ATOM	364	CG	ASP	23	18.221	6.674	6.929	1.00	0.00	C
ATOM	365	OD1	ASP	23	18.687	5.694	6.273	1.00	0.00	O
ATOM	366	OD2	ASP	23	18.349	6.722	8.166	1.00	0.00	O
ATOM	367	C	ASP	23	19.686	8.683	5.294	1.00	0.00	C
ATOM	368	O	ASP	23	20.719	8.460	5.891	1.00	0.00	O
ATOM	369	N	ARG	24	19.635	8.956	3.954	1.00	0.00	N
ATOM	370	H	ARG	24	18.705	9.106	3.591	1.00	0.00	H
ATOM	371	CA	ARG	24	20.672	8.721	3.035	1.00	0.00	C
ATOM	372	HA	ARG	24	21.173	7.810	3.361	1.00	0.00	H
ATOM	373	CB	ARG	24	20.209	8.471	1.650	1.00	0.00	C
ATOM	374	HB2	ARG	24	19.554	7.602	1.580	1.00	0.00	H
ATOM	375	HB3	ARG	24	21.088	8.081	1.136	1.00	0.00	H
ATOM	376	CG	ARG	24	19.584	9.651	0.902	1.00	0.00	C
ATOM	377	HG2	ARG	24	20.108	10.605	0.965	1.00	0.00	H
ATOM	378	HG3	ARG	24	18.690	9.707	1.522	1.00	0.00	H
ATOM	379	CD	ARG	24	19.384	9.359	-0.581	1.00	0.00	C
ATOM	380	HD2	ARG	24	20.307	9.189	-1.135	1.00	0.00	H
ATOM	381	HD3	ARG	24	19.020	10.318	-0.948	1.00	0.00	H
ATOM	382	NE	ARG	24	18.337	8.366	-0.866	1.00	0.00	N
ATOM	383	HE	ARG	24	17.987	7.719	-0.174	1.00	0.00	H
ATOM	384	CZ	ARG	24	17.776	7.980	-2.063	1.00	0.00	C
ATOM	385	NH1	ARG	24	18.106	8.529	-3.260	1.00	0.00	N
ATOM	386	HH11	ARG	24	18.622	9.388	-3.388	1.00	0.00	H
ATOM	387	HH12	ARG	24	17.704	8.169	-4.114	1.00	0.00	H
ATOM	388	NH2	ARG	24	16.695	7.202	-2.039	1.00	0.00	N
ATOM	389	HH21	ARG	24	16.283	6.864	-1.181	1.00	0.00	H
ATOM	390	HH22	ARG	24	16.206	7.000	-2.900	1.00	0.00	H
ATOM	391	C	ARG	24	21.893	9.692	2.986	1.00	0.00	C
ATOM	392	O	ARG	24	22.822	9.538	2.159	1.00	0.00	O

ATOM	393	N	TYR	25	21.951	10.616	3.916	1.00	0.00	N
ATOM	394	H	TYR	25	21.288	10.489	4.667	1.00	0.00	H
ATOM	395	CA	TYR	25	23.041	11.626	3.967	1.00	0.00	C
ATOM	396	HA	TYR	25	23.883	11.483	3.290	1.00	0.00	H
ATOM	397	CB	TYR	25	22.532	13.098	3.616	1.00	0.00	C
ATOM	398	HB2	TYR	25	23.335	13.807	3.816	1.00	0.00	H
ATOM	399	HB3	TYR	25	21.736	13.258	4.343	1.00	0.00	H
ATOM	400	CG	TYR	25	22.103	13.393	2.198	1.00	0.00	C
ATOM	401	CD1	TYR	25	20.909	14.119	1.896	1.00	0.00	C
ATOM	402	HD1	TYR	25	20.268	14.453	2.699	1.00	0.00	H
ATOM	403	CE1	TYR	25	20.505	14.349	0.578	1.00	0.00	C
ATOM	404	HE1	TYR	25	19.580	14.861	0.356	1.00	0.00	H
ATOM	405	CZ	TYR	25	21.343	13.959	-0.490	1.00	0.00	C
ATOM	406	OH	TYR	25	20.860	14.063	-1.817	1.00	0.00	O
ATOM	407	HH	TYR	25	21.487	13.796	-2.494	1.00	0.00	H
ATOM	408	CE2	TYR	25	22.530	13.263	-0.235	1.00	0.00	C
ATOM	409	HE2	TYR	25	23.187	13.003	-1.052	1.00	0.00	H
ATOM	410	CD2	TYR	25	22.953	12.986	1.125	1.00	0.00	C
ATOM	411	HD2	TYR	25	23.854	12.424	1.325	1.00	0.00	H
ATOM	412	C	TYR	25	23.774	11.644	5.342	1.00	0.00	C
ATOM	413	O	TYR	25	24.937	11.133	5.392	1.00	0.00	O
ATOM	414	N	TYR	26	23.197	12.218	6.405	1.00	0.00	N
ATOM	415	H	TYR	26	22.306	12.681	6.296	1.00	0.00	H
ATOM	416	CA	TYR	26	23.755	12.492	7.726	1.00	0.00	C
ATOM	417	HA	TYR	26	24.621	13.149	7.647	1.00	0.00	H
ATOM	418	CB	TYR	26	22.875	13.351	8.682	1.00	0.00	C
ATOM	419	HB2	TYR	26	23.381	13.651	9.599	1.00	0.00	H
ATOM	420	HB3	TYR	26	22.200	12.584	9.063	1.00	0.00	H
ATOM	421	CG	TYR	26	22.042	14.540	8.162	1.00	0.00	C
ATOM	422	CD1	TYR	26	22.526	15.845	8.098	1.00	0.00	C
ATOM	423	HD1	TYR	26	23.564	15.926	8.386	1.00	0.00	H
ATOM	424	CE1	TYR	26	21.617	16.924	7.710	1.00	0.00	C
ATOM	425	HE1	TYR	26	22.000	17.933	7.687	1.00	0.00	H
ATOM	426	CZ	TYR	26	20.305	16.634	7.292	1.00	0.00	C
ATOM	427	OH	TYR	26	19.509	17.623	6.863	1.00	0.00	O
ATOM	428	HH	TYR	26	18.758	17.242	6.402	1.00	0.00	H
ATOM	429	CE2	TYR	26	19.805	15.371	7.435	1.00	0.00	C
ATOM	430	HE2	TYR	26	18.798	15.107	7.149	1.00	0.00	H
ATOM	431	CD2	TYR	26	20.671	14.343	7.954	1.00	0.00	C
ATOM	432	HD2	TYR	26	20.228	13.373	8.121	1.00	0.00	H
ATOM	433	C	TYR	26	24.296	11.227	8.506	1.00	0.00	C
ATOM	434	O	TYR	26	25.306	11.267	9.122	1.00	0.00	O
ATOM	435	N	ARG	27	23.596	10.080	8.296	1.00	0.00	N
ATOM	436	H	ARG	27	22.678	10.296	7.932	1.00	0.00	H
ATOM	437	CA	ARG	27	23.888	8.785	9.014	1.00	0.00	C
ATOM	438	HA	ARG	27	24.337	9.040	9.974	1.00	0.00	H
ATOM	439	CB	ARG	27	22.594	8.006	9.339	1.00	0.00	C
ATOM	440	HB2	ARG	27	22.346	7.382	8.481	1.00	0.00	H
ATOM	441	HB3	ARG	27	21.760	8.706	9.398	1.00	0.00	H
ATOM	442	CG	ARG	27	22.605	7.094	10.584	1.00	0.00	C
ATOM	443	HG2	ARG	27	22.930	7.660	11.457	1.00	0.00	H
ATOM	444	HG3	ARG	27	23.346	6.338	10.325	1.00	0.00	H
ATOM	445	CD	ARG	27	21.184	6.480	10.834	1.00	0.00	C
ATOM	446	HD2	ARG	27	20.506	7.287	11.111	1.00	0.00	H
ATOM	447	HD3	ARG	27	21.300	5.785	11.666	1.00	0.00	H
ATOM	448	NE	ARG	27	20.587	5.825	9.591	1.00	0.00	N
ATOM	449	HE	ARG	27	19.891	6.314	9.046	1.00	0.00	H
ATOM	450	CZ	ARG	27	20.734	4.573	9.200	1.00	0.00	C
ATOM	451	NH1	ARG	27	21.494	3.770	9.860	1.00	0.00	N
ATOM	452	HH11	ARG	27	22.078	4.064	10.629	1.00	0.00	H
ATOM	453	HH12	ARG	27	21.611	2.832	9.503	1.00	0.00	H
ATOM	454	NH2	ARG	27	20.031	4.166	8.202	1.00	0.00	N
ATOM	455	HH21	ARG	27	19.542	4.878	7.679	1.00	0.00	H

ATOM	456	HH22	ARG	27	19.908	3.213	7.890	1.00	0.00	H
ATOM	457	C	ARG	27	24.926	7.931	8.247	1.00	0.00	C
ATOM	458	O	ARG	27	25.209	6.735	8.557	1.00	0.00	O
ATOM	459	N	GLU	28	25.544	8.471	7.291	1.00	0.00	N
ATOM	460	H	GLU	28	25.107	9.331	6.991	1.00	0.00	H
ATOM	461	CA	GLU	28	26.581	7.915	6.440	1.00	0.00	C
ATOM	462	HA	GLU	28	27.208	7.166	6.923	1.00	0.00	H
ATOM	463	CB	GLU	28	26.010	7.157	5.221	1.00	0.00	C
ATOM	464	HB2	GLU	28	25.417	6.339	5.630	1.00	0.00	H
ATOM	465	HB3	GLU	28	26.894	6.868	4.653	1.00	0.00	H
ATOM	466	CG	GLU	28	25.028	7.896	4.280	1.00	0.00	C
ATOM	467	HG2	GLU	28	25.312	8.918	4.029	1.00	0.00	H
ATOM	468	HG3	GLU	28	24.113	8.033	4.856	1.00	0.00	H
ATOM	469	CD	GLU	28	24.811	7.095	2.924	1.00	0.00	C
ATOM	470	OE1	GLU	28	23.872	6.349	2.729	1.00	0.00	O
ATOM	471	OE2	GLU	28	25.778	7.217	2.104	1.00	0.00	O
ATOM	472	C	GLU	28	27.533	9.014	5.911	1.00	0.00	C
ATOM	473	O	GLU	28	28.076	9.051	4.815	1.00	0.00	O
ATOM	474	N	ASN	29	27.950	9.880	6.873	1.00	0.00	N
ATOM	475	H	ASN	29	27.589	9.648	7.787	1.00	0.00	H
ATOM	476	CA	ASN	29	28.717	11.122	6.755	1.00	0.00	C
ATOM	477	HA	ASN	29	28.239	11.737	5.993	1.00	0.00	H
ATOM	478	CB	ASN	29	28.714	11.809	8.124	1.00	0.00	C
ATOM	479	HB2	ASN	29	29.633	11.592	8.669	1.00	0.00	H
ATOM	480	HB3	ASN	29	27.790	11.577	8.654	1.00	0.00	H
ATOM	481	CG	ASN	29	28.611	13.384	7.956	1.00	0.00	C
ATOM	482	OD1	ASN	29	27.601	14.035	7.713	1.00	0.00	O
ATOM	483	ND2	ASN	29	29.726	14.042	8.080	1.00	0.00	N
ATOM	484	HD21	ASN	29	29.712	15.052	8.057	1.00	0.00	H
ATOM	485	HD22	ASN	29	30.554	13.495	8.262	1.00	0.00	H
ATOM	486	C	ASN	29	30.138	10.978	6.201	1.00	0.00	C
ATOM	487	O	ASN	29	30.855	11.983	5.912	1.00	0.00	O
ATOM	488	N	MET	30	30.587	9.728	6.060	1.00	0.00	N
ATOM	489	H	MET	30	30.050	9.018	6.537	1.00	0.00	H
ATOM	490	CA	MET	30	31.841	9.407	5.413	1.00	0.00	C
ATOM	491	HA	MET	30	32.497	10.267	5.541	1.00	0.00	H
ATOM	492	CB	MET	30	32.578	8.341	6.234	1.00	0.00	C
ATOM	493	HB2	MET	30	32.814	8.762	7.211	1.00	0.00	H
ATOM	494	HB3	MET	30	33.446	8.296	5.575	1.00	0.00	H
ATOM	495	CG	MET	30	31.861	6.977	6.321	1.00	0.00	C
ATOM	496	HG2	MET	30	32.509	6.166	6.653	1.00	0.00	H
ATOM	497	HG3	MET	30	31.676	6.668	5.292	1.00	0.00	H
ATOM	498	SD	MET	30	30.337	6.769	7.340	1.00	0.00	S
ATOM	499	CE	MET	30	30.931	7.076	9.047	1.00	0.00	C
ATOM	500	HE1	MET	30	31.951	7.459	9.008	1.00	0.00	H
ATOM	501	HE2	MET	30	30.366	7.811	9.620	1.00	0.00	H
ATOM	502	HE3	MET	30	30.908	6.145	9.614	1.00	0.00	H
ATOM	503	C	MET	30	31.743	9.101	3.917	1.00	0.00	C
ATOM	504	O	MET	30	32.633	9.288	3.164	1.00	0.00	O
ATOM	505	N	TYR	31	30.547	8.663	3.488	1.00	0.00	N
ATOM	506	H	TYR	31	29.827	8.559	4.189	1.00	0.00	H
ATOM	507	CA	TYR	31	30.193	8.570	2.061	1.00	0.00	C
ATOM	508	HA	TYR	31	31.122	8.440	1.506	1.00	0.00	H
ATOM	509	CB	TYR	31	29.463	7.288	1.804	1.00	0.00	C
ATOM	510	HB2	TYR	31	29.307	7.190	0.729	1.00	0.00	H
ATOM	511	HB3	TYR	31	28.525	7.561	2.287	1.00	0.00	H
ATOM	512	CG	TYR	31	30.011	5.964	2.259	1.00	0.00	C
ATOM	513	CD1	TYR	31	29.801	5.536	3.613	1.00	0.00	C
ATOM	514	HD1	TYR	31	29.337	6.219	4.309	1.00	0.00	H
ATOM	515	CE1	TYR	31	30.410	4.354	4.065	1.00	0.00	C
ATOM	516	HE1	TYR	31	30.177	4.014	5.064	1.00	0.00	H
ATOM	517	CZ	TYR	31	31.136	3.507	3.196	1.00	0.00	C
ATOM	518	OH	TYR	31	31.660	2.383	3.773	1.00	0.00	O

```
ATOM    519  HH  TYR    31      31.612   2.294   4.727  1.00  0.00           H
ATOM    520  CE2 TYR    31      31.238   3.890   1.782  1.00  0.00           C
ATOM    521  HE2 TYR    31      31.769   3.198   1.146  1.00  0.00           H
ATOM    522  CD2 TYR    31      30.689   5.091   1.363  1.00  0.00           C
ATOM    523  HD2 TYR    31      30.803   5.402   0.335  1.00  0.00           H
ATOM    524  C   TYR    31      29.606   9.908   1.555  1.00  0.00           C
ATOM    525  O   TYR    31      30.136  10.397   0.560  1.00  0.00           O
ATOM    526  N   ARG    32      28.594  10.536   2.138  1.00  0.00           N
ATOM    527  H   ARG    32      28.229  10.005   2.916  1.00  0.00           H
ATOM    528  CA  ARG    32      27.749  11.665   1.767  1.00  0.00           C
ATOM    529  HA  ARG    32      28.239  12.293   1.023  1.00  0.00           H
ATOM    530  CB  ARG    32      26.434  11.125   1.068  1.00  0.00           C
ATOM    531  HB2 ARG    32      25.924  11.898   0.493  1.00  0.00           H
ATOM    532  HB3 ARG    32      25.771  10.695   1.819  1.00  0.00           H
ATOM    533  CG  ARG    32      26.700  10.159  -0.139  1.00  0.00           C
ATOM    534  HG2 ARG    32      27.324   9.299   0.106  1.00  0.00           H
ATOM    535  HG3 ARG    32      27.309  10.824  -0.752  1.00  0.00           H
ATOM    536  CD  ARG    32      25.542   9.657  -0.902  1.00  0.00           C
ATOM    537  HD2 ARG    32      25.925   9.260  -1.842  1.00  0.00           H
ATOM    538  HD3 ARG    32      24.898  10.521  -1.064  1.00  0.00           H
ATOM    539  NE  ARG    32      24.863   8.559  -0.246  1.00  0.00           N
ATOM    540  HE  ARG    32      25.227   8.212   0.630  1.00  0.00           H
ATOM    541  CZ  ARG    32      23.902   7.832  -0.803  1.00  0.00           C
ATOM    542  NH1 ARG    32      23.403   8.065  -2.053  1.00  0.00           N
ATOM    543 HH11 ARG    32      23.664   8.835  -2.652  1.00  0.00           H
ATOM    544 HH12 ARG    32      22.701   7.404  -2.356  1.00  0.00           H
ATOM    545  NH2 ARG    32      23.427   6.834  -0.087  1.00  0.00           N
ATOM    546 HH21 ARG    32      23.500   6.780   0.919  1.00  0.00           H
ATOM    547 HH22 ARG    32      22.803   6.283  -0.659  1.00  0.00           H
ATOM    548  C   ARG    32      27.467  12.700   2.910  1.00  0.00           C
ATOM    549  O   ARG    32      26.307  13.004   3.254  1.00  0.00           O
ATOM    550  N   TYR    33      28.507  13.197   3.522  1.00  0.00           N
ATOM    551  H   TYR    33      29.383  12.706   3.418  1.00  0.00           H
ATOM    552  CA  TYR    33      28.486  14.408   4.337  1.00  0.00           C
ATOM    553  HA  TYR    33      28.176  14.074   5.327  1.00  0.00           H
ATOM    554  CB  TYR    33      29.889  14.913   4.398  1.00  0.00           C
ATOM    555  HB2 TYR    33      30.564  14.059   4.460  1.00  0.00           H
ATOM    556  HB3 TYR    33      29.865  15.516   5.306  1.00  0.00           H
ATOM    557  CG  TYR    33      30.364  15.792   3.255  1.00  0.00           C
ATOM    558  CD1 TYR    33      30.324  17.125   3.595  1.00  0.00           C
ATOM    559  HD1 TYR    33      29.932  17.507   4.527  1.00  0.00           H
ATOM    560  CE1 TYR    33      30.597  18.102   2.651  1.00  0.00           C
ATOM    561  HE1 TYR    33      30.508  19.150   2.894  1.00  0.00           H
ATOM    562  CZ  TYR    33      31.138  17.716   1.451  1.00  0.00           C
ATOM    563  OH  TYR    33      31.548  18.658   0.534  1.00  0.00           O
ATOM    564  HH  TYR    33      32.084  18.330  -0.192  1.00  0.00           H
ATOM    565  CE2 TYR    33      31.234  16.344   1.120  1.00  0.00           C
ATOM    566  HE2 TYR    33      31.601  16.161   0.121  1.00  0.00           H
ATOM    567  CD2 TYR    33      30.788  15.390   2.019  1.00  0.00           C
ATOM    568  HD2 TYR    33      30.716  14.359   1.705  1.00  0.00           H
ATOM    569  C   TYR    33      27.574  15.622   3.940  1.00  0.00           C
ATOM    570  O   TYR    33      26.887  16.173   4.840  1.00  0.00           O
ATOM    571  N   NME    34      27.486  16.012   2.649  1.00  0.00           N
ATOM    572  H   NME    34      28.177  15.661   2.002  1.00  0.00           H
ATOM    573  CH3 NME    34      26.380  16.904   2.206  1.00  0.00           C
ATOM    574 HH31 NME    34      26.514  17.825   2.773  1.00  0.00           H
ATOM    575 HH32 NME    34      26.374  17.148   1.143  1.00  0.00           H
ATOM    576 HH33 NME    34      25.458  16.375   2.444  1.00  0.00           H
TER     577      NME    34
END
```

Cluster 4:

```
ATOM      1 HH31 ACE     1       9.361  -0.136  -6.411  1.00  0.00           H
ATOM      2  CH3 ACE     1       9.890  -0.896  -6.987  1.00  0.00           C
ATOM      3 HH32 ACE     1       9.358  -0.886  -7.938  1.00  0.00           H
ATOM      4 HH33 ACE     1       9.961  -1.891  -6.548  1.00  0.00           H
ATOM      5  C   ACE     1      11.278  -0.389  -7.336  1.00  0.00           C
ATOM      6  O   ACE     1      12.311  -1.079  -7.244  1.00  0.00           O
ATOM      7  N   ASN     2      11.412   0.881  -7.630  1.00  0.00           N
ATOM      8  H   ASN     2      10.535   1.380  -7.689  1.00  0.00           H
ATOM      9  CA  ASN     2      12.650   1.598  -8.012  1.00  0.00           C
ATOM     10  HA  ASN     2      13.273   0.865  -8.522  1.00  0.00           H
ATOM     11  CB  ASN     2      12.259   2.657  -9.044  1.00  0.00           C
ATOM     12  HB2 ASN     2      11.727   3.487  -8.581  1.00  0.00           H
ATOM     13  HB3 ASN     2      11.599   2.188  -9.774  1.00  0.00           H
ATOM     14  CG  ASN     2      13.508   3.238  -9.799  1.00  0.00           C
ATOM     15  OD1 ASN     2      14.237   2.570 -10.469  1.00  0.00           O
ATOM     16  ND2 ASN     2      13.700   4.476  -9.589  1.00  0.00           N
ATOM     17 HD21 ASN     2      14.330   4.961 -10.211  1.00  0.00           H
ATOM     18 HD22 ASN     2      13.167   5.028  -8.931  1.00  0.00           H
ATOM     19  C   ASN     2      13.545   2.184  -6.859  1.00  0.00           C
ATOM     20  O   ASN     2      14.766   2.121  -6.910  1.00  0.00           O
ATOM     21  N   ASP     3      12.855   2.729  -5.851  1.00  0.00           N
ATOM     22  H   ASP     3      11.848   2.772  -5.924  1.00  0.00           H
ATOM     23  CA  ASP     3      13.445   3.497  -4.802  1.00  0.00           C
ATOM     24  HA  ASP     3      14.151   4.207  -5.232  1.00  0.00           H
ATOM     25  CB  ASP     3      12.380   4.543  -4.393  1.00  0.00           C
ATOM     26  HB2 ASP     3      11.460   4.041  -4.095  1.00  0.00           H
ATOM     27  HB3 ASP     3      12.291   4.962  -5.395  1.00  0.00           H
ATOM     28  CG  ASP     3      12.819   5.518  -3.235  1.00  0.00           C
ATOM     29  OD1 ASP     3      13.307   6.621  -3.617  1.00  0.00           O
ATOM     30  OD2 ASP     3      12.505   5.231  -2.047  1.00  0.00           O
ATOM     31  C   ASP     3      14.044   2.747  -3.596  1.00  0.00           C
ATOM     32  O   ASP     3      14.750   3.291  -2.808  1.00  0.00           O
ATOM     33  N   TYR     4      13.677   1.498  -3.494  1.00  0.00           N
ATOM     34  H   TYR     4      13.007   1.267  -4.215  1.00  0.00           H
ATOM     35  CA  TYR     4      14.051   0.511  -2.437  1.00  0.00           C
ATOM     36  HA  TYR     4      13.556   0.733  -1.492  1.00  0.00           H
ATOM     37  CB  TYR     4      13.370  -0.802  -2.828  1.00  0.00           C
ATOM     38  HB2 TYR     4      13.805  -1.139  -3.768  1.00  0.00           H
ATOM     39  HB3 TYR     4      12.320  -0.548  -2.979  1.00  0.00           H
ATOM     40  CG  TYR     4      13.389  -1.984  -1.910  1.00  0.00           C
ATOM     41  CD1 TYR     4      12.532  -1.957  -0.815  1.00  0.00           C
ATOM     42  HD1 TYR     4      11.909  -1.091  -0.646  1.00  0.00           H
ATOM     43  CE1 TYR     4      12.423  -3.039   0.069  1.00  0.00           C
ATOM     44  HE1 TYR     4      11.790  -2.898   0.933  1.00  0.00           H
ATOM     45  CZ  TYR     4      13.257  -4.138  -0.221  1.00  0.00           C
ATOM     46  OH  TYR     4      13.341  -5.178   0.653  1.00  0.00           O
ATOM     47  HH  TYR     4      12.829  -5.028   1.451  1.00  0.00           H
ATOM     48  CE2 TYR     4      14.075  -4.276  -1.368  1.00  0.00           C
ATOM     49  HE2 TYR     4      14.664  -5.170  -1.516  1.00  0.00           H
ATOM     50  CD2 TYR     4      14.145  -3.172  -2.232  1.00  0.00           C
ATOM     51  HD2 TYR     4      14.821  -3.286  -3.067  1.00  0.00           H
ATOM     52  C   TYR     4      15.524   0.297  -2.190  1.00  0.00           C
ATOM     53  O   TYR     4      16.022  -0.041  -1.107  1.00  0.00           O
ATOM     54  N   GLU     5      16.318   0.311  -3.294  1.00  0.00           N
ATOM     55  H   GLU     5      15.884   0.438  -4.197  1.00  0.00           H
ATOM     56  CA  GLU     5      17.761   0.055  -3.349  1.00  0.00           C
ATOM     57  HA  GLU     5      17.948  -0.840  -2.756  1.00  0.00           H
ATOM     58  CB  GLU     5      18.119  -0.258  -4.818  1.00  0.00           C
ATOM     59  HB2 GLU     5      18.027   0.640  -5.430  1.00  0.00           H
ATOM     60  HB3 GLU     5      17.427  -0.984  -5.245  1.00  0.00           H
ATOM     61  CG  GLU     5      19.561  -0.721  -5.092  1.00  0.00           C
```

ATOM	62	HG2	GLU	5	20.272	-0.023	-4.652	1.00	0.00	H
ATOM	63	HG3	GLU	5	19.551	-0.753	-6.182	1.00	0.00	H
ATOM	64	CD	GLU	5	19.959	-2.119	-4.513	1.00	0.00	C
ATOM	65	OE1	GLU	5	20.023	-2.276	-3.278	1.00	0.00	O
ATOM	66	OE2	GLU	5	20.158	-3.090	-5.262	1.00	0.00	O
ATOM	67	C	GLU	5	18.422	1.343	-2.888	1.00	0.00	C
ATOM	68	O	GLU	5	19.460	1.324	-2.252	1.00	0.00	O
ATOM	69	N	ASP	6	17.895	2.555	-3.185	1.00	0.00	N
ATOM	70	H	ASP	6	16.927	2.598	-3.470	1.00	0.00	H
ATOM	71	CA	ASP	6	18.426	3.889	-2.858	1.00	0.00	C
ATOM	72	HA	ASP	6	19.509	3.792	-2.795	1.00	0.00	H
ATOM	73	CB	ASP	6	18.052	4.852	-3.979	1.00	0.00	C
ATOM	74	HB2	ASP	6	17.012	5.168	-3.888	1.00	0.00	H
ATOM	75	HB3	ASP	6	18.354	4.374	-4.910	1.00	0.00	H
ATOM	76	CG	ASP	6	18.847	6.142	-3.749	1.00	0.00	C
ATOM	77	OD1	ASP	6	18.184	7.104	-3.361	1.00	0.00	O
ATOM	78	OD2	ASP	6	20.095	6.197	-3.915	1.00	0.00	O
ATOM	79	C	ASP	6	18.000	4.384	-1.468	1.00	0.00	C
ATOM	80	O	ASP	6	18.677	5.253	-0.898	1.00	0.00	O
ATOM	81	N	ARG	7	16.844	3.893	-0.964	1.00	0.00	N
ATOM	82	H	ARG	7	16.311	3.254	-1.538	1.00	0.00	H
ATOM	83	CA	ARG	7	16.459	4.010	0.488	1.00	0.00	C
ATOM	84	HA	ARG	7	16.678	5.012	0.855	1.00	0.00	H
ATOM	85	CB	ARG	7	14.939	3.902	0.634	1.00	0.00	C
ATOM	86	HB2	ARG	7	14.369	4.563	-0.020	1.00	0.00	H
ATOM	87	HB3	ARG	7	14.772	4.189	1.672	1.00	0.00	H
ATOM	88	CG	ARG	7	14.341	2.564	0.462	1.00	0.00	C
ATOM	89	HG2	ARG	7	14.862	1.811	1.051	1.00	0.00	H
ATOM	90	HG3	ARG	7	14.464	2.265	-0.578	1.00	0.00	H
ATOM	91	CD	ARG	7	12.881	2.445	0.794	1.00	0.00	C
ATOM	92	HD2	ARG	7	12.669	2.785	1.808	1.00	0.00	H
ATOM	93	HD3	ARG	7	12.638	1.391	0.667	1.00	0.00	H
ATOM	94	NE	ARG	7	12.024	3.251	-0.102	1.00	0.00	N
ATOM	95	HE	ARG	7	12.440	3.847	-0.804	1.00	0.00	H
ATOM	96	CZ	ARG	7	10.705	3.177	-0.201	1.00	0.00	C
ATOM	97	NH1	ARG	7	9.848	2.371	0.440	1.00	0.00	N
ATOM	98	HH11	ARG	7	10.187	1.842	1.231	1.00	0.00	H
ATOM	99	HH12	ARG	7	8.866	2.609	0.443	1.00	0.00	H
ATOM	100	NH2	ARG	7	10.097	4.135	-0.957	1.00	0.00	N
ATOM	101	HH21	ARG	7	10.733	4.796	-1.380	1.00	0.00	H
ATOM	102	HH22	ARG	7	9.097	4.273	-0.980	1.00	0.00	H
ATOM	103	C	ARG	7	17.239	3.139	1.420	1.00	0.00	C
ATOM	104	O	ARG	7	17.413	3.580	2.563	1.00	0.00	O
ATOM	105	N	TYR	8	17.789	2.005	0.994	1.00	0.00	N
ATOM	106	H	TYR	8	17.504	1.673	0.084	1.00	0.00	H
ATOM	107	CA	TYR	8	18.533	1.115	1.764	1.00	0.00	C
ATOM	108	HA	TYR	8	18.097	0.969	2.752	1.00	0.00	H
ATOM	109	CB	TYR	8	18.540	-0.263	1.126	1.00	0.00	C
ATOM	110	HB2	TYR	8	19.112	-0.274	0.197	1.00	0.00	H
ATOM	111	HB3	TYR	8	17.530	-0.499	0.790	1.00	0.00	H
ATOM	112	CG	TYR	8	18.948	-1.403	2.022	1.00	0.00	C
ATOM	113	CD1	TYR	8	18.229	-1.796	3.133	1.00	0.00	C
ATOM	114	HD1	TYR	8	17.356	-1.220	3.403	1.00	0.00	H
ATOM	115	CE1	TYR	8	18.532	-3.022	3.847	1.00	0.00	C
ATOM	116	HE1	TYR	8	18.032	-3.266	4.772	1.00	0.00	H
ATOM	117	CZ	TYR	8	19.725	-3.761	3.515	1.00	0.00	C
ATOM	118	OH	TYR	8	20.213	-4.879	4.186	1.00	0.00	O
ATOM	119	HH	TYR	8	21.062	-5.241	3.922	1.00	0.00	H
ATOM	120	CE2	TYR	8	20.528	-3.291	2.391	1.00	0.00	C
ATOM	121	HE2	TYR	8	21.410	-3.797	2.029	1.00	0.00	H
ATOM	122	CD2	TYR	8	20.107	-2.139	1.706	1.00	0.00	C
ATOM	123	HD2	TYR	8	20.635	-1.922	0.789	1.00	0.00	H
ATOM	124	C	TYR	8	19.916	1.636	2.034	1.00	0.00	C

ATOM	125	O	TYR	8	20.611	2.324	1.253	1.00	0.00	O
ATOM	126	N	TYR	9	20.360	1.330	3.287	1.00	0.00	N
ATOM	127	H	TYR	9	19.838	0.671	3.846	1.00	0.00	H
ATOM	128	CA	TYR	9	21.786	1.483	3.800	1.00	0.00	C
ATOM	129	HA	TYR	9	22.008	2.548	3.737	1.00	0.00	H
ATOM	130	CB	TYR	9	21.809	0.964	5.263	1.00	0.00	C
ATOM	131	HB2	TYR	9	22.813	0.972	5.690	1.00	0.00	H
ATOM	132	HB3	TYR	9	21.398	-0.043	5.190	1.00	0.00	H
ATOM	133	CG	TYR	9	20.920	1.711	6.232	1.00	0.00	C
ATOM	134	CD1	TYR	9	19.742	1.143	6.754	1.00	0.00	C
ATOM	135	HD1	TYR	9	19.464	0.166	6.386	1.00	0.00	H
ATOM	136	CE1	TYR	9	19.026	1.714	7.795	1.00	0.00	C
ATOM	137	HE1	TYR	9	18.240	1.193	8.322	1.00	0.00	H
ATOM	138	CZ	TYR	9	19.498	2.915	8.343	1.00	0.00	C
ATOM	139	OH	TYR	9	18.902	3.386	9.476	1.00	0.00	O
ATOM	140	HH	TYR	9	19.443	4.112	9.794	1.00	0.00	H
ATOM	141	CE2	TYR	9	20.585	3.593	7.697	1.00	0.00	C
ATOM	142	HE2	TYR	9	20.969	4.511	8.117	1.00	0.00	H
ATOM	143	CD2	TYR	9	21.319	2.925	6.757	1.00	0.00	C
ATOM	144	HD2	TYR	9	22.223	3.351	6.345	1.00	0.00	H
ATOM	145	C	TYR	9	22.911	0.777	2.985	1.00	0.00	C
ATOM	146	O	TYR	9	22.704	-0.311	2.484	1.00	0.00	O
ATOM	147	N	ARG	10	23.997	1.471	2.908	1.00	0.00	N
ATOM	148	H	ARG	10	24.044	2.330	3.438	1.00	0.00	H
ATOM	149	CA	ARG	10	25.295	1.120	2.331	1.00	0.00	C
ATOM	150	HA	ARG	10	25.241	0.313	1.601	1.00	0.00	H
ATOM	151	CB	ARG	10	25.887	2.305	1.611	1.00	0.00	C
ATOM	152	HB2	ARG	10	26.902	2.048	1.308	1.00	0.00	H
ATOM	153	HB3	ARG	10	25.934	3.160	2.286	1.00	0.00	H
ATOM	154	CG	ARG	10	25.254	2.707	0.272	1.00	0.00	C
ATOM	155	HG2	ARG	10	25.325	1.931	-0.490	1.00	0.00	H
ATOM	156	HG3	ARG	10	25.986	3.399	-0.145	1.00	0.00	H
ATOM	157	CD	ARG	10	23.908	3.474	0.338	1.00	0.00	C
ATOM	158	HD2	ARG	10	24.023	4.234	1.112	1.00	0.00	H
ATOM	159	HD3	ARG	10	23.236	2.666	0.627	1.00	0.00	H
ATOM	160	NE	ARG	10	23.616	4.157	-0.996	1.00	0.00	N
ATOM	161	HE	ARG	10	24.389	4.169	-1.647	1.00	0.00	H
ATOM	162	CZ	ARG	10	22.469	4.688	-1.287	1.00	0.00	C
ATOM	163	NH1	ARG	10	21.420	4.671	-0.505	1.00	0.00	N
ATOM	164	HH11	ARG	10	21.445	4.406	0.469	1.00	0.00	H
ATOM	165	HH12	ARG	10	20.542	5.066	-0.809	1.00	0.00	H
ATOM	166	NH2	ARG	10	22.173	5.166	-2.448	1.00	0.00	N
ATOM	167	HH21	ARG	10	22.900	5.167	-3.150	1.00	0.00	H
ATOM	168	HH22	ARG	10	21.259	5.432	-2.780	1.00	0.00	H
ATOM	169	C	ARG	10	26.394	0.573	3.331	1.00	0.00	C
ATOM	170	O	ARG	10	27.385	0.027	2.885	1.00	0.00	O
ATOM	171	N	GLU	11	26.018	0.331	4.574	1.00	0.00	N
ATOM	172	H	GLU	11	25.203	0.831	4.904	1.00	0.00	H
ATOM	173	CA	GLU	11	26.733	-0.391	5.692	1.00	0.00	C
ATOM	174	HA	GLU	11	27.778	-0.106	5.578	1.00	0.00	H
ATOM	175	CB	GLU	11	26.308	0.192	7.031	1.00	0.00	C
ATOM	176	HB2	GLU	11	26.680	-0.425	7.848	1.00	0.00	H
ATOM	177	HB3	GLU	11	25.223	0.271	6.964	1.00	0.00	H
ATOM	178	CG	GLU	11	26.979	1.574	7.259	1.00	0.00	C
ATOM	179	HG2	GLU	11	26.705	2.329	6.521	1.00	0.00	H
ATOM	180	HG3	GLU	11	28.049	1.410	7.131	1.00	0.00	H
ATOM	181	CD	GLU	11	26.636	2.254	8.573	1.00	0.00	C
ATOM	182	OE1	GLU	11	27.565	2.513	9.382	1.00	0.00	O
ATOM	183	OE2	GLU	11	25.414	2.543	8.725	1.00	0.00	O
ATOM	184	C	GLU	11	26.767	-1.902	5.557	1.00	0.00	C
ATOM	185	O	GLU	11	27.376	-2.591	6.392	1.00	0.00	O
ATOM	186	N	ASN	12	26.049	-2.402	4.552	1.00	0.00	N
ATOM	187	H	ASN	12	25.509	-1.711	4.052	1.00	0.00	H

ATOM	188	CA	ASN	12	25.858	-3.833	4.250	1.00	0.00	C
ATOM	189	HA	ASN	12	25.801	-4.291	5.237	1.00	0.00	H
ATOM	190	CB	ASN	12	24.448	-3.970	3.603	1.00	0.00	C
ATOM	191	HB2	ASN	12	24.384	-3.353	2.706	1.00	0.00	H
ATOM	192	HB3	ASN	12	23.763	-3.605	4.367	1.00	0.00	H
ATOM	193	CG	ASN	12	24.161	-5.472	3.190	1.00	0.00	C
ATOM	194	OD1	ASN	12	23.341	-6.144	3.784	1.00	0.00	O
ATOM	195	ND2	ASN	12	24.633	-5.940	2.038	1.00	0.00	N
ATOM	196	HD21	ASN	12	24.183	-6.749	1.635	1.00	0.00	H
ATOM	197	HD22	ASN	12	25.416	-5.481	1.594	1.00	0.00	H
ATOM	198	C	ASN	12	27.088	-4.490	3.557	1.00	0.00	C
ATOM	199	O	ASN	12	27.237	-5.736	3.627	1.00	0.00	O
ATOM	200	N	MET	13	28.050	-3.619	3.179	1.00	0.00	N
ATOM	201	H	MET	13	27.809	-2.639	3.154	1.00	0.00	H
ATOM	202	CA	MET	13	29.314	-3.922	2.563	1.00	0.00	C
ATOM	203	HA	MET	13	29.359	-5.004	2.442	1.00	0.00	H
ATOM	204	CB	MET	13	29.371	-3.236	1.185	1.00	0.00	C
ATOM	205	HB2	MET	13	30.168	-3.667	0.578	1.00	0.00	H
ATOM	206	HB3	MET	13	29.543	-2.177	1.377	1.00	0.00	H
ATOM	207	CG	MET	13	28.152	-3.503	0.304	1.00	0.00	C
ATOM	208	HG2	MET	13	27.614	-4.438	0.463	1.00	0.00	H
ATOM	209	HG3	MET	13	28.658	-3.564	-0.660	1.00	0.00	H
ATOM	210	SD	MET	13	26.986	-2.146	0.158	1.00	0.00	S
ATOM	211	CE	MET	13	27.800	-0.891	-0.877	1.00	0.00	C
ATOM	212	HE1	MET	13	27.304	0.065	-1.049	1.00	0.00	H
ATOM	213	HE2	MET	13	28.765	-0.768	-0.385	1.00	0.00	H
ATOM	214	HE3	MET	13	28.091	-1.350	-1.821	1.00	0.00	H
ATOM	215	C	MET	13	30.559	-3.568	3.452	1.00	0.00	C
ATOM	216	O	MET	13	30.684	-2.445	3.942	1.00	0.00	O
ATOM	217	N	TYR	14	31.427	-4.544	3.711	1.00	0.00	N
ATOM	218	H	TYR	14	31.307	-5.372	3.146	1.00	0.00	H
ATOM	219	CA	TYR	14	32.499	-4.459	4.718	1.00	0.00	C
ATOM	220	HA	TYR	14	32.147	-3.829	5.535	1.00	0.00	H
ATOM	221	CB	TYR	14	32.747	-5.797	5.366	1.00	0.00	C
ATOM	222	HB2	TYR	14	33.553	-5.833	6.100	1.00	0.00	H
ATOM	223	HB3	TYR	14	33.005	-6.409	4.500	1.00	0.00	H
ATOM	224	CG	TYR	14	31.602	-6.503	5.978	1.00	0.00	C
ATOM	225	CD1	TYR	14	31.412	-6.513	7.368	1.00	0.00	C
ATOM	226	HD1	TYR	14	32.184	-6.054	7.968	1.00	0.00	H
ATOM	227	CE1	TYR	14	30.297	-7.157	7.990	1.00	0.00	C
ATOM	228	HE1	TYR	14	30.090	-7.040	9.044	1.00	0.00	H
ATOM	229	CZ	TYR	14	29.275	-7.891	7.271	1.00	0.00	C
ATOM	230	OH	TYR	14	28.108	-8.332	7.745	1.00	0.00	O
ATOM	231	HH	TYR	14	28.205	-8.368	8.699	1.00	0.00	H
ATOM	232	CE2	TYR	14	29.494	-7.800	5.833	1.00	0.00	C
ATOM	233	HE2	TYR	14	28.726	-8.238	5.215	1.00	0.00	H
ATOM	234	CD2	TYR	14	30.633	-7.216	5.200	1.00	0.00	C
ATOM	235	HD2	TYR	14	30.750	-7.191	4.128	1.00	0.00	H
ATOM	236	C	TYR	14	33.769	-3.802	4.142	1.00	0.00	C
ATOM	237	O	TYR	14	34.809	-4.431	3.829	1.00	0.00	O
ATOM	238	N	ARG	15	33.733	-2.508	3.876	1.00	0.00	N
ATOM	239	H	ARG	15	32.867	-2.096	4.194	1.00	0.00	H
ATOM	240	CA	ARG	15	34.657	-1.542	3.277	1.00	0.00	C
ATOM	241	HA	ARG	15	35.685	-1.851	3.464	1.00	0.00	H
ATOM	242	CB	ARG	15	34.540	-1.530	1.720	1.00	0.00	C
ATOM	243	HB2	ARG	15	34.569	-2.545	1.322	1.00	0.00	H
ATOM	244	HB3	ARG	15	35.434	-0.972	1.442	1.00	0.00	H
ATOM	245	CG	ARG	15	33.224	-1.138	1.146	1.00	0.00	C
ATOM	246	HG2	ARG	15	32.403	-1.818	1.374	1.00	0.00	H
ATOM	247	HG3	ARG	15	33.463	-1.152	0.083	1.00	0.00	H
ATOM	248	CD	ARG	15	32.679	0.245	1.497	1.00	0.00	C
ATOM	249	HD2	ARG	15	33.365	1.088	1.407	1.00	0.00	H
ATOM	250	HD3	ARG	15	32.383	0.155	2.542	1.00	0.00	H

ATOM	251	NE	ARG	15	31.502	0.589	0.620	1.00	0.00	N
ATOM	252	HE	ARG	15	31.385	0.189	-0.299	1.00	0.00	H
ATOM	253	CZ	ARG	15	30.679	1.615	0.742	1.00	0.00	C
ATOM	254	NH1	ARG	15	30.574	2.225	1.901	1.00	0.00	N
ATOM	255	HH11	ARG	15	31.114	1.866	2.675	1.00	0.00	H
ATOM	256	HH12	ARG	15	29.895	2.968	1.982	1.00	0.00	H
ATOM	257	NH2	ARG	15	29.920	2.019	-0.194	1.00	0.00	N
ATOM	258	HH21	ARG	15	30.059	1.699	-1.141	1.00	0.00	H
ATOM	259	HH22	ARG	15	29.369	2.856	-0.060	1.00	0.00	H
ATOM	260	C	ARG	15	34.682	-0.211	3.993	1.00	0.00	C
ATOM	261	O	ARG	15	33.697	0.185	4.651	1.00	0.00	O
ATOM	262	N	TYR	16	35.766	0.465	3.834	1.00	0.00	N
ATOM	263	H	TYR	16	36.548	-0.066	3.478	1.00	0.00	H
ATOM	264	CA	TYR	16	35.975	1.768	4.404	1.00	0.00	C
ATOM	265	HA	TYR	16	35.917	1.596	5.480	1.00	0.00	H
ATOM	266	CB	TYR	16	37.427	2.217	4.086	1.00	0.00	C
ATOM	267	HB2	TYR	16	37.466	2.428	3.017	1.00	0.00	H
ATOM	268	HB3	TYR	16	37.929	1.291	4.366	1.00	0.00	H
ATOM	269	CG	TYR	16	38.041	3.379	4.961	1.00	0.00	C
ATOM	270	CD1	TYR	16	38.706	3.091	6.155	1.00	0.00	C
ATOM	271	HD1	TYR	16	38.743	2.072	6.512	1.00	0.00	H
ATOM	272	CE1	TYR	16	39.255	4.164	6.998	1.00	0.00	C
ATOM	273	HE1	TYR	16	39.733	3.980	7.950	1.00	0.00	H
ATOM	274	CZ	TYR	16	39.064	5.471	6.544	1.00	0.00	C
ATOM	275	OH	TYR	16	39.578	6.522	7.241	1.00	0.00	O
ATOM	276	HH	TYR	16	39.978	6.036	7.966	1.00	0.00	H
ATOM	277	CE2	TYR	16	38.416	5.783	5.352	1.00	0.00	C
ATOM	278	HE2	TYR	16	38.196	6.815	5.122	1.00	0.00	H
ATOM	279	CD2	TYR	16	37.909	4.704	4.568	1.00	0.00	C
ATOM	280	HD2	TYR	16	37.263	4.977	3.746	1.00	0.00	H
ATOM	281	C	TYR	16	34.914	2.851	3.971	1.00	0.00	C
ATOM	282	O	TYR	16	34.238	2.817	2.896	1.00	0.00	O
ATOM	283	N	NME	17	34.931	3.956	4.757	1.00	0.00	N
ATOM	284	H	NME	17	35.680	4.092	5.421	1.00	0.00	H
ATOM	285	CH3	NME	17	34.023	5.029	4.749	1.00	0.00	C
ATOM	286	HH31	NME	17	34.271	5.824	4.045	1.00	0.00	H
ATOM	287	HH32	NME	17	33.044	4.603	4.526	1.00	0.00	H
ATOM	288	HH33	NME	17	33.980	5.500	5.730	1.00	0.00	H
TER	289		NME	17						
ATOM	289	HH31	ACE	18	12.772	13.365	9.473	1.00	0.00	H
ATOM	290	CH3	ACE	18	11.707	13.239	9.279	1.00	0.00	C
ATOM	291	HH32	ACE	18	11.171	14.177	9.426	1.00	0.00	H
ATOM	292	HH33	ACE	18	11.369	12.524	10.029	1.00	0.00	H
ATOM	293	C	ACE	18	11.441	12.678	7.933	1.00	0.00	C
ATOM	294	O	ACE	18	11.171	11.446	7.795	1.00	0.00	O
ATOM	295	N	ASN	19	11.562	13.537	6.959	1.00	0.00	N
ATOM	296	H	ASN	19	11.861	14.473	7.188	1.00	0.00	H
ATOM	297	CA	ASN	19	11.285	13.223	5.599	1.00	0.00	C
ATOM	298	HA	ASN	19	10.332	12.701	5.518	1.00	0.00	H
ATOM	299	CB	ASN	19	11.088	14.540	4.761	1.00	0.00	C
ATOM	300	HB2	ASN	19	11.742	15.396	4.929	1.00	0.00	H
ATOM	301	HB3	ASN	19	10.081	14.908	4.963	1.00	0.00	H
ATOM	302	CG	ASN	19	11.149	14.220	3.208	1.00	0.00	C
ATOM	303	OD1	ASN	19	10.741	13.162	2.758	1.00	0.00	O
ATOM	304	ND2	ASN	19	11.574	15.199	2.443	1.00	0.00	N
ATOM	305	HD21	ASN	19	11.493	14.977	1.462	1.00	0.00	H
ATOM	306	HD22	ASN	19	12.164	15.919	2.836	1.00	0.00	H
ATOM	307	C	ASN	19	12.432	12.344	5.114	1.00	0.00	C
ATOM	308	O	ASN	19	13.640	12.641	5.176	1.00	0.00	O
ATOM	309	N	ASP	20	11.993	11.202	4.604	1.00	0.00	N
ATOM	310	H	ASP	20	11.040	10.905	4.755	1.00	0.00	H
ATOM	311	CA	ASP	20	12.919	10.112	4.232	1.00	0.00	C
ATOM	312	HA	ASP	20	13.454	9.863	5.149	1.00	0.00	H

ATOM	313	CB	ASP	20	12.173	8.846	3.755	1.00	0.00	C
ATOM	314	HB2	ASP	20	11.508	9.054	2.917	1.00	0.00	H
ATOM	315	HB3	ASP	20	11.598	8.541	4.628	1.00	0.00	H
ATOM	316	CG	ASP	20	12.971	7.640	3.306	1.00	0.00	C
ATOM	317	OD1	ASP	20	13.463	6.844	4.177	1.00	0.00	O
ATOM	318	OD2	ASP	20	13.335	7.496	2.087	1.00	0.00	O
ATOM	319	C	ASP	20	13.930	10.525	3.216	1.00	0.00	C
ATOM	320	O	ASP	20	15.103	10.156	3.347	1.00	0.00	O
ATOM	321	N	TYR	21	13.543	11.200	2.166	1.00	0.00	N
ATOM	322	H	TYR	21	12.558	11.323	1.983	1.00	0.00	H
ATOM	323	CA	TYR	21	14.370	11.398	0.947	1.00	0.00	C
ATOM	324	HA	TYR	21	14.796	10.436	0.664	1.00	0.00	H
ATOM	325	CB	TYR	21	13.404	11.897	-0.058	1.00	0.00	C
ATOM	326	HB2	TYR	21	13.950	12.149	-0.968	1.00	0.00	H
ATOM	327	HB3	TYR	21	12.857	12.744	0.353	1.00	0.00	H
ATOM	328	CG	TYR	21	12.388	10.843	-0.536	1.00	0.00	C
ATOM	329	CD1	TYR	21	12.697	10.030	-1.686	1.00	0.00	C
ATOM	330	HD1	TYR	21	13.618	10.149	-2.236	1.00	0.00	H
ATOM	331	CE1	TYR	21	11.777	9.023	-2.062	1.00	0.00	C
ATOM	332	HE1	TYR	21	12.008	8.469	-2.959	1.00	0.00	H
ATOM	333	CZ	TYR	21	10.606	8.723	-1.319	1.00	0.00	C
ATOM	334	OH	TYR	21	9.917	7.587	-1.598	1.00	0.00	O
ATOM	335	HH	TYR	21	10.340	7.164	-2.349	1.00	0.00	H
ATOM	336	CE2	TYR	21	10.366	9.470	-0.140	1.00	0.00	C
ATOM	337	HE2	TYR	21	9.524	9.219	0.489	1.00	0.00	H
ATOM	338	CD2	TYR	21	11.195	10.574	0.195	1.00	0.00	C
ATOM	339	HD2	TYR	21	10.960	11.119	1.098	1.00	0.00	H
ATOM	340	C	TYR	21	15.541	12.360	1.067	1.00	0.00	C
ATOM	341	O	TYR	21	16.443	12.406	0.207	1.00	0.00	O
ATOM	342	N	GLU	22	15.621	13.078	2.178	1.00	0.00	N
ATOM	343	H	GLU	22	14.763	13.153	2.705	1.00	0.00	H
ATOM	344	CA	GLU	22	16.711	14.050	2.485	1.00	0.00	C
ATOM	345	HA	GLU	22	17.171	14.355	1.545	1.00	0.00	H
ATOM	346	CB	GLU	22	16.129	15.151	3.283	1.00	0.00	C
ATOM	347	HB2	GLU	22	16.993	15.608	3.764	1.00	0.00	H
ATOM	348	HB3	GLU	22	15.430	14.707	3.992	1.00	0.00	H
ATOM	349	CG	GLU	22	15.455	16.164	2.449	1.00	0.00	C
ATOM	350	HG2	GLU	22	14.601	15.685	1.971	1.00	0.00	H
ATOM	351	HG3	GLU	22	16.158	16.543	1.707	1.00	0.00	H
ATOM	352	CD	GLU	22	14.773	17.322	3.266	1.00	0.00	C
ATOM	353	OE1	GLU	22	13.547	17.309	3.450	1.00	0.00	O
ATOM	354	OE2	GLU	22	15.454	18.400	3.461	1.00	0.00	O
ATOM	355	C	GLU	22	17.802	13.312	3.331	1.00	0.00	C
ATOM	356	O	GLU	22	18.988	13.550	3.216	1.00	0.00	O
ATOM	357	N	ASP	23	17.349	12.417	4.204	1.00	0.00	N
ATOM	358	H	ASP	23	16.375	12.152	4.190	1.00	0.00	H
ATOM	359	CA	ASP	23	18.211	11.709	5.134	1.00	0.00	C
ATOM	360	HA	ASP	23	18.909	12.432	5.555	1.00	0.00	H
ATOM	361	CB	ASP	23	17.315	11.052	6.111	1.00	0.00	C
ATOM	362	HB2	ASP	23	16.529	10.546	5.550	1.00	0.00	H
ATOM	363	HB3	ASP	23	17.006	11.816	6.824	1.00	0.00	H
ATOM	364	CG	ASP	23	17.917	9.920	7.034	1.00	0.00	C
ATOM	365	OD1	ASP	23	17.444	8.761	6.887	1.00	0.00	O
ATOM	366	OD2	ASP	23	18.643	10.204	8.021	1.00	0.00	O
ATOM	367	C	ASP	23	19.115	10.720	4.427	1.00	0.00	C
ATOM	368	O	ASP	23	20.029	10.202	5.033	1.00	0.00	O
ATOM	369	N	ARG	24	18.986	10.521	3.103	1.00	0.00	N
ATOM	370	H	ARG	24	18.220	11.009	2.665	1.00	0.00	H
ATOM	371	CA	ARG	24	19.822	9.580	2.334	1.00	0.00	C
ATOM	372	HA	ARG	24	19.640	8.518	2.503	1.00	0.00	H
ATOM	373	CB	ARG	24	19.329	9.762	0.813	1.00	0.00	C
ATOM	374	HB2	ARG	24	20.037	9.246	0.163	1.00	0.00	H
ATOM	375	HB3	ARG	24	19.399	10.847	0.758	1.00	0.00	H

ATOM	376	CG	ARG	24	17.884	9.297	0.399	1.00	0.00	C
ATOM	377	HG2	ARG	24	17.487	9.797	-0.484	1.00	0.00	H
ATOM	378	HG3	ARG	24	17.189	9.612	1.177	1.00	0.00	H
ATOM	379	CD	ARG	24	17.518	7.860	0.286	1.00	0.00	C
ATOM	380	HD2	ARG	24	17.937	7.186	1.034	1.00	0.00	H
ATOM	381	HD3	ARG	24	18.038	7.575	-0.628	1.00	0.00	H
ATOM	382	NE	ARG	24	16.079	7.639	0.181	1.00	0.00	N
ATOM	383	HE	ARG	24	15.575	7.748	1.049	1.00	0.00	H
ATOM	384	CZ	ARG	24	15.336	7.313	-0.883	1.00	0.00	C
ATOM	385	NH1	ARG	24	15.773	7.114	-2.085	1.00	0.00	N
ATOM	386	HH11	ARG	24	16.752	7.160	-2.327	1.00	0.00	H
ATOM	387	HH12	ARG	24	15.197	6.813	-2.858	1.00	0.00	H
ATOM	388	NH2	ARG	24	14.111	7.234	-0.709	1.00	0.00	N
ATOM	389	HH21	ARG	24	13.710	7.332	0.213	1.00	0.00	H
ATOM	390	HH22	ARG	24	13.552	6.870	-1.466	1.00	0.00	H
ATOM	391	C	ARG	24	21.313	9.872	2.443	1.00	0.00	C
ATOM	392	O	ARG	24	22.148	9.026	2.144	1.00	0.00	O
ATOM	393	N	TYR	25	21.697	11.093	2.939	1.00	0.00	N
ATOM	394	H	TYR	25	21.037	11.855	2.993	1.00	0.00	H
ATOM	395	CA	TYR	25	23.064	11.381	3.149	1.00	0.00	C
ATOM	396	HA	TYR	25	23.561	11.103	2.219	1.00	0.00	H
ATOM	397	CB	TYR	25	23.170	12.907	3.439	1.00	0.00	C
ATOM	398	HB2	TYR	25	22.579	13.511	2.750	1.00	0.00	H
ATOM	399	HB3	TYR	25	24.203	13.094	3.144	1.00	0.00	H
ATOM	400	CG	TYR	25	23.027	13.367	4.836	1.00	0.00	C
ATOM	401	CD1	TYR	25	24.121	13.205	5.762	1.00	0.00	C
ATOM	402	HD1	TYR	25	24.978	12.658	5.397	1.00	0.00	H
ATOM	403	CE1	TYR	25	23.950	13.578	7.082	1.00	0.00	C
ATOM	404	HE1	TYR	25	24.699	13.359	7.827	1.00	0.00	H
ATOM	405	CZ	TYR	25	22.751	14.186	7.531	1.00	0.00	C
ATOM	406	OH	TYR	25	22.338	14.451	8.819	1.00	0.00	O
ATOM	407	HH	TYR	25	23.020	14.275	9.472	1.00	0.00	H
ATOM	408	CE2	TYR	25	21.792	14.491	6.610	1.00	0.00	C
ATOM	409	HE2	TYR	25	20.962	15.057	7.007	1.00	0.00	H
ATOM	410	CD2	TYR	25	21.938	14.163	5.258	1.00	0.00	C
ATOM	411	HD2	TYR	25	21.095	14.444	4.645	1.00	0.00	H
ATOM	412	C	TYR	25	23.697	10.493	4.233	1.00	0.00	C
ATOM	413	O	TYR	25	24.768	9.880	3.992	1.00	0.00	O
ATOM	414	N	TYR	26	23.051	10.330	5.372	1.00	0.00	N
ATOM	415	H	TYR	26	22.197	10.846	5.532	1.00	0.00	H
ATOM	416	CA	TYR	26	23.529	9.565	6.511	1.00	0.00	C
ATOM	417	HA	TYR	26	24.549	9.868	6.747	1.00	0.00	H
ATOM	418	CB	TYR	26	22.709	9.962	7.812	1.00	0.00	C
ATOM	419	HB2	TYR	26	21.662	9.692	7.682	1.00	0.00	H
ATOM	420	HB3	TYR	26	22.575	11.042	7.872	1.00	0.00	H
ATOM	421	CG	TYR	26	23.062	9.503	9.195	1.00	0.00	C
ATOM	422	CD1	TYR	26	22.663	8.291	9.740	1.00	0.00	C
ATOM	423	HD1	TYR	26	21.901	7.740	9.210	1.00	0.00	H
ATOM	424	CE1	TYR	26	23.125	7.850	10.997	1.00	0.00	C
ATOM	425	HE1	TYR	26	22.812	6.898	11.399	1.00	0.00	H
ATOM	426	CZ	TYR	26	24.085	8.662	11.729	1.00	0.00	C
ATOM	427	OH	TYR	26	24.528	8.261	12.947	1.00	0.00	O
ATOM	428	HH	TYR	26	24.175	7.371	13.019	1.00	0.00	H
ATOM	429	CE2	TYR	26	24.549	9.845	11.100	1.00	0.00	C
ATOM	430	HE2	TYR	26	25.306	10.374	11.661	1.00	0.00	H
ATOM	431	CD2	TYR	26	23.995	10.348	9.906	1.00	0.00	C
ATOM	432	HD2	TYR	26	24.177	11.363	9.584	1.00	0.00	H
ATOM	433	C	TYR	26	23.468	8.054	6.313	1.00	0.00	C
ATOM	434	O	TYR	26	24.200	7.385	7.030	1.00	0.00	O
ATOM	435	N	ARG	27	22.771	7.512	5.304	1.00	0.00	N
ATOM	436	H	ARG	27	22.214	8.158	4.765	1.00	0.00	H
ATOM	437	CA	ARG	27	22.737	6.063	4.896	1.00	0.00	C
ATOM	438	HA	ARG	27	22.444	5.534	5.803	1.00	0.00	H

ATOM	439	CB	ARG	27	21.624	5.775	3.908	1.00	0.00	C
ATOM	440	HB2	ARG	27	21.479	4.715	3.700	1.00	0.00	H
ATOM	441	HB3	ARG	27	21.941	6.370	3.052	1.00	0.00	H
ATOM	442	CG	ARG	27	20.280	6.362	4.482	1.00	0.00	C
ATOM	443	HG2	ARG	27	20.348	7.429	4.265	1.00	0.00	H
ATOM	444	HG3	ARG	27	20.437	6.357	5.560	1.00	0.00	H
ATOM	445	CD	ARG	27	18.930	5.789	4.109	1.00	0.00	C
ATOM	446	HD2	ARG	27	18.827	4.715	4.256	1.00	0.00	H
ATOM	447	HD3	ARG	27	18.888	5.913	3.027	1.00	0.00	H
ATOM	448	NE	ARG	27	17.840	6.401	4.918	1.00	0.00	N
ATOM	449	HE	ARG	27	18.133	6.943	5.717	1.00	0.00	H
ATOM	450	CZ	ARG	27	16.595	6.483	4.592	1.00	0.00	C
ATOM	451	NH1	ARG	27	15.962	5.736	3.704	1.00	0.00	N
ATOM	452	HH11	ARG	27	14.968	5.902	3.648	1.00	0.00	H
ATOM	453	HH12	ARG	27	16.324	4.978	3.143	1.00	0.00	H
ATOM	454	NH2	ARG	27	15.840	7.303	5.305	1.00	0.00	N
ATOM	455	HH21	ARG	27	14.832	7.260	5.244	1.00	0.00	H
ATOM	456	HH22	ARG	27	16.262	7.739	6.112	1.00	0.00	H
ATOM	457	C	ARG	27	24.102	5.573	4.281	1.00	0.00	C
ATOM	458	O	ARG	27	24.318	4.356	4.187	1.00	0.00	O
ATOM	459	N	GLU	28	25.048	6.526	3.935	1.00	0.00	N
ATOM	460	H	GLU	28	24.767	7.496	3.970	1.00	0.00	H
ATOM	461	CA	GLU	28	26.413	6.171	3.488	1.00	0.00	C
ATOM	462	HA	GLU	28	26.514	5.087	3.543	1.00	0.00	H
ATOM	463	CB	GLU	28	26.658	6.677	2.036	1.00	0.00	C
ATOM	464	HB2	GLU	28	26.398	7.732	1.949	1.00	0.00	H
ATOM	465	HB3	GLU	28	25.979	6.079	1.428	1.00	0.00	H
ATOM	466	CG	GLU	28	28.026	6.467	1.550	1.00	0.00	C
ATOM	467	HG2	GLU	28	28.757	7.057	2.104	1.00	0.00	H
ATOM	468	HG3	GLU	28	28.037	6.874	0.539	1.00	0.00	H
ATOM	469	CD	GLU	28	28.587	5.030	1.422	1.00	0.00	C
ATOM	470	OE1	GLU	28	28.935	4.407	2.480	1.00	0.00	O
ATOM	471	OE2	GLU	28	28.619	4.518	0.326	1.00	0.00	O
ATOM	472	C	GLU	28	27.423	6.705	4.537	1.00	0.00	C
ATOM	473	O	GLU	28	27.435	7.873	4.930	1.00	0.00	O
ATOM	474	N	ASN	29	28.337	5.828	4.925	1.00	0.00	N
ATOM	475	H	ASN	29	28.438	4.899	4.542	1.00	0.00	H
ATOM	476	CA	ASN	29	29.327	6.033	5.969	1.00	0.00	C
ATOM	477	HA	ASN	29	28.833	6.349	6.887	1.00	0.00	H
ATOM	478	CB	ASN	29	30.103	4.737	6.162	1.00	0.00	C
ATOM	479	HB2	ASN	29	30.804	4.496	5.363	1.00	0.00	H
ATOM	480	HB3	ASN	29	29.335	3.964	6.159	1.00	0.00	H
ATOM	481	CG	ASN	29	30.888	4.512	7.472	1.00	0.00	C
ATOM	482	OD1	ASN	29	32.071	4.268	7.425	1.00	0.00	O
ATOM	483	ND2	ASN	29	30.172	4.375	8.606	1.00	0.00	N
ATOM	484	HD21	ASN	29	30.582	4.203	9.513	1.00	0.00	H
ATOM	485	HD22	ASN	29	29.170	4.499	8.610	1.00	0.00	H
ATOM	486	C	ASN	29	30.397	7.132	5.728	1.00	0.00	C
ATOM	487	O	ASN	29	30.832	7.783	6.728	1.00	0.00	O
ATOM	488	N	MET	30	30.900	7.320	4.471	1.00	0.00	N
ATOM	489	H	MET	30	30.657	6.640	3.765	1.00	0.00	H
ATOM	490	CA	MET	30	31.676	8.539	4.107	1.00	0.00	C
ATOM	491	HA	MET	30	32.472	8.708	4.833	1.00	0.00	H
ATOM	492	CB	MET	30	32.229	8.244	2.726	1.00	0.00	C
ATOM	493	HB2	MET	30	31.365	7.924	2.142	1.00	0.00	H
ATOM	494	HB3	MET	30	32.832	7.373	2.979	1.00	0.00	H
ATOM	495	CG	MET	30	33.033	9.394	2.092	1.00	0.00	C
ATOM	496	HG2	MET	30	33.617	9.984	2.800	1.00	0.00	H
ATOM	497	HG3	MET	30	32.251	10.045	1.703	1.00	0.00	H
ATOM	498	SD	MET	30	34.162	8.815	0.811	1.00	0.00	S
ATOM	499	CE	MET	30	34.829	10.431	0.256	1.00	0.00	C
ATOM	500	HE1	MET	30	34.206	11.228	0.663	1.00	0.00	H
ATOM	501	HE2	MET	30	35.845	10.567	0.629	1.00	0.00	H

ATOM	502	HE3	MET	30	34.862	10.549	-0.826	1.00	0.00	H
ATOM	503	C	MET	30	30.920	9.807	4.188	1.00	0.00	C
ATOM	504	O	MET	30	31.529	10.820	4.532	1.00	0.00	O
ATOM	505	N	TYR	31	29.652	9.795	3.788	1.00	0.00	N
ATOM	506	H	TYR	31	29.182	8.928	3.568	1.00	0.00	H
ATOM	507	CA	TYR	31	28.875	10.999	3.591	1.00	0.00	C
ATOM	508	HA	TYR	31	29.431	11.670	2.935	1.00	0.00	H
ATOM	509	CB	TYR	31	27.660	10.627	2.704	1.00	0.00	C
ATOM	510	HB2	TYR	31	26.767	11.183	2.987	1.00	0.00	H
ATOM	511	HB3	TYR	31	27.447	9.573	2.883	1.00	0.00	H
ATOM	512	CG	TYR	31	27.863	10.928	1.215	1.00	0.00	C
ATOM	513	CD1	TYR	31	27.394	12.143	0.700	1.00	0.00	C
ATOM	514	HD1	TYR	31	26.800	12.862	1.243	1.00	0.00	H
ATOM	515	CE1	TYR	31	27.810	12.607	-0.554	1.00	0.00	C
ATOM	516	HE1	TYR	31	27.371	13.436	-1.089	1.00	0.00	H
ATOM	517	CZ	TYR	31	28.718	11.821	-1.307	1.00	0.00	C
ATOM	518	OH	TYR	31	29.139	12.338	-2.529	1.00	0.00	O
ATOM	519	HH	TYR	31	29.636	11.733	-3.087	1.00	0.00	H
ATOM	520	CE2	TYR	31	29.088	10.543	-0.881	1.00	0.00	C
ATOM	521	HE2	TYR	31	29.590	9.907	-1.596	1.00	0.00	H
ATOM	522	CD2	TYR	31	28.765	10.124	0.438	1.00	0.00	C
ATOM	523	HD2	TYR	31	29.253	9.253	0.849	1.00	0.00	H
ATOM	524	C	TYR	31	28.336	11.723	4.903	1.00	0.00	C
ATOM	525	O	TYR	31	27.861	12.881	4.826	1.00	0.00	O
ATOM	526	N	ARG	32	28.453	11.041	6.093	1.00	0.00	N
ATOM	527	H	ARG	32	28.865	10.126	5.977	1.00	0.00	H
ATOM	528	CA	ARG	32	28.286	11.668	7.420	1.00	0.00	C
ATOM	529	HA	ARG	32	27.272	12.052	7.532	1.00	0.00	H
ATOM	530	CB	ARG	32	28.487	10.555	8.487	1.00	0.00	C
ATOM	531	HB2	ARG	32	28.502	11.050	9.458	1.00	0.00	H
ATOM	532	HB3	ARG	32	29.491	10.222	8.225	1.00	0.00	H
ATOM	533	CG	ARG	32	27.486	9.397	8.357	1.00	0.00	C
ATOM	534	HG2	ARG	32	27.687	8.830	7.449	1.00	0.00	H
ATOM	535	HG3	ARG	32	26.557	9.913	8.116	1.00	0.00	H
ATOM	536	CD	ARG	32	27.227	8.346	9.479	1.00	0.00	C
ATOM	537	HD2	ARG	32	26.827	8.775	10.398	1.00	0.00	H
ATOM	538	HD3	ARG	32	28.218	7.908	9.599	1.00	0.00	H
ATOM	539	NE	ARG	32	26.327	7.342	9.005	1.00	0.00	N
ATOM	540	HE	ARG	32	25.545	7.665	8.453	1.00	0.00	H
ATOM	541	CZ	ARG	32	26.308	6.047	9.153	1.00	0.00	C
ATOM	542	NH1	ARG	32	27.015	5.431	10.059	1.00	0.00	N
ATOM	543	HH11	ARG	32	27.544	5.970	10.728	1.00	0.00	H
ATOM	544	HH12	ARG	32	27.285	4.458	10.028	1.00	0.00	H
ATOM	545	NH2	ARG	32	25.558	5.318	8.453	1.00	0.00	N
ATOM	546	HH21	ARG	32	24.835	5.674	7.845	1.00	0.00	H
ATOM	547	HH22	ARG	32	25.474	4.356	8.751	1.00	0.00	H
ATOM	548	C	ARG	32	29.236	12.866	7.750	1.00	0.00	C
ATOM	549	O	ARG	32	28.942	13.695	8.622	1.00	0.00	O
ATOM	550	N	TYR	33	30.366	13.007	7.037	1.00	0.00	N
ATOM	551	H	TYR	33	30.562	12.252	6.395	1.00	0.00	H
ATOM	552	CA	TYR	33	31.343	14.098	7.141	1.00	0.00	C
ATOM	553	HA	TYR	33	30.976	14.995	7.639	1.00	0.00	H
ATOM	554	CB	TYR	33	32.526	13.532	7.872	1.00	0.00	C
ATOM	555	HB2	TYR	33	32.205	13.343	8.897	1.00	0.00	H
ATOM	556	HB3	TYR	33	33.351	14.241	7.798	1.00	0.00	H
ATOM	557	CG	TYR	33	33.170	12.159	7.476	1.00	0.00	C
ATOM	558	CD1	TYR	33	32.646	10.964	7.986	1.00	0.00	C
ATOM	559	HD1	TYR	33	31.964	10.999	8.822	1.00	0.00	H
ATOM	560	CE1	TYR	33	33.196	9.720	7.574	1.00	0.00	C
ATOM	561	HE1	TYR	33	32.830	8.812	8.029	1.00	0.00	H
ATOM	562	CZ	TYR	33	34.267	9.629	6.657	1.00	0.00	C
ATOM	563	OH	TYR	33	34.864	8.446	6.373	1.00	0.00	O
ATOM	564	HH	TYR	33	35.675	8.487	5.862	1.00	0.00	H

```
ATOM    565  CE2 TYR    33      34.739  10.834   6.063  1.00  0.00           C
ATOM    566  HE2 TYR    33      35.533  10.806   5.331  1.00  0.00           H
ATOM    567  CD2 TYR    33      34.164  12.052   6.483  1.00  0.00           C
ATOM    568  HD2 TYR    33      34.502  13.012   6.119  1.00  0.00           H
ATOM    569  C   TYR    33      31.760  14.558   5.726  1.00  0.00           C
ATOM    570  O   TYR    33      31.871  15.777   5.572  1.00  0.00           O
ATOM    571  N   NME    34      31.979  13.719   4.671  1.00  0.00           N
ATOM    572  H   NME    34      31.858  12.730   4.836  1.00  0.00           H
ATOM    573  CH3 NME    34      32.531  14.160   3.418  1.00  0.00           C
ATOM    574 HH31 NME    34      31.748  14.654   2.845  1.00  0.00           H
ATOM    575 HH32 NME    34      33.368  14.859   3.406  1.00  0.00           H
ATOM    576 HH33 NME    34      32.680  13.247   2.840  1.00  0.00           H
TER     577      NME    34
END
```

H1 + K^{197}GENFTETDIKIMER^{211} simulation:

```
#Cluster   Frames      Frac  AvgDist    Stdev Centroid AvgCDist
       0    86370     0.864    1.717    0.629    38998    4.508
       1     8611     0.086    0.920    0.337    73013    5.719
       2     4190     0.042    2.482    1.141    21884    4.695
       3      273     0.003    2.399    0.636      210    4.855
       4      272     0.003    2.098    0.914     8424    4.874
       5      187     0.002    2.085    0.437    35596    5.072
       6       97     0.001    1.878    0.587    19267    5.052
```

Cluster 0, Figure 4A:

```
ATOM      1 HH31 ACE     1      37.886   6.400   7.360  1.00  0.00           H
ATOM      2  CH3 ACE     1      37.943   6.108   8.409  1.00  0.00           C
ATOM      3 HH32 ACE     1      38.678   6.564   9.073  1.00  0.00           H
ATOM      4 HH33 ACE     1      38.247   5.063   8.445  1.00  0.00           H
ATOM      5  C   ACE     1      36.569   6.284   9.096  1.00  0.00           C
ATOM      6  O   ACE     1      36.104   5.375   9.797  1.00  0.00           O
ATOM      7  N   ASN     2      35.999   7.504   8.922  1.00  0.00           N
ATOM      8  H   ASN     2      36.592   8.216   8.522  1.00  0.00           H
ATOM      9  CA  ASN     2      34.703   7.919   9.519  1.00  0.00           C
ATOM     10  HA  ASN     2      34.463   7.310  10.391  1.00  0.00           H
ATOM     11  CB  ASN     2      34.819   9.469   9.930  1.00  0.00           C
ATOM     12  HB2 ASN     2      34.788  10.060   9.015  1.00  0.00           H
ATOM     13  HB3 ASN     2      35.800   9.761  10.304  1.00  0.00           H
ATOM     14  CG  ASN     2      33.735   9.894  10.843  1.00  0.00           C
ATOM     15  OD1 ASN     2      32.663  10.368  10.427  1.00  0.00           O
ATOM     16  ND2 ASN     2      33.736   9.387  12.054  1.00  0.00           N
ATOM     17 HD21 ASN     2      32.961   9.702  12.619  1.00  0.00           H
ATOM     18 HD22 ASN     2      34.488   8.818  12.413  1.00  0.00           H
ATOM     19  C   ASN     2      33.544   7.704   8.613  1.00  0.00           C
ATOM     20  O   ASN     2      33.695   7.770   7.429  1.00  0.00           O
ATOM     21  N   ASP     3      32.342   7.490   9.189  1.00  0.00           N
ATOM     22  H   ASP     3      32.151   7.552  10.179  1.00  0.00           H
ATOM     23  CA  ASP     3      31.068   7.284   8.384  1.00  0.00           C
ATOM     24  HA  ASP     3      31.308   6.446   7.730  1.00  0.00           H
ATOM     25  CB  ASP     3      29.857   6.933   9.196  1.00  0.00           C
ATOM     26  HB2 ASP     3      29.663   7.753   9.887  1.00  0.00           H
ATOM     27  HB3 ASP     3      30.142   6.005   9.695  1.00  0.00           H
ATOM     28  CG  ASP     3      28.598   6.537   8.393  1.00  0.00           C
ATOM     29  OD1 ASP     3      28.682   5.523   7.694  1.00  0.00           O
ATOM     30  OD2 ASP     3      27.650   7.320   8.374  1.00  0.00           O
ATOM     31  C   ASP     3      30.695   8.508   7.473  1.00  0.00           C
ATOM     32  O   ASP     3      30.027   8.338   6.472  1.00  0.00           O
```

ATOM	33	N	TYR	4	31.145	9.731	7.797	1.00	0.00	N
ATOM	34	H	TYR	4	31.572	9.748	8.712	1.00	0.00	H
ATOM	35	CA	TYR	4	30.920	10.993	7.047	1.00	0.00	C
ATOM	36	HA	TYR	4	29.844	10.987	6.871	1.00	0.00	H
ATOM	37	CB	TYR	4	31.506	12.218	7.869	1.00	0.00	C
ATOM	38	HB2	TYR	4	32.538	11.947	8.089	1.00	0.00	H
ATOM	39	HB3	TYR	4	30.816	12.212	8.713	1.00	0.00	H
ATOM	40	CG	TYR	4	31.537	13.602	7.177	1.00	0.00	C
ATOM	41	CD1	TYR	4	32.755	14.027	6.585	1.00	0.00	C
ATOM	42	HD1	TYR	4	33.654	13.431	6.647	1.00	0.00	H
ATOM	43	CE1	TYR	4	32.855	15.247	5.874	1.00	0.00	C
ATOM	44	HE1	TYR	4	33.791	15.583	5.456	1.00	0.00	H
ATOM	45	CZ	TYR	4	31.668	16.014	5.800	1.00	0.00	C
ATOM	46	OH	TYR	4	31.822	17.208	5.150	1.00	0.00	O
ATOM	47	HH	TYR	4	30.972	17.637	5.037	1.00	0.00	H
ATOM	48	CE2	TYR	4	30.414	15.624	6.343	1.00	0.00	C
ATOM	49	HE2	TYR	4	29.529	16.236	6.245	1.00	0.00	H
ATOM	50	CD2	TYR	4	30.343	14.335	6.926	1.00	0.00	C
ATOM	51	HD2	TYR	4	29.400	14.008	7.338	1.00	0.00	H
ATOM	52	C	TYR	4	31.651	10.875	5.674	1.00	0.00	C
ATOM	53	O	TYR	4	31.124	11.277	4.641	1.00	0.00	O
ATOM	54	N	GLU	5	32.896	10.392	5.652	1.00	0.00	N
ATOM	55	H	GLU	5	33.355	10.012	6.467	1.00	0.00	H
ATOM	56	CA	GLU	5	33.709	10.325	4.425	1.00	0.00	C
ATOM	57	HA	GLU	5	33.820	11.368	4.129	1.00	0.00	H
ATOM	58	CB	GLU	5	35.150	9.873	4.711	1.00	0.00	C
ATOM	59	HB2	GLU	5	35.597	9.853	3.717	1.00	0.00	H
ATOM	60	HB3	GLU	5	35.149	8.882	5.166	1.00	0.00	H
ATOM	61	CG	GLU	5	35.968	10.794	5.603	1.00	0.00	C
ATOM	62	HG2	GLU	5	35.583	11.066	6.586	1.00	0.00	H
ATOM	63	HG3	GLU	5	35.855	11.730	5.057	1.00	0.00	H
ATOM	64	CD	GLU	5	37.430	10.302	5.734	1.00	0.00	C
ATOM	65	OE1	GLU	5	38.355	10.865	5.065	1.00	0.00	O
ATOM	66	OE2	GLU	5	37.734	9.331	6.466	1.00	0.00	O
ATOM	67	C	GLU	5	33.022	9.613	3.319	1.00	0.00	C
ATOM	68	O	GLU	5	32.959	10.131	2.207	1.00	0.00	O
ATOM	69	N	ASP	6	32.494	8.457	3.606	1.00	0.00	N
ATOM	70	H	ASP	6	32.704	7.933	4.443	1.00	0.00	H
ATOM	71	CA	ASP	6	31.643	7.598	2.729	1.00	0.00	C
ATOM	72	HA	ASP	6	32.259	7.233	1.907	1.00	0.00	H
ATOM	73	CB	ASP	6	31.249	6.377	3.566	1.00	0.00	C
ATOM	74	HB2	ASP	6	30.640	6.725	4.399	1.00	0.00	H
ATOM	75	HB3	ASP	6	32.228	6.032	3.897	1.00	0.00	H
ATOM	76	CG	ASP	6	30.387	5.464	2.800	1.00	0.00	C
ATOM	77	OD1	ASP	6	29.180	5.343	3.041	1.00	0.00	O
ATOM	78	OD2	ASP	6	30.928	4.742	1.948	1.00	0.00	O
ATOM	79	C	ASP	6	30.366	8.282	2.069	1.00	0.00	C
ATOM	80	O	ASP	6	29.871	7.882	1.010	1.00	0.00	O
ATOM	81	N	ARG	7	29.872	9.383	2.690	1.00	0.00	N
ATOM	82	H	ARG	7	30.284	9.746	3.538	1.00	0.00	H
ATOM	83	CA	ARG	7	28.662	10.028	2.146	1.00	0.00	C
ATOM	84	HA	ARG	7	28.048	9.322	1.589	1.00	0.00	H
ATOM	85	CB	ARG	7	27.791	10.670	3.247	1.00	0.00	C
ATOM	86	HB2	ARG	7	26.853	10.951	2.770	1.00	0.00	H
ATOM	87	HB3	ARG	7	28.357	11.503	3.662	1.00	0.00	H
ATOM	88	CG	ARG	7	27.396	9.729	4.382	1.00	0.00	C
ATOM	89	HG2	ARG	7	26.736	10.269	5.061	1.00	0.00	H
ATOM	90	HG3	ARG	7	28.365	9.727	4.881	1.00	0.00	H
ATOM	91	CD	ARG	7	26.820	8.377	4.067	1.00	0.00	C
ATOM	92	HD2	ARG	7	27.439	7.855	3.337	1.00	0.00	H
ATOM	93	HD3	ARG	7	25.844	8.674	3.685	1.00	0.00	H
ATOM	94	NE	ARG	7	26.560	7.590	5.228	1.00	0.00	N
ATOM	95	HE	ARG	7	26.832	8.050	6.085	1.00	0.00	H

ATOM	96	CZ	ARG	7	26.189	6.343	5.396	1.00	0.00	C
ATOM	97	NH1	ARG	7	25.932	5.472	4.423	1.00	0.00	N
ATOM	98	HH11	ARG	7	25.973	5.811	3.473	1.00	0.00	H
ATOM	99	HH12	ARG	7	25.774	4.508	4.677	1.00	0.00	H
ATOM	100	NH2	ARG	7	26.109	5.889	6.565	1.00	0.00	N
ATOM	101	HH21	ARG	7	26.720	6.304	7.254	1.00	0.00	H
ATOM	102	HH22	ARG	7	25.905	4.900	6.603	1.00	0.00	H
ATOM	103	C	ARG	7	29.002	11.093	1.019	1.00	0.00	C
ATOM	104	O	ARG	7	28.105	11.620	0.370	1.00	0.00	O
ATOM	105	N	TYR	8	30.344	11.308	0.807	1.00	0.00	N
ATOM	106	H	TYR	8	31.085	10.840	1.309	1.00	0.00	H
ATOM	107	CA	TYR	8	30.831	12.439	-0.029	1.00	0.00	C
ATOM	108	HA	TYR	8	29.942	12.981	-0.355	1.00	0.00	H
ATOM	109	CB	TYR	8	31.555	13.415	0.905	1.00	0.00	C
ATOM	110	HB2	TYR	8	32.281	12.914	1.545	1.00	0.00	H
ATOM	111	HB3	TYR	8	30.695	13.720	1.502	1.00	0.00	H
ATOM	112	CG	TYR	8	32.108	14.571	0.199	1.00	0.00	C
ATOM	113	CD1	TYR	8	33.533	14.667	0.066	1.00	0.00	C
ATOM	114	HD1	TYR	8	34.173	13.918	0.506	1.00	0.00	H
ATOM	115	CE1	TYR	8	34.167	15.820	-0.499	1.00	0.00	C
ATOM	116	HE1	TYR	8	35.241	15.907	-0.569	1.00	0.00	H
ATOM	117	CZ	TYR	8	33.365	16.876	-0.951	1.00	0.00	C
ATOM	118	OH	TYR	8	33.961	17.918	-1.601	1.00	0.00	O
ATOM	119	HH	TYR	8	34.898	17.752	-1.725	1.00	0.00	H
ATOM	120	CE2	TYR	8	31.968	16.800	-0.903	1.00	0.00	C
ATOM	121	HE2	TYR	8	31.404	17.584	-1.387	1.00	0.00	H
ATOM	122	CD2	TYR	8	31.358	15.665	-0.302	1.00	0.00	C
ATOM	123	HD2	TYR	8	30.282	15.665	-0.207	1.00	0.00	H
ATOM	124	C	TYR	8	31.720	12.031	-1.219	1.00	0.00	C
ATOM	125	O	TYR	8	31.396	12.416	-2.371	1.00	0.00	O
ATOM	126	N	TYR	9	32.843	11.223	-1.097	1.00	0.00	N
ATOM	127	H	TYR	9	33.149	10.958	-0.171	1.00	0.00	H
ATOM	128	CA	TYR	9	33.752	10.853	-2.163	1.00	0.00	C
ATOM	129	HA	TYR	9	34.038	11.829	-2.555	1.00	0.00	H
ATOM	130	CB	TYR	9	34.981	10.039	-1.564	1.00	0.00	C
ATOM	131	HB2	TYR	9	35.376	10.585	-0.708	1.00	0.00	H
ATOM	132	HB3	TYR	9	35.683	10.170	-2.388	1.00	0.00	H
ATOM	133	CG	TYR	9	34.863	8.521	-1.291	1.00	0.00	C
ATOM	134	CD1	TYR	9	35.375	7.630	-2.192	1.00	0.00	C
ATOM	135	HD1	TYR	9	35.673	7.952	-3.178	1.00	0.00	H
ATOM	136	CE1	TYR	9	35.492	6.277	-1.856	1.00	0.00	C
ATOM	137	HE1	TYR	9	35.660	5.477	-2.561	1.00	0.00	H
ATOM	138	CZ	TYR	9	35.247	5.886	-0.527	1.00	0.00	C
ATOM	139	OH	TYR	9	35.444	4.571	-0.212	1.00	0.00	O
ATOM	140	HH	TYR	9	35.539	4.093	-1.039	1.00	0.00	H
ATOM	141	CE2	TYR	9	34.889	6.835	0.481	1.00	0.00	C
ATOM	142	HE2	TYR	9	34.916	6.534	1.517	1.00	0.00	H
ATOM	143	CD2	TYR	9	34.606	8.133	0.022	1.00	0.00	C
ATOM	144	HD2	TYR	9	34.432	8.951	0.707	1.00	0.00	H
ATOM	145	C	TYR	9	33.155	10.127	-3.372	1.00	0.00	C
ATOM	146	O	TYR	9	33.498	10.362	-4.546	1.00	0.00	O
ATOM	147	N	ARG	10	32.222	9.196	-3.049	1.00	0.00	N
ATOM	148	H	ARG	10	31.901	9.220	-2.091	1.00	0.00	H
ATOM	149	CA	ARG	10	31.444	8.473	-4.007	1.00	0.00	C
ATOM	150	HA	ARG	10	31.984	8.028	-4.843	1.00	0.00	H
ATOM	151	CB	ARG	10	30.691	7.294	-3.288	1.00	0.00	C
ATOM	152	HB2	ARG	10	29.945	6.869	-3.959	1.00	0.00	H
ATOM	153	HB3	ARG	10	30.172	7.715	-2.428	1.00	0.00	H
ATOM	154	CG	ARG	10	31.573	6.193	-2.760	1.00	0.00	C
ATOM	155	HG2	ARG	10	32.255	6.622	-2.027	1.00	0.00	H
ATOM	156	HG3	ARG	10	32.260	5.930	-3.566	1.00	0.00	H
ATOM	157	CD	ARG	10	30.953	4.920	-2.152	1.00	0.00	C
ATOM	158	HD2	ARG	10	31.804	4.271	-1.945	1.00	0.00	H

ATOM	159	HD3	ARG	10	30.321	4.534	-2.953	1.00	0.00	H
ATOM	160	NE	ARG	10	30.088	5.040	-0.913	1.00	0.00	N
ATOM	161	HE	ARG	10	30.544	4.957	-0.015	1.00	0.00	H
ATOM	162	CZ	ARG	10	28.837	5.467	-0.821	1.00	0.00	C
ATOM	163	NH1	ARG	10	28.060	5.787	-1.827	1.00	0.00	N
ATOM	164	HH11	ARG	10	28.267	5.589	-2.795	1.00	0.00	H
ATOM	165	HH12	ARG	10	27.124	6.003	-1.518	1.00	0.00	H
ATOM	166	NH2	ARG	10	28.381	5.616	0.363	1.00	0.00	N
ATOM	167	HH21	ARG	10	29.023	5.613	1.142	1.00	0.00	H
ATOM	168	HH22	ARG	10	27.438	5.974	0.415	1.00	0.00	H
ATOM	169	C	ARG	10	30.394	9.365	-4.719	1.00	0.00	C
ATOM	170	O	ARG	10	29.527	9.963	-4.024	1.00	0.00	O
ATOM	171	N	GLU	11	30.366	9.369	-6.125	1.00	0.00	N
ATOM	172	H	GLU	11	31.195	9.016	-6.582	1.00	0.00	H
ATOM	173	CA	GLU	11	29.341	10.085	-6.925	1.00	0.00	C
ATOM	174	HA	GLU	11	29.126	11.048	-6.463	1.00	0.00	H
ATOM	175	CB	GLU	11	29.890	10.451	-8.316	1.00	0.00	C
ATOM	176	HB2	GLU	11	29.047	10.966	-8.775	1.00	0.00	H
ATOM	177	HB3	GLU	11	30.146	9.546	-8.867	1.00	0.00	H
ATOM	178	CG	GLU	11	31.165	11.339	-8.293	1.00	0.00	C
ATOM	179	HG2	GLU	11	32.044	10.850	-7.874	1.00	0.00	H
ATOM	180	HG3	GLU	11	30.957	12.257	-7.744	1.00	0.00	H
ATOM	181	CD	GLU	11	31.610	11.916	-9.679	1.00	0.00	C
ATOM	182	OE1	GLU	11	31.506	11.244	-10.734	1.00	0.00	O
ATOM	183	OE2	GLU	11	32.010	13.107	-9.658	1.00	0.00	O
ATOM	184	C	GLU	11	28.033	9.339	-7.057	1.00	0.00	C
ATOM	185	O	GLU	11	27.004	9.942	-7.181	1.00	0.00	O
ATOM	186	N	ASN	12	28.033	8.013	-6.905	1.00	0.00	N
ATOM	187	H	ASN	12	28.941	7.580	-6.821	1.00	0.00	H
ATOM	188	CA	ASN	12	26.884	7.159	-6.851	1.00	0.00	C
ATOM	189	HA	ASN	12	26.006	7.621	-7.302	1.00	0.00	H
ATOM	190	CB	ASN	12	27.199	5.924	-7.656	1.00	0.00	C
ATOM	191	HB2	ASN	12	27.876	5.283	-7.091	1.00	0.00	H
ATOM	192	HB3	ASN	12	27.551	6.320	-8.608	1.00	0.00	H
ATOM	193	CG	ASN	12	25.899	5.112	-7.985	1.00	0.00	C
ATOM	194	OD1	ASN	12	24.773	5.580	-7.839	1.00	0.00	O
ATOM	195	ND2	ASN	12	26.121	3.985	-8.604	1.00	0.00	N
ATOM	196	HD21	ASN	12	25.274	3.552	-8.943	1.00	0.00	H
ATOM	197	HD22	ASN	12	27.075	3.780	-8.869	1.00	0.00	H
ATOM	198	C	ASN	12	26.468	6.830	-5.405	1.00	0.00	C
ATOM	199	O	ASN	12	27.320	6.721	-4.522	1.00	0.00	O
ATOM	200	N	MET	13	25.161	6.753	-5.124	1.00	0.00	N
ATOM	201	H	MET	13	24.550	6.913	-5.912	1.00	0.00	H
ATOM	202	CA	MET	13	24.579	6.568	-3.787	1.00	0.00	C
ATOM	203	HA	MET	13	25.290	6.873	-3.020	1.00	0.00	H
ATOM	204	CB	MET	13	23.261	7.406	-3.576	1.00	0.00	C
ATOM	205	HB2	MET	13	22.792	7.082	-2.647	1.00	0.00	H
ATOM	206	HB3	MET	13	22.758	7.208	-4.524	1.00	0.00	H
ATOM	207	CG	MET	13	23.452	8.906	-3.618	1.00	0.00	C
ATOM	208	HG2	MET	13	22.518	9.400	-3.352	1.00	0.00	H
ATOM	209	HG3	MET	13	23.748	9.139	-4.641	1.00	0.00	H
ATOM	210	SD	MET	13	24.632	9.513	-2.411	1.00	0.00	S
ATOM	211	CE	MET	13	25.034	11.156	-3.175	1.00	0.00	C
ATOM	212	HE1	MET	13	25.793	11.673	-2.589	1.00	0.00	H
ATOM	213	HE2	MET	13	25.458	10.931	-4.154	1.00	0.00	H
ATOM	214	HE3	MET	13	24.100	11.717	-3.136	1.00	0.00	H
ATOM	215	C	MET	13	24.358	5.051	-3.586	1.00	0.00	C
ATOM	216	O	MET	13	23.547	4.645	-2.738	1.00	0.00	O
ATOM	217	N	TYR	14	25.094	4.181	-4.278	1.00	0.00	N
ATOM	218	H	TYR	14	25.717	4.486	-5.012	1.00	0.00	H
ATOM	219	CA	TYR	14	25.148	2.746	-4.172	1.00	0.00	C
ATOM	220	HA	TYR	14	24.166	2.433	-3.816	1.00	0.00	H
ATOM	221	CB	TYR	14	25.287	2.307	-5.617	1.00	0.00	C

ATOM	222	HB2	TYR	14	26.279	2.484	-6.033	1.00	0.00	H
ATOM	223	HB3	TYR	14	24.515	2.861	-6.151	1.00	0.00	H
ATOM	224	CG	TYR	14	24.992	0.925	-6.040	1.00	0.00	C
ATOM	225	CD1	TYR	14	26.057	0.114	-6.604	1.00	0.00	C
ATOM	226	HD1	TYR	14	27.051	0.529	-6.522	1.00	0.00	H
ATOM	227	CE1	TYR	14	25.786	-1.157	-7.133	1.00	0.00	C
ATOM	228	HE1	TYR	14	26.604	-1.807	-7.406	1.00	0.00	H
ATOM	229	CZ	TYR	14	24.448	-1.580	-7.248	1.00	0.00	C
ATOM	230	OH	TYR	14	24.109	-2.887	-7.602	1.00	0.00	O
ATOM	231	HH	TYR	14	24.823	-3.378	-8.015	1.00	0.00	H
ATOM	232	CE2	TYR	14	23.496	-0.891	-6.552	1.00	0.00	C
ATOM	233	HE2	TYR	14	22.499	-1.268	-6.380	1.00	0.00	H
ATOM	234	CD2	TYR	14	23.728	0.354	-5.982	1.00	0.00	C
ATOM	235	HD2	TYR	14	22.942	0.713	-5.333	1.00	0.00	H
ATOM	236	C	TYR	14	26.227	2.195	-3.174	1.00	0.00	C
ATOM	237	O	TYR	14	27.213	2.876	-2.778	1.00	0.00	O
ATOM	238	N	ARG	15	26.183	0.886	-2.997	1.00	0.00	N
ATOM	239	H	ARG	15	25.394	0.338	-3.307	1.00	0.00	H
ATOM	240	CA	ARG	15	27.105	0.322	-1.986	1.00	0.00	C
ATOM	241	HA	ARG	15	27.705	1.090	-1.499	1.00	0.00	H
ATOM	242	CB	ARG	15	26.188	-0.248	-0.956	1.00	0.00	C
ATOM	243	HB2	ARG	15	25.588	-1.073	-1.341	1.00	0.00	H
ATOM	244	HB3	ARG	15	25.410	0.490	-0.764	1.00	0.00	H
ATOM	245	CG	ARG	15	26.634	-0.698	0.463	1.00	0.00	C
ATOM	246	HG2	ARG	15	27.242	-1.600	0.398	1.00	0.00	H
ATOM	247	HG3	ARG	15	25.708	-0.899	1.001	1.00	0.00	H
ATOM	248	CD	ARG	15	27.588	0.136	1.273	1.00	0.00	C
ATOM	249	HD2	ARG	15	28.513	0.316	0.726	1.00	0.00	H
ATOM	250	HD3	ARG	15	27.893	-0.407	2.167	1.00	0.00	H
ATOM	251	NE	ARG	15	26.996	1.396	1.600	1.00	0.00	N
ATOM	252	HE	ARG	15	25.988	1.454	1.569	1.00	0.00	H
ATOM	253	CZ	ARG	15	27.616	2.585	1.754	1.00	0.00	C
ATOM	254	NH1	ARG	15	28.880	2.738	1.695	1.00	0.00	N
ATOM	255	HH11	ARG	15	29.498	1.946	1.599	1.00	0.00	H
ATOM	256	HH12	ARG	15	29.194	3.655	1.977	1.00	0.00	H
ATOM	257	NH2	ARG	15	26.879	3.641	1.977	1.00	0.00	N
ATOM	258	HH21	ARG	15	25.933	3.469	2.281	1.00	0.00	H
ATOM	259	HH22	ARG	15	27.361	4.494	2.221	1.00	0.00	H
ATOM	260	C	ARG	15	28.120	-0.729	-2.470	1.00	0.00	C
ATOM	261	O	ARG	15	29.092	-1.113	-1.777	1.00	0.00	O
ATOM	262	N	TYR	16	27.894	-1.276	-3.660	1.00	0.00	N
ATOM	263	H	TYR	16	27.122	-0.844	-4.147	1.00	0.00	H
ATOM	264	CA	TYR	16	28.578	-2.392	-4.345	1.00	0.00	C
ATOM	265	HA	TYR	16	29.154	-3.042	-3.687	1.00	0.00	H
ATOM	266	CB	TYR	16	27.483	-3.355	-4.829	1.00	0.00	C
ATOM	267	HB2	TYR	16	27.784	-4.325	-5.222	1.00	0.00	H
ATOM	268	HB3	TYR	16	26.902	-2.800	-5.567	1.00	0.00	H
ATOM	269	CG	TYR	16	26.441	-3.776	-3.765	1.00	0.00	C
ATOM	270	CD1	TYR	16	25.124	-3.970	-4.244	1.00	0.00	C
ATOM	271	HD1	TYR	16	24.979	-3.783	-5.298	1.00	0.00	H
ATOM	272	CE1	TYR	16	24.083	-4.404	-3.444	1.00	0.00	C
ATOM	273	HE1	TYR	16	23.048	-4.426	-3.749	1.00	0.00	H
ATOM	274	CZ	TYR	16	24.330	-4.636	-2.090	1.00	0.00	C
ATOM	275	OH	TYR	16	23.362	-5.127	-1.280	1.00	0.00	O
ATOM	276	HH	TYR	16	22.524	-5.366	-1.682	1.00	0.00	H
ATOM	277	CE2	TYR	16	25.627	-4.449	-1.521	1.00	0.00	C
ATOM	278	HE2	TYR	16	25.815	-4.662	-0.479	1.00	0.00	H
ATOM	279	CD2	TYR	16	26.718	-3.983	-2.386	1.00	0.00	C
ATOM	280	HD2	TYR	16	27.646	-3.721	-1.900	1.00	0.00	H
ATOM	281	C	TYR	16	29.545	-1.907	-5.512	1.00	0.00	C
ATOM	282	O	TYR	16	30.078	-2.761	-6.259	1.00	0.00	O
ATOM	283	N	NME	17	29.743	-0.619	-5.732	1.00	0.00	N
ATOM	284	H	NME	17	29.256	0.073	-5.182	1.00	0.00	H

```
ATOM    285  CH3 NME    17      30.638  -0.079  -6.809  1.00  0.00           C
ATOM    286 HH31 NME    17      30.954  -0.910  -7.440  1.00  0.00           H
ATOM    287 HH32 NME    17      30.188   0.701  -7.423  1.00  0.00           H
ATOM    288 HH33 NME    17      31.497   0.409  -6.348  1.00  0.00           H
TER     289      NME    17
ATOM    289 HH31 ACE    18      19.927   7.544   0.204  1.00  0.00           H
ATOM    290  CH3 ACE    18      20.011   6.890  -0.664  1.00  0.00           C
ATOM    291 HH32 ACE    18      21.003   7.035  -1.092  1.00  0.00           H
ATOM    292 HH33 ACE    18      19.362   7.260  -1.458  1.00  0.00           H
ATOM    293  C   ACE    18      19.781   5.420  -0.519  1.00  0.00           C
ATOM    294  O   ACE    18      19.535   4.867   0.617  1.00  0.00           O
ATOM    295  N   LYS    19      19.942   4.640  -1.614  1.00  0.00           N
ATOM    296  H   LYS    19      20.407   5.092  -2.389  1.00  0.00           H
ATOM    297  CA  LYS    19      19.698   3.256  -1.707  1.00  0.00           C
ATOM    298  HA  LYS    19      18.778   3.166  -1.130  1.00  0.00           H
ATOM    299  CB  LYS    19      19.463   2.962  -3.212  1.00  0.00           C
ATOM    300  HB2 LYS    19      19.303   1.896  -3.369  1.00  0.00           H
ATOM    301  HB3 LYS    19      20.366   3.231  -3.759  1.00  0.00           H
ATOM    302  CG  LYS    19      18.311   3.810  -3.817  1.00  0.00           C
ATOM    303  HG2 LYS    19      18.450   4.876  -3.638  1.00  0.00           H
ATOM    304  HG3 LYS    19      17.441   3.559  -3.210  1.00  0.00           H
ATOM    305  CD  LYS    19      18.070   3.726  -5.328  1.00  0.00           C
ATOM    306  HD2 LYS    19      17.972   2.710  -5.709  1.00  0.00           H
ATOM    307  HD3 LYS    19      18.985   4.081  -5.802  1.00  0.00           H
ATOM    308  CE  LYS    19      16.801   4.552  -5.702  1.00  0.00           C
ATOM    309  HE2 LYS    19      17.022   5.614  -5.797  1.00  0.00           H
ATOM    310  HE3 LYS    19      16.116   4.434  -4.862  1.00  0.00           H
ATOM    311  NZ  LYS    19      16.208   4.034  -7.027  1.00  0.00           N
ATOM    312  HZ1 LYS    19      15.858   3.087  -7.024  1.00  0.00           H
ATOM    313  HZ2 LYS    19      15.446   4.602  -7.368  1.00  0.00           H
ATOM    314  HZ3 LYS    19      16.900   4.218  -7.740  1.00  0.00           H
ATOM    315  C   LYS    19      20.726   2.484  -0.921  1.00  0.00           C
ATOM    316  O   LYS    19      20.355   1.499  -0.382  1.00  0.00           O
ATOM    317  N   GLY    20      21.978   2.987  -0.937  1.00  0.00           N
ATOM    318  H   GLY    20      22.113   3.883  -1.382  1.00  0.00           H
ATOM    319  CA  GLY    20      23.133   2.390  -0.232  1.00  0.00           C
ATOM    320  HA2 GLY    20      22.752   1.387  -0.040  1.00  0.00           H
ATOM    321  HA3 GLY    20      24.064   2.415  -0.797  1.00  0.00           H
ATOM    322  C   GLY    20      23.439   3.118   1.081  1.00  0.00           C
ATOM    323  O   GLY    20      24.317   2.659   1.791  1.00  0.00           O
ATOM    324  N   GLU    21      22.780   4.173   1.546  1.00  0.00           N
ATOM    325  H   GLU    21      21.967   4.489   1.036  1.00  0.00           H
ATOM    326  CA  GLU    21      23.231   5.066   2.631  1.00  0.00           C
ATOM    327  HA  GLU    21      24.312   4.934   2.648  1.00  0.00           H
ATOM    328  CB  GLU    21      23.057   6.508   2.156  1.00  0.00           C
ATOM    329  HB2 GLU    21      23.570   7.173   2.851  1.00  0.00           H
ATOM    330  HB3 GLU    21      21.986   6.713   2.146  1.00  0.00           H
ATOM    331  CG  GLU    21      23.763   6.855   0.837  1.00  0.00           C
ATOM    332  HG2 GLU    21      23.720   7.927   0.641  1.00  0.00           H
ATOM    333  HG3 GLU    21      23.240   6.358   0.019  1.00  0.00           H
ATOM    334  CD  GLU    21      25.304   6.396   0.710  1.00  0.00           C
ATOM    335  OE1 GLU    21      25.714   6.053  -0.413  1.00  0.00           O
ATOM    336  OE2 GLU    21      26.035   6.443   1.759  1.00  0.00           O
ATOM    337  C   GLU    21      22.441   4.937   3.907  1.00  0.00           C
ATOM    338  O   GLU    21      22.860   5.312   5.016  1.00  0.00           O
ATOM    339  N   ASN    22      21.183   4.483   3.850  1.00  0.00           N
ATOM    340  H   ASN    22      20.779   4.233   2.958  1.00  0.00           H
ATOM    341  CA  ASN    22      20.326   4.401   5.091  1.00  0.00           C
ATOM    342  HA  ASN    22      20.677   5.269   5.648  1.00  0.00           H
ATOM    343  CB  ASN    22      18.772   4.566   4.780  1.00  0.00           C
ATOM    344  HB2 ASN    22      18.347   3.742   4.207  1.00  0.00           H
ATOM    345  HB3 ASN    22      18.806   5.487   4.199  1.00  0.00           H
ATOM    346  CG  ASN    22      17.873   4.858   6.010  1.00  0.00           C
```

ATOM	347	OD1	ASN	22	18.369	5.267	7.037	1.00	0.00	O
ATOM	348	ND2	ASN	22	16.593	4.570	5.950	1.00	0.00	N
ATOM	349	HD21	ASN	22	16.211	5.205	6.635	1.00	0.00	H
ATOM	350	HD22	ASN	22	16.127	4.295	5.097	1.00	0.00	H
ATOM	351	C	ASN	22	20.620	3.172	6.020	1.00	0.00	C
ATOM	352	O	ASN	22	20.336	2.008	5.723	1.00	0.00	O
ATOM	353	N	PHE	23	21.076	3.497	7.263	1.00	0.00	N
ATOM	354	H	PHE	23	21.207	4.493	7.366	1.00	0.00	H
ATOM	355	CA	PHE	23	21.569	2.699	8.385	1.00	0.00	C
ATOM	356	HA	PHE	23	22.564	2.332	8.132	1.00	0.00	H
ATOM	357	CB	PHE	23	21.696	3.547	9.654	1.00	0.00	C
ATOM	358	HB2	PHE	23	22.157	3.007	10.480	1.00	0.00	H
ATOM	359	HB3	PHE	23	20.684	3.861	9.911	1.00	0.00	H
ATOM	360	CG	PHE	23	22.571	4.754	9.405	1.00	0.00	C
ATOM	361	CD1	PHE	23	23.989	4.614	9.552	1.00	0.00	C
ATOM	362	HD1	PHE	23	24.417	3.642	9.748	1.00	0.00	H
ATOM	363	CE1	PHE	23	24.786	5.767	9.363	1.00	0.00	C
ATOM	364	HE1	PHE	23	25.862	5.752	9.458	1.00	0.00	H
ATOM	365	CZ	PHE	23	24.187	6.943	8.962	1.00	0.00	C
ATOM	366	HZ	PHE	23	24.857	7.782	8.843	1.00	0.00	H
ATOM	367	CE2	PHE	23	22.828	7.085	8.750	1.00	0.00	C
ATOM	368	HE2	PHE	23	22.446	8.057	8.472	1.00	0.00	H
ATOM	369	CD2	PHE	23	22.014	5.945	9.102	1.00	0.00	C
ATOM	370	HD2	PHE	23	20.938	6.015	9.051	1.00	0.00	H
ATOM	371	C	PHE	23	20.866	1.409	8.721	1.00	0.00	C
ATOM	372	O	PHE	23	21.492	0.489	9.234	1.00	0.00	O
ATOM	373	N	THR	24	19.578	1.299	8.447	1.00	0.00	N
ATOM	374	H	THR	24	19.179	2.198	8.218	1.00	0.00	H
ATOM	375	CA	THR	24	18.577	0.256	8.755	1.00	0.00	C
ATOM	376	HA	THR	24	19.133	-0.554	9.227	1.00	0.00	H
ATOM	377	CB	THR	24	17.499	0.778	9.724	1.00	0.00	C
ATOM	378	HB	THR	24	16.836	-0.073	9.878	1.00	0.00	H
ATOM	379	CG2	THR	24	18.085	1.175	11.077	1.00	0.00	C
ATOM	380	HG21	THR	24	18.534	0.313	11.572	1.00	0.00	H
ATOM	381	HG22	THR	24	18.869	1.931	11.040	1.00	0.00	H
ATOM	382	HG23	THR	24	17.262	1.527	11.699	1.00	0.00	H
ATOM	383	OG1	THR	24	16.874	1.951	9.152	1.00	0.00	O
ATOM	384	HG1	THR	24	16.929	2.675	9.780	1.00	0.00	H
ATOM	385	C	THR	24	17.865	-0.387	7.486	1.00	0.00	C
ATOM	386	O	THR	24	17.117	-1.352	7.717	1.00	0.00	O
ATOM	387	N	GLU	25	18.359	-0.105	6.248	1.00	0.00	N
ATOM	388	H	GLU	25	18.885	0.750	6.140	1.00	0.00	H
ATOM	389	CA	GLU	25	17.858	-0.788	5.060	1.00	0.00	C
ATOM	390	HA	GLU	25	17.051	-1.468	5.336	1.00	0.00	H
ATOM	391	CB	GLU	25	17.341	0.310	4.108	1.00	0.00	C
ATOM	392	HB2	GLU	25	17.020	-0.139	3.168	1.00	0.00	H
ATOM	393	HB3	GLU	25	18.161	1.026	4.049	1.00	0.00	H
ATOM	394	CG	GLU	25	16.228	1.215	4.534	1.00	0.00	C
ATOM	395	HG2	GLU	25	16.547	1.852	5.358	1.00	0.00	H
ATOM	396	HG3	GLU	25	15.487	0.467	4.815	1.00	0.00	H
ATOM	397	CD	GLU	25	15.590	2.088	3.400	1.00	0.00	C
ATOM	398	OE1	GLU	25	15.229	1.582	2.273	1.00	0.00	O
ATOM	399	OE2	GLU	25	15.301	3.304	3.601	1.00	0.00	O
ATOM	400	C	GLU	25	18.823	-1.785	4.370	1.00	0.00	C
ATOM	401	O	GLU	25	18.464	-2.900	3.872	1.00	0.00	O
ATOM	402	N	THR	26	20.090	-1.325	4.273	1.00	0.00	N
ATOM	403	H	THR	26	20.220	-0.415	4.692	1.00	0.00	H
ATOM	404	CA	THR	26	21.195	-1.855	3.507	1.00	0.00	C
ATOM	405	HA	THR	26	21.162	-2.927	3.710	1.00	0.00	H
ATOM	406	CB	THR	26	21.130	-1.483	2.042	1.00	0.00	C
ATOM	407	HB	THR	26	22.092	-1.690	1.572	1.00	0.00	H
ATOM	408	CG2	THR	26	20.018	-2.162	1.271	1.00	0.00	C
ATOM	409	HG21	THR	26	20.286	-2.038	0.221	1.00	0.00	H

ATOM	410	HG22	THR	26	19.952	-3.236	1.440	1.00	0.00	H
ATOM	411	HG23	THR	26	19.081	-1.654	1.498	1.00	0.00	H
ATOM	412	OG1	THR	26	20.928	-0.084	1.926	1.00	0.00	O
ATOM	413	HG1	THR	26	20.770	-0.131	0.979	1.00	0.00	H
ATOM	414	C	THR	26	22.620	-1.487	3.978	1.00	0.00	C
ATOM	415	O	THR	26	23.558	-2.266	3.869	1.00	0.00	O
ATOM	416	N	ASP	27	22.767	-0.262	4.600	1.00	0.00	N
ATOM	417	H	ASP	27	21.875	0.213	4.581	1.00	0.00	H
ATOM	418	CA	ASP	27	23.984	0.382	5.035	1.00	0.00	C
ATOM	419	HA	ASP	27	24.702	0.222	4.230	1.00	0.00	H
ATOM	420	CB	ASP	27	23.692	1.910	5.098	1.00	0.00	C
ATOM	421	HB2	ASP	27	22.860	2.025	5.793	1.00	0.00	H
ATOM	422	HB3	ASP	27	23.444	2.090	4.052	1.00	0.00	H
ATOM	423	CG	ASP	27	25.029	2.643	5.383	1.00	0.00	C
ATOM	424	OD1	ASP	27	25.893	2.619	4.516	1.00	0.00	O
ATOM	425	OD2	ASP	27	25.196	3.199	6.518	1.00	0.00	O
ATOM	426	C	ASP	27	24.565	-0.153	6.384	1.00	0.00	C
ATOM	427	O	ASP	27	25.626	0.307	6.803	1.00	0.00	O
ATOM	428	N	ILE	28	23.871	-1.109	7.021	1.00	0.00	N
ATOM	429	H	ILE	28	23.070	-1.487	6.535	1.00	0.00	H
ATOM	430	CA	ILE	28	24.372	-1.852	8.184	1.00	0.00	C
ATOM	431	HA	ILE	28	24.747	-1.180	8.956	1.00	0.00	H
ATOM	432	CB	ILE	28	23.195	-2.633	8.735	1.00	0.00	C
ATOM	433	HB	ILE	28	22.402	-1.889	8.810	1.00	0.00	H
ATOM	434	CG2	ILE	28	22.636	-3.809	7.890	1.00	0.00	C
ATOM	435	HG21	ILE	28	23.378	-4.591	7.727	1.00	0.00	H
ATOM	436	HG22	ILE	28	21.713	-4.292	8.212	1.00	0.00	H
ATOM	437	HG23	ILE	28	22.273	-3.409	6.943	1.00	0.00	H
ATOM	438	CG1	ILE	28	23.516	-3.341	10.032	1.00	0.00	C
ATOM	439	HG12	ILE	28	24.117	-4.231	9.849	1.00	0.00	H
ATOM	440	HG13	ILE	28	22.500	-3.558	10.364	1.00	0.00	H
ATOM	441	CD1	ILE	28	24.252	-2.501	11.164	1.00	0.00	C
ATOM	442	HD11	ILE	28	23.635	-1.615	11.313	1.00	0.00	H
ATOM	443	HD12	ILE	28	24.355	-2.953	12.150	1.00	0.00	H
ATOM	444	HD13	ILE	28	25.209	-2.059	10.886	1.00	0.00	H
ATOM	445	C	ILE	28	25.529	-2.781	7.755	1.00	0.00	C
ATOM	446	O	ILE	28	26.300	-3.173	8.633	1.00	0.00	O
ATOM	447	N	LYS	29	25.655	-3.120	6.447	1.00	0.00	N
ATOM	448	H	LYS	29	25.085	-2.639	5.766	1.00	0.00	H
ATOM	449	CA	LYS	29	26.772	-3.942	5.985	1.00	0.00	C
ATOM	450	HA	LYS	29	27.090	-4.632	6.766	1.00	0.00	H
ATOM	451	CB	LYS	29	26.303	-4.708	4.797	1.00	0.00	C
ATOM	452	HB2	LYS	29	27.174	-5.184	4.347	1.00	0.00	H
ATOM	453	HB3	LYS	29	25.891	-3.944	4.138	1.00	0.00	H
ATOM	454	CG	LYS	29	25.290	-5.940	5.125	1.00	0.00	C
ATOM	455	HG2	LYS	29	24.950	-6.377	4.186	1.00	0.00	H
ATOM	456	HG3	LYS	29	24.504	-5.339	5.582	1.00	0.00	H
ATOM	457	CD	LYS	29	25.786	-7.092	6.029	1.00	0.00	C
ATOM	458	HD2	LYS	29	26.819	-7.318	5.766	1.00	0.00	H
ATOM	459	HD3	LYS	29	25.232	-7.989	5.752	1.00	0.00	H
ATOM	460	CE	LYS	29	25.787	-6.831	7.528	1.00	0.00	C
ATOM	461	HE2	LYS	29	24.926	-6.240	7.839	1.00	0.00	H
ATOM	462	HE3	LYS	29	26.632	-6.145	7.598	1.00	0.00	H
ATOM	463	NZ	LYS	29	25.898	-8.033	8.417	1.00	0.00	N
ATOM	464	HZ1	LYS	29	26.693	-8.611	8.185	1.00	0.00	H
ATOM	465	HZ2	LYS	29	26.070	-7.733	9.366	1.00	0.00	H
ATOM	466	HZ3	LYS	29	25.058	-8.595	8.407	1.00	0.00	H
ATOM	467	C	LYS	29	27.880	-2.892	5.687	1.00	0.00	C
ATOM	468	O	LYS	29	27.491	-1.811	5.226	1.00	0.00	O
ATOM	469	N	ILE	30	29.138	-3.273	5.876	1.00	0.00	N
ATOM	470	H	ILE	30	29.337	-4.115	6.397	1.00	0.00	H
ATOM	471	CA	ILE	30	30.233	-2.345	5.568	1.00	0.00	C
ATOM	472	HA	ILE	30	29.821	-1.481	5.047	1.00	0.00	H

ATOM	473	CB	ILE	30	30.839	-1.748	6.894	1.00	0.00	C
ATOM	474	HB	ILE	30	31.648	-1.081	6.596	1.00	0.00	H
ATOM	475	CG2	ILE	30	29.883	-0.860	7.738	1.00	0.00	C
ATOM	476	HG21	ILE	30	30.424	-0.302	8.502	1.00	0.00	H
ATOM	477	HG22	ILE	30	29.261	-0.186	7.147	1.00	0.00	H
ATOM	478	HG23	ILE	30	29.177	-1.568	8.171	1.00	0.00	H
ATOM	479	CG1	ILE	30	31.532	-2.799	7.780	1.00	0.00	C
ATOM	480	HG12	ILE	30	32.177	-3.427	7.165	1.00	0.00	H
ATOM	481	HG13	ILE	30	30.737	-3.464	8.121	1.00	0.00	H
ATOM	482	CD1	ILE	30	32.485	-2.331	8.913	1.00	0.00	C
ATOM	483	HD11	ILE	30	31.971	-1.732	9.665	1.00	0.00	H
ATOM	484	HD12	ILE	30	32.961	-3.240	9.280	1.00	0.00	H
ATOM	485	HD13	ILE	30	33.335	-1.788	8.502	1.00	0.00	H
ATOM	486	C	ILE	30	31.205	-2.852	4.569	1.00	0.00	C
ATOM	487	O	ILE	30	31.544	-4.062	4.574	1.00	0.00	O
ATOM	488	N	MET	31	31.750	-2.014	3.644	1.00	0.00	N
ATOM	489	H	MET	31	31.415	-1.064	3.570	1.00	0.00	H
ATOM	490	CA	MET	31	32.856	-2.449	2.710	1.00	0.00	C
ATOM	491	HA	MET	31	33.232	-3.436	2.979	1.00	0.00	H
ATOM	492	CB	MET	31	32.284	-2.452	1.316	1.00	0.00	C
ATOM	493	HB2	MET	31	32.990	-2.909	0.623	1.00	0.00	H
ATOM	494	HB3	MET	31	32.147	-1.427	0.973	1.00	0.00	H
ATOM	495	CG	MET	31	30.982	-3.160	1.099	1.00	0.00	C
ATOM	496	HG2	MET	31	30.573	-2.980	0.105	1.00	0.00	H
ATOM	497	HG3	MET	31	30.258	-2.814	1.836	1.00	0.00	H
ATOM	498	SD	MET	31	30.951	-5.000	1.190	1.00	0.00	S
ATOM	499	CE	MET	31	29.245	-5.280	0.801	1.00	0.00	C
ATOM	500	HE1	MET	31	29.159	-6.315	0.469	1.00	0.00	H
ATOM	501	HE2	MET	31	28.544	-5.170	1.629	1.00	0.00	H
ATOM	502	HE3	MET	31	28.943	-4.663	-0.046	1.00	0.00	H
ATOM	503	C	MET	31	34.062	-1.605	2.664	1.00	0.00	C
ATOM	504	O	MET	31	35.052	-1.969	2.064	1.00	0.00	O
ATOM	505	N	GLU	32	34.029	-0.434	3.254	1.00	0.00	N
ATOM	506	H	GLU	32	33.293	-0.359	3.942	1.00	0.00	H
ATOM	507	CA	GLU	32	34.973	0.700	3.066	1.00	0.00	C
ATOM	508	HA	GLU	32	35.963	0.375	2.744	1.00	0.00	H
ATOM	509	CB	GLU	32	34.380	1.503	1.871	1.00	0.00	C
ATOM	510	HB2	GLU	32	34.276	0.771	1.071	1.00	0.00	H
ATOM	511	HB3	GLU	32	35.186	2.180	1.585	1.00	0.00	H
ATOM	512	CG	GLU	32	33.025	2.215	2.143	1.00	0.00	C
ATOM	513	HG2	GLU	32	32.684	2.652	1.204	1.00	0.00	H
ATOM	514	HG3	GLU	32	33.191	3.035	2.844	1.00	0.00	H
ATOM	515	CD	GLU	32	31.814	1.389	2.648	1.00	0.00	C
ATOM	516	OE1	GLU	32	30.969	1.080	1.788	1.00	0.00	O
ATOM	517	OE2	GLU	32	31.693	1.148	3.872	1.00	0.00	O
ATOM	518	C	GLU	32	35.192	1.465	4.393	1.00	0.00	C
ATOM	519	O	GLU	32	36.302	1.857	4.692	1.00	0.00	O
ATOM	520	N	ARG	33	34.151	1.694	5.208	1.00	0.00	N
ATOM	521	H	ARG	33	33.244	1.432	4.852	1.00	0.00	H
ATOM	522	CA	ARG	33	34.267	2.313	6.542	1.00	0.00	C
ATOM	523	HA	ARG	33	34.743	3.266	6.310	1.00	0.00	H
ATOM	524	CB	ARG	33	32.892	2.673	6.958	1.00	0.00	C
ATOM	525	HB2	ARG	33	32.826	2.847	8.033	1.00	0.00	H
ATOM	526	HB3	ARG	33	32.238	1.846	6.678	1.00	0.00	H
ATOM	527	CG	ARG	33	32.488	4.009	6.254	1.00	0.00	C
ATOM	528	HG2	ARG	33	32.713	4.010	5.187	1.00	0.00	H
ATOM	529	HG3	ARG	33	32.857	4.765	6.946	1.00	0.00	H
ATOM	530	CD	ARG	33	30.959	4.056	6.272	1.00	0.00	C
ATOM	531	HD2	ARG	33	30.514	4.985	5.918	1.00	0.00	H
ATOM	532	HD3	ARG	33	30.623	4.015	7.308	1.00	0.00	H
ATOM	533	NE	ARG	33	30.297	2.935	5.542	1.00	0.00	N
ATOM	534	HE	ARG	33	30.823	2.350	4.909	1.00	0.00	H
ATOM	535	CZ	ARG	33	29.019	2.794	5.445	1.00	0.00	C

```
ATOM    536  NH1 ARG    33      28.148   3.584   5.972  1.00  0.00           N
ATOM    537 HH11 ARG    33      28.394   4.369   6.558  1.00  0.00           H
ATOM    538 HH12 ARG    33      27.196   3.340   5.738  1.00  0.00           H
ATOM    539  NH2 ARG    33      28.650   1.839   4.633  1.00  0.00           N
ATOM    540 HH21 ARG    33      29.322   1.297   4.109  1.00  0.00           H
ATOM    541 HH22 ARG    33      27.644   1.773   4.572  1.00  0.00           H
ATOM    542  C   ARG    33      35.002   1.395   7.587  1.00  0.00           C
ATOM    543  O   ARG    33      35.427   0.249   7.302  1.00  0.00           O
ATOM    544  N   NME    34      35.340   1.967   8.721  1.00  0.00           N
ATOM    545  H   NME    34      35.107   2.940   8.864  1.00  0.00           H
ATOM    546  CH3 NME    34      35.985   1.355   9.866  1.00  0.00           C
ATOM    547 HH31 NME    34      35.535   0.401  10.138  1.00  0.00           H
ATOM    548 HH32 NME    34      37.059   1.333   9.681  1.00  0.00           H
ATOM    549 HH33 NME    34      35.572   2.038  10.610  1.00  0.00           H
TER     550      NME    34
END
```

Cluster 1:

```
ATOM      1 HH31 ACE     1      27.381   5.884  -9.891  1.00  0.00           H
ATOM      2  CH3 ACE     1      27.989   5.966  -8.989  1.00  0.00           C
ATOM      3 HH32 ACE     1      27.297   6.106  -8.159  1.00  0.00           H
ATOM      4 HH33 ACE     1      28.774   6.721  -8.934  1.00  0.00           H
ATOM      5  C   ACE     1      28.732   4.657  -8.801  1.00  0.00           C
ATOM      6  O   ACE     1      29.538   4.374  -9.735  1.00  0.00           O
ATOM      7  N   ASN     2      28.577   3.934  -7.709  1.00  0.00           N
ATOM      8  H   ASN     2      28.019   4.395  -7.004  1.00  0.00           H
ATOM      9  CA  ASN     2      29.071   2.609  -7.251  1.00  0.00           C
ATOM     10  HA  ASN     2      29.401   2.102  -8.158  1.00  0.00           H
ATOM     11  CB  ASN     2      30.274   2.951  -6.395  1.00  0.00           C
ATOM     12  HB2 ASN     2      30.041   3.595  -5.547  1.00  0.00           H
ATOM     13  HB3 ASN     2      30.974   3.364  -7.121  1.00  0.00           H
ATOM     14  CG  ASN     2      30.867   1.679  -5.865  1.00  0.00           C
ATOM     15  OD1 ASN     2      30.565   1.228  -4.791  1.00  0.00           O
ATOM     16  ND2 ASN     2      31.749   1.006  -6.610  1.00  0.00           N
ATOM     17 HD21 ASN     2      31.790   1.253  -7.588  1.00  0.00           H
ATOM     18 HD22 ASN     2      32.295   0.218  -6.291  1.00  0.00           H
ATOM     19  C   ASN     2      27.925   1.687  -6.587  1.00  0.00           C
ATOM     20  O   ASN     2      27.149   2.286  -5.817  1.00  0.00           O
ATOM     21  N   ASP     3      27.829   0.369  -6.782  1.00  0.00           N
ATOM     22  H   ASP     3      28.500  -0.046  -7.411  1.00  0.00           H
ATOM     23  CA  ASP     3      26.798  -0.406  -6.189  1.00  0.00           C
ATOM     24  HA  ASP     3      25.854  -0.097  -6.641  1.00  0.00           H
ATOM     25  CB  ASP     3      26.974  -1.891  -6.733  1.00  0.00           C
ATOM     26  HB2 ASP     3      27.908  -2.386  -6.464  1.00  0.00           H
ATOM     27  HB3 ASP     3      26.906  -1.829  -7.818  1.00  0.00           H
ATOM     28  CG  ASP     3      25.812  -2.883  -6.249  1.00  0.00           C
ATOM     29  OD1 ASP     3      25.960  -3.562  -5.240  1.00  0.00           O
ATOM     30  OD2 ASP     3      24.715  -2.744  -6.782  1.00  0.00           O
ATOM     31  C   ASP     3      26.606  -0.339  -4.629  1.00  0.00           C
ATOM     32  O   ASP     3      25.494  -0.157  -4.177  1.00  0.00           O
ATOM     33  N   TYR     4      27.711  -0.205  -3.790  1.00  0.00           N
ATOM     34  H   TYR     4      28.623  -0.327  -4.206  1.00  0.00           H
ATOM     35  CA  TYR     4      27.563  -0.054  -2.340  1.00  0.00           C
ATOM     36  HA  TYR     4      26.715  -0.646  -2.000  1.00  0.00           H
ATOM     37  CB  TYR     4      28.832  -0.517  -1.633  1.00  0.00           C
ATOM     38  HB2 TYR     4      28.645  -0.383  -0.566  1.00  0.00           H
ATOM     39  HB3 TYR     4      29.589   0.196  -1.959  1.00  0.00           H
ATOM     40  CG  TYR     4      29.303  -1.984  -1.891  1.00  0.00           C
ATOM     41  CD1 TYR     4      28.630  -3.100  -1.295  1.00  0.00           C
ATOM     42  HD1 TYR     4      27.805  -2.870  -0.637  1.00  0.00           H
ATOM     43  CE1 TYR     4      29.011  -4.394  -1.576  1.00  0.00           C
ATOM     44  HE1 TYR     4      28.447  -5.264  -1.271  1.00  0.00           H
```

ATOM	45	CZ	TYR	4	30.129	-4.611	-2.476	1.00	0.00	C
ATOM	46	OH	TYR	4	30.459	-5.890	-2.812	1.00	0.00	O
ATOM	47	HH	TYR	4	29.711	-6.476	-2.672	1.00	0.00	H
ATOM	48	CE2	TYR	4	30.810	-3.480	-3.122	1.00	0.00	C
ATOM	49	HE2	TYR	4	31.659	-3.618	-3.775	1.00	0.00	H
ATOM	50	CD2	TYR	4	30.387	-2.199	-2.735	1.00	0.00	C
ATOM	51	HD2	TYR	4	30.871	-1.352	-3.197	1.00	0.00	H
ATOM	52	C	TYR	4	27.240	1.395	-1.952	1.00	0.00	C
ATOM	53	O	TYR	4	27.135	1.739	-0.821	1.00	0.00	O
ATOM	54	N	GLU	5	26.917	2.271	-2.874	1.00	0.00	N
ATOM	55	H	GLU	5	27.088	2.024	-3.837	1.00	0.00	H
ATOM	56	CA	GLU	5	26.592	3.685	-2.633	1.00	0.00	C
ATOM	57	HA	GLU	5	26.579	3.893	-1.564	1.00	0.00	H
ATOM	58	CB	GLU	5	27.675	4.626	-3.268	1.00	0.00	C
ATOM	59	HB2	GLU	5	27.438	5.687	-3.343	1.00	0.00	H
ATOM	60	HB3	GLU	5	27.902	4.347	-4.298	1.00	0.00	H
ATOM	61	CG	GLU	5	29.031	4.596	-2.519	1.00	0.00	C
ATOM	62	HG2	GLU	5	29.922	5.017	-2.987	1.00	0.00	H
ATOM	63	HG3	GLU	5	29.280	3.546	-2.367	1.00	0.00	H
ATOM	64	CD	GLU	5	29.044	5.219	-1.107	1.00	0.00	C
ATOM	65	OE1	GLU	5	29.386	4.598	-0.124	1.00	0.00	O
ATOM	66	OE2	GLU	5	28.840	6.456	-0.962	1.00	0.00	O
ATOM	67	C	GLU	5	25.185	4.052	-3.153	1.00	0.00	C
ATOM	68	O	GLU	5	24.230	4.402	-2.441	1.00	0.00	O
ATOM	69	N	ASP	6	25.010	3.645	-4.384	1.00	0.00	N
ATOM	70	H	ASP	6	25.826	3.336	-4.894	1.00	0.00	H
ATOM	71	CA	ASP	6	23.736	3.454	-5.199	1.00	0.00	C
ATOM	72	HA	ASP	6	23.371	4.405	-5.589	1.00	0.00	H
ATOM	73	CB	ASP	6	24.029	2.604	-6.527	1.00	0.00	C
ATOM	74	HB2	ASP	6	24.364	1.585	-6.329	1.00	0.00	H
ATOM	75	HB3	ASP	6	24.637	3.198	-7.207	1.00	0.00	H
ATOM	76	CG	ASP	6	22.685	2.319	-7.222	1.00	0.00	C
ATOM	77	OD1	ASP	6	22.077	3.254	-7.820	1.00	0.00	O
ATOM	78	OD2	ASP	6	22.185	1.156	-7.142	1.00	0.00	O
ATOM	79	C	ASP	6	22.502	2.840	-4.362	1.00	0.00	C
ATOM	80	O	ASP	6	21.331	3.329	-4.441	1.00	0.00	O
ATOM	81	N	ARG	7	22.831	1.819	-3.560	1.00	0.00	N
ATOM	82	H	ARG	7	23.723	1.388	-3.758	1.00	0.00	H
ATOM	83	CA	ARG	7	21.843	1.126	-2.751	1.00	0.00	C
ATOM	84	HA	ARG	7	20.909	1.140	-3.313	1.00	0.00	H
ATOM	85	CB	ARG	7	22.282	-0.316	-2.363	1.00	0.00	C
ATOM	86	HB2	ARG	7	23.243	-0.361	-1.852	1.00	0.00	H
ATOM	87	HB3	ARG	7	22.349	-0.694	-3.383	1.00	0.00	H
ATOM	88	CG	ARG	7	21.274	-1.157	-1.645	1.00	0.00	C
ATOM	89	HG2	ARG	7	20.261	-1.176	-2.045	1.00	0.00	H
ATOM	90	HG3	ARG	7	21.336	-0.786	-0.621	1.00	0.00	H
ATOM	91	CD	ARG	7	21.556	-2.629	-1.522	1.00	0.00	C
ATOM	92	HD2	ARG	7	20.758	-3.064	-0.921	1.00	0.00	H
ATOM	93	HD3	ARG	7	22.500	-2.644	-0.977	1.00	0.00	H
ATOM	94	NE	ARG	7	21.635	-3.259	-2.894	1.00	0.00	N
ATOM	95	HE	ARG	7	20.753	-3.422	-3.357	1.00	0.00	H
ATOM	96	CZ	ARG	7	22.718	-3.411	-3.636	1.00	0.00	C
ATOM	97	NH1	ARG	7	23.871	-3.239	-3.164	1.00	0.00	N
ATOM	98	HH11	ARG	7	24.006	-3.204	-2.163	1.00	0.00	H
ATOM	99	HH12	ARG	7	24.694	-3.286	-3.749	1.00	0.00	H
ATOM	100	NH2	ARG	7	22.671	-3.666	-4.907	1.00	0.00	N
ATOM	101	HH21	ARG	7	23.431	-3.491	-5.549	1.00	0.00	H
ATOM	102	HH22	ARG	7	21.828	-4.005	-5.349	1.00	0.00	H
ATOM	103	C	ARG	7	21.527	1.855	-1.462	1.00	0.00	C
ATOM	104	O	ARG	7	20.510	1.516	-0.778	1.00	0.00	O
ATOM	105	N	TYR	8	22.203	2.929	-1.190	1.00	0.00	N
ATOM	106	H	TYR	8	22.938	3.212	-1.821	1.00	0.00	H
ATOM	107	CA	TYR	8	21.856	3.957	-0.119	1.00	0.00	C

ATOM	108	HA	TYR	8	20.996	3.583	0.436	1.00	0.00	H
ATOM	109	CB	TYR	8	23.023	4.124	0.852	1.00	0.00	C
ATOM	110	HB2	TYR	8	22.683	4.618	1.761	1.00	0.00	H
ATOM	111	HB3	TYR	8	23.803	4.722	0.380	1.00	0.00	H
ATOM	112	CG	TYR	8	23.410	2.814	1.412	1.00	0.00	C
ATOM	113	CD1	TYR	8	24.589	2.152	0.972	1.00	0.00	C
ATOM	114	HD1	TYR	8	25.235	2.593	0.229	1.00	0.00	H
ATOM	115	CE1	TYR	8	24.873	0.779	1.320	1.00	0.00	C
ATOM	116	HE1	TYR	8	25.790	0.307	0.999	1.00	0.00	H
ATOM	117	CZ	TYR	8	23.926	0.107	2.105	1.00	0.00	C
ATOM	118	OH	TYR	8	24.213	-1.169	2.424	1.00	0.00	O
ATOM	119	HH	TYR	8	24.916	-1.445	1.831	1.00	0.00	H
ATOM	120	CE2	TYR	8	22.714	0.691	2.480	1.00	0.00	C
ATOM	121	HE2	TYR	8	22.065	0.064	3.073	1.00	0.00	H
ATOM	122	CD2	TYR	8	22.471	2.064	2.160	1.00	0.00	C
ATOM	123	HD2	TYR	8	21.534	2.540	2.414	1.00	0.00	H
ATOM	124	C	TYR	8	21.476	5.294	-0.807	1.00	0.00	C
ATOM	125	O	TYR	8	21.563	6.335	-0.123	1.00	0.00	O
ATOM	126	N	TYR	9	21.225	5.301	-2.074	1.00	0.00	N
ATOM	127	H	TYR	9	21.028	4.416	-2.520	1.00	0.00	H
ATOM	128	CA	TYR	9	21.019	6.511	-2.940	1.00	0.00	C
ATOM	129	HA	TYR	9	20.914	6.118	-3.950	1.00	0.00	H
ATOM	130	CB	TYR	9	19.648	7.089	-2.431	1.00	0.00	C
ATOM	131	HB2	TYR	9	19.322	7.699	-3.273	1.00	0.00	H
ATOM	132	HB3	TYR	9	19.948	7.736	-1.608	1.00	0.00	H
ATOM	133	CG	TYR	9	18.608	6.091	-2.079	1.00	0.00	C
ATOM	134	CD1	TYR	9	17.869	6.127	-0.831	1.00	0.00	C
ATOM	135	HD1	TYR	9	18.121	6.913	-0.134	1.00	0.00	H
ATOM	136	CE1	TYR	9	16.936	5.090	-0.515	1.00	0.00	C
ATOM	137	HE1	TYR	9	16.482	5.010	0.462	1.00	0.00	H
ATOM	138	CZ	TYR	9	16.679	4.127	-1.508	1.00	0.00	C
ATOM	139	OH	TYR	9	15.801	3.169	-1.223	1.00	0.00	O
ATOM	140	HH	TYR	9	15.626	2.598	-1.975	1.00	0.00	H
ATOM	141	CE2	TYR	9	17.290	4.162	-2.779	1.00	0.00	C
ATOM	142	HE2	TYR	9	16.912	3.425	-3.473	1.00	0.00	H
ATOM	143	CD2	TYR	9	18.281	5.130	-3.076	1.00	0.00	C
ATOM	144	HD2	TYR	9	18.898	5.074	-3.960	1.00	0.00	H
ATOM	145	C	TYR	9	22.147	7.583	-3.064	1.00	0.00	C
ATOM	146	O	TYR	9	21.971	8.654	-3.589	1.00	0.00	O
ATOM	147	N	ARG	10	23.347	7.221	-2.656	1.00	0.00	N
ATOM	148	H	ARG	10	23.410	6.338	-2.169	1.00	0.00	H
ATOM	149	CA	ARG	10	24.595	8.025	-2.858	1.00	0.00	C
ATOM	150	HA	ARG	10	24.256	9.056	-2.757	1.00	0.00	H
ATOM	151	CB	ARG	10	25.571	7.817	-1.695	1.00	0.00	C
ATOM	152	HB2	ARG	10	26.491	8.313	-2.005	1.00	0.00	H
ATOM	153	HB3	ARG	10	25.654	6.734	-1.609	1.00	0.00	H
ATOM	154	CG	ARG	10	25.233	8.288	-0.247	1.00	0.00	C
ATOM	155	HG2	ARG	10	24.371	7.714	0.090	1.00	0.00	H
ATOM	156	HG3	ARG	10	24.807	9.287	-0.353	1.00	0.00	H
ATOM	157	CD	ARG	10	26.393	8.299	0.787	1.00	0.00	C
ATOM	158	HD2	ARG	10	25.972	8.778	1.672	1.00	0.00	H
ATOM	159	HD3	ARG	10	27.103	8.912	0.232	1.00	0.00	H
ATOM	160	NE	ARG	10	27.002	6.962	0.905	1.00	0.00	N
ATOM	161	HE	ARG	10	27.946	6.838	0.571	1.00	0.00	H
ATOM	162	CZ	ARG	10	26.624	5.919	1.642	1.00	0.00	C
ATOM	163	NH1	ARG	10	25.534	5.887	2.297	1.00	0.00	N
ATOM	164	HH11	ARG	10	25.456	5.188	3.022	1.00	0.00	H
ATOM	165	HH12	ARG	10	24.907	6.678	2.259	1.00	0.00	H
ATOM	166	NH2	ARG	10	27.277	4.778	1.702	1.00	0.00	N
ATOM	167	HH21	ARG	10	26.977	4.078	2.366	1.00	0.00	H
ATOM	168	HH22	ARG	10	28.135	4.743	1.171	1.00	0.00	H
ATOM	169	C	ARG	10	25.221	7.916	-4.276	1.00	0.00	C
ATOM	170	O	ARG	10	25.479	6.836	-4.762	1.00	0.00	O

ATOM	171	N	GLU	11	25.516	9.036	-5.013	1.00	0.00	N
ATOM	172	H	GLU	11	25.212	9.851	-4.499	1.00	0.00	H
ATOM	173	CA	GLU	11	25.970	9.225	-6.389	1.00	0.00	C
ATOM	174	HA	GLU	11	25.804	8.406	-7.090	1.00	0.00	H
ATOM	175	CB	GLU	11	25.234	10.481	-7.046	1.00	0.00	C
ATOM	176	HB2	GLU	11	25.851	10.897	-7.842	1.00	0.00	H
ATOM	177	HB3	GLU	11	25.028	11.209	-6.262	1.00	0.00	H
ATOM	178	CG	GLU	11	23.784	10.109	-7.479	1.00	0.00	C
ATOM	179	HG2	GLU	11	23.204	9.585	-6.719	1.00	0.00	H
ATOM	180	HG3	GLU	11	24.021	9.463	-8.324	1.00	0.00	H
ATOM	181	CD	GLU	11	22.920	11.324	-8.030	1.00	0.00	C
ATOM	182	OE1	GLU	11	21.640	11.251	-7.957	1.00	0.00	O
ATOM	183	OE2	GLU	11	23.475	12.352	-8.485	1.00	0.00	O
ATOM	184	C	GLU	11	27.487	9.372	-6.527	1.00	0.00	C
ATOM	185	O	GLU	11	28.007	9.327	-7.625	1.00	0.00	O
ATOM	186	N	ASN	12	28.217	9.402	-5.421	1.00	0.00	N
ATOM	187	H	ASN	12	27.735	9.515	-4.541	1.00	0.00	H
ATOM	188	CA	ASN	12	29.699	9.382	-5.434	1.00	0.00	C
ATOM	189	HA	ASN	12	29.931	10.049	-6.264	1.00	0.00	H
ATOM	190	CB	ASN	12	30.344	10.009	-4.130	1.00	0.00	C
ATOM	191	HB2	ASN	12	29.941	11.017	-4.032	1.00	0.00	H
ATOM	192	HB3	ASN	12	31.432	9.938	-4.141	1.00	0.00	H
ATOM	193	CG	ASN	12	29.679	9.478	-2.874	1.00	0.00	C
ATOM	194	OD1	ASN	12	28.522	9.876	-2.633	1.00	0.00	O
ATOM	195	ND2	ASN	12	30.157	8.552	-2.102	1.00	0.00	N
ATOM	196	HD21	ASN	12	29.499	7.985	-1.585	1.00	0.00	H
ATOM	197	HD22	ASN	12	31.072	8.183	-2.317	1.00	0.00	H
ATOM	198	C	ASN	12	30.372	8.019	-5.741	1.00	0.00	C
ATOM	199	O	ASN	12	30.002	6.893	-5.305	1.00	0.00	O
ATOM	200	N	MET	13	31.524	8.129	-6.470	1.00	0.00	N
ATOM	201	H	MET	13	31.825	9.052	-6.749	1.00	0.00	H
ATOM	202	CA	MET	13	32.209	6.903	-6.920	1.00	0.00	C
ATOM	203	HA	MET	13	31.515	6.161	-7.315	1.00	0.00	H
ATOM	204	CB	MET	13	33.157	7.242	-8.082	1.00	0.00	C
ATOM	205	HB2	MET	13	33.898	6.475	-8.311	1.00	0.00	H
ATOM	206	HB3	MET	13	33.599	8.147	-7.665	1.00	0.00	H
ATOM	207	CG	MET	13	32.455	7.567	-9.397	1.00	0.00	C
ATOM	208	HG2	MET	13	31.793	8.405	-9.172	1.00	0.00	H
ATOM	209	HG3	MET	13	31.894	6.650	-9.584	1.00	0.00	H
ATOM	210	SD	MET	13	33.334	7.817	-10.905	1.00	0.00	S
ATOM	211	CE	MET	13	32.000	8.471	-12.031	1.00	0.00	C
ATOM	212	HE1	MET	13	32.333	8.369	-13.065	1.00	0.00	H
ATOM	213	HE2	MET	13	31.136	7.832	-11.851	1.00	0.00	H
ATOM	214	HE3	MET	13	31.878	9.524	-11.777	1.00	0.00	H
ATOM	215	C	MET	13	32.957	6.210	-5.774	1.00	0.00	C
ATOM	216	O	MET	13	33.058	4.955	-5.847	1.00	0.00	O
ATOM	217	N	TYR	14	33.469	6.988	-4.814	1.00	0.00	N
ATOM	218	H	TYR	14	33.297	7.983	-4.842	1.00	0.00	H
ATOM	219	CA	TYR	14	34.105	6.283	-3.631	1.00	0.00	C
ATOM	220	HA	TYR	14	34.554	5.409	-4.101	1.00	0.00	H
ATOM	221	CB	TYR	14	35.368	7.151	-3.132	1.00	0.00	C
ATOM	222	HB2	TYR	14	35.112	7.903	-2.386	1.00	0.00	H
ATOM	223	HB3	TYR	14	35.727	7.742	-3.974	1.00	0.00	H
ATOM	224	CG	TYR	14	36.511	6.308	-2.596	1.00	0.00	C
ATOM	225	CD1	TYR	14	36.703	6.026	-1.211	1.00	0.00	C
ATOM	226	HD1	TYR	14	35.975	6.314	-0.468	1.00	0.00	H
ATOM	227	CE1	TYR	14	37.901	5.325	-0.947	1.00	0.00	C
ATOM	228	HE1	TYR	14	38.079	5.144	0.103	1.00	0.00	H
ATOM	229	CZ	TYR	14	38.846	4.844	-1.910	1.00	0.00	C
ATOM	230	OH	TYR	14	39.901	4.149	-1.467	1.00	0.00	O
ATOM	231	HH	TYR	14	39.915	3.901	-0.540	1.00	0.00	H
ATOM	232	CE2	TYR	14	38.597	5.067	-3.248	1.00	0.00	C
ATOM	233	HE2	TYR	14	39.208	4.539	-3.964	1.00	0.00	H

ATOM	234	CD2	TYR	14	37.431	5.842	-3.552	1.00	0.00	C
ATOM	235	HD2	TYR	14	37.200	6.034	-4.590	1.00	0.00	H
ATOM	236	C	TYR	14	33.202	5.894	-2.485	1.00	0.00	C
ATOM	237	O	TYR	14	32.227	6.564	-2.193	1.00	0.00	O
ATOM	238	N	ARG	15	33.514	4.829	-1.740	1.00	0.00	N
ATOM	239	H	ARG	15	34.212	4.148	-2.003	1.00	0.00	H
ATOM	240	CA	ARG	15	32.872	4.450	-0.444	1.00	0.00	C
ATOM	241	HA	ARG	15	31.835	4.163	-0.616	1.00	0.00	H
ATOM	242	CB	ARG	15	33.501	3.165	0.126	1.00	0.00	C
ATOM	243	HB2	ARG	15	34.235	3.398	0.896	1.00	0.00	H
ATOM	244	HB3	ARG	15	34.121	2.703	-0.642	1.00	0.00	H
ATOM	245	CG	ARG	15	32.581	2.211	0.828	1.00	0.00	C
ATOM	246	HG2	ARG	15	32.085	2.790	1.608	1.00	0.00	H
ATOM	247	HG3	ARG	15	33.241	1.425	1.193	1.00	0.00	H
ATOM	248	CD	ARG	15	31.462	1.500	0.025	1.00	0.00	C
ATOM	249	HD2	ARG	15	31.837	0.700	-0.613	1.00	0.00	H
ATOM	250	HD3	ARG	15	30.894	2.256	-0.517	1.00	0.00	H
ATOM	251	NE	ARG	15	30.656	0.742	0.945	1.00	0.00	N
ATOM	252	HE	ARG	15	31.030	-0.160	1.203	1.00	0.00	H
ATOM	253	CZ	ARG	15	29.720	1.188	1.738	1.00	0.00	C
ATOM	254	NH1	ARG	15	29.157	2.379	1.580	1.00	0.00	N
ATOM	255	HH11	ARG	15	29.199	2.922	0.729	1.00	0.00	H
ATOM	256	HH12	ARG	15	28.524	2.635	2.324	1.00	0.00	H
ATOM	257	NH2	ARG	15	29.265	0.557	2.783	1.00	0.00	N
ATOM	258	HH21	ARG	15	29.537	-0.402	2.943	1.00	0.00	H
ATOM	259	HH22	ARG	15	28.588	1.015	3.376	1.00	0.00	H
ATOM	260	C	ARG	15	32.908	5.673	0.555	1.00	0.00	C
ATOM	261	O	ARG	15	34.002	6.124	0.940	1.00	0.00	O
ATOM	262	N	TYR	16	31.790	6.110	1.165	1.00	0.00	N
ATOM	263	H	TYR	16	31.008	5.559	0.845	1.00	0.00	H
ATOM	264	CA	TYR	16	31.662	7.258	2.127	1.00	0.00	C
ATOM	265	HA	TYR	16	30.619	7.394	2.415	1.00	0.00	H
ATOM	266	CB	TYR	16	32.353	6.987	3.441	1.00	0.00	C
ATOM	267	HB2	TYR	16	32.061	7.763	4.148	1.00	0.00	H
ATOM	268	HB3	TYR	16	33.396	7.157	3.173	1.00	0.00	H
ATOM	269	CG	TYR	16	32.105	5.657	4.115	1.00	0.00	C
ATOM	270	CD1	TYR	16	33.179	4.795	4.334	1.00	0.00	C
ATOM	271	HD1	TYR	16	34.181	5.190	4.255	1.00	0.00	H
ATOM	272	CE1	TYR	16	32.965	3.517	4.780	1.00	0.00	C
ATOM	273	HE1	TYR	16	33.743	2.777	4.897	1.00	0.00	H
ATOM	274	CZ	TYR	16	31.647	3.054	4.868	1.00	0.00	C
ATOM	275	OH	TYR	16	31.456	1.713	5.131	1.00	0.00	O
ATOM	276	HH	TYR	16	30.547	1.512	5.367	1.00	0.00	H
ATOM	277	CE2	TYR	16	30.545	3.963	4.675	1.00	0.00	C
ATOM	278	HE2	TYR	16	29.535	3.580	4.651	1.00	0.00	H
ATOM	279	CD2	TYR	16	30.769	5.277	4.327	1.00	0.00	C
ATOM	280	HD2	TYR	16	29.896	5.897	4.180	1.00	0.00	H
ATOM	281	C	TYR	16	32.092	8.593	1.479	1.00	0.00	C
ATOM	282	O	TYR	16	31.941	9.638	2.147	1.00	0.00	O
ATOM	283	N	NME	17	32.547	8.595	0.224	1.00	0.00	N
ATOM	284	H	NME	17	32.560	7.699	-0.242	1.00	0.00	H
ATOM	285	CH3	NME	17	33.115	9.723	-0.594	1.00	0.00	C
ATOM	286	HH31	NME	17	33.411	10.572	0.022	1.00	0.00	H
ATOM	287	HH32	NME	17	33.895	9.357	-1.263	1.00	0.00	H
ATOM	288	HH33	NME	17	32.304	10.074	-1.231	1.00	0.00	H
TER	289		NME	17						
ATOM	289	HH31	ACE	18	26.685	7.283	11.362	1.00	0.00	H
ATOM	290	CH3	ACE	18	27.298	7.967	10.773	1.00	0.00	C
ATOM	291	HH32	ACE	18	27.271	7.785	9.698	1.00	0.00	H
ATOM	292	HH33	ACE	18	28.350	7.816	11.011	1.00	0.00	H
ATOM	293	C	ACE	18	26.912	9.406	11.074	1.00	0.00	C
ATOM	294	O	ACE	18	26.248	9.663	12.101	1.00	0.00	O
ATOM	295	N	LYS	19	27.374	10.393	10.268	1.00	0.00	N

ATOM	296	H	LYS	19	28.007	10.029	9.570	1.00	0.00	H
ATOM	297	CA	LYS	19	27.319	11.853	10.493	1.00	0.00	C
ATOM	298	HA	LYS	19	27.187	12.095	11.548	1.00	0.00	H
ATOM	299	CB	LYS	19	28.614	12.535	9.925	1.00	0.00	C
ATOM	300	HB2	LYS	19	29.523	12.051	10.282	1.00	0.00	H
ATOM	301	HB3	LYS	19	28.490	13.508	10.399	1.00	0.00	H
ATOM	302	CG	LYS	19	28.748	12.587	8.399	1.00	0.00	C
ATOM	303	HG2	LYS	19	27.779	12.717	7.918	1.00	0.00	H
ATOM	304	HG3	LYS	19	29.114	11.605	8.102	1.00	0.00	H
ATOM	305	CD	LYS	19	29.651	13.651	7.858	1.00	0.00	C
ATOM	306	HD2	LYS	19	30.093	13.230	6.956	1.00	0.00	H
ATOM	307	HD3	LYS	19	30.386	13.679	8.663	1.00	0.00	H
ATOM	308	CE	LYS	19	29.266	15.062	7.514	1.00	0.00	C
ATOM	309	HE2	LYS	19	28.624	15.489	8.283	1.00	0.00	H
ATOM	310	HE3	LYS	19	28.705	14.921	6.590	1.00	0.00	H
ATOM	311	NZ	LYS	19	30.350	16.037	7.135	1.00	0.00	N
ATOM	312	HZ1	LYS	19	29.855	16.872	6.856	1.00	0.00	H
ATOM	313	HZ2	LYS	19	30.947	16.312	7.902	1.00	0.00	H
ATOM	314	HZ3	LYS	19	30.958	15.730	6.389	1.00	0.00	H
ATOM	315	C	LYS	19	25.974	12.400	9.961	1.00	0.00	C
ATOM	316	O	LYS	19	25.854	13.599	9.741	1.00	0.00	O
ATOM	317	N	GLY	20	24.919	11.601	9.894	1.00	0.00	N
ATOM	318	H	GLY	20	25.050	10.599	9.903	1.00	0.00	H
ATOM	319	CA	GLY	20	23.512	12.024	9.510	1.00	0.00	C
ATOM	320	HA2	GLY	20	23.130	12.745	10.234	1.00	0.00	H
ATOM	321	HA3	GLY	20	23.755	12.534	8.578	1.00	0.00	H
ATOM	322	C	GLY	20	22.486	10.963	9.183	1.00	0.00	C
ATOM	323	O	GLY	20	22.635	9.763	9.390	1.00	0.00	O
ATOM	324	N	GLU	21	21.556	11.521	8.386	1.00	0.00	N
ATOM	325	H	GLU	21	21.639	12.518	8.247	1.00	0.00	H
ATOM	326	CA	GLU	21	20.262	10.934	8.001	1.00	0.00	C
ATOM	327	HA	GLU	21	19.765	10.642	8.926	1.00	0.00	H
ATOM	328	CB	GLU	21	19.356	12.034	7.287	1.00	0.00	C
ATOM	329	HB2	GLU	21	18.971	12.704	8.054	1.00	0.00	H
ATOM	330	HB3	GLU	21	18.557	11.530	6.741	1.00	0.00	H
ATOM	331	CG	GLU	21	20.168	12.909	6.251	1.00	0.00	C
ATOM	332	HG2	GLU	21	20.877	12.285	5.705	1.00	0.00	H
ATOM	333	HG3	GLU	21	20.855	13.576	6.772	1.00	0.00	H
ATOM	334	CD	GLU	21	19.394	13.716	5.246	1.00	0.00	C
ATOM	335	OE1	GLU	21	18.956	14.865	5.516	1.00	0.00	O
ATOM	336	OE2	GLU	21	19.219	13.158	4.140	1.00	0.00	O
ATOM	337	C	GLU	21	20.449	9.682	7.065	1.00	0.00	C
ATOM	338	O	GLU	21	19.576	8.783	7.058	1.00	0.00	O
ATOM	339	N	ASN	22	21.301	9.720	6.095	1.00	0.00	N
ATOM	340	H	ASN	22	21.642	10.660	5.956	1.00	0.00	H
ATOM	341	CA	ASN	22	21.446	8.795	4.971	1.00	0.00	C
ATOM	342	HA	ASN	22	20.458	8.510	4.608	1.00	0.00	H
ATOM	343	CB	ASN	22	22.226	9.490	3.802	1.00	0.00	C
ATOM	344	HB2	ASN	22	23.234	9.821	4.057	1.00	0.00	H
ATOM	345	HB3	ASN	22	21.570	10.354	3.695	1.00	0.00	H
ATOM	346	CG	ASN	22	22.396	8.519	2.550	1.00	0.00	C
ATOM	347	OD1	ASN	22	23.404	7.904	2.425	1.00	0.00	O
ATOM	348	ND2	ASN	22	21.463	8.358	1.684	1.00	0.00	N
ATOM	349	HD21	ASN	22	21.559	7.605	1.018	1.00	0.00	H
ATOM	350	HD22	ASN	22	20.595	8.874	1.719	1.00	0.00	H
ATOM	351	C	ASN	22	22.268	7.514	5.360	1.00	0.00	C
ATOM	352	O	ASN	22	21.820	6.431	5.066	1.00	0.00	O
ATOM	353	N	PHE	23	23.404	7.595	6.035	1.00	0.00	N
ATOM	354	H	PHE	23	23.761	8.525	6.200	1.00	0.00	H
ATOM	355	CA	PHE	23	24.212	6.414	6.464	1.00	0.00	C
ATOM	356	HA	PHE	23	24.553	5.927	5.550	1.00	0.00	H
ATOM	357	CB	PHE	23	25.377	6.914	7.293	1.00	0.00	C
ATOM	358	HB2	PHE	23	26.001	6.039	7.482	1.00	0.00	H

ATOM	359	HB3	PHE	23	24.893	7.322	8.181	1.00	0.00	H
ATOM	360	CG	PHE	23	26.366	7.882	6.628	1.00	0.00	C
ATOM	361	CD1	PHE	23	26.088	9.275	6.724	1.00	0.00	C
ATOM	362	HD1	PHE	23	25.324	9.579	7.423	1.00	0.00	H
ATOM	363	CE1	PHE	23	26.877	10.200	6.023	1.00	0.00	C
ATOM	364	HE1	PHE	23	26.652	11.250	6.147	1.00	0.00	H
ATOM	365	CZ	PHE	23	27.860	9.753	5.105	1.00	0.00	C
ATOM	366	HZ	PHE	23	28.419	10.483	4.538	1.00	0.00	H
ATOM	367	CE2	PHE	23	28.037	8.317	4.922	1.00	0.00	C
ATOM	368	HE2	PHE	23	28.749	7.950	4.197	1.00	0.00	H
ATOM	369	CD2	PHE	23	27.300	7.414	5.714	1.00	0.00	C
ATOM	370	HD2	PHE	23	27.413	6.340	5.700	1.00	0.00	H
ATOM	371	C	PHE	23	23.304	5.478	7.264	1.00	0.00	C
ATOM	372	O	PHE	23	22.493	5.892	8.098	1.00	0.00	O
ATOM	373	N	THR	24	23.482	4.149	6.979	1.00	0.00	N
ATOM	374	H	THR	24	24.267	3.846	6.421	1.00	0.00	H
ATOM	375	CA	THR	24	22.716	3.033	7.535	1.00	0.00	C
ATOM	376	HA	THR	24	21.904	3.367	8.181	1.00	0.00	H
ATOM	377	CB	THR	24	22.080	2.201	6.390	1.00	0.00	C
ATOM	378	HB	THR	24	22.837	1.512	6.013	1.00	0.00	H
ATOM	379	CG2	THR	24	20.996	1.309	6.984	1.00	0.00	C
ATOM	380	HG21	THR	24	21.281	0.527	7.687	1.00	0.00	H
ATOM	381	HG22	THR	24	20.277	1.984	7.446	1.00	0.00	H
ATOM	382	HG23	THR	24	20.539	0.744	6.171	1.00	0.00	H
ATOM	383	OG1	THR	24	21.506	2.871	5.253	1.00	0.00	O
ATOM	384	HG1	THR	24	21.360	3.775	5.539	1.00	0.00	H
ATOM	385	C	THR	24	23.607	2.217	8.427	1.00	0.00	C
ATOM	386	O	THR	24	24.815	2.068	8.123	1.00	0.00	O
ATOM	387	N	GLU	25	23.079	1.612	9.482	1.00	0.00	N
ATOM	388	H	GLU	25	22.143	1.805	9.811	1.00	0.00	H
ATOM	389	CA	GLU	25	23.866	0.661	10.278	1.00	0.00	C
ATOM	390	HA	GLU	25	24.741	1.155	10.702	1.00	0.00	H
ATOM	391	CB	GLU	25	22.947	0.150	11.386	1.00	0.00	C
ATOM	392	HB2	GLU	25	22.469	0.940	11.966	1.00	0.00	H
ATOM	393	HB3	GLU	25	23.655	-0.254	12.110	1.00	0.00	H
ATOM	394	CG	GLU	25	21.877	-0.974	11.090	1.00	0.00	C
ATOM	395	HG2	GLU	25	22.452	-1.900	11.074	1.00	0.00	H
ATOM	396	HG3	GLU	25	21.515	-0.781	10.080	1.00	0.00	H
ATOM	397	CD	GLU	25	20.783	-1.233	12.180	1.00	0.00	C
ATOM	398	OE1	GLU	25	21.264	-1.618	13.272	1.00	0.00	O
ATOM	399	OE2	GLU	25	19.529	-1.098	12.042	1.00	0.00	O
ATOM	400	C	GLU	25	24.586	-0.495	9.568	1.00	0.00	C
ATOM	401	O	GLU	25	25.733	-0.753	9.952	1.00	0.00	O
ATOM	402	N	THR	26	24.124	-1.023	8.458	1.00	0.00	N
ATOM	403	H	THR	26	23.200	-0.776	8.132	1.00	0.00	H
ATOM	404	CA	THR	26	24.843	-1.981	7.538	1.00	0.00	C
ATOM	405	HA	THR	26	25.493	-2.624	8.132	1.00	0.00	H
ATOM	406	CB	THR	26	23.799	-2.859	6.864	1.00	0.00	C
ATOM	407	HB	THR	26	24.235	-3.569	6.161	1.00	0.00	H
ATOM	408	CG2	THR	26	23.038	-3.714	7.931	1.00	0.00	C
ATOM	409	HG21	THR	26	23.743	-4.339	8.477	1.00	0.00	H
ATOM	410	HG22	THR	26	22.513	-3.053	8.621	1.00	0.00	H
ATOM	411	HG23	THR	26	22.292	-4.328	7.427	1.00	0.00	H
ATOM	412	OG1	THR	26	22.857	-1.941	6.274	1.00	0.00	O
ATOM	413	HG1	THR	26	22.162	-2.398	5.795	1.00	0.00	H
ATOM	414	C	THR	26	25.744	-1.302	6.519	1.00	0.00	C
ATOM	415	O	THR	26	26.322	-1.978	5.688	1.00	0.00	O
ATOM	416	N	ASP	27	25.874	0.027	6.511	1.00	0.00	N
ATOM	417	H	ASP	27	25.242	0.499	7.141	1.00	0.00	H
ATOM	418	CA	ASP	27	26.627	0.788	5.540	1.00	0.00	C
ATOM	419	HA	ASP	27	26.951	0.190	4.689	1.00	0.00	H
ATOM	420	CB	ASP	27	25.572	1.863	4.985	1.00	0.00	C
ATOM	421	HB2	ASP	27	24.925	2.302	5.744	1.00	0.00	H

ATOM	422	HB3	ASP	27	24.814	1.276	4.467	1.00	0.00	H
ATOM	423	CG	ASP	27	26.293	2.882	4.167	1.00	0.00	C
ATOM	424	OD1	ASP	27	27.216	2.516	3.441	1.00	0.00	O
ATOM	425	OD2	ASP	27	25.987	4.078	4.143	1.00	0.00	O
ATOM	426	C	ASP	27	27.819	1.457	6.068	1.00	0.00	C
ATOM	427	O	ASP	27	28.905	1.230	5.525	1.00	0.00	O
ATOM	428	N	ILE	28	27.831	1.937	7.318	1.00	0.00	N
ATOM	429	H	ILE	28	27.052	1.764	7.938	1.00	0.00	H
ATOM	430	CA	ILE	28	29.017	2.497	8.111	1.00	0.00	C
ATOM	431	HA	ILE	28	29.837	2.556	7.394	1.00	0.00	H
ATOM	432	CB	ILE	28	28.751	3.944	8.689	1.00	0.00	C
ATOM	433	HB	ILE	28	29.567	4.128	9.389	1.00	0.00	H
ATOM	434	CG2	ILE	28	28.937	5.050	7.698	1.00	0.00	C
ATOM	435	HG21	ILE	28	28.688	6.029	8.106	1.00	0.00	H
ATOM	436	HG22	ILE	28	29.958	5.158	7.330	1.00	0.00	H
ATOM	437	HG23	ILE	28	28.277	4.930	6.839	1.00	0.00	H
ATOM	438	CG1	ILE	28	27.414	4.227	9.507	1.00	0.00	C
ATOM	439	HG12	ILE	28	26.499	4.158	8.918	1.00	0.00	H
ATOM	440	HG13	ILE	28	27.492	5.271	9.810	1.00	0.00	H
ATOM	441	CD1	ILE	28	27.147	3.337	10.747	1.00	0.00	C
ATOM	442	HD11	ILE	28	27.167	2.298	10.418	1.00	0.00	H
ATOM	443	HD12	ILE	28	27.923	3.598	11.466	1.00	0.00	H
ATOM	444	HD13	ILE	28	26.133	3.403	11.144	1.00	0.00	H
ATOM	445	C	ILE	28	29.651	1.454	9.175	1.00	0.00	C
ATOM	446	O	ILE	28	30.356	1.861	10.109	1.00	0.00	O
ATOM	447	N	LYS	29	29.408	0.128	8.976	1.00	0.00	N
ATOM	448	H	LYS	29	28.846	-0.133	8.179	1.00	0.00	H
ATOM	449	CA	LYS	29	29.850	-0.985	9.891	1.00	0.00	C
ATOM	450	HA	LYS	29	29.658	-0.794	10.947	1.00	0.00	H
ATOM	451	CB	LYS	29	29.117	-2.339	9.520	1.00	0.00	C
ATOM	452	HB2	LYS	29	28.067	-2.096	9.362	1.00	0.00	H
ATOM	453	HB3	LYS	29	29.209	-3.031	10.357	1.00	0.00	H
ATOM	454	CG	LYS	29	29.494	-2.961	8.186	1.00	0.00	C
ATOM	455	HG2	LYS	29	30.566	-3.129	8.092	1.00	0.00	H
ATOM	456	HG3	LYS	29	29.091	-2.303	7.415	1.00	0.00	H
ATOM	457	CD	LYS	29	28.826	-4.309	7.854	1.00	0.00	C
ATOM	458	HD2	LYS	29	27.748	-4.167	7.917	1.00	0.00	H
ATOM	459	HD3	LYS	29	29.131	-4.983	8.655	1.00	0.00	H
ATOM	460	CE	LYS	29	29.179	-4.998	6.483	1.00	0.00	C
ATOM	461	HE2	LYS	29	28.755	-6.002	6.505	1.00	0.00	H
ATOM	462	HE3	LYS	29	30.255	-5.167	6.411	1.00	0.00	H
ATOM	463	NZ	LYS	29	28.580	-4.183	5.291	1.00	0.00	N
ATOM	464	HZ1	LYS	29	27.579	-4.083	5.384	1.00	0.00	H
ATOM	465	HZ2	LYS	29	29.044	-3.287	5.249	1.00	0.00	H
ATOM	466	HZ3	LYS	29	28.622	-4.678	4.413	1.00	0.00	H
ATOM	467	C	LYS	29	31.330	-1.150	9.876	1.00	0.00	C
ATOM	468	O	LYS	29	31.969	-0.780	8.889	1.00	0.00	O
ATOM	469	N	ILE	30	31.946	-1.620	10.917	1.00	0.00	N
ATOM	470	H	ILE	30	31.359	-2.076	11.600	1.00	0.00	H
ATOM	471	CA	ILE	30	33.401	-1.562	11.163	1.00	0.00	C
ATOM	472	HA	ILE	30	33.735	-0.589	10.802	1.00	0.00	H
ATOM	473	CB	ILE	30	33.703	-1.556	12.713	1.00	0.00	C
ATOM	474	HB	ILE	30	34.786	-1.461	12.784	1.00	0.00	H
ATOM	475	CG2	ILE	30	32.981	-0.352	13.365	1.00	0.00	C
ATOM	476	HG21	ILE	30	32.065	-0.601	13.901	1.00	0.00	H
ATOM	477	HG22	ILE	30	33.598	-0.048	14.213	1.00	0.00	H
ATOM	478	HG23	ILE	30	32.819	0.394	12.588	1.00	0.00	H
ATOM	479	CG1	ILE	30	33.263	-2.913	13.242	1.00	0.00	C
ATOM	480	HG12	ILE	30	33.933	-3.659	12.816	1.00	0.00	H
ATOM	481	HG13	ILE	30	32.212	-3.067	12.998	1.00	0.00	H
ATOM	482	CD1	ILE	30	33.356	-3.077	14.735	1.00	0.00	C
ATOM	483	HD11	ILE	30	34.327	-2.688	15.041	1.00	0.00	H
ATOM	484	HD12	ILE	30	32.566	-2.708	15.389	1.00	0.00	H

ATOM	485	HD13	ILE	30	33.342	-4.145	14.950	1.00	0.00	H
ATOM	486	C	ILE	30	34.235	-2.552	10.319	1.00	0.00	C
ATOM	487	O	ILE	30	35.455	-2.487	10.286	1.00	0.00	O
ATOM	488	N	MET	31	33.517	-3.524	9.666	1.00	0.00	N
ATOM	489	H	MET	31	32.555	-3.520	9.974	1.00	0.00	H
ATOM	490	CA	MET	31	34.074	-4.580	8.823	1.00	0.00	C
ATOM	491	HA	MET	31	34.862	-5.096	9.371	1.00	0.00	H
ATOM	492	CB	MET	31	33.067	-5.646	8.509	1.00	0.00	C
ATOM	493	HB2	MET	31	33.467	-6.516	7.990	1.00	0.00	H
ATOM	494	HB3	MET	31	32.276	-5.219	7.892	1.00	0.00	H
ATOM	495	CG	MET	31	32.469	-6.232	9.834	1.00	0.00	C
ATOM	496	HG2	MET	31	33.219	-6.263	10.624	1.00	0.00	H
ATOM	497	HG3	MET	31	32.168	-7.230	9.513	1.00	0.00	H
ATOM	498	SD	MET	31	31.047	-5.496	10.686	1.00	0.00	S
ATOM	499	CE	MET	31	30.774	-6.842	11.889	1.00	0.00	C
ATOM	500	HE1	MET	31	30.130	-6.519	12.706	1.00	0.00	H
ATOM	501	HE2	MET	31	30.310	-7.700	11.404	1.00	0.00	H
ATOM	502	HE3	MET	31	31.677	-7.096	12.444	1.00	0.00	H
ATOM	503	C	MET	31	34.632	-4.043	7.511	1.00	0.00	C
ATOM	504	O	MET	31	35.625	-4.604	7.035	1.00	0.00	O
ATOM	505	N	GLU	32	34.023	-3.002	6.940	1.00	0.00	N
ATOM	506	H	GLU	32	33.150	-2.717	7.360	1.00	0.00	H
ATOM	507	CA	GLU	32	34.542	-2.372	5.697	1.00	0.00	C
ATOM	508	HA	GLU	32	35.068	-3.108	5.089	1.00	0.00	H
ATOM	509	CB	GLU	32	33.394	-1.749	4.885	1.00	0.00	C
ATOM	510	HB2	GLU	32	33.823	-1.272	4.003	1.00	0.00	H
ATOM	511	HB3	GLU	32	32.958	-0.994	5.539	1.00	0.00	H
ATOM	512	CG	GLU	32	32.269	-2.690	4.426	1.00	0.00	C
ATOM	513	HG2	GLU	32	31.852	-3.381	5.159	1.00	0.00	H
ATOM	514	HG3	GLU	32	32.853	-3.258	3.702	1.00	0.00	H
ATOM	515	CD	GLU	32	30.981	-1.954	3.892	1.00	0.00	C
ATOM	516	OE1	GLU	32	29.999	-1.757	4.672	1.00	0.00	O
ATOM	517	OE2	GLU	32	30.919	-1.768	2.639	1.00	0.00	O
ATOM	518	C	GLU	32	35.543	-1.204	5.910	1.00	0.00	C
ATOM	519	O	GLU	32	36.555	-1.057	5.173	1.00	0.00	O
ATOM	520	N	ARG	33	35.345	-0.466	7.013	1.00	0.00	N
ATOM	521	H	ARG	33	34.582	-0.658	7.648	1.00	0.00	H
ATOM	522	CA	ARG	33	36.137	0.703	7.523	1.00	0.00	C
ATOM	523	HA	ARG	33	36.184	1.409	6.694	1.00	0.00	H
ATOM	524	CB	ARG	33	35.402	1.320	8.729	1.00	0.00	C
ATOM	525	HB2	ARG	33	36.118	1.982	9.216	1.00	0.00	H
ATOM	526	HB3	ARG	33	35.200	0.425	9.316	1.00	0.00	H
ATOM	527	CG	ARG	33	34.114	2.000	8.293	1.00	0.00	C
ATOM	528	HG2	ARG	33	33.550	1.461	7.531	1.00	0.00	H
ATOM	529	HG3	ARG	33	34.446	2.957	7.893	1.00	0.00	H
ATOM	530	CD	ARG	33	33.222	2.292	9.482	1.00	0.00	C
ATOM	531	HD2	ARG	33	32.813	1.388	9.931	1.00	0.00	H
ATOM	532	HD3	ARG	33	32.511	2.991	9.042	1.00	0.00	H
ATOM	533	NE	ARG	33	33.853	2.958	10.696	1.00	0.00	N
ATOM	534	HE	ARG	33	34.855	2.915	10.816	1.00	0.00	H
ATOM	535	CZ	ARG	33	33.163	3.623	11.648	1.00	0.00	C
ATOM	536	NH1	ARG	33	31.858	3.679	11.700	1.00	0.00	N
ATOM	537	HH11	ARG	33	31.231	3.195	11.075	1.00	0.00	H
ATOM	538	HH12	ARG	33	31.371	4.214	12.405	1.00	0.00	H
ATOM	539	NH2	ARG	33	33.843	4.267	12.637	1.00	0.00	N
ATOM	540	HH21	ARG	33	34.851	4.294	12.580	1.00	0.00	H
ATOM	541	HH22	ARG	33	33.297	4.568	13.431	1.00	0.00	H
ATOM	542	C	ARG	33	37.512	0.147	7.935	1.00	0.00	C
ATOM	543	O	ARG	33	37.605	-0.984	8.296	1.00	0.00	O
ATOM	544	N	NME	34	38.509	1.037	7.797	1.00	0.00	N
ATOM	545	H	NME	34	38.285	1.939	7.402	1.00	0.00	H
ATOM	546	CH3	NME	34	39.938	0.848	8.046	1.00	0.00	C
ATOM	547	HH31	NME	34	40.345	0.169	7.297	1.00	0.00	H

```
ATOM    548 HH32 NME    34      40.426   1.808   7.874  1.00  0.00           H
ATOM    549 HH33 NME    34      40.022   0.473   9.067  1.00  0.00           H
TER     550      NME    34
END
```

Cluster 2, Figure 4B:

```
ATOM      1 HH31 ACE     1      28.250  -9.281  -1.615  1.00  0.00           H
ATOM      2  CH3 ACE     1      28.925  -9.259  -0.761  1.00  0.00           C
ATOM      3 HH32 ACE     1      29.461 -10.208  -0.743  1.00  0.00           H
ATOM      4 HH33 ACE     1      28.314  -9.211   0.141  1.00  0.00           H
ATOM      5  C   ACE     1      29.873  -8.090  -0.730  1.00  0.00           C
ATOM      6  O   ACE     1      30.488  -7.862   0.308  1.00  0.00           O
ATOM      7  N   ASN     2      30.125  -7.331  -1.815  1.00  0.00           N
ATOM      8  H   ASN     2      29.602  -7.408  -2.676  1.00  0.00           H
ATOM      9  CA  ASN     2      31.130  -6.256  -1.873  1.00  0.00           C
ATOM     10  HA  ASN     2      31.859  -6.377  -1.071  1.00  0.00           H
ATOM     11  CB  ASN     2      31.928  -6.201  -3.201  1.00  0.00           C
ATOM     12  HB2 ASN     2      31.409  -5.930  -4.120  1.00  0.00           H
ATOM     13  HB3 ASN     2      32.219  -7.251  -3.152  1.00  0.00           H
ATOM     14  CG  ASN     2      33.168  -5.352  -3.235  1.00  0.00           C
ATOM     15  OD1 ASN     2      33.077  -4.176  -3.177  1.00  0.00           O
ATOM     16  ND2 ASN     2      34.357  -5.846  -3.316  1.00  0.00           N
ATOM     17 HD21 ASN     2      35.075  -5.184  -3.062  1.00  0.00           H
ATOM     18 HD22 ASN     2      34.517  -6.843  -3.276  1.00  0.00           H
ATOM     19  C   ASN     2      30.454  -4.888  -1.600  1.00  0.00           C
ATOM     20  O   ASN     2      29.506  -4.488  -2.264  1.00  0.00           O
ATOM     21  N   ASP     3      30.990  -4.057  -0.645  1.00  0.00           N
ATOM     22  H   ASP     3      31.902  -4.384  -0.359  1.00  0.00           H
ATOM     23  CA  ASP     3      30.331  -2.894  -0.077  1.00  0.00           C
ATOM     24  HA  ASP     3      29.327  -3.183   0.236  1.00  0.00           H
ATOM     25  CB  ASP     3      31.115  -2.321   1.057  1.00  0.00           C
ATOM     26  HB2 ASP     3      32.191  -2.194   0.940  1.00  0.00           H
ATOM     27  HB3 ASP     3      31.043  -3.158   1.751  1.00  0.00           H
ATOM     28  CG  ASP     3      30.495  -1.190   1.875  1.00  0.00           C
ATOM     29  OD1 ASP     3      31.106  -0.094   1.926  1.00  0.00           O
ATOM     30  OD2 ASP     3      29.368  -1.267   2.319  1.00  0.00           O
ATOM     31  C   ASP     3      30.328  -1.748  -1.067  1.00  0.00           C
ATOM     32  O   ASP     3      29.445  -0.915  -1.035  1.00  0.00           O
ATOM     33  N   TYR     4      31.164  -1.664  -2.125  1.00  0.00           N
ATOM     34  H   TYR     4      31.908  -2.346  -2.133  1.00  0.00           H
ATOM     35  CA  TYR     4      31.102  -0.686  -3.175  1.00  0.00           C
ATOM     36  HA  TYR     4      30.947   0.190  -2.543  1.00  0.00           H
ATOM     37  CB  TYR     4      32.447  -0.472  -3.782  1.00  0.00           C
ATOM     38  HB2 TYR     4      32.386   0.267  -4.581  1.00  0.00           H
ATOM     39  HB3 TYR     4      32.670  -1.464  -4.176  1.00  0.00           H
ATOM     40  CG  TYR     4      33.524  -0.090  -2.805  1.00  0.00           C
ATOM     41  CD1 TYR     4      33.673   1.299  -2.455  1.00  0.00           C
ATOM     42  HD1 TYR     4      32.936   1.999  -2.819  1.00  0.00           H
ATOM     43  CE1 TYR     4      34.694   1.701  -1.591  1.00  0.00           C
ATOM     44  HE1 TYR     4      34.873   2.726  -1.298  1.00  0.00           H
ATOM     45  CZ  TYR     4      35.587   0.758  -1.020  1.00  0.00           C
ATOM     46  OH  TYR     4      36.554   1.224  -0.198  1.00  0.00           O
ATOM     47  HH  TYR     4      37.063   0.527   0.223  1.00  0.00           H
ATOM     48  CE2 TYR     4      35.553  -0.577  -1.452  1.00  0.00           C
ATOM     49  HE2 TYR     4      36.253  -1.314  -1.088  1.00  0.00           H
ATOM     50  CD2 TYR     4      34.445  -1.001  -2.300  1.00  0.00           C
ATOM     51  HD2 TYR     4      34.385  -2.031  -2.619  1.00  0.00           H
ATOM     52  C   TYR     4      30.001  -0.855  -4.288  1.00  0.00           C
ATOM     53  O   TYR     4      30.114  -0.087  -5.238  1.00  0.00           O
ATOM     54  N   GLU     5      29.185  -1.918  -4.300  1.00  0.00           N
```

ATOM	55	H	GLU	5	29.133	-2.450	-3.443	1.00	0.00	H
ATOM	56	CA	GLU	5	28.219	-2.138	-5.374	1.00	0.00	C
ATOM	57	HA	GLU	5	28.722	-2.008	-6.333	1.00	0.00	H
ATOM	58	CB	GLU	5	27.589	-3.571	-5.305	1.00	0.00	C
ATOM	59	HB2	GLU	5	26.685	-3.849	-5.846	1.00	0.00	H
ATOM	60	HB3	GLU	5	27.284	-3.742	-4.272	1.00	0.00	H
ATOM	61	CG	GLU	5	28.645	-4.674	-5.606	1.00	0.00	C
ATOM	62	HG2	GLU	5	29.512	-4.516	-4.964	1.00	0.00	H
ATOM	63	HG3	GLU	5	28.931	-4.578	-6.654	1.00	0.00	H
ATOM	64	CD	GLU	5	28.158	-6.141	-5.305	1.00	0.00	C
ATOM	65	OE1	GLU	5	27.003	-6.298	-4.879	1.00	0.00	O
ATOM	66	OE2	GLU	5	28.861	-7.126	-5.614	1.00	0.00	O
ATOM	67	C	GLU	5	27.092	-1.193	-5.386	1.00	0.00	C
ATOM	68	O	GLU	5	26.807	-0.644	-4.350	1.00	0.00	O
ATOM	69	N	ASP	6	26.438	-0.994	-6.595	1.00	0.00	N
ATOM	70	H	ASP	6	26.658	-1.562	-7.399	1.00	0.00	H
ATOM	71	CA	ASP	6	25.467	0.059	-6.713	1.00	0.00	C
ATOM	72	HA	ASP	6	26.031	0.945	-6.424	1.00	0.00	H
ATOM	73	CB	ASP	6	24.969	0.250	-8.142	1.00	0.00	C
ATOM	74	HB2	ASP	6	25.740	0.060	-8.890	1.00	0.00	H
ATOM	75	HB3	ASP	6	24.661	1.296	-8.146	1.00	0.00	H
ATOM	76	CG	ASP	6	23.693	-0.549	-8.644	1.00	0.00	C
ATOM	77	OD1	ASP	6	22.596	0.032	-8.836	1.00	0.00	O
ATOM	78	OD2	ASP	6	23.969	-1.771	-8.950	1.00	0.00	O
ATOM	79	C	ASP	6	24.194	-0.124	-5.823	1.00	0.00	C
ATOM	80	O	ASP	6	23.745	0.871	-5.322	1.00	0.00	O
ATOM	81	N	ARG	7	23.868	-1.388	-5.550	1.00	0.00	N
ATOM	82	H	ARG	7	24.339	-2.133	-6.042	1.00	0.00	H
ATOM	83	CA	ARG	7	22.805	-1.764	-4.545	1.00	0.00	C
ATOM	84	HA	ARG	7	21.937	-1.127	-4.713	1.00	0.00	H
ATOM	85	CB	ARG	7	22.295	-3.236	-4.856	1.00	0.00	C
ATOM	86	HB2	ARG	7	22.007	-3.395	-5.895	1.00	0.00	H
ATOM	87	HB3	ARG	7	21.405	-3.336	-4.236	1.00	0.00	H
ATOM	88	CG	ARG	7	23.327	-4.279	-4.435	1.00	0.00	C
ATOM	89	HG2	ARG	7	23.902	-4.184	-3.514	1.00	0.00	H
ATOM	90	HG3	ARG	7	24.017	-4.081	-5.256	1.00	0.00	H
ATOM	91	CD	ARG	7	22.904	-5.731	-4.424	1.00	0.00	C
ATOM	92	HD2	ARG	7	22.337	-6.038	-5.303	1.00	0.00	H
ATOM	93	HD3	ARG	7	22.292	-5.948	-3.548	1.00	0.00	H
ATOM	94	NE	ARG	7	24.102	-6.619	-4.415	1.00	0.00	N
ATOM	95	HE	ARG	7	24.973	-6.116	-4.322	1.00	0.00	H
ATOM	96	CZ	ARG	7	24.142	-7.868	-4.028	1.00	0.00	C
ATOM	97	NH1	ARG	7	23.078	-8.524	-3.758	1.00	0.00	N
ATOM	98	HH11	ARG	7	22.271	-8.117	-4.209	1.00	0.00	H
ATOM	99	HH12	ARG	7	23.063	-9.443	-3.342	1.00	0.00	H
ATOM	100	NH2	ARG	7	25.358	-8.315	-3.910	1.00	0.00	N
ATOM	101	HH21	ARG	7	26.130	-7.780	-4.283	1.00	0.00	H
ATOM	102	HH22	ARG	7	25.546	-9.299	-3.789	1.00	0.00	H
ATOM	103	C	ARG	7	23.088	-1.397	-3.115	1.00	0.00	C
ATOM	104	O	ARG	7	22.181	-1.557	-2.273	1.00	0.00	O
ATOM	105	N	TYR	8	24.298	-0.932	-2.713	1.00	0.00	N
ATOM	106	H	TYR	8	24.937	-0.650	-3.441	1.00	0.00	H
ATOM	107	CA	TYR	8	24.768	-0.559	-1.345	1.00	0.00	C
ATOM	108	HA	TYR	8	23.919	-0.400	-0.681	1.00	0.00	H
ATOM	109	CB	TYR	8	25.747	-1.583	-0.811	1.00	0.00	C
ATOM	110	HB2	TYR	8	25.956	-1.195	0.187	1.00	0.00	H
ATOM	111	HB3	TYR	8	26.600	-1.561	-1.489	1.00	0.00	H
ATOM	112	CG	TYR	8	25.232	-3.004	-0.807	1.00	0.00	C
ATOM	113	CD1	TYR	8	25.875	-4.016	-1.483	1.00	0.00	C
ATOM	114	HD1	TYR	8	26.773	-3.662	-1.968	1.00	0.00	H
ATOM	115	CE1	TYR	8	25.490	-5.369	-1.271	1.00	0.00	C
ATOM	116	HE1	TYR	8	26.050	-6.166	-1.737	1.00	0.00	H
ATOM	117	CZ	TYR	8	24.381	-5.732	-0.446	1.00	0.00	C

ATOM	118	OH	TYR	8	24.159	-7.020	-0.117	1.00	0.00	O
ATOM	119	HH	TYR	8	23.341	-7.203	0.348	1.00	0.00	H
ATOM	120	CE2	TYR	8	23.645	-4.641	0.135	1.00	0.00	C
ATOM	121	HE2	TYR	8	22.913	-4.885	0.892	1.00	0.00	H
ATOM	122	CD2	TYR	8	24.108	-3.300	0.003	1.00	0.00	C
ATOM	123	HD2	TYR	8	23.616	-2.532	0.583	1.00	0.00	H
ATOM	124	C	TYR	8	25.498	0.820	-1.338	1.00	0.00	C
ATOM	125	O	TYR	8	25.367	1.533	-0.390	1.00	0.00	O
ATOM	126	N	TYR	9	26.147	1.243	-2.436	1.00	0.00	N
ATOM	127	H	TYR	9	26.451	0.489	-3.034	1.00	0.00	H
ATOM	128	CA	TYR	9	26.989	2.437	-2.460	1.00	0.00	C
ATOM	129	HA	TYR	9	27.844	2.362	-1.789	1.00	0.00	H
ATOM	130	CB	TYR	9	27.641	2.397	-3.902	1.00	0.00	C
ATOM	131	HB2	TYR	9	26.816	2.478	-4.610	1.00	0.00	H
ATOM	132	HB3	TYR	9	28.075	1.409	-4.054	1.00	0.00	H
ATOM	133	CG	TYR	9	28.715	3.405	-4.275	1.00	0.00	C
ATOM	134	CD1	TYR	9	30.065	3.045	-4.316	1.00	0.00	C
ATOM	135	HD1	TYR	9	30.363	2.028	-4.110	1.00	0.00	H
ATOM	136	CE1	TYR	9	31.153	4.032	-4.447	1.00	0.00	C
ATOM	137	HE1	TYR	9	32.154	3.628	-4.474	1.00	0.00	H
ATOM	138	CZ	TYR	9	30.828	5.414	-4.755	1.00	0.00	C
ATOM	139	OH	TYR	9	31.827	6.301	-5.023	1.00	0.00	O
ATOM	140	HH	TYR	9	31.505	7.206	-5.039	1.00	0.00	H
ATOM	141	CE2	TYR	9	29.472	5.733	-4.887	1.00	0.00	C
ATOM	142	HE2	TYR	9	29.169	6.739	-5.137	1.00	0.00	H
ATOM	143	CD2	TYR	9	28.433	4.805	-4.574	1.00	0.00	C
ATOM	144	HD2	TYR	9	27.412	5.074	-4.800	1.00	0.00	H
ATOM	145	C	TYR	9	26.218	3.676	-2.117	1.00	0.00	C
ATOM	146	O	TYR	9	25.299	4.065	-2.850	1.00	0.00	O
ATOM	147	N	ARG	10	26.519	4.337	-0.997	1.00	0.00	N
ATOM	148	H	ARG	10	27.201	3.880	-0.407	1.00	0.00	H
ATOM	149	CA	ARG	10	26.000	5.623	-0.600	1.00	0.00	C
ATOM	150	HA	ARG	10	25.188	5.837	-1.295	1.00	0.00	H
ATOM	151	CB	ARG	10	25.363	5.526	0.794	1.00	0.00	C
ATOM	152	HB2	ARG	10	24.457	4.937	0.937	1.00	0.00	H
ATOM	153	HB3	ARG	10	24.977	6.507	1.075	1.00	0.00	H
ATOM	154	CG	ARG	10	26.247	5.097	1.975	1.00	0.00	C
ATOM	155	HG2	ARG	10	25.935	5.512	2.933	1.00	0.00	H
ATOM	156	HG3	ARG	10	27.278	5.413	1.814	1.00	0.00	H
ATOM	157	CD	ARG	10	26.265	3.559	2.258	1.00	0.00	C
ATOM	158	HD2	ARG	10	25.485	2.905	1.865	1.00	0.00	H
ATOM	159	HD3	ARG	10	26.257	3.391	3.335	1.00	0.00	H
ATOM	160	NE	ARG	10	27.512	3.025	1.706	1.00	0.00	N
ATOM	161	HE	ARG	10	28.016	3.643	1.087	1.00	0.00	H
ATOM	162	CZ	ARG	10	28.154	1.921	1.886	1.00	0.00	C
ATOM	163	NH1	ARG	10	27.714	0.921	2.463	1.00	0.00	N
ATOM	164	HH11	ARG	10	26.885	0.962	3.037	1.00	0.00	H
ATOM	165	HH12	ARG	10	28.269	0.088	2.320	1.00	0.00	H
ATOM	166	NH2	ARG	10	29.225	1.684	1.222	1.00	0.00	N
ATOM	167	HH21	ARG	10	29.790	2.408	0.799	1.00	0.00	H
ATOM	168	HH22	ARG	10	29.706	0.827	1.456	1.00	0.00	H
ATOM	169	C	ARG	10	27.062	6.790	-0.704	1.00	0.00	C
ATOM	170	O	ARG	10	26.740	7.866	-0.304	1.00	0.00	O
ATOM	171	N	GLU	11	28.258	6.550	-1.240	1.00	0.00	N
ATOM	172	H	GLU	11	28.505	5.575	-1.339	1.00	0.00	H
ATOM	173	CA	GLU	11	29.394	7.490	-1.324	1.00	0.00	C
ATOM	174	HA	GLU	11	29.448	8.159	-0.466	1.00	0.00	H
ATOM	175	CB	GLU	11	30.705	6.627	-1.349	1.00	0.00	C
ATOM	176	HB2	GLU	11	31.562	7.277	-1.524	1.00	0.00	H
ATOM	177	HB3	GLU	11	30.626	6.005	-2.240	1.00	0.00	H
ATOM	178	CG	GLU	11	31.049	5.983	0.003	1.00	0.00	C
ATOM	179	HG2	GLU	11	30.808	6.605	0.864	1.00	0.00	H
ATOM	180	HG3	GLU	11	32.133	5.978	-0.118	1.00	0.00	H

ATOM	181	CD	GLU	11	30.558	4.479	0.231	1.00	0.00	C
ATOM	182	OE1	GLU	11	31.336	3.500	0.370	1.00	0.00	O
ATOM	183	OE2	GLU	11	29.325	4.242	0.112	1.00	0.00	O
ATOM	184	C	GLU	11	29.217	8.506	-2.505	1.00	0.00	C
ATOM	185	O	GLU	11	30.067	9.415	-2.608	1.00	0.00	O
ATOM	186	N	ASN	12	28.138	8.454	-3.327	1.00	0.00	N
ATOM	187	H	ASN	12	27.463	7.723	-3.154	1.00	0.00	H
ATOM	188	CA	ASN	12	27.767	9.454	-4.295	1.00	0.00	C
ATOM	189	HA	ASN	12	28.664	9.642	-4.885	1.00	0.00	H
ATOM	190	CB	ASN	12	26.656	8.773	-5.181	1.00	0.00	C
ATOM	191	HB2	ASN	12	25.711	8.681	-4.648	1.00	0.00	H
ATOM	192	HB3	ASN	12	26.996	7.789	-5.507	1.00	0.00	H
ATOM	193	CG	ASN	12	26.407	9.404	-6.569	1.00	0.00	C
ATOM	194	OD1	ASN	12	27.156	10.233	-7.078	1.00	0.00	O
ATOM	195	ND2	ASN	12	25.380	9.034	-7.248	1.00	0.00	N
ATOM	196	HD21	ASN	12	25.354	9.398	-8.190	1.00	0.00	H
ATOM	197	HD22	ASN	12	24.743	8.314	-6.940	1.00	0.00	H
ATOM	198	C	ASN	12	27.294	10.685	-3.614	1.00	0.00	C
ATOM	199	O	ASN	12	27.222	11.705	-4.284	1.00	0.00	O
ATOM	200	N	MET	13	26.775	10.658	-2.362	1.00	0.00	N
ATOM	201	H	MET	13	26.644	9.742	-1.958	1.00	0.00	H
ATOM	202	CA	MET	13	26.154	11.776	-1.728	1.00	0.00	C
ATOM	203	HA	MET	13	26.459	12.755	-2.097	1.00	0.00	H
ATOM	204	CB	MET	13	24.604	11.672	-1.856	1.00	0.00	C
ATOM	205	HB2	MET	13	24.369	11.788	-2.913	1.00	0.00	H
ATOM	206	HB3	MET	13	24.290	12.557	-1.302	1.00	0.00	H
ATOM	207	CG	MET	13	23.929	10.476	-1.226	1.00	0.00	C
ATOM	208	HG2	MET	13	22.884	10.488	-0.916	1.00	0.00	H
ATOM	209	HG3	MET	13	24.534	10.233	-0.352	1.00	0.00	H
ATOM	210	SD	MET	13	24.014	8.970	-2.308	1.00	0.00	S
ATOM	211	CE	MET	13	22.679	9.565	-3.442	1.00	0.00	C
ATOM	212	HE1	MET	13	21.705	9.733	-2.981	1.00	0.00	H
ATOM	213	HE2	MET	13	22.955	10.384	-4.105	1.00	0.00	H
ATOM	214	HE3	MET	13	22.418	8.768	-4.139	1.00	0.00	H
ATOM	215	C	MET	13	26.456	11.953	-0.163	1.00	0.00	C
ATOM	216	O	MET	13	26.250	13.078	0.328	1.00	0.00	O
ATOM	217	N	TYR	14	26.849	10.883	0.531	1.00	0.00	N
ATOM	218	H	TYR	14	26.824	9.953	0.139	1.00	0.00	H
ATOM	219	CA	TYR	14	27.065	10.897	1.936	1.00	0.00	C
ATOM	220	HA	TYR	14	26.354	11.616	2.345	1.00	0.00	H
ATOM	221	CB	TYR	14	26.645	9.557	2.477	1.00	0.00	C
ATOM	222	HB2	TYR	14	27.348	8.779	2.181	1.00	0.00	H
ATOM	223	HB3	TYR	14	25.682	9.252	2.068	1.00	0.00	H
ATOM	224	CG	TYR	14	26.578	9.682	3.997	1.00	0.00	C
ATOM	225	CD1	TYR	14	27.472	8.935	4.862	1.00	0.00	C
ATOM	226	HD1	TYR	14	28.177	8.223	4.460	1.00	0.00	H
ATOM	227	CE1	TYR	14	27.517	9.147	6.275	1.00	0.00	C
ATOM	228	HE1	TYR	14	28.292	8.716	6.890	1.00	0.00	H
ATOM	229	CZ	TYR	14	26.650	10.140	6.804	1.00	0.00	C
ATOM	230	OH	TYR	14	26.695	10.383	8.149	1.00	0.00	O
ATOM	231	HH	TYR	14	27.457	9.967	8.560	1.00	0.00	H
ATOM	232	CE2	TYR	14	25.835	10.806	5.909	1.00	0.00	C
ATOM	233	HE2	TYR	14	25.214	11.600	6.298	1.00	0.00	H
ATOM	234	CD2	TYR	14	25.676	10.543	4.515	1.00	0.00	C
ATOM	235	HD2	TYR	14	24.980	11.115	3.919	1.00	0.00	H
ATOM	236	C	TYR	14	28.417	11.497	2.452	1.00	0.00	C
ATOM	237	O	TYR	14	28.508	12.702	2.765	1.00	0.00	O
ATOM	238	N	ARG	15	29.414	10.629	2.601	1.00	0.00	N
ATOM	239	H	ARG	15	29.119	9.725	2.260	1.00	0.00	H
ATOM	240	CA	ARG	15	30.751	10.909	3.098	1.00	0.00	C
ATOM	241	HA	ARG	15	31.005	11.957	2.943	1.00	0.00	H
ATOM	242	CB	ARG	15	30.879	10.633	4.573	1.00	0.00	C
ATOM	243	HB2	ARG	15	31.910	10.745	4.907	1.00	0.00	H

ATOM	244	HB3	ARG	15	30.548	9.602	4.695	1.00	0.00	H
ATOM	245	CG	ARG	15	30.006	11.416	5.560	1.00	0.00	C
ATOM	246	HG2	ARG	15	30.052	10.927	6.532	1.00	0.00	H
ATOM	247	HG3	ARG	15	28.951	11.241	5.346	1.00	0.00	H
ATOM	248	CD	ARG	15	30.331	12.904	5.821	1.00	0.00	C
ATOM	249	HD2	ARG	15	30.449	13.401	4.858	1.00	0.00	H
ATOM	250	HD3	ARG	15	31.252	12.843	6.402	1.00	0.00	H
ATOM	251	NE	ARG	15	29.304	13.579	6.668	1.00	0.00	N
ATOM	252	HE	ARG	15	29.559	13.829	7.613	1.00	0.00	H
ATOM	253	CZ	ARG	15	28.092	13.947	6.388	1.00	0.00	C
ATOM	254	NH1	ARG	15	27.626	13.933	5.192	1.00	0.00	N
ATOM	255	HH11	ARG	15	28.191	13.609	4.421	1.00	0.00	H
ATOM	256	HH12	ARG	15	26.685	14.257	5.016	1.00	0.00	H
ATOM	257	NH2	ARG	15	27.396	14.357	7.380	1.00	0.00	N
ATOM	258	HH21	ARG	15	27.655	14.214	8.345	1.00	0.00	H
ATOM	259	HH22	ARG	15	26.414	14.519	7.208	1.00	0.00	H
ATOM	260	C	ARG	15	31.880	10.218	2.297	1.00	0.00	C
ATOM	261	O	ARG	15	31.719	9.144	1.719	1.00	0.00	O
ATOM	262	N	TYR	16	32.907	10.979	2.075	1.00	0.00	N
ATOM	263	H	TYR	16	33.034	11.831	2.603	1.00	0.00	H
ATOM	264	CA	TYR	16	34.051	10.629	1.284	1.00	0.00	C
ATOM	265	HA	TYR	16	33.759	9.800	0.639	1.00	0.00	H
ATOM	266	CB	TYR	16	34.504	11.745	0.369	1.00	0.00	C
ATOM	267	HB2	TYR	16	35.153	11.311	-0.390	1.00	0.00	H
ATOM	268	HB3	TYR	16	35.071	12.367	1.061	1.00	0.00	H
ATOM	269	CG	TYR	16	33.336	12.540	-0.218	1.00	0.00	C
ATOM	270	CD1	TYR	16	33.462	13.946	-0.197	1.00	0.00	C
ATOM	271	HD1	TYR	16	34.339	14.461	0.165	1.00	0.00	H
ATOM	272	CE1	TYR	16	32.442	14.659	-0.825	1.00	0.00	C
ATOM	273	HE1	TYR	16	32.531	15.727	-0.696	1.00	0.00	H
ATOM	274	CZ	TYR	16	31.298	14.006	-1.328	1.00	0.00	C
ATOM	275	OH	TYR	16	30.257	14.733	-1.793	1.00	0.00	O
ATOM	276	HH	TYR	16	30.473	15.664	-1.708	1.00	0.00	H
ATOM	277	CE2	TYR	16	31.161	12.621	-1.292	1.00	0.00	C
ATOM	278	HE2	TYR	16	30.329	12.195	-1.831	1.00	0.00	H
ATOM	279	CD2	TYR	16	32.221	11.864	-0.800	1.00	0.00	C
ATOM	280	HD2	TYR	16	32.115	10.801	-0.956	1.00	0.00	H
ATOM	281	C	TYR	16	35.307	10.145	2.137	1.00	0.00	C
ATOM	282	O	TYR	16	35.476	10.484	3.313	1.00	0.00	O
ATOM	283	N	NME	17	36.128	9.329	1.502	1.00	0.00	N
ATOM	284	H	NME	17	35.935	8.983	0.573	1.00	0.00	H
ATOM	285	CH3	NME	17	37.205	8.677	2.198	1.00	0.00	C
ATOM	286	HH31	NME	17	37.889	8.291	1.442	1.00	0.00	H
ATOM	287	HH32	NME	17	37.720	9.419	2.808	1.00	0.00	H
ATOM	288	HH33	NME	17	36.844	7.915	2.890	1.00	0.00	H
TER	289		NME	17						
ATOM	289	HH31	ACE	18	13.543	17.305	4.279	1.00	0.00	H
ATOM	290	CH3	ACE	18	13.692	18.377	4.148	1.00	0.00	C
ATOM	291	HH32	ACE	18	12.867	18.866	4.665	1.00	0.00	H
ATOM	292	HH33	ACE	18	13.645	18.558	3.074	1.00	0.00	H
ATOM	293	C	ACE	18	15.022	18.723	4.717	1.00	0.00	C
ATOM	294	O	ACE	18	15.819	19.349	4.037	1.00	0.00	O
ATOM	295	N	LYS	19	15.340	18.310	5.925	1.00	0.00	N
ATOM	296	H	LYS	19	14.658	17.681	6.327	1.00	0.00	H
ATOM	297	CA	LYS	19	16.664	18.512	6.569	1.00	0.00	C
ATOM	298	HA	LYS	19	17.358	19.130	5.998	1.00	0.00	H
ATOM	299	CB	LYS	19	16.420	19.138	7.952	1.00	0.00	C
ATOM	300	HB2	LYS	19	15.831	20.049	7.838	1.00	0.00	H
ATOM	301	HB3	LYS	19	17.468	19.332	8.176	1.00	0.00	H
ATOM	302	CG	LYS	19	15.696	18.504	9.097	1.00	0.00	C
ATOM	303	HG2	LYS	19	16.123	17.560	9.437	1.00	0.00	H
ATOM	304	HG3	LYS	19	14.721	18.343	8.635	1.00	0.00	H
ATOM	305	CD	LYS	19	15.425	19.501	10.274	1.00	0.00	C

ATOM	306	HD2	LYS	19	14.938	20.410	9.921	1.00	0.00	H
ATOM	307	HD3	LYS	19	16.353	19.724	10.799	1.00	0.00	H
ATOM	308	CE	LYS	19	14.389	18.936	11.362	1.00	0.00	C
ATOM	309	HE2	LYS	19	13.450	18.530	10.987	1.00	0.00	H
ATOM	310	HE3	LYS	19	14.229	19.848	11.937	1.00	0.00	H
ATOM	311	NZ	LYS	19	15.010	17.893	12.203	1.00	0.00	N
ATOM	312	HZ1	LYS	19	15.921	18.141	12.561	1.00	0.00	H
ATOM	313	HZ2	LYS	19	14.398	17.565	12.936	1.00	0.00	H
ATOM	314	HZ3	LYS	19	15.182	17.128	11.564	1.00	0.00	H
ATOM	315	C	LYS	19	17.287	17.091	6.785	1.00	0.00	C
ATOM	316	O	LYS	19	16.602	16.047	6.738	1.00	0.00	O
ATOM	317	N	GLY	20	18.591	17.105	7.131	1.00	0.00	N
ATOM	318	H	GLY	20	19.081	17.980	7.247	1.00	0.00	H
ATOM	319	CA	GLY	20	19.303	15.846	7.318	1.00	0.00	C
ATOM	320	HA2	GLY	20	20.153	15.979	7.987	1.00	0.00	H
ATOM	321	HA3	GLY	20	18.600	15.248	7.898	1.00	0.00	H
ATOM	322	C	GLY	20	19.717	15.014	6.045	1.00	0.00	C
ATOM	323	O	GLY	20	19.555	15.461	4.902	1.00	0.00	O
ATOM	324	N	GLU	21	20.123	13.757	6.338	1.00	0.00	N
ATOM	325	H	GLU	21	20.093	13.532	7.322	1.00	0.00	H
ATOM	326	CA	GLU	21	20.708	12.841	5.367	1.00	0.00	C
ATOM	327	HA	GLU	21	20.353	13.178	4.394	1.00	0.00	H
ATOM	328	CB	GLU	21	22.236	12.925	5.598	1.00	0.00	C
ATOM	329	HB2	GLU	21	22.633	12.210	4.878	1.00	0.00	H
ATOM	330	HB3	GLU	21	22.427	12.679	6.643	1.00	0.00	H
ATOM	331	CG	GLU	21	22.829	14.350	5.385	1.00	0.00	C
ATOM	332	HG2	GLU	21	22.330	15.159	5.918	1.00	0.00	H
ATOM	333	HG3	GLU	21	22.419	14.457	4.381	1.00	0.00	H
ATOM	334	CD	GLU	21	24.354	14.418	5.392	1.00	0.00	C
ATOM	335	OE1	GLU	21	24.936	14.511	6.530	1.00	0.00	O
ATOM	336	OE2	GLU	21	25.096	14.466	4.372	1.00	0.00	O
ATOM	337	C	GLU	21	20.215	11.370	5.584	1.00	0.00	C
ATOM	338	O	GLU	21	19.810	10.957	6.692	1.00	0.00	O
ATOM	339	N	ASN	22	20.120	10.569	4.552	1.00	0.00	N
ATOM	340	H	ASN	22	20.431	11.003	3.694	1.00	0.00	H
ATOM	341	CA	ASN	22	19.424	9.218	4.574	1.00	0.00	C
ATOM	342	HA	ASN	22	18.651	9.279	5.340	1.00	0.00	H
ATOM	343	CB	ASN	22	18.835	9.054	3.157	1.00	0.00	C
ATOM	344	HB2	ASN	22	19.581	9.008	2.363	1.00	0.00	H
ATOM	345	HB3	ASN	22	18.320	9.986	2.929	1.00	0.00	H
ATOM	346	CG	ASN	22	17.843	7.848	3.033	1.00	0.00	C
ATOM	347	OD1	ASN	22	16.640	7.951	3.260	1.00	0.00	O
ATOM	348	ND2	ASN	22	18.291	6.645	2.746	1.00	0.00	N
ATOM	349	HD21	ASN	22	17.595	5.916	2.808	1.00	0.00	H
ATOM	350	HD22	ASN	22	19.275	6.510	2.928	1.00	0.00	H
ATOM	351	C	ASN	22	20.310	8.096	5.055	1.00	0.00	C
ATOM	352	O	ASN	22	19.887	6.949	5.091	1.00	0.00	O
ATOM	353	N	PHE	23	21.572	8.344	5.401	1.00	0.00	N
ATOM	354	H	PHE	23	21.806	9.321	5.509	1.00	0.00	H
ATOM	355	CA	PHE	23	22.560	7.314	5.792	1.00	0.00	C
ATOM	356	HA	PHE	23	21.991	6.439	6.107	1.00	0.00	H
ATOM	357	CB	PHE	23	23.443	6.911	4.650	1.00	0.00	C
ATOM	358	HB2	PHE	23	23.925	5.955	4.854	1.00	0.00	H
ATOM	359	HB3	PHE	23	24.209	7.684	4.586	1.00	0.00	H
ATOM	360	CG	PHE	23	22.713	6.669	3.425	1.00	0.00	C
ATOM	361	CD1	PHE	23	22.086	5.406	3.220	1.00	0.00	C
ATOM	362	HD1	PHE	23	22.313	4.512	3.783	1.00	0.00	H
ATOM	363	CE1	PHE	23	21.210	5.300	2.071	1.00	0.00	C
ATOM	364	HE1	PHE	23	20.628	4.392	2.013	1.00	0.00	H
ATOM	365	CZ	PHE	23	20.961	6.351	1.223	1.00	0.00	C
ATOM	366	HZ	PHE	23	20.261	6.127	0.431	1.00	0.00	H
ATOM	367	CE2	PHE	23	21.660	7.558	1.393	1.00	0.00	C
ATOM	368	HE2	PHE	23	21.493	8.368	0.699	1.00	0.00	H

ATOM	369	CD2	PHE	23	22.581	7.687	2.453	1.00	0.00	C
ATOM	370	HD2	PHE	23	23.136	8.613	2.451	1.00	0.00	H
ATOM	371	C	PHE	23	23.326	7.635	7.015	1.00	0.00	C
ATOM	372	O	PHE	23	23.288	8.794	7.487	1.00	0.00	O
ATOM	373	N	THR	24	24.049	6.629	7.505	1.00	0.00	N
ATOM	374	H	THR	24	23.875	5.710	7.122	1.00	0.00	H
ATOM	375	CA	THR	24	24.726	6.655	8.885	1.00	0.00	C
ATOM	376	HA	THR	24	24.688	7.714	9.141	1.00	0.00	H
ATOM	377	CB	THR	24	23.996	5.752	9.955	1.00	0.00	C
ATOM	378	HB	THR	24	24.487	4.779	9.920	1.00	0.00	H
ATOM	379	CG2	THR	24	24.129	6.326	11.366	1.00	0.00	C
ATOM	380	HG21	THR	24	23.735	5.575	12.051	1.00	0.00	H
ATOM	381	HG22	THR	24	25.149	6.645	11.582	1.00	0.00	H
ATOM	382	HG23	THR	24	23.566	7.248	11.513	1.00	0.00	H
ATOM	383	OG1	THR	24	22.570	5.611	9.763	1.00	0.00	O
ATOM	384	HG1	THR	24	22.218	4.985	10.400	1.00	0.00	H
ATOM	385	C	THR	24	26.233	6.330	8.844	1.00	0.00	C
ATOM	386	O	THR	24	26.649	5.473	8.030	1.00	0.00	O
ATOM	387	N	GLU	25	27.086	6.848	9.724	1.00	0.00	N
ATOM	388	H	GLU	25	26.748	7.432	10.476	1.00	0.00	H
ATOM	389	CA	GLU	25	28.533	6.615	9.764	1.00	0.00	C
ATOM	390	HA	GLU	25	28.862	6.852	8.752	1.00	0.00	H
ATOM	391	CB	GLU	25	29.274	7.665	10.576	1.00	0.00	C
ATOM	392	HB2	GLU	25	28.968	8.626	10.165	1.00	0.00	H
ATOM	393	HB3	GLU	25	30.322	7.386	10.464	1.00	0.00	H
ATOM	394	CG	GLU	25	29.033	7.680	12.090	1.00	0.00	C
ATOM	395	HG2	GLU	25	28.902	6.629	12.343	1.00	0.00	H
ATOM	396	HG3	GLU	25	28.124	8.266	12.224	1.00	0.00	H
ATOM	397	CD	GLU	25	30.228	8.352	12.775	1.00	0.00	C
ATOM	398	OE1	GLU	25	30.144	9.595	12.989	1.00	0.00	O
ATOM	399	OE2	GLU	25	31.268	7.733	13.052	1.00	0.00	O
ATOM	400	C	GLU	25	28.962	5.171	10.097	1.00	0.00	C
ATOM	401	O	GLU	25	30.170	4.874	10.060	1.00	0.00	O
ATOM	402	N	THR	26	28.007	4.261	10.528	1.00	0.00	N
ATOM	403	H	THR	26	27.041	4.476	10.733	1.00	0.00	H
ATOM	404	CA	THR	26	28.285	2.869	10.828	1.00	0.00	C
ATOM	405	HA	THR	26	29.275	2.890	11.286	1.00	0.00	H
ATOM	406	CB	THR	26	27.299	2.340	11.923	1.00	0.00	C
ATOM	407	HB	THR	26	27.194	1.259	11.837	1.00	0.00	H
ATOM	408	CG2	THR	26	27.739	2.621	13.358	1.00	0.00	C
ATOM	409	HG21	THR	26	27.070	2.167	14.089	1.00	0.00	H
ATOM	410	HG22	THR	26	28.743	2.199	13.356	1.00	0.00	H
ATOM	411	HG23	THR	26	27.799	3.665	13.668	1.00	0.00	H
ATOM	412	OG1	THR	26	26.027	2.844	11.693	1.00	0.00	O
ATOM	413	HG1	THR	26	25.474	2.218	12.166	1.00	0.00	H
ATOM	414	C	THR	26	28.204	1.865	9.634	1.00	0.00	C
ATOM	415	O	THR	26	28.422	0.671	9.779	1.00	0.00	O
ATOM	416	N	ASP	27	27.857	2.326	8.430	1.00	0.00	N
ATOM	417	H	ASP	27	27.715	3.321	8.325	1.00	0.00	H
ATOM	418	CA	ASP	27	27.674	1.370	7.290	1.00	0.00	C
ATOM	419	HA	ASP	27	27.391	0.371	7.619	1.00	0.00	H
ATOM	420	CB	ASP	27	26.362	1.800	6.497	1.00	0.00	C
ATOM	421	HB2	ASP	27	26.435	2.707	5.896	1.00	0.00	H
ATOM	422	HB3	ASP	27	25.581	2.046	7.216	1.00	0.00	H
ATOM	423	CG	ASP	27	25.799	0.623	5.603	1.00	0.00	C
ATOM	424	OD1	ASP	27	25.784	0.683	4.366	1.00	0.00	O
ATOM	425	OD2	ASP	27	25.429	-0.437	6.091	1.00	0.00	O
ATOM	426	C	ASP	27	28.825	1.222	6.316	1.00	0.00	C
ATOM	427	O	ASP	27	29.046	0.149	5.759	1.00	0.00	O
ATOM	428	N	ILE	28	29.430	2.338	6.062	1.00	0.00	N
ATOM	429	H	ILE	28	29.008	3.203	6.368	1.00	0.00	H
ATOM	430	CA	ILE	28	30.623	2.454	5.235	1.00	0.00	C
ATOM	431	HA	ILE	28	30.494	1.881	4.318	1.00	0.00	H

ATOM	432	CB	ILE	28	30.856	3.955	4.883	1.00	0.00	C
ATOM	433	HB	ILE	28	31.775	4.029	4.300	1.00	0.00	H
ATOM	434	CG2	ILE	28	29.731	4.379	3.885	1.00	0.00	C
ATOM	435	HG21	ILE	28	29.453	3.550	3.234	1.00	0.00	H
ATOM	436	HG22	ILE	28	28.823	4.622	4.436	1.00	0.00	H
ATOM	437	HG23	ILE	28	29.963	5.265	3.293	1.00	0.00	H
ATOM	438	CG1	ILE	28	30.922	4.856	6.180	1.00	0.00	C
ATOM	439	HG12	ILE	28	31.337	4.357	7.055	1.00	0.00	H
ATOM	440	HG13	ILE	28	29.871	5.050	6.393	1.00	0.00	H
ATOM	441	CD1	ILE	28	31.780	6.147	6.068	1.00	0.00	C
ATOM	442	HD11	ILE	28	32.852	5.958	6.132	1.00	0.00	H
ATOM	443	HD12	ILE	28	31.569	6.763	5.194	1.00	0.00	H
ATOM	444	HD13	ILE	28	31.518	6.814	6.888	1.00	0.00	H
ATOM	445	C	ILE	28	31.826	1.798	5.873	1.00	0.00	C
ATOM	446	O	ILE	28	32.854	1.706	5.194	1.00	0.00	O
ATOM	447	N	LYS	29	31.829	1.438	7.229	1.00	0.00	N
ATOM	448	H	LYS	29	30.989	1.546	7.779	1.00	0.00	H
ATOM	449	CA	LYS	29	32.997	0.961	7.919	1.00	0.00	C
ATOM	450	HA	LYS	29	33.856	1.589	7.682	1.00	0.00	H
ATOM	451	CB	LYS	29	32.733	0.996	9.459	1.00	0.00	C
ATOM	452	HB2	LYS	29	33.606	0.546	9.930	1.00	0.00	H
ATOM	453	HB3	LYS	29	31.954	0.242	9.579	1.00	0.00	H
ATOM	454	CG	LYS	29	32.348	2.270	10.169	1.00	0.00	C
ATOM	455	HG2	LYS	29	32.002	1.997	11.166	1.00	0.00	H
ATOM	456	HG3	LYS	29	31.554	2.689	9.551	1.00	0.00	H
ATOM	457	CD	LYS	29	33.532	3.259	10.307	1.00	0.00	C
ATOM	458	HD2	LYS	29	34.163	3.136	9.425	1.00	0.00	H
ATOM	459	HD3	LYS	29	34.124	3.051	11.198	1.00	0.00	H
ATOM	460	CE	LYS	29	33.119	4.706	10.345	1.00	0.00	C
ATOM	461	HE2	LYS	29	32.445	4.792	11.197	1.00	0.00	H
ATOM	462	HE3	LYS	29	32.666	4.844	9.363	1.00	0.00	H
ATOM	463	NZ	LYS	29	34.308	5.536	10.678	1.00	0.00	N
ATOM	464	HZ1	LYS	29	34.104	6.500	10.455	1.00	0.00	H
ATOM	465	HZ2	LYS	29	34.637	5.468	11.630	1.00	0.00	H
ATOM	466	HZ3	LYS	29	35.112	5.343	10.098	1.00	0.00	H
ATOM	467	C	LYS	29	33.524	-0.439	7.463	1.00	0.00	C
ATOM	468	O	LYS	29	34.614	-0.860	7.753	1.00	0.00	O
ATOM	469	N	ILE	30	32.647	-1.227	6.859	1.00	0.00	N
ATOM	470	H	ILE	30	31.783	-0.773	6.602	1.00	0.00	H
ATOM	471	CA	ILE	30	32.832	-2.637	6.525	1.00	0.00	C
ATOM	472	HA	ILE	30	33.665	-2.936	7.162	1.00	0.00	H
ATOM	473	CB	ILE	30	31.560	-3.345	7.017	1.00	0.00	C
ATOM	474	HB	ILE	30	31.499	-4.267	6.438	1.00	0.00	H
ATOM	475	CG2	ILE	30	31.598	-3.684	8.491	1.00	0.00	C
ATOM	476	HG21	ILE	30	32.486	-4.253	8.768	1.00	0.00	H
ATOM	477	HG22	ILE	30	31.558	-2.770	9.083	1.00	0.00	H
ATOM	478	HG23	ILE	30	30.806	-4.384	8.755	1.00	0.00	H
ATOM	479	CG1	ILE	30	30.256	-2.559	6.729	1.00	0.00	C
ATOM	480	HG12	ILE	30	30.200	-2.087	5.749	1.00	0.00	H
ATOM	481	HG13	ILE	30	30.194	-1.877	7.577	1.00	0.00	H
ATOM	482	CD1	ILE	30	28.975	-3.371	6.680	1.00	0.00	C
ATOM	483	HD11	ILE	30	28.133	-2.682	6.611	1.00	0.00	H
ATOM	484	HD12	ILE	30	29.055	-3.980	5.780	1.00	0.00	H
ATOM	485	HD13	ILE	30	28.897	-4.071	7.513	1.00	0.00	H
ATOM	486	C	ILE	30	33.016	-2.753	5.007	1.00	0.00	C
ATOM	487	O	ILE	30	32.147	-2.190	4.313	1.00	0.00	O
ATOM	488	N	MET	31	34.010	-3.484	4.522	1.00	0.00	N
ATOM	489	H	MET	31	34.580	-4.043	5.140	1.00	0.00	H
ATOM	490	CA	MET	31	34.189	-3.753	3.093	1.00	0.00	C
ATOM	491	HA	MET	31	33.731	-2.971	2.487	1.00	0.00	H
ATOM	492	CB	MET	31	35.646	-3.612	2.728	1.00	0.00	C
ATOM	493	HB2	MET	31	35.795	-3.566	1.648	1.00	0.00	H
ATOM	494	HB3	MET	31	36.122	-4.475	3.191	1.00	0.00	H

```
ATOM    495  CG  MET    31      36.308  -2.307   3.291  1.00  0.00           C
ATOM    496  HG2 MET    31      36.290  -2.297   4.381  1.00  0.00           H
ATOM    497  HG3 MET    31      35.754  -1.490   2.832  1.00  0.00           H
ATOM    498  SD  MET    31      37.975  -1.949   2.801  1.00  0.00           S
ATOM    499  CE  MET    31      38.967  -2.149   4.300  1.00  0.00           C
ATOM    500  HE1 MET    31      38.759  -3.110   4.772  1.00  0.00           H
ATOM    501  HE2 MET    31      40.018  -2.156   4.016  1.00  0.00           H
ATOM    502  HE3 MET    31      38.688  -1.392   5.033  1.00  0.00           H
ATOM    503  C   MET    31      33.513  -4.965   2.567  1.00  0.00           C
ATOM    504  O   MET    31      33.303  -5.018   1.368  1.00  0.00           O
ATOM    505  N   GLU    32      32.906  -5.775   3.421  1.00  0.00           N
ATOM    506  H   GLU    32      33.084  -5.525   4.383  1.00  0.00           H
ATOM    507  CA  GLU    32      32.399  -7.147   3.163  1.00  0.00           C
ATOM    508  HA  GLU    32      32.259  -7.209   2.084  1.00  0.00           H
ATOM    509  CB  GLU    32      33.379  -8.146   3.808  1.00  0.00           C
ATOM    510  HB2 GLU    32      33.566  -7.962   4.866  1.00  0.00           H
ATOM    511  HB3 GLU    32      34.227  -7.808   3.211  1.00  0.00           H
ATOM    512  CG  GLU    32      33.018  -9.587   3.476  1.00  0.00           C
ATOM    513  HG2 GLU    32      32.993  -9.712   2.393  1.00  0.00           H
ATOM    514  HG3 GLU    32      32.065  -9.682   3.999  1.00  0.00           H
ATOM    515  CD  GLU    32      33.966 -10.613   4.133  1.00  0.00           C
ATOM    516  OE1 GLU    32      34.348 -11.607   3.468  1.00  0.00           O
ATOM    517  OE2 GLU    32      34.164 -10.416   5.328  1.00  0.00           O
ATOM    518  C   GLU    32      30.985  -7.139   3.799  1.00  0.00           C
ATOM    519  O   GLU    32      30.767  -6.695   4.950  1.00  0.00           O
ATOM    520  N   ARG    33      29.925  -7.399   3.007  1.00  0.00           N
ATOM    521  H   ARG    33      30.153  -7.479   2.027  1.00  0.00           H
ATOM    522  CA  ARG    33      28.499  -7.464   3.435  1.00  0.00           C
ATOM    523  HA  ARG    33      28.426  -7.167   4.481  1.00  0.00           H
ATOM    524  CB  ARG    33      27.601  -6.561   2.608  1.00  0.00           C
ATOM    525  HB2 ARG    33      26.578  -6.563   2.985  1.00  0.00           H
ATOM    526  HB3 ARG    33      27.724  -6.823   1.557  1.00  0.00           H
ATOM    527  CG  ARG    33      28.109  -5.113   2.664  1.00  0.00           C
ATOM    528  HG2 ARG    33      29.118  -4.905   2.305  1.00  0.00           H
ATOM    529  HG3 ARG    33      28.108  -4.942   3.740  1.00  0.00           H
ATOM    530  CD  ARG    33      27.185  -4.113   1.997  1.00  0.00           C
ATOM    531  HD2 ARG    33      26.784  -4.592   1.104  1.00  0.00           H
ATOM    532  HD3 ARG    33      27.860  -3.279   1.802  1.00  0.00           H
ATOM    533  NE  ARG    33      26.110  -3.800   2.966  1.00  0.00           N
ATOM    534  HE  ARG    33      25.282  -4.381   2.966  1.00  0.00           H
ATOM    535  CZ  ARG    33      26.092  -2.608   3.595  1.00  0.00           C
ATOM    536  NH1 ARG    33      27.018  -1.626   3.510  1.00  0.00           N
ATOM    537 HH11 ARG    33      27.912  -1.748   3.058  1.00  0.00           H
ATOM    538 HH12 ARG    33      26.794  -0.852   4.119  1.00  0.00           H
ATOM    539  NH2 ARG    33      25.048  -2.361   4.342  1.00  0.00           N
ATOM    540 HH21 ARG    33      24.274  -2.997   4.472  1.00  0.00           H
ATOM    541 HH22 ARG    33      24.964  -1.429   4.720  1.00  0.00           H
ATOM    542  C   ARG    33      27.991  -8.935   3.379  1.00  0.00           C
ATOM    543  O   ARG    33      28.652  -9.853   2.868  1.00  0.00           O
ATOM    544  N   NME    34      26.763  -9.163   3.863  1.00  0.00           N
ATOM    545  H   NME    34      26.410  -8.369   4.376  1.00  0.00           H
ATOM    546  CH3 NME    34      26.084 -10.473   3.898  1.00  0.00           C
ATOM    547 HH31 NME    34      25.659 -10.700   2.920  1.00  0.00           H
ATOM    548 HH32 NME    34      26.796 -11.249   4.178  1.00  0.00           H
ATOM    549 HH33 NME    34      25.321 -10.451   4.675  1.00  0.00           H
TER     550      NME    34
END
```

Cluster 3, Figure 4C:

```
ATOM      1 HH31 ACE     1      21.059   3.080   0.377  1.00  0.00           H
```

ATOM	2	CH3	ACE	1	20.057	3.454	0.167	1.00	0.00	C
ATOM	3	HH32	ACE	1	19.483	3.379	1.091	1.00	0.00	H
ATOM	4	HH33	ACE	1	19.559	2.857	-0.596	1.00	0.00	H
ATOM	5	C	ACE	1	20.313	4.896	-0.185	1.00	0.00	C
ATOM	6	O	ACE	1	21.300	5.102	-0.909	1.00	0.00	O
ATOM	7	N	ASN	2	19.432	5.806	0.262	1.00	0.00	N
ATOM	8	H	ASN	2	18.755	5.391	0.887	1.00	0.00	H
ATOM	9	CA	ASN	2	19.577	7.250	0.105	1.00	0.00	C
ATOM	10	HA	ASN	2	19.313	7.453	-0.933	1.00	0.00	H
ATOM	11	CB	ASN	2	18.602	7.948	1.079	1.00	0.00	C
ATOM	12	HB2	ASN	2	18.637	7.513	2.078	1.00	0.00	H
ATOM	13	HB3	ASN	2	17.616	7.682	0.697	1.00	0.00	H
ATOM	14	CG	ASN	2	18.730	9.417	1.247	1.00	0.00	C
ATOM	15	OD1	ASN	2	17.950	10.170	0.663	1.00	0.00	O
ATOM	16	ND2	ASN	2	19.623	9.957	2.066	1.00	0.00	N
ATOM	17	HD21	ASN	2	19.500	10.953	2.181	1.00	0.00	H
ATOM	18	HD22	ASN	2	20.303	9.372	2.528	1.00	0.00	H
ATOM	19	C	ASN	2	21.034	7.760	0.345	1.00	0.00	C
ATOM	20	O	ASN	2	21.636	8.550	-0.410	1.00	0.00	O
ATOM	21	N	ASP	3	21.694	7.313	1.430	1.00	0.00	N
ATOM	22	H	ASP	3	21.014	6.837	2.006	1.00	0.00	H
ATOM	23	CA	ASP	3	23.049	7.637	1.897	1.00	0.00	C
ATOM	24	HA	ASP	3	23.220	8.708	1.994	1.00	0.00	H
ATOM	25	CB	ASP	3	23.187	7.011	3.304	1.00	0.00	C
ATOM	26	HB2	ASP	3	22.819	5.985	3.284	1.00	0.00	H
ATOM	27	HB3	ASP	3	22.602	7.630	3.984	1.00	0.00	H
ATOM	28	CG	ASP	3	24.610	6.997	3.801	1.00	0.00	C
ATOM	29	OD1	ASP	3	25.240	5.989	3.593	1.00	0.00	O
ATOM	30	OD2	ASP	3	25.189	8.035	4.196	1.00	0.00	O
ATOM	31	C	ASP	3	24.177	7.023	0.926	1.00	0.00	C
ATOM	32	O	ASP	3	25.354	7.385	1.017	1.00	0.00	O
ATOM	33	N	TYR	4	23.806	6.318	-0.189	1.00	0.00	N
ATOM	34	H	TYR	4	22.859	5.980	-0.291	1.00	0.00	H
ATOM	35	CA	TYR	4	24.674	5.983	-1.331	1.00	0.00	C
ATOM	36	HA	TYR	4	25.708	5.993	-0.986	1.00	0.00	H
ATOM	37	CB	TYR	4	24.425	4.482	-1.743	1.00	0.00	C
ATOM	38	HB2	TYR	4	25.126	4.166	-2.518	1.00	0.00	H
ATOM	39	HB3	TYR	4	23.421	4.501	-2.164	1.00	0.00	H
ATOM	40	CG	TYR	4	24.401	3.389	-0.680	1.00	0.00	C
ATOM	41	CD1	TYR	4	25.232	3.536	0.537	1.00	0.00	C
ATOM	42	HD1	TYR	4	25.914	4.356	0.703	1.00	0.00	H
ATOM	43	CE1	TYR	4	25.150	2.548	1.505	1.00	0.00	C
ATOM	44	HE1	TYR	4	25.663	2.613	2.454	1.00	0.00	H
ATOM	45	CZ	TYR	4	24.187	1.520	1.368	1.00	0.00	C
ATOM	46	OH	TYR	4	24.085	0.709	2.498	1.00	0.00	O
ATOM	47	HH	TYR	4	23.593	-0.071	2.231	1.00	0.00	H
ATOM	48	CE2	TYR	4	23.398	1.384	0.199	1.00	0.00	C
ATOM	49	HE2	TYR	4	22.648	0.612	0.110	1.00	0.00	H
ATOM	50	CD2	TYR	4	23.550	2.288	-0.840	1.00	0.00	C
ATOM	51	HD2	TYR	4	22.885	2.291	-1.691	1.00	0.00	H
ATOM	52	C	TYR	4	24.438	6.910	-2.539	1.00	0.00	C
ATOM	53	O	TYR	4	24.977	6.622	-3.573	1.00	0.00	O
ATOM	54	N	GLU	5	23.761	8.030	-2.332	1.00	0.00	N
ATOM	55	H	GLU	5	23.296	8.246	-1.461	1.00	0.00	H
ATOM	56	CA	GLU	5	23.763	9.123	-3.332	1.00	0.00	C
ATOM	57	HA	GLU	5	23.841	8.723	-4.343	1.00	0.00	H
ATOM	58	CB	GLU	5	22.463	9.944	-3.149	1.00	0.00	C
ATOM	59	HB2	GLU	5	22.662	10.853	-3.717	1.00	0.00	H
ATOM	60	HB3	GLU	5	22.495	10.154	-2.081	1.00	0.00	H
ATOM	61	CG	GLU	5	21.053	9.507	-3.600	1.00	0.00	C
ATOM	62	HG2	GLU	5	20.360	10.300	-3.319	1.00	0.00	H
ATOM	63	HG3	GLU	5	20.905	8.689	-2.893	1.00	0.00	H
ATOM	64	CD	GLU	5	20.678	9.124	-4.981	1.00	0.00	C

ATOM	65	OE1	GLU	5	21.184	8.072	-5.472	1.00	0.00	O
ATOM	66	OE2	GLU	5	20.037	9.904	-5.718	1.00	0.00	O
ATOM	67	C	GLU	5	24.951	10.087	-3.217	1.00	0.00	C
ATOM	68	O	GLU	5	25.555	10.276	-2.130	1.00	0.00	O
ATOM	69	N	ASP	6	25.289	10.860	-4.226	1.00	0.00	N
ATOM	70	H	ASP	6	24.727	10.830	-5.065	1.00	0.00	H
ATOM	71	CA	ASP	6	26.609	11.634	-4.303	1.00	0.00	C
ATOM	72	HA	ASP	6	27.419	10.938	-4.081	1.00	0.00	H
ATOM	73	CB	ASP	6	26.833	12.104	-5.753	1.00	0.00	C
ATOM	74	HB2	ASP	6	26.863	11.151	-6.281	1.00	0.00	H
ATOM	75	HB3	ASP	6	27.769	12.660	-5.806	1.00	0.00	H
ATOM	76	CG	ASP	6	25.716	12.952	-6.288	1.00	0.00	C
ATOM	77	OD1	ASP	6	24.985	12.517	-7.206	1.00	0.00	O
ATOM	78	OD2	ASP	6	25.618	14.143	-5.985	1.00	0.00	O
ATOM	79	C	ASP	6	26.721	12.765	-3.252	1.00	0.00	C
ATOM	80	O	ASP	6	27.833	13.235	-3.028	1.00	0.00	O
ATOM	81	N	ARG	7	25.619	13.213	-2.660	1.00	0.00	N
ATOM	82	H	ARG	7	24.775	13.014	-3.176	1.00	0.00	H
ATOM	83	CA	ARG	7	25.709	14.290	-1.621	1.00	0.00	C
ATOM	84	HA	ARG	7	26.487	15.000	-1.902	1.00	0.00	H
ATOM	85	CB	ARG	7	24.325	15.030	-1.465	1.00	0.00	C
ATOM	86	HB2	ARG	7	24.301	15.682	-0.592	1.00	0.00	H
ATOM	87	HB3	ARG	7	23.623	14.197	-1.515	1.00	0.00	H
ATOM	88	CG	ARG	7	23.953	15.883	-2.663	1.00	0.00	C
ATOM	89	HG2	ARG	7	23.037	16.407	-2.390	1.00	0.00	H
ATOM	90	HG3	ARG	7	23.826	15.270	-3.555	1.00	0.00	H
ATOM	91	CD	ARG	7	25.020	17.004	-3.065	1.00	0.00	C
ATOM	92	HD2	ARG	7	25.408	17.522	-2.189	1.00	0.00	H
ATOM	93	HD3	ARG	7	24.551	17.742	-3.716	1.00	0.00	H
ATOM	94	NE	ARG	7	26.249	16.600	-3.816	1.00	0.00	N
ATOM	95	HE	ARG	7	26.102	15.906	-4.534	1.00	0.00	H
ATOM	96	CZ	ARG	7	27.502	16.975	-3.582	1.00	0.00	C
ATOM	97	NH1	ARG	7	27.913	17.610	-2.522	1.00	0.00	N
ATOM	98	HH11	ARG	7	27.239	18.033	-1.898	1.00	0.00	H
ATOM	99	HH12	ARG	7	28.906	17.732	-2.386	1.00	0.00	H
ATOM	100	NH2	ARG	7	28.390	16.547	-4.463	1.00	0.00	N
ATOM	101	HH21	ARG	7	28.101	16.040	-5.288	1.00	0.00	H
ATOM	102	HH22	ARG	7	29.386	16.677	-4.353	1.00	0.00	H
ATOM	103	C	ARG	7	26.225	13.617	-0.340	1.00	0.00	C
ATOM	104	O	ARG	7	26.477	14.369	0.612	1.00	0.00	O
ATOM	105	N	TYR	8	26.239	12.284	-0.189	1.00	0.00	N
ATOM	106	H	TYR	8	26.036	11.715	-0.998	1.00	0.00	H
ATOM	107	CA	TYR	8	26.590	11.602	1.080	1.00	0.00	C
ATOM	108	HA	TYR	8	26.922	12.243	1.896	1.00	0.00	H
ATOM	109	CB	TYR	8	25.268	10.969	1.631	1.00	0.00	C
ATOM	110	HB2	TYR	8	25.445	10.671	2.664	1.00	0.00	H
ATOM	111	HB3	TYR	8	25.196	10.135	0.934	1.00	0.00	H
ATOM	112	CG	TYR	8	24.036	11.854	1.593	1.00	0.00	C
ATOM	113	CD1	TYR	8	23.849	12.850	2.533	1.00	0.00	C
ATOM	114	HD1	TYR	8	24.713	13.072	3.140	1.00	0.00	H
ATOM	115	CE1	TYR	8	22.682	13.572	2.601	1.00	0.00	C
ATOM	116	HE1	TYR	8	22.718	14.419	3.271	1.00	0.00	H
ATOM	117	CZ	TYR	8	21.605	13.284	1.756	1.00	0.00	C
ATOM	118	OH	TYR	8	20.471	14.015	1.834	1.00	0.00	O
ATOM	119	HH	TYR	8	20.599	14.763	2.421	1.00	0.00	H
ATOM	120	CE2	TYR	8	21.685	12.214	0.852	1.00	0.00	C
ATOM	121	HE2	TYR	8	20.874	11.844	0.240	1.00	0.00	H
ATOM	122	CD2	TYR	8	22.897	11.515	0.750	1.00	0.00	C
ATOM	123	HD2	TYR	8	23.024	10.660	0.104	1.00	0.00	H
ATOM	124	C	TYR	8	27.665	10.549	0.861	1.00	0.00	C
ATOM	125	O	TYR	8	28.350	10.167	1.793	1.00	0.00	O
ATOM	126	N	TYR	9	27.885	10.085	-0.424	1.00	0.00	N
ATOM	127	H	TYR	9	27.283	10.441	-1.153	1.00	0.00	H

ATOM	128	CA	TYR	9	28.933	9.163	-0.785	1.00	0.00	C
ATOM	129	HA	TYR	9	28.737	8.218	-0.281	1.00	0.00	H
ATOM	130	CB	TYR	9	28.782	8.865	-2.293	1.00	0.00	C
ATOM	131	HB2	TYR	9	29.058	9.717	-2.917	1.00	0.00	H
ATOM	132	HB3	TYR	9	27.739	8.550	-2.323	1.00	0.00	H
ATOM	133	CG	TYR	9	29.600	7.708	-2.859	1.00	0.00	C
ATOM	134	CD1	TYR	9	30.881	8.062	-3.389	1.00	0.00	C
ATOM	135	HD1	TYR	9	31.323	9.045	-3.341	1.00	0.00	H
ATOM	136	CE1	TYR	9	31.785	7.046	-3.827	1.00	0.00	C
ATOM	137	HE1	TYR	9	32.785	7.297	-4.152	1.00	0.00	H
ATOM	138	CZ	TYR	9	31.433	5.742	-3.638	1.00	0.00	C
ATOM	139	OH	TYR	9	32.198	4.731	-4.145	1.00	0.00	O
ATOM	140	HH	TYR	9	31.842	3.846	-4.038	1.00	0.00	H
ATOM	141	CE2	TYR	9	30.110	5.434	-3.178	1.00	0.00	C
ATOM	142	HE2	TYR	9	29.711	4.430	-3.203	1.00	0.00	H
ATOM	143	CD2	TYR	9	29.188	6.367	-2.718	1.00	0.00	C
ATOM	144	HD2	TYR	9	28.310	5.983	-2.221	1.00	0.00	H
ATOM	145	C	TYR	9	30.396	9.553	-0.405	1.00	0.00	C
ATOM	146	O	TYR	9	30.695	10.725	-0.427	1.00	0.00	O
ATOM	147	N	ARG	10	31.231	8.555	-0.014	1.00	0.00	N
ATOM	148	H	ARG	10	30.729	7.680	0.030	1.00	0.00	H
ATOM	149	CA	ARG	10	32.658	8.644	0.488	1.00	0.00	C
ATOM	150	HA	ARG	10	33.165	9.458	-0.030	1.00	0.00	H
ATOM	151	CB	ARG	10	32.725	9.111	1.992	1.00	0.00	C
ATOM	152	HB2	ARG	10	32.006	9.907	2.185	1.00	0.00	H
ATOM	153	HB3	ARG	10	33.781	9.337	2.131	1.00	0.00	H
ATOM	154	CG	ARG	10	32.285	8.115	3.074	1.00	0.00	C
ATOM	155	HG2	ARG	10	32.882	8.300	3.968	1.00	0.00	H
ATOM	156	HG3	ARG	10	32.484	7.144	2.618	1.00	0.00	H
ATOM	157	CD	ARG	10	30.868	8.066	3.677	1.00	0.00	C
ATOM	158	HD2	ARG	10	30.530	9.070	3.931	1.00	0.00	H
ATOM	159	HD3	ARG	10	30.924	7.520	4.619	1.00	0.00	H
ATOM	160	NE	ARG	10	29.848	7.505	2.771	1.00	0.00	N
ATOM	161	HE	ARG	10	30.214	7.028	1.960	1.00	0.00	H
ATOM	162	CZ	ARG	10	28.526	7.334	2.978	1.00	0.00	C
ATOM	163	NH1	ARG	10	27.904	7.738	4.005	1.00	0.00	N
ATOM	164	HH11	ARG	10	28.316	8.234	4.782	1.00	0.00	H
ATOM	165	HH12	ARG	10	26.938	7.445	3.973	1.00	0.00	H
ATOM	166	NH2	ARG	10	27.903	6.688	2.049	1.00	0.00	N
ATOM	167	HH21	ARG	10	28.384	6.348	1.229	1.00	0.00	H
ATOM	168	HH22	ARG	10	26.905	6.832	2.000	1.00	0.00	H
ATOM	169	C	ARG	10	33.552	7.411	0.278	1.00	0.00	C
ATOM	170	O	ARG	10	34.778	7.485	0.537	1.00	0.00	O
ATOM	171	N	GLU	11	32.891	6.272	-0.108	1.00	0.00	N
ATOM	172	H	GLU	11	31.948	6.477	-0.408	1.00	0.00	H
ATOM	173	CA	GLU	11	33.407	4.930	-0.140	1.00	0.00	C
ATOM	174	HA	GLU	11	33.916	4.629	0.775	1.00	0.00	H
ATOM	175	CB	GLU	11	32.217	3.967	-0.310	1.00	0.00	C
ATOM	176	HB2	GLU	11	32.705	2.999	-0.190	1.00	0.00	H
ATOM	177	HB3	GLU	11	31.876	4.130	-1.333	1.00	0.00	H
ATOM	178	CG	GLU	11	31.095	3.869	0.698	1.00	0.00	C
ATOM	179	HG2	GLU	11	31.555	3.977	1.680	1.00	0.00	H
ATOM	180	HG3	GLU	11	30.686	2.875	0.513	1.00	0.00	H
ATOM	181	CD	GLU	11	29.939	4.811	0.360	1.00	0.00	C
ATOM	182	OE1	GLU	11	30.074	6.042	0.471	1.00	0.00	O
ATOM	183	OE2	GLU	11	28.804	4.295	0.277	1.00	0.00	O
ATOM	184	C	GLU	11	34.460	4.787	-1.258	1.00	0.00	C
ATOM	185	O	GLU	11	34.432	5.466	-2.285	1.00	0.00	O
ATOM	186	N	ASN	12	35.454	3.843	-1.010	1.00	0.00	N
ATOM	187	H	ASN	12	35.471	3.343	-0.132	1.00	0.00	H
ATOM	188	CA	ASN	12	36.263	3.377	-2.085	1.00	0.00	C
ATOM	189	HA	ASN	12	36.745	4.252	-2.521	1.00	0.00	H
ATOM	190	CB	ASN	12	37.352	2.584	-1.371	1.00	0.00	C

ATOM	191	HB2	ASN	12	36.893	1.852	-0.706	1.00	0.00	H
ATOM	192	HB3	ASN	12	37.840	3.370	-0.796	1.00	0.00	H
ATOM	193	CG	ASN	12	38.392	1.907	-2.289	1.00	0.00	C
ATOM	194	OD1	ASN	12	38.157	1.568	-3.459	1.00	0.00	O
ATOM	195	ND2	ASN	12	39.579	1.567	-1.736	1.00	0.00	N
ATOM	196	HD21	ASN	12	40.191	1.133	-2.413	1.00	0.00	H
ATOM	197	HD22	ASN	12	39.806	1.917	-0.817	1.00	0.00	H
ATOM	198	C	ASN	12	35.378	2.613	-3.136	1.00	0.00	C
ATOM	199	O	ASN	12	35.032	3.025	-4.226	1.00	0.00	O
ATOM	200	N	MET	13	34.790	1.483	-2.643	1.00	0.00	N
ATOM	201	H	MET	13	34.914	1.210	-1.678	1.00	0.00	H
ATOM	202	CA	MET	13	33.825	0.608	-3.334	1.00	0.00	C
ATOM	203	HA	MET	13	34.372	0.165	-4.168	1.00	0.00	H
ATOM	204	CB	MET	13	33.359	-0.541	-2.438	1.00	0.00	C
ATOM	205	HB2	MET	13	34.218	-1.146	-2.151	1.00	0.00	H
ATOM	206	HB3	MET	13	32.713	-1.212	-3.006	1.00	0.00	H
ATOM	207	CG	MET	13	32.649	-0.026	-1.158	1.00	0.00	C
ATOM	208	HG2	MET	13	31.985	-0.793	-0.760	1.00	0.00	H
ATOM	209	HG3	MET	13	32.002	0.749	-1.570	1.00	0.00	H
ATOM	210	SD	MET	13	33.721	0.526	0.312	1.00	0.00	S
ATOM	211	CE	MET	13	34.779	-0.866	0.641	1.00	0.00	C
ATOM	212	HE1	MET	13	35.609	-0.902	-0.065	1.00	0.00	H
ATOM	213	HE2	MET	13	35.192	-0.939	1.648	1.00	0.00	H
ATOM	214	HE3	MET	13	34.336	-1.854	0.518	1.00	0.00	H
ATOM	215	C	MET	13	32.696	1.371	-4.065	1.00	0.00	C
ATOM	216	O	MET	13	31.872	2.185	-3.545	1.00	0.00	O
ATOM	217	N	TYR	14	32.271	0.847	-5.203	1.00	0.00	N
ATOM	218	H	TYR	14	32.619	-0.075	-5.423	1.00	0.00	H
ATOM	219	CA	TYR	14	31.207	1.342	-6.156	1.00	0.00	C
ATOM	220	HA	TYR	14	30.951	2.356	-5.851	1.00	0.00	H
ATOM	221	CB	TYR	14	31.826	1.386	-7.600	1.00	0.00	C
ATOM	222	HB2	TYR	14	31.908	0.400	-8.055	1.00	0.00	H
ATOM	223	HB3	TYR	14	32.805	1.854	-7.493	1.00	0.00	H
ATOM	224	CG	TYR	14	31.185	2.303	-8.704	1.00	0.00	C
ATOM	225	CD1	TYR	14	31.757	3.544	-8.920	1.00	0.00	C
ATOM	226	HD1	TYR	14	32.648	3.817	-8.374	1.00	0.00	H
ATOM	227	CE1	TYR	14	31.209	4.435	-9.925	1.00	0.00	C
ATOM	228	HE1	TYR	14	31.604	5.424	-10.107	1.00	0.00	H
ATOM	229	CZ	TYR	14	30.148	3.930	-10.709	1.00	0.00	C
ATOM	230	OH	TYR	14	29.630	4.736	-11.730	1.00	0.00	O
ATOM	231	HH	TYR	14	29.021	4.273	-12.311	1.00	0.00	H
ATOM	232	CE2	TYR	14	29.598	2.670	-10.433	1.00	0.00	C
ATOM	233	HE2	TYR	14	28.701	2.349	-10.942	1.00	0.00	H
ATOM	234	CD2	TYR	14	30.113	1.820	-9.399	1.00	0.00	C
ATOM	235	HD2	TYR	14	29.725	0.823	-9.259	1.00	0.00	H
ATOM	236	C	TYR	14	29.940	0.481	-6.018	1.00	0.00	C
ATOM	237	O	TYR	14	30.010	-0.751	-6.061	1.00	0.00	O
ATOM	238	N	ARG	15	28.734	1.066	-6.014	1.00	0.00	N
ATOM	239	H	ARG	15	28.685	2.070	-6.112	1.00	0.00	H
ATOM	240	CA	ARG	15	27.571	0.328	-5.555	1.00	0.00	C
ATOM	241	HA	ARG	15	27.923	-0.462	-4.892	1.00	0.00	H
ATOM	242	CB	ARG	15	26.695	1.306	-4.733	1.00	0.00	C
ATOM	243	HB2	ARG	15	25.799	0.801	-4.373	1.00	0.00	H
ATOM	244	HB3	ARG	15	26.402	2.070	-5.453	1.00	0.00	H
ATOM	245	CG	ARG	15	27.353	1.890	-3.451	1.00	0.00	C
ATOM	246	HG2	ARG	15	26.704	2.561	-2.886	1.00	0.00	H
ATOM	247	HG3	ARG	15	28.204	2.481	-3.790	1.00	0.00	H
ATOM	248	CD	ARG	15	27.781	0.917	-2.355	1.00	0.00	C
ATOM	249	HD2	ARG	15	28.520	0.228	-2.765	1.00	0.00	H
ATOM	250	HD3	ARG	15	26.866	0.403	-2.062	1.00	0.00	H
ATOM	251	NE	ARG	15	28.255	1.620	-1.127	1.00	0.00	N
ATOM	252	HE	ARG	15	28.337	2.623	-1.213	1.00	0.00	H
ATOM	253	CZ	ARG	15	28.387	1.050	0.062	1.00	0.00	C

ATOM	254	NH1	ARG	15	28.363	-0.219	0.261	1.00	0.00	N
ATOM	255	HH11	ARG	15	28.475	-0.932	-0.444	1.00	0.00	H
ATOM	256	HH12	ARG	15	28.207	-0.473	1.227	1.00	0.00	H
ATOM	257	NH2	ARG	15	28.466	1.690	1.154	1.00	0.00	N
ATOM	258	HH21	ARG	15	28.639	2.678	1.047	1.00	0.00	H
ATOM	259	HH22	ARG	15	28.547	1.237	2.053	1.00	0.00	H
ATOM	260	C	ARG	15	26.806	-0.470	-6.627	1.00	0.00	C
ATOM	261	O	ARG	15	26.089	-1.414	-6.350	1.00	0.00	O
ATOM	262	N	TYR	16	26.867	-0.069	-7.871	1.00	0.00	N
ATOM	263	H	TYR	16	27.579	0.574	-8.190	1.00	0.00	H
ATOM	264	CA	TYR	16	26.023	-0.701	-8.932	1.00	0.00	C
ATOM	265	HA	TYR	16	25.476	-1.498	-8.429	1.00	0.00	H
ATOM	266	CB	TYR	16	24.942	0.287	-9.454	1.00	0.00	C
ATOM	267	HB2	TYR	16	24.414	0.793	-8.647	1.00	0.00	H
ATOM	268	HB3	TYR	16	24.231	-0.397	-9.919	1.00	0.00	H
ATOM	269	CG	TYR	16	25.401	1.321	-10.493	1.00	0.00	C
ATOM	270	CD1	TYR	16	24.996	1.212	-11.865	1.00	0.00	C
ATOM	271	HD1	TYR	16	24.578	0.297	-12.257	1.00	0.00	H
ATOM	272	CE1	TYR	16	25.267	2.275	-12.717	1.00	0.00	C
ATOM	273	HE1	TYR	16	25.036	2.204	-13.769	1.00	0.00	H
ATOM	274	CZ	TYR	16	25.940	3.446	-12.271	1.00	0.00	C
ATOM	275	OH	TYR	16	26.055	4.558	-13.103	1.00	0.00	O
ATOM	276	HH	TYR	16	26.475	5.343	-12.743	1.00	0.00	H
ATOM	277	CE2	TYR	16	26.351	3.522	-10.936	1.00	0.00	C
ATOM	278	HE2	TYR	16	26.806	4.415	-10.532	1.00	0.00	H
ATOM	279	CD2	TYR	16	26.047	2.489	-10.055	1.00	0.00	C
ATOM	280	HD2	TYR	16	26.251	2.583	-8.998	1.00	0.00	H
ATOM	281	C	TYR	16	26.892	-1.311	-10.056	1.00	0.00	C
ATOM	282	O	TYR	16	27.860	-0.764	-10.504	1.00	0.00	O
ATOM	283	N	NME	17	26.440	-2.511	-10.484	1.00	0.00	N
ATOM	284	H	NME	17	25.636	-2.911	-10.024	1.00	0.00	H
ATOM	285	CH3	NME	17	26.896	-3.359	-11.516	1.00	0.00	C
ATOM	286	HH31	NME	17	26.321	-4.272	-11.361	1.00	0.00	H
ATOM	287	HH32	NME	17	26.725	-3.066	-12.553	1.00	0.00	H
ATOM	288	HH33	NME	17	27.923	-3.683	-11.353	1.00	0.00	H
TER	289		NME	17						
ATOM	289	HH31	ACE	18	19.227	4.833	11.255	1.00	0.00	H
ATOM	290	CH3	ACE	18	18.191	4.801	10.915	1.00	0.00	C
ATOM	291	HH32	ACE	18	17.890	5.796	10.584	1.00	0.00	H
ATOM	292	HH33	ACE	18	17.481	4.415	11.644	1.00	0.00	H
ATOM	293	C	ACE	18	18.267	3.827	9.727	1.00	0.00	C
ATOM	294	O	ACE	18	17.799	4.096	8.598	1.00	0.00	O
ATOM	295	N	LYS	19	18.980	2.749	9.984	1.00	0.00	N
ATOM	296	H	LYS	19	19.509	2.679	10.841	1.00	0.00	H
ATOM	297	CA	LYS	19	19.080	1.550	9.085	1.00	0.00	C
ATOM	298	HA	LYS	19	19.747	1.862	8.282	1.00	0.00	H
ATOM	299	CB	LYS	19	19.753	0.471	9.850	1.00	0.00	C
ATOM	300	HB2	LYS	19	18.973	0.167	10.550	1.00	0.00	H
ATOM	301	HB3	LYS	19	20.625	0.761	10.435	1.00	0.00	H
ATOM	302	CG	LYS	19	20.204	-0.720	8.986	1.00	0.00	C
ATOM	303	HG2	LYS	19	21.178	-0.488	8.558	1.00	0.00	H
ATOM	304	HG3	LYS	19	19.441	-0.934	8.237	1.00	0.00	H
ATOM	305	CD	LYS	19	20.433	-1.999	9.824	1.00	0.00	C
ATOM	306	HD2	LYS	19	21.421	-1.956	10.282	1.00	0.00	H
ATOM	307	HD3	LYS	19	20.461	-2.677	8.971	1.00	0.00	H
ATOM	308	CE	LYS	19	19.409	-2.361	10.812	1.00	0.00	C
ATOM	309	HE2	LYS	19	18.410	-2.100	10.466	1.00	0.00	H
ATOM	310	HE3	LYS	19	19.533	-1.615	11.596	1.00	0.00	H
ATOM	311	NZ	LYS	19	19.410	-3.784	11.171	1.00	0.00	N
ATOM	312	HZ1	LYS	19	18.734	-4.095	11.855	1.00	0.00	H
ATOM	313	HZ2	LYS	19	19.179	-4.212	10.287	1.00	0.00	H
ATOM	314	HZ3	LYS	19	20.288	-4.095	11.562	1.00	0.00	H
ATOM	315	C	LYS	19	17.695	1.045	8.511	1.00	0.00	C

ATOM	316	O	LYS	19	16.687	0.871	9.228	1.00	0.00	O
ATOM	317	N	GLY	20	17.682	0.802	7.229	1.00	0.00	N
ATOM	318	H	GLY	20	18.488	0.918	6.631	1.00	0.00	H
ATOM	319	CA	GLY	20	16.549	0.310	6.459	1.00	0.00	C
ATOM	320	HA2	GLY	20	16.763	-0.552	5.828	1.00	0.00	H
ATOM	321	HA3	GLY	20	15.829	-0.111	7.161	1.00	0.00	H
ATOM	322	C	GLY	20	15.730	1.369	5.775	1.00	0.00	C
ATOM	323	O	GLY	20	15.052	1.060	4.781	1.00	0.00	O
ATOM	324	N	GLU	21	15.941	2.634	6.201	1.00	0.00	N
ATOM	325	H	GLU	21	16.567	2.784	6.978	1.00	0.00	H
ATOM	326	CA	GLU	21	15.303	3.826	5.620	1.00	0.00	C
ATOM	327	HA	GLU	21	14.526	3.491	4.932	1.00	0.00	H
ATOM	328	CB	GLU	21	14.694	4.598	6.773	1.00	0.00	C
ATOM	329	HB2	GLU	21	15.443	4.762	7.548	1.00	0.00	H
ATOM	330	HB3	GLU	21	14.030	3.840	7.189	1.00	0.00	H
ATOM	331	CG	GLU	21	13.987	5.894	6.508	1.00	0.00	C
ATOM	332	HG2	GLU	21	13.251	5.894	5.705	1.00	0.00	H
ATOM	333	HG3	GLU	21	14.760	6.629	6.282	1.00	0.00	H
ATOM	334	CD	GLU	21	13.372	6.456	7.814	1.00	0.00	C
ATOM	335	OE1	GLU	21	13.772	7.574	8.243	1.00	0.00	O
ATOM	336	OE2	GLU	21	12.408	5.778	8.383	1.00	0.00	O
ATOM	337	C	GLU	21	16.220	4.730	4.790	1.00	0.00	C
ATOM	338	O	GLU	21	15.628	5.440	3.944	1.00	0.00	O
ATOM	339	N	ASN	22	17.476	4.813	5.084	1.00	0.00	N
ATOM	340	H	ASN	22	17.894	4.226	5.792	1.00	0.00	H
ATOM	341	CA	ASN	22	18.414	5.803	4.566	1.00	0.00	C
ATOM	342	HA	ASN	22	17.986	6.227	3.657	1.00	0.00	H
ATOM	343	CB	ASN	22	18.435	6.914	5.666	1.00	0.00	C
ATOM	344	HB2	ASN	22	18.599	6.419	6.624	1.00	0.00	H
ATOM	345	HB3	ASN	22	17.497	7.461	5.578	1.00	0.00	H
ATOM	346	CG	ASN	22	19.438	8.042	5.624	1.00	0.00	C
ATOM	347	OD1	ASN	22	20.149	8.301	4.675	1.00	0.00	O
ATOM	348	ND2	ASN	22	19.420	8.860	6.693	1.00	0.00	N
ATOM	349	HD21	ASN	22	20.241	9.443	6.791	1.00	0.00	H
ATOM	350	HD22	ASN	22	18.909	8.570	7.515	1.00	0.00	H
ATOM	351	C	ASN	22	19.816	5.155	4.260	1.00	0.00	C
ATOM	352	O	ASN	22	20.370	5.539	3.269	1.00	0.00	O
ATOM	353	N	PHE	23	20.297	4.156	5.002	1.00	0.00	N
ATOM	354	H	PHE	23	19.905	3.916	5.901	1.00	0.00	H
ATOM	355	CA	PHE	23	21.385	3.311	4.793	1.00	0.00	C
ATOM	356	HA	PHE	23	21.634	3.376	3.734	1.00	0.00	H
ATOM	357	CB	PHE	23	22.562	3.949	5.542	1.00	0.00	C
ATOM	358	HB2	PHE	23	23.017	4.803	5.040	1.00	0.00	H
ATOM	359	HB3	PHE	23	23.309	3.188	5.315	1.00	0.00	H
ATOM	360	CG	PHE	23	22.392	4.341	7.030	1.00	0.00	C
ATOM	361	CD1	PHE	23	22.208	3.345	7.988	1.00	0.00	C
ATOM	362	HD1	PHE	23	22.461	2.314	7.789	1.00	0.00	H
ATOM	363	CE1	PHE	23	22.002	3.710	9.347	1.00	0.00	C
ATOM	364	HE1	PHE	23	21.942	2.921	10.082	1.00	0.00	H
ATOM	365	CZ	PHE	23	22.017	5.045	9.744	1.00	0.00	C
ATOM	366	HZ	PHE	23	21.924	5.360	10.773	1.00	0.00	H
ATOM	367	CE2	PHE	23	22.226	6.019	8.812	1.00	0.00	C
ATOM	368	HE2	PHE	23	22.335	7.034	9.164	1.00	0.00	H
ATOM	369	CD2	PHE	23	22.418	5.676	7.454	1.00	0.00	C
ATOM	370	HD2	PHE	23	22.552	6.466	6.731	1.00	0.00	H
ATOM	371	C	PHE	23	21.040	1.875	5.007	1.00	0.00	C
ATOM	372	O	PHE	23	20.180	1.627	5.892	1.00	0.00	O
ATOM	373	N	THR	24	21.603	0.891	4.307	1.00	0.00	N
ATOM	374	H	THR	24	22.079	1.058	3.432	1.00	0.00	H
ATOM	375	CA	THR	24	21.310	-0.549	4.486	1.00	0.00	C
ATOM	376	HA	THR	24	20.523	-0.608	5.237	1.00	0.00	H
ATOM	377	CB	THR	24	20.767	-1.291	3.326	1.00	0.00	C
ATOM	378	HB	THR	24	20.608	-2.347	3.548	1.00	0.00	H

ATOM	379	CG2	THR	24	19.526	-0.613	2.854	1.00	0.00	C
ATOM	380	HG21	THR	24	19.831	0.263	2.283	1.00	0.00	H
ATOM	381	HG22	THR	24	18.981	-1.358	2.275	1.00	0.00	H
ATOM	382	HG23	THR	24	18.879	-0.297	3.672	1.00	0.00	H
ATOM	383	OG1	THR	24	21.831	-1.255	2.413	1.00	0.00	O
ATOM	384	HG1	THR	24	21.611	-1.838	1.683	1.00	0.00	H
ATOM	385	C	THR	24	22.374	-1.363	5.222	1.00	0.00	C
ATOM	386	O	THR	24	22.118	-2.520	5.563	1.00	0.00	O
ATOM	387	N	GLU	25	23.515	-0.739	5.525	1.00	0.00	N
ATOM	388	H	GLU	25	23.694	0.171	5.126	1.00	0.00	H
ATOM	389	CA	GLU	25	24.614	-1.316	6.285	1.00	0.00	C
ATOM	390	HA	GLU	25	24.601	-2.400	6.175	1.00	0.00	H
ATOM	391	CB	GLU	25	25.950	-0.757	5.813	1.00	0.00	C
ATOM	392	HB2	GLU	25	26.757	-0.980	6.512	1.00	0.00	H
ATOM	393	HB3	GLU	25	25.829	0.321	5.707	1.00	0.00	H
ATOM	394	CG	GLU	25	26.451	-1.312	4.471	1.00	0.00	C
ATOM	395	HG2	GLU	25	25.696	-1.205	3.692	1.00	0.00	H
ATOM	396	HG3	GLU	25	26.661	-2.337	4.779	1.00	0.00	H
ATOM	397	CD	GLU	25	27.619	-0.470	3.849	1.00	0.00	C
ATOM	398	OE1	GLU	25	27.990	0.731	4.000	1.00	0.00	O
ATOM	399	OE2	GLU	25	28.281	-1.039	2.969	1.00	0.00	O
ATOM	400	C	GLU	25	24.604	-0.987	7.752	1.00	0.00	C
ATOM	401	O	GLU	25	24.110	0.074	8.126	1.00	0.00	O
ATOM	402	N	THR	26	25.251	-1.769	8.593	1.00	0.00	N
ATOM	403	H	THR	26	25.689	-2.613	8.249	1.00	0.00	H
ATOM	404	CA	THR	26	25.471	-1.475	10.009	1.00	0.00	C
ATOM	405	HA	THR	26	24.622	-0.964	10.462	1.00	0.00	H
ATOM	406	CB	THR	26	25.782	-2.724	10.815	1.00	0.00	C
ATOM	407	HB	THR	26	26.167	-2.433	11.792	1.00	0.00	H
ATOM	408	CG2	THR	26	24.461	-3.564	10.933	1.00	0.00	C
ATOM	409	HG21	THR	26	24.224	-4.202	10.082	1.00	0.00	H
ATOM	410	HG22	THR	26	24.276	-4.222	11.782	1.00	0.00	H
ATOM	411	HG23	THR	26	23.622	-2.873	11.017	1.00	0.00	H
ATOM	412	OG1	THR	26	26.537	-3.623	9.955	1.00	0.00	O
ATOM	413	HG1	THR	26	27.062	-4.111	10.594	1.00	0.00	H
ATOM	414	C	THR	26	26.638	-0.496	10.169	1.00	0.00	C
ATOM	415	O	THR	26	26.561	0.464	10.886	1.00	0.00	O
ATOM	416	N	ASP	27	27.746	-0.693	9.443	1.00	0.00	N
ATOM	417	H	ASP	27	27.689	-1.410	8.733	1.00	0.00	H
ATOM	418	CA	ASP	27	28.867	0.223	9.289	1.00	0.00	C
ATOM	419	HA	ASP	27	28.823	0.926	10.120	1.00	0.00	H
ATOM	420	CB	ASP	27	30.106	-0.663	9.450	1.00	0.00	C
ATOM	421	HB2	ASP	27	30.130	-1.547	8.813	1.00	0.00	H
ATOM	422	HB3	ASP	27	30.060	-0.961	10.497	1.00	0.00	H
ATOM	423	CG	ASP	27	31.489	0.114	9.209	1.00	0.00	C
ATOM	424	OD1	ASP	27	32.433	-0.420	8.582	1.00	0.00	O
ATOM	425	OD2	ASP	27	31.664	1.143	9.894	1.00	0.00	O
ATOM	426	C	ASP	27	28.973	0.947	7.895	1.00	0.00	C
ATOM	427	O	ASP	27	29.173	0.393	6.810	1.00	0.00	O
ATOM	428	N	ILE	28	28.766	2.230	8.025	1.00	0.00	N
ATOM	429	H	ILE	28	28.589	2.607	8.945	1.00	0.00	H
ATOM	430	CA	ILE	28	28.682	3.206	6.908	1.00	0.00	C
ATOM	431	HA	ILE	28	28.552	2.544	6.050	1.00	0.00	H
ATOM	432	CB	ILE	28	27.443	4.166	7.038	1.00	0.00	C
ATOM	433	HB	ILE	28	27.340	4.813	6.167	1.00	0.00	H
ATOM	434	CG2	ILE	28	26.129	3.371	7.129	1.00	0.00	C
ATOM	435	HG21	ILE	28	25.289	4.001	7.422	1.00	0.00	H
ATOM	436	HG22	ILE	28	25.883	2.905	6.174	1.00	0.00	H
ATOM	437	HG23	ILE	28	26.282	2.652	7.932	1.00	0.00	H
ATOM	438	CG1	ILE	28	27.583	5.030	8.250	1.00	0.00	C
ATOM	439	HG12	ILE	28	28.547	5.536	8.311	1.00	0.00	H
ATOM	440	HG13	ILE	28	27.537	4.309	9.066	1.00	0.00	H
ATOM	441	CD1	ILE	28	26.438	6.077	8.431	1.00	0.00	C

ATOM	442	HD11	ILE	28	25.731	5.827	9.223	1.00	0.00	H
ATOM	443	HD12	ILE	28	26.752	7.111	8.575	1.00	0.00	H
ATOM	444	HD13	ILE	28	25.937	6.156	7.467	1.00	0.00	H
ATOM	445	C	ILE	28	30.004	4.032	6.642	1.00	0.00	C
ATOM	446	O	ILE	28	30.069	4.645	5.613	1.00	0.00	O
ATOM	447	N	LYS	29	30.976	3.993	7.580	1.00	0.00	N
ATOM	448	H	LYS	29	30.844	3.322	8.323	1.00	0.00	H
ATOM	449	CA	LYS	29	32.230	4.627	7.458	1.00	0.00	C
ATOM	450	HA	LYS	29	32.171	5.567	6.908	1.00	0.00	H
ATOM	451	CB	LYS	29	32.699	5.031	8.882	1.00	0.00	C
ATOM	452	HB2	LYS	29	33.334	5.915	8.829	1.00	0.00	H
ATOM	453	HB3	LYS	29	33.249	4.185	9.295	1.00	0.00	H
ATOM	454	CG	LYS	29	31.543	5.500	9.758	1.00	0.00	C
ATOM	455	HG2	LYS	29	30.942	4.625	10.004	1.00	0.00	H
ATOM	456	HG3	LYS	29	31.100	6.169	9.021	1.00	0.00	H
ATOM	457	CD	LYS	29	31.847	6.258	11.026	1.00	0.00	C
ATOM	458	HD2	LYS	29	30.892	6.338	11.545	1.00	0.00	H
ATOM	459	HD3	LYS	29	32.218	7.242	10.741	1.00	0.00	H
ATOM	460	CE	LYS	29	32.806	5.741	12.058	1.00	0.00	C
ATOM	461	HE2	LYS	29	32.930	6.605	12.712	1.00	0.00	H
ATOM	462	HE3	LYS	29	33.678	5.377	11.514	1.00	0.00	H
ATOM	463	NZ	LYS	29	32.285	4.702	12.955	1.00	0.00	N
ATOM	464	HZ1	LYS	29	32.893	4.549	13.747	1.00	0.00	H
ATOM	465	HZ2	LYS	29	31.391	5.011	13.310	1.00	0.00	H
ATOM	466	HZ3	LYS	29	32.113	3.816	12.502	1.00	0.00	H
ATOM	467	C	LYS	29	33.221	3.822	6.577	1.00	0.00	C
ATOM	468	O	LYS	29	32.775	2.789	5.993	1.00	0.00	O
ATOM	469	N	ILE	30	34.506	4.234	6.437	1.00	0.00	N
ATOM	470	H	ILE	30	34.596	5.209	6.682	1.00	0.00	H
ATOM	471	CA	ILE	30	35.582	3.531	5.715	1.00	0.00	C
ATOM	472	HA	ILE	30	35.115	2.622	5.336	1.00	0.00	H
ATOM	473	CB	ILE	30	35.965	4.389	4.459	1.00	0.00	C
ATOM	474	HB	ILE	30	36.832	3.876	4.041	1.00	0.00	H
ATOM	475	CG2	ILE	30	34.884	4.305	3.351	1.00	0.00	C
ATOM	476	HG21	ILE	30	34.049	4.910	3.704	1.00	0.00	H
ATOM	477	HG22	ILE	30	35.133	4.786	2.404	1.00	0.00	H
ATOM	478	HG23	ILE	30	34.470	3.312	3.178	1.00	0.00	H
ATOM	479	CG1	ILE	30	36.462	5.800	4.765	1.00	0.00	C
ATOM	480	HG12	ILE	30	35.652	6.178	5.390	1.00	0.00	H
ATOM	481	HG13	ILE	30	36.510	6.330	3.814	1.00	0.00	H
ATOM	482	CD1	ILE	30	37.685	6.040	5.564	1.00	0.00	C
ATOM	483	HD11	ILE	30	37.732	7.120	5.706	1.00	0.00	H
ATOM	484	HD12	ILE	30	37.644	5.473	6.494	1.00	0.00	H
ATOM	485	HD13	ILE	30	38.524	5.666	4.977	1.00	0.00	H
ATOM	486	C	ILE	30	36.757	3.095	6.649	1.00	0.00	C
ATOM	487	O	ILE	30	37.880	2.701	6.187	1.00	0.00	O
ATOM	488	N	MET	31	36.500	3.020	7.923	1.00	0.00	N
ATOM	489	H	MET	31	35.595	3.316	8.261	1.00	0.00	H
ATOM	490	CA	MET	31	37.402	2.523	8.968	1.00	0.00	C
ATOM	491	HA	MET	31	38.388	2.971	8.848	1.00	0.00	H
ATOM	492	CB	MET	31	36.852	2.920	10.304	1.00	0.00	C
ATOM	493	HB2	MET	31	37.098	2.155	11.041	1.00	0.00	H
ATOM	494	HB3	MET	31	35.764	2.871	10.247	1.00	0.00	H
ATOM	495	CG	MET	31	37.269	4.286	10.866	1.00	0.00	C
ATOM	496	HG2	MET	31	38.355	4.383	10.867	1.00	0.00	H
ATOM	497	HG3	MET	31	36.925	4.230	11.899	1.00	0.00	H
ATOM	498	SD	MET	31	36.401	5.773	10.127	1.00	0.00	S
ATOM	499	CE	MET	31	37.610	7.166	10.674	1.00	0.00	C
ATOM	500	HE1	MET	31	38.629	6.874	10.420	1.00	0.00	H
ATOM	501	HE2	MET	31	37.541	8.025	10.008	1.00	0.00	H
ATOM	502	HE3	MET	31	37.569	7.353	11.748	1.00	0.00	H
ATOM	503	C	MET	31	37.423	0.992	8.718	1.00	0.00	C
ATOM	504	O	MET	31	36.440	0.314	8.555	1.00	0.00	O

```
ATOM    505  N   GLU    32      38.679   0.515   8.461  1.00  0.00           N
ATOM    506  H   GLU    32      39.452   1.162   8.523  1.00  0.00           H
ATOM    507  CA  GLU    32      39.013  -0.941   8.244  1.00  0.00           C
ATOM    508  HA  GLU    32      40.095  -0.985   8.116  1.00  0.00           H
ATOM    509  CB  GLU    32      38.810  -1.816   9.453  1.00  0.00           C
ATOM    510  HB2 GLU    32      38.942  -2.879   9.246  1.00  0.00           H
ATOM    511  HB3 GLU    32      37.764  -1.605   9.675  1.00  0.00           H
ATOM    512  CG  GLU    32      39.760  -1.493  10.652  1.00  0.00           C
ATOM    513  HG2 GLU    32      39.529  -0.478  10.976  1.00  0.00           H
ATOM    514  HG3 GLU    32      40.726  -1.354  10.166  1.00  0.00           H
ATOM    515  CD  GLU    32      39.657  -2.539  11.812  1.00  0.00           C
ATOM    516  OE1 GLU    32      38.507  -2.718  12.348  1.00  0.00           O
ATOM    517  OE2 GLU    32      40.606  -3.183  12.244  1.00  0.00           O
ATOM    518  C   GLU    32      38.438  -1.609   6.995  1.00  0.00           C
ATOM    519  O   GLU    32      38.421  -2.833   6.789  1.00  0.00           O
ATOM    520  N   ARG    33      38.063  -0.732   6.051  1.00  0.00           N
ATOM    521  H   ARG    33      37.943   0.215   6.383  1.00  0.00           H
ATOM    522  CA  ARG    33      37.651  -1.208   4.688  1.00  0.00           C
ATOM    523  HA  ARG    33      38.044  -2.211   4.523  1.00  0.00           H
ATOM    524  CB  ARG    33      36.199  -1.443   4.598  1.00  0.00           C
ATOM    525  HB2 ARG    33      35.897  -1.973   5.502  1.00  0.00           H
ATOM    526  HB3 ARG    33      36.012  -1.941   3.646  1.00  0.00           H
ATOM    527  CG  ARG    33      35.210  -0.311   4.478  1.00  0.00           C
ATOM    528  HG2 ARG    33      35.509   0.392   3.700  1.00  0.00           H
ATOM    529  HG3 ARG    33      35.282   0.075   5.495  1.00  0.00           H
ATOM    530  CD  ARG    33      33.698  -0.681   4.203  1.00  0.00           C
ATOM    531  HD2 ARG    33      33.411  -1.567   4.770  1.00  0.00           H
ATOM    532  HD3 ARG    33      33.705  -0.991   3.157  1.00  0.00           H
ATOM    533  NE  ARG    33      32.658   0.323   4.524  1.00  0.00           N
ATOM    534  HE  ARG    33      32.906   1.178   5.001  1.00  0.00           H
ATOM    535  CZ  ARG    33      31.387   0.267   4.259  1.00  0.00           C
ATOM    536  NH1 ARG    33      30.875  -0.769   3.739  1.00  0.00           N
ATOM    537 HH11 ARG    33      31.327  -1.671   3.788  1.00  0.00           H
ATOM    538 HH12 ARG    33      29.938  -0.799   3.362  1.00  0.00           H
ATOM    539  NH2 ARG    33      30.589   1.326   4.430  1.00  0.00           N
ATOM    540 HH21 ARG    33      31.057   2.119   4.846  1.00  0.00           H
ATOM    541 HH22 ARG    33      29.585   1.230   4.370  1.00  0.00           H
ATOM    542  C   ARG    33      38.253  -0.410   3.518  1.00  0.00           C
ATOM    543  O   ARG    33      37.906  -0.591   2.396  1.00  0.00           O
ATOM    544  N   NME    34      39.102   0.556   3.906  1.00  0.00           N
ATOM    545  H   NME    34      39.321   0.539   4.891  1.00  0.00           H
ATOM    546  CH3 NME    34      39.688   1.559   3.015  1.00  0.00           C
ATOM    547 HH31 NME    34      40.406   2.239   3.477  1.00  0.00           H
ATOM    548 HH32 NME    34      40.342   1.221   2.211  1.00  0.00           H
ATOM    549 HH33 NME    34      38.914   2.124   2.496  1.00  0.00           H
TER     550      NME    34
END
```

Cluster 4:

```
ATOM      1 HH31 ACE     1      36.722  -6.122   2.485  1.00  0.00           H
ATOM      2  CH3 ACE     1      37.229  -6.004   1.526  1.00  0.00           C
ATOM      3 HH32 ACE     1      37.513  -6.922   1.012  1.00  0.00           H
ATOM      4 HH33 ACE     1      38.154  -5.536   1.863  1.00  0.00           H
ATOM      5  C   ACE     1      36.430  -5.181   0.537  1.00  0.00           C
ATOM      6  O   ACE     1      37.001  -4.326  -0.173  1.00  0.00           O
ATOM      7  N   ASN     2      35.103  -5.465   0.408  1.00  0.00           N
ATOM      8  H   ASN     2      34.742  -6.096   1.109  1.00  0.00           H
ATOM      9  CA  ASN     2      34.211  -4.946  -0.753  1.00  0.00           C
ATOM     10  HA  ASN     2      34.845  -4.856  -1.634  1.00  0.00           H
ATOM     11  CB  ASN     2      33.282  -6.067  -1.090  1.00  0.00           C
```

ATOM	12	HB2	ASN	2	32.766	-6.462	-0.215	1.00	0.00	H
ATOM	13	HB3	ASN	2	34.016	-6.828	-1.355	1.00	0.00	H
ATOM	14	CG	ASN	2	32.432	-5.897	-2.253	1.00	0.00	C
ATOM	15	OD1	ASN	2	32.840	-5.789	-3.360	1.00	0.00	O
ATOM	16	ND2	ASN	2	31.129	-5.958	-2.090	1.00	0.00	N
ATOM	17	HD21	ASN	2	30.555	-5.789	-2.903	1.00	0.00	H
ATOM	18	HD22	ASN	2	30.723	-6.287	-1.225	1.00	0.00	H
ATOM	19	C	ASN	2	33.569	-3.529	-0.604	1.00	0.00	C
ATOM	20	O	ASN	2	32.622	-3.119	-1.278	1.00	0.00	O
ATOM	21	N	ASP	3	34.137	-2.741	0.321	1.00	0.00	N
ATOM	22	H	ASP	3	34.904	-3.169	0.823	1.00	0.00	H
ATOM	23	CA	ASP	3	33.615	-1.468	0.786	1.00	0.00	C
ATOM	24	HA	ASP	3	32.649	-1.611	1.269	1.00	0.00	H
ATOM	25	CB	ASP	3	34.605	-0.880	1.802	1.00	0.00	C
ATOM	26	HB2	ASP	3	35.608	-0.868	1.376	1.00	0.00	H
ATOM	27	HB3	ASP	3	34.590	-1.600	2.620	1.00	0.00	H
ATOM	28	CG	ASP	3	34.204	0.394	2.352	1.00	0.00	C
ATOM	29	OD1	ASP	3	34.818	1.452	1.996	1.00	0.00	O
ATOM	30	OD2	ASP	3	33.147	0.416	3.087	1.00	0.00	O
ATOM	31	C	ASP	3	33.409	-0.405	-0.362	1.00	0.00	C
ATOM	32	O	ASP	3	32.557	0.482	-0.273	1.00	0.00	O
ATOM	33	N	TYR	4	34.091	-0.635	-1.490	1.00	0.00	N
ATOM	34	H	TYR	4	34.738	-1.405	-1.404	1.00	0.00	H
ATOM	35	CA	TYR	4	33.959	0.108	-2.765	1.00	0.00	C
ATOM	36	HA	TYR	4	34.018	1.167	-2.512	1.00	0.00	H
ATOM	37	CB	TYR	4	35.266	-0.132	-3.496	1.00	0.00	C
ATOM	38	HB2	TYR	4	35.247	-1.117	-3.963	1.00	0.00	H
ATOM	39	HB3	TYR	4	36.018	-0.174	-2.708	1.00	0.00	H
ATOM	40	CG	TYR	4	35.639	0.864	-4.515	1.00	0.00	C
ATOM	41	CD1	TYR	4	36.261	2.058	-4.201	1.00	0.00	C
ATOM	42	HD1	TYR	4	36.497	2.221	-3.161	1.00	0.00	H
ATOM	43	CE1	TYR	4	36.593	3.007	-5.192	1.00	0.00	C
ATOM	44	HE1	TYR	4	37.083	3.918	-4.882	1.00	0.00	H
ATOM	45	CZ	TYR	4	36.315	2.733	-6.557	1.00	0.00	C
ATOM	46	OH	TYR	4	36.707	3.628	-7.523	1.00	0.00	O
ATOM	47	HH	TYR	4	36.365	3.261	-8.341	1.00	0.00	H
ATOM	48	CE2	TYR	4	35.834	1.462	-6.908	1.00	0.00	C
ATOM	49	HE2	TYR	4	35.804	1.162	-7.945	1.00	0.00	H
ATOM	50	CD2	TYR	4	35.518	0.533	-5.880	1.00	0.00	C
ATOM	51	HD2	TYR	4	35.017	-0.409	-6.057	1.00	0.00	H
ATOM	52	C	TYR	4	32.700	-0.168	-3.683	1.00	0.00	C
ATOM	53	O	TYR	4	32.449	0.553	-4.617	1.00	0.00	O
ATOM	54	N	GLU	5	31.933	-1.297	-3.515	1.00	0.00	N
ATOM	55	H	GLU	5	32.152	-1.942	-2.769	1.00	0.00	H
ATOM	56	CA	GLU	5	30.846	-1.604	-4.440	1.00	0.00	C
ATOM	57	HA	GLU	5	31.327	-1.343	-5.383	1.00	0.00	H
ATOM	58	CB	GLU	5	30.618	-3.076	-4.243	1.00	0.00	C
ATOM	59	HB2	GLU	5	29.882	-3.182	-3.445	1.00	0.00	H
ATOM	60	HB3	GLU	5	31.536	-3.584	-3.950	1.00	0.00	H
ATOM	61	CG	GLU	5	30.090	-3.666	-5.540	1.00	0.00	C
ATOM	62	HG2	GLU	5	30.652	-3.438	-6.446	1.00	0.00	H
ATOM	63	HG3	GLU	5	29.160	-3.126	-5.715	1.00	0.00	H
ATOM	64	CD	GLU	5	29.866	-5.178	-5.484	1.00	0.00	C
ATOM	65	OE1	GLU	5	29.574	-5.723	-4.398	1.00	0.00	O
ATOM	66	OE2	GLU	5	29.979	-5.828	-6.557	1.00	0.00	O
ATOM	67	C	GLU	5	29.634	-0.716	-4.219	1.00	0.00	C
ATOM	68	O	GLU	5	29.485	-0.238	-3.108	1.00	0.00	O
ATOM	69	N	ASP	6	28.783	-0.509	-5.196	1.00	0.00	N
ATOM	70	H	ASP	6	29.109	-0.752	-6.120	1.00	0.00	H
ATOM	71	CA	ASP	6	27.684	0.438	-5.193	1.00	0.00	C
ATOM	72	HA	ASP	6	28.138	1.409	-5.389	1.00	0.00	H
ATOM	73	CB	ASP	6	26.823	0.029	-6.419	1.00	0.00	C
ATOM	74	HB2	ASP	6	26.442	-0.989	-6.345	1.00	0.00	H

ATOM	75	HB3	ASP	6	27.573	0.002	-7.208	1.00	0.00	H
ATOM	76	CG	ASP	6	25.636	1.009	-6.629	1.00	0.00	C
ATOM	77	OD1	ASP	6	24.533	0.688	-6.190	1.00	0.00	O
ATOM	78	OD2	ASP	6	25.663	2.081	-7.320	1.00	0.00	O
ATOM	79	C	ASP	6	26.806	0.375	-3.973	1.00	0.00	C
ATOM	80	O	ASP	6	26.413	1.418	-3.434	1.00	0.00	O
ATOM	81	N	ARG	7	26.569	-0.876	-3.468	1.00	0.00	N
ATOM	82	H	ARG	7	26.932	-1.670	-3.974	1.00	0.00	H
ATOM	83	CA	ARG	7	25.688	-1.068	-2.340	1.00	0.00	C
ATOM	84	HA	ARG	7	24.816	-0.415	-2.376	1.00	0.00	H
ATOM	85	CB	ARG	7	25.130	-2.493	-2.434	1.00	0.00	C
ATOM	86	HB2	ARG	7	24.695	-2.653	-3.420	1.00	0.00	H
ATOM	87	HB3	ARG	7	24.424	-2.566	-1.607	1.00	0.00	H
ATOM	88	CG	ARG	7	26.089	-3.605	-2.321	1.00	0.00	C
ATOM	89	HG2	ARG	7	26.493	-3.673	-1.311	1.00	0.00	H
ATOM	90	HG3	ARG	7	26.911	-3.378	-3.000	1.00	0.00	H
ATOM	91	CD	ARG	7	25.598	-5.064	-2.519	1.00	0.00	C
ATOM	92	HD2	ARG	7	24.858	-5.117	-3.318	1.00	0.00	H
ATOM	93	HD3	ARG	7	25.264	-5.344	-1.520	1.00	0.00	H
ATOM	94	NE	ARG	7	26.659	-5.955	-2.979	1.00	0.00	N
ATOM	95	HE	ARG	7	27.592	-5.575	-3.054	1.00	0.00	H
ATOM	96	CZ	ARG	7	26.690	-7.290	-3.200	1.00	0.00	C
ATOM	97	NH1	ARG	7	25.710	-8.096	-3.002	1.00	0.00	N
ATOM	98	HH11	ARG	7	24.961	-7.753	-2.418	1.00	0.00	H
ATOM	99	HH12	ARG	7	25.800	-9.085	-3.183	1.00	0.00	H
ATOM	100	NH2	ARG	7	27.815	-7.778	-3.668	1.00	0.00	N
ATOM	101	HH21	ARG	7	28.556	-7.157	-3.964	1.00	0.00	H
ATOM	102	HH22	ARG	7	27.928	-8.779	-3.609	1.00	0.00	H
ATOM	103	C	ARG	7	26.247	-0.641	-1.021	1.00	0.00	C
ATOM	104	O	ARG	7	25.511	-0.552	-0.021	1.00	0.00	O
ATOM	105	N	TYR	8	27.550	-0.475	-0.890	1.00	0.00	N
ATOM	106	H	TYR	8	28.159	-0.639	-1.680	1.00	0.00	H
ATOM	107	CA	TYR	8	28.247	-0.162	0.336	1.00	0.00	C
ATOM	108	HA	TYR	8	27.547	0.055	1.143	1.00	0.00	H
ATOM	109	CB	TYR	8	29.011	-1.385	0.694	1.00	0.00	C
ATOM	110	HB2	TYR	8	29.398	-1.236	1.702	1.00	0.00	H
ATOM	111	HB3	TYR	8	29.849	-1.315	0.001	1.00	0.00	H
ATOM	112	CG	TYR	8	28.333	-2.797	0.789	1.00	0.00	C
ATOM	113	CD1	TYR	8	28.762	-3.931	0.087	1.00	0.00	C
ATOM	114	HD1	TYR	8	29.563	-3.889	-0.635	1.00	0.00	H
ATOM	115	CE1	TYR	8	28.154	-5.230	0.345	1.00	0.00	C
ATOM	116	HE1	TYR	8	28.527	-6.095	-0.184	1.00	0.00	H
ATOM	117	CZ	TYR	8	27.077	-5.337	1.271	1.00	0.00	C
ATOM	118	OH	TYR	8	26.364	-6.445	1.564	1.00	0.00	O
ATOM	119	HH	TYR	8	25.817	-6.192	2.312	1.00	0.00	H
ATOM	120	CE2	TYR	8	26.768	-4.221	2.113	1.00	0.00	C
ATOM	121	HE2	TYR	8	25.928	-4.270	2.789	1.00	0.00	H
ATOM	122	CD2	TYR	8	27.398	-2.993	1.837	1.00	0.00	C
ATOM	123	HD2	TYR	8	27.023	-2.135	2.374	1.00	0.00	H
ATOM	124	C	TYR	8	29.096	1.133	0.364	1.00	0.00	C
ATOM	125	O	TYR	8	29.402	1.648	1.398	1.00	0.00	O
ATOM	126	N	TYR	9	29.474	1.598	-0.834	1.00	0.00	N
ATOM	127	H	TYR	9	29.419	0.929	-1.588	1.00	0.00	H
ATOM	128	CA	TYR	9	30.022	2.960	-0.994	1.00	0.00	C
ATOM	129	HA	TYR	9	30.823	3.105	-0.268	1.00	0.00	H
ATOM	130	CB	TYR	9	30.687	2.897	-2.316	1.00	0.00	C
ATOM	131	HB2	TYR	9	29.964	2.661	-3.097	1.00	0.00	H
ATOM	132	HB3	TYR	9	31.371	2.064	-2.155	1.00	0.00	H
ATOM	133	CG	TYR	9	31.501	4.078	-2.760	1.00	0.00	C
ATOM	134	CD1	TYR	9	32.831	4.116	-2.419	1.00	0.00	C
ATOM	135	HD1	TYR	9	33.214	3.269	-1.870	1.00	0.00	H
ATOM	136	CE1	TYR	9	33.662	5.185	-2.852	1.00	0.00	C
ATOM	137	HE1	TYR	9	34.670	5.061	-2.483	1.00	0.00	H

ATOM	138	CZ	TYR	9	33.111	6.192	-3.693	1.00	0.00	C
ATOM	139	OH	TYR	9	33.855	7.246	-4.062	1.00	0.00	O
ATOM	140	HH	TYR	9	34.751	7.280	-3.721	1.00	0.00	H
ATOM	141	CE2	TYR	9	31.692	6.210	-3.948	1.00	0.00	C
ATOM	142	HE2	TYR	9	31.345	7.002	-4.593	1.00	0.00	H
ATOM	143	CD2	TYR	9	30.918	5.117	-3.469	1.00	0.00	C
ATOM	144	HD2	TYR	9	29.855	5.108	-3.660	1.00	0.00	H
ATOM	145	C	TYR	9	29.074	4.097	-0.771	1.00	0.00	C
ATOM	146	O	TYR	9	27.905	3.973	-1.156	1.00	0.00	O
ATOM	147	N	ARG	10	29.501	5.245	-0.282	1.00	0.00	N
ATOM	148	H	ARG	10	30.502	5.350	-0.206	1.00	0.00	H
ATOM	149	CA	ARG	10	28.660	6.348	0.134	1.00	0.00	C
ATOM	150	HA	ARG	10	27.859	6.376	-0.605	1.00	0.00	H
ATOM	151	CB	ARG	10	27.993	6.004	1.454	1.00	0.00	C
ATOM	152	HB2	ARG	10	27.369	5.110	1.437	1.00	0.00	H
ATOM	153	HB3	ARG	10	27.311	6.847	1.562	1.00	0.00	H
ATOM	154	CG	ARG	10	28.754	6.085	2.742	1.00	0.00	C
ATOM	155	HG2	ARG	10	28.094	6.177	3.605	1.00	0.00	H
ATOM	156	HG3	ARG	10	29.454	6.921	2.728	1.00	0.00	H
ATOM	157	CD	ARG	10	29.520	4.816	3.070	1.00	0.00	C
ATOM	158	HD2	ARG	10	28.878	3.948	2.919	1.00	0.00	H
ATOM	159	HD3	ARG	10	29.588	4.912	4.153	1.00	0.00	H
ATOM	160	NE	ARG	10	30.825	4.528	2.407	1.00	0.00	N
ATOM	161	HE	ARG	10	31.247	5.239	1.826	1.00	0.00	H
ATOM	162	CZ	ARG	10	31.646	3.449	2.632	1.00	0.00	C
ATOM	163	NH1	ARG	10	31.446	2.476	3.481	1.00	0.00	N
ATOM	164	HH11	ARG	10	30.556	2.490	3.958	1.00	0.00	H
ATOM	165	HH12	ARG	10	32.018	1.644	3.444	1.00	0.00	H
ATOM	166	NH2	ARG	10	32.679	3.339	1.874	1.00	0.00	N
ATOM	167	HH21	ARG	10	32.866	4.196	1.375	1.00	0.00	H
ATOM	168	HH22	ARG	10	33.266	2.605	2.243	1.00	0.00	H
ATOM	169	C	ARG	10	29.241	7.771	0.014	1.00	0.00	C
ATOM	170	O	ARG	10	28.509	8.714	0.221	1.00	0.00	O
ATOM	171	N	GLU	11	30.487	7.872	-0.460	1.00	0.00	N
ATOM	172	H	GLU	11	31.045	7.042	-0.593	1.00	0.00	H
ATOM	173	CA	GLU	11	31.415	8.991	-0.653	1.00	0.00	C
ATOM	174	HA	GLU	11	31.292	9.652	0.205	1.00	0.00	H
ATOM	175	CB	GLU	11	32.834	8.506	-0.642	1.00	0.00	C
ATOM	176	HB2	GLU	11	33.445	9.376	-0.885	1.00	0.00	H
ATOM	177	HB3	GLU	11	32.884	7.679	-1.352	1.00	0.00	H
ATOM	178	CG	GLU	11	33.251	7.841	0.658	1.00	0.00	C
ATOM	179	HG2	GLU	11	32.642	8.114	1.520	1.00	0.00	H
ATOM	180	HG3	GLU	11	34.307	8.112	0.671	1.00	0.00	H
ATOM	181	CD	GLU	11	33.246	6.321	0.635	1.00	0.00	C
ATOM	182	OE1	GLU	11	34.239	5.606	1.006	1.00	0.00	O
ATOM	183	OE2	GLU	11	32.146	5.760	0.395	1.00	0.00	O
ATOM	184	C	GLU	11	31.030	9.832	-1.926	1.00	0.00	C
ATOM	185	O	GLU	11	31.629	10.882	-2.206	1.00	0.00	O
ATOM	186	N	ASN	12	30.105	9.399	-2.795	1.00	0.00	N
ATOM	187	H	ASN	12	29.617	8.542	-2.579	1.00	0.00	H
ATOM	188	CA	ASN	12	29.630	10.244	-3.926	1.00	0.00	C
ATOM	189	HA	ASN	12	30.532	10.385	-4.521	1.00	0.00	H
ATOM	190	CB	ASN	12	28.684	9.533	-4.873	1.00	0.00	C
ATOM	191	HB2	ASN	12	27.678	9.399	-4.475	1.00	0.00	H
ATOM	192	HB3	ASN	12	29.178	8.581	-5.063	1.00	0.00	H
ATOM	193	CG	ASN	12	28.590	10.141	-6.304	1.00	0.00	C
ATOM	194	OD1	ASN	12	28.526	11.355	-6.534	1.00	0.00	O
ATOM	195	ND2	ASN	12	28.566	9.319	-7.308	1.00	0.00	N
ATOM	196	HD21	ASN	12	28.419	9.697	-8.233	1.00	0.00	H
ATOM	197	HD22	ASN	12	28.659	8.316	-7.248	1.00	0.00	H
ATOM	198	C	ASN	12	29.078	11.580	-3.455	1.00	0.00	C
ATOM	199	O	ASN	12	28.173	11.711	-2.664	1.00	0.00	O
ATOM	200	N	MET	13	29.432	12.685	-4.129	1.00	0.00	N

ATOM	201	H	MET	13	30.208	12.576	-4.767	1.00	0.00	H
ATOM	202	CA	MET	13	29.015	14.087	-3.882	1.00	0.00	C
ATOM	203	HA	MET	13	29.142	14.323	-2.825	1.00	0.00	H
ATOM	204	CB	MET	13	29.977	15.007	-4.663	1.00	0.00	C
ATOM	205	HB2	MET	13	31.008	14.874	-4.336	1.00	0.00	H
ATOM	206	HB3	MET	13	29.588	16.004	-4.454	1.00	0.00	H
ATOM	207	CG	MET	13	30.100	14.757	-6.163	1.00	0.00	C
ATOM	208	HG2	MET	13	29.118	15.029	-6.552	1.00	0.00	H
ATOM	209	HG3	MET	13	30.282	13.697	-6.341	1.00	0.00	H
ATOM	210	SD	MET	13	31.340	15.728	-6.982	1.00	0.00	S
ATOM	211	CE	MET	13	30.930	15.350	-8.776	1.00	0.00	C
ATOM	212	HE1	MET	13	31.022	16.264	-9.363	1.00	0.00	H
ATOM	213	HE2	MET	13	31.529	14.508	-9.124	1.00	0.00	H
ATOM	214	HE3	MET	13	29.894	15.022	-8.857	1.00	0.00	H
ATOM	215	C	MET	13	27.508	14.364	-4.183	1.00	0.00	C
ATOM	216	O	MET	13	26.884	15.209	-3.547	1.00	0.00	O
ATOM	217	N	TYR	14	26.920	13.587	-5.134	1.00	0.00	N
ATOM	218	H	TYR	14	27.482	12.879	-5.584	1.00	0.00	H
ATOM	219	CA	TYR	14	25.530	13.723	-5.621	1.00	0.00	C
ATOM	220	HA	TYR	14	25.174	14.748	-5.711	1.00	0.00	H
ATOM	221	CB	TYR	14	25.499	13.125	-7.019	1.00	0.00	C
ATOM	222	HB2	TYR	14	25.901	12.113	-6.979	1.00	0.00	H
ATOM	223	HB3	TYR	14	26.128	13.720	-7.682	1.00	0.00	H
ATOM	224	CG	TYR	14	24.109	13.035	-7.563	1.00	0.00	C
ATOM	225	CD1	TYR	14	23.210	14.138	-7.823	1.00	0.00	C
ATOM	226	HD1	TYR	14	23.539	15.163	-7.748	1.00	0.00	H
ATOM	227	CE1	TYR	14	21.882	13.842	-8.301	1.00	0.00	C
ATOM	228	HE1	TYR	14	21.247	14.652	-8.626	1.00	0.00	H
ATOM	229	CZ	TYR	14	21.479	12.533	-8.577	1.00	0.00	C
ATOM	230	OH	TYR	14	20.269	12.269	-9.122	1.00	0.00	O
ATOM	231	HH	TYR	14	20.212	11.353	-9.402	1.00	0.00	H
ATOM	232	CE2	TYR	14	22.391	11.506	-8.420	1.00	0.00	C
ATOM	233	HE2	TYR	14	22.106	10.468	-8.502	1.00	0.00	H
ATOM	234	CD2	TYR	14	23.674	11.753	-7.858	1.00	0.00	C
ATOM	235	HD2	TYR	14	24.257	10.870	-7.638	1.00	0.00	H
ATOM	236	C	TYR	14	24.556	12.993	-4.654	1.00	0.00	C
ATOM	237	O	TYR	14	25.013	12.061	-3.976	1.00	0.00	O
ATOM	238	N	ARG	15	23.342	13.482	-4.496	1.00	0.00	N
ATOM	239	H	ARG	15	23.177	14.328	-5.024	1.00	0.00	H
ATOM	240	CA	ARG	15	22.346	13.172	-3.484	1.00	0.00	C
ATOM	241	HA	ARG	15	22.907	12.986	-2.568	1.00	0.00	H
ATOM	242	CB	ARG	15	21.704	14.510	-3.107	1.00	0.00	C
ATOM	243	HB2	ARG	15	22.427	15.264	-2.796	1.00	0.00	H
ATOM	244	HB3	ARG	15	21.069	14.200	-2.277	1.00	0.00	H
ATOM	245	CG	ARG	15	20.722	15.300	-4.001	1.00	0.00	C
ATOM	246	HG2	ARG	15	19.988	14.595	-4.392	1.00	0.00	H
ATOM	247	HG3	ARG	15	21.240	15.801	-4.820	1.00	0.00	H
ATOM	248	CD	ARG	15	19.887	16.392	-3.285	1.00	0.00	C
ATOM	249	HD2	ARG	15	19.570	16.102	-2.283	1.00	0.00	H
ATOM	250	HD3	ARG	15	18.928	16.318	-3.797	1.00	0.00	H
ATOM	251	NE	ARG	15	20.498	17.652	-3.308	1.00	0.00	N
ATOM	252	HE	ARG	15	21.489	17.585	-3.491	1.00	0.00	H
ATOM	253	CZ	ARG	15	19.973	18.896	-3.124	1.00	0.00	C
ATOM	254	NH1	ARG	15	18.723	19.044	-2.999	1.00	0.00	N
ATOM	255	HH11	ARG	15	18.225	18.199	-2.760	1.00	0.00	H
ATOM	256	HH12	ARG	15	18.272	19.916	-2.763	1.00	0.00	H
ATOM	257	NH2	ARG	15	20.790	19.936	-3.197	1.00	0.00	N
ATOM	258	HH21	ARG	15	21.790	19.799	-3.167	1.00	0.00	H
ATOM	259	HH22	ARG	15	20.359	20.831	-3.382	1.00	0.00	H
ATOM	260	C	ARG	15	21.389	12.027	-3.727	1.00	0.00	C
ATOM	261	O	ARG	15	20.536	11.741	-2.871	1.00	0.00	O
ATOM	262	N	TYR	16	21.531	11.405	-4.901	1.00	0.00	N
ATOM	263	H	TYR	16	22.271	11.669	-5.535	1.00	0.00	H

ATOM	264	CA	TYR	16	20.597	10.347	-5.375	1.00	0.00	C
ATOM	265	HA	TYR	16	20.705	10.345	-6.460	1.00	0.00	H
ATOM	266	CB	TYR	16	20.988	8.887	-4.844	1.00	0.00	C
ATOM	267	HB2	TYR	16	20.309	8.090	-5.147	1.00	0.00	H
ATOM	268	HB3	TYR	16	21.040	9.041	-3.766	1.00	0.00	H
ATOM	269	CG	TYR	16	22.334	8.432	-5.399	1.00	0.00	C
ATOM	270	CD1	TYR	16	22.327	7.905	-6.702	1.00	0.00	C
ATOM	271	HD1	TYR	16	21.435	7.474	-7.131	1.00	0.00	H
ATOM	272	CE1	TYR	16	23.550	7.726	-7.328	1.00	0.00	C
ATOM	273	HE1	TYR	16	23.509	7.246	-8.295	1.00	0.00	H
ATOM	274	CZ	TYR	16	24.786	8.033	-6.717	1.00	0.00	C
ATOM	275	OH	TYR	16	25.976	7.603	-7.226	1.00	0.00	O
ATOM	276	HH	TYR	16	25.873	7.281	-8.125	1.00	0.00	H
ATOM	277	CE2	TYR	16	24.779	8.602	-5.445	1.00	0.00	C
ATOM	278	HE2	TYR	16	25.655	8.860	-4.867	1.00	0.00	H
ATOM	279	CD2	TYR	16	23.563	8.862	-4.826	1.00	0.00	C
ATOM	280	HD2	TYR	16	23.556	9.369	-3.872	1.00	0.00	H
ATOM	281	C	TYR	16	19.103	10.688	-5.263	1.00	0.00	C
ATOM	282	O	TYR	16	18.296	9.801	-4.972	1.00	0.00	O
ATOM	283	N	NME	17	18.591	11.991	-5.323	1.00	0.00	N
ATOM	284	H	NME	17	19.286	12.674	-5.587	1.00	0.00	H
ATOM	285	CH3	NME	17	17.205	12.339	-5.369	1.00	0.00	C
ATOM	286	HH31	NME	17	16.604	11.744	-4.680	1.00	0.00	H
ATOM	287	HH32	NME	17	17.174	13.371	-5.017	1.00	0.00	H
ATOM	288	HH33	NME	17	16.916	12.269	-6.417	1.00	0.00	H
TER	289		NME	17						
ATOM	289	HH31	ACE	18	18.330	5.995	0.471	1.00	0.00	H
ATOM	290	CH3	ACE	18	18.857	5.960	-0.483	1.00	0.00	C
ATOM	291	HH32	ACE	18	18.189	5.899	-1.342	1.00	0.00	H
ATOM	292	HH33	ACE	18	19.487	5.071	-0.435	1.00	0.00	H
ATOM	293	C	ACE	18	19.801	7.048	-0.611	1.00	0.00	C
ATOM	294	O	ACE	18	19.436	8.121	-1.104	1.00	0.00	O
ATOM	295	N	LYS	19	21.028	6.757	-0.124	1.00	0.00	N
ATOM	296	H	LYS	19	21.081	5.819	0.248	1.00	0.00	H
ATOM	297	CA	LYS	19	22.254	7.626	-0.029	1.00	0.00	C
ATOM	298	HA	LYS	19	22.913	7.278	0.767	1.00	0.00	H
ATOM	299	CB	LYS	19	23.119	7.673	-1.297	1.00	0.00	C
ATOM	300	HB2	LYS	19	24.086	8.104	-1.038	1.00	0.00	H
ATOM	301	HB3	LYS	19	22.487	8.204	-2.009	1.00	0.00	H
ATOM	302	CG	LYS	19	23.595	6.328	-1.794	1.00	0.00	C
ATOM	303	HG2	LYS	19	24.223	6.379	-2.683	1.00	0.00	H
ATOM	304	HG3	LYS	19	22.699	5.807	-2.132	1.00	0.00	H
ATOM	305	CD	LYS	19	24.397	5.501	-0.794	1.00	0.00	C
ATOM	306	HD2	LYS	19	23.892	5.304	0.152	1.00	0.00	H
ATOM	307	HD3	LYS	19	25.298	6.093	-0.640	1.00	0.00	H
ATOM	308	CE	LYS	19	24.670	4.021	-1.317	1.00	0.00	C
ATOM	309	HE2	LYS	19	23.740	3.475	-1.480	1.00	0.00	H
ATOM	310	HE3	LYS	19	25.255	3.552	-0.526	1.00	0.00	H
ATOM	311	NZ	LYS	19	25.525	3.961	-2.508	1.00	0.00	N
ATOM	312	HZ1	LYS	19	25.734	3.022	-2.817	1.00	0.00	H
ATOM	313	HZ2	LYS	19	25.102	4.429	-3.297	1.00	0.00	H
ATOM	314	HZ3	LYS	19	26.358	4.511	-2.366	1.00	0.00	H
ATOM	315	C	LYS	19	22.031	9.046	0.512	1.00	0.00	C
ATOM	316	O	LYS	19	22.813	9.955	0.157	1.00	0.00	O
ATOM	317	N	GLY	20	21.025	9.129	1.391	1.00	0.00	N
ATOM	318	H	GLY	20	20.528	8.267	1.563	1.00	0.00	H
ATOM	319	CA	GLY	20	20.747	10.348	2.140	1.00	0.00	C
ATOM	320	HA2	GLY	20	20.553	11.113	1.388	1.00	0.00	H
ATOM	321	HA3	GLY	20	19.875	10.183	2.773	1.00	0.00	H
ATOM	322	C	GLY	20	21.855	10.826	3.083	1.00	0.00	C
ATOM	323	O	GLY	20	22.931	10.218	3.045	1.00	0.00	O
ATOM	324	N	GLU	21	21.655	11.924	3.757	1.00	0.00	N
ATOM	325	H	GLU	21	20.768	12.370	3.570	1.00	0.00	H

ATOM	326	CA	GLU	21	22.672	12.630	4.491	1.00	0.00	C
ATOM	327	HA	GLU	21	23.578	12.515	3.897	1.00	0.00	H
ATOM	328	CB	GLU	21	22.503	14.112	4.529	1.00	0.00	C
ATOM	329	HB2	GLU	21	22.173	14.388	3.529	1.00	0.00	H
ATOM	330	HB3	GLU	21	23.489	14.539	4.717	1.00	0.00	H
ATOM	331	CG	GLU	21	21.521	14.663	5.512	1.00	0.00	C
ATOM	332	HG2	GLU	21	21.833	14.392	6.520	1.00	0.00	H
ATOM	333	HG3	GLU	21	20.589	14.121	5.351	1.00	0.00	H
ATOM	334	CD	GLU	21	21.443	16.223	5.433	1.00	0.00	C
ATOM	335	OE1	GLU	21	20.398	16.730	4.943	1.00	0.00	O
ATOM	336	OE2	GLU	21	22.372	16.821	5.990	1.00	0.00	O
ATOM	337	C	GLU	21	23.050	11.953	5.842	1.00	0.00	C
ATOM	338	O	GLU	21	23.996	12.357	6.501	1.00	0.00	O
ATOM	339	N	ASN	22	22.238	10.954	6.297	1.00	0.00	N
ATOM	340	H	ASN	22	21.394	10.735	5.788	1.00	0.00	H
ATOM	341	CA	ASN	22	22.438	10.139	7.485	1.00	0.00	C
ATOM	342	HA	ASN	22	23.250	10.613	8.035	1.00	0.00	H
ATOM	343	CB	ASN	22	21.225	10.111	8.371	1.00	0.00	C
ATOM	344	HB2	ASN	22	20.796	11.109	8.460	1.00	0.00	H
ATOM	345	HB3	ASN	22	21.556	9.728	9.336	1.00	0.00	H
ATOM	346	CG	ASN	22	20.041	9.217	7.872	1.00	0.00	C
ATOM	347	OD1	ASN	22	19.110	9.642	7.233	1.00	0.00	O
ATOM	348	ND2	ASN	22	20.134	7.922	8.250	1.00	0.00	N
ATOM	349	HD21	ASN	22	19.355	7.285	8.159	1.00	0.00	H
ATOM	350	HD22	ASN	22	21.001	7.637	8.684	1.00	0.00	H
ATOM	351	C	ASN	22	23.042	8.726	7.236	1.00	0.00	C
ATOM	352	O	ASN	22	23.159	7.965	8.208	1.00	0.00	O
ATOM	353	N	PHE	23	23.338	8.367	5.969	1.00	0.00	N
ATOM	354	H	PHE	23	23.306	9.117	5.294	1.00	0.00	H
ATOM	355	CA	PHE	23	23.659	6.976	5.620	1.00	0.00	C
ATOM	356	HA	PHE	23	22.853	6.351	6.003	1.00	0.00	H
ATOM	357	CB	PHE	23	23.498	6.793	4.071	1.00	0.00	C
ATOM	358	HB2	PHE	23	24.211	7.444	3.566	1.00	0.00	H
ATOM	359	HB3	PHE	23	22.485	7.085	3.796	1.00	0.00	H
ATOM	360	CG	PHE	23	23.619	5.371	3.555	1.00	0.00	C
ATOM	361	CD1	PHE	23	22.452	4.532	3.438	1.00	0.00	C
ATOM	362	HD1	PHE	23	21.526	5.016	3.713	1.00	0.00	H
ATOM	363	CE1	PHE	23	22.472	3.320	2.731	1.00	0.00	C
ATOM	364	HE1	PHE	23	21.566	2.735	2.672	1.00	0.00	H
ATOM	365	CZ	PHE	23	23.670	2.822	2.289	1.00	0.00	C
ATOM	366	HZ	PHE	23	23.761	1.926	1.692	1.00	0.00	H
ATOM	367	CE2	PHE	23	24.890	3.573	2.490	1.00	0.00	C
ATOM	368	HE2	PHE	23	25.789	3.172	2.046	1.00	0.00	H
ATOM	369	CD2	PHE	23	24.831	4.840	2.998	1.00	0.00	C
ATOM	370	HD2	PHE	23	25.718	5.452	3.065	1.00	0.00	H
ATOM	371	C	PHE	23	24.975	6.451	6.162	1.00	0.00	C
ATOM	372	O	PHE	23	24.968	5.297	6.621	1.00	0.00	O
ATOM	373	N	THR	24	25.999	7.262	6.267	1.00	0.00	N
ATOM	374	H	THR	24	25.792	8.241	6.136	1.00	0.00	H
ATOM	375	CA	THR	24	27.364	6.975	6.829	1.00	0.00	C
ATOM	376	HA	THR	24	27.801	6.185	6.218	1.00	0.00	H
ATOM	377	CB	THR	24	28.276	8.229	6.763	1.00	0.00	C
ATOM	378	HB	THR	24	29.214	8.080	7.298	1.00	0.00	H
ATOM	379	CG2	THR	24	28.417	8.786	5.351	1.00	0.00	C
ATOM	380	HG21	THR	24	27.461	9.122	4.948	1.00	0.00	H
ATOM	381	HG22	THR	24	29.110	9.627	5.391	1.00	0.00	H
ATOM	382	HG23	THR	24	28.792	8.065	4.626	1.00	0.00	H
ATOM	383	OG1	THR	24	27.609	9.298	7.357	1.00	0.00	O
ATOM	384	HG1	THR	24	28.224	10.024	7.227	1.00	0.00	H
ATOM	385	C	THR	24	27.180	6.443	8.256	1.00	0.00	C
ATOM	386	O	THR	24	27.652	5.364	8.524	1.00	0.00	O
ATOM	387	N	GLU	25	26.408	7.109	9.067	1.00	0.00	N
ATOM	388	H	GLU	25	25.995	7.953	8.696	1.00	0.00	H

ATOM	389	CA	GLU	25	26.044	6.673	10.434	1.00	0.00	C
ATOM	390	HA	GLU	25	26.975	6.768	10.993	1.00	0.00	H
ATOM	391	CB	GLU	25	24.921	7.520	11.004	1.00	0.00	C
ATOM	392	HB2	GLU	25	24.032	7.482	10.376	1.00	0.00	H
ATOM	393	HB3	GLU	25	25.407	8.490	10.911	1.00	0.00	H
ATOM	394	CG	GLU	25	24.344	7.413	12.381	1.00	0.00	C
ATOM	395	HG2	GLU	25	23.745	6.508	12.474	1.00	0.00	H
ATOM	396	HG3	GLU	25	23.753	8.328	12.393	1.00	0.00	H
ATOM	397	CD	GLU	25	25.371	7.281	13.490	1.00	0.00	C
ATOM	398	OE1	GLU	25	26.223	8.166	13.741	1.00	0.00	O
ATOM	399	OE2	GLU	25	25.246	6.342	14.341	1.00	0.00	O
ATOM	400	C	GLU	25	25.531	5.248	10.514	1.00	0.00	C
ATOM	401	O	GLU	25	25.928	4.490	11.400	1.00	0.00	O
ATOM	402	N	THR	26	24.644	4.855	9.637	1.00	0.00	N
ATOM	403	H	THR	26	24.276	5.528	8.981	1.00	0.00	H
ATOM	404	CA	THR	26	23.971	3.482	9.694	1.00	0.00	C
ATOM	405	HA	THR	26	24.061	3.109	10.714	1.00	0.00	H
ATOM	406	CB	THR	26	22.434	3.614	9.329	1.00	0.00	C
ATOM	407	HB	THR	26	22.266	3.466	8.263	1.00	0.00	H
ATOM	408	CG2	THR	26	21.690	2.597	10.135	1.00	0.00	C
ATOM	409	HG21	THR	26	21.794	2.741	11.211	1.00	0.00	H
ATOM	410	HG22	THR	26	20.683	2.723	9.739	1.00	0.00	H
ATOM	411	HG23	THR	26	21.852	1.556	9.855	1.00	0.00	H
ATOM	412	OG1	THR	26	22.000	4.905	9.628	1.00	0.00	O
ATOM	413	HG1	THR	26	22.382	5.291	10.420	1.00	0.00	H
ATOM	414	C	THR	26	24.774	2.531	8.801	1.00	0.00	C
ATOM	415	O	THR	26	24.509	1.277	8.883	1.00	0.00	O
ATOM	416	N	ASP	27	25.702	2.959	7.944	1.00	0.00	N
ATOM	417	H	ASP	27	25.816	3.963	7.918	1.00	0.00	H
ATOM	418	CA	ASP	27	26.562	2.006	7.113	1.00	0.00	C
ATOM	419	HA	ASP	27	25.899	1.239	6.714	1.00	0.00	H
ATOM	420	CB	ASP	27	27.229	2.728	5.968	1.00	0.00	C
ATOM	421	HB2	ASP	27	27.890	3.524	6.310	1.00	0.00	H
ATOM	422	HB3	ASP	27	26.359	2.885	5.330	1.00	0.00	H
ATOM	423	CG	ASP	27	28.122	1.772	5.262	1.00	0.00	C
ATOM	424	OD1	ASP	27	29.344	1.996	5.117	1.00	0.00	O
ATOM	425	OD2	ASP	27	27.614	0.710	4.806	1.00	0.00	O
ATOM	426	C	ASP	27	27.633	1.346	7.999	1.00	0.00	C
ATOM	427	O	ASP	27	27.781	0.089	8.095	1.00	0.00	O
ATOM	428	N	ILE	28	28.414	2.203	8.648	1.00	0.00	N
ATOM	429	H	ILE	28	28.230	3.175	8.442	1.00	0.00	H
ATOM	430	CA	ILE	28	29.681	1.845	9.360	1.00	0.00	C
ATOM	431	HA	ILE	28	30.236	1.092	8.800	1.00	0.00	H
ATOM	432	CB	ILE	28	30.483	3.192	9.319	1.00	0.00	C
ATOM	433	HB	ILE	28	31.516	2.870	9.443	1.00	0.00	H
ATOM	434	CG2	ILE	28	30.643	3.885	7.953	1.00	0.00	C
ATOM	435	HG21	ILE	28	31.252	3.162	7.410	1.00	0.00	H
ATOM	436	HG22	ILE	28	29.770	4.146	7.355	1.00	0.00	H
ATOM	437	HG23	ILE	28	31.409	4.643	8.120	1.00	0.00	H
ATOM	438	CG1	ILE	28	30.187	4.140	10.484	1.00	0.00	C
ATOM	439	HG12	ILE	28	30.353	3.742	11.485	1.00	0.00	H
ATOM	440	HG13	ILE	28	29.120	4.363	10.507	1.00	0.00	H
ATOM	441	CD1	ILE	28	30.944	5.447	10.641	1.00	0.00	C
ATOM	442	HD11	ILE	28	30.623	5.794	11.624	1.00	0.00	H
ATOM	443	HD12	ILE	28	32.004	5.213	10.547	1.00	0.00	H
ATOM	444	HD13	ILE	28	30.633	6.188	9.905	1.00	0.00	H
ATOM	445	C	ILE	28	29.570	1.133	10.719	1.00	0.00	C
ATOM	446	O	ILE	28	30.567	0.683	11.237	1.00	0.00	O
ATOM	447	N	LYS	29	28.397	1.052	11.332	1.00	0.00	N
ATOM	448	H	LYS	29	27.614	1.231	10.720	1.00	0.00	H
ATOM	449	CA	LYS	29	28.127	0.397	12.603	1.00	0.00	C
ATOM	450	HA	LYS	29	28.958	0.534	13.297	1.00	0.00	H
ATOM	451	CB	LYS	29	26.907	0.937	13.288	1.00	0.00	C

ATOM	452	HB2	LYS	29	26.625	0.349	14.162	1.00	0.00	H
ATOM	453	HB3	LYS	29	26.119	0.806	12.547	1.00	0.00	H
ATOM	454	CG	LYS	29	27.030	2.419	13.647	1.00	0.00	C
ATOM	455	HG2	LYS	29	26.039	2.797	13.896	1.00	0.00	H
ATOM	456	HG3	LYS	29	27.414	2.832	12.714	1.00	0.00	H
ATOM	457	CD	LYS	29	28.062	2.697	14.697	1.00	0.00	C
ATOM	458	HD2	LYS	29	29.002	2.216	14.428	1.00	0.00	H
ATOM	459	HD3	LYS	29	27.772	2.285	15.663	1.00	0.00	H
ATOM	460	CE	LYS	29	28.521	4.130	14.759	1.00	0.00	C
ATOM	461	HE2	LYS	29	29.031	4.517	13.876	1.00	0.00	H
ATOM	462	HE3	LYS	29	29.340	4.079	15.477	1.00	0.00	H
ATOM	463	NZ	LYS	29	27.549	5.085	15.406	1.00	0.00	N
ATOM	464	HZ1	LYS	29	26.645	5.112	14.957	1.00	0.00	H
ATOM	465	HZ2	LYS	29	27.220	4.849	16.331	1.00	0.00	H
ATOM	466	HZ3	LYS	29	27.904	6.030	15.389	1.00	0.00	H
ATOM	467	C	LYS	29	28.169	-1.117	12.504	1.00	0.00	C
ATOM	468	O	LYS	29	28.306	-1.834	13.512	1.00	0.00	O
ATOM	469	N	ILE	30	27.808	-1.601	11.280	1.00	0.00	N
ATOM	470	H	ILE	30	27.830	-1.017	10.456	1.00	0.00	H
ATOM	471	CA	ILE	30	27.597	-3.018	11.079	1.00	0.00	C
ATOM	472	HA	ILE	30	27.551	-3.476	12.068	1.00	0.00	H
ATOM	473	CB	ILE	30	26.196	-3.275	10.419	1.00	0.00	C
ATOM	474	HB	ILE	30	26.272	-4.345	10.228	1.00	0.00	H
ATOM	475	CG2	ILE	30	25.072	-3.041	11.373	1.00	0.00	C
ATOM	476	HG21	ILE	30	25.261	-3.348	12.403	1.00	0.00	H
ATOM	477	HG22	ILE	30	24.865	-1.971	11.370	1.00	0.00	H
ATOM	478	HG23	ILE	30	24.200	-3.601	11.036	1.00	0.00	H
ATOM	479	CG1	ILE	30	26.095	-2.535	9.082	1.00	0.00	C
ATOM	480	HG12	ILE	30	27.026	-2.643	8.526	1.00	0.00	H
ATOM	481	HG13	ILE	30	26.123	-1.470	9.312	1.00	0.00	H
ATOM	482	CD1	ILE	30	24.933	-2.984	8.178	1.00	0.00	C
ATOM	483	HD11	ILE	30	24.923	-4.069	8.274	1.00	0.00	H
ATOM	484	HD12	ILE	30	23.987	-2.619	8.576	1.00	0.00	H
ATOM	485	HD13	ILE	30	25.128	-2.759	7.129	1.00	0.00	H
ATOM	486	C	ILE	30	28.842	-3.693	10.404	1.00	0.00	C
ATOM	487	O	ILE	30	29.707	-3.016	9.903	1.00	0.00	O
ATOM	488	N	MET	31	28.982	-5.041	10.503	1.00	0.00	N
ATOM	489	H	MET	31	28.281	-5.530	11.041	1.00	0.00	H
ATOM	490	CA	MET	31	30.108	-5.744	9.899	1.00	0.00	C
ATOM	491	HA	MET	31	30.980	-5.100	10.014	1.00	0.00	H
ATOM	492	CB	MET	31	30.297	-7.091	10.699	1.00	0.00	C
ATOM	493	HB2	MET	31	31.249	-7.504	10.363	1.00	0.00	H
ATOM	494	HB3	MET	31	29.472	-7.732	10.390	1.00	0.00	H
ATOM	495	CG	MET	31	30.433	-6.879	12.212	1.00	0.00	C
ATOM	496	HG2	MET	31	29.558	-6.465	12.712	1.00	0.00	H
ATOM	497	HG3	MET	31	31.252	-6.160	12.265	1.00	0.00	H
ATOM	498	SD	MET	31	30.898	-8.364	13.159	1.00	0.00	S
ATOM	499	CE	MET	31	31.183	-7.579	14.763	1.00	0.00	C
ATOM	500	HE1	MET	31	30.323	-6.942	14.967	1.00	0.00	H
ATOM	501	HE2	MET	31	31.165	-8.417	15.461	1.00	0.00	H
ATOM	502	HE3	MET	31	32.131	-7.042	14.760	1.00	0.00	H
ATOM	503	C	MET	31	29.890	-6.083	8.369	1.00	0.00	C
ATOM	504	O	MET	31	30.836	-6.281	7.627	1.00	0.00	O
ATOM	505	N	GLU	32	28.671	-6.102	7.890	1.00	0.00	N
ATOM	506	H	GLU	32	27.894	-5.932	8.513	1.00	0.00	H
ATOM	507	CA	GLU	32	28.376	-6.716	6.596	1.00	0.00	C
ATOM	508	HA	GLU	32	28.785	-7.727	6.608	1.00	0.00	H
ATOM	509	CB	GLU	32	26.882	-6.753	6.543	1.00	0.00	C
ATOM	510	HB2	GLU	32	26.503	-5.750	6.738	1.00	0.00	H
ATOM	511	HB3	GLU	32	26.735	-7.507	7.317	1.00	0.00	H
ATOM	512	CG	GLU	32	26.267	-7.268	5.228	1.00	0.00	C
ATOM	513	HG2	GLU	32	26.838	-6.883	4.383	1.00	0.00	H
ATOM	514	HG3	GLU	32	25.262	-6.858	5.129	1.00	0.00	H

```
ATOM    515  CD  GLU    32      26.301  -8.781   5.012  1.00  0.00           C
ATOM    516  OE1 GLU    32      25.287  -9.315   4.512  1.00  0.00           O
ATOM    517  OE2 GLU    32      27.423  -9.383   5.124  1.00  0.00           O
ATOM    518  C   GLU    32      29.004  -5.887   5.447  1.00  0.00           C
ATOM    519  O   GLU    32      28.754  -4.692   5.282  1.00  0.00           O
ATOM    520  N   ARG    33      29.794  -6.601   4.650  1.00  0.00           N
ATOM    521  H   ARG    33      29.990  -7.560   4.896  1.00  0.00           H
ATOM    522  CA  ARG    33      30.346  -6.160   3.420  1.00  0.00           C
ATOM    523  HA  ARG    33      29.872  -5.228   3.112  1.00  0.00           H
ATOM    524  CB  ARG    33      31.849  -5.838   3.631  1.00  0.00           C
ATOM    525  HB2 ARG    33      32.432  -5.770   2.713  1.00  0.00           H
ATOM    526  HB3 ARG    33      32.230  -6.697   4.184  1.00  0.00           H
ATOM    527  CG  ARG    33      32.331  -4.628   4.442  1.00  0.00           C
ATOM    528  HG2 ARG    33      33.403  -4.817   4.375  1.00  0.00           H
ATOM    529  HG3 ARG    33      31.921  -4.851   5.427  1.00  0.00           H
ATOM    530  CD  ARG    33      32.040  -3.195   3.967  1.00  0.00           C
ATOM    531  HD2 ARG    33      32.088  -3.097   2.882  1.00  0.00           H
ATOM    532  HD3 ARG    33      32.731  -2.509   4.458  1.00  0.00           H
ATOM    533  NE  ARG    33      30.619  -2.880   4.282  1.00  0.00           N
ATOM    534  HE  ARG    33      29.984  -3.654   4.421  1.00  0.00           H
ATOM    535  CZ  ARG    33      30.045  -1.703   4.571  1.00  0.00           C
ATOM    536  NH1 ARG    33      30.602  -0.584   4.419  1.00  0.00           N
ATOM    537 HH11 ARG    33      31.572  -0.510   4.146  1.00  0.00           H
ATOM    538 HH12 ARG    33      30.056   0.256   4.551  1.00  0.00           H
ATOM    539  NH2 ARG    33      28.827  -1.662   4.973  1.00  0.00           N
ATOM    540 HH21 ARG    33      28.293  -2.473   5.253  1.00  0.00           H
ATOM    541 HH22 ARG    33      28.412  -0.743   5.023  1.00  0.00           H
ATOM    542  C   ARG    33      30.056  -7.119   2.205  1.00  0.00           C
ATOM    543  O   ARG    33      30.745  -6.971   1.238  1.00  0.00           O
ATOM    544  N   NME    34      29.110  -8.070   2.308  1.00  0.00           N
ATOM    545  H   NME    34      28.522  -7.990   3.124  1.00  0.00           H
ATOM    546  CH3 NME    34      28.730  -9.007   1.348  1.00  0.00           C
ATOM    547 HH31 NME    34      28.788  -8.586   0.344  1.00  0.00           H
ATOM    548 HH32 NME    34      29.462  -9.813   1.294  1.00  0.00           H
ATOM    549 HH33 NME    34      27.721  -9.354   1.572  1.00  0.00           H
TER     550      NME    34
END
```

Cluster 5:

```
ATOM      1 HH31 ACE     1      19.496  -8.045   0.176  1.00  0.00           H
ATOM      2  CH3 ACE     1      20.327  -8.216  -0.509  1.00  0.00           C
ATOM      3 HH32 ACE     1      19.963  -9.004  -1.168  1.00  0.00           H
ATOM      4 HH33 ACE     1      21.163  -8.467   0.144  1.00  0.00           H
ATOM      5  C   ACE     1      20.500  -7.005  -1.406  1.00  0.00           C
ATOM      6  O   ACE     1      21.543  -6.412  -1.574  1.00  0.00           O
ATOM      7  N   ASN     2      19.390  -6.608  -1.983  1.00  0.00           N
ATOM      8  H   ASN     2      18.570  -7.112  -1.679  1.00  0.00           H
ATOM      9  CA  ASN     2      19.356  -5.437  -2.903  1.00  0.00           C
ATOM     10  HA  ASN     2      19.933  -5.701  -3.790  1.00  0.00           H
ATOM     11  CB  ASN     2      17.918  -5.226  -3.466  1.00  0.00           C
ATOM     12  HB2 ASN     2      17.204  -5.082  -2.655  1.00  0.00           H
ATOM     13  HB3 ASN     2      17.631  -6.135  -3.994  1.00  0.00           H
ATOM     14  CG  ASN     2      17.758  -4.059  -4.433  1.00  0.00           C
ATOM     15  OD1 ASN     2      17.208  -3.053  -4.032  1.00  0.00           O
ATOM     16  ND2 ASN     2      18.281  -4.083  -5.609  1.00  0.00           N
ATOM     17 HD21 ASN     2      18.079  -3.316  -6.234  1.00  0.00           H
ATOM     18 HD22 ASN     2      18.662  -4.970  -5.909  1.00  0.00           H
ATOM     19  C   ASN     2      19.955  -4.120  -2.362  1.00  0.00           C
ATOM     20  O   ASN     2      20.342  -3.238  -3.158  1.00  0.00           O
ATOM     21  N   ASP     3      20.100  -3.867  -1.066  1.00  0.00           N
```

ATOM	22	H	ASP	3	19.798	-4.637	-0.486	1.00	0.00	H
ATOM	23	CA	ASP	3	20.744	-2.688	-0.419	1.00	0.00	C
ATOM	24	HA	ASP	3	20.505	-1.767	-0.951	1.00	0.00	H
ATOM	25	CB	ASP	3	20.073	-2.514	0.972	1.00	0.00	C
ATOM	26	HB2	ASP	3	20.294	-3.358	1.623	1.00	0.00	H
ATOM	27	HB3	ASP	3	19.002	-2.405	0.798	1.00	0.00	H
ATOM	28	CG	ASP	3	20.578	-1.220	1.715	1.00	0.00	C
ATOM	29	OD1	ASP	3	20.458	-0.080	1.211	1.00	0.00	O
ATOM	30	OD2	ASP	3	21.169	-1.425	2.827	1.00	0.00	O
ATOM	31	C	ASP	3	22.265	-2.714	-0.490	1.00	0.00	C
ATOM	32	O	ASP	3	22.927	-1.715	-0.293	1.00	0.00	O
ATOM	33	N	TYR	4	22.859	-3.857	-0.697	1.00	0.00	N
ATOM	34	H	TYR	4	22.411	-4.715	-0.980	1.00	0.00	H
ATOM	35	CA	TYR	4	24.302	-3.923	-0.879	1.00	0.00	C
ATOM	36	HA	TYR	4	24.798	-3.138	-0.310	1.00	0.00	H
ATOM	37	CB	TYR	4	24.896	-5.232	-0.383	1.00	0.00	C
ATOM	38	HB2	TYR	4	25.983	-5.222	-0.461	1.00	0.00	H
ATOM	39	HB3	TYR	4	24.323	-5.907	-1.019	1.00	0.00	H
ATOM	40	CG	TYR	4	24.652	-5.602	1.042	1.00	0.00	C
ATOM	41	CD1	TYR	4	23.844	-6.700	1.424	1.00	0.00	C
ATOM	42	HD1	TYR	4	23.443	-7.363	0.673	1.00	0.00	H
ATOM	43	CE1	TYR	4	23.565	-6.902	2.825	1.00	0.00	C
ATOM	44	HE1	TYR	4	22.931	-7.734	3.094	1.00	0.00	H
ATOM	45	CZ	TYR	4	24.015	-5.986	3.768	1.00	0.00	C
ATOM	46	OH	TYR	4	23.766	-6.137	5.110	1.00	0.00	O
ATOM	47	HH	TYR	4	23.178	-6.862	5.337	1.00	0.00	H
ATOM	48	CE2	TYR	4	24.799	-4.884	3.394	1.00	0.00	C
ATOM	49	HE2	TYR	4	25.197	-4.283	4.197	1.00	0.00	H
ATOM	50	CD2	TYR	4	25.166	-4.748	2.050	1.00	0.00	C
ATOM	51	HD2	TYR	4	25.695	-3.879	1.689	1.00	0.00	H
ATOM	52	C	TYR	4	24.709	-3.550	-2.328	1.00	0.00	C
ATOM	53	O	TYR	4	25.846	-3.110	-2.640	1.00	0.00	O
ATOM	54	N	GLU	5	23.796	-3.663	-3.316	1.00	0.00	N
ATOM	55	H	GLU	5	22.851	-3.942	-3.097	1.00	0.00	H
ATOM	56	CA	GLU	5	23.909	-3.452	-4.773	1.00	0.00	C
ATOM	57	HA	GLU	5	24.953	-3.665	-5.002	1.00	0.00	H
ATOM	58	CB	GLU	5	22.953	-4.351	-5.476	1.00	0.00	C
ATOM	59	HB2	GLU	5	23.004	-4.153	-6.547	1.00	0.00	H
ATOM	60	HB3	GLU	5	21.996	-4.136	-5.002	1.00	0.00	H
ATOM	61	CG	GLU	5	23.341	-5.770	-5.261	1.00	0.00	C
ATOM	62	HG2	GLU	5	23.321	-6.053	-4.209	1.00	0.00	H
ATOM	63	HG3	GLU	5	24.331	-5.848	-5.712	1.00	0.00	H
ATOM	64	CD	GLU	5	22.458	-6.799	-5.916	1.00	0.00	C
ATOM	65	OE1	GLU	5	23.019	-7.927	-6.094	1.00	0.00	O
ATOM	66	OE2	GLU	5	21.232	-6.739	-6.138	1.00	0.00	O
ATOM	67	C	GLU	5	23.841	-2.010	-5.243	1.00	0.00	C
ATOM	68	O	GLU	5	23.985	-1.781	-6.445	1.00	0.00	O
ATOM	69	N	ASP	6	23.550	-1.065	-4.329	1.00	0.00	N
ATOM	70	H	ASP	6	23.542	-1.294	-3.345	1.00	0.00	H
ATOM	71	CA	ASP	6	23.389	0.346	-4.519	1.00	0.00	C
ATOM	72	HA	ASP	6	23.366	0.627	-5.571	1.00	0.00	H
ATOM	73	CB	ASP	6	21.928	0.721	-4.171	1.00	0.00	C
ATOM	74	HB2	ASP	6	21.971	1.078	-3.142	1.00	0.00	H
ATOM	75	HB3	ASP	6	21.233	-0.118	-4.144	1.00	0.00	H
ATOM	76	CG	ASP	6	21.241	1.750	-5.041	1.00	0.00	C
ATOM	77	OD1	ASP	6	21.444	1.727	-6.283	1.00	0.00	O
ATOM	78	OD2	ASP	6	20.572	2.636	-4.403	1.00	0.00	O
ATOM	79	C	ASP	6	24.424	1.279	-3.795	1.00	0.00	C
ATOM	80	O	ASP	6	24.126	2.473	-3.571	1.00	0.00	O
ATOM	81	N	ARG	7	25.621	0.782	-3.449	1.00	0.00	N
ATOM	82	H	ARG	7	25.864	-0.129	-3.813	1.00	0.00	H
ATOM	83	CA	ARG	7	26.671	1.417	-2.621	1.00	0.00	C
ATOM	84	HA	ARG	7	26.267	2.415	-2.449	1.00	0.00	H

ATOM	85	CB	ARG	7	26.798	0.787	-1.199	1.00	0.00	C
ATOM	86	HB2	ARG	7	27.516	1.347	-0.601	1.00	0.00	H
ATOM	87	HB3	ARG	7	27.258	-0.188	-1.365	1.00	0.00	H
ATOM	88	CG	ARG	7	25.567	0.717	-0.291	1.00	0.00	C
ATOM	89	HG2	ARG	7	24.677	0.469	-0.869	1.00	0.00	H
ATOM	90	HG3	ARG	7	25.367	1.738	0.034	1.00	0.00	H
ATOM	91	CD	ARG	7	25.772	-0.240	0.833	1.00	0.00	C
ATOM	92	HD2	ARG	7	26.614	0.040	1.466	1.00	0.00	H
ATOM	93	HD3	ARG	7	26.016	-1.182	0.343	1.00	0.00	H
ATOM	94	NE	ARG	7	24.557	-0.348	1.738	1.00	0.00	N
ATOM	95	HE	ARG	7	23.752	-0.613	1.188	1.00	0.00	H
ATOM	96	CZ	ARG	7	24.487	0.090	2.947	1.00	0.00	C
ATOM	97	NH1	ARG	7	25.438	0.566	3.648	1.00	0.00	N
ATOM	98	HH11	ARG	7	26.256	0.889	3.150	1.00	0.00	H
ATOM	99	HH12	ARG	7	25.298	0.872	4.601	1.00	0.00	H
ATOM	100	NH2	ARG	7	23.358	-0.006	3.543	1.00	0.00	N
ATOM	101	HH21	ARG	7	23.256	0.449	4.439	1.00	0.00	H
ATOM	102	HH22	ARG	7	22.586	-0.541	3.173	1.00	0.00	H
ATOM	103	C	ARG	7	28.119	1.433	-3.205	1.00	0.00	C
ATOM	104	O	ARG	7	28.406	0.545	-3.975	1.00	0.00	O
ATOM	105	N	TYR	8	28.906	2.449	-2.797	1.00	0.00	N
ATOM	106	H	TYR	8	28.657	3.175	-2.139	1.00	0.00	H
ATOM	107	CA	TYR	8	30.300	2.555	-3.240	1.00	0.00	C
ATOM	108	HA	TYR	8	30.531	1.819	-4.011	1.00	0.00	H
ATOM	109	CB	TYR	8	30.486	3.949	-3.841	1.00	0.00	C
ATOM	110	HB2	TYR	8	30.201	3.924	-4.894	1.00	0.00	H
ATOM	111	HB3	TYR	8	31.575	3.936	-3.871	1.00	0.00	H
ATOM	112	CG	TYR	8	29.927	5.258	-3.235	1.00	0.00	C
ATOM	113	CD1	TYR	8	30.785	6.069	-2.464	1.00	0.00	C
ATOM	114	HD1	TYR	8	31.813	5.766	-2.329	1.00	0.00	H
ATOM	115	CE1	TYR	8	30.316	7.206	-1.764	1.00	0.00	C
ATOM	116	HE1	TYR	8	30.961	7.699	-1.052	1.00	0.00	H
ATOM	117	CZ	TYR	8	28.937	7.522	-1.925	1.00	0.00	C
ATOM	118	OH	TYR	8	28.401	8.498	-1.098	1.00	0.00	O
ATOM	119	HH	TYR	8	29.048	8.944	-0.548	1.00	0.00	H
ATOM	120	CE2	TYR	8	28.150	6.786	-2.840	1.00	0.00	C
ATOM	121	HE2	TYR	8	27.159	7.125	-3.105	1.00	0.00	H
ATOM	122	CD2	TYR	8	28.654	5.689	-3.517	1.00	0.00	C
ATOM	123	HD2	TYR	8	28.072	5.217	-4.296	1.00	0.00	H
ATOM	124	C	TYR	8	31.293	2.293	-2.096	1.00	0.00	C
ATOM	125	O	TYR	8	31.007	2.598	-0.982	1.00	0.00	O
ATOM	126	N	TYR	9	32.566	2.019	-2.474	1.00	0.00	N
ATOM	127	H	TYR	9	32.781	1.995	-3.461	1.00	0.00	H
ATOM	128	CA	TYR	9	33.687	2.049	-1.593	1.00	0.00	C
ATOM	129	HA	TYR	9	33.429	1.529	-0.671	1.00	0.00	H
ATOM	130	CB	TYR	9	34.833	1.304	-2.233	1.00	0.00	C
ATOM	131	HB2	TYR	9	35.076	1.556	-3.265	1.00	0.00	H
ATOM	132	HB3	TYR	9	34.347	0.328	-2.246	1.00	0.00	H
ATOM	133	CG	TYR	9	36.175	1.223	-1.476	1.00	0.00	C
ATOM	134	CD1	TYR	9	36.332	0.487	-0.277	1.00	0.00	C
ATOM	135	HD1	TYR	9	35.533	-0.127	0.110	1.00	0.00	H
ATOM	136	CE1	TYR	9	37.529	0.469	0.396	1.00	0.00	C
ATOM	137	HE1	TYR	9	37.540	0.101	1.410	1.00	0.00	H
ATOM	138	CZ	TYR	9	38.654	1.077	-0.127	1.00	0.00	C
ATOM	139	OH	TYR	9	39.867	1.068	0.590	1.00	0.00	O
ATOM	140	HH	TYR	9	40.514	1.651	0.186	1.00	0.00	H
ATOM	141	CE2	TYR	9	38.656	1.583	-1.426	1.00	0.00	C
ATOM	142	HE2	TYR	9	39.520	2.128	-1.776	1.00	0.00	H
ATOM	143	CD2	TYR	9	37.366	1.675	-2.112	1.00	0.00	C
ATOM	144	HD2	TYR	9	37.285	2.126	-3.089	1.00	0.00	H
ATOM	145	C	TYR	9	34.044	3.470	-1.238	1.00	0.00	C
ATOM	146	O	TYR	9	33.946	4.375	-2.086	1.00	0.00	O
ATOM	147	N	ARG	10	34.467	3.759	-0.014	1.00	0.00	N

ATOM	148	H	ARG	10	34.448	3.052	0.706	1.00	0.00	H
ATOM	149	CA	ARG	10	34.580	5.141	0.609	1.00	0.00	C
ATOM	150	HA	ARG	10	33.641	5.622	0.338	1.00	0.00	H
ATOM	151	CB	ARG	10	34.577	5.009	2.192	1.00	0.00	C
ATOM	152	HB2	ARG	10	33.824	4.290	2.512	1.00	0.00	H
ATOM	153	HB3	ARG	10	34.270	6.017	2.469	1.00	0.00	H
ATOM	154	CG	ARG	10	35.889	4.684	2.919	1.00	0.00	C
ATOM	155	HG2	ARG	10	36.663	5.404	2.654	1.00	0.00	H
ATOM	156	HG3	ARG	10	36.224	3.721	2.532	1.00	0.00	H
ATOM	157	CD	ARG	10	35.972	4.665	4.502	1.00	0.00	C
ATOM	158	HD2	ARG	10	35.291	3.985	5.015	1.00	0.00	H
ATOM	159	HD3	ARG	10	35.736	5.699	4.750	1.00	0.00	H
ATOM	160	NE	ARG	10	37.431	4.316	4.826	1.00	0.00	N
ATOM	161	HE	ARG	10	38.076	4.232	4.053	1.00	0.00	H
ATOM	162	CZ	ARG	10	37.811	3.932	6.012	1.00	0.00	C
ATOM	163	NH1	ARG	10	37.015	3.893	7.047	1.00	0.00	N
ATOM	164	HH11	ARG	10	36.015	4.015	7.120	1.00	0.00	H
ATOM	165	HH12	ARG	10	37.410	3.539	7.907	1.00	0.00	H
ATOM	166	NH2	ARG	10	39.041	3.523	6.162	1.00	0.00	N
ATOM	167	HH21	ARG	10	39.428	3.250	7.053	1.00	0.00	H
ATOM	168	HH22	ARG	10	39.610	3.440	5.332	1.00	0.00	H
ATOM	169	C	ARG	10	35.776	6.017	0.188	1.00	0.00	C
ATOM	170	O	ARG	10	35.828	7.147	0.585	1.00	0.00	O
ATOM	171	N	GLU	11	36.762	5.486	-0.574	1.00	0.00	N
ATOM	172	H	GLU	11	36.604	4.560	-0.946	1.00	0.00	H
ATOM	173	CA	GLU	11	37.901	6.141	-1.258	1.00	0.00	C
ATOM	174	HA	GLU	11	38.173	7.118	-0.859	1.00	0.00	H
ATOM	175	CB	GLU	11	39.194	5.331	-1.183	1.00	0.00	C
ATOM	176	HB2	GLU	11	39.957	5.869	-1.745	1.00	0.00	H
ATOM	177	HB3	GLU	11	38.857	4.496	-1.797	1.00	0.00	H
ATOM	178	CG	GLU	11	39.662	4.878	0.241	1.00	0.00	C
ATOM	179	HG2	GLU	11	39.002	4.033	0.434	1.00	0.00	H
ATOM	180	HG3	GLU	11	39.553	5.724	0.920	1.00	0.00	H
ATOM	181	CD	GLU	11	41.176	4.436	0.208	1.00	0.00	C
ATOM	182	OE1	GLU	11	42.091	5.346	0.354	1.00	0.00	O
ATOM	183	OE2	GLU	11	41.524	3.202	0.038	1.00	0.00	O
ATOM	184	C	GLU	11	37.493	6.554	-2.669	1.00	0.00	C
ATOM	185	O	GLU	11	37.570	7.757	-3.023	1.00	0.00	O
ATOM	186	N	ASN	12	36.824	5.624	-3.286	1.00	0.00	N
ATOM	187	H	ASN	12	36.862	4.701	-2.876	1.00	0.00	H
ATOM	188	CA	ASN	12	36.419	5.906	-4.724	1.00	0.00	C
ATOM	189	HA	ASN	12	37.253	6.208	-5.358	1.00	0.00	H
ATOM	190	CB	ASN	12	35.945	4.517	-5.182	1.00	0.00	C
ATOM	191	HB2	ASN	12	35.079	4.176	-4.614	1.00	0.00	H
ATOM	192	HB3	ASN	12	36.835	3.903	-5.052	1.00	0.00	H
ATOM	193	CG	ASN	12	35.554	4.544	-6.725	1.00	0.00	C
ATOM	194	OD1	ASN	12	36.283	5.069	-7.529	1.00	0.00	O
ATOM	195	ND2	ASN	12	34.455	3.893	-7.181	1.00	0.00	N
ATOM	196	HD21	ASN	12	34.238	3.947	-8.167	1.00	0.00	H
ATOM	197	HD22	ASN	12	33.855	3.474	-6.485	1.00	0.00	H
ATOM	198	C	ASN	12	35.405	6.988	-4.859	1.00	0.00	C
ATOM	199	O	ASN	12	35.496	7.725	-5.798	1.00	0.00	O
ATOM	200	N	MET	13	34.492	7.075	-3.892	1.00	0.00	N
ATOM	201	H	MET	13	34.368	6.355	-3.194	1.00	0.00	H
ATOM	202	CA	MET	13	33.642	8.261	-3.789	1.00	0.00	C
ATOM	203	HA	MET	13	34.184	9.114	-4.196	1.00	0.00	H
ATOM	204	CB	MET	13	32.343	8.157	-4.487	1.00	0.00	C
ATOM	205	HB2	MET	13	31.816	7.210	-4.378	1.00	0.00	H
ATOM	206	HB3	MET	13	32.794	8.040	-5.473	1.00	0.00	H
ATOM	207	CG	MET	13	31.348	9.264	-4.437	1.00	0.00	C
ATOM	208	HG2	MET	13	31.900	10.201	-4.517	1.00	0.00	H
ATOM	209	HG3	MET	13	30.913	9.221	-3.439	1.00	0.00	H
ATOM	210	SD	MET	13	30.031	9.097	-5.672	1.00	0.00	S

ATOM	211	CE	MET	13	28.905	10.293	-5.079	1.00	0.00	C
ATOM	212	HE1	MET	13	27.983	10.141	-5.641	1.00	0.00	H
ATOM	213	HE2	MET	13	28.676	10.204	-4.018	1.00	0.00	H
ATOM	214	HE3	MET	13	29.205	11.330	-5.230	1.00	0.00	H
ATOM	215	C	MET	13	33.470	8.695	-2.286	1.00	0.00	C
ATOM	216	O	MET	13	33.496	7.781	-1.463	1.00	0.00	O
ATOM	217	N	TYR	14	33.373	9.949	-1.965	1.00	0.00	N
ATOM	218	H	TYR	14	33.464	10.642	-2.695	1.00	0.00	H
ATOM	219	CA	TYR	14	33.436	10.478	-0.636	1.00	0.00	C
ATOM	220	HA	TYR	14	34.174	9.881	-0.100	1.00	0.00	H
ATOM	221	CB	TYR	14	33.914	11.897	-0.750	1.00	0.00	C
ATOM	222	HB2	TYR	14	33.152	12.429	-1.318	1.00	0.00	H
ATOM	223	HB3	TYR	14	34.771	11.734	-1.404	1.00	0.00	H
ATOM	224	CG	TYR	14	34.394	12.614	0.533	1.00	0.00	C
ATOM	225	CD1	TYR	14	35.427	12.087	1.286	1.00	0.00	C
ATOM	226	HD1	TYR	14	35.972	11.251	0.872	1.00	0.00	H
ATOM	227	CE1	TYR	14	35.929	12.753	2.401	1.00	0.00	C
ATOM	228	HE1	TYR	14	36.884	12.466	2.815	1.00	0.00	H
ATOM	229	CZ	TYR	14	35.256	13.890	2.936	1.00	0.00	C
ATOM	230	OH	TYR	14	35.735	14.527	4.007	1.00	0.00	O
ATOM	231	HH	TYR	14	35.113	15.210	4.268	1.00	0.00	H
ATOM	232	CE2	TYR	14	34.227	14.456	2.181	1.00	0.00	C
ATOM	233	HE2	TYR	14	33.804	15.404	2.480	1.00	0.00	H
ATOM	234	CD2	TYR	14	33.818	13.777	1.006	1.00	0.00	C
ATOM	235	HD2	TYR	14	33.125	14.312	0.373	1.00	0.00	H
ATOM	236	C	TYR	14	32.109	10.428	0.175	1.00	0.00	C
ATOM	237	O	TYR	14	31.071	10.037	-0.332	1.00	0.00	O
ATOM	238	N	ARG	15	32.230	10.724	1.489	1.00	0.00	N
ATOM	239	H	ARG	15	33.136	11.038	1.806	1.00	0.00	H
ATOM	240	CA	ARG	15	31.170	10.642	2.493	1.00	0.00	C
ATOM	241	HA	ARG	15	30.397	10.014	2.051	1.00	0.00	H
ATOM	242	CB	ARG	15	31.596	9.840	3.695	1.00	0.00	C
ATOM	243	HB2	ARG	15	32.176	8.962	3.411	1.00	0.00	H
ATOM	244	HB3	ARG	15	30.671	9.400	4.070	1.00	0.00	H
ATOM	245	CG	ARG	15	32.316	10.650	4.840	1.00	0.00	C
ATOM	246	HG2	ARG	15	31.632	11.264	5.425	1.00	0.00	H
ATOM	247	HG3	ARG	15	32.933	11.305	4.224	1.00	0.00	H
ATOM	248	CD	ARG	15	33.183	9.779	5.834	1.00	0.00	C
ATOM	249	HD2	ARG	15	33.473	10.425	6.662	1.00	0.00	H
ATOM	250	HD3	ARG	15	34.030	9.466	5.223	1.00	0.00	H
ATOM	251	NE	ARG	15	32.369	8.580	6.370	1.00	0.00	N
ATOM	252	HE	ARG	15	31.370	8.710	6.312	1.00	0.00	H
ATOM	253	CZ	ARG	15	32.782	7.440	6.917	1.00	0.00	C
ATOM	254	NH1	ARG	15	33.967	6.862	6.879	1.00	0.00	N
ATOM	255	HH11	ARG	15	34.785	7.393	6.612	1.00	0.00	H
ATOM	256	HH12	ARG	15	34.053	5.891	7.142	1.00	0.00	H
ATOM	257	NH2	ARG	15	31.925	6.710	7.462	1.00	0.00	N
ATOM	258	HH21	ARG	15	32.097	5.727	7.619	1.00	0.00	H
ATOM	259	HH22	ARG	15	30.995	7.002	7.198	1.00	0.00	H
ATOM	260	C	ARG	15	30.360	11.927	2.735	1.00	0.00	C
ATOM	261	O	ARG	15	29.518	11.960	3.636	1.00	0.00	O
ATOM	262	N	TYR	16	30.572	12.959	1.895	1.00	0.00	N
ATOM	263	H	TYR	16	31.141	12.641	1.123	1.00	0.00	H
ATOM	264	CA	TYR	16	29.843	14.290	1.886	1.00	0.00	C
ATOM	265	HA	TYR	16	30.621	15.043	2.012	1.00	0.00	H
ATOM	266	CB	TYR	16	29.126	14.571	0.594	1.00	0.00	C
ATOM	267	HB2	TYR	16	28.528	15.480	0.660	1.00	0.00	H
ATOM	268	HB3	TYR	16	28.495	13.691	0.467	1.00	0.00	H
ATOM	269	CG	TYR	16	29.939	14.605	-0.676	1.00	0.00	C
ATOM	270	CD1	TYR	16	30.014	13.435	-1.520	1.00	0.00	C
ATOM	271	HD1	TYR	16	29.452	12.528	-1.355	1.00	0.00	H
ATOM	272	CE1	TYR	16	30.815	13.615	-2.705	1.00	0.00	C
ATOM	273	HE1	TYR	16	30.799	12.844	-3.461	1.00	0.00	H

ATOM	274	CZ	TYR	16	31.613	14.725	-2.988	1.00	0.00	C
ATOM	275	OH	TYR	16	32.295	14.754	-4.195	1.00	0.00	O
ATOM	276	HH	TYR	16	32.224	13.932	-4.686	1.00	0.00	H
ATOM	277	CE2	TYR	16	31.584	15.755	-2.010	1.00	0.00	C
ATOM	278	HE2	TYR	16	32.134	16.661	-2.220	1.00	0.00	H
ATOM	279	CD2	TYR	16	30.724	15.702	-0.884	1.00	0.00	C
ATOM	280	HD2	TYR	16	30.833	16.490	-0.154	1.00	0.00	H
ATOM	281	C	TYR	16	29.099	14.609	3.176	1.00	0.00	C
ATOM	282	O	TYR	16	27.863	14.584	3.141	1.00	0.00	O
ATOM	283	N	NME	17	29.888	14.864	4.244	1.00	0.00	N
ATOM	284	H	NME	17	30.885	14.872	4.081	1.00	0.00	H
ATOM	285	CH3	NME	17	29.450	14.647	5.626	1.00	0.00	C
ATOM	286	HH31	NME	17	28.384	14.815	5.775	1.00	0.00	H
ATOM	287	HH32	NME	17	29.745	13.656	5.968	1.00	0.00	H
ATOM	288	HH33	NME	17	30.036	15.378	6.184	1.00	0.00	H
TER	289		NME	17						
ATOM	289	HH31	ACE	18	18.946	-5.119	7.898	1.00	0.00	H
ATOM	290	CH3	ACE	18	20.020	-5.298	7.864	1.00	0.00	C
ATOM	291	HH32	ACE	18	20.458	-4.828	6.983	1.00	0.00	H
ATOM	292	HH33	ACE	18	20.247	-6.359	7.972	1.00	0.00	H
ATOM	293	C	ACE	18	20.602	-4.464	8.987	1.00	0.00	C
ATOM	294	O	ACE	18	19.849	-4.044	9.784	1.00	0.00	O
ATOM	295	N	LYS	19	21.923	-4.405	9.095	1.00	0.00	N
ATOM	296	H	LYS	19	22.478	-4.758	8.328	1.00	0.00	H
ATOM	297	CA	LYS	19	22.577	-3.797	10.279	1.00	0.00	C
ATOM	298	HA	LYS	19	22.116	-4.284	11.139	1.00	0.00	H
ATOM	299	CB	LYS	19	24.077	-4.140	10.391	1.00	0.00	C
ATOM	300	HB2	LYS	19	24.633	-3.430	9.779	1.00	0.00	H
ATOM	301	HB3	LYS	19	24.155	-5.154	9.996	1.00	0.00	H
ATOM	302	CG	LYS	19	24.707	-4.190	11.740	1.00	0.00	C
ATOM	303	HG2	LYS	19	24.443	-5.058	12.344	1.00	0.00	H
ATOM	304	HG3	LYS	19	24.466	-3.263	12.259	1.00	0.00	H
ATOM	305	CD	LYS	19	26.246	-4.245	11.608	1.00	0.00	C
ATOM	306	HD2	LYS	19	26.604	-3.589	10.815	1.00	0.00	H
ATOM	307	HD3	LYS	19	26.542	-5.262	11.348	1.00	0.00	H
ATOM	308	CE	LYS	19	26.908	-3.833	12.925	1.00	0.00	C
ATOM	309	HE2	LYS	19	26.627	-4.352	13.841	1.00	0.00	H
ATOM	310	HE3	LYS	19	26.462	-2.849	13.070	1.00	0.00	H
ATOM	311	NZ	LYS	19	28.374	-3.709	12.755	1.00	0.00	N
ATOM	312	HZ1	LYS	19	28.804	-4.619	12.674	1.00	0.00	H
ATOM	313	HZ2	LYS	19	28.756	-3.171	11.991	1.00	0.00	H
ATOM	314	HZ3	LYS	19	28.837	-3.434	13.610	1.00	0.00	H
ATOM	315	C	LYS	19	22.320	-2.250	10.282	1.00	0.00	C
ATOM	316	O	LYS	19	22.107	-1.604	9.206	1.00	0.00	O
ATOM	317	N	GLY	20	22.323	-1.730	11.523	1.00	0.00	N
ATOM	318	H	GLY	20	22.501	-2.367	12.286	1.00	0.00	H
ATOM	319	CA	GLY	20	21.927	-0.352	11.711	1.00	0.00	C
ATOM	320	HA2	GLY	20	21.083	-0.176	11.044	1.00	0.00	H
ATOM	321	HA3	GLY	20	21.734	-0.189	12.772	1.00	0.00	H
ATOM	322	C	GLY	20	22.992	0.580	11.189	1.00	0.00	C
ATOM	323	O	GLY	20	24.145	0.487	11.625	1.00	0.00	O
ATOM	324	N	GLU	21	22.634	1.644	10.490	1.00	0.00	N
ATOM	325	H	GLU	21	21.671	1.731	10.198	1.00	0.00	H
ATOM	326	CA	GLU	21	23.435	2.839	10.184	1.00	0.00	C
ATOM	327	HA	GLU	21	24.389	2.665	10.682	1.00	0.00	H
ATOM	328	CB	GLU	21	23.736	2.907	8.703	1.00	0.00	C
ATOM	329	HB2	GLU	21	24.301	3.787	8.397	1.00	0.00	H
ATOM	330	HB3	GLU	21	22.782	2.954	8.179	1.00	0.00	H
ATOM	331	CG	GLU	21	24.543	1.757	8.183	1.00	0.00	C
ATOM	332	HG2	GLU	21	24.300	0.808	8.660	1.00	0.00	H
ATOM	333	HG3	GLU	21	25.550	1.905	8.571	1.00	0.00	H
ATOM	334	CD	GLU	21	24.606	1.615	6.738	1.00	0.00	C
ATOM	335	OE1	GLU	21	23.617	1.485	5.963	1.00	0.00	O

ATOM	336	OE2	GLU	21	25.779	1.669	6.215	1.00	0.00	O
ATOM	337	C	GLU	21	22.952	4.117	10.921	1.00	0.00	C
ATOM	338	O	GLU	21	21.923	4.690	10.530	1.00	0.00	O
ATOM	339	N	ASN	22	23.614	4.519	12.044	1.00	0.00	N
ATOM	340	H	ASN	22	24.241	3.884	12.516	1.00	0.00	H
ATOM	341	CA	ASN	22	23.224	5.698	12.847	1.00	0.00	C
ATOM	342	HA	ASN	22	22.170	5.901	12.657	1.00	0.00	H
ATOM	343	CB	ASN	22	23.493	5.406	14.350	1.00	0.00	C
ATOM	344	HB2	ASN	22	24.546	5.592	14.560	1.00	0.00	H
ATOM	345	HB3	ASN	22	23.286	4.336	14.329	1.00	0.00	H
ATOM	346	CG	ASN	22	22.706	6.226	15.343	1.00	0.00	C
ATOM	347	OD1	ASN	22	21.702	6.817	15.016	1.00	0.00	O
ATOM	348	ND2	ASN	22	23.114	6.139	16.630	1.00	0.00	N
ATOM	349	HD21	ASN	22	22.427	6.469	17.292	1.00	0.00	H
ATOM	350	HD22	ASN	22	23.997	5.716	16.878	1.00	0.00	H
ATOM	351	C	ASN	22	24.015	6.970	12.371	1.00	0.00	C
ATOM	352	O	ASN	22	24.724	7.586	13.141	1.00	0.00	O
ATOM	353	N	PHE	23	23.763	7.377	11.103	1.00	0.00	N
ATOM	354	H	PHE	23	23.136	6.807	10.555	1.00	0.00	H
ATOM	355	CA	PHE	23	24.529	8.390	10.292	1.00	0.00	C
ATOM	356	HA	PHE	23	25.056	9.096	10.933	1.00	0.00	H
ATOM	357	CB	PHE	23	25.676	7.652	9.511	1.00	0.00	C
ATOM	358	HB2	PHE	23	26.292	8.338	8.930	1.00	0.00	H
ATOM	359	HB3	PHE	23	25.107	6.957	8.894	1.00	0.00	H
ATOM	360	CG	PHE	23	26.594	6.745	10.367	1.00	0.00	C
ATOM	361	CD1	PHE	23	26.638	5.404	10.246	1.00	0.00	C
ATOM	362	HD1	PHE	23	26.001	4.908	9.528	1.00	0.00	H
ATOM	363	CE1	PHE	23	27.333	4.594	11.125	1.00	0.00	C
ATOM	364	HE1	PHE	23	27.262	3.521	11.027	1.00	0.00	H
ATOM	365	CZ	PHE	23	28.130	5.175	12.174	1.00	0.00	C
ATOM	366	HZ	PHE	23	28.625	4.515	12.871	1.00	0.00	H
ATOM	367	CE2	PHE	23	28.127	6.527	12.318	1.00	0.00	C
ATOM	368	HE2	PHE	23	28.711	6.967	13.113	1.00	0.00	H
ATOM	369	CD2	PHE	23	27.418	7.318	11.402	1.00	0.00	C
ATOM	370	HD2	PHE	23	27.391	8.395	11.486	1.00	0.00	H
ATOM	371	C	PHE	23	23.617	9.131	9.357	1.00	0.00	C
ATOM	372	O	PHE	23	22.482	8.716	9.138	1.00	0.00	O
ATOM	373	N	THR	24	24.120	10.240	8.745	1.00	0.00	N
ATOM	374	H	THR	24	25.122	10.319	8.840	1.00	0.00	H
ATOM	375	CA	THR	24	23.461	11.221	7.822	1.00	0.00	C
ATOM	376	HA	THR	24	22.531	11.476	8.330	1.00	0.00	H
ATOM	377	CB	THR	24	24.338	12.515	7.792	1.00	0.00	C
ATOM	378	HB	THR	24	24.507	12.932	8.785	1.00	0.00	H
ATOM	379	CG2	THR	24	25.645	12.264	7.014	1.00	0.00	C
ATOM	380	HG21	THR	24	26.317	13.102	7.196	1.00	0.00	H
ATOM	381	HG22	THR	24	26.111	11.365	7.418	1.00	0.00	H
ATOM	382	HG23	THR	24	25.356	12.234	5.963	1.00	0.00	H
ATOM	383	OG1	THR	24	23.589	13.559	7.082	1.00	0.00	O
ATOM	384	HG1	THR	24	24.202	14.290	6.975	1.00	0.00	H
ATOM	385	C	THR	24	23.173	10.537	6.445	1.00	0.00	C
ATOM	386	O	THR	24	23.668	9.442	6.128	1.00	0.00	O
ATOM	387	N	GLU	25	22.397	11.205	5.587	1.00	0.00	N
ATOM	388	H	GLU	25	22.323	12.210	5.644	1.00	0.00	H
ATOM	389	CA	GLU	25	21.729	10.541	4.440	1.00	0.00	C
ATOM	390	HA	GLU	25	21.370	9.576	4.796	1.00	0.00	H
ATOM	391	CB	GLU	25	20.472	11.367	4.043	1.00	0.00	C
ATOM	392	HB2	GLU	25	20.738	12.361	3.685	1.00	0.00	H
ATOM	393	HB3	GLU	25	19.946	11.553	4.980	1.00	0.00	H
ATOM	394	CG	GLU	25	19.407	10.843	3.058	1.00	0.00	C
ATOM	395	HG2	GLU	25	18.994	9.890	3.389	1.00	0.00	H
ATOM	396	HG3	GLU	25	19.923	10.666	2.114	1.00	0.00	H
ATOM	397	CD	GLU	25	18.174	11.775	2.825	1.00	0.00	C
ATOM	398	OE1	GLU	25	17.382	11.927	3.734	1.00	0.00	O

ATOM	399	OE2	GLU	25	17.986	12.337	1.739	1.00	0.00	O
ATOM	400	C	GLU	25	22.644	10.328	3.210	1.00	0.00	C
ATOM	401	O	GLU	25	22.402	9.347	2.521	1.00	0.00	O
ATOM	402	N	THR	26	23.673	11.134	3.126	1.00	0.00	N
ATOM	403	H	THR	26	23.682	12.027	3.598	1.00	0.00	H
ATOM	404	CA	THR	26	24.851	10.910	2.223	1.00	0.00	C
ATOM	405	HA	THR	26	24.470	10.623	1.243	1.00	0.00	H
ATOM	406	CB	THR	26	25.751	12.120	2.121	1.00	0.00	C
ATOM	407	HB	THR	26	26.720	11.910	1.667	1.00	0.00	H
ATOM	408	CG2	THR	26	24.974	13.186	1.356	1.00	0.00	C
ATOM	409	HG21	THR	26	23.903	13.152	1.556	1.00	0.00	H
ATOM	410	HG22	THR	26	25.341	14.203	1.497	1.00	0.00	H
ATOM	411	HG23	THR	26	25.079	12.935	0.301	1.00	0.00	H
ATOM	412	OG1	THR	26	26.032	12.593	3.445	1.00	0.00	O
ATOM	413	HG1	THR	26	26.684	13.273	3.263	1.00	0.00	H
ATOM	414	C	THR	26	25.736	9.814	2.776	1.00	0.00	C
ATOM	415	O	THR	26	26.339	9.128	1.882	1.00	0.00	O
ATOM	416	N	ASP	27	25.868	9.594	4.119	1.00	0.00	N
ATOM	417	H	ASP	27	25.372	10.176	4.779	1.00	0.00	H
ATOM	418	CA	ASP	27	26.787	8.531	4.543	1.00	0.00	C
ATOM	419	HA	ASP	27	27.688	8.560	3.931	1.00	0.00	H
ATOM	420	CB	ASP	27	27.214	8.763	6.013	1.00	0.00	C
ATOM	421	HB2	ASP	27	26.392	8.611	6.713	1.00	0.00	H
ATOM	422	HB3	ASP	27	27.267	9.845	6.137	1.00	0.00	H
ATOM	423	CG	ASP	27	28.523	8.040	6.371	1.00	0.00	C
ATOM	424	OD1	ASP	27	28.456	6.821	6.465	1.00	0.00	O
ATOM	425	OD2	ASP	27	29.600	8.583	6.681	1.00	0.00	O
ATOM	426	C	ASP	27	26.356	7.086	4.389	1.00	0.00	C
ATOM	427	O	ASP	27	27.086	6.105	4.028	1.00	0.00	O
ATOM	428	N	ILE	28	25.047	6.770	4.665	1.00	0.00	N
ATOM	429	H	ILE	28	24.459	7.545	4.934	1.00	0.00	H
ATOM	430	CA	ILE	28	24.360	5.459	4.522	1.00	0.00	C
ATOM	431	HA	ILE	28	24.981	4.844	5.173	1.00	0.00	H
ATOM	432	CB	ILE	28	22.965	5.387	5.036	1.00	0.00	C
ATOM	433	HB	ILE	28	22.638	4.349	5.077	1.00	0.00	H
ATOM	434	CG2	ILE	28	22.943	5.950	6.508	1.00	0.00	C
ATOM	435	HG21	ILE	28	22.808	7.031	6.521	1.00	0.00	H
ATOM	436	HG22	ILE	28	22.191	5.476	7.138	1.00	0.00	H
ATOM	437	HG23	ILE	28	23.831	5.623	7.051	1.00	0.00	H
ATOM	438	CG1	ILE	28	22.028	6.334	4.133	1.00	0.00	C
ATOM	439	HG12	ILE	28	22.101	6.197	3.054	1.00	0.00	H
ATOM	440	HG13	ILE	28	22.349	7.351	4.357	1.00	0.00	H
ATOM	441	CD1	ILE	28	20.582	6.097	4.583	1.00	0.00	C
ATOM	442	HD11	ILE	28	19.949	6.797	4.038	1.00	0.00	H
ATOM	443	HD12	ILE	28	20.306	5.085	4.286	1.00	0.00	H
ATOM	444	HD13	ILE	28	20.425	6.211	5.655	1.00	0.00	H
ATOM	445	C	ILE	28	24.424	4.736	3.133	1.00	0.00	C
ATOM	446	O	ILE	28	23.982	3.562	2.991	1.00	0.00	O
ATOM	447	N	LYS	29	25.043	5.328	2.145	1.00	0.00	N
ATOM	448	H	LYS	29	25.493	6.204	2.370	1.00	0.00	H
ATOM	449	CA	LYS	29	25.149	4.773	0.805	1.00	0.00	C
ATOM	450	HA	LYS	29	24.460	3.932	0.728	1.00	0.00	H
ATOM	451	CB	LYS	29	24.678	5.779	-0.188	1.00	0.00	C
ATOM	452	HB2	LYS	29	24.703	5.323	-1.178	1.00	0.00	H
ATOM	453	HB3	LYS	29	25.313	6.659	-0.083	1.00	0.00	H
ATOM	454	CG	LYS	29	23.239	6.367	-0.040	1.00	0.00	C
ATOM	455	HG2	LYS	29	23.068	7.117	-0.812	1.00	0.00	H
ATOM	456	HG3	LYS	29	23.201	6.759	0.976	1.00	0.00	H
ATOM	457	CD	LYS	29	22.093	5.320	-0.232	1.00	0.00	C
ATOM	458	HD2	LYS	29	21.115	5.678	0.091	1.00	0.00	H
ATOM	459	HD3	LYS	29	22.410	4.491	0.402	1.00	0.00	H
ATOM	460	CE	LYS	29	22.019	4.689	-1.606	1.00	0.00	C
ATOM	461	HE2	LYS	29	22.915	4.126	-1.866	1.00	0.00	H

ATOM	462	HE3	LYS	29	22.136	5.551	-2.262	1.00	0.00	H
ATOM	463	NZ	LYS	29	20.885	3.760	-1.737	1.00	0.00	N
ATOM	464	HZ1	LYS	29	20.041	4.314	-1.762	1.00	0.00	H
ATOM	465	HZ2	LYS	29	20.984	3.352	-2.655	1.00	0.00	H
ATOM	466	HZ3	LYS	29	20.856	3.053	-1.017	1.00	0.00	H
ATOM	467	C	LYS	29	26.601	4.273	0.559	1.00	0.00	C
ATOM	468	O	LYS	29	27.013	3.975	-0.565	1.00	0.00	O
ATOM	469	N	ILE	30	27.433	4.136	1.621	1.00	0.00	N
ATOM	470	H	ILE	30	27.092	4.398	2.534	1.00	0.00	H
ATOM	471	CA	ILE	30	28.801	3.603	1.453	1.00	0.00	C
ATOM	472	HA	ILE	30	28.978	3.741	0.387	1.00	0.00	H
ATOM	473	CB	ILE	30	29.860	4.525	2.195	1.00	0.00	C
ATOM	474	HB	ILE	30	30.805	4.159	1.793	1.00	0.00	H
ATOM	475	CG2	ILE	30	29.711	5.968	1.746	1.00	0.00	C
ATOM	476	HG21	ILE	30	28.761	6.369	2.098	1.00	0.00	H
ATOM	477	HG22	ILE	30	30.492	6.599	2.171	1.00	0.00	H
ATOM	478	HG23	ILE	30	29.662	6.071	0.663	1.00	0.00	H
ATOM	479	CG1	ILE	30	29.865	4.329	3.721	1.00	0.00	C
ATOM	480	HG12	ILE	30	30.155	3.307	3.963	1.00	0.00	H
ATOM	481	HG13	ILE	30	28.864	4.608	4.050	1.00	0.00	H
ATOM	482	CD1	ILE	30	30.922	5.142	4.451	1.00	0.00	C
ATOM	483	HD11	ILE	30	30.557	6.161	4.581	1.00	0.00	H
ATOM	484	HD12	ILE	30	31.133	4.808	5.465	1.00	0.00	H
ATOM	485	HD13	ILE	30	31.893	4.987	3.981	1.00	0.00	H
ATOM	486	C	ILE	30	28.846	2.095	1.851	1.00	0.00	C
ATOM	487	O	ILE	30	27.987	1.631	2.589	1.00	0.00	O
ATOM	488	N	MET	31	29.915	1.354	1.420	1.00	0.00	N
ATOM	489	H	MET	31	30.517	1.736	0.705	1.00	0.00	H
ATOM	490	CA	MET	31	30.191	-0.072	1.722	1.00	0.00	C
ATOM	491	HA	MET	31	29.227	-0.561	1.857	1.00	0.00	H
ATOM	492	CB	MET	31	30.949	-0.657	0.537	1.00	0.00	C
ATOM	493	HB2	MET	31	32.025	-0.514	0.636	1.00	0.00	H
ATOM	494	HB3	MET	31	30.560	-0.051	-0.281	1.00	0.00	H
ATOM	495	CG	MET	31	30.661	-2.186	0.257	1.00	0.00	C
ATOM	496	HG2	MET	31	29.576	-2.293	0.291	1.00	0.00	H
ATOM	497	HG3	MET	31	31.055	-2.706	1.128	1.00	0.00	H
ATOM	498	SD	MET	31	31.230	-3.003	-1.208	1.00	0.00	S
ATOM	499	CE	MET	31	32.966	-3.419	-0.798	1.00	0.00	C
ATOM	500	HE1	MET	31	33.081	-4.070	0.069	1.00	0.00	H
ATOM	501	HE2	MET	31	33.463	-3.699	-1.727	1.00	0.00	H
ATOM	502	HE3	MET	31	33.512	-2.506	-0.558	1.00	0.00	H
ATOM	503	C	MET	31	30.958	-0.321	3.025	1.00	0.00	C
ATOM	504	O	MET	31	31.358	-1.449	3.307	1.00	0.00	O
ATOM	505	N	GLU	32	31.187	0.690	3.867	1.00	0.00	N
ATOM	506	H	GLU	32	30.750	1.572	3.641	1.00	0.00	H
ATOM	507	CA	GLU	32	31.902	0.635	5.146	1.00	0.00	C
ATOM	508	HA	GLU	32	32.929	0.372	4.893	1.00	0.00	H
ATOM	509	CB	GLU	32	31.983	1.967	5.812	1.00	0.00	C
ATOM	510	HB2	GLU	32	31.002	2.399	6.010	1.00	0.00	H
ATOM	511	HB3	GLU	32	32.549	2.620	5.149	1.00	0.00	H
ATOM	512	CG	GLU	32	32.883	2.003	7.075	1.00	0.00	C
ATOM	513	HG2	GLU	32	33.846	1.513	6.930	1.00	0.00	H
ATOM	514	HG3	GLU	32	32.325	1.467	7.842	1.00	0.00	H
ATOM	515	CD	GLU	32	33.112	3.513	7.469	1.00	0.00	C
ATOM	516	OE1	GLU	32	32.116	4.075	7.997	1.00	0.00	O
ATOM	517	OE2	GLU	32	34.134	4.098	7.055	1.00	0.00	O
ATOM	518	C	GLU	32	31.336	-0.458	6.122	1.00	0.00	C
ATOM	519	O	GLU	32	32.109	-1.113	6.863	1.00	0.00	O
ATOM	520	N	ARG	33	29.982	-0.511	6.166	1.00	0.00	N
ATOM	521	H	ARG	33	29.551	0.077	5.467	1.00	0.00	H
ATOM	522	CA	ARG	33	29.139	-1.475	6.944	1.00	0.00	C
ATOM	523	HA	ARG	33	29.716	-1.901	7.764	1.00	0.00	H
ATOM	524	CB	ARG	33	28.044	-0.645	7.681	1.00	0.00	C

```
ATOM    525  HB2 ARG    33       27.283  -1.341   8.033  1.00  0.00           H
ATOM    526  HB3 ARG    33       27.593  -0.088   6.860  1.00  0.00           H
ATOM    527  CG  ARG    33       28.329   0.270   8.862  1.00  0.00           C
ATOM    528  HG2 ARG    33       29.054  -0.222   9.510  1.00  0.00           H
ATOM    529  HG3 ARG    33       27.397   0.393   9.415  1.00  0.00           H
ATOM    530  CD  ARG    33       28.900   1.688   8.583  1.00  0.00           C
ATOM    531  HD2 ARG    33       29.882   1.616   8.115  1.00  0.00           H
ATOM    532  HD3 ARG    33       28.835   2.139   9.573  1.00  0.00           H
ATOM    533  NE  ARG    33       28.086   2.417   7.616  1.00  0.00           N
ATOM    534  HE  ARG    33       27.349   1.982   7.080  1.00  0.00           H
ATOM    535  CZ  ARG    33       28.166   3.701   7.378  1.00  0.00           C
ATOM    536  NH1 ARG    33       28.936   4.533   7.955  1.00  0.00           N
ATOM    537 HH11 ARG    33       29.679   4.192   8.548  1.00  0.00           H
ATOM    538 HH12 ARG    33       28.862   5.477   7.605  1.00  0.00           H
ATOM    539  NH2 ARG    33       27.230   4.233   6.677  1.00  0.00           N
ATOM    540 HH21 ARG    33       26.516   3.623   6.307  1.00  0.00           H
ATOM    541 HH22 ARG    33       27.318   5.215   6.460  1.00  0.00           H
ATOM    542  C   ARG    33       28.596  -2.586   6.086  1.00  0.00           C
ATOM    543  O   ARG    33       28.294  -2.427   4.883  1.00  0.00           O
ATOM    544  N   NME    34       28.429  -3.815   6.648  1.00  0.00           N
ATOM    545  H   NME    34       28.621  -3.932   7.633  1.00  0.00           H
ATOM    546  CH3 NME    34       27.822  -4.954   6.008  1.00  0.00           C
ATOM    547 HH31 NME    34       27.944  -5.827   6.647  1.00  0.00           H
ATOM    548 HH32 NME    34       26.777  -4.864   5.713  1.00  0.00           H
ATOM    549 HH33 NME    34       28.483  -5.102   5.153  1.00  0.00           H
TER     550      NME    34
END
```

Cluster 6

```
ATOM      1 HH31 ACE     1       21.583 -10.400  -2.267  1.00  0.00           H
ATOM      2  CH3 ACE     1       21.225  -9.419  -2.580  1.00  0.00           C
ATOM      3 HH32 ACE     1       21.437  -8.652  -1.834  1.00  0.00           H
ATOM      4 HH33 ACE     1       20.167  -9.598  -2.766  1.00  0.00           H
ATOM      5  C   ACE     1       21.843  -9.216  -3.972  1.00  0.00           C
ATOM      6  O   ACE     1       22.146 -10.257  -4.606  1.00  0.00           O
ATOM      7  N   ASN     2       21.939  -7.969  -4.464  1.00  0.00           N
ATOM      8  H   ASN     2       21.586  -7.163  -3.969  1.00  0.00           H
ATOM      9  CA  ASN     2       22.535  -7.627  -5.764  1.00  0.00           C
ATOM     10  HA  ASN     2       23.288  -8.395  -5.943  1.00  0.00           H
ATOM     11  CB  ASN     2       21.396  -7.765  -6.743  1.00  0.00           C
ATOM     12  HB2 ASN     2       20.670  -6.972  -6.561  1.00  0.00           H
ATOM     13  HB3 ASN     2       21.104  -8.807  -6.614  1.00  0.00           H
ATOM     14  CG  ASN     2       21.816  -7.576  -8.237  1.00  0.00           C
ATOM     15  OD1 ASN     2       22.953  -7.374  -8.632  1.00  0.00           O
ATOM     16  ND2 ASN     2       20.846  -7.817  -9.118  1.00  0.00           N
ATOM     17 HD21 ASN     2       21.066  -7.829 -10.104  1.00  0.00           H
ATOM     18 HD22 ASN     2       19.920  -8.029  -8.777  1.00  0.00           H
ATOM     19  C   ASN     2       23.168  -6.266  -5.845  1.00  0.00           C
ATOM     20  O   ASN     2       22.598  -5.226  -5.508  1.00  0.00           O
ATOM     21  N   ASP     3       24.437  -6.222  -6.358  1.00  0.00           N
ATOM     22  H   ASP     3       24.934  -6.989  -6.789  1.00  0.00           H
ATOM     23  CA  ASP     3       25.282  -5.033  -6.509  1.00  0.00           C
ATOM     24  HA  ASP     3       25.515  -4.554  -5.559  1.00  0.00           H
ATOM     25  CB  ASP     3       26.671  -5.353  -6.983  1.00  0.00           C
ATOM     26  HB2 ASP     3       27.073  -6.148  -6.357  1.00  0.00           H
ATOM     27  HB3 ASP     3       27.345  -4.503  -6.888  1.00  0.00           H
ATOM     28  CG  ASP     3       26.667  -5.853  -8.418  1.00  0.00           C
ATOM     29  OD1 ASP     3       26.921  -4.987  -9.253  1.00  0.00           O
ATOM     30  OD2 ASP     3       26.539  -7.055  -8.716  1.00  0.00           O
ATOM     31  C   ASP     3       24.731  -3.896  -7.414  1.00  0.00           C
```

ATOM	32	O	ASP	3	25.285	-2.804	-7.321	1.00	0.00	O
ATOM	33	N	TYR	4	23.633	-4.137	-8.171	1.00	0.00	N
ATOM	34	H	TYR	4	23.377	-5.114	-8.163	1.00	0.00	H
ATOM	35	CA	TYR	4	22.819	-3.193	-8.957	1.00	0.00	C
ATOM	36	HA	TYR	4	23.292	-2.839	-9.873	1.00	0.00	H
ATOM	37	CB	TYR	4	21.686	-4.029	-9.650	1.00	0.00	C
ATOM	38	HB2	TYR	4	20.928	-4.301	-8.916	1.00	0.00	H
ATOM	39	HB3	TYR	4	22.164	-4.908	-10.081	1.00	0.00	H
ATOM	40	CG	TYR	4	21.001	-3.400	-10.801	1.00	0.00	C
ATOM	41	CD1	TYR	4	21.482	-3.701	-12.086	1.00	0.00	C
ATOM	42	HD1	TYR	4	22.421	-4.229	-12.163	1.00	0.00	H
ATOM	43	CE1	TYR	4	20.773	-3.142	-13.178	1.00	0.00	C
ATOM	44	HE1	TYR	4	21.128	-3.436	-14.155	1.00	0.00	H
ATOM	45	CZ	TYR	4	19.586	-2.406	-13.023	1.00	0.00	C
ATOM	46	OH	TYR	4	18.853	-1.974	-14.116	1.00	0.00	O
ATOM	47	HH	TYR	4	19.354	-2.181	-14.907	1.00	0.00	H
ATOM	48	CE2	TYR	4	19.174	-2.020	-11.734	1.00	0.00	C
ATOM	49	HE2	TYR	4	18.349	-1.326	-11.678	1.00	0.00	H
ATOM	50	CD2	TYR	4	19.855	-2.541	-10.573	1.00	0.00	C
ATOM	51	HD2	TYR	4	19.466	-2.325	-9.588	1.00	0.00	H
ATOM	52	C	TYR	4	22.269	-2.096	-8.046	1.00	0.00	C
ATOM	53	O	TYR	4	22.052	-1.018	-8.574	1.00	0.00	O
ATOM	54	N	GLU	5	21.985	-2.421	-6.780	1.00	0.00	N
ATOM	55	H	GLU	5	22.092	-3.382	-6.486	1.00	0.00	H
ATOM	56	CA	GLU	5	21.608	-1.494	-5.723	1.00	0.00	C
ATOM	57	HA	GLU	5	21.180	-0.571	-6.118	1.00	0.00	H
ATOM	58	CB	GLU	5	20.408	-2.100	-4.952	1.00	0.00	C
ATOM	59	HB2	GLU	5	19.983	-1.416	-4.217	1.00	0.00	H
ATOM	60	HB3	GLU	5	20.789	-2.929	-4.357	1.00	0.00	H
ATOM	61	CG	GLU	5	19.163	-2.580	-5.718	1.00	0.00	C
ATOM	62	HG2	GLU	5	19.363	-3.381	-6.430	1.00	0.00	H
ATOM	63	HG3	GLU	5	19.012	-1.803	-6.468	1.00	0.00	H
ATOM	64	CD	GLU	5	17.854	-2.817	-4.838	1.00	0.00	C
ATOM	65	OE1	GLU	5	17.683	-3.838	-4.123	1.00	0.00	O
ATOM	66	OE2	GLU	5	16.983	-1.860	-4.953	1.00	0.00	O
ATOM	67	C	GLU	5	22.675	-0.854	-4.803	1.00	0.00	C
ATOM	68	O	GLU	5	22.479	0.291	-4.465	1.00	0.00	O
ATOM	69	N	ASP	6	23.717	-1.661	-4.463	1.00	0.00	N
ATOM	70	H	ASP	6	23.743	-2.512	-5.008	1.00	0.00	H
ATOM	71	CA	ASP	6	24.910	-1.392	-3.668	1.00	0.00	C
ATOM	72	HA	ASP	6	24.522	-1.105	-2.692	1.00	0.00	H
ATOM	73	CB	ASP	6	25.695	-2.695	-3.461	1.00	0.00	C
ATOM	74	HB2	ASP	6	26.142	-2.973	-4.417	1.00	0.00	H
ATOM	75	HB3	ASP	6	24.902	-3.399	-3.212	1.00	0.00	H
ATOM	76	CG	ASP	6	26.815	-2.578	-2.398	1.00	0.00	C
ATOM	77	OD1	ASP	6	26.603	-2.166	-1.205	1.00	0.00	O
ATOM	78	OD2	ASP	6	27.966	-2.840	-2.829	1.00	0.00	O
ATOM	79	C	ASP	6	25.771	-0.292	-4.332	1.00	0.00	C
ATOM	80	O	ASP	6	26.220	0.675	-3.691	1.00	0.00	O
ATOM	81	N	ARG	7	25.931	-0.318	-5.674	1.00	0.00	N
ATOM	82	H	ARG	7	25.471	-1.110	-6.101	1.00	0.00	H
ATOM	83	CA	ARG	7	26.794	0.547	-6.578	1.00	0.00	C
ATOM	84	HA	ARG	7	27.822	0.273	-6.339	1.00	0.00	H
ATOM	85	CB	ARG	7	26.616	0.156	-8.045	1.00	0.00	C
ATOM	86	HB2	ARG	7	26.917	-0.884	-8.168	1.00	0.00	H
ATOM	87	HB3	ARG	7	27.366	0.762	-8.553	1.00	0.00	H
ATOM	88	CG	ARG	7	25.223	0.176	-8.722	1.00	0.00	C
ATOM	89	HG2	ARG	7	24.896	1.216	-8.693	1.00	0.00	H
ATOM	90	HG3	ARG	7	24.534	-0.418	-8.121	1.00	0.00	H
ATOM	91	CD	ARG	7	25.279	-0.345	-10.146	1.00	0.00	C
ATOM	92	HD2	ARG	7	25.917	0.288	-10.761	1.00	0.00	H
ATOM	93	HD3	ARG	7	24.233	-0.368	-10.453	1.00	0.00	H
ATOM	94	NE	ARG	7	25.943	-1.708	-10.279	1.00	0.00	N

ATOM	95	HE	ARG	7	25.670	-2.385	-9.581	1.00	0.00	H
ATOM	96	CZ	ARG	7	26.730	-2.040	-11.282	1.00	0.00	C
ATOM	97	NH1	ARG	7	27.036	-1.278	-12.274	1.00	0.00	N
ATOM	98	HH11	ARG	7	26.813	-0.294	-12.222	1.00	0.00	H
ATOM	99	HH12	ARG	7	27.780	-1.616	-12.867	1.00	0.00	H
ATOM	100	NH2	ARG	7	27.277	-3.240	-11.124	1.00	0.00	N
ATOM	101	HH21	ARG	7	27.058	-3.775	-10.295	1.00	0.00	H
ATOM	102	HH22	ARG	7	28.074	-3.570	-11.647	1.00	0.00	H
ATOM	103	C	ARG	7	26.679	2.008	-6.392	1.00	0.00	C
ATOM	104	O	ARG	7	27.665	2.653	-6.594	1.00	0.00	O
ATOM	105	N	TYR	8	25.519	2.571	-6.145	1.00	0.00	N
ATOM	106	H	TYR	8	24.766	1.901	-6.189	1.00	0.00	H
ATOM	107	CA	TYR	8	25.248	3.961	-5.893	1.00	0.00	C
ATOM	108	HA	TYR	8	25.568	4.607	-6.711	1.00	0.00	H
ATOM	109	CB	TYR	8	23.713	4.268	-5.910	1.00	0.00	C
ATOM	110	HB2	TYR	8	23.471	5.216	-5.429	1.00	0.00	H
ATOM	111	HB3	TYR	8	23.159	3.545	-5.311	1.00	0.00	H
ATOM	112	CG	TYR	8	23.111	4.305	-7.332	1.00	0.00	C
ATOM	113	CD1	TYR	8	23.171	5.477	-8.119	1.00	0.00	C
ATOM	114	HD1	TYR	8	23.730	6.335	-7.774	1.00	0.00	H
ATOM	115	CE1	TYR	8	22.656	5.533	-9.435	1.00	0.00	C
ATOM	116	HE1	TYR	8	22.919	6.330	-10.115	1.00	0.00	H
ATOM	117	CZ	TYR	8	21.920	4.448	-9.839	1.00	0.00	C
ATOM	118	OH	TYR	8	21.489	4.370	-11.080	1.00	0.00	O
ATOM	119	HH	TYR	8	21.760	5.137	-11.590	1.00	0.00	H
ATOM	120	CE2	TYR	8	21.955	3.236	-9.133	1.00	0.00	C
ATOM	121	HE2	TYR	8	21.360	2.420	-9.515	1.00	0.00	H
ATOM	122	CD2	TYR	8	22.429	3.191	-7.844	1.00	0.00	C
ATOM	123	HD2	TYR	8	22.386	2.272	-7.278	1.00	0.00	H
ATOM	124	C	TYR	8	25.768	4.455	-4.523	1.00	0.00	C
ATOM	125	O	TYR	8	26.517	5.410	-4.443	1.00	0.00	O
ATOM	126	N	TYR	9	25.483	3.611	-3.485	1.00	0.00	N
ATOM	127	H	TYR	9	25.047	2.722	-3.684	1.00	0.00	H
ATOM	128	CA	TYR	9	25.633	3.818	-2.043	1.00	0.00	C
ATOM	129	HA	TYR	9	25.571	4.901	-1.933	1.00	0.00	H
ATOM	130	CB	TYR	9	24.369	3.301	-1.384	1.00	0.00	C
ATOM	131	HB2	TYR	9	24.382	3.629	-0.344	1.00	0.00	H
ATOM	132	HB3	TYR	9	24.565	2.241	-1.542	1.00	0.00	H
ATOM	133	CG	TYR	9	23.089	3.683	-2.006	1.00	0.00	C
ATOM	134	CD1	TYR	9	22.608	4.999	-1.951	1.00	0.00	C
ATOM	135	HD1	TYR	9	23.332	5.727	-1.617	1.00	0.00	H
ATOM	136	CE1	TYR	9	21.337	5.299	-2.444	1.00	0.00	C
ATOM	137	HE1	TYR	9	20.950	6.306	-2.406	1.00	0.00	H
ATOM	138	CZ	TYR	9	20.461	4.269	-2.831	1.00	0.00	C
ATOM	139	OH	TYR	9	19.230	4.597	-3.338	1.00	0.00	O
ATOM	140	HH	TYR	9	18.928	5.485	-3.138	1.00	0.00	H
ATOM	141	CE2	TYR	9	20.892	2.926	-2.685	1.00	0.00	C
ATOM	142	HE2	TYR	9	20.307	2.074	-2.999	1.00	0.00	H
ATOM	143	CD2	TYR	9	22.184	2.656	-2.314	1.00	0.00	C
ATOM	144	HD2	TYR	9	22.496	1.624	-2.247	1.00	0.00	H
ATOM	145	C	TYR	9	26.996	3.439	-1.456	1.00	0.00	C
ATOM	146	O	TYR	9	27.398	4.134	-0.466	1.00	0.00	O
ATOM	147	N	ARG	10	27.783	2.513	-2.061	1.00	0.00	N
ATOM	148	H	ARG	10	27.277	1.881	-2.663	1.00	0.00	H
ATOM	149	CA	ARG	10	29.052	2.025	-1.522	1.00	0.00	C
ATOM	150	HA	ARG	10	28.847	1.866	-0.463	1.00	0.00	H
ATOM	151	CB	ARG	10	29.474	0.704	-2.107	1.00	0.00	C
ATOM	152	HB2	ARG	10	28.659	-0.017	-2.088	1.00	0.00	H
ATOM	153	HB3	ARG	10	30.316	0.417	-1.477	1.00	0.00	H
ATOM	154	CG	ARG	10	29.754	0.766	-3.514	1.00	0.00	C
ATOM	155	HG2	ARG	10	30.402	1.600	-3.783	1.00	0.00	H
ATOM	156	HG3	ARG	10	28.833	1.023	-4.037	1.00	0.00	H
ATOM	157	CD	ARG	10	30.260	-0.541	-4.178	1.00	0.00	C

ATOM	158	HD2	ARG	10	30.510	-0.409	-5.231	1.00	0.00	H
ATOM	159	HD3	ARG	10	29.425	-1.240	-4.205	1.00	0.00	H
ATOM	160	NE	ARG	10	31.486	-1.038	-3.504	1.00	0.00	N
ATOM	161	HE	ARG	10	32.325	-0.545	-3.775	1.00	0.00	H
ATOM	162	CZ	ARG	10	31.590	-1.973	-2.555	1.00	0.00	C
ATOM	163	NH1	ARG	10	30.657	-2.721	-2.090	1.00	0.00	N
ATOM	164	HH11	ARG	10	29.686	-2.545	-2.299	1.00	0.00	H
ATOM	165	HH12	ARG	10	30.875	-3.098	-1.178	1.00	0.00	H
ATOM	166	NH2	ARG	10	32.716	-2.059	-1.931	1.00	0.00	N
ATOM	167	HH21	ARG	10	33.460	-1.497	-2.319	1.00	0.00	H
ATOM	168	HH22	ARG	10	32.911	-2.773	-1.243	1.00	0.00	H
ATOM	169	C	ARG	10	30.144	3.065	-1.540	1.00	0.00	C
ATOM	170	O	ARG	10	30.193	3.916	-2.456	1.00	0.00	O
ATOM	171	N	GLU	11	31.120	2.833	-0.659	1.00	0.00	N
ATOM	172	H	GLU	11	30.962	2.020	-0.080	1.00	0.00	H
ATOM	173	CA	GLU	11	32.412	3.572	-0.582	1.00	0.00	C
ATOM	174	HA	GLU	11	32.804	3.551	0.435	1.00	0.00	H
ATOM	175	CB	GLU	11	33.489	2.812	-1.435	1.00	0.00	C
ATOM	176	HB2	GLU	11	34.486	3.234	-1.304	1.00	0.00	H
ATOM	177	HB3	GLU	11	33.195	3.097	-2.445	1.00	0.00	H
ATOM	178	CG	GLU	11	33.634	1.343	-1.286	1.00	0.00	C
ATOM	179	HG2	GLU	11	32.720	0.750	-1.301	1.00	0.00	H
ATOM	180	HG3	GLU	11	34.129	1.236	-0.321	1.00	0.00	H
ATOM	181	CD	GLU	11	34.579	0.824	-2.364	1.00	0.00	C
ATOM	182	OE1	GLU	11	35.729	1.302	-2.463	1.00	0.00	O
ATOM	183	OE2	GLU	11	34.091	-0.051	-3.222	1.00	0.00	O
ATOM	184	C	GLU	11	32.308	5.082	-0.866	1.00	0.00	C
ATOM	185	O	GLU	11	33.098	5.822	-1.465	1.00	0.00	O
ATOM	186	N	ASN	12	31.225	5.730	-0.342	1.00	0.00	N
ATOM	187	H	ASN	12	30.721	5.159	0.321	1.00	0.00	H
ATOM	188	CA	ASN	12	30.880	7.136	-0.777	1.00	0.00	C
ATOM	189	HA	ASN	12	30.971	7.310	-1.849	1.00	0.00	H
ATOM	190	CB	ASN	12	29.390	7.313	-0.411	1.00	0.00	C
ATOM	191	HB2	ASN	12	28.733	6.814	-1.124	1.00	0.00	H
ATOM	192	HB3	ASN	12	29.148	8.375	-0.454	1.00	0.00	H
ATOM	193	CG	ASN	12	29.006	7.021	1.051	1.00	0.00	C
ATOM	194	OD1	ASN	12	29.248	7.886	1.976	1.00	0.00	O
ATOM	195	ND2	ASN	12	28.492	5.850	1.394	1.00	0.00	N
ATOM	196	HD21	ASN	12	27.976	5.840	2.261	1.00	0.00	H
ATOM	197	HD22	ASN	12	28.189	5.226	0.658	1.00	0.00	H
ATOM	198	C	ASN	12	31.928	8.123	-0.147	1.00	0.00	C
ATOM	199	O	ASN	12	32.236	9.180	-0.755	1.00	0.00	O
ATOM	200	N	MET	13	32.393	7.711	1.005	1.00	0.00	N
ATOM	201	H	MET	13	31.976	6.853	1.341	1.00	0.00	H
ATOM	202	CA	MET	13	33.492	8.425	1.733	1.00	0.00	C
ATOM	203	HA	MET	13	33.139	9.420	2.005	1.00	0.00	H
ATOM	204	CB	MET	13	33.823	7.592	2.942	1.00	0.00	C
ATOM	205	HB2	MET	13	34.285	6.644	2.668	1.00	0.00	H
ATOM	206	HB3	MET	13	32.847	7.539	3.424	1.00	0.00	H
ATOM	207	CG	MET	13	34.753	8.385	3.931	1.00	0.00	C
ATOM	208	HG2	MET	13	34.398	9.416	3.965	1.00	0.00	H
ATOM	209	HG3	MET	13	35.669	8.172	3.380	1.00	0.00	H
ATOM	210	SD	MET	13	34.742	7.803	5.672	1.00	0.00	S
ATOM	211	CE	MET	13	35.749	8.985	6.562	1.00	0.00	C
ATOM	212	HE1	MET	13	35.966	9.899	6.009	1.00	0.00	H
ATOM	213	HE2	MET	13	35.193	9.240	7.464	1.00	0.00	H
ATOM	214	HE3	MET	13	36.718	8.499	6.677	1.00	0.00	H
ATOM	215	C	MET	13	34.661	8.569	0.766	1.00	0.00	C
ATOM	216	O	MET	13	35.439	9.464	0.947	1.00	0.00	O
ATOM	217	N	TYR	14	34.955	7.506	-0.056	1.00	0.00	N
ATOM	218	H	TYR	14	34.245	6.801	-0.189	1.00	0.00	H
ATOM	219	CA	TYR	14	36.174	7.386	-0.881	1.00	0.00	C
ATOM	220	HA	TYR	14	37.001	7.984	-0.502	1.00	0.00	H

ATOM	221	CB	TYR	14	36.608	5.918	-0.964	1.00	0.00	C
ATOM	222	HB2	TYR	14	37.581	5.823	-1.445	1.00	0.00	H
ATOM	223	HB3	TYR	14	35.817	5.358	-1.465	1.00	0.00	H
ATOM	224	CG	TYR	14	36.837	5.122	0.345	1.00	0.00	C
ATOM	225	CD1	TYR	14	36.797	3.654	0.355	1.00	0.00	C
ATOM	226	HD1	TYR	14	36.672	3.129	-0.580	1.00	0.00	H
ATOM	227	CE1	TYR	14	37.055	2.838	1.481	1.00	0.00	C
ATOM	228	HE1	TYR	14	37.117	1.760	1.463	1.00	0.00	H
ATOM	229	CZ	TYR	14	37.514	3.539	2.635	1.00	0.00	C
ATOM	230	OH	TYR	14	37.721	2.789	3.766	1.00	0.00	O
ATOM	231	HH	TYR	14	38.006	3.393	4.454	1.00	0.00	H
ATOM	232	CE2	TYR	14	37.597	4.948	2.669	1.00	0.00	C
ATOM	233	HE2	TYR	14	37.997	5.491	3.513	1.00	0.00	H
ATOM	234	CD2	TYR	14	37.208	5.763	1.564	1.00	0.00	C
ATOM	235	HD2	TYR	14	37.344	6.834	1.530	1.00	0.00	H
ATOM	236	C	TYR	14	35.944	8.009	-2.318	1.00	0.00	C
ATOM	237	O	TYR	14	36.912	8.472	-2.862	1.00	0.00	O
ATOM	238	N	ARG	15	34.715	7.837	-2.813	1.00	0.00	N
ATOM	239	H	ARG	15	34.050	7.363	-2.219	1.00	0.00	H
ATOM	240	CA	ARG	15	34.318	8.118	-4.187	1.00	0.00	C
ATOM	241	HA	ARG	15	35.230	8.127	-4.785	1.00	0.00	H
ATOM	242	CB	ARG	15	33.296	7.071	-4.716	1.00	0.00	C
ATOM	243	HB2	ARG	15	32.897	7.351	-5.692	1.00	0.00	H
ATOM	244	HB3	ARG	15	32.473	7.196	-4.012	1.00	0.00	H
ATOM	245	CG	ARG	15	33.700	5.656	-4.819	1.00	0.00	C
ATOM	246	HG2	ARG	15	34.165	5.323	-3.891	1.00	0.00	H
ATOM	247	HG3	ARG	15	34.314	5.555	-5.714	1.00	0.00	H
ATOM	248	CD	ARG	15	32.454	4.761	-4.865	1.00	0.00	C
ATOM	249	HD2	ARG	15	31.872	5.045	-3.988	1.00	0.00	H
ATOM	250	HD3	ARG	15	32.839	3.742	-4.849	1.00	0.00	H
ATOM	251	NE	ARG	15	31.638	4.898	-6.118	1.00	0.00	N
ATOM	252	HE	ARG	15	32.132	5.003	-6.992	1.00	0.00	H
ATOM	253	CZ	ARG	15	30.343	4.983	-6.197	1.00	0.00	C
ATOM	254	NH1	ARG	15	29.592	4.870	-5.174	1.00	0.00	N
ATOM	255	HH11	ARG	15	29.924	4.616	-4.254	1.00	0.00	H
ATOM	256	HH12	ARG	15	28.618	5.136	-5.149	1.00	0.00	H
ATOM	257	NH2	ARG	15	29.828	5.118	-7.347	1.00	0.00	N
ATOM	258	HH21	ARG	15	28.921	4.672	-7.355	1.00	0.00	H
ATOM	259	HH22	ARG	15	30.234	5.419	-8.221	1.00	0.00	H
ATOM	260	C	ARG	15	33.793	9.583	-4.373	1.00	0.00	C
ATOM	261	O	ARG	15	34.233	10.183	-5.310	1.00	0.00	O
ATOM	262	N	TYR	16	32.959	10.118	-3.469	1.00	0.00	N
ATOM	263	H	TYR	16	32.534	9.575	-2.731	1.00	0.00	H
ATOM	264	CA	TYR	16	32.498	11.563	-3.473	1.00	0.00	C
ATOM	265	HA	TYR	16	32.749	11.956	-4.458	1.00	0.00	H
ATOM	266	CB	TYR	16	31.000	11.783	-3.432	1.00	0.00	C
ATOM	267	HB2	TYR	16	30.794	12.743	-3.904	1.00	0.00	H
ATOM	268	HB3	TYR	16	30.824	11.795	-2.355	1.00	0.00	H
ATOM	269	CG	TYR	16	30.145	10.741	-4.170	1.00	0.00	C
ATOM	270	CD1	TYR	16	29.906	10.952	-5.547	1.00	0.00	C
ATOM	271	HD1	TYR	16	30.345	11.799	-6.054	1.00	0.00	H
ATOM	272	CE1	TYR	16	29.152	9.937	-6.229	1.00	0.00	C
ATOM	273	HE1	TYR	16	28.746	10.030	-7.226	1.00	0.00	H
ATOM	274	CZ	TYR	16	28.778	8.768	-5.555	1.00	0.00	C
ATOM	275	OH	TYR	16	28.107	7.697	-6.147	1.00	0.00	O
ATOM	276	HH	TYR	16	27.534	7.883	-6.895	1.00	0.00	H
ATOM	277	CE2	TYR	16	28.975	8.657	-4.179	1.00	0.00	C
ATOM	278	HE2	TYR	16	28.723	7.716	-3.712	1.00	0.00	H
ATOM	279	CD2	TYR	16	29.646	9.636	-3.447	1.00	0.00	C
ATOM	280	HD2	TYR	16	29.747	9.450	-2.388	1.00	0.00	H
ATOM	281	C	TYR	16	33.273	12.477	-2.485	1.00	0.00	C
ATOM	282	O	TYR	16	33.440	13.660	-2.698	1.00	0.00	O
ATOM	283	N	NME	17	33.683	11.789	-1.442	1.00	0.00	N

```
ATOM    284  H   NME    17      33.603  10.784  -1.380  1.00  0.00           H
ATOM    285  CH3 NME    17      34.343  12.473  -0.291  1.00  0.00           C
ATOM    286 HH31 NME    17      34.557  13.529  -0.459  1.00  0.00           H
ATOM    287 HH32 NME    17      33.934  12.123   0.656  1.00  0.00           H
ATOM    288 HH33 NME    17      35.333  12.025  -0.198  1.00  0.00           H
TER     289      NME    17
ATOM    289 HH31 ACE    18      15.096  20.205  13.701  1.00  0.00           H
ATOM    290  CH3 ACE    18      15.211  19.659  12.764  1.00  0.00           C
ATOM    291 HH32 ACE    18      15.644  20.245  11.953  1.00  0.00           H
ATOM    292 HH33 ACE    18      14.242  19.318  12.401  1.00  0.00           H
ATOM    293  C   ACE    18      16.058  18.386  13.074  1.00  0.00           C
ATOM    294  O   ACE    18      17.234  18.537  12.857  1.00  0.00           O
ATOM    295  N   LYS    19      15.446  17.332  13.544  1.00  0.00           N
ATOM    296  H   LYS    19      14.438  17.283  13.537  1.00  0.00           H
ATOM    297  CA  LYS    19      16.253  16.183  14.190  1.00  0.00           C
ATOM    298  HA  LYS    19      17.019  16.629  14.825  1.00  0.00           H
ATOM    299  CB  LYS    19      15.405  15.186  14.995  1.00  0.00           C
ATOM    300  HB2 LYS    19      15.798  14.176  15.102  1.00  0.00           H
ATOM    301  HB3 LYS    19      14.536  15.048  14.350  1.00  0.00           H
ATOM    302  CG  LYS    19      15.027  15.691  16.429  1.00  0.00           C
ATOM    303  HG2 LYS    19      15.699  15.389  17.232  1.00  0.00           H
ATOM    304  HG3 LYS    19      14.068  15.189  16.559  1.00  0.00           H
ATOM    305  CD  LYS    19      14.684  17.113  16.635  1.00  0.00           C
ATOM    306  HD2 LYS    19      13.743  17.402  16.166  1.00  0.00           H
ATOM    307  HD3 LYS    19      15.529  17.732  16.332  1.00  0.00           H
ATOM    308  CE  LYS    19      14.356  17.411  18.115  1.00  0.00           C
ATOM    309  HE2 LYS    19      13.979  16.562  18.685  1.00  0.00           H
ATOM    310  HE3 LYS    19      13.580  18.176  18.090  1.00  0.00           H
ATOM    311  NZ  LYS    19      15.464  18.023  18.907  1.00  0.00           N
ATOM    312  HZ1 LYS    19      16.312  17.474  18.889  1.00  0.00           H
ATOM    313  HZ2 LYS    19      15.772  18.925  18.572  1.00  0.00           H
ATOM    314  HZ3 LYS    19      15.042  18.107  19.821  1.00  0.00           H
ATOM    315  C   LYS    19      17.087  15.437  13.138  1.00  0.00           C
ATOM    316  O   LYS    19      16.619  15.153  12.056  1.00  0.00           O
ATOM    317  N   GLY    20      18.281  15.126  13.531  1.00  0.00           N
ATOM    318  H   GLY    20      18.556  15.395  14.466  1.00  0.00           H
ATOM    319  CA  GLY    20      19.076  14.098  12.792  1.00  0.00           C
ATOM    320  HA2 GLY    20      19.101  14.344  11.730  1.00  0.00           H
ATOM    321  HA3 GLY    20      20.110  14.202  13.122  1.00  0.00           H
ATOM    322  C   GLY    20      18.628  12.675  12.959  1.00  0.00           C
ATOM    323  O   GLY    20      18.021  12.363  13.922  1.00  0.00           O
ATOM    324  N   GLU    21      18.893  11.791  11.990  1.00  0.00           N
ATOM    325  H   GLU    21      19.560  12.029  11.270  1.00  0.00           H
ATOM    326  CA  GLU    21      18.577  10.309  12.001  1.00  0.00           C
ATOM    327  HA  GLU    21      18.420   9.980  13.029  1.00  0.00           H
ATOM    328  CB  GLU    21      17.407  10.006  11.085  1.00  0.00           C
ATOM    329  HB2 GLU    21      17.152   8.949  10.997  1.00  0.00           H
ATOM    330  HB3 GLU    21      17.751  10.312  10.096  1.00  0.00           H
ATOM    331  CG  GLU    21      16.105  10.719  11.471  1.00  0.00           C
ATOM    332  HG2 GLU    21      16.120  11.809  11.438  1.00  0.00           H
ATOM    333  HG3 GLU    21      15.880  10.416  12.493  1.00  0.00           H
ATOM    334  CD  GLU    21      14.913  10.361  10.566  1.00  0.00           C
ATOM    335  OE1 GLU    21      14.871  10.765   9.366  1.00  0.00           O
ATOM    336  OE2 GLU    21      14.101   9.492  11.036  1.00  0.00           O
ATOM    337  C   GLU    21      19.816   9.576  11.441  1.00  0.00           C
ATOM    338  O   GLU    21      20.487   9.957  10.404  1.00  0.00           O
ATOM    339  N   ASN    22      20.173   8.368  12.019  1.00  0.00           N
ATOM    340  H   ASN    22      19.442   7.912  12.545  1.00  0.00           H
ATOM    341  CA  ASN    22      21.360   7.544  11.781  1.00  0.00           C
ATOM    342  HA  ASN    22      22.016   8.181  11.189  1.00  0.00           H
ATOM    343  CB  ASN    22      22.166   7.299  13.078  1.00  0.00           C
ATOM    344  HB2 ASN    22      21.448   7.003  13.843  1.00  0.00           H
ATOM    345  HB3 ASN    22      22.508   8.306  13.318  1.00  0.00           H
```

ATOM	346	CG	ASN	22	23.333	6.387	13.090	1.00	0.00	C
ATOM	347	OD1	ASN	22	24.436	6.691	12.657	1.00	0.00	O
ATOM	348	ND2	ASN	22	23.231	5.156	13.465	1.00	0.00	N
ATOM	349	HD21	ASN	22	24.042	4.610	13.211	1.00	0.00	H
ATOM	350	HD22	ASN	22	22.332	4.725	13.626	1.00	0.00	H
ATOM	351	C	ASN	22	21.141	6.323	10.878	1.00	0.00	C
ATOM	352	O	ASN	22	20.554	5.317	11.331	1.00	0.00	O
ATOM	353	N	PHE	23	21.570	6.257	9.679	1.00	0.00	N
ATOM	354	H	PHE	23	21.929	7.147	9.365	1.00	0.00	H
ATOM	355	CA	PHE	23	21.486	5.140	8.759	1.00	0.00	C
ATOM	356	HA	PHE	23	20.783	4.361	9.054	1.00	0.00	H
ATOM	357	CB	PHE	23	20.928	5.521	7.437	1.00	0.00	C
ATOM	358	HB2	PHE	23	21.071	4.714	6.718	1.00	0.00	H
ATOM	359	HB3	PHE	23	21.561	6.387	7.245	1.00	0.00	H
ATOM	360	CG	PHE	23	19.484	6.014	7.314	1.00	0.00	C
ATOM	361	CD1	PHE	23	18.478	5.034	7.117	1.00	0.00	C
ATOM	362	HD1	PHE	23	18.669	3.970	7.138	1.00	0.00	H
ATOM	363	CE1	PHE	23	17.134	5.477	6.988	1.00	0.00	C
ATOM	364	HE1	PHE	23	16.323	4.764	6.965	1.00	0.00	H
ATOM	365	CZ	PHE	23	16.859	6.882	7.172	1.00	0.00	C
ATOM	366	HZ	PHE	23	15.845	7.230	7.308	1.00	0.00	H
ATOM	367	CE2	PHE	23	17.879	7.824	7.364	1.00	0.00	C
ATOM	368	HE2	PHE	23	17.697	8.886	7.447	1.00	0.00	H
ATOM	369	CD2	PHE	23	19.226	7.349	7.389	1.00	0.00	C
ATOM	370	HD2	PHE	23	20.011	8.050	7.636	1.00	0.00	H
ATOM	371	C	PHE	23	22.866	4.447	8.634	1.00	0.00	C
ATOM	372	O	PHE	23	23.901	5.005	8.892	1.00	0.00	O
ATOM	373	N	THR	24	22.804	3.208	8.220	1.00	0.00	N
ATOM	374	H	THR	24	21.909	2.805	7.984	1.00	0.00	H
ATOM	375	CA	THR	24	23.875	2.241	7.967	1.00	0.00	C
ATOM	376	HA	THR	24	24.611	2.246	8.771	1.00	0.00	H
ATOM	377	CB	THR	24	23.343	0.805	7.835	1.00	0.00	C
ATOM	378	HB	THR	24	24.065	0.166	7.327	1.00	0.00	H
ATOM	379	CG2	THR	24	23.009	0.287	9.242	1.00	0.00	C
ATOM	380	HG21	THR	24	22.513	-0.683	9.277	1.00	0.00	H
ATOM	381	HG22	THR	24	23.950	0.258	9.790	1.00	0.00	H
ATOM	382	HG23	THR	24	22.437	0.997	9.839	1.00	0.00	H
ATOM	383	OG1	THR	24	22.162	0.794	7.046	1.00	0.00	O
ATOM	384	HG1	THR	24	22.350	0.781	6.105	1.00	0.00	H
ATOM	385	C	THR	24	24.755	2.594	6.763	1.00	0.00	C
ATOM	386	O	THR	24	25.806	1.932	6.586	1.00	0.00	O
ATOM	387	N	GLU	25	24.317	3.555	5.901	1.00	0.00	N
ATOM	388	H	GLU	25	23.494	4.088	6.140	1.00	0.00	H
ATOM	389	CA	GLU	25	24.847	3.852	4.546	1.00	0.00	C
ATOM	390	HA	GLU	25	25.459	3.005	4.238	1.00	0.00	H
ATOM	391	CB	GLU	25	23.666	4.100	3.620	1.00	0.00	C
ATOM	392	HB2	GLU	25	23.975	3.980	2.582	1.00	0.00	H
ATOM	393	HB3	GLU	25	23.461	5.141	3.873	1.00	0.00	H
ATOM	394	CG	GLU	25	22.383	3.285	3.728	1.00	0.00	C
ATOM	395	HG2	GLU	25	21.625	3.472	2.966	1.00	0.00	H
ATOM	396	HG3	GLU	25	21.951	3.541	4.695	1.00	0.00	H
ATOM	397	CD	GLU	25	22.642	1.709	3.524	1.00	0.00	C
ATOM	398	OE1	GLU	25	22.675	0.845	4.447	1.00	0.00	O
ATOM	399	OE2	GLU	25	23.006	1.349	2.377	1.00	0.00	O
ATOM	400	C	GLU	25	25.961	4.949	4.472	1.00	0.00	C
ATOM	401	O	GLU	25	26.254	5.394	3.346	1.00	0.00	O
ATOM	402	N	THR	26	26.247	5.615	5.611	1.00	0.00	N
ATOM	403	H	THR	26	25.866	5.242	6.468	1.00	0.00	H
ATOM	404	CA	THR	26	27.248	6.705	5.740	1.00	0.00	C
ATOM	405	HA	THR	26	27.762	6.796	4.783	1.00	0.00	H
ATOM	406	CB	THR	26	26.575	8.077	5.913	1.00	0.00	C
ATOM	407	HB	THR	26	27.385	8.750	6.192	1.00	0.00	H
ATOM	408	CG2	THR	26	25.721	8.491	4.681	1.00	0.00	C

ATOM	409	HG21	THR	26	25.078	9.279	5.071	1.00	0.00	H
ATOM	410	HG22	THR	26	26.442	8.652	3.879	1.00	0.00	H
ATOM	411	HG23	THR	26	25.064	7.710	4.301	1.00	0.00	H
ATOM	412	OG1	THR	26	25.646	7.928	6.990	1.00	0.00	O
ATOM	413	HG1	THR	26	25.211	8.782	6.935	1.00	0.00	H
ATOM	414	C	THR	26	28.351	6.482	6.786	1.00	0.00	C
ATOM	415	O	THR	26	29.272	7.259	6.774	1.00	0.00	O
ATOM	416	N	ASP	27	28.242	5.412	7.582	1.00	0.00	N
ATOM	417	H	ASP	27	27.478	4.752	7.566	1.00	0.00	H
ATOM	418	CA	ASP	27	29.088	5.016	8.745	1.00	0.00	C
ATOM	419	HA	ASP	27	29.058	5.873	9.418	1.00	0.00	H
ATOM	420	CB	ASP	27	28.636	3.705	9.398	1.00	0.00	C
ATOM	421	HB2	ASP	27	28.885	2.738	8.963	1.00	0.00	H
ATOM	422	HB3	ASP	27	27.546	3.718	9.404	1.00	0.00	H
ATOM	423	CG	ASP	27	29.203	3.614	10.858	1.00	0.00	C
ATOM	424	OD1	ASP	27	28.514	4.004	11.819	1.00	0.00	O
ATOM	425	OD2	ASP	27	30.365	3.281	11.033	1.00	0.00	O
ATOM	426	C	ASP	27	30.578	4.946	8.316	1.00	0.00	C
ATOM	427	O	ASP	27	30.931	4.364	7.260	1.00	0.00	O
ATOM	428	N	ILE	28	31.391	5.647	9.153	1.00	0.00	N
ATOM	429	H	ILE	28	30.957	6.094	9.948	1.00	0.00	H
ATOM	430	CA	ILE	28	32.870	5.778	8.916	1.00	0.00	C
ATOM	431	HA	ILE	28	32.958	5.781	7.830	1.00	0.00	H
ATOM	432	CB	ILE	28	33.448	7.165	9.332	1.00	0.00	C
ATOM	433	HB	ILE	28	34.425	7.356	8.889	1.00	0.00	H
ATOM	434	CG2	ILE	28	32.499	8.248	8.802	1.00	0.00	C
ATOM	435	HG21	ILE	28	32.435	8.203	7.714	1.00	0.00	H
ATOM	436	HG22	ILE	28	31.465	8.181	9.141	1.00	0.00	H
ATOM	437	HG23	ILE	28	32.869	9.192	9.200	1.00	0.00	H
ATOM	438	CG1	ILE	28	33.535	7.154	10.864	1.00	0.00	C
ATOM	439	HG12	ILE	28	34.213	6.374	11.212	1.00	0.00	H
ATOM	440	HG13	ILE	28	32.497	6.910	11.086	1.00	0.00	H
ATOM	441	CD1	ILE	28	33.911	8.392	11.601	1.00	0.00	C
ATOM	442	HD11	ILE	28	34.000	8.205	12.671	1.00	0.00	H
ATOM	443	HD12	ILE	28	34.879	8.732	11.234	1.00	0.00	H
ATOM	444	HD13	ILE	28	33.151	9.141	11.376	1.00	0.00	H
ATOM	445	C	ILE	28	33.810	4.586	9.328	1.00	0.00	C
ATOM	446	O	ILE	28	34.979	4.691	8.978	1.00	0.00	O
ATOM	447	N	LYS	29	33.268	3.504	9.870	1.00	0.00	N
ATOM	448	H	LYS	29	32.280	3.488	10.076	1.00	0.00	H
ATOM	449	CA	LYS	29	34.003	2.243	10.272	1.00	0.00	C
ATOM	450	HA	LYS	29	35.067	2.452	10.153	1.00	0.00	H
ATOM	451	CB	LYS	29	33.652	2.054	11.796	1.00	0.00	C
ATOM	452	HB2	LYS	29	34.318	1.264	12.145	1.00	0.00	H
ATOM	453	HB3	LYS	29	32.630	1.678	11.756	1.00	0.00	H
ATOM	454	CG	LYS	29	33.643	3.270	12.677	1.00	0.00	C
ATOM	455	HG2	LYS	29	33.065	4.098	12.264	1.00	0.00	H
ATOM	456	HG3	LYS	29	34.682	3.567	12.817	1.00	0.00	H
ATOM	457	CD	LYS	29	33.092	3.080	14.138	1.00	0.00	C
ATOM	458	HD2	LYS	29	33.458	3.814	14.856	1.00	0.00	H
ATOM	459	HD3	LYS	29	33.634	2.231	14.552	1.00	0.00	H
ATOM	460	CE	LYS	29	31.597	2.908	14.282	1.00	0.00	C
ATOM	461	HE2	LYS	29	31.422	2.844	15.356	1.00	0.00	H
ATOM	462	HE3	LYS	29	31.327	1.936	13.870	1.00	0.00	H
ATOM	463	NZ	LYS	29	30.740	4.034	13.743	1.00	0.00	N
ATOM	464	HZ1	LYS	29	29.769	3.773	13.637	1.00	0.00	H
ATOM	465	HZ2	LYS	29	30.904	4.781	14.404	1.00	0.00	H
ATOM	466	HZ3	LYS	29	30.995	4.311	12.807	1.00	0.00	H
ATOM	467	C	LYS	29	33.687	1.096	9.374	1.00	0.00	C
ATOM	468	O	LYS	29	34.505	0.175	9.187	1.00	0.00	O
ATOM	469	N	ILE	30	32.529	1.150	8.663	1.00	0.00	N
ATOM	470	H	ILE	30	31.942	1.966	8.751	1.00	0.00	H
ATOM	471	CA	ILE	30	32.156	0.079	7.626	1.00	0.00	C

ATOM	472	HA	ILE	30	32.555	-0.855	8.023	1.00	0.00	H
ATOM	473	CB	ILE	30	30.647	-0.145	7.513	1.00	0.00	C
ATOM	474	HB	ILE	30	30.228	0.783	7.124	1.00	0.00	H
ATOM	475	CG2	ILE	30	30.238	-1.253	6.539	1.00	0.00	C
ATOM	476	HG21	ILE	30	29.251	-0.988	6.160	1.00	0.00	H
ATOM	477	HG22	ILE	30	30.880	-1.315	5.659	1.00	0.00	H
ATOM	478	HG23	ILE	30	30.219	-2.261	6.953	1.00	0.00	H
ATOM	479	CG1	ILE	30	30.079	-0.447	8.925	1.00	0.00	C
ATOM	480	HG12	ILE	30	30.223	0.348	9.656	1.00	0.00	H
ATOM	481	HG13	ILE	30	30.413	-1.375	9.389	1.00	0.00	H
ATOM	482	CD1	ILE	30	28.539	-0.530	8.967	1.00	0.00	C
ATOM	483	HD11	ILE	30	28.078	-1.117	8.172	1.00	0.00	H
ATOM	484	HD12	ILE	30	28.174	-1.104	9.817	1.00	0.00	H
ATOM	485	HD13	ILE	30	28.164	0.493	8.915	1.00	0.00	H
ATOM	486	C	ILE	30	32.795	0.337	6.287	1.00	0.00	C
ATOM	487	O	ILE	30	32.353	1.252	5.547	1.00	0.00	O
ATOM	488	N	MET	31	33.834	-0.412	5.963	1.00	0.00	N
ATOM	489	H	MET	31	34.166	-1.052	6.669	1.00	0.00	H
ATOM	490	CA	MET	31	34.642	-0.188	4.784	1.00	0.00	C
ATOM	491	HA	MET	31	34.977	0.847	4.716	1.00	0.00	H
ATOM	492	CB	MET	31	35.900	-1.196	4.846	1.00	0.00	C
ATOM	493	HB2	MET	31	36.321	-1.391	5.831	1.00	0.00	H
ATOM	494	HB3	MET	31	36.714	-0.754	4.271	1.00	0.00	H
ATOM	495	CG	MET	31	35.503	-2.508	4.229	1.00	0.00	C
ATOM	496	HG2	MET	31	35.024	-2.324	3.267	1.00	0.00	H
ATOM	497	HG3	MET	31	34.815	-2.887	4.985	1.00	0.00	H
ATOM	498	SD	MET	31	36.641	-3.837	3.977	1.00	0.00	S
ATOM	499	CE	MET	31	36.001	-4.581	2.449	1.00	0.00	C
ATOM	500	HE1	MET	31	34.921	-4.438	2.413	1.00	0.00	H
ATOM	501	HE2	MET	31	36.412	-4.031	1.602	1.00	0.00	H
ATOM	502	HE3	MET	31	36.175	-5.657	2.439	1.00	0.00	H
ATOM	503	C	MET	31	33.885	-0.240	3.463	1.00	0.00	C
ATOM	504	O	MET	31	34.258	0.304	2.442	1.00	0.00	O
ATOM	505	N	GLU	32	32.664	-0.761	3.419	1.00	0.00	N
ATOM	506	H	GLU	32	32.272	-1.132	4.273	1.00	0.00	H
ATOM	507	CA	GLU	32	31.745	-0.847	2.285	1.00	0.00	C
ATOM	508	HA	GLU	32	32.276	-1.079	1.362	1.00	0.00	H
ATOM	509	CB	GLU	32	30.838	-1.978	2.564	1.00	0.00	C
ATOM	510	HB2	GLU	32	29.982	-1.909	1.894	1.00	0.00	H
ATOM	511	HB3	GLU	32	30.453	-1.837	3.575	1.00	0.00	H
ATOM	512	CG	GLU	32	31.431	-3.389	2.416	1.00	0.00	C
ATOM	513	HG2	GLU	32	30.691	-4.185	2.481	1.00	0.00	H
ATOM	514	HG3	GLU	32	32.110	-3.650	3.228	1.00	0.00	H
ATOM	515	CD	GLU	32	32.102	-3.630	1.015	1.00	0.00	C
ATOM	516	OE1	GLU	32	33.272	-3.290	0.838	1.00	0.00	O
ATOM	517	OE2	GLU	32	31.454	-4.178	0.090	1.00	0.00	O
ATOM	518	C	GLU	32	30.974	0.469	1.986	1.00	0.00	C
ATOM	519	O	GLU	32	30.042	0.558	1.227	1.00	0.00	O
ATOM	520	N	ARG	33	31.343	1.497	2.782	1.00	0.00	N
ATOM	521	H	ARG	33	32.082	1.345	3.453	1.00	0.00	H
ATOM	522	CA	ARG	33	30.529	2.668	2.987	1.00	0.00	C
ATOM	523	HA	ARG	33	30.013	2.949	2.068	1.00	0.00	H
ATOM	524	CB	ARG	33	29.395	2.458	4.116	1.00	0.00	C
ATOM	525	HB2	ARG	33	28.895	3.349	4.495	1.00	0.00	H
ATOM	526	HB3	ARG	33	29.915	1.952	4.930	1.00	0.00	H
ATOM	527	CG	ARG	33	28.217	1.651	3.829	1.00	0.00	C
ATOM	528	HG2	ARG	33	27.511	1.724	4.656	1.00	0.00	H
ATOM	529	HG3	ARG	33	28.621	0.646	3.707	1.00	0.00	H
ATOM	530	CD	ARG	33	27.467	2.062	2.458	1.00	0.00	C
ATOM	531	HD2	ARG	33	28.096	1.947	1.575	1.00	0.00	H
ATOM	532	HD3	ARG	33	27.162	3.085	2.673	1.00	0.00	H
ATOM	533	NE	ARG	33	26.305	1.197	2.239	1.00	0.00	N
ATOM	534	HE	ARG	33	25.451	1.678	2.480	1.00	0.00	H

```
ATOM    535  CZ  ARG    33      26.273   0.123   1.425  1.00  0.00            C
ATOM    536  NH1 ARG    33      27.298  -0.463   0.926  1.00  0.00            N
ATOM    537 HH11 ARG    33      28.190  -0.087   1.218  1.00  0.00            H
ATOM    538 HH12 ARG    33      27.151  -1.070   0.133  1.00  0.00            H
ATOM    539  NH2 ARG    33      25.110  -0.392   1.101  1.00  0.00            N
ATOM    540 HH21 ARG    33      24.282  -0.013   1.537  1.00  0.00            H
ATOM    541 HH22 ARG    33      25.106  -1.223   0.527  1.00  0.00            H
ATOM    542  C   ARG    33      31.517   3.806   3.274  1.00  0.00            C
ATOM    543  O   ARG    33      31.395   4.706   2.491  1.00  0.00            O
ATOM    544  N   NME    34      32.369   3.773   4.357  1.00  0.00            N
ATOM    545  H   NME    34      32.168   3.070   5.054  1.00  0.00            H
ATOM    546  CH3 NME    34      33.168   4.851   4.783  1.00  0.00            C
ATOM    547 HH31 NME    34      33.977   5.087   4.090  1.00  0.00            H
ATOM    548 HH32 NME    34      32.563   5.752   4.685  1.00  0.00            H
ATOM    549 HH33 NME    34      33.552   4.635   5.779  1.00  0.00            H
TER     550      NME    34
END
```

H1 + H[99]SQWNKPSKPKTNMK[113] simulation

```
#Cluster   Frames     Frac  AvgDist    Stdev Centroid AvgCDist
       0   183773    0.919    1.522    0.705    70735    6.046
       1     9113    0.046    2.482    1.090   133321    5.207
       2     6727    0.034    2.577    1.275   178027    4.964
       3      379    0.002    1.896    0.644   153707    5.525
       4        8    0.000    0.000    0.000        5    5.774
```

Cluster 0, Figure 5A:

```
ATOM      1 HH31 ACE     1      39.716  11.223  -1.179  1.00  0.00            H
ATOM      2  CH3 ACE     1      39.140  11.739  -0.411  1.00  0.00            C
ATOM      3 HH32 ACE     1      39.255  12.787  -0.684  1.00  0.00            H
ATOM      4 HH33 ACE     1      39.405  11.622   0.640  1.00  0.00            H
ATOM      5  C   ACE     1      37.688  11.290  -0.512  1.00  0.00            C
ATOM      6  O   ACE     1      36.893  12.220  -0.572  1.00  0.00            O
ATOM      7  N   ASN     2      37.257  10.004  -0.715  1.00  0.00            N
ATOM      8  H   ASN     2      37.900   9.242  -0.555  1.00  0.00            H
ATOM      9  CA  ASN     2      35.804   9.635  -0.864  1.00  0.00            C
ATOM     10  HA  ASN     2      35.102  10.467  -0.904  1.00  0.00            H
ATOM     11  CB  ASN     2      35.658   8.915  -2.217  1.00  0.00            C
ATOM     12  HB2 ASN     2      36.210   9.523  -2.933  1.00  0.00            H
ATOM     13  HB3 ASN     2      34.602   8.876  -2.485  1.00  0.00            H
ATOM     14  CG  ASN     2      36.317   7.475  -2.323  1.00  0.00            C
ATOM     15  OD1 ASN     2      35.735   6.472  -2.000  1.00  0.00            O
ATOM     16  ND2 ASN     2      37.575   7.325  -2.771  1.00  0.00            N
ATOM     17 HD21 ASN     2      37.913   6.391  -2.956  1.00  0.00            H
ATOM     18 HD22 ASN     2      38.242   8.068  -2.924  1.00  0.00            H
ATOM     19  C   ASN     2      35.286   8.756   0.278  1.00  0.00            C
ATOM     20  O   ASN     2      36.071   8.124   1.012  1.00  0.00            O
ATOM     21  N   ASP     3      33.985   8.575   0.335  1.00  0.00            N
ATOM     22  H   ASP     3      33.424   9.060  -0.351  1.00  0.00            H
ATOM     23  CA  ASP     3      33.384   7.483   1.157  1.00  0.00            C
ATOM     24  HA  ASP     3      34.147   6.704   1.160  1.00  0.00            H
ATOM     25  CB  ASP     3      33.155   7.950   2.603  1.00  0.00            C
ATOM     26  HB2 ASP     3      34.005   8.413   3.105  1.00  0.00            H
ATOM     27  HB3 ASP     3      32.921   7.027   3.133  1.00  0.00            H
ATOM     28  CG  ASP     3      31.924   8.757   2.939  1.00  0.00            C
ATOM     29  OD1 ASP     3      30.799   8.257   2.988  1.00  0.00            O
```

ATOM	30	OD2	ASP	3	32.154	9.989	3.083	1.00	0.00	O
ATOM	31	C	ASP	3	32.040	6.897	0.569	1.00	0.00	C
ATOM	32	O	ASP	3	31.171	7.597	0.093	1.00	0.00	O
ATOM	33	N	TYR	4	31.960	5.567	0.530	1.00	0.00	N
ATOM	34	H	TYR	4	32.713	5.097	1.013	1.00	0.00	H
ATOM	35	CA	TYR	4	30.870	4.779	0.021	1.00	0.00	C
ATOM	36	HA	TYR	4	30.706	5.095	-1.010	1.00	0.00	H
ATOM	37	CB	TYR	4	31.205	3.289	0.067	1.00	0.00	C
ATOM	38	HB2	TYR	4	31.328	2.959	1.097	1.00	0.00	H
ATOM	39	HB3	TYR	4	32.205	3.196	-0.356	1.00	0.00	H
ATOM	40	CG	TYR	4	30.099	2.349	-0.530	1.00	0.00	C
ATOM	41	CD1	TYR	4	30.188	1.939	-1.853	1.00	0.00	C
ATOM	42	HD1	TYR	4	30.942	2.383	-2.485	1.00	0.00	H
ATOM	43	CE1	TYR	4	29.273	0.997	-2.403	1.00	0.00	C
ATOM	44	HE1	TYR	4	29.407	0.821	-3.460	1.00	0.00	H
ATOM	45	CZ	TYR	4	28.181	0.594	-1.634	1.00	0.00	C
ATOM	46	OH	TYR	4	27.309	-0.268	-2.163	1.00	0.00	O
ATOM	47	HH	TYR	4	27.523	-0.785	-2.943	1.00	0.00	H
ATOM	48	CE2	TYR	4	28.055	0.972	-0.288	1.00	0.00	C
ATOM	49	HE2	TYR	4	27.124	0.726	0.200	1.00	0.00	H
ATOM	50	CD2	TYR	4	29.063	1.767	0.258	1.00	0.00	C
ATOM	51	HD2	TYR	4	28.978	1.966	1.316	1.00	0.00	H
ATOM	52	C	TYR	4	29.557	4.980	0.845	1.00	0.00	C
ATOM	53	O	TYR	4	29.530	4.808	2.041	1.00	0.00	O
ATOM	54	N	GLU	5	28.495	5.414	0.130	1.00	0.00	N
ATOM	55	H	GLU	5	28.549	5.492	-0.876	1.00	0.00	H
ATOM	56	CA	GLU	5	27.231	5.814	0.666	1.00	0.00	C
ATOM	57	HA	GLU	5	27.268	5.690	1.748	1.00	0.00	H
ATOM	58	CB	GLU	5	27.055	7.274	0.290	1.00	0.00	C
ATOM	59	HB2	GLU	5	26.985	7.302	-0.798	1.00	0.00	H
ATOM	60	HB3	GLU	5	27.881	7.961	0.472	1.00	0.00	H
ATOM	61	CG	GLU	5	25.743	7.948	0.652	1.00	0.00	C
ATOM	62	HG2	GLU	5	24.849	7.407	0.343	1.00	0.00	H
ATOM	63	HG3	GLU	5	25.861	8.879	0.098	1.00	0.00	H
ATOM	64	CD	GLU	5	25.681	8.156	2.166	1.00	0.00	C
ATOM	65	OE1	GLU	5	26.377	9.052	2.742	1.00	0.00	O
ATOM	66	OE2	GLU	5	25.094	7.215	2.811	1.00	0.00	O
ATOM	67	C	GLU	5	26.056	4.919	0.191	1.00	0.00	C
ATOM	68	O	GLU	5	26.119	4.490	-0.929	1.00	0.00	O
ATOM	69	N	ASP	6	25.052	4.555	1.070	1.00	0.00	N
ATOM	70	H	ASP	6	25.133	4.978	1.984	1.00	0.00	H
ATOM	71	CA	ASP	6	23.954	3.565	0.694	1.00	0.00	C
ATOM	72	HA	ASP	6	24.082	3.306	-0.356	1.00	0.00	H
ATOM	73	CB	ASP	6	23.903	2.347	1.586	1.00	0.00	C
ATOM	74	HB2	ASP	6	23.563	2.637	2.580	1.00	0.00	H
ATOM	75	HB3	ASP	6	24.932	2.031	1.757	1.00	0.00	H
ATOM	76	CG	ASP	6	23.105	1.226	1.011	1.00	0.00	C
ATOM	77	OD1	ASP	6	23.428	0.810	-0.154	1.00	0.00	O
ATOM	78	OD2	ASP	6	22.259	0.603	1.653	1.00	0.00	O
ATOM	79	C	ASP	6	22.583	4.238	0.730	1.00	0.00	C
ATOM	80	O	ASP	6	21.648	3.811	0.054	1.00	0.00	O
ATOM	81	N	ARG	7	22.372	5.333	1.420	1.00	0.00	N
ATOM	82	H	ARG	7	23.123	5.762	1.941	1.00	0.00	H
ATOM	83	CA	ARG	7	21.175	6.140	1.307	1.00	0.00	C
ATOM	84	HA	ARG	7	20.275	5.561	1.509	1.00	0.00	H
ATOM	85	CB	ARG	7	21.301	7.283	2.335	1.00	0.00	C
ATOM	86	HB2	ARG	7	20.413	7.907	2.231	1.00	0.00	H
ATOM	87	HB3	ARG	7	22.267	7.705	2.058	1.00	0.00	H
ATOM	88	CG	ARG	7	21.245	6.807	3.779	1.00	0.00	C
ATOM	89	HG2	ARG	7	21.930	5.976	3.945	1.00	0.00	H
ATOM	90	HG3	ARG	7	20.177	6.642	3.923	1.00	0.00	H
ATOM	91	CD	ARG	7	21.734	7.794	4.778	1.00	0.00	C
ATOM	92	HD2	ARG	7	21.780	7.522	5.833	1.00	0.00	H

ATOM	93	HD3	ARG	7	21.004	8.590	4.632	1.00	0.00	H
ATOM	94	NE	ARG	7	23.081	8.406	4.476	1.00	0.00	N
ATOM	95	HE	ARG	7	23.748	7.898	3.912	1.00	0.00	H
ATOM	96	CZ	ARG	7	23.430	9.608	4.949	1.00	0.00	C
ATOM	97	NH1	ARG	7	22.626	10.486	5.530	1.00	0.00	N
ATOM	98	HH11	ARG	7	21.680	10.229	5.772	1.00	0.00	H
ATOM	99	HH12	ARG	7	22.930	11.425	5.745	1.00	0.00	H
ATOM	100	NH2	ARG	7	24.632	10.023	4.719	1.00	0.00	N
ATOM	101	HH21	ARG	7	25.246	9.530	4.085	1.00	0.00	H
ATOM	102	HH22	ARG	7	24.778	11.013	4.848	1.00	0.00	H
ATOM	103	C	ARG	7	21.049	6.683	-0.097	1.00	0.00	C
ATOM	104	O	ARG	7	22.007	6.792	-0.855	1.00	0.00	O
ATOM	105	N	TYR	8	19.774	7.063	-0.338	1.00	0.00	N
ATOM	106	H	TYR	8	19.041	6.901	0.338	1.00	0.00	H
ATOM	107	CA	TYR	8	19.340	7.720	-1.553	1.00	0.00	C
ATOM	108	HA	TYR	8	19.955	7.363	-2.378	1.00	0.00	H
ATOM	109	CB	TYR	8	17.889	7.377	-1.836	1.00	0.00	C
ATOM	110	HB2	TYR	8	17.722	6.308	-1.971	1.00	0.00	H
ATOM	111	HB3	TYR	8	17.806	7.799	-2.838	1.00	0.00	H
ATOM	112	CG	TYR	8	16.750	7.899	-0.964	1.00	0.00	C
ATOM	113	CD1	TYR	8	16.186	9.142	-1.337	1.00	0.00	C
ATOM	114	HD1	TYR	8	16.619	9.656	-2.183	1.00	0.00	H
ATOM	115	CE1	TYR	8	15.221	9.694	-0.441	1.00	0.00	C
ATOM	116	HE1	TYR	8	14.815	10.659	-0.703	1.00	0.00	H
ATOM	117	CZ	TYR	8	14.701	8.912	0.588	1.00	0.00	C
ATOM	118	OH	TYR	8	13.646	9.493	1.295	1.00	0.00	O
ATOM	119	HH	TYR	8	13.217	8.849	1.862	1.00	0.00	H
ATOM	120	CE2	TYR	8	15.225	7.694	0.908	1.00	0.00	C
ATOM	121	HE2	TYR	8	14.889	7.079	1.730	1.00	0.00	H
ATOM	122	CD2	TYR	8	16.251	7.139	0.103	1.00	0.00	C
ATOM	123	HD2	TYR	8	16.611	6.155	0.364	1.00	0.00	H
ATOM	124	C	TYR	8	19.636	9.276	-1.532	1.00	0.00	C
ATOM	125	O	TYR	8	19.799	9.868	-2.567	1.00	0.00	O
ATOM	126	N	TYR	9	19.875	9.830	-0.329	1.00	0.00	N
ATOM	127	H	TYR	9	19.870	9.283	0.520	1.00	0.00	H
ATOM	128	CA	TYR	9	20.453	11.196	-0.181	1.00	0.00	C
ATOM	129	HA	TYR	9	20.597	11.638	-1.167	1.00	0.00	H
ATOM	130	CB	TYR	9	19.401	12.056	0.553	1.00	0.00	C
ATOM	131	HB2	TYR	9	18.481	12.085	-0.029	1.00	0.00	H
ATOM	132	HB3	TYR	9	19.817	13.063	0.565	1.00	0.00	H
ATOM	133	CG	TYR	9	18.791	11.608	1.880	1.00	0.00	C
ATOM	134	CD1	TYR	9	19.157	12.310	3.111	1.00	0.00	C
ATOM	135	HD1	TYR	9	19.866	13.124	3.069	1.00	0.00	H
ATOM	136	CE1	TYR	9	18.642	11.889	4.385	1.00	0.00	C
ATOM	137	HE1	TYR	9	18.894	12.459	5.266	1.00	0.00	H
ATOM	138	CZ	TYR	9	17.645	10.859	4.419	1.00	0.00	C
ATOM	139	OH	TYR	9	17.140	10.514	5.614	1.00	0.00	O
ATOM	140	HH	TYR	9	16.481	9.828	5.492	1.00	0.00	H
ATOM	141	CE2	TYR	9	17.167	10.313	3.213	1.00	0.00	C
ATOM	142	HE2	TYR	9	16.429	9.526	3.211	1.00	0.00	H
ATOM	143	CD2	TYR	9	17.764	10.680	1.962	1.00	0.00	C
ATOM	144	HD2	TYR	9	17.391	10.230	1.054	1.00	0.00	H
ATOM	145	C	TYR	9	21.888	11.300	0.461	1.00	0.00	C
ATOM	146	O	TYR	9	22.447	10.302	0.901	1.00	0.00	O
ATOM	147	N	ARG	10	22.415	12.493	0.515	1.00	0.00	N
ATOM	148	H	ARG	10	21.858	13.310	0.307	1.00	0.00	H
ATOM	149	CA	ARG	10	23.754	12.747	0.996	1.00	0.00	C
ATOM	150	HA	ARG	10	23.984	12.066	1.815	1.00	0.00	H
ATOM	151	CB	ARG	10	24.694	12.349	-0.167	1.00	0.00	C
ATOM	152	HB2	ARG	10	24.382	12.862	-1.077	1.00	0.00	H
ATOM	153	HB3	ARG	10	24.595	11.279	-0.352	1.00	0.00	H
ATOM	154	CG	ARG	10	26.185	12.698	0.054	1.00	0.00	C
ATOM	155	HG2	ARG	10	26.410	13.762	0.123	1.00	0.00	H

ATOM	156	HG3	ARG	10	26.677	12.286	-0.829	1.00	0.00	H
ATOM	157	CD	ARG	10	26.802	11.869	1.251	1.00	0.00	C
ATOM	158	HD2	ARG	10	26.797	10.829	0.929	1.00	0.00	H
ATOM	159	HD3	ARG	10	26.012	11.961	1.996	1.00	0.00	H
ATOM	160	NE	ARG	10	28.144	12.246	1.603	1.00	0.00	N
ATOM	161	HE	ARG	10	28.361	13.229	1.691	1.00	0.00	H
ATOM	162	CZ	ARG	10	29.078	11.382	1.931	1.00	0.00	C
ATOM	163	NH1	ARG	10	28.934	10.113	2.163	1.00	0.00	N
ATOM	164	HH11	ARG	10	28.019	9.708	2.301	1.00	0.00	H
ATOM	165	HH12	ARG	10	29.745	9.514	2.211	1.00	0.00	H
ATOM	166	NH2	ARG	10	30.277	11.787	2.206	1.00	0.00	N
ATOM	167	HH21	ARG	10	30.484	12.740	2.468	1.00	0.00	H
ATOM	168	HH22	ARG	10	30.836	11.136	2.739	1.00	0.00	H
ATOM	169	C	ARG	10	23.936	14.198	1.316	1.00	0.00	C
ATOM	170	O	ARG	10	23.604	15.118	0.520	1.00	0.00	O
ATOM	171	N	GLU	11	24.451	14.421	2.483	1.00	0.00	N
ATOM	172	H	GLU	11	24.493	13.625	3.103	1.00	0.00	H
ATOM	173	CA	GLU	11	24.865	15.725	2.983	1.00	0.00	C
ATOM	174	HA	GLU	11	24.334	16.526	2.468	1.00	0.00	H
ATOM	175	CB	GLU	11	24.400	15.944	4.528	1.00	0.00	C
ATOM	176	HB2	GLU	11	23.316	15.847	4.462	1.00	0.00	H
ATOM	177	HB3	GLU	11	24.702	16.951	4.817	1.00	0.00	H
ATOM	178	CG	GLU	11	24.899	15.032	5.685	1.00	0.00	C
ATOM	179	HG2	GLU	11	24.464	15.437	6.600	1.00	0.00	H
ATOM	180	HG3	GLU	11	25.966	15.210	5.555	1.00	0.00	H
ATOM	181	CD	GLU	11	24.529	13.555	5.600	1.00	0.00	C
ATOM	182	OE1	GLU	11	25.145	12.837	4.731	1.00	0.00	O
ATOM	183	OE2	GLU	11	23.564	13.063	6.211	1.00	0.00	O
ATOM	184	C	GLU	11	26.378	16.043	2.799	1.00	0.00	C
ATOM	185	O	GLU	11	27.164	15.205	2.372	1.00	0.00	O
ATOM	186	N	ASN	12	26.785	17.216	3.332	1.00	0.00	N
ATOM	187	H	ASN	12	26.098	17.867	3.686	1.00	0.00	H
ATOM	188	CA	ASN	12	28.069	17.813	3.078	1.00	0.00	C
ATOM	189	HA	ASN	12	28.388	17.566	2.065	1.00	0.00	H
ATOM	190	CB	ASN	12	27.857	19.254	3.061	1.00	0.00	C
ATOM	191	HB2	ASN	12	27.001	19.600	2.481	1.00	0.00	H
ATOM	192	HB3	ASN	12	28.712	19.637	2.505	1.00	0.00	H
ATOM	193	CG	ASN	12	27.777	20.022	4.445	1.00	0.00	C
ATOM	194	OD1	ASN	12	27.603	19.462	5.490	1.00	0.00	O
ATOM	195	ND2	ASN	12	28.012	21.305	4.444	1.00	0.00	N
ATOM	196	HD21	ASN	12	27.785	21.850	5.263	1.00	0.00	H
ATOM	197	HD22	ASN	12	27.819	21.704	3.537	1.00	0.00	H
ATOM	198	C	ASN	12	29.230	17.342	3.955	1.00	0.00	C
ATOM	199	O	ASN	12	30.322	17.946	4.006	1.00	0.00	O
ATOM	200	N	MET	13	29.085	16.251	4.698	1.00	0.00	N
ATOM	201	H	MET	13	28.286	15.669	4.492	1.00	0.00	H
ATOM	202	CA	MET	13	30.046	15.715	5.639	1.00	0.00	C
ATOM	203	HA	MET	13	30.949	16.319	5.730	1.00	0.00	H
ATOM	204	CB	MET	13	29.403	15.645	7.045	1.00	0.00	C
ATOM	205	HB2	MET	13	29.932	14.933	7.677	1.00	0.00	H
ATOM	206	HB3	MET	13	28.392	15.247	6.972	1.00	0.00	H
ATOM	207	CG	MET	13	29.288	17.010	7.819	1.00	0.00	C
ATOM	208	HG2	MET	13	28.750	17.808	7.309	1.00	0.00	H
ATOM	209	HG3	MET	13	30.336	17.282	7.949	1.00	0.00	H
ATOM	210	SD	MET	13	28.546	16.926	9.484	1.00	0.00	S
ATOM	211	CE	MET	13	26.908	16.397	9.110	1.00	0.00	C
ATOM	212	HE1	MET	13	26.907	15.478	8.523	1.00	0.00	H
ATOM	213	HE2	MET	13	26.316	16.185	10.000	1.00	0.00	H
ATOM	214	HE3	MET	13	26.526	17.199	8.478	1.00	0.00	H
ATOM	215	C	MET	13	30.647	14.383	5.199	1.00	0.00	C
ATOM	216	O	MET	13	30.202	13.724	4.252	1.00	0.00	O
ATOM	217	N	TYR	14	31.746	13.952	5.849	1.00	0.00	N
ATOM	218	H	TYR	14	32.060	14.525	6.620	1.00	0.00	H

ATOM	219	CA	TYR	14	32.494	12.785	5.403	1.00	0.00	C
ATOM	220	HA	TYR	14	31.968	12.397	4.531	1.00	0.00	H
ATOM	221	CB	TYR	14	33.963	13.090	4.919	1.00	0.00	C
ATOM	222	HB2	TYR	14	34.593	12.207	4.813	1.00	0.00	H
ATOM	223	HB3	TYR	14	34.377	13.816	5.619	1.00	0.00	H
ATOM	224	CG	TYR	14	33.967	13.674	3.521	1.00	0.00	C
ATOM	225	CD1	TYR	14	34.074	15.106	3.432	1.00	0.00	C
ATOM	226	HD1	TYR	14	33.944	15.681	4.337	1.00	0.00	H
ATOM	227	CE1	TYR	14	34.359	15.806	2.201	1.00	0.00	C
ATOM	228	HE1	TYR	14	34.319	16.884	2.143	1.00	0.00	H
ATOM	229	CZ	TYR	14	34.451	15.071	0.993	1.00	0.00	C
ATOM	230	OH	TYR	14	34.548	15.729	-0.209	1.00	0.00	O
ATOM	231	HH	TYR	14	34.760	16.661	-0.119	1.00	0.00	H
ATOM	232	CE2	TYR	14	34.304	13.645	1.056	1.00	0.00	C
ATOM	233	HE2	TYR	14	34.535	13.100	0.153	1.00	0.00	H
ATOM	234	CD2	TYR	14	34.068	12.917	2.310	1.00	0.00	C
ATOM	235	HD2	TYR	14	34.141	11.839	2.352	1.00	0.00	H
ATOM	236	C	TYR	14	32.567	11.728	6.503	1.00	0.00	C
ATOM	237	O	TYR	14	32.689	12.153	7.646	1.00	0.00	O
ATOM	238	N	ARG	15	32.542	10.437	6.127	1.00	0.00	N
ATOM	239	H	ARG	15	32.418	10.245	5.142	1.00	0.00	H
ATOM	240	CA	ARG	15	32.747	9.289	7.054	1.00	0.00	C
ATOM	241	HA	ARG	15	32.069	9.579	7.858	1.00	0.00	H
ATOM	242	CB	ARG	15	32.267	7.976	6.580	1.00	0.00	C
ATOM	243	HB2	ARG	15	32.452	7.155	7.271	1.00	0.00	H
ATOM	244	HB3	ARG	15	32.823	7.819	5.656	1.00	0.00	H
ATOM	245	CG	ARG	15	30.808	7.874	6.338	1.00	0.00	C
ATOM	246	HG2	ARG	15	30.440	8.601	5.614	1.00	0.00	H
ATOM	247	HG3	ARG	15	30.456	7.982	7.364	1.00	0.00	H
ATOM	248	CD	ARG	15	30.450	6.536	5.748	1.00	0.00	C
ATOM	249	HD2	ARG	15	30.864	5.711	6.329	1.00	0.00	H
ATOM	250	HD3	ARG	15	30.807	6.514	4.718	1.00	0.00	H
ATOM	251	NE	ARG	15	29.006	6.323	5.753	1.00	0.00	N
ATOM	252	HE	ARG	15	28.610	5.826	6.539	1.00	0.00	H
ATOM	253	CZ	ARG	15	28.151	6.600	4.866	1.00	0.00	C
ATOM	254	NH1	ARG	15	28.408	7.354	3.767	1.00	0.00	N
ATOM	255	HH11	ARG	15	29.318	7.771	3.624	1.00	0.00	H
ATOM	256	HH12	ARG	15	27.698	7.698	3.138	1.00	0.00	H
ATOM	257	NH2	ARG	15	26.882	6.194	4.876	1.00	0.00	N
ATOM	258	HH21	ARG	15	26.448	5.776	5.687	1.00	0.00	H
ATOM	259	HH22	ARG	15	26.286	6.570	4.155	1.00	0.00	H
ATOM	260	C	ARG	15	34.139	9.250	7.722	1.00	0.00	C
ATOM	261	O	ARG	15	34.277	8.636	8.755	1.00	0.00	O
ATOM	262	N	TYR	16	35.151	9.732	7.018	1.00	0.00	N
ATOM	263	H	TYR	16	34.988	10.024	6.065	1.00	0.00	H
ATOM	264	CA	TYR	16	36.557	9.644	7.396	1.00	0.00	C
ATOM	265	HA	TYR	16	36.786	8.702	7.897	1.00	0.00	H
ATOM	266	CB	TYR	16	37.374	9.592	6.100	1.00	0.00	C
ATOM	267	HB2	TYR	16	37.013	8.779	5.470	1.00	0.00	H
ATOM	268	HB3	TYR	16	38.354	9.397	6.533	1.00	0.00	H
ATOM	269	CG	TYR	16	37.473	10.842	5.209	1.00	0.00	C
ATOM	270	CD1	TYR	16	38.373	11.741	5.664	1.00	0.00	C
ATOM	271	HD1	TYR	16	38.936	11.601	6.575	1.00	0.00	H
ATOM	272	CE1	TYR	16	38.492	12.970	4.959	1.00	0.00	C
ATOM	273	HE1	TYR	16	39.126	13.762	5.333	1.00	0.00	H
ATOM	274	CZ	TYR	16	37.788	13.145	3.763	1.00	0.00	C
ATOM	275	OH	TYR	16	37.857	14.335	3.120	1.00	0.00	O
ATOM	276	HH	TYR	16	37.423	14.274	2.266	1.00	0.00	H
ATOM	277	CE2	TYR	16	36.950	12.140	3.283	1.00	0.00	C
ATOM	278	HE2	TYR	16	36.498	12.230	2.306	1.00	0.00	H
ATOM	279	CD2	TYR	16	36.737	10.983	4.055	1.00	0.00	C
ATOM	280	HD2	TYR	16	36.227	10.085	3.737	1.00	0.00	H
ATOM	281	C	TYR	16	36.945	10.840	8.307	1.00	0.00	C

ATOM	282	O	TYR	16	36.418	11.921	8.147	1.00	0.00	O
ATOM	283	N	NME	17	37.900	10.703	9.192	1.00	0.00	N
ATOM	284	H	NME	17	38.444	9.852	9.196	1.00	0.00	H
ATOM	285	CH3	NME	17	38.551	11.894	9.913	1.00	0.00	C
ATOM	286	HH31	NME	17	39.597	11.752	10.185	1.00	0.00	H
ATOM	287	HH32	NME	17	38.591	12.692	9.173	1.00	0.00	H
ATOM	288	HH33	NME	17	37.930	12.110	10.782	1.00	0.00	H
TER	289		NME	17						
ATOM	289	HH31	ACE	18	39.868	7.702	4.476	1.00	0.00	H
ATOM	290	CH3	ACE	18	40.599	8.448	4.787	1.00	0.00	C
ATOM	291	HH32	ACE	18	40.111	9.411	4.636	1.00	0.00	H
ATOM	292	HH33	ACE	18	41.419	8.317	4.082	1.00	0.00	H
ATOM	293	C	ACE	18	41.120	8.328	6.221	1.00	0.00	C
ATOM	294	O	ACE	18	41.550	9.327	6.842	1.00	0.00	O
ATOM	295	N	HIE	19	41.024	7.089	6.762	1.00	0.00	N
ATOM	296	H	HIE	19	40.734	6.381	6.102	1.00	0.00	H
ATOM	297	CA	HIE	19	41.488	6.664	8.092	1.00	0.00	C
ATOM	298	HA	HIE	19	42.282	7.325	8.438	1.00	0.00	H
ATOM	299	CB	HIE	19	40.306	6.665	9.045	1.00	0.00	C
ATOM	300	HB2	HIE	19	39.582	5.864	8.906	1.00	0.00	H
ATOM	301	HB3	HIE	19	39.846	7.643	8.895	1.00	0.00	H
ATOM	302	CG	HIE	19	40.441	6.554	10.544	1.00	0.00	C
ATOM	303	ND1	HIE	19	41.259	5.642	11.203	1.00	0.00	N
ATOM	304	CE1	HIE	19	40.910	5.686	12.491	1.00	0.00	C
ATOM	305	HE1	HIE	19	41.387	5.103	13.264	1.00	0.00	H
ATOM	306	NE2	HIE	19	39.946	6.620	12.692	1.00	0.00	N
ATOM	307	HE2	HIE	19	39.438	6.823	13.541	1.00	0.00	H
ATOM	308	CD2	HIE	19	39.638	7.135	11.455	1.00	0.00	C
ATOM	309	HD2	HIE	19	38.874	7.871	11.250	1.00	0.00	H
ATOM	310	C	HIE	19	42.115	5.273	7.954	1.00	0.00	C
ATOM	311	O	HIE	19	41.794	4.393	7.051	1.00	0.00	O
ATOM	312	N	SER	20	43.094	5.037	8.781	1.00	0.00	N
ATOM	313	H	SER	20	43.240	5.818	9.406	1.00	0.00	H
ATOM	314	CA	SER	20	43.806	3.769	8.974	1.00	0.00	C
ATOM	315	HA	SER	20	44.122	3.421	7.990	1.00	0.00	H
ATOM	316	CB	SER	20	45.063	3.957	9.930	1.00	0.00	C
ATOM	317	HB2	SER	20	45.434	2.970	10.206	1.00	0.00	H
ATOM	318	HB3	SER	20	44.630	4.342	10.852	1.00	0.00	H
ATOM	319	OG	SER	20	46.071	4.963	9.419	1.00	0.00	O
ATOM	320	HG	SER	20	46.845	4.926	9.986	1.00	0.00	H
ATOM	321	C	SER	20	42.970	2.629	9.573	1.00	0.00	C
ATOM	322	O	SER	20	43.413	1.509	9.630	1.00	0.00	O
ATOM	323	N	GLN	21	41.698	2.915	9.989	1.00	0.00	N
ATOM	324	H	GLN	21	41.421	3.873	9.835	1.00	0.00	H
ATOM	325	CA	GLN	21	40.573	1.957	10.443	1.00	0.00	C
ATOM	326	HA	GLN	21	40.893	0.953	10.170	1.00	0.00	H
ATOM	327	CB	GLN	21	40.390	1.956	11.957	1.00	0.00	C
ATOM	328	HB2	GLN	21	39.673	1.200	12.277	1.00	0.00	H
ATOM	329	HB3	GLN	21	40.069	2.973	12.183	1.00	0.00	H
ATOM	330	CG	GLN	21	41.666	1.695	12.720	1.00	0.00	C
ATOM	331	HG2	GLN	21	42.468	2.362	12.404	1.00	0.00	H
ATOM	332	HG3	GLN	21	41.873	0.634	12.580	1.00	0.00	H
ATOM	333	CD	GLN	21	41.559	1.854	14.291	1.00	0.00	C
ATOM	334	OE1	GLN	21	40.754	2.500	14.950	1.00	0.00	O
ATOM	335	NE2	GLN	21	42.365	1.035	15.013	1.00	0.00	N
ATOM	336	HE21	GLN	21	42.450	1.244	15.997	1.00	0.00	H
ATOM	337	HE22	GLN	21	42.851	0.247	14.608	1.00	0.00	H
ATOM	338	C	GLN	21	39.180	2.179	9.793	1.00	0.00	C
ATOM	339	O	GLN	21	38.157	1.701	10.311	1.00	0.00	O
ATOM	340	N	TRP	22	39.176	2.833	8.649	1.00	0.00	N
ATOM	341	H	TRP	22	40.067	2.992	8.200	1.00	0.00	H
ATOM	342	CA	TRP	22	37.970	2.881	7.738	1.00	0.00	C
ATOM	343	HA	TRP	22	37.084	3.193	8.291	1.00	0.00	H

ATOM	344	CB	TRP	22	38.122	4.071	6.770	1.00	0.00	C
ATOM	345	HB2	TRP	22	38.845	3.882	5.977	1.00	0.00	H
ATOM	346	HB3	TRP	22	38.424	4.910	7.398	1.00	0.00	H
ATOM	347	CG	TRP	22	37.017	4.602	5.993	1.00	0.00	C
ATOM	348	CD1	TRP	22	36.102	5.496	6.433	1.00	0.00	C
ATOM	349	HD1	TRP	22	36.153	5.873	7.444	1.00	0.00	H
ATOM	350	NE1	TRP	22	35.054	5.611	5.473	1.00	0.00	N
ATOM	351	HE1	TRP	22	34.194	6.123	5.605	1.00	0.00	H
ATOM	352	CE2	TRP	22	35.209	4.716	4.443	1.00	0.00	C
ATOM	353	CZ2	TRP	22	34.364	4.301	3.418	1.00	0.00	C
ATOM	354	HZ2	TRP	22	33.431	4.806	3.218	1.00	0.00	H
ATOM	355	CH2	TRP	22	34.798	3.246	2.555	1.00	0.00	C
ATOM	356	HH2	TRP	22	34.315	2.871	1.665	1.00	0.00	H
ATOM	357	CZ3	TRP	22	36.057	2.688	2.775	1.00	0.00	C
ATOM	358	HZ3	TRP	22	36.302	1.854	2.133	1.00	0.00	H
ATOM	359	CE3	TRP	22	36.853	2.980	3.881	1.00	0.00	C
ATOM	360	HE3	TRP	22	37.728	2.406	4.150	1.00	0.00	H
ATOM	361	CD2	TRP	22	36.447	4.014	4.747	1.00	0.00	C
ATOM	362	C	TRP	22	37.717	1.647	6.953	1.00	0.00	C
ATOM	363	O	TRP	22	38.635	1.128	6.269	1.00	0.00	O
ATOM	364	N	ASN	23	36.520	1.089	7.021	1.00	0.00	N
ATOM	365	H	ASN	23	35.862	1.359	7.739	1.00	0.00	H
ATOM	366	CA	ASN	23	36.095	0.055	6.214	1.00	0.00	C
ATOM	367	HA	ASN	23	36.643	0.067	5.272	1.00	0.00	H
ATOM	368	CB	ASN	23	36.145	-1.246	7.095	1.00	0.00	C
ATOM	369	HB2	ASN	23	35.357	-1.134	7.839	1.00	0.00	H
ATOM	370	HB3	ASN	23	37.086	-1.193	7.642	1.00	0.00	H
ATOM	371	CG	ASN	23	35.903	-2.569	6.314	1.00	0.00	C
ATOM	372	OD1	ASN	23	36.836	-3.097	5.710	1.00	0.00	O
ATOM	373	ND2	ASN	23	34.715	-3.146	6.355	1.00	0.00	N
ATOM	374	HD21	ASN	23	34.571	-3.949	5.758	1.00	0.00	H
ATOM	375	HD22	ASN	23	33.912	-2.757	6.827	1.00	0.00	H
ATOM	376	C	ASN	23	34.601	0.378	5.869	1.00	0.00	C
ATOM	377	O	ASN	23	34.037	1.331	6.517	1.00	0.00	O
ATOM	378	N	LYS	24	33.963	-0.241	4.866	1.00	0.00	N
ATOM	379	H	LYS	24	34.488	-0.876	4.283	1.00	0.00	H
ATOM	380	CA	LYS	24	32.531	-0.042	4.447	1.00	0.00	C
ATOM	381	HA	LYS	24	32.541	0.899	3.896	1.00	0.00	H
ATOM	382	CB	LYS	24	32.154	-1.204	3.436	1.00	0.00	C
ATOM	383	HB2	LYS	24	31.093	-1.436	3.524	1.00	0.00	H
ATOM	384	HB3	LYS	24	32.690	-2.082	3.798	1.00	0.00	H
ATOM	385	CG	LYS	24	32.583	-0.847	1.969	1.00	0.00	C
ATOM	386	HG2	LYS	24	33.666	-0.764	1.875	1.00	0.00	H
ATOM	387	HG3	LYS	24	32.060	0.105	1.877	1.00	0.00	H
ATOM	388	CD	LYS	24	32.059	-1.841	0.941	1.00	0.00	C
ATOM	389	HD2	LYS	24	32.499	-2.834	1.030	1.00	0.00	H
ATOM	390	HD3	LYS	24	32.470	-1.281	0.101	1.00	0.00	H
ATOM	391	CE	LYS	24	30.496	-1.874	0.892	1.00	0.00	C
ATOM	392	HE2	LYS	24	30.055	-0.881	0.810	1.00	0.00	H
ATOM	393	HE3	LYS	24	30.139	-2.304	1.828	1.00	0.00	H
ATOM	394	NZ	LYS	24	29.955	-2.655	-0.214	1.00	0.00	N
ATOM	395	HZ1	LYS	24	28.949	-2.648	-0.128	1.00	0.00	H
ATOM	396	HZ2	LYS	24	30.178	-2.205	-1.089	1.00	0.00	H
ATOM	397	HZ3	LYS	24	30.265	-3.612	-0.116	1.00	0.00	H
ATOM	398	C	LYS	24	31.574	0.163	5.703	1.00	0.00	C
ATOM	399	O	LYS	24	31.684	-0.539	6.644	1.00	0.00	O
ATOM	400	N	PRO	25	30.505	1.038	5.590	1.00	0.00	N
ATOM	401	CD	PRO	25	30.092	1.739	4.374	1.00	0.00	C
ATOM	402	HD2	PRO	25	30.973	2.238	3.970	1.00	0.00	H
ATOM	403	HD3	PRO	25	29.641	1.129	3.592	1.00	0.00	H
ATOM	404	CG	PRO	25	29.082	2.799	4.845	1.00	0.00	C
ATOM	405	HG2	PRO	25	29.438	3.801	4.604	1.00	0.00	H
ATOM	406	HG3	PRO	25	28.046	2.588	4.583	1.00	0.00	H

ATOM	407	CB	PRO	25	29.081	2.854	6.371	1.00	0.00	C
ATOM	408	HB2	PRO	25	29.670	3.669	6.793	1.00	0.00	H
ATOM	409	HB3	PRO	25	28.102	3.052	6.805	1.00	0.00	H
ATOM	410	CA	PRO	25	29.697	1.444	6.785	1.00	0.00	C
ATOM	411	HA	PRO	25	30.314	1.529	7.681	1.00	0.00	H
ATOM	412	C	PRO	25	28.727	0.381	7.190	1.00	0.00	C
ATOM	413	O	PRO	25	27.982	-0.085	6.329	1.00	0.00	O
ATOM	414	N	SER	26	28.697	0.003	8.514	1.00	0.00	N
ATOM	415	H	SER	26	29.354	0.452	9.137	1.00	0.00	H
ATOM	416	CA	SER	26	27.775	-1.027	9.222	1.00	0.00	C
ATOM	417	HA	SER	26	27.854	-1.996	8.727	1.00	0.00	H
ATOM	418	CB	SER	26	28.053	-1.163	10.689	1.00	0.00	C
ATOM	419	HB2	SER	26	27.339	-1.866	11.116	1.00	0.00	H
ATOM	420	HB3	SER	26	27.746	-0.199	11.093	1.00	0.00	H
ATOM	421	OG	SER	26	29.403	-1.583	11.019	1.00	0.00	O
ATOM	422	HG	SER	26	29.419	-1.923	11.918	1.00	0.00	H
ATOM	423	C	SER	26	26.252	-0.730	9.138	1.00	0.00	C
ATOM	424	O	SER	26	25.446	-1.606	9.441	1.00	0.00	O
ATOM	425	N	LYS	27	25.834	0.483	8.713	1.00	0.00	N
ATOM	426	H	LYS	27	26.556	1.003	8.236	1.00	0.00	H
ATOM	427	CA	LYS	27	24.470	1.095	8.715	1.00	0.00	C
ATOM	428	HA	LYS	27	23.698	0.392	8.401	1.00	0.00	H
ATOM	429	CB	LYS	27	24.174	1.612	10.156	1.00	0.00	C
ATOM	430	HB2	LYS	27	24.919	2.381	10.359	1.00	0.00	H
ATOM	431	HB3	LYS	27	24.290	0.690	10.725	1.00	0.00	H
ATOM	432	CG	LYS	27	22.795	2.203	10.429	1.00	0.00	C
ATOM	433	HG2	LYS	27	22.540	2.870	9.606	1.00	0.00	H
ATOM	434	HG3	LYS	27	22.953	2.819	11.315	1.00	0.00	H
ATOM	435	CD	LYS	27	21.688	1.251	10.627	1.00	0.00	C
ATOM	436	HD2	LYS	27	21.626	0.560	9.786	1.00	0.00	H
ATOM	437	HD3	LYS	27	20.778	1.853	10.619	1.00	0.00	H
ATOM	438	CE	LYS	27	21.722	0.366	11.895	1.00	0.00	C
ATOM	439	HE2	LYS	27	22.036	0.957	12.755	1.00	0.00	H
ATOM	440	HE3	LYS	27	22.497	-0.379	11.711	1.00	0.00	H
ATOM	441	NZ	LYS	27	20.431	-0.302	12.271	1.00	0.00	N
ATOM	442	HZ1	LYS	27	20.617	-0.975	13.000	1.00	0.00	H
ATOM	443	HZ2	LYS	27	19.830	0.432	12.619	1.00	0.00	H
ATOM	444	HZ3	LYS	27	19.986	-0.659	11.438	1.00	0.00	H
ATOM	445	C	LYS	27	24.506	2.261	7.692	1.00	0.00	C
ATOM	446	O	LYS	27	25.455	3.094	7.742	1.00	0.00	O
ATOM	447	N	PRO	28	23.408	2.491	6.920	1.00	0.00	N
ATOM	448	CD	PRO	28	22.194	1.729	6.835	1.00	0.00	C
ATOM	449	HD2	PRO	28	22.469	0.677	6.921	1.00	0.00	H
ATOM	450	HD3	PRO	28	21.413	2.025	7.536	1.00	0.00	H
ATOM	451	CG	PRO	28	21.642	1.967	5.454	1.00	0.00	C
ATOM	452	HG2	PRO	28	22.202	1.246	4.858	1.00	0.00	H
ATOM	453	HG3	PRO	28	20.591	1.714	5.312	1.00	0.00	H
ATOM	454	CB	PRO	28	21.989	3.435	5.163	1.00	0.00	C
ATOM	455	HB2	PRO	28	22.050	3.563	4.082	1.00	0.00	H
ATOM	456	HB3	PRO	28	21.263	4.112	5.613	1.00	0.00	H
ATOM	457	CA	PRO	28	23.315	3.564	5.877	1.00	0.00	C
ATOM	458	HA	PRO	28	24.143	3.405	5.187	1.00	0.00	H
ATOM	459	C	PRO	28	23.624	5.024	6.616	1.00	0.00	C
ATOM	460	O	PRO	28	24.509	5.771	6.131	1.00	0.00	O
ATOM	461	N	LYS	29	23.047	5.290	7.779	1.00	0.00	N
ATOM	462	H	LYS	29	22.362	4.663	8.178	1.00	0.00	H
ATOM	463	CA	LYS	29	23.075	6.637	8.420	1.00	0.00	C
ATOM	464	HA	LYS	29	23.301	7.388	7.664	1.00	0.00	H
ATOM	465	CB	LYS	29	21.701	7.033	8.939	1.00	0.00	C
ATOM	466	HB2	LYS	29	21.379	6.416	9.780	1.00	0.00	H
ATOM	467	HB3	LYS	29	21.092	6.882	8.048	1.00	0.00	H
ATOM	468	CG	LYS	29	21.572	8.573	9.189	1.00	0.00	C
ATOM	469	HG2	LYS	29	21.974	9.176	8.374	1.00	0.00	H

ATOM	470	HG3	LYS	29	22.138	8.705	10.112	1.00	0.00	H
ATOM	471	CD	LYS	29	20.188	9.062	9.374	1.00	0.00	C
ATOM	472	HD2	LYS	29	19.591	8.328	9.915	1.00	0.00	H
ATOM	473	HD3	LYS	29	19.874	9.204	8.339	1.00	0.00	H
ATOM	474	CE	LYS	29	20.176	10.402	10.153	1.00	0.00	C
ATOM	475	HE2	LYS	29	20.208	11.260	9.481	1.00	0.00	H
ATOM	476	HE3	LYS	29	21.072	10.338	10.771	1.00	0.00	H
ATOM	477	NZ	LYS	29	19.039	10.417	11.088	1.00	0.00	N
ATOM	478	HZ1	LYS	29	18.939	11.142	11.784	1.00	0.00	H
ATOM	479	HZ2	LYS	29	18.138	10.444	10.632	1.00	0.00	H
ATOM	480	HZ3	LYS	29	19.019	9.659	11.756	1.00	0.00	H
ATOM	481	C	LYS	29	24.268	6.714	9.397	1.00	0.00	C
ATOM	482	O	LYS	29	24.130	6.636	10.599	1.00	0.00	O
ATOM	483	N	THR	30	25.445	6.805	8.821	1.00	0.00	N
ATOM	484	H	THR	30	25.368	6.559	7.844	1.00	0.00	H
ATOM	485	CA	THR	30	26.781	6.906	9.473	1.00	0.00	C
ATOM	486	HA	THR	30	26.698	6.943	10.559	1.00	0.00	H
ATOM	487	CB	THR	30	27.606	5.668	8.972	1.00	0.00	C
ATOM	488	HB	THR	30	28.641	6.007	8.931	1.00	0.00	H
ATOM	489	CG2	THR	30	27.417	4.524	9.929	1.00	0.00	C
ATOM	490	HG21	THR	30	27.729	4.931	10.891	1.00	0.00	H
ATOM	491	HG22	THR	30	26.361	4.255	9.952	1.00	0.00	H
ATOM	492	HG23	THR	30	28.125	3.726	9.699	1.00	0.00	H
ATOM	493	OG1	THR	30	27.220	5.274	7.665	1.00	0.00	O
ATOM	494	HG1	THR	30	26.779	4.425	7.752	1.00	0.00	H
ATOM	495	C	THR	30	27.493	8.213	9.075	1.00	0.00	C
ATOM	496	O	THR	30	28.428	8.630	9.702	1.00	0.00	O
ATOM	497	N	ASN	31	27.061	8.916	8.077	1.00	0.00	N
ATOM	498	H	ASN	31	26.527	8.447	7.360	1.00	0.00	H
ATOM	499	CA	ASN	31	27.692	10.173	7.654	1.00	0.00	C
ATOM	500	HA	ASN	31	28.757	9.942	7.659	1.00	0.00	H
ATOM	501	CB	ASN	31	27.337	10.471	6.239	1.00	0.00	C
ATOM	502	HB2	ASN	31	26.398	11.023	6.194	1.00	0.00	H
ATOM	503	HB3	ASN	31	27.385	9.474	5.802	1.00	0.00	H
ATOM	504	CG	ASN	31	28.401	11.175	5.444	1.00	0.00	C
ATOM	505	OD1	ASN	31	29.513	10.765	5.335	1.00	0.00	O
ATOM	506	ND2	ASN	31	27.977	12.265	4.972	1.00	0.00	N
ATOM	507	HD21	ASN	31	28.705	12.753	4.470	1.00	0.00	H
ATOM	508	HD22	ASN	31	27.004	12.530	4.914	1.00	0.00	H
ATOM	509	C	ASN	31	27.325	11.439	8.499	1.00	0.00	C
ATOM	510	O	ASN	31	27.965	12.491	8.249	1.00	0.00	O
ATOM	511	N	MET	32	26.352	11.350	9.456	1.00	0.00	N
ATOM	512	H	MET	32	25.949	10.435	9.593	1.00	0.00	H
ATOM	513	CA	MET	32	25.865	12.545	10.172	1.00	0.00	C
ATOM	514	HA	MET	32	25.953	13.370	9.464	1.00	0.00	H
ATOM	515	CB	MET	32	24.362	12.465	10.499	1.00	0.00	C
ATOM	516	HB2	MET	32	23.812	11.995	9.685	1.00	0.00	H
ATOM	517	HB3	MET	32	24.002	13.454	10.785	1.00	0.00	H
ATOM	518	CG	MET	32	24.070	11.495	11.641	1.00	0.00	C
ATOM	519	HG2	MET	32	22.990	11.357	11.710	1.00	0.00	H
ATOM	520	HG3	MET	32	24.381	11.951	12.580	1.00	0.00	H
ATOM	521	SD	MET	32	24.735	9.825	11.615	1.00	0.00	S
ATOM	522	CE	MET	32	23.933	9.164	13.030	1.00	0.00	C
ATOM	523	HE1	MET	32	24.095	9.838	13.871	1.00	0.00	H
ATOM	524	HE2	MET	32	22.862	9.110	12.835	1.00	0.00	H
ATOM	525	HE3	MET	32	24.304	8.190	13.348	1.00	0.00	H
ATOM	526	C	MET	32	26.757	13.040	11.378	1.00	0.00	C
ATOM	527	O	MET	32	26.488	14.140	11.871	1.00	0.00	O
ATOM	528	N	LYS	33	27.735	12.322	11.938	1.00	0.00	N
ATOM	529	H	LYS	33	27.721	11.359	11.633	1.00	0.00	H
ATOM	530	CA	LYS	33	28.589	12.766	13.044	1.00	0.00	C
ATOM	531	HA	LYS	33	28.101	13.522	13.656	1.00	0.00	H
ATOM	532	CB	LYS	33	29.063	11.529	13.833	1.00	0.00	C

```
ATOM    533  HB2 LYS    33      30.020  11.721  14.319  1.00  0.00           H
ATOM    534  HB3 LYS    33      29.235  10.798  13.043  1.00  0.00           H
ATOM    535  CG  LYS    33      28.175  11.004  14.937  1.00  0.00           C
ATOM    536  HG2 LYS    33      28.527  10.020  15.251  1.00  0.00           H
ATOM    537  HG3 LYS    33      27.211  10.820  14.461  1.00  0.00           H
ATOM    538  CD  LYS    33      28.230  12.009  16.090  1.00  0.00           C
ATOM    539  HD2 LYS    33      27.933  13.048  15.949  1.00  0.00           H
ATOM    540  HD3 LYS    33      29.269  12.025  16.421  1.00  0.00           H
ATOM    541  CE  LYS    33      27.270  11.645  17.196  1.00  0.00           C
ATOM    542  HE2 LYS    33      26.245  11.560  16.834  1.00  0.00           H
ATOM    543  HE3 LYS    33      27.348  12.448  17.928  1.00  0.00           H
ATOM    544  NZ  LYS    33      27.489  10.324  17.924  1.00  0.00           N
ATOM    545  HZ1 LYS    33      28.469  10.124  18.073  1.00  0.00           H
ATOM    546  HZ2 LYS    33      26.931  10.264  18.763  1.00  0.00           H
ATOM    547  HZ3 LYS    33      27.166   9.572  17.333  1.00  0.00           H
ATOM    548  C   LYS    33      29.765  13.610  12.533  1.00  0.00           C
ATOM    549  O   LYS    33      30.255  14.452  13.317  1.00  0.00           O
ATOM    550  N   NME    34      30.264  13.360  11.275  1.00  0.00           N
ATOM    551  H   NME    34      29.986  12.562  10.722  1.00  0.00           H
ATOM    552  CH3 NME    34      31.168  14.227  10.547  1.00  0.00           C
ATOM    553 HH31 NME    34      30.741  15.230  10.526  1.00  0.00           H
ATOM    554 HH32 NME    34      32.135  14.232  11.050  1.00  0.00           H
ATOM    555 HH33 NME    34      31.456  13.865   9.559  1.00  0.00           H
TER     556      NME    34
END
```

Cluster 1, Figure 5B:

```
ATOM      1 HH31 ACE     1      26.362  14.583  -3.363  1.00  0.00           H
ATOM      2  CH3 ACE     1      26.612  13.538  -3.181  1.00  0.00           C
ATOM      3 HH32 ACE     1      27.288  13.174  -3.955  1.00  0.00           H
ATOM      4 HH33 ACE     1      25.694  12.988  -3.388  1.00  0.00           H
ATOM      5  C   ACE     1      27.164  13.267  -1.855  1.00  0.00           C
ATOM      6  O   ACE     1      28.366  13.100  -1.691  1.00  0.00           O
ATOM      7  N   ASN     2      26.227  13.303  -0.913  1.00  0.00           N
ATOM      8  H   ASN     2      25.337  13.756  -1.064  1.00  0.00           H
ATOM      9  CA  ASN     2      26.601  13.005   0.473  1.00  0.00           C
ATOM     10  HA  ASN     2      27.682  12.879   0.529  1.00  0.00           H
ATOM     11  CB  ASN     2      26.299  14.307   1.298  1.00  0.00           C
ATOM     12  HB2 ASN     2      25.260  14.610   1.171  1.00  0.00           H
ATOM     13  HB3 ASN     2      26.956  15.105   0.952  1.00  0.00           H
ATOM     14  CG  ASN     2      26.518  14.208   2.799  1.00  0.00           C
ATOM     15  OD1 ASN     2      26.789  13.122   3.361  1.00  0.00           O
ATOM     16  ND2 ASN     2      26.537  15.361   3.538  1.00  0.00           N
ATOM     17 HD21 ASN     2      26.592  15.273   4.543  1.00  0.00           H
ATOM     18 HD22 ASN     2      26.076  16.145   3.098  1.00  0.00           H
ATOM     19  C   ASN     2      25.851  11.793   0.828  1.00  0.00           C
ATOM     20  O   ASN     2      26.446  10.847   1.290  1.00  0.00           O
ATOM     21  N   ASP     3      24.559  11.685   0.629  1.00  0.00           N
ATOM     22  H   ASP     3      24.066  12.529   0.373  1.00  0.00           H
ATOM     23  CA  ASP     3      23.699  10.514   0.838  1.00  0.00           C
ATOM     24  HA  ASP     3      23.943  10.098   1.816  1.00  0.00           H
ATOM     25  CB  ASP     3      22.238  11.041   0.856  1.00  0.00           C
ATOM     26  HB2 ASP     3      21.907  11.298  -0.150  1.00  0.00           H
ATOM     27  HB3 ASP     3      22.274  11.861   1.574  1.00  0.00           H
ATOM     28  CG  ASP     3      21.237  10.088   1.544  1.00  0.00           C
ATOM     29  OD1 ASP     3      20.104   9.812   0.948  1.00  0.00           O
ATOM     30  OD2 ASP     3      21.638   9.485   2.589  1.00  0.00           O
ATOM     31  C   ASP     3      23.817   9.319  -0.146  1.00  0.00           C
ATOM     32  O   ASP     3      23.416   8.123   0.175  1.00  0.00           O
ATOM     33  N   TYR     4      24.364   9.653  -1.343  1.00  0.00           N
```

ATOM	34	H	TYR	4	24.858	10.534	-1.326	1.00	0.00	H
ATOM	35	CA	TYR	4	24.575	8.807	-2.528	1.00	0.00	C
ATOM	36	HA	TYR	4	24.558	7.752	-2.257	1.00	0.00	H
ATOM	37	CB	TYR	4	23.457	9.012	-3.531	1.00	0.00	C
ATOM	38	HB2	TYR	4	23.612	10.037	-3.867	1.00	0.00	H
ATOM	39	HB3	TYR	4	22.512	8.843	-3.013	1.00	0.00	H
ATOM	40	CG	TYR	4	23.519	8.102	-4.723	1.00	0.00	C
ATOM	41	CD1	TYR	4	23.817	8.605	-6.005	1.00	0.00	C
ATOM	42	HD1	TYR	4	23.812	9.681	-6.100	1.00	0.00	H
ATOM	43	CE1	TYR	4	23.689	7.713	-7.124	1.00	0.00	C
ATOM	44	HE1	TYR	4	23.948	7.995	-8.134	1.00	0.00	H
ATOM	45	CZ	TYR	4	23.369	6.400	-6.940	1.00	0.00	C
ATOM	46	OH	TYR	4	23.605	5.509	-7.936	1.00	0.00	O
ATOM	47	HH	TYR	4	23.656	4.557	-7.824	1.00	0.00	H
ATOM	48	CE2	TYR	4	23.059	5.894	-5.665	1.00	0.00	C
ATOM	49	HE2	TYR	4	22.779	4.853	-5.608	1.00	0.00	H
ATOM	50	CD2	TYR	4	23.144	6.756	-4.570	1.00	0.00	C
ATOM	51	HD2	TYR	4	22.987	6.453	-3.546	1.00	0.00	H
ATOM	52	C	TYR	4	26.001	9.091	-3.124	1.00	0.00	C
ATOM	53	O	TYR	4	26.304	10.264	-3.415	1.00	0.00	O
ATOM	54	N	GLU	5	26.780	8.023	-3.283	1.00	0.00	N
ATOM	55	H	GLU	5	26.320	7.142	-3.104	1.00	0.00	H
ATOM	56	CA	GLU	5	28.228	8.085	-3.477	1.00	0.00	C
ATOM	57	HA	GLU	5	28.588	9.022	-3.053	1.00	0.00	H
ATOM	58	CB	GLU	5	28.916	7.098	-2.494	1.00	0.00	C
ATOM	59	HB2	GLU	5	29.920	6.914	-2.876	1.00	0.00	H
ATOM	60	HB3	GLU	5	28.341	6.173	-2.437	1.00	0.00	H
ATOM	61	CG	GLU	5	29.134	7.494	-1.099	1.00	0.00	C
ATOM	62	HG2	GLU	5	29.463	6.627	-0.526	1.00	0.00	H
ATOM	63	HG3	GLU	5	28.193	7.935	-0.770	1.00	0.00	H
ATOM	64	CD	GLU	5	30.244	8.520	-0.983	1.00	0.00	C
ATOM	65	OE1	GLU	5	31.399	7.994	-0.989	1.00	0.00	O
ATOM	66	OE2	GLU	5	30.025	9.740	-0.696	1.00	0.00	O
ATOM	67	C	GLU	5	28.750	7.946	-4.958	1.00	0.00	C
ATOM	68	O	GLU	5	29.866	7.459	-5.208	1.00	0.00	O
ATOM	69	N	ASP	6	27.932	8.446	-5.859	1.00	0.00	N
ATOM	70	H	ASP	6	26.970	8.686	-5.663	1.00	0.00	H
ATOM	71	CA	ASP	6	28.315	8.616	-7.278	1.00	0.00	C
ATOM	72	HA	ASP	6	28.884	7.721	-7.528	1.00	0.00	H
ATOM	73	CB	ASP	6	27.148	8.619	-8.267	1.00	0.00	C
ATOM	74	HB2	ASP	6	26.572	9.535	-8.399	1.00	0.00	H
ATOM	75	HB3	ASP	6	26.451	7.973	-7.734	1.00	0.00	H
ATOM	76	CG	ASP	6	27.516	7.919	-9.673	1.00	0.00	C
ATOM	77	OD1	ASP	6	26.914	6.888	-10.048	1.00	0.00	O
ATOM	78	OD2	ASP	6	28.256	8.529	-10.501	1.00	0.00	O
ATOM	79	C	ASP	6	29.334	9.795	-7.480	1.00	0.00	C
ATOM	80	O	ASP	6	29.153	10.715	-8.312	1.00	0.00	O
ATOM	81	N	ARG	7	30.371	9.799	-6.665	1.00	0.00	N
ATOM	82	H	ARG	7	30.411	9.017	-6.027	1.00	0.00	H
ATOM	83	CA	ARG	7	31.295	10.836	-6.428	1.00	0.00	C
ATOM	84	HA	ARG	7	31.139	11.432	-7.328	1.00	0.00	H
ATOM	85	CB	ARG	7	30.894	11.485	-5.141	1.00	0.00	C
ATOM	86	HB2	ARG	7	30.859	10.792	-4.301	1.00	0.00	H
ATOM	87	HB3	ARG	7	29.952	12.017	-5.276	1.00	0.00	H
ATOM	88	CG	ARG	7	31.778	12.644	-4.790	1.00	0.00	C
ATOM	89	HG2	ARG	7	31.663	13.335	-5.626	1.00	0.00	H
ATOM	90	HG3	ARG	7	32.811	12.319	-4.664	1.00	0.00	H
ATOM	91	CD	ARG	7	31.319	13.429	-3.583	1.00	0.00	C
ATOM	92	HD2	ARG	7	30.316	13.837	-3.710	1.00	0.00	H
ATOM	93	HD3	ARG	7	32.058	14.229	-3.559	1.00	0.00	H
ATOM	94	NE	ARG	7	31.225	12.644	-2.373	1.00	0.00	N
ATOM	95	HE	ARG	7	30.442	12.006	-2.349	1.00	0.00	H
ATOM	96	CZ	ARG	7	31.895	12.752	-1.189	1.00	0.00	C

ATOM	97	NH1	ARG	7	32.810	13.654	-0.945	1.00	0.00	N
ATOM	98	HH11	ARG	7	32.880	14.483	-1.517	1.00	0.00	H
ATOM	99	HH12	ARG	7	33.256	13.687	-0.040	1.00	0.00	H
ATOM	100	NH2	ARG	7	31.700	11.884	-0.232	1.00	0.00	N
ATOM	101	HH21	ARG	7	31.011	11.149	-0.295	1.00	0.00	H
ATOM	102	HH22	ARG	7	32.306	11.943	0.574	1.00	0.00	H
ATOM	103	C	ARG	7	32.722	10.316	-6.352	1.00	0.00	C
ATOM	104	O	ARG	7	32.942	9.292	-5.782	1.00	0.00	O
ATOM	105	N	TYR	8	33.702	11.002	-7.079	1.00	0.00	N
ATOM	106	H	TYR	8	33.404	11.771	-7.661	1.00	0.00	H
ATOM	107	CA	TYR	8	35.071	10.563	-7.235	1.00	0.00	C
ATOM	108	HA	TYR	8	35.073	9.476	-7.308	1.00	0.00	H
ATOM	109	CB	TYR	8	35.703	11.247	-8.458	1.00	0.00	C
ATOM	110	HB2	TYR	8	35.579	12.330	-8.445	1.00	0.00	H
ATOM	111	HB3	TYR	8	35.216	10.764	-9.306	1.00	0.00	H
ATOM	112	CG	TYR	8	37.178	11.081	-8.639	1.00	0.00	C
ATOM	113	CD1	TYR	8	37.956	12.150	-8.968	1.00	0.00	C
ATOM	114	HD1	TYR	8	37.466	13.055	-9.294	1.00	0.00	H
ATOM	115	CE1	TYR	8	39.371	12.056	-8.900	1.00	0.00	C
ATOM	116	HE1	TYR	8	40.044	12.875	-9.105	1.00	0.00	H
ATOM	117	CZ	TYR	8	39.982	10.854	-8.439	1.00	0.00	C
ATOM	118	OH	TYR	8	41.314	10.776	-8.146	1.00	0.00	O
ATOM	119	HH	TYR	8	41.577	9.869	-7.973	1.00	0.00	H
ATOM	120	CE2	TYR	8	39.184	9.684	-8.276	1.00	0.00	C
ATOM	121	HE2	TYR	8	39.645	8.749	-7.993	1.00	0.00	H
ATOM	122	CD2	TYR	8	37.782	9.779	-8.427	1.00	0.00	C
ATOM	123	HD2	TYR	8	37.178	8.933	-8.135	1.00	0.00	H
ATOM	124	C	TYR	8	35.877	10.856	-5.907	1.00	0.00	C
ATOM	125	O	TYR	8	36.550	9.962	-5.463	1.00	0.00	O
ATOM	126	N	TYR	9	35.666	12.024	-5.232	1.00	0.00	N
ATOM	127	H	TYR	9	35.110	12.713	-5.718	1.00	0.00	H
ATOM	128	CA	TYR	9	36.131	12.353	-3.931	1.00	0.00	C
ATOM	129	HA	TYR	9	37.189	12.099	-3.860	1.00	0.00	H
ATOM	130	CB	TYR	9	36.040	13.917	-3.722	1.00	0.00	C
ATOM	131	HB2	TYR	9	36.561	14.124	-2.787	1.00	0.00	H
ATOM	132	HB3	TYR	9	35.017	14.224	-3.504	1.00	0.00	H
ATOM	133	CG	TYR	9	36.600	14.732	-4.849	1.00	0.00	C
ATOM	134	CD1	TYR	9	35.851	15.601	-5.600	1.00	0.00	C
ATOM	135	HD1	TYR	9	34.815	15.679	-5.306	1.00	0.00	H
ATOM	136	CE1	TYR	9	36.387	16.349	-6.594	1.00	0.00	C
ATOM	137	HE1	TYR	9	35.787	17.087	-7.105	1.00	0.00	H
ATOM	138	CZ	TYR	9	37.729	16.183	-6.970	1.00	0.00	C
ATOM	139	OH	TYR	9	38.288	16.974	-7.922	1.00	0.00	O
ATOM	140	HH	TYR	9	39.171	16.632	-8.076	1.00	0.00	H
ATOM	141	CE2	TYR	9	38.529	15.149	-6.351	1.00	0.00	C
ATOM	142	HE2	TYR	9	39.551	14.957	-6.643	1.00	0.00	H
ATOM	143	CD2	TYR	9	37.958	14.461	-5.231	1.00	0.00	C
ATOM	144	HD2	TYR	9	38.582	13.787	-4.663	1.00	0.00	H
ATOM	145	C	TYR	9	35.379	11.601	-2.790	1.00	0.00	C
ATOM	146	O	TYR	9	34.200	11.367	-2.900	1.00	0.00	O
ATOM	147	N	ARG	10	36.169	11.159	-1.798	1.00	0.00	N
ATOM	148	H	ARG	10	37.126	11.444	-1.948	1.00	0.00	H
ATOM	149	CA	ARG	10	35.687	10.548	-0.538	1.00	0.00	C
ATOM	150	HA	ARG	10	34.711	10.959	-0.281	1.00	0.00	H
ATOM	151	CB	ARG	10	35.688	9.010	-0.619	1.00	0.00	C
ATOM	152	HB2	ARG	10	35.401	8.452	0.273	1.00	0.00	H
ATOM	153	HB3	ARG	10	36.729	8.831	-0.888	1.00	0.00	H
ATOM	154	CG	ARG	10	34.812	8.397	-1.700	1.00	0.00	C
ATOM	155	HG2	ARG	10	35.075	8.798	-2.679	1.00	0.00	H
ATOM	156	HG3	ARG	10	33.841	8.845	-1.489	1.00	0.00	H
ATOM	157	CD	ARG	10	34.789	6.869	-1.906	1.00	0.00	C
ATOM	158	HD2	ARG	10	33.983	6.377	-1.361	1.00	0.00	H
ATOM	159	HD3	ARG	10	35.785	6.538	-1.611	1.00	0.00	H

ATOM	160	NE	ARG	10	34.598	6.424	-3.265	1.00	0.00	N
ATOM	161	HE	ARG	10	35.396	5.912	-3.613	1.00	0.00	H
ATOM	162	CZ	ARG	10	33.633	6.703	-4.046	1.00	0.00	C
ATOM	163	NH1	ARG	10	32.574	7.249	-3.553	1.00	0.00	N
ATOM	164	HH11	ARG	10	32.452	7.410	-2.563	1.00	0.00	H
ATOM	165	HH12	ARG	10	31.851	7.256	-4.259	1.00	0.00	H
ATOM	166	NH2	ARG	10	33.621	6.364	-5.311	1.00	0.00	N
ATOM	167	HH21	ARG	10	34.497	6.031	-5.688	1.00	0.00	H
ATOM	168	HH22	ARG	10	33.054	7.009	-5.842	1.00	0.00	H
ATOM	169	C	ARG	10	36.602	11.151	0.602	1.00	0.00	C
ATOM	170	O	ARG	10	37.723	11.728	0.336	1.00	0.00	O
ATOM	171	N	GLU	11	36.274	10.848	1.859	1.00	0.00	N
ATOM	172	H	GLU	11	35.526	10.202	2.068	1.00	0.00	H
ATOM	173	CA	GLU	11	36.903	11.439	3.027	1.00	0.00	C
ATOM	174	HA	GLU	11	37.989	11.378	2.963	1.00	0.00	H
ATOM	175	CB	GLU	11	36.284	12.836	3.449	1.00	0.00	C
ATOM	176	HB2	GLU	11	36.536	13.614	2.728	1.00	0.00	H
ATOM	177	HB3	GLU	11	36.725	13.115	4.406	1.00	0.00	H
ATOM	178	CG	GLU	11	34.718	12.966	3.499	1.00	0.00	C
ATOM	179	HG2	GLU	11	34.276	13.873	3.909	1.00	0.00	H
ATOM	180	HG3	GLU	11	34.487	12.281	4.315	1.00	0.00	H
ATOM	181	CD	GLU	11	34.088	12.625	2.168	1.00	0.00	C
ATOM	182	OE1	GLU	11	34.321	13.387	1.200	1.00	0.00	O
ATOM	183	OE2	GLU	11	33.492	11.485	2.115	1.00	0.00	O
ATOM	184	C	GLU	11	36.584	10.424	4.180	1.00	0.00	C
ATOM	185	O	GLU	11	35.686	9.583	3.947	1.00	0.00	O
ATOM	186	N	ASN	12	37.210	10.522	5.342	1.00	0.00	N
ATOM	187	H	ASN	12	37.971	11.181	5.427	1.00	0.00	H
ATOM	188	CA	ASN	12	36.946	9.640	6.497	1.00	0.00	C
ATOM	189	HA	ASN	12	36.724	8.658	6.079	1.00	0.00	H
ATOM	190	CB	ASN	12	38.276	9.448	7.234	1.00	0.00	C
ATOM	191	HB2	ASN	12	39.127	9.305	6.569	1.00	0.00	H
ATOM	192	HB3	ASN	12	38.106	8.531	7.798	1.00	0.00	H
ATOM	193	CG	ASN	12	38.691	10.602	8.149	1.00	0.00	C
ATOM	194	OD1	ASN	12	39.494	11.516	7.816	1.00	0.00	O
ATOM	195	ND2	ASN	12	38.085	10.758	9.336	1.00	0.00	N
ATOM	196	HD21	ASN	12	38.162	11.615	9.866	1.00	0.00	H
ATOM	197	HD22	ASN	12	37.574	9.950	9.662	1.00	0.00	H
ATOM	198	C	ASN	12	35.806	9.990	7.469	1.00	0.00	C
ATOM	199	O	ASN	12	35.642	9.253	8.455	1.00	0.00	O
ATOM	200	N	MET	13	34.958	11.022	7.236	1.00	0.00	N
ATOM	201	H	MET	13	34.867	11.375	6.294	1.00	0.00	H
ATOM	202	CA	MET	13	33.894	11.413	8.166	1.00	0.00	C
ATOM	203	HA	MET	13	34.428	11.363	9.115	1.00	0.00	H
ATOM	204	CB	MET	13	33.591	12.887	8.079	1.00	0.00	C
ATOM	205	HB2	MET	13	32.910	13.013	8.920	1.00	0.00	H
ATOM	206	HB3	MET	13	33.048	12.958	7.136	1.00	0.00	H
ATOM	207	CG	MET	13	34.632	13.945	8.068	1.00	0.00	C
ATOM	208	HG2	MET	13	35.118	14.036	7.096	1.00	0.00	H
ATOM	209	HG3	MET	13	35.199	13.641	8.947	1.00	0.00	H
ATOM	210	SD	MET	13	33.889	15.473	8.261	1.00	0.00	S
ATOM	211	CE	MET	13	33.317	15.839	6.628	1.00	0.00	C
ATOM	212	HE1	MET	13	33.019	16.887	6.609	1.00	0.00	H
ATOM	213	HE2	MET	13	34.136	15.636	5.938	1.00	0.00	H
ATOM	214	HE3	MET	13	32.429	15.243	6.417	1.00	0.00	H
ATOM	215	C	MET	13	32.593	10.567	8.127	1.00	0.00	C
ATOM	216	O	MET	13	32.210	10.116	7.077	1.00	0.00	O
ATOM	217	N	TYR	14	31.945	10.361	9.247	1.00	0.00	N
ATOM	218	H	TYR	14	32.282	10.854	10.062	1.00	0.00	H
ATOM	219	CA	TYR	14	30.739	9.569	9.441	1.00	0.00	C
ATOM	220	HA	TYR	14	30.792	9.235	10.478	1.00	0.00	H
ATOM	221	CB	TYR	14	29.544	10.544	9.455	1.00	0.00	C
ATOM	222	HB2	TYR	14	28.677	10.119	9.961	1.00	0.00	H

ATOM	223	HB3	TYR	14	29.233	10.690	8.421	1.00	0.00	H
ATOM	224	CG	TYR	14	29.676	11.868	10.218	1.00	0.00	C
ATOM	225	CD1	TYR	14	30.107	13.020	9.528	1.00	0.00	C
ATOM	226	HD1	TYR	14	30.420	12.980	8.495	1.00	0.00	H
ATOM	227	CE1	TYR	14	30.084	14.332	10.200	1.00	0.00	C
ATOM	228	HE1	TYR	14	30.250	15.275	9.701	1.00	0.00	H
ATOM	229	CZ	TYR	14	29.769	14.368	11.544	1.00	0.00	C
ATOM	230	OH	TYR	14	29.674	15.585	12.182	1.00	0.00	O
ATOM	231	HH	TYR	14	29.706	16.269	11.510	1.00	0.00	H
ATOM	232	CE2	TYR	14	29.398	13.198	12.223	1.00	0.00	C
ATOM	233	HE2	TYR	14	29.225	13.361	13.277	1.00	0.00	H
ATOM	234	CD2	TYR	14	29.305	11.963	11.605	1.00	0.00	C
ATOM	235	HD2	TYR	14	28.979	11.059	12.098	1.00	0.00	H
ATOM	236	C	TYR	14	30.512	8.220	8.606	1.00	0.00	C
ATOM	237	O	TYR	14	29.378	7.847	8.115	1.00	0.00	O
ATOM	238	N	ARG	15	31.663	7.510	8.339	1.00	0.00	N
ATOM	239	H	ARG	15	32.460	7.603	8.952	1.00	0.00	H
ATOM	240	CA	ARG	15	31.832	6.358	7.475	1.00	0.00	C
ATOM	241	HA	ARG	15	30.878	6.173	6.983	1.00	0.00	H
ATOM	242	CB	ARG	15	32.757	6.706	6.273	1.00	0.00	C
ATOM	243	HB2	ARG	15	33.809	6.727	6.559	1.00	0.00	H
ATOM	244	HB3	ARG	15	32.396	7.691	5.977	1.00	0.00	H
ATOM	245	CG	ARG	15	32.989	5.891	4.991	1.00	0.00	C
ATOM	246	HG2	ARG	15	32.048	5.561	4.550	1.00	0.00	H
ATOM	247	HG3	ARG	15	33.362	4.952	5.401	1.00	0.00	H
ATOM	248	CD	ARG	15	33.785	6.497	3.834	1.00	0.00	C
ATOM	249	HD2	ARG	15	33.891	5.669	3.133	1.00	0.00	H
ATOM	250	HD3	ARG	15	34.796	6.525	4.242	1.00	0.00	H
ATOM	251	NE	ARG	15	33.342	7.757	3.223	1.00	0.00	N
ATOM	252	HE	ARG	15	33.686	8.637	3.581	1.00	0.00	H
ATOM	253	CZ	ARG	15	32.538	7.866	2.151	1.00	0.00	C
ATOM	254	NH1	ARG	15	32.133	6.783	1.522	1.00	0.00	N
ATOM	255	HH11	ARG	15	32.184	5.890	1.991	1.00	0.00	H
ATOM	256	HH12	ARG	15	31.715	6.873	0.607	1.00	0.00	H
ATOM	257	NH2	ARG	15	32.139	8.961	1.712	1.00	0.00	N
ATOM	258	HH21	ARG	15	32.515	9.853	2.000	1.00	0.00	H
ATOM	259	HH22	ARG	15	31.522	8.926	0.913	1.00	0.00	H
ATOM	260	C	ARG	15	32.196	5.082	8.134	1.00	0.00	C
ATOM	261	O	ARG	15	33.021	5.097	9.043	1.00	0.00	O
ATOM	262	N	TYR	16	31.648	3.931	7.684	1.00	0.00	N
ATOM	263	H	TYR	16	31.028	4.031	6.894	1.00	0.00	H
ATOM	264	CA	TYR	16	31.856	2.616	8.378	1.00	0.00	C
ATOM	265	HA	TYR	16	31.854	2.609	9.468	1.00	0.00	H
ATOM	266	CB	TYR	16	30.857	1.609	7.791	1.00	0.00	C
ATOM	267	HB2	TYR	16	30.904	1.498	6.708	1.00	0.00	H
ATOM	268	HB3	TYR	16	29.895	2.115	7.861	1.00	0.00	H
ATOM	269	CG	TYR	16	30.764	0.195	8.387	1.00	0.00	C
ATOM	270	CD1	TYR	16	29.633	-0.199	9.122	1.00	0.00	C
ATOM	271	HD1	TYR	16	28.766	0.433	9.249	1.00	0.00	H
ATOM	272	CE1	TYR	16	29.585	-1.513	9.652	1.00	0.00	C
ATOM	273	HE1	TYR	16	28.798	-1.707	10.366	1.00	0.00	H
ATOM	274	CZ	TYR	16	30.600	-2.404	9.423	1.00	0.00	C
ATOM	275	OH	TYR	16	30.547	-3.639	9.952	1.00	0.00	O
ATOM	276	HH	TYR	16	31.364	-4.102	9.752	1.00	0.00	H
ATOM	277	CE2	TYR	16	31.741	-2.029	8.727	1.00	0.00	C
ATOM	278	HE2	TYR	16	32.601	-2.679	8.662	1.00	0.00	H
ATOM	279	CD2	TYR	16	31.842	-0.716	8.173	1.00	0.00	C
ATOM	280	HD2	TYR	16	32.744	-0.413	7.663	1.00	0.00	H
ATOM	281	C	TYR	16	33.305	1.999	8.103	1.00	0.00	C
ATOM	282	O	TYR	16	33.873	2.014	6.937	1.00	0.00	O
ATOM	283	N	NME	17	33.920	1.542	9.132	1.00	0.00	N
ATOM	284	H	NME	17	33.350	1.618	9.963	1.00	0.00	H
ATOM	285	CH3	NME	17	35.218	0.919	9.205	1.00	0.00	C

```
ATOM    286 HH31 NME    17      35.634   1.109  10.194  1.00  0.00           H
ATOM    287 HH32 NME    17      35.127  -0.164   9.111  1.00  0.00           H
ATOM    288 HH33 NME    17      35.938   1.328   8.496  1.00  0.00           H
TER     289      NME    17
ATOM    289 HH31 ACE    18      27.798   3.315  -6.312  1.00  0.00           H
ATOM    290  CH3 ACE    18      28.376   4.073  -5.783  1.00  0.00           C
ATOM    291 HH32 ACE    18      27.928   4.691  -5.005  1.00  0.00           H
ATOM    292 HH33 ACE    18      28.726   4.728  -6.581  1.00  0.00           H
ATOM    293  C   ACE    18      29.627   3.444  -5.165  1.00  0.00           C
ATOM    294  O   ACE    18      30.732   3.475  -5.757  1.00  0.00           O
ATOM    295  N   HIE    19      29.625   3.143  -3.839  1.00  0.00           N
ATOM    296  H   HIE    19      28.746   3.137  -3.342  1.00  0.00           H
ATOM    297  CA  HIE    19      30.865   2.614  -3.119  1.00  0.00           C
ATOM    298  HA  HIE    19      31.606   2.328  -3.866  1.00  0.00           H
ATOM    299  CB  HIE    19      31.481   3.669  -2.218  1.00  0.00           C
ATOM    300  HB2 HIE    19      30.888   3.821  -1.316  1.00  0.00           H
ATOM    301  HB3 HIE    19      31.546   4.639  -2.711  1.00  0.00           H
ATOM    302  CG  HIE    19      33.005   3.458  -1.910  1.00  0.00           C
ATOM    303  ND1 HIE    19      33.554   3.279  -0.631  1.00  0.00           N
ATOM    304  CE1 HIE    19      34.861   3.092  -0.830  1.00  0.00           C
ATOM    305  HE1 HIE    19      35.633   3.034  -0.076  1.00  0.00           H
ATOM    306  NE2 HIE    19      35.122   3.134  -2.088  1.00  0.00           N
ATOM    307  HE2 HIE    19      36.051   2.874  -2.387  1.00  0.00           H
ATOM    308  CD2 HIE    19      33.983   3.307  -2.856  1.00  0.00           C
ATOM    309  HD2 HIE    19      33.888   3.252  -3.930  1.00  0.00           H
ATOM    310  C   HIE    19      30.634   1.344  -2.206  1.00  0.00           C
ATOM    311  O   HIE    19      29.515   0.935  -1.940  1.00  0.00           O
ATOM    312  N   SER    20      31.690   0.725  -1.805  1.00  0.00           N
ATOM    313  H   SER    20      32.570   1.075  -2.159  1.00  0.00           H
ATOM    314  CA  SER    20      31.785  -0.393  -0.814  1.00  0.00           C
ATOM    315  HA  SER    20      30.900  -0.991  -1.030  1.00  0.00           H
ATOM    316  CB  SER    20      32.992  -1.227  -1.248  1.00  0.00           C
ATOM    317  HB2 SER    20      32.922  -1.544  -2.289  1.00  0.00           H
ATOM    318  HB3 SER    20      33.155  -2.099  -0.615  1.00  0.00           H
ATOM    319  OG  SER    20      34.239  -0.496  -1.118  1.00  0.00           O
ATOM    320  HG  SER    20      34.612  -0.367  -1.993  1.00  0.00           H
ATOM    321  C   SER    20      31.837  -0.191   0.698  1.00  0.00           C
ATOM    322  O   SER    20      32.054  -1.132   1.488  1.00  0.00           O
ATOM    323  N   GLN    21      31.761   1.089   1.056  1.00  0.00           N
ATOM    324  H   GLN    21      31.813   1.859   0.404  1.00  0.00           H
ATOM    325  CA  GLN    21      31.804   1.519   2.440  1.00  0.00           C
ATOM    326  HA  GLN    21      31.627   0.661   3.088  1.00  0.00           H
ATOM    327  CB  GLN    21      33.282   1.989   2.776  1.00  0.00           C
ATOM    328  HB2 GLN    21      33.294   2.637   3.652  1.00  0.00           H
ATOM    329  HB3 GLN    21      33.613   2.618   1.949  1.00  0.00           H
ATOM    330  CG  GLN    21      34.345   0.830   2.829  1.00  0.00           C
ATOM    331  HG2 GLN    21      34.238   0.305   1.880  1.00  0.00           H
ATOM    332  HG3 GLN    21      34.049   0.157   3.634  1.00  0.00           H
ATOM    333  CD  GLN    21      35.811   1.239   2.815  1.00  0.00           C
ATOM    334  OE1 GLN    21      36.266   2.310   3.227  1.00  0.00           O
ATOM    335  NE2 GLN    21      36.625   0.211   2.499  1.00  0.00           N
ATOM    336 HE21 GLN    21      37.593   0.456   2.348  1.00  0.00           H
ATOM    337 HE22 GLN    21      36.235  -0.679   2.225  1.00  0.00           H
ATOM    338  C   GLN    21      30.816   2.654   2.705  1.00  0.00           C
ATOM    339  O   GLN    21      30.830   3.682   2.071  1.00  0.00           O
ATOM    340  N   TRP    22      29.956   2.382   3.723  1.00  0.00           N
ATOM    341  H   TRP    22      30.029   1.513   4.233  1.00  0.00           H
ATOM    342  CA  TRP    22      28.710   3.182   3.917  1.00  0.00           C
ATOM    343  HA  TRP    22      28.326   3.518   2.954  1.00  0.00           H
ATOM    344  CB  TRP    22      27.676   2.238   4.525  1.00  0.00           C
ATOM    345  HB2 TRP    22      28.053   1.685   5.385  1.00  0.00           H
ATOM    346  HB3 TRP    22      27.603   1.506   3.721  1.00  0.00           H
ATOM    347  CG  TRP    22      26.404   2.721   4.923  1.00  0.00           C
```

ATOM	348	CD1	TRP	22	25.398	3.126	4.112	1.00	0.00	C
ATOM	349	HD1	TRP	22	25.554	3.267	3.053	1.00	0.00	H
ATOM	350	NE1	TRP	22	24.287	3.443	4.903	1.00	0.00	N
ATOM	351	HE1	TRP	22	23.488	3.956	4.559	1.00	0.00	H
ATOM	352	CE2	TRP	22	24.513	3.324	6.216	1.00	0.00	C
ATOM	353	CZ2	TRP	22	23.788	3.638	7.361	1.00	0.00	C
ATOM	354	HZ2	TRP	22	22.784	4.034	7.403	1.00	0.00	H
ATOM	355	CH2	TRP	22	24.371	3.380	8.609	1.00	0.00	C
ATOM	356	HH2	TRP	22	23.866	3.568	9.545	1.00	0.00	H
ATOM	357	CZ3	TRP	22	25.629	2.874	8.753	1.00	0.00	C
ATOM	358	HZ3	TRP	22	25.986	2.778	9.768	1.00	0.00	H
ATOM	359	CE3	TRP	22	26.371	2.572	7.588	1.00	0.00	C
ATOM	360	HE3	TRP	22	27.329	2.130	7.820	1.00	0.00	H
ATOM	361	CD2	TRP	22	25.826	2.856	6.268	1.00	0.00	C
ATOM	362	C	TRP	22	28.943	4.486	4.718	1.00	0.00	C
ATOM	363	O	TRP	22	29.684	4.480	5.718	1.00	0.00	O
ATOM	364	N	ASN	23	28.253	5.591	4.404	1.00	0.00	N
ATOM	365	H	ASN	23	27.725	5.608	3.543	1.00	0.00	H
ATOM	366	CA	ASN	23	28.241	6.890	5.184	1.00	0.00	C
ATOM	367	HA	ASN	23	28.743	6.673	6.127	1.00	0.00	H
ATOM	368	CB	ASN	23	29.115	7.924	4.479	1.00	0.00	C
ATOM	369	HB2	ASN	23	30.120	7.512	4.388	1.00	0.00	H
ATOM	370	HB3	ASN	23	29.189	8.716	5.224	1.00	0.00	H
ATOM	371	CG	ASN	23	28.585	8.352	3.150	1.00	0.00	C
ATOM	372	OD1	ASN	23	28.675	7.671	2.113	1.00	0.00	O
ATOM	373	ND2	ASN	23	27.870	9.488	3.246	1.00	0.00	N
ATOM	374	HD21	ASN	23	27.672	9.971	2.382	1.00	0.00	H
ATOM	375	HD22	ASN	23	27.926	10.226	3.933	1.00	0.00	H
ATOM	376	C	ASN	23	26.857	7.388	5.523	1.00	0.00	C
ATOM	377	O	ASN	23	25.873	7.226	4.818	1.00	0.00	O
ATOM	378	N	LYS	24	26.761	8.021	6.719	1.00	0.00	N
ATOM	379	H	LYS	24	27.574	8.053	7.317	1.00	0.00	H
ATOM	380	CA	LYS	24	25.463	8.792	7.159	1.00	0.00	C
ATOM	381	HA	LYS	24	25.058	9.295	6.281	1.00	0.00	H
ATOM	382	CB	LYS	24	24.501	7.741	7.784	1.00	0.00	C
ATOM	383	HB2	LYS	24	24.836	7.527	8.799	1.00	0.00	H
ATOM	384	HB3	LYS	24	24.638	6.904	7.100	1.00	0.00	H
ATOM	385	CG	LYS	24	22.999	8.158	7.784	1.00	0.00	C
ATOM	386	HG2	LYS	24	22.854	9.053	8.388	1.00	0.00	H
ATOM	387	HG3	LYS	24	22.470	7.325	8.247	1.00	0.00	H
ATOM	388	CD	LYS	24	22.556	8.341	6.337	1.00	0.00	C
ATOM	389	HD2	LYS	24	22.770	7.515	5.658	1.00	0.00	H
ATOM	390	HD3	LYS	24	22.935	9.313	6.022	1.00	0.00	H
ATOM	391	CE	LYS	24	21.021	8.546	6.202	1.00	0.00	C
ATOM	392	HE2	LYS	24	20.853	9.573	6.527	1.00	0.00	H
ATOM	393	HE3	LYS	24	20.523	7.912	6.935	1.00	0.00	H
ATOM	394	NZ	LYS	24	20.458	8.285	4.859	1.00	0.00	N
ATOM	395	HZ1	LYS	24	19.471	8.499	4.895	1.00	0.00	H
ATOM	396	HZ2	LYS	24	20.836	8.919	4.170	1.00	0.00	H
ATOM	397	HZ3	LYS	24	20.627	7.348	4.523	1.00	0.00	H
ATOM	398	C	LYS	24	25.888	9.898	8.234	1.00	0.00	C
ATOM	399	O	LYS	24	26.456	9.434	9.252	1.00	0.00	O
ATOM	400	N	PRO	25	25.784	11.240	8.026	1.00	0.00	N
ATOM	401	CD	PRO	25	25.216	11.846	6.796	1.00	0.00	C
ATOM	402	HD2	PRO	25	25.980	11.690	6.034	1.00	0.00	H
ATOM	403	HD3	PRO	25	24.221	11.493	6.524	1.00	0.00	H
ATOM	404	CG	PRO	25	25.030	13.328	7.166	1.00	0.00	C
ATOM	405	HG2	PRO	25	25.047	13.885	6.229	1.00	0.00	H
ATOM	406	HG3	PRO	25	24.136	13.558	7.745	1.00	0.00	H
ATOM	407	CB	PRO	25	26.203	13.571	8.140	1.00	0.00	C
ATOM	408	HB2	PRO	25	27.059	13.651	7.470	1.00	0.00	H
ATOM	409	HB3	PRO	25	26.152	14.403	8.842	1.00	0.00	H
ATOM	410	CA	PRO	25	26.243	12.285	8.959	1.00	0.00	C

ATOM	411	HA	PRO	25	27.246	12.000	9.276	1.00	0.00	H
ATOM	412	C	PRO	25	25.508	12.283	10.326	1.00	0.00	C
ATOM	413	O	PRO	25	24.328	11.988	10.496	1.00	0.00	O
ATOM	414	N	SER	26	26.225	12.680	11.371	1.00	0.00	N
ATOM	415	H	SER	26	27.163	12.994	11.167	1.00	0.00	H
ATOM	416	CA	SER	26	25.818	12.666	12.813	1.00	0.00	C
ATOM	417	HA	SER	26	26.795	12.768	13.286	1.00	0.00	H
ATOM	418	CB	SER	26	25.028	13.971	13.204	1.00	0.00	C
ATOM	419	HB2	SER	26	24.843	13.918	14.276	1.00	0.00	H
ATOM	420	HB3	SER	26	24.056	13.871	12.720	1.00	0.00	H
ATOM	421	OG	SER	26	25.691	15.183	12.815	1.00	0.00	O
ATOM	422	HG	SER	26	26.181	15.020	12.006	1.00	0.00	H
ATOM	423	C	SER	26	25.056	11.370	13.360	1.00	0.00	C
ATOM	424	O	SER	26	23.952	11.475	13.870	1.00	0.00	O
ATOM	425	N	LYS	27	25.711	10.191	13.167	1.00	0.00	N
ATOM	426	H	LYS	27	26.641	10.258	12.779	1.00	0.00	H
ATOM	427	CA	LYS	27	25.211	8.915	13.633	1.00	0.00	C
ATOM	428	HA	LYS	27	24.378	9.120	14.306	1.00	0.00	H
ATOM	429	CB	LYS	27	24.740	8.011	12.495	1.00	0.00	C
ATOM	430	HB2	LYS	27	24.366	7.153	13.055	1.00	0.00	H
ATOM	431	HB3	LYS	27	25.562	7.752	11.829	1.00	0.00	H
ATOM	432	CG	LYS	27	23.709	8.334	11.424	1.00	0.00	C
ATOM	433	HG2	LYS	27	23.410	7.493	10.798	1.00	0.00	H
ATOM	434	HG3	LYS	27	24.012	9.228	10.879	1.00	0.00	H
ATOM	435	CD	LYS	27	22.419	8.675	12.144	1.00	0.00	C
ATOM	436	HD2	LYS	27	22.571	9.694	12.502	1.00	0.00	H
ATOM	437	HD3	LYS	27	22.310	8.008	12.999	1.00	0.00	H
ATOM	438	CE	LYS	27	21.186	8.812	11.227	1.00	0.00	C
ATOM	439	HE2	LYS	27	21.120	8.023	10.477	1.00	0.00	H
ATOM	440	HE3	LYS	27	21.325	9.805	10.798	1.00	0.00	H
ATOM	441	NZ	LYS	27	19.940	8.796	12.075	1.00	0.00	N
ATOM	442	HZ1	LYS	27	19.986	9.454	12.840	1.00	0.00	H
ATOM	443	HZ2	LYS	27	19.104	8.887	11.516	1.00	0.00	H
ATOM	444	HZ3	LYS	27	19.898	7.874	12.484	1.00	0.00	H
ATOM	445	C	LYS	27	26.278	8.297	14.567	1.00	0.00	C
ATOM	446	O	LYS	27	27.478	8.261	14.222	1.00	0.00	O
ATOM	447	N	PRO	28	25.928	7.888	15.819	1.00	0.00	N
ATOM	448	CD	PRO	28	24.590	7.827	16.423	1.00	0.00	C
ATOM	449	HD2	PRO	28	24.295	8.872	16.516	1.00	0.00	H
ATOM	450	HD3	PRO	28	23.877	7.254	15.830	1.00	0.00	H
ATOM	451	CG	PRO	28	24.703	7.153	17.739	1.00	0.00	C
ATOM	452	HG2	PRO	28	23.967	7.527	18.451	1.00	0.00	H
ATOM	453	HG3	PRO	28	24.754	6.065	17.734	1.00	0.00	H
ATOM	454	CB	PRO	28	26.080	7.575	18.186	1.00	0.00	C
ATOM	455	HB2	PRO	28	26.045	8.565	18.641	1.00	0.00	H
ATOM	456	HB3	PRO	28	26.653	6.987	18.903	1.00	0.00	H
ATOM	457	CA	PRO	28	26.966	7.590	16.901	1.00	0.00	C
ATOM	458	HA	PRO	28	27.733	8.363	16.857	1.00	0.00	H
ATOM	459	C	PRO	28	27.552	6.203	16.743	1.00	0.00	C
ATOM	460	O	PRO	28	27.117	5.442	15.934	1.00	0.00	O
ATOM	461	N	LYS	29	28.566	5.816	17.489	1.00	0.00	N
ATOM	462	H	LYS	29	28.824	6.427	18.251	1.00	0.00	H
ATOM	463	CA	LYS	29	29.310	4.590	17.295	1.00	0.00	C
ATOM	464	HA	LYS	29	29.652	4.656	16.262	1.00	0.00	H
ATOM	465	CB	LYS	29	30.607	4.407	18.108	1.00	0.00	C
ATOM	466	HB2	LYS	29	31.024	3.446	17.808	1.00	0.00	H
ATOM	467	HB3	LYS	29	30.222	4.448	19.127	1.00	0.00	H
ATOM	468	CG	LYS	29	31.588	5.596	17.831	1.00	0.00	C
ATOM	469	HG2	LYS	29	31.051	6.460	18.225	1.00	0.00	H
ATOM	470	HG3	LYS	29	31.897	5.610	16.786	1.00	0.00	H
ATOM	471	CD	LYS	29	32.907	5.641	18.635	1.00	0.00	C
ATOM	472	HD2	LYS	29	33.412	4.690	18.466	1.00	0.00	H
ATOM	473	HD3	LYS	29	32.619	5.670	19.686	1.00	0.00	H

ATOM	474	CE	LYS	29	33.786	6.812	18.254	1.00	0.00	C
ATOM	475	HE2	LYS	29	33.267	7.764	18.360	1.00	0.00	H
ATOM	476	HE3	LYS	29	33.968	6.533	17.216	1.00	0.00	H
ATOM	477	NZ	LYS	29	34.996	6.707	19.132	1.00	0.00	N
ATOM	478	HZ1	LYS	29	34.823	6.858	20.116	1.00	0.00	H
ATOM	479	HZ2	LYS	29	35.379	5.781	19.000	1.00	0.00	H
ATOM	480	HZ3	LYS	29	35.655	7.435	18.896	1.00	0.00	H
ATOM	481	C	LYS	29	28.466	3.310	17.480	1.00	0.00	C
ATOM	482	O	LYS	29	28.734	2.372	16.755	1.00	0.00	O
ATOM	483	N	THR	30	27.439	3.306	18.369	1.00	0.00	N
ATOM	484	H	THR	30	27.260	4.189	18.826	1.00	0.00	H
ATOM	485	CA	THR	30	26.518	2.191	18.586	1.00	0.00	C
ATOM	486	HA	THR	30	27.041	1.247	18.743	1.00	0.00	H
ATOM	487	CB	THR	30	25.520	2.560	19.783	1.00	0.00	C
ATOM	488	HB	THR	30	24.785	1.779	19.978	1.00	0.00	H
ATOM	489	CG2	THR	30	26.429	2.718	21.022	1.00	0.00	C
ATOM	490	HG21	THR	30	26.871	1.766	21.313	1.00	0.00	H
ATOM	491	HG22	THR	30	27.167	3.512	20.912	1.00	0.00	H
ATOM	492	HG23	THR	30	25.808	3.081	21.841	1.00	0.00	H
ATOM	493	OG1	THR	30	24.914	3.795	19.439	1.00	0.00	O
ATOM	494	HG1	THR	30	24.269	3.887	20.144	1.00	0.00	H
ATOM	495	C	THR	30	25.627	1.980	17.338	1.00	0.00	C
ATOM	496	O	THR	30	25.037	0.899	17.106	1.00	0.00	O
ATOM	497	N	ASN	31	25.563	3.029	16.448	1.00	0.00	N
ATOM	498	H	ASN	31	26.011	3.930	16.540	1.00	0.00	H
ATOM	499	CA	ASN	31	24.772	2.868	15.239	1.00	0.00	C
ATOM	500	HA	ASN	31	23.957	2.152	15.345	1.00	0.00	H
ATOM	501	CB	ASN	31	24.184	4.162	14.802	1.00	0.00	C
ATOM	502	HB2	ASN	31	24.900	4.981	14.726	1.00	0.00	H
ATOM	503	HB3	ASN	31	23.539	4.440	15.636	1.00	0.00	H
ATOM	504	CG	ASN	31	23.385	4.155	13.512	1.00	0.00	C
ATOM	505	OD1	ASN	31	23.888	4.190	12.424	1.00	0.00	O
ATOM	506	ND2	ASN	31	22.084	4.045	13.585	1.00	0.00	N
ATOM	507	HD21	ASN	31	21.586	4.302	12.745	1.00	0.00	H
ATOM	508	HD22	ASN	31	21.676	3.911	14.500	1.00	0.00	H
ATOM	509	C	ASN	31	25.680	2.272	14.166	1.00	0.00	C
ATOM	510	O	ASN	31	25.205	1.303	13.524	1.00	0.00	O
ATOM	511	N	MET	32	26.963	2.730	14.049	1.00	0.00	N
ATOM	512	H	MET	32	27.170	3.657	14.392	1.00	0.00	H
ATOM	513	CA	MET	32	27.949	2.295	13.037	1.00	0.00	C
ATOM	514	HA	MET	32	27.481	2.255	12.054	1.00	0.00	H
ATOM	515	CB	MET	32	29.196	3.197	13.190	1.00	0.00	C
ATOM	516	HB2	MET	32	30.070	2.784	12.687	1.00	0.00	H
ATOM	517	HB3	MET	32	29.436	3.220	14.253	1.00	0.00	H
ATOM	518	CG	MET	32	29.023	4.667	12.763	1.00	0.00	C
ATOM	519	HG2	MET	32	29.865	5.310	13.019	1.00	0.00	H
ATOM	520	HG3	MET	32	28.239	4.884	13.489	1.00	0.00	H
ATOM	521	SD	MET	32	28.298	4.955	11.098	1.00	0.00	S
ATOM	522	CE	MET	32	29.400	3.925	10.082	1.00	0.00	C
ATOM	523	HE1	MET	32	29.309	2.884	10.391	1.00	0.00	H
ATOM	524	HE2	MET	32	29.108	4.076	9.043	1.00	0.00	H
ATOM	525	HE3	MET	32	30.441	4.181	10.278	1.00	0.00	H
ATOM	526	C	MET	32	28.357	0.825	13.179	1.00	0.00	C
ATOM	527	O	MET	32	28.524	0.099	12.153	1.00	0.00	O
ATOM	528	N	LYS	33	28.516	0.392	14.472	1.00	0.00	N
ATOM	529	H	LYS	33	28.270	1.040	15.207	1.00	0.00	H
ATOM	530	CA	LYS	33	29.036	-0.927	14.957	1.00	0.00	C
ATOM	531	HA	LYS	33	29.483	-1.327	14.047	1.00	0.00	H
ATOM	532	CB	LYS	33	30.210	-0.826	16.045	1.00	0.00	C
ATOM	533	HB2	LYS	33	30.653	-1.790	16.298	1.00	0.00	H
ATOM	534	HB3	LYS	33	29.763	-0.363	16.924	1.00	0.00	H
ATOM	535	CG	LYS	33	31.407	0.060	15.535	1.00	0.00	C
ATOM	536	HG2	LYS	33	30.991	0.993	15.156	1.00	0.00	H

```
ATOM    537  HG3 LYS    33      31.760  -0.510  14.675  1.00  0.00           H
ATOM    538  CD  LYS    33      32.687   0.149  16.505  1.00  0.00           C
ATOM    539  HD2 LYS    33      33.017  -0.885  16.610  1.00  0.00           H
ATOM    540  HD3 LYS    33      32.396   0.612  17.448  1.00  0.00           H
ATOM    541  CE  LYS    33      33.897   0.795  15.881  1.00  0.00           C
ATOM    542  HE2 LYS    33      33.800   1.863  15.689  1.00  0.00           H
ATOM    543  HE3 LYS    33      34.050   0.251  14.949  1.00  0.00           H
ATOM    544  NZ  LYS    33      35.161   0.591  16.610  1.00  0.00           N
ATOM    545  HZ1 LYS    33      36.011   0.925  16.178  1.00  0.00           H
ATOM    546  HZ2 LYS    33      34.989   1.034  17.502  1.00  0.00           H
ATOM    547  HZ3 LYS    33      35.244  -0.396  16.809  1.00  0.00           H
ATOM    548  C   LYS    33      27.970  -1.977  15.299  1.00  0.00           C
ATOM    549  O   LYS    33      28.278  -3.052  15.908  1.00  0.00           O
ATOM    550  N   NME    34      26.678  -1.792  14.963  1.00  0.00           N
ATOM    551  H   NME    34      26.466  -0.976  14.407  1.00  0.00           H
ATOM    552  CH3 NME    34      25.660  -2.673  15.346  1.00  0.00           C
ATOM    553 HH31 NME    34      25.551  -2.816  16.421  1.00  0.00           H
ATOM    554 HH32 NME    34      24.691  -2.303  15.009  1.00  0.00           H
ATOM    555 HH33 NME    34      25.953  -3.659  14.983  1.00  0.00           H
TER     556      NME    34
END
```

Cluster 2:

```
ATOM      1 HH31 ACE     1      30.678  11.853  -7.137  1.00  0.00           H
ATOM      2  CH3 ACE     1      30.221  12.254  -6.233  1.00  0.00           C
ATOM      3 HH32 ACE     1      30.914  12.915  -5.711  1.00  0.00           H
ATOM      4 HH33 ACE     1      29.964  11.441  -5.554  1.00  0.00           H
ATOM      5  C   ACE     1      29.027  12.955  -6.671  1.00  0.00           C
ATOM      6  O   ACE     1      28.696  14.025  -6.136  1.00  0.00           O
ATOM      7  N   ASN     2      28.346  12.443  -7.632  1.00  0.00           N
ATOM      8  H   ASN     2      28.754  11.629  -8.070  1.00  0.00           H
ATOM      9  CA  ASN     2      27.171  13.129  -8.115  1.00  0.00           C
ATOM     10  HA  ASN     2      27.397  14.185  -8.266  1.00  0.00           H
ATOM     11  CB  ASN     2      26.738  12.498  -9.447  1.00  0.00           C
ATOM     12  HB2 ASN     2      26.475  11.446  -9.338  1.00  0.00           H
ATOM     13  HB3 ASN     2      27.626  12.649 -10.061  1.00  0.00           H
ATOM     14  CG  ASN     2      25.603  13.200 -10.056  1.00  0.00           C
ATOM     15  OD1 ASN     2      25.655  14.421 -10.191  1.00  0.00           O
ATOM     16  ND2 ASN     2      24.608  12.512 -10.494  1.00  0.00           N
ATOM     17 HD21 ASN     2      23.795  12.958 -10.895  1.00  0.00           H
ATOM     18 HD22 ASN     2      24.733  11.514 -10.577  1.00  0.00           H
ATOM     19  C   ASN     2      25.999  13.072  -7.104  1.00  0.00           C
ATOM     20  O   ASN     2      25.506  11.963  -6.842  1.00  0.00           O
ATOM     21  N   ASP     3      25.574  14.285  -6.629  1.00  0.00           N
ATOM     22  H   ASP     3      25.790  15.134  -7.130  1.00  0.00           H
ATOM     23  CA  ASP     3      24.643  14.355  -5.459  1.00  0.00           C
ATOM     24  HA  ASP     3      25.019  13.730  -4.648  1.00  0.00           H
ATOM     25  CB  ASP     3      24.454  15.791  -4.973  1.00  0.00           C
ATOM     26  HB2 ASP     3      24.122  16.450  -5.776  1.00  0.00           H
ATOM     27  HB3 ASP     3      25.445  16.129  -4.667  1.00  0.00           H
ATOM     28  CG  ASP     3      23.366  15.881  -3.883  1.00  0.00           C
ATOM     29  OD1 ASP     3      23.712  15.760  -2.685  1.00  0.00           O
ATOM     30  OD2 ASP     3      22.186  16.061  -4.193  1.00  0.00           O
ATOM     31  C   ASP     3      23.333  13.693  -5.886  1.00  0.00           C
ATOM     32  O   ASP     3      22.536  13.151  -5.063  1.00  0.00           O
ATOM     33  N   TYR     4      23.114  13.730  -7.216  1.00  0.00           N
ATOM     34  H   TYR     4      23.715  14.276  -7.816  1.00  0.00           H
ATOM     35  CA  TYR     4      21.794  13.218  -7.791  1.00  0.00           C
ATOM     36  HA  TYR     4      21.036  13.653  -7.140  1.00  0.00           H
ATOM     37  CB  TYR     4      21.662  13.897  -9.174  1.00  0.00           C
```

ATOM	38	HB2	TYR	4	20.982	13.311	-9.793	1.00	0.00	H
ATOM	39	HB3	TYR	4	22.651	13.803	-9.621	1.00	0.00	H
ATOM	40	CG	TYR	4	21.154	15.333	-9.190	1.00	0.00	C
ATOM	41	CD1	TYR	4	19.744	15.669	-9.308	1.00	0.00	C
ATOM	42	HD1	TYR	4	19.038	14.860	-9.419	1.00	0.00	H
ATOM	43	CE1	TYR	4	19.346	16.984	-9.252	1.00	0.00	C
ATOM	44	HE1	TYR	4	18.305	17.256	-9.352	1.00	0.00	H
ATOM	45	CZ	TYR	4	20.296	18.050	-8.826	1.00	0.00	C
ATOM	46	OH	TYR	4	19.893	19.316	-8.804	1.00	0.00	O
ATOM	47	HH	TYR	4	18.935	19.364	-8.843	1.00	0.00	H
ATOM	48	CE2	TYR	4	21.656	17.651	-8.650	1.00	0.00	C
ATOM	49	HE2	TYR	4	22.333	18.439	-8.355	1.00	0.00	H
ATOM	50	CD2	TYR	4	22.073	16.352	-8.873	1.00	0.00	C
ATOM	51	HD2	TYR	4	23.090	16.014	-8.746	1.00	0.00	H
ATOM	52	C	TYR	4	21.727	11.705	-7.855	1.00	0.00	C
ATOM	53	O	TYR	4	20.589	11.262	-7.936	1.00	0.00	O
ATOM	54	N	GLU	5	22.808	10.943	-7.561	1.00	0.00	N
ATOM	55	H	GLU	5	23.704	11.408	-7.588	1.00	0.00	H
ATOM	56	CA	GLU	5	22.841	9.428	-7.398	1.00	0.00	C
ATOM	57	HA	GLU	5	21.856	8.974	-7.507	1.00	0.00	H
ATOM	58	CB	GLU	5	23.727	8.828	-8.554	1.00	0.00	C
ATOM	59	HB2	GLU	5	24.151	7.855	-8.307	1.00	0.00	H
ATOM	60	HB3	GLU	5	24.576	9.508	-8.622	1.00	0.00	H
ATOM	61	CG	GLU	5	22.904	8.812	-9.875	1.00	0.00	C
ATOM	62	HG2	GLU	5	22.253	9.670	-10.042	1.00	0.00	H
ATOM	63	HG3	GLU	5	22.122	8.055	-9.804	1.00	0.00	H
ATOM	64	CD	GLU	5	23.868	8.713	-11.102	1.00	0.00	C
ATOM	65	OE1	GLU	5	24.717	9.620	-11.399	1.00	0.00	O
ATOM	66	OE2	GLU	5	23.750	7.721	-11.889	1.00	0.00	O
ATOM	67	C	GLU	5	23.329	8.891	-6.053	1.00	0.00	C
ATOM	68	O	GLU	5	22.887	7.879	-5.538	1.00	0.00	O
ATOM	69	N	ASP	6	24.009	9.784	-5.315	1.00	0.00	N
ATOM	70	H	ASP	6	24.168	10.626	-5.848	1.00	0.00	H
ATOM	71	CA	ASP	6	24.406	9.723	-3.878	1.00	0.00	C
ATOM	72	HA	ASP	6	24.930	8.779	-3.725	1.00	0.00	H
ATOM	73	CB	ASP	6	25.407	10.888	-3.644	1.00	0.00	C
ATOM	74	HB2	ASP	6	24.966	11.885	-3.689	1.00	0.00	H
ATOM	75	HB3	ASP	6	26.256	10.767	-4.316	1.00	0.00	H
ATOM	76	CG	ASP	6	25.969	10.792	-2.194	1.00	0.00	C
ATOM	77	OD1	ASP	6	26.787	9.832	-2.006	1.00	0.00	O
ATOM	78	OD2	ASP	6	25.635	11.570	-1.239	1.00	0.00	O
ATOM	79	C	ASP	6	23.214	9.741	-2.899	1.00	0.00	C
ATOM	80	O	ASP	6	23.270	8.944	-1.951	1.00	0.00	O
ATOM	81	N	ARG	7	22.114	10.459	-3.156	1.00	0.00	N
ATOM	82	H	ARG	7	22.132	10.897	-4.066	1.00	0.00	H
ATOM	83	CA	ARG	7	20.957	10.604	-2.180	1.00	0.00	C
ATOM	84	HA	ARG	7	21.330	11.052	-1.259	1.00	0.00	H
ATOM	85	CB	ARG	7	19.962	11.586	-2.804	1.00	0.00	C
ATOM	86	HB2	ARG	7	20.527	12.517	-2.821	1.00	0.00	H
ATOM	87	HB3	ARG	7	19.173	11.713	-2.063	1.00	0.00	H
ATOM	88	CG	ARG	7	19.362	11.411	-4.277	1.00	0.00	C
ATOM	89	HG2	ARG	7	18.917	10.426	-4.412	1.00	0.00	H
ATOM	90	HG3	ARG	7	20.225	11.504	-4.938	1.00	0.00	H
ATOM	91	CD	ARG	7	18.318	12.467	-4.628	1.00	0.00	C
ATOM	92	HD2	ARG	7	17.692	12.434	-3.736	1.00	0.00	H
ATOM	93	HD3	ARG	7	17.795	12.091	-5.507	1.00	0.00	H
ATOM	94	NE	ARG	7	19.019	13.802	-4.833	1.00	0.00	N
ATOM	95	HE	ARG	7	19.959	13.829	-4.464	1.00	0.00	H
ATOM	96	CZ	ARG	7	18.501	14.719	-5.664	1.00	0.00	C
ATOM	97	NH1	ARG	7	17.309	14.651	-6.170	1.00	0.00	N
ATOM	98	HH11	ARG	7	17.093	15.230	-6.968	1.00	0.00	H
ATOM	99	HH12	ARG	7	16.616	13.954	-5.936	1.00	0.00	H
ATOM	100	NH2	ARG	7	19.148	15.828	-5.796	1.00	0.00	N

ATOM	101	HH21	ARG	7	18.564	16.572	-6.152	1.00	0.00	H
ATOM	102	HH22	ARG	7	19.941	16.106	-5.235	1.00	0.00	H
ATOM	103	C	ARG	7	20.236	9.251	-1.858	1.00	0.00	C
ATOM	104	O	ARG	7	19.563	9.229	-0.809	1.00	0.00	O
ATOM	105	N	TYR	8	20.458	8.169	-2.626	1.00	0.00	N
ATOM	106	H	TYR	8	20.921	8.425	-3.487	1.00	0.00	H
ATOM	107	CA	TYR	8	19.811	6.807	-2.567	1.00	0.00	C
ATOM	108	HA	TYR	8	18.813	6.864	-2.133	1.00	0.00	H
ATOM	109	CB	TYR	8	19.485	6.375	-4.017	1.00	0.00	C
ATOM	110	HB2	TYR	8	19.071	5.368	-3.964	1.00	0.00	H
ATOM	111	HB3	TYR	8	20.484	6.402	-4.451	1.00	0.00	H
ATOM	112	CG	TYR	8	18.657	7.218	-4.903	1.00	0.00	C
ATOM	113	CD1	TYR	8	17.330	7.561	-4.632	1.00	0.00	C
ATOM	114	HD1	TYR	8	16.854	7.191	-3.736	1.00	0.00	H
ATOM	115	CE1	TYR	8	16.620	8.556	-5.394	1.00	0.00	C
ATOM	116	HE1	TYR	8	15.582	8.785	-5.204	1.00	0.00	H
ATOM	117	CZ	TYR	8	17.143	8.895	-6.646	1.00	0.00	C
ATOM	118	OH	TYR	8	16.407	9.816	-7.427	1.00	0.00	O
ATOM	119	HH	TYR	8	17.005	10.114	-8.116	1.00	0.00	H
ATOM	120	CE2	TYR	8	18.424	8.387	-7.068	1.00	0.00	C
ATOM	121	HE2	TYR	8	18.802	8.641	-8.048	1.00	0.00	H
ATOM	122	CD2	TYR	8	19.200	7.642	-6.144	1.00	0.00	C
ATOM	123	HD2	TYR	8	20.176	7.309	-6.466	1.00	0.00	H
ATOM	124	C	TYR	8	20.737	5.856	-1.797	1.00	0.00	C
ATOM	125	O	TYR	8	20.283	4.898	-1.280	1.00	0.00	O
ATOM	126	N	TYR	9	22.101	6.109	-1.686	1.00	0.00	N
ATOM	127	H	TYR	9	22.346	7.025	-2.033	1.00	0.00	H
ATOM	128	CA	TYR	9	23.095	5.469	-0.811	1.00	0.00	C
ATOM	129	HA	TYR	9	23.021	4.383	-0.761	1.00	0.00	H
ATOM	130	CB	TYR	9	24.423	5.840	-1.461	1.00	0.00	C
ATOM	131	HB2	TYR	9	24.666	6.894	-1.326	1.00	0.00	H
ATOM	132	HB3	TYR	9	24.198	5.671	-2.514	1.00	0.00	H
ATOM	133	CG	TYR	9	25.462	4.895	-1.062	1.00	0.00	C
ATOM	134	CD1	TYR	9	25.787	3.755	-1.827	1.00	0.00	C
ATOM	135	HD1	TYR	9	25.165	3.489	-2.669	1.00	0.00	H
ATOM	136	CE1	TYR	9	26.987	3.050	-1.572	1.00	0.00	C
ATOM	137	HE1	TYR	9	27.216	2.209	-2.210	1.00	0.00	H
ATOM	138	CZ	TYR	9	27.864	3.462	-0.586	1.00	0.00	C
ATOM	139	OH	TYR	9	28.978	2.819	-0.217	1.00	0.00	O
ATOM	140	HH	TYR	9	29.264	2.092	-0.775	1.00	0.00	H
ATOM	141	CE2	TYR	9	27.423	4.479	0.313	1.00	0.00	C
ATOM	142	HE2	TYR	9	28.063	4.771	1.132	1.00	0.00	H
ATOM	143	CD2	TYR	9	26.234	5.173	0.091	1.00	0.00	C
ATOM	144	HD2	TYR	9	25.969	5.931	0.814	1.00	0.00	H
ATOM	145	C	TYR	9	22.936	6.065	0.538	1.00	0.00	C
ATOM	146	O	TYR	9	22.605	5.314	1.503	1.00	0.00	O
ATOM	147	N	ARG	10	23.145	7.355	0.710	1.00	0.00	N
ATOM	148	H	ARG	10	23.501	7.943	-0.030	1.00	0.00	H
ATOM	149	CA	ARG	10	23.214	8.071	2.014	1.00	0.00	C
ATOM	150	HA	ARG	10	23.922	7.495	2.609	1.00	0.00	H
ATOM	151	CB	ARG	10	23.841	9.507	1.942	1.00	0.00	C
ATOM	152	HB2	ARG	10	24.704	9.376	1.290	1.00	0.00	H
ATOM	153	HB3	ARG	10	24.061	9.762	2.979	1.00	0.00	H
ATOM	154	CG	ARG	10	22.879	10.504	1.390	1.00	0.00	C
ATOM	155	HG2	ARG	10	21.828	10.415	1.666	1.00	0.00	H
ATOM	156	HG3	ARG	10	22.926	10.472	0.302	1.00	0.00	H
ATOM	157	CD	ARG	10	23.356	11.916	1.746	1.00	0.00	C
ATOM	158	HD2	ARG	10	24.388	12.004	1.406	1.00	0.00	H
ATOM	159	HD3	ARG	10	23.339	11.910	2.836	1.00	0.00	H
ATOM	160	NE	ARG	10	22.494	12.948	1.143	1.00	0.00	N
ATOM	161	HE	ARG	10	21.624	13.165	1.608	1.00	0.00	H
ATOM	162	CZ	ARG	10	22.773	13.675	0.072	1.00	0.00	C
ATOM	163	NH1	ARG	10	21.886	14.532	-0.327	1.00	0.00	N

ATOM	164	HH11	ARG	10	22.069	14.998	-1.204	1.00	0.00	H
ATOM	165	HH12	ARG	10	21.217	14.893	0.337	1.00	0.00	H
ATOM	166	NH2	ARG	10	23.765	13.578	-0.718	1.00	0.00	N
ATOM	167	HH21	ARG	10	24.350	12.756	-0.656	1.00	0.00	H
ATOM	168	HH22	ARG	10	23.946	14.287	-1.413	1.00	0.00	H
ATOM	169	C	ARG	10	21.901	8.016	2.859	1.00	0.00	C
ATOM	170	O	ARG	10	21.950	8.491	3.977	1.00	0.00	O
ATOM	171	N	GLU	11	20.807	7.520	2.370	1.00	0.00	N
ATOM	172	H	GLU	11	20.863	7.210	1.410	1.00	0.00	H
ATOM	173	CA	GLU	11	19.522	7.232	3.164	1.00	0.00	C
ATOM	174	HA	GLU	11	19.384	7.891	4.022	1.00	0.00	H
ATOM	175	CB	GLU	11	18.407	7.457	2.198	1.00	0.00	C
ATOM	176	HB2	GLU	11	18.588	8.415	1.712	1.00	0.00	H
ATOM	177	HB3	GLU	11	17.588	7.430	2.917	1.00	0.00	H
ATOM	178	CG	GLU	11	18.147	6.361	1.107	1.00	0.00	C
ATOM	179	HG2	GLU	11	18.155	5.361	1.541	1.00	0.00	H
ATOM	180	HG3	GLU	11	18.897	6.382	0.317	1.00	0.00	H
ATOM	181	CD	GLU	11	16.832	6.478	0.427	1.00	0.00	C
ATOM	182	OE1	GLU	11	16.182	5.425	0.147	1.00	0.00	O
ATOM	183	OE2	GLU	11	16.314	7.600	0.302	1.00	0.00	O
ATOM	184	C	GLU	11	19.494	5.817	3.826	1.00	0.00	C
ATOM	185	O	GLU	11	18.588	5.419	4.582	1.00	0.00	O
ATOM	186	N	ASN	12	20.488	4.977	3.525	1.00	0.00	N
ATOM	187	H	ASN	12	21.078	5.251	2.752	1.00	0.00	H
ATOM	188	CA	ASN	12	20.640	3.701	4.168	1.00	0.00	C
ATOM	189	HA	ASN	12	19.966	3.541	5.010	1.00	0.00	H
ATOM	190	CB	ASN	12	20.383	2.554	3.142	1.00	0.00	C
ATOM	191	HB2	ASN	12	20.999	2.754	2.265	1.00	0.00	H
ATOM	192	HB3	ASN	12	19.314	2.542	2.933	1.00	0.00	H
ATOM	193	CG	ASN	12	20.838	1.127	3.532	1.00	0.00	C
ATOM	194	OD1	ASN	12	20.837	0.747	4.752	1.00	0.00	O
ATOM	195	ND2	ASN	12	21.131	0.223	2.654	1.00	0.00	N
ATOM	196	HD21	ASN	12	21.391	-0.605	3.172	1.00	0.00	H
ATOM	197	HD22	ASN	12	21.277	0.582	1.722	1.00	0.00	H
ATOM	198	C	ASN	12	22.035	3.577	4.795	1.00	0.00	C
ATOM	199	O	ASN	12	22.052	3.296	5.963	1.00	0.00	O
ATOM	200	N	MET	13	23.141	3.738	4.001	1.00	0.00	N
ATOM	201	H	MET	13	23.022	3.906	3.012	1.00	0.00	H
ATOM	202	CA	MET	13	24.527	3.817	4.526	1.00	0.00	C
ATOM	203	HA	MET	13	24.669	3.016	5.252	1.00	0.00	H
ATOM	204	CB	MET	13	25.506	3.622	3.328	1.00	0.00	C
ATOM	205	HB2	MET	13	26.536	3.851	3.600	1.00	0.00	H
ATOM	206	HB3	MET	13	25.186	4.390	2.624	1.00	0.00	H
ATOM	207	CG	MET	13	25.613	2.205	2.729	1.00	0.00	C
ATOM	208	HG2	MET	13	25.690	1.380	3.437	1.00	0.00	H
ATOM	209	HG3	MET	13	26.565	2.277	2.203	1.00	0.00	H
ATOM	210	SD	MET	13	24.311	1.807	1.520	1.00	0.00	S
ATOM	211	CE	MET	13	24.979	0.295	0.752	1.00	0.00	C
ATOM	212	HE1	MET	13	25.475	-0.364	1.465	1.00	0.00	H
ATOM	213	HE2	MET	13	24.259	-0.270	0.159	1.00	0.00	H
ATOM	214	HE3	MET	13	25.808	0.645	0.137	1.00	0.00	H
ATOM	215	C	MET	13	24.699	5.117	5.220	1.00	0.00	C
ATOM	216	O	MET	13	24.689	6.227	4.629	1.00	0.00	O
ATOM	217	N	TYR	14	24.825	5.059	6.504	1.00	0.00	N
ATOM	218	H	TYR	14	24.488	4.219	6.953	1.00	0.00	H
ATOM	219	CA	TYR	14	25.033	6.271	7.267	1.00	0.00	C
ATOM	220	HA	TYR	14	24.589	7.153	6.805	1.00	0.00	H
ATOM	221	CB	TYR	14	24.490	6.135	8.702	1.00	0.00	C
ATOM	222	HB2	TYR	14	24.818	6.969	9.322	1.00	0.00	H
ATOM	223	HB3	TYR	14	25.018	5.288	9.140	1.00	0.00	H
ATOM	224	CG	TYR	14	22.982	5.820	8.964	1.00	0.00	C
ATOM	225	CD1	TYR	14	22.048	6.731	8.468	1.00	0.00	C
ATOM	226	HD1	TYR	14	22.407	7.626	7.983	1.00	0.00	H

ATOM	227	CE1	TYR	14	20.701	6.502	8.793	1.00	0.00	C
ATOM	228	HE1	TYR	14	19.959	7.199	8.432	1.00	0.00	H
ATOM	229	CZ	TYR	14	20.314	5.394	9.581	1.00	0.00	C
ATOM	230	OH	TYR	14	18.950	5.165	9.832	1.00	0.00	O
ATOM	231	HH	TYR	14	18.393	5.769	9.335	1.00	0.00	H
ATOM	232	CE2	TYR	14	21.270	4.427	9.976	1.00	0.00	C
ATOM	233	HE2	TYR	14	21.022	3.545	10.548	1.00	0.00	H
ATOM	234	CD2	TYR	14	22.612	4.600	9.589	1.00	0.00	C
ATOM	235	HD2	TYR	14	23.324	3.880	9.965	1.00	0.00	H
ATOM	236	C	TYR	14	26.486	6.706	7.378	1.00	0.00	C
ATOM	237	O	TYR	14	27.422	5.898	7.430	1.00	0.00	O
ATOM	238	N	ARG	15	26.627	8.059	7.190	1.00	0.00	N
ATOM	239	H	ARG	15	25.856	8.605	6.832	1.00	0.00	H
ATOM	240	CA	ARG	15	27.883	8.804	7.312	1.00	0.00	C
ATOM	241	HA	ARG	15	28.677	8.110	7.589	1.00	0.00	H
ATOM	242	CB	ARG	15	28.348	9.376	5.923	1.00	0.00	C
ATOM	243	HB2	ARG	15	29.394	9.644	6.078	1.00	0.00	H
ATOM	244	HB3	ARG	15	27.688	10.232	5.782	1.00	0.00	H
ATOM	245	CG	ARG	15	28.092	8.530	4.655	1.00	0.00	C
ATOM	246	HG2	ARG	15	27.028	8.337	4.523	1.00	0.00	H
ATOM	247	HG3	ARG	15	28.646	7.620	4.883	1.00	0.00	H
ATOM	248	CD	ARG	15	28.495	9.221	3.393	1.00	0.00	C
ATOM	249	HD2	ARG	15	29.503	9.622	3.504	1.00	0.00	H
ATOM	250	HD3	ARG	15	27.781	10.039	3.492	1.00	0.00	H
ATOM	251	NE	ARG	15	28.136	8.442	2.140	1.00	0.00	N
ATOM	252	HE	ARG	15	28.272	7.445	2.228	1.00	0.00	H
ATOM	253	CZ	ARG	15	27.665	8.865	0.988	1.00	0.00	C
ATOM	254	NH1	ARG	15	27.310	8.128	-0.003	1.00	0.00	N
ATOM	255	HH11	ARG	15	27.463	7.138	-0.128	1.00	0.00	H
ATOM	256	HH12	ARG	15	27.120	8.610	-0.871	1.00	0.00	H
ATOM	257	NH2	ARG	15	27.475	10.124	0.862	1.00	0.00	N
ATOM	258	HH21	ARG	15	27.637	10.684	1.687	1.00	0.00	H
ATOM	259	HH22	ARG	15	27.070	10.522	0.027	1.00	0.00	H
ATOM	260	C	ARG	15	27.691	9.817	8.448	1.00	0.00	C
ATOM	261	O	ARG	15	26.593	10.167	8.821	1.00	0.00	O
ATOM	262	N	TYR	16	28.855	10.306	8.914	1.00	0.00	N
ATOM	263	H	TYR	16	29.621	9.930	8.375	1.00	0.00	H
ATOM	264	CA	TYR	16	28.966	11.263	9.973	1.00	0.00	C
ATOM	265	HA	TYR	16	28.092	11.124	10.609	1.00	0.00	H
ATOM	266	CB	TYR	16	30.208	10.968	10.821	1.00	0.00	C
ATOM	267	HB2	TYR	16	30.119	9.996	11.305	1.00	0.00	H
ATOM	268	HB3	TYR	16	30.278	11.681	11.643	1.00	0.00	H
ATOM	269	CG	TYR	16	31.489	11.114	10.049	1.00	0.00	C
ATOM	270	CD1	TYR	16	32.011	10.064	9.231	1.00	0.00	C
ATOM	271	HD1	TYR	16	31.588	9.079	9.101	1.00	0.00	H
ATOM	272	CE1	TYR	16	33.244	10.172	8.581	1.00	0.00	C
ATOM	273	HE1	TYR	16	33.588	9.337	7.988	1.00	0.00	H
ATOM	274	CZ	TYR	16	34.085	11.264	8.801	1.00	0.00	C
ATOM	275	OH	TYR	16	35.199	11.395	8.049	1.00	0.00	O
ATOM	276	HH	TYR	16	35.734	12.155	8.289	1.00	0.00	H
ATOM	277	CE2	TYR	16	33.589	12.348	9.566	1.00	0.00	C
ATOM	278	HE2	TYR	16	34.142	13.276	9.558	1.00	0.00	H
ATOM	279	CD2	TYR	16	32.311	12.276	10.192	1.00	0.00	C
ATOM	280	HD2	TYR	16	31.966	13.058	10.852	1.00	0.00	H
ATOM	281	C	TYR	16	28.784	12.787	9.530	1.00	0.00	C
ATOM	282	O	TYR	16	28.672	13.693	10.389	1.00	0.00	O
ATOM	283	N	NME	17	28.614	12.973	8.240	1.00	0.00	N
ATOM	284	H	NME	17	28.793	12.132	7.709	1.00	0.00	H
ATOM	285	CH3	NME	17	28.638	14.256	7.505	1.00	0.00	C
ATOM	286	HH31	NME	17	29.582	14.289	6.963	1.00	0.00	H
ATOM	287	HH32	NME	17	27.803	14.233	6.805	1.00	0.00	H
ATOM	288	HH33	NME	17	28.697	15.115	8.172	1.00	0.00	H
TER	289		NME	17						

ATOM	289	HH31	ACE	18	42.350	8.322	15.853	1.00	0.00	H
ATOM	290	CH3	ACE	18	42.193	7.502	16.554	1.00	0.00	C
ATOM	291	HH32	ACE	18	41.284	7.634	17.140	1.00	0.00	H
ATOM	292	HH33	ACE	18	43.131	7.375	17.096	1.00	0.00	H
ATOM	293	C	ACE	18	42.007	6.315	15.572	1.00	0.00	C
ATOM	294	O	ACE	18	41.555	5.247	15.981	1.00	0.00	O
ATOM	295	N	HIE	19	42.417	6.515	14.328	1.00	0.00	N
ATOM	296	H	HIE	19	42.957	7.345	14.126	1.00	0.00	H
ATOM	297	CA	HIE	19	42.300	5.564	13.249	1.00	0.00	C
ATOM	298	HA	HIE	19	41.621	4.765	13.549	1.00	0.00	H
ATOM	299	CB	HIE	19	41.616	6.317	12.098	1.00	0.00	C
ATOM	300	HB2	HIE	19	42.256	7.090	11.671	1.00	0.00	H
ATOM	301	HB3	HIE	19	40.792	6.903	12.505	1.00	0.00	H
ATOM	302	CG	HIE	19	41.269	5.543	10.796	1.00	0.00	C
ATOM	303	ND1	HIE	19	42.168	5.295	9.808	1.00	0.00	N
ATOM	304	CE1	HIE	19	41.563	4.499	8.970	1.00	0.00	C
ATOM	305	HE1	HIE	19	42.100	4.115	8.115	1.00	0.00	H
ATOM	306	NE2	HIE	19	40.295	4.215	9.386	1.00	0.00	N
ATOM	307	HE2	HIE	19	39.604	3.567	9.036	1.00	0.00	H
ATOM	308	CD2	HIE	19	40.097	5.018	10.509	1.00	0.00	C
ATOM	309	HD2	HIE	19	39.300	4.988	11.238	1.00	0.00	H
ATOM	310	C	HIE	19	43.639	4.985	12.981	1.00	0.00	C
ATOM	311	O	HIE	19	44.658	5.199	13.684	1.00	0.00	O
ATOM	312	N	SER	20	43.728	4.142	11.961	1.00	0.00	N
ATOM	313	H	SER	20	42.939	4.274	11.345	1.00	0.00	H
ATOM	314	CA	SER	20	45.046	3.552	11.489	1.00	0.00	C
ATOM	315	HA	SER	20	45.566	3.200	12.380	1.00	0.00	H
ATOM	316	CB	SER	20	44.745	2.360	10.598	1.00	0.00	C
ATOM	317	HB2	SER	20	44.145	2.723	9.763	1.00	0.00	H
ATOM	318	HB3	SER	20	44.184	1.587	11.123	1.00	0.00	H
ATOM	319	OG	SER	20	45.865	1.742	10.072	1.00	0.00	O
ATOM	320	HG	SER	20	45.731	0.793	10.131	1.00	0.00	H
ATOM	321	C	SER	20	45.952	4.594	10.731	1.00	0.00	C
ATOM	322	O	SER	20	47.211	4.720	10.860	1.00	0.00	O
ATOM	323	N	GLN	21	45.256	5.542	10.071	1.00	0.00	N
ATOM	324	H	GLN	21	44.249	5.463	10.100	1.00	0.00	H
ATOM	325	CA	GLN	21	45.898	6.614	9.211	1.00	0.00	C
ATOM	326	HA	GLN	21	46.975	6.605	9.379	1.00	0.00	H
ATOM	327	CB	GLN	21	45.857	6.204	7.730	1.00	0.00	C
ATOM	328	HB2	GLN	21	46.129	6.924	6.959	1.00	0.00	H
ATOM	329	HB3	GLN	21	44.800	6.093	7.489	1.00	0.00	H
ATOM	330	CG	GLN	21	46.622	5.049	7.137	1.00	0.00	C
ATOM	331	HG2	GLN	21	47.599	4.946	7.609	1.00	0.00	H
ATOM	332	HG3	GLN	21	46.822	5.243	6.084	1.00	0.00	H
ATOM	333	CD	GLN	21	45.965	3.638	7.141	1.00	0.00	C
ATOM	334	OE1	GLN	21	44.828	3.549	7.515	1.00	0.00	O
ATOM	335	NE2	GLN	21	46.694	2.611	6.763	1.00	0.00	N
ATOM	336	HE21	GLN	21	47.635	2.752	6.423	1.00	0.00	H
ATOM	337	HE22	GLN	21	46.378	1.658	6.872	1.00	0.00	H
ATOM	338	C	GLN	21	45.404	8.046	9.460	1.00	0.00	C
ATOM	339	O	GLN	21	45.725	8.960	8.674	1.00	0.00	O
ATOM	340	N	TRP	22	44.622	8.319	10.537	1.00	0.00	N
ATOM	341	H	TRP	22	44.526	7.632	11.271	1.00	0.00	H
ATOM	342	CA	TRP	22	43.966	9.710	10.700	1.00	0.00	C
ATOM	343	HA	TRP	22	44.694	10.465	10.402	1.00	0.00	H
ATOM	344	CB	TRP	22	42.747	9.836	9.825	1.00	0.00	C
ATOM	345	HB2	TRP	22	41.924	9.148	10.021	1.00	0.00	H
ATOM	346	HB3	TRP	22	43.142	9.635	8.829	1.00	0.00	H
ATOM	347	CG	TRP	22	42.139	11.233	9.748	1.00	0.00	C
ATOM	348	CD1	TRP	22	42.823	12.285	9.259	1.00	0.00	C
ATOM	349	HD1	TRP	22	43.781	12.382	8.770	1.00	0.00	H
ATOM	350	NE1	TRP	22	41.985	13.431	9.426	1.00	0.00	N
ATOM	351	HE1	TRP	22	42.294	14.371	9.228	1.00	0.00	H

ATOM	352	CE2	TRP	22	40.773	13.163	10.013	1.00	0.00	C
ATOM	353	CZ2	TRP	22	39.658	13.932	10.209	1.00	0.00	C
ATOM	354	HZ2	TRP	22	39.620	15.010	10.181	1.00	0.00	H
ATOM	355	CH2	TRP	22	38.461	13.287	10.606	1.00	0.00	C
ATOM	356	HH2	TRP	22	37.582	13.861	10.858	1.00	0.00	H
ATOM	357	CZ3	TRP	22	38.390	11.869	10.725	1.00	0.00	C
ATOM	358	HZ3	TRP	22	37.452	11.401	10.981	1.00	0.00	H
ATOM	359	CE3	TRP	22	39.529	11.107	10.471	1.00	0.00	C
ATOM	360	HE3	TRP	22	39.420	10.033	10.437	1.00	0.00	H
ATOM	361	CD2	TRP	22	40.818	11.738	10.129	1.00	0.00	C
ATOM	362	C	TRP	22	43.771	9.960	12.206	1.00	0.00	C
ATOM	363	O	TRP	22	43.717	9.069	13.015	1.00	0.00	O
ATOM	364	N	ASN	23	43.836	11.219	12.702	1.00	0.00	N
ATOM	365	H	ASN	23	43.735	11.906	11.968	1.00	0.00	H
ATOM	366	CA	ASN	23	43.946	11.646	14.122	1.00	0.00	C
ATOM	367	HA	ASN	23	44.886	11.260	14.516	1.00	0.00	H
ATOM	368	CB	ASN	23	44.078	13.156	14.173	1.00	0.00	C
ATOM	369	HB2	ASN	23	43.304	13.638	13.574	1.00	0.00	H
ATOM	370	HB3	ASN	23	45.046	13.270	13.685	1.00	0.00	H
ATOM	371	CG	ASN	23	44.263	13.873	15.588	1.00	0.00	C
ATOM	372	OD1	ASN	23	43.733	13.512	16.648	1.00	0.00	O
ATOM	373	ND2	ASN	23	45.040	14.881	15.731	1.00	0.00	N
ATOM	374	HD21	ASN	23	45.019	15.414	16.589	1.00	0.00	H
ATOM	375	HD22	ASN	23	45.655	15.146	14.974	1.00	0.00	H
ATOM	376	C	ASN	23	42.743	11.294	15.000	1.00	0.00	C
ATOM	377	O	ASN	23	42.836	10.912	16.209	1.00	0.00	O
ATOM	378	N	LYS	24	41.559	11.345	14.390	1.00	0.00	N
ATOM	379	H	LYS	24	41.630	11.559	13.405	1.00	0.00	H
ATOM	380	CA	LYS	24	40.236	10.997	14.923	1.00	0.00	C
ATOM	381	HA	LYS	24	40.362	11.191	15.988	1.00	0.00	H
ATOM	382	CB	LYS	24	39.221	12.044	14.431	1.00	0.00	C
ATOM	383	HB2	LYS	24	38.212	11.832	14.785	1.00	0.00	H
ATOM	384	HB3	LYS	24	39.146	11.935	13.349	1.00	0.00	H
ATOM	385	CG	LYS	24	39.531	13.570	14.726	1.00	0.00	C
ATOM	386	HG2	LYS	24	40.339	13.975	14.117	1.00	0.00	H
ATOM	387	HG3	LYS	24	39.874	13.523	15.760	1.00	0.00	H
ATOM	388	CD	LYS	24	38.307	14.405	14.444	1.00	0.00	C
ATOM	389	HD2	LYS	24	37.411	13.948	14.866	1.00	0.00	H
ATOM	390	HD3	LYS	24	38.150	14.370	13.366	1.00	0.00	H
ATOM	391	CE	LYS	24	38.333	15.784	15.025	1.00	0.00	C
ATOM	392	HE2	LYS	24	39.143	16.404	14.641	1.00	0.00	H
ATOM	393	HE3	LYS	24	38.519	15.599	16.083	1.00	0.00	H
ATOM	394	NZ	LYS	24	37.095	16.566	14.893	1.00	0.00	N
ATOM	395	HZ1	LYS	24	37.118	17.416	15.437	1.00	0.00	H
ATOM	396	HZ2	LYS	24	36.818	16.744	13.938	1.00	0.00	H
ATOM	397	HZ3	LYS	24	36.261	16.103	15.225	1.00	0.00	H
ATOM	398	C	LYS	24	39.741	9.565	14.699	1.00	0.00	C
ATOM	399	O	LYS	24	40.184	8.930	13.755	1.00	0.00	O
ATOM	400	N	PRO	25	38.786	9.048	15.571	1.00	0.00	N
ATOM	401	CD	PRO	25	38.495	9.588	16.883	1.00	0.00	C
ATOM	402	HD2	PRO	25	39.365	9.456	17.526	1.00	0.00	H
ATOM	403	HD3	PRO	25	38.098	10.586	16.700	1.00	0.00	H
ATOM	404	CG	PRO	25	37.364	8.810	17.463	1.00	0.00	C
ATOM	405	HG2	PRO	25	37.417	8.846	18.551	1.00	0.00	H
ATOM	406	HG3	PRO	25	36.408	9.126	17.047	1.00	0.00	H
ATOM	407	CB	PRO	25	37.605	7.376	16.901	1.00	0.00	C
ATOM	408	HB2	PRO	25	38.436	6.977	17.482	1.00	0.00	H
ATOM	409	HB3	PRO	25	36.652	6.848	16.916	1.00	0.00	H
ATOM	410	CA	PRO	25	38.201	7.664	15.459	1.00	0.00	C
ATOM	411	HA	PRO	25	38.960	6.891	15.334	1.00	0.00	H
ATOM	412	C	PRO	25	37.192	7.363	14.350	1.00	0.00	C
ATOM	413	O	PRO	25	36.938	6.303	13.836	1.00	0.00	O
ATOM	414	N	SER	26	36.622	8.474	13.834	1.00	0.00	N

ATOM	415	H	SER	26	36.871	9.371	14.227	1.00	0.00	H
ATOM	416	CA	SER	26	35.528	8.450	12.814	1.00	0.00	C
ATOM	417	HA	SER	26	34.630	7.922	13.133	1.00	0.00	H
ATOM	418	CB	SER	26	34.894	9.899	12.693	1.00	0.00	C
ATOM	419	HB2	SER	26	33.805	9.870	12.697	1.00	0.00	H
ATOM	420	HB3	SER	26	35.277	10.414	11.812	1.00	0.00	H
ATOM	421	OG	SER	26	35.063	10.650	13.836	1.00	0.00	O
ATOM	422	HG	SER	26	34.829	11.568	13.679	1.00	0.00	H
ATOM	423	C	SER	26	35.918	7.930	11.422	1.00	0.00	C
ATOM	424	O	SER	26	36.899	8.347	10.849	1.00	0.00	O
ATOM	425	N	LYS	27	35.337	6.790	10.938	1.00	0.00	N
ATOM	426	H	LYS	27	34.639	6.327	11.501	1.00	0.00	H
ATOM	427	CA	LYS	27	35.582	6.218	9.584	1.00	0.00	C
ATOM	428	HA	LYS	27	36.454	6.806	9.298	1.00	0.00	H
ATOM	429	CB	LYS	27	36.067	4.797	9.808	1.00	0.00	C
ATOM	430	HB2	LYS	27	36.815	4.843	10.600	1.00	0.00	H
ATOM	431	HB3	LYS	27	36.631	4.549	8.909	1.00	0.00	H
ATOM	432	CG	LYS	27	35.180	3.529	9.915	1.00	0.00	C
ATOM	433	HG2	LYS	27	35.645	2.544	9.889	1.00	0.00	H
ATOM	434	HG3	LYS	27	34.426	3.685	9.142	1.00	0.00	H
ATOM	435	CD	LYS	27	34.482	3.456	11.273	1.00	0.00	C
ATOM	436	HD2	LYS	27	33.922	4.376	11.443	1.00	0.00	H
ATOM	437	HD3	LYS	27	35.301	3.366	11.986	1.00	0.00	H
ATOM	438	CE	LYS	27	33.494	2.286	11.542	1.00	0.00	C
ATOM	439	HE2	LYS	27	33.990	1.380	11.193	1.00	0.00	H
ATOM	440	HE3	LYS	27	32.630	2.460	10.901	1.00	0.00	H
ATOM	441	NZ	LYS	27	33.254	2.077	13.000	1.00	0.00	N
ATOM	442	HZ1	LYS	27	32.631	1.308	13.200	1.00	0.00	H
ATOM	443	HZ2	LYS	27	34.161	1.864	13.391	1.00	0.00	H
ATOM	444	HZ3	LYS	27	32.922	2.954	13.374	1.00	0.00	H
ATOM	445	C	LYS	27	34.450	6.512	8.599	1.00	0.00	C
ATOM	446	O	LYS	27	33.272	6.672	9.017	1.00	0.00	O
ATOM	447	N	PRO	28	34.723	6.576	7.267	1.00	0.00	N
ATOM	448	CD	PRO	28	36.028	6.645	6.679	1.00	0.00	C
ATOM	449	HD2	PRO	28	36.436	7.646	6.546	1.00	0.00	H
ATOM	450	HD3	PRO	28	36.685	5.893	7.116	1.00	0.00	H
ATOM	451	CG	PRO	28	35.769	6.072	5.283	1.00	0.00	C
ATOM	452	HG2	PRO	28	36.636	6.440	4.734	1.00	0.00	H
ATOM	453	HG3	PRO	28	35.725	4.983	5.296	1.00	0.00	H
ATOM	454	CB	PRO	28	34.336	6.598	4.961	1.00	0.00	C
ATOM	455	HB2	PRO	28	34.229	7.551	4.442	1.00	0.00	H
ATOM	456	HB3	PRO	28	33.846	5.826	4.368	1.00	0.00	H
ATOM	457	CA	PRO	28	33.642	6.547	6.309	1.00	0.00	C
ATOM	458	HA	PRO	28	33.033	7.417	6.556	1.00	0.00	H
ATOM	459	C	PRO	28	32.788	5.322	6.503	1.00	0.00	C
ATOM	460	O	PRO	28	33.131	4.227	6.972	1.00	0.00	O
ATOM	461	N	LYS	29	31.574	5.448	5.942	1.00	0.00	N
ATOM	462	H	LYS	29	31.427	6.268	5.371	1.00	0.00	H
ATOM	463	CA	LYS	29	30.461	4.462	5.991	1.00	0.00	C
ATOM	464	HA	LYS	29	29.539	5.015	5.812	1.00	0.00	H
ATOM	465	CB	LYS	29	30.653	3.581	4.730	1.00	0.00	C
ATOM	466	HB2	LYS	29	31.579	3.006	4.726	1.00	0.00	H
ATOM	467	HB3	LYS	29	30.722	4.416	4.033	1.00	0.00	H
ATOM	468	CG	LYS	29	29.526	2.662	4.420	1.00	0.00	C
ATOM	469	HG2	LYS	29	28.586	3.196	4.274	1.00	0.00	H
ATOM	470	HG3	LYS	29	29.522	1.887	5.186	1.00	0.00	H
ATOM	471	CD	LYS	29	29.855	2.123	2.969	1.00	0.00	C
ATOM	472	HD2	LYS	29	30.133	2.950	2.315	1.00	0.00	H
ATOM	473	HD3	LYS	29	28.889	1.743	2.637	1.00	0.00	H
ATOM	474	CE	LYS	29	31.026	1.101	2.950	1.00	0.00	C
ATOM	475	HE2	LYS	29	30.736	0.152	3.401	1.00	0.00	H
ATOM	476	HE3	LYS	29	31.845	1.597	3.471	1.00	0.00	H
ATOM	477	NZ	LYS	29	31.457	0.769	1.538	1.00	0.00	N

ATOM	478	HZ1	LYS	29	32.002	1.500	1.102	1.00	0.00	H
ATOM	479	HZ2	LYS	29	32.076	-0.026	1.609	1.00	0.00	H
ATOM	480	HZ3	LYS	29	30.654	0.461	1.008	1.00	0.00	H
ATOM	481	C	LYS	29	30.278	3.744	7.343	1.00	0.00	C
ATOM	482	O	LYS	29	30.427	2.539	7.457	1.00	0.00	O
ATOM	483	N	THR	30	29.991	4.529	8.394	1.00	0.00	N
ATOM	484	H	THR	30	29.826	5.509	8.214	1.00	0.00	H
ATOM	485	CA	THR	30	29.906	4.074	9.754	1.00	0.00	C
ATOM	486	HA	THR	30	30.905	3.787	10.082	1.00	0.00	H
ATOM	487	CB	THR	30	29.439	5.241	10.642	1.00	0.00	C
ATOM	488	HB	THR	30	28.352	5.247	10.711	1.00	0.00	H
ATOM	489	CG2	THR	30	30.131	5.044	11.969	1.00	0.00	C
ATOM	490	HG21	THR	30	29.917	4.052	12.365	1.00	0.00	H
ATOM	491	HG22	THR	30	31.212	5.184	11.941	1.00	0.00	H
ATOM	492	HG23	THR	30	29.714	5.820	12.611	1.00	0.00	H
ATOM	493	OG1	THR	30	29.800	6.543	10.234	1.00	0.00	O
ATOM	494	HG1	THR	30	29.783	7.088	11.025	1.00	0.00	H
ATOM	495	C	THR	30	29.093	2.771	9.936	1.00	0.00	C
ATOM	496	O	THR	30	29.626	1.896	10.641	1.00	0.00	O
ATOM	497	N	ASN	31	27.872	2.631	9.416	1.00	0.00	N
ATOM	498	H	ASN	31	27.453	3.362	8.858	1.00	0.00	H
ATOM	499	CA	ASN	31	27.075	1.428	9.508	1.00	0.00	C
ATOM	500	HA	ASN	31	27.748	0.582	9.369	1.00	0.00	H
ATOM	501	CB	ASN	31	26.543	1.381	10.903	1.00	0.00	C
ATOM	502	HB2	ASN	31	27.373	1.506	11.598	1.00	0.00	H
ATOM	503	HB3	ASN	31	26.201	0.350	10.988	1.00	0.00	H
ATOM	504	CG	ASN	31	25.473	2.322	11.410	1.00	0.00	C
ATOM	505	OD1	ASN	31	25.557	3.549	11.172	1.00	0.00	O
ATOM	506	ND2	ASN	31	24.442	1.945	12.184	1.00	0.00	N
ATOM	507	HD21	ASN	31	23.843	2.738	12.364	1.00	0.00	H
ATOM	508	HD22	ASN	31	24.197	0.986	12.384	1.00	0.00	H
ATOM	509	C	ASN	31	25.884	1.442	8.458	1.00	0.00	C
ATOM	510	O	ASN	31	25.717	2.369	7.736	1.00	0.00	O
ATOM	511	N	MET	32	25.072	0.407	8.418	1.00	0.00	N
ATOM	512	H	MET	32	25.086	-0.144	9.265	1.00	0.00	H
ATOM	513	CA	MET	32	23.990	0.090	7.541	1.00	0.00	C
ATOM	514	HA	MET	32	23.934	0.874	6.786	1.00	0.00	H
ATOM	515	CB	MET	32	24.178	-1.309	6.838	1.00	0.00	C
ATOM	516	HB2	MET	32	23.271	-1.649	6.337	1.00	0.00	H
ATOM	517	HB3	MET	32	24.195	-2.036	7.649	1.00	0.00	H
ATOM	518	CG	MET	32	25.391	-1.313	5.915	1.00	0.00	C
ATOM	519	HG2	MET	32	25.151	-0.732	5.024	1.00	0.00	H
ATOM	520	HG3	MET	32	25.345	-2.351	5.586	1.00	0.00	H
ATOM	521	SD	MET	32	27.108	-1.055	6.495	1.00	0.00	S
ATOM	522	CE	MET	32	27.923	-0.765	4.915	1.00	0.00	C
ATOM	523	HE1	MET	32	27.910	-1.625	4.246	1.00	0.00	H
ATOM	524	HE2	MET	32	27.352	0.074	4.517	1.00	0.00	H
ATOM	525	HE3	MET	32	28.938	-0.388	5.042	1.00	0.00	H
ATOM	526	C	MET	32	22.672	0.084	8.357	1.00	0.00	C
ATOM	527	O	MET	32	22.730	-0.416	9.466	1.00	0.00	O
ATOM	528	N	LYS	33	21.569	0.524	7.739	1.00	0.00	N
ATOM	529	H	LYS	33	21.648	0.849	6.786	1.00	0.00	H
ATOM	530	CA	LYS	33	20.184	0.530	8.189	1.00	0.00	C
ATOM	531	HA	LYS	33	20.200	0.644	9.273	1.00	0.00	H
ATOM	532	CB	LYS	33	19.450	1.683	7.648	1.00	0.00	C
ATOM	533	HB2	LYS	33	18.958	1.458	6.702	1.00	0.00	H
ATOM	534	HB3	LYS	33	20.217	2.386	7.325	1.00	0.00	H
ATOM	535	CG	LYS	33	18.478	2.402	8.600	1.00	0.00	C
ATOM	536	HG2	LYS	33	18.136	3.312	8.108	1.00	0.00	H
ATOM	537	HG3	LYS	33	19.106	2.603	9.468	1.00	0.00	H
ATOM	538	CD	LYS	33	17.268	1.549	9.019	1.00	0.00	C
ATOM	539	HD2	LYS	33	17.765	0.755	9.576	1.00	0.00	H
ATOM	540	HD3	LYS	33	16.801	1.155	8.116	1.00	0.00	H

```
ATOM    541  CE  LYS    33      16.262   2.329   9.959  1.00  0.00           C
ATOM    542  HE2 LYS    33      16.795   2.816  10.776  1.00  0.00           H
ATOM    543  HE3 LYS    33      15.614   1.509  10.269  1.00  0.00           H
ATOM    544  NZ  LYS    33      15.309   3.280   9.240  1.00  0.00           N
ATOM    545  HZ1 LYS    33      15.724   3.846   8.514  1.00  0.00           H
ATOM    546  HZ2 LYS    33      14.590   2.799   8.718  1.00  0.00           H
ATOM    547  HZ3 LYS    33      14.941   3.887   9.959  1.00  0.00           H
ATOM    548  C   LYS    33      19.485  -0.885   7.910  1.00  0.00           C
ATOM    549  O   LYS    33      19.155  -1.586   8.892  1.00  0.00           O
ATOM    550  N   NME    34      19.283  -1.178   6.607  1.00  0.00           N
ATOM    551  H   NME    34      19.773  -0.561   5.975  1.00  0.00           H
ATOM    552  CH3 NME    34      18.399  -2.172   5.993  1.00  0.00           C
ATOM    553 HH31 NME    34      19.064  -2.925   5.572  1.00  0.00           H
ATOM    554 HH32 NME    34      17.802  -2.590   6.803  1.00  0.00           H
ATOM    555 HH33 NME    34      17.637  -1.815   5.300  1.00  0.00           H
TER     556      NME    34
END
```

Cluster 3:

```
ATOM      1 HH31 ACE     1      19.333   7.936 -12.829  1.00  0.00           H
ATOM      2  CH3 ACE     1      18.797   7.344 -12.087  1.00  0.00           C
ATOM      3 HH32 ACE     1      19.065   6.288 -12.082  1.00  0.00           H
ATOM      4 HH33 ACE     1      17.710   7.383 -12.156  1.00  0.00           H
ATOM      5  C   ACE     1      19.192   7.766 -10.672  1.00  0.00           C
ATOM      6  O   ACE     1      19.711   8.875 -10.398  1.00  0.00           O
ATOM      7  N   ASN     2      18.928   6.884  -9.693  1.00  0.00           N
ATOM      8  H   ASN     2      18.667   5.942  -9.947  1.00  0.00           H
ATOM      9  CA  ASN     2      19.127   7.049  -8.206  1.00  0.00           C
ATOM     10  HA  ASN     2      18.518   7.925  -7.984  1.00  0.00           H
ATOM     11  CB  ASN     2      18.415   5.965  -7.434  1.00  0.00           C
ATOM     12  HB2 ASN     2      18.421   6.218  -6.374  1.00  0.00           H
ATOM     13  HB3 ASN     2      19.054   5.089  -7.544  1.00  0.00           H
ATOM     14  CG  ASN     2      17.075   5.444  -7.949  1.00  0.00           C
ATOM     15  OD1 ASN     2      16.989   4.264  -8.245  1.00  0.00           O
ATOM     16  ND2 ASN     2      16.125   6.249  -8.274  1.00  0.00           N
ATOM     17 HD21 ASN     2      15.321   5.854  -8.740  1.00  0.00           H
ATOM     18 HD22 ASN     2      16.189   7.257  -8.258  1.00  0.00           H
ATOM     19  C   ASN     2      20.591   7.250  -7.775  1.00  0.00           C
ATOM     20  O   ASN     2      20.827   7.560  -6.621  1.00  0.00           O
ATOM     21  N   ASP     3      21.541   7.180  -8.747  1.00  0.00           N
ATOM     22  H   ASP     3      21.145   6.843  -9.613  1.00  0.00           H
ATOM     23  CA  ASP     3      22.961   7.412  -8.510  1.00  0.00           C
ATOM     24  HA  ASP     3      23.479   6.891  -7.704  1.00  0.00           H
ATOM     25  CB  ASP     3      23.677   6.970  -9.830  1.00  0.00           C
ATOM     26  HB2 ASP     3      23.101   7.341 -10.678  1.00  0.00           H
ATOM     27  HB3 ASP     3      23.547   5.888  -9.837  1.00  0.00           H
ATOM     28  CG  ASP     3      25.166   7.372  -9.887  1.00  0.00           C
ATOM     29  OD1 ASP     3      26.003   6.851  -9.121  1.00  0.00           O
ATOM     30  OD2 ASP     3      25.477   8.326 -10.629  1.00  0.00           O
ATOM     31  C   ASP     3      23.332   8.920  -8.238  1.00  0.00           C
ATOM     32  O   ASP     3      24.265   9.309  -7.557  1.00  0.00           O
ATOM     33  N   TYR     4      22.588   9.817  -8.790  1.00  0.00           N
ATOM     34  H   TYR     4      22.019   9.621  -9.601  1.00  0.00           H
ATOM     35  CA  TYR     4      22.666  11.248  -8.576  1.00  0.00           C
ATOM     36  HA  TYR     4      23.560  11.629  -9.069  1.00  0.00           H
ATOM     37  CB  TYR     4      21.549  11.967  -9.264  1.00  0.00           C
ATOM     38  HB2 TYR     4      21.054  11.371 -10.030  1.00  0.00           H
ATOM     39  HB3 TYR     4      22.025  12.793  -9.792  1.00  0.00           H
ATOM     40  CG  TYR     4      20.523  12.731  -8.450  1.00  0.00           C
ATOM     41  CD1 TYR     4      19.303  12.112  -8.017  1.00  0.00           C
```

ATOM	42	HD1	TYR	4	19.087	11.055	-8.082	1.00	0.00	H
ATOM	43	CE1	TYR	4	18.400	12.887	-7.274	1.00	0.00	C
ATOM	44	HE1	TYR	4	17.521	12.370	-6.919	1.00	0.00	H
ATOM	45	CZ	TYR	4	18.710	14.195	-6.967	1.00	0.00	C
ATOM	46	OH	TYR	4	17.814	14.953	-6.251	1.00	0.00	O
ATOM	47	HH	TYR	4	16.955	14.556	-6.412	1.00	0.00	H
ATOM	48	CE2	TYR	4	19.969	14.744	-7.232	1.00	0.00	C
ATOM	49	HE2	TYR	4	20.204	15.742	-6.893	1.00	0.00	H
ATOM	50	CD2	TYR	4	20.829	14.067	-8.057	1.00	0.00	C
ATOM	51	HD2	TYR	4	21.772	14.502	-8.355	1.00	0.00	H
ATOM	52	C	TYR	4	22.844	11.699	-7.092	1.00	0.00	C
ATOM	53	O	TYR	4	23.622	12.617	-6.859	1.00	0.00	O
ATOM	54	N	GLU	5	22.225	11.076	-6.089	1.00	0.00	N
ATOM	55	H	GLU	5	21.432	10.482	-6.289	1.00	0.00	H
ATOM	56	CA	GLU	5	22.377	11.497	-4.671	1.00	0.00	C
ATOM	57	HA	GLU	5	22.499	12.578	-4.602	1.00	0.00	H
ATOM	58	CB	GLU	5	21.247	10.932	-3.789	1.00	0.00	C
ATOM	59	HB2	GLU	5	21.366	11.366	-2.796	1.00	0.00	H
ATOM	60	HB3	GLU	5	21.204	9.844	-3.748	1.00	0.00	H
ATOM	61	CG	GLU	5	19.883	11.405	-4.352	1.00	0.00	C
ATOM	62	HG2	GLU	5	19.542	10.575	-4.971	1.00	0.00	H
ATOM	63	HG3	GLU	5	20.147	12.274	-4.955	1.00	0.00	H
ATOM	64	CD	GLU	5	18.875	11.753	-3.327	1.00	0.00	C
ATOM	65	OE1	GLU	5	17.843	12.323	-3.664	1.00	0.00	O
ATOM	66	OE2	GLU	5	18.947	11.358	-2.160	1.00	0.00	O
ATOM	67	C	GLU	5	23.653	10.988	-3.964	1.00	0.00	C
ATOM	68	O	GLU	5	24.183	11.659	-3.125	1.00	0.00	O
ATOM	69	N	ASP	6	24.271	9.892	-4.499	1.00	0.00	N
ATOM	70	H	ASP	6	23.728	9.488	-5.249	1.00	0.00	H
ATOM	71	CA	ASP	6	25.442	9.375	-3.950	1.00	0.00	C
ATOM	72	HA	ASP	6	25.424	9.587	-2.881	1.00	0.00	H
ATOM	73	CB	ASP	6	25.454	7.866	-4.193	1.00	0.00	C
ATOM	74	HB2	ASP	6	25.532	7.559	-5.236	1.00	0.00	H
ATOM	75	HB3	ASP	6	24.627	7.492	-3.589	1.00	0.00	H
ATOM	76	CG	ASP	6	26.786	7.203	-3.604	1.00	0.00	C
ATOM	77	OD1	ASP	6	27.599	6.663	-4.361	1.00	0.00	O
ATOM	78	OD2	ASP	6	27.071	7.267	-2.339	1.00	0.00	O
ATOM	79	C	ASP	6	26.740	10.107	-4.305	1.00	0.00	C
ATOM	80	O	ASP	6	27.814	9.950	-3.702	1.00	0.00	O
ATOM	81	N	ARG	7	26.629	11.016	-5.307	1.00	0.00	N
ATOM	82	H	ARG	7	25.713	11.113	-5.721	1.00	0.00	H
ATOM	83	CA	ARG	7	27.692	11.836	-5.865	1.00	0.00	C
ATOM	84	HA	ARG	7	28.553	11.178	-5.983	1.00	0.00	H
ATOM	85	CB	ARG	7	27.215	12.417	-7.284	1.00	0.00	C
ATOM	86	HB2	ARG	7	28.033	12.882	-7.835	1.00	0.00	H
ATOM	87	HB3	ARG	7	26.535	13.222	-7.005	1.00	0.00	H
ATOM	88	CG	ARG	7	26.744	11.403	-8.312	1.00	0.00	C
ATOM	89	HG2	ARG	7	25.984	10.774	-7.847	1.00	0.00	H
ATOM	90	HG3	ARG	7	27.679	10.883	-8.521	1.00	0.00	H
ATOM	91	CD	ARG	7	26.158	12.108	-9.547	1.00	0.00	C
ATOM	92	HD2	ARG	7	26.951	12.646	-10.066	1.00	0.00	H
ATOM	93	HD3	ARG	7	25.329	12.738	-9.225	1.00	0.00	H
ATOM	94	NE	ARG	7	25.853	11.096	-10.647	1.00	0.00	N
ATOM	95	HE	ARG	7	25.512	10.199	-10.332	1.00	0.00	H
ATOM	96	CZ	ARG	7	26.182	11.246	-11.949	1.00	0.00	C
ATOM	97	NH1	ARG	7	26.451	12.442	-12.402	1.00	0.00	N
ATOM	98	HH11	ARG	7	26.559	12.656	-13.383	1.00	0.00	H
ATOM	99	HH12	ARG	7	26.112	13.127	-11.742	1.00	0.00	H
ATOM	100	NH2	ARG	7	26.198	10.296	-12.826	1.00	0.00	N
ATOM	101	HH21	ARG	7	26.533	10.502	-13.756	1.00	0.00	H
ATOM	102	HH22	ARG	7	25.939	9.371	-12.514	1.00	0.00	H
ATOM	103	C	ARG	7	28.209	12.984	-5.037	1.00	0.00	C
ATOM	104	O	ARG	7	29.411	13.256	-5.018	1.00	0.00	O

ATOM	105	N	TYR	8	27.351	13.643	-4.258	1.00	0.00	N
ATOM	106	H	TYR	8	26.438	13.221	-4.168	1.00	0.00	H
ATOM	107	CA	TYR	8	27.733	14.667	-3.313	1.00	0.00	C
ATOM	108	HA	TYR	8	28.230	15.432	-3.911	1.00	0.00	H
ATOM	109	CB	TYR	8	26.452	15.236	-2.586	1.00	0.00	C
ATOM	110	HB2	TYR	8	26.769	15.908	-1.788	1.00	0.00	H
ATOM	111	HB3	TYR	8	25.885	14.417	-2.144	1.00	0.00	H
ATOM	112	CG	TYR	8	25.624	16.145	-3.470	1.00	0.00	C
ATOM	113	CD1	TYR	8	26.233	17.356	-3.839	1.00	0.00	C
ATOM	114	HD1	TYR	8	27.199	17.611	-3.429	1.00	0.00	H
ATOM	115	CE1	TYR	8	25.596	18.200	-4.620	1.00	0.00	C
ATOM	116	HE1	TYR	8	26.046	19.133	-4.924	1.00	0.00	H
ATOM	117	CZ	TYR	8	24.345	17.854	-5.167	1.00	0.00	C
ATOM	118	OH	TYR	8	23.741	18.638	-6.090	1.00	0.00	O
ATOM	119	HH	TYR	8	24.158	19.501	-6.140	1.00	0.00	H
ATOM	120	CE2	TYR	8	23.714	16.631	-4.884	1.00	0.00	C
ATOM	121	HE2	TYR	8	22.724	16.449	-5.276	1.00	0.00	H
ATOM	122	CD2	TYR	8	24.348	15.796	-4.017	1.00	0.00	C
ATOM	123	HD2	TYR	8	23.965	14.830	-3.724	1.00	0.00	H
ATOM	124	C	TYR	8	28.765	14.290	-2.217	1.00	0.00	C
ATOM	125	O	TYR	8	29.554	15.106	-1.747	1.00	0.00	O
ATOM	126	N	TYR	9	28.648	13.026	-1.838	1.00	0.00	N
ATOM	127	H	TYR	9	27.817	12.526	-2.121	1.00	0.00	H
ATOM	128	CA	TYR	9	29.618	12.300	-1.035	1.00	0.00	C
ATOM	129	HA	TYR	9	29.930	13.026	-0.285	1.00	0.00	H
ATOM	130	CB	TYR	9	28.956	11.101	-0.306	1.00	0.00	C
ATOM	131	HB2	TYR	9	29.713	10.697	0.367	1.00	0.00	H
ATOM	132	HB3	TYR	9	28.696	10.520	-1.190	1.00	0.00	H
ATOM	133	CG	TYR	9	27.665	11.439	0.429	1.00	0.00	C
ATOM	134	CD1	TYR	9	27.660	12.365	1.486	1.00	0.00	C
ATOM	135	HD1	TYR	9	28.589	12.768	1.861	1.00	0.00	H
ATOM	136	CE1	TYR	9	26.464	12.795	2.028	1.00	0.00	C
ATOM	137	HE1	TYR	9	26.399	13.544	2.803	1.00	0.00	H
ATOM	138	CZ	TYR	9	25.284	12.250	1.531	1.00	0.00	C
ATOM	139	OH	TYR	9	24.063	12.860	1.945	1.00	0.00	O
ATOM	140	HH	TYR	9	24.285	13.489	2.635	1.00	0.00	H
ATOM	141	CE2	TYR	9	25.269	11.445	0.359	1.00	0.00	C
ATOM	142	HE2	TYR	9	24.341	11.043	-0.018	1.00	0.00	H
ATOM	143	CD2	TYR	9	26.478	10.973	-0.188	1.00	0.00	C
ATOM	144	HD2	TYR	9	26.442	10.268	-1.006	1.00	0.00	H
ATOM	145	C	TYR	9	30.855	11.804	-1.758	1.00	0.00	C
ATOM	146	O	TYR	9	31.970	11.683	-1.226	1.00	0.00	O
ATOM	147	N	ARG	10	30.732	11.217	-2.985	1.00	0.00	N
ATOM	148	H	ARG	10	29.783	11.135	-3.318	1.00	0.00	H
ATOM	149	CA	ARG	10	31.762	10.657	-3.923	1.00	0.00	C
ATOM	150	HA	ARG	10	32.369	9.910	-3.411	1.00	0.00	H
ATOM	151	CB	ARG	10	31.097	9.954	-5.127	1.00	0.00	C
ATOM	152	HB2	ARG	10	30.653	10.719	-5.765	1.00	0.00	H
ATOM	153	HB3	ARG	10	30.351	9.309	-4.663	1.00	0.00	H
ATOM	154	CG	ARG	10	31.911	8.991	-6.018	1.00	0.00	C
ATOM	155	HG2	ARG	10	32.404	8.200	-5.453	1.00	0.00	H
ATOM	156	HG3	ARG	10	32.653	9.626	-6.503	1.00	0.00	H
ATOM	157	CD	ARG	10	31.078	8.430	-7.179	1.00	0.00	C
ATOM	158	HD2	ARG	10	31.670	7.711	-7.745	1.00	0.00	H
ATOM	159	HD3	ARG	10	30.852	9.303	-7.792	1.00	0.00	H
ATOM	160	NE	ARG	10	29.796	7.777	-6.776	1.00	0.00	N
ATOM	161	HE	ARG	10	29.649	7.612	-5.790	1.00	0.00	H
ATOM	162	CZ	ARG	10	28.786	7.481	-7.570	1.00	0.00	C
ATOM	163	NH1	ARG	10	27.678	7.193	-7.059	1.00	0.00	N
ATOM	164	HH11	ARG	10	26.935	6.993	-7.713	1.00	0.00	H
ATOM	165	HH12	ARG	10	27.503	7.164	-6.065	1.00	0.00	H
ATOM	166	NH2	ARG	10	28.852	7.531	-8.840	1.00	0.00	N
ATOM	167	HH21	ARG	10	29.728	7.889	-9.192	1.00	0.00	H

ATOM	168	HH22	ARG	10	28.038	7.347	-9.408	1.00	0.00	H
ATOM	169	C	ARG	10	32.680	11.762	-4.441	1.00	0.00	C
ATOM	170	O	ARG	10	33.883	11.475	-4.480	1.00	0.00	O
ATOM	171	N	GLU	11	32.189	12.914	-4.800	1.00	0.00	N
ATOM	172	H	GLU	11	31.181	12.971	-4.822	1.00	0.00	H
ATOM	173	CA	GLU	11	33.005	14.106	-5.230	1.00	0.00	C
ATOM	174	HA	GLU	11	33.738	13.757	-5.958	1.00	0.00	H
ATOM	175	CB	GLU	11	32.059	15.126	-5.977	1.00	0.00	C
ATOM	176	HB2	GLU	11	31.388	15.634	-5.284	1.00	0.00	H
ATOM	177	HB3	GLU	11	31.470	14.438	-6.582	1.00	0.00	H
ATOM	178	CG	GLU	11	32.641	16.185	-6.929	1.00	0.00	C
ATOM	179	HG2	GLU	11	33.305	15.721	-7.658	1.00	0.00	H
ATOM	180	HG3	GLU	11	33.186	16.890	-6.301	1.00	0.00	H
ATOM	181	CD	GLU	11	31.647	16.980	-7.729	1.00	0.00	C
ATOM	182	OE1	GLU	11	30.753	16.404	-8.428	1.00	0.00	O
ATOM	183	OE2	GLU	11	31.707	18.229	-7.690	1.00	0.00	O
ATOM	184	C	GLU	11	33.595	14.937	-4.073	1.00	0.00	C
ATOM	185	O	GLU	11	34.544	15.712	-4.246	1.00	0.00	O
ATOM	186	N	ASN	12	33.281	14.561	-2.790	1.00	0.00	N
ATOM	187	H	ASN	12	32.678	13.762	-2.656	1.00	0.00	H
ATOM	188	CA	ASN	12	33.893	15.302	-1.601	1.00	0.00	C
ATOM	189	HA	ASN	12	33.971	16.342	-1.918	1.00	0.00	H
ATOM	190	CB	ASN	12	32.938	15.228	-0.423	1.00	0.00	C
ATOM	191	HB2	ASN	12	32.975	14.254	0.064	1.00	0.00	H
ATOM	192	HB3	ASN	12	31.924	15.291	-0.819	1.00	0.00	H
ATOM	193	CG	ASN	12	33.196	16.356	0.561	1.00	0.00	C
ATOM	194	OD1	ASN	12	32.998	17.498	0.138	1.00	0.00	O
ATOM	195	ND2	ASN	12	33.651	16.223	1.743	1.00	0.00	N
ATOM	196	HD21	ASN	12	33.703	17.074	2.284	1.00	0.00	H
ATOM	197	HD22	ASN	12	33.643	15.328	2.211	1.00	0.00	H
ATOM	198	C	ASN	12	35.317	14.745	-1.179	1.00	0.00	C
ATOM	199	O	ASN	12	36.064	15.370	-0.412	1.00	0.00	O
ATOM	200	N	MET	13	35.631	13.524	-1.665	1.00	0.00	N
ATOM	201	H	MET	13	34.881	13.014	-2.109	1.00	0.00	H
ATOM	202	CA	MET	13	36.796	12.756	-1.301	1.00	0.00	C
ATOM	203	HA	MET	13	37.162	12.957	-0.294	1.00	0.00	H
ATOM	204	CB	MET	13	36.623	11.254	-1.555	1.00	0.00	C
ATOM	205	HB2	MET	13	37.546	10.719	-1.332	1.00	0.00	H
ATOM	206	HB3	MET	13	36.345	11.000	-2.578	1.00	0.00	H
ATOM	207	CG	MET	13	35.460	10.627	-0.703	1.00	0.00	C
ATOM	208	HG2	MET	13	35.554	9.565	-0.932	1.00	0.00	H
ATOM	209	HG3	MET	13	34.538	10.993	-1.155	1.00	0.00	H
ATOM	210	SD	MET	13	35.407	10.811	1.025	1.00	0.00	S
ATOM	211	CE	MET	13	33.691	10.127	1.440	1.00	0.00	C
ATOM	212	HE1	MET	13	33.713	9.904	2.507	1.00	0.00	H
ATOM	213	HE2	MET	13	32.941	10.909	1.316	1.00	0.00	H
ATOM	214	HE3	MET	13	33.641	9.182	0.900	1.00	0.00	H
ATOM	215	C	MET	13	37.973	13.188	-2.145	1.00	0.00	C
ATOM	216	O	MET	13	39.083	13.155	-1.648	1.00	0.00	O
ATOM	217	N	TYR	14	37.805	13.442	-3.438	1.00	0.00	N
ATOM	218	H	TYR	14	36.878	13.573	-3.819	1.00	0.00	H
ATOM	219	CA	TYR	14	38.905	13.739	-4.339	1.00	0.00	C
ATOM	220	HA	TYR	14	39.795	13.315	-3.873	1.00	0.00	H
ATOM	221	CB	TYR	14	38.768	13.089	-5.722	1.00	0.00	C
ATOM	222	HB2	TYR	14	39.488	13.538	-6.407	1.00	0.00	H
ATOM	223	HB3	TYR	14	37.768	13.449	-5.962	1.00	0.00	H
ATOM	224	CG	TYR	14	38.808	11.581	-5.894	1.00	0.00	C
ATOM	225	CD1	TYR	14	38.676	11.133	-7.212	1.00	0.00	C
ATOM	226	HD1	TYR	14	38.482	11.797	-8.041	1.00	0.00	H
ATOM	227	CE1	TYR	14	38.930	9.789	-7.545	1.00	0.00	C
ATOM	228	HE1	TYR	14	38.812	9.534	-8.588	1.00	0.00	H
ATOM	229	CZ	TYR	14	39.378	8.831	-6.522	1.00	0.00	C
ATOM	230	OH	TYR	14	39.496	7.531	-6.878	1.00	0.00	O

ATOM	231	HH	TYR	14	40.117	7.183	-6.234	1.00	0.00	H
ATOM	232	CE2	TYR	14	39.559	9.365	-5.212	1.00	0.00	C
ATOM	233	HE2	TYR	14	39.803	8.651	-4.440	1.00	0.00	H
ATOM	234	CD2	TYR	14	39.261	10.740	-4.915	1.00	0.00	C
ATOM	235	HD2	TYR	14	39.536	11.111	-3.939	1.00	0.00	H
ATOM	236	C	TYR	14	39.153	15.254	-4.559	1.00	0.00	C
ATOM	237	O	TYR	14	40.266	15.655	-4.786	1.00	0.00	O
ATOM	238	N	ARG	15	38.115	16.013	-4.467	1.00	0.00	N
ATOM	239	H	ARG	15	37.251	15.499	-4.364	1.00	0.00	H
ATOM	240	CA	ARG	15	38.176	17.500	-4.732	1.00	0.00	C
ATOM	241	HA	ARG	15	38.770	17.673	-5.629	1.00	0.00	H
ATOM	242	CB	ARG	15	36.789	18.009	-4.992	1.00	0.00	C
ATOM	243	HB2	ARG	15	36.274	18.104	-4.036	1.00	0.00	H
ATOM	244	HB3	ARG	15	36.265	17.284	-5.614	1.00	0.00	H
ATOM	245	CG	ARG	15	36.511	19.355	-5.707	1.00	0.00	C
ATOM	246	HG2	ARG	15	37.124	20.120	-5.231	1.00	0.00	H
ATOM	247	HG3	ARG	15	35.444	19.541	-5.585	1.00	0.00	H
ATOM	248	CD	ARG	15	36.830	19.436	-7.193	1.00	0.00	C
ATOM	249	HD2	ARG	15	36.271	18.694	-7.763	1.00	0.00	H
ATOM	250	HD3	ARG	15	37.855	19.073	-7.261	1.00	0.00	H
ATOM	251	NE	ARG	15	36.705	20.876	-7.617	1.00	0.00	N
ATOM	252	HE	ARG	15	35.786	21.126	-7.954	1.00	0.00	H
ATOM	253	CZ	ARG	15	37.653	21.758	-7.574	1.00	0.00	C
ATOM	254	NH1	ARG	15	37.431	22.976	-7.792	1.00	0.00	N
ATOM	255	HH11	ARG	15	38.199	23.605	-7.607	1.00	0.00	H
ATOM	256	HH12	ARG	15	36.469	23.269	-7.885	1.00	0.00	H
ATOM	257	NH2	ARG	15	38.924	21.545	-7.170	1.00	0.00	N
ATOM	258	HH21	ARG	15	39.160	20.670	-6.724	1.00	0.00	H
ATOM	259	HH22	ARG	15	39.564	22.327	-7.173	1.00	0.00	H
ATOM	260	C	ARG	15	38.921	18.346	-3.607	1.00	0.00	C
ATOM	261	O	ARG	15	39.383	19.439	-3.986	1.00	0.00	O
ATOM	262	N	TYR	16	39.060	17.820	-2.430	1.00	0.00	N
ATOM	263	H	TYR	16	38.729	16.869	-2.350	1.00	0.00	H
ATOM	264	CA	TYR	16	39.539	18.429	-1.200	1.00	0.00	C
ATOM	265	HA	TYR	16	39.070	19.400	-1.046	1.00	0.00	H
ATOM	266	CB	TYR	16	39.135	17.499	0.045	1.00	0.00	C
ATOM	267	HB2	TYR	16	39.556	16.497	-0.033	1.00	0.00	H
ATOM	268	HB3	TYR	16	38.063	17.375	-0.109	1.00	0.00	H
ATOM	269	CG	TYR	16	39.225	18.078	1.431	1.00	0.00	C
ATOM	270	CD1	TYR	16	40.029	17.440	2.395	1.00	0.00	C
ATOM	271	HD1	TYR	16	40.613	16.576	2.115	1.00	0.00	H
ATOM	272	CE1	TYR	16	40.065	17.913	3.736	1.00	0.00	C
ATOM	273	HE1	TYR	16	40.736	17.505	4.477	1.00	0.00	H
ATOM	274	CZ	TYR	16	39.419	19.130	3.995	1.00	0.00	C
ATOM	275	OH	TYR	16	39.269	19.553	5.276	1.00	0.00	O
ATOM	276	HH	TYR	16	39.676	18.882	5.829	1.00	0.00	H
ATOM	277	CE2	TYR	16	38.685	19.765	3.045	1.00	0.00	C
ATOM	278	HE2	TYR	16	37.999	20.585	3.198	1.00	0.00	H
ATOM	279	CD2	TYR	16	38.453	19.162	1.776	1.00	0.00	C
ATOM	280	HD2	TYR	16	37.654	19.525	1.146	1.00	0.00	H
ATOM	281	C	TYR	16	41.074	18.608	-1.266	1.00	0.00	C
ATOM	282	O	TYR	16	41.747	18.157	-2.163	1.00	0.00	O
ATOM	283	N	NME	17	41.578	19.517	-0.432	1.00	0.00	N
ATOM	284	H	NME	17	40.920	19.677	0.317	1.00	0.00	H
ATOM	285	CH3	NME	17	42.949	19.922	-0.337	1.00	0.00	C
ATOM	286	HH31	NME	17	43.272	20.459	0.555	1.00	0.00	H
ATOM	287	HH32	NME	17	43.532	19.005	-0.422	1.00	0.00	H
ATOM	288	HH33	NME	17	43.296	20.489	-1.201	1.00	0.00	H
TER	289		NME	17						
ATOM	289	HH31	ACE	18	45.577	0.987	11.099	1.00	0.00	H
ATOM	290	CH3	ACE	18	45.623	-0.000	11.558	1.00	0.00	C
ATOM	291	HH32	ACE	18	45.675	0.127	12.639	1.00	0.00	H
ATOM	292	HH33	ACE	18	46.510	-0.499	11.168	1.00	0.00	H

ATOM	293	C	ACE	18	44.331	-0.758	11.170	1.00	0.00	C
ATOM	294	O	ACE	18	43.659	-0.343	10.226	1.00	0.00	O
ATOM	295	N	HIE	19	43.949	-1.813	11.869	1.00	0.00	N
ATOM	296	H	HIE	19	44.465	-2.074	12.697	1.00	0.00	H
ATOM	297	CA	HIE	19	42.619	-2.428	11.767	1.00	0.00	C
ATOM	298	HA	HIE	19	41.835	-1.671	11.746	1.00	0.00	H
ATOM	299	CB	HIE	19	42.536	-3.284	10.497	1.00	0.00	C
ATOM	300	HB2	HIE	19	43.400	-3.941	10.594	1.00	0.00	H
ATOM	301	HB3	HIE	19	42.629	-2.559	9.689	1.00	0.00	H
ATOM	302	CG	HIE	19	41.298	-4.119	10.305	1.00	0.00	C
ATOM	303	ND1	HIE	19	40.037	-3.569	10.091	1.00	0.00	N
ATOM	304	CE1	HIE	19	39.258	-4.591	9.650	1.00	0.00	C
ATOM	305	HE1	HIE	19	38.188	-4.620	9.506	1.00	0.00	H
ATOM	306	NE2	HIE	19	39.978	-5.725	9.715	1.00	0.00	N
ATOM	307	HE2	HIE	19	39.575	-6.648	9.645	1.00	0.00	H
ATOM	308	CD2	HIE	19	41.252	-5.441	10.057	1.00	0.00	C
ATOM	309	HD2	HIE	19	42.039	-6.172	10.166	1.00	0.00	H
ATOM	310	C	HIE	19	42.310	-3.130	13.113	1.00	0.00	C
ATOM	311	O	HIE	19	43.258	-3.556	13.782	1.00	0.00	O
ATOM	312	N	SER	20	41.044	-3.239	13.575	1.00	0.00	N
ATOM	313	H	SER	20	40.298	-2.905	12.981	1.00	0.00	H
ATOM	314	CA	SER	20	40.653	-3.631	14.935	1.00	0.00	C
ATOM	315	HA	SER	20	41.410	-4.198	15.476	1.00	0.00	H
ATOM	316	CB	SER	20	40.420	-2.356	15.643	1.00	0.00	C
ATOM	317	HB2	SER	20	39.497	-1.819	15.423	1.00	0.00	H
ATOM	318	HB3	SER	20	41.323	-1.765	15.490	1.00	0.00	H
ATOM	319	OG	SER	20	40.416	-2.613	17.026	1.00	0.00	O
ATOM	320	HG	SER	20	40.610	-1.789	17.479	1.00	0.00	H
ATOM	321	C	SER	20	39.439	-4.603	14.941	1.00	0.00	C
ATOM	322	O	SER	20	38.976	-5.080	13.925	1.00	0.00	O
ATOM	323	N	GLN	21	38.819	-4.773	16.113	1.00	0.00	N
ATOM	324	H	GLN	21	39.219	-4.175	16.823	1.00	0.00	H
ATOM	325	CA	GLN	21	37.725	-5.678	16.311	1.00	0.00	C
ATOM	326	HA	GLN	21	37.737	-6.438	15.529	1.00	0.00	H
ATOM	327	CB	GLN	21	37.959	-6.442	17.638	1.00	0.00	C
ATOM	328	HB2	GLN	21	37.216	-7.218	17.824	1.00	0.00	H
ATOM	329	HB3	GLN	21	37.835	-5.636	18.362	1.00	0.00	H
ATOM	330	CG	GLN	21	39.199	-7.251	17.750	1.00	0.00	C
ATOM	331	HG2	GLN	21	40.118	-6.762	17.426	1.00	0.00	H
ATOM	332	HG3	GLN	21	38.986	-8.073	17.067	1.00	0.00	H
ATOM	333	CD	GLN	21	39.518	-7.759	19.194	1.00	0.00	C
ATOM	334	OE1	GLN	21	40.297	-7.225	19.912	1.00	0.00	O
ATOM	335	NE2	GLN	21	38.744	-8.638	19.740	1.00	0.00	N
ATOM	336	HE21	GLN	21	39.020	-8.746	20.705	1.00	0.00	H
ATOM	337	HE22	GLN	21	38.189	-9.273	19.185	1.00	0.00	H
ATOM	338	C	GLN	21	36.329	-4.977	16.328	1.00	0.00	C
ATOM	339	O	GLN	21	35.316	-5.633	16.689	1.00	0.00	O
ATOM	340	N	TRP	22	36.243	-3.695	15.989	1.00	0.00	N
ATOM	341	H	TRP	22	37.086	-3.203	15.731	1.00	0.00	H
ATOM	342	CA	TRP	22	35.037	-2.915	15.965	1.00	0.00	C
ATOM	343	HA	TRP	22	34.511	-3.311	16.834	1.00	0.00	H
ATOM	344	CB	TRP	22	35.376	-1.395	16.243	1.00	0.00	C
ATOM	345	HB2	TRP	22	34.436	-0.932	16.543	1.00	0.00	H
ATOM	346	HB3	TRP	22	35.828	-1.037	15.318	1.00	0.00	H
ATOM	347	CG	TRP	22	36.283	-0.989	17.452	1.00	0.00	C
ATOM	348	CD1	TRP	22	36.391	-1.707	18.594	1.00	0.00	C
ATOM	349	HD1	TRP	22	35.941	-2.675	18.756	1.00	0.00	H
ATOM	350	NE1	TRP	22	37.141	-0.960	19.483	1.00	0.00	N
ATOM	351	HE1	TRP	22	37.286	-1.132	20.467	1.00	0.00	H
ATOM	352	CE2	TRP	22	37.787	0.103	18.780	1.00	0.00	C
ATOM	353	CZ2	TRP	22	38.712	1.077	19.203	1.00	0.00	C
ATOM	354	HZ2	TRP	22	38.983	1.030	20.247	1.00	0.00	H
ATOM	355	CH2	TRP	22	39.118	2.034	18.277	1.00	0.00	C

ATOM	356	HH2	TRP	22	39.906	2.711	18.574	1.00	0.00	H
ATOM	357	CZ3	TRP	22	38.579	2.105	16.930	1.00	0.00	C
ATOM	358	HZ3	TRP	22	38.806	2.906	16.243	1.00	0.00	H
ATOM	359	CE3	TRP	22	37.636	1.083	16.536	1.00	0.00	C
ATOM	360	HE3	TRP	22	37.234	1.069	15.534	1.00	0.00	H
ATOM	361	CD2	TRP	22	37.260	0.065	17.489	1.00	0.00	C
ATOM	362	C	TRP	22	34.032	-3.209	14.846	1.00	0.00	C
ATOM	363	O	TRP	22	34.403	-3.394	13.673	1.00	0.00	O
ATOM	364	N	ASN	23	32.735	-3.347	15.206	1.00	0.00	N
ATOM	365	H	ASN	23	32.510	-3.184	16.177	1.00	0.00	H
ATOM	366	CA	ASN	23	31.722	-3.847	14.230	1.00	0.00	C
ATOM	367	HA	ASN	23	32.130	-4.669	13.643	1.00	0.00	H
ATOM	368	CB	ASN	23	30.515	-4.430	14.977	1.00	0.00	C
ATOM	369	HB2	ASN	23	29.946	-3.656	15.493	1.00	0.00	H
ATOM	370	HB3	ASN	23	31.048	-5.012	15.729	1.00	0.00	H
ATOM	371	CG	ASN	23	29.599	-5.383	14.276	1.00	0.00	C
ATOM	372	OD1	ASN	23	29.728	-5.653	13.122	1.00	0.00	O
ATOM	373	ND2	ASN	23	28.468	-5.765	14.889	1.00	0.00	N
ATOM	374	HD21	ASN	23	27.791	-6.383	14.464	1.00	0.00	H
ATOM	375	HD22	ASN	23	28.308	-5.278	15.759	1.00	0.00	H
ATOM	376	C	ASN	23	31.299	-2.703	13.253	1.00	0.00	C
ATOM	377	O	ASN	23	31.631	-1.509	13.396	1.00	0.00	O
ATOM	378	N	LYS	24	30.342	-2.993	12.395	1.00	0.00	N
ATOM	379	H	LYS	24	30.133	-3.980	12.355	1.00	0.00	H
ATOM	380	CA	LYS	24	29.570	-2.010	11.542	1.00	0.00	C
ATOM	381	HA	LYS	24	30.287	-1.402	10.989	1.00	0.00	H
ATOM	382	CB	LYS	24	28.718	-2.794	10.490	1.00	0.00	C
ATOM	383	HB2	LYS	24	29.299	-3.514	9.914	1.00	0.00	H
ATOM	384	HB3	LYS	24	28.263	-1.957	9.959	1.00	0.00	H
ATOM	385	CG	LYS	24	27.587	-3.615	11.097	1.00	0.00	C
ATOM	386	HG2	LYS	24	27.100	-3.207	11.983	1.00	0.00	H
ATOM	387	HG3	LYS	24	28.101	-4.524	11.407	1.00	0.00	H
ATOM	388	CD	LYS	24	26.496	-3.940	10.108	1.00	0.00	C
ATOM	389	HD2	LYS	24	26.827	-4.686	9.386	1.00	0.00	H
ATOM	390	HD3	LYS	24	26.378	-2.987	9.592	1.00	0.00	H
ATOM	391	CE	LYS	24	25.186	-4.418	10.649	1.00	0.00	C
ATOM	392	HE2	LYS	24	24.742	-3.694	11.333	1.00	0.00	H
ATOM	393	HE3	LYS	24	25.517	-5.259	11.257	1.00	0.00	H
ATOM	394	NZ	LYS	24	24.237	-4.817	9.562	1.00	0.00	N
ATOM	395	HZ1	LYS	24	24.623	-5.583	9.028	1.00	0.00	H
ATOM	396	HZ2	LYS	24	23.396	-5.048	10.070	1.00	0.00	H
ATOM	397	HZ3	LYS	24	24.069	-4.012	8.974	1.00	0.00	H
ATOM	398	C	LYS	24	28.797	-1.063	12.391	1.00	0.00	C
ATOM	399	O	LYS	24	28.234	-1.434	13.407	1.00	0.00	O
ATOM	400	N	PRO	25	28.789	0.244	12.097	1.00	0.00	N
ATOM	401	CD	PRO	25	29.370	0.874	10.887	1.00	0.00	C
ATOM	402	HD2	PRO	25	30.460	0.910	10.902	1.00	0.00	H
ATOM	403	HD3	PRO	25	28.836	0.376	10.078	1.00	0.00	H
ATOM	404	CG	PRO	25	28.994	2.358	10.909	1.00	0.00	C
ATOM	405	HG2	PRO	25	29.831	2.952	10.544	1.00	0.00	H
ATOM	406	HG3	PRO	25	28.021	2.400	10.419	1.00	0.00	H
ATOM	407	CB	PRO	25	28.683	2.508	12.374	1.00	0.00	C
ATOM	408	HB2	PRO	25	29.601	2.805	12.880	1.00	0.00	H
ATOM	409	HB3	PRO	25	27.943	3.309	12.361	1.00	0.00	H
ATOM	410	CA	PRO	25	28.095	1.261	12.920	1.00	0.00	C
ATOM	411	HA	PRO	25	28.316	1.197	13.986	1.00	0.00	H
ATOM	412	C	PRO	25	26.568	1.059	12.761	1.00	0.00	C
ATOM	413	O	PRO	25	26.072	1.042	11.593	1.00	0.00	O
ATOM	414	N	SER	26	25.798	0.854	13.826	1.00	0.00	N
ATOM	415	H	SER	26	26.251	1.001	14.717	1.00	0.00	H
ATOM	416	CA	SER	26	24.346	0.511	13.882	1.00	0.00	C
ATOM	417	HA	SER	26	24.185	-0.374	13.265	1.00	0.00	H
ATOM	418	CB	SER	26	23.972	0.106	15.302	1.00	0.00	C

ATOM	419	HB2	SER	26	23.064	-0.491	15.224	1.00	0.00	H
ATOM	420	HB3	SER	26	23.682	1.080	15.694	1.00	0.00	H
ATOM	421	OG	SER	26	25.034	-0.417	16.080	1.00	0.00	O
ATOM	422	HG	SER	26	24.654	-1.021	16.723	1.00	0.00	H
ATOM	423	C	SER	26	23.346	1.532	13.347	1.00	0.00	C
ATOM	424	O	SER	26	22.297	1.194	12.830	1.00	0.00	O
ATOM	425	N	LYS	27	23.798	2.775	13.522	1.00	0.00	N
ATOM	426	H	LYS	27	24.675	2.788	14.022	1.00	0.00	H
ATOM	427	CA	LYS	27	23.223	4.031	13.076	1.00	0.00	C
ATOM	428	HA	LYS	27	22.333	3.860	12.470	1.00	0.00	H
ATOM	429	CB	LYS	27	22.876	4.788	14.338	1.00	0.00	C
ATOM	430	HB2	LYS	27	22.766	5.869	14.252	1.00	0.00	H
ATOM	431	HB3	LYS	27	23.795	4.615	14.899	1.00	0.00	H
ATOM	432	CG	LYS	27	21.633	4.251	15.090	1.00	0.00	C
ATOM	433	HG2	LYS	27	21.634	4.611	16.119	1.00	0.00	H
ATOM	434	HG3	LYS	27	21.738	3.177	15.248	1.00	0.00	H
ATOM	435	CD	LYS	27	20.207	4.629	14.505	1.00	0.00	C
ATOM	436	HD2	LYS	27	20.031	4.165	13.534	1.00	0.00	H
ATOM	437	HD3	LYS	27	20.264	5.717	14.472	1.00	0.00	H
ATOM	438	CE	LYS	27	19.178	4.173	15.503	1.00	0.00	C
ATOM	439	HE2	LYS	27	19.526	4.272	16.531	1.00	0.00	H
ATOM	440	HE3	LYS	27	19.060	3.101	15.348	1.00	0.00	H
ATOM	441	NZ	LYS	27	17.817	4.732	15.172	1.00	0.00	N
ATOM	442	HZ1	LYS	27	17.654	4.575	14.188	1.00	0.00	H
ATOM	443	HZ2	LYS	27	17.232	4.203	15.803	1.00	0.00	H
ATOM	444	HZ3	LYS	27	17.758	5.705	15.438	1.00	0.00	H
ATOM	445	C	LYS	27	24.326	4.822	12.298	1.00	0.00	C
ATOM	446	O	LYS	27	25.534	4.644	12.501	1.00	0.00	O
ATOM	447	N	PRO	28	23.991	5.722	11.391	1.00	0.00	N
ATOM	448	CD	PRO	28	22.636	5.751	10.793	1.00	0.00	C
ATOM	449	HD2	PRO	28	22.126	4.808	10.592	1.00	0.00	H
ATOM	450	HD3	PRO	28	22.043	6.399	11.438	1.00	0.00	H
ATOM	451	CG	PRO	28	22.900	6.627	9.535	1.00	0.00	C
ATOM	452	HG2	PRO	28	23.225	5.932	8.761	1.00	0.00	H
ATOM	453	HG3	PRO	28	22.124	7.347	9.277	1.00	0.00	H
ATOM	454	CB	PRO	28	24.060	7.526	9.841	1.00	0.00	C
ATOM	455	HB2	PRO	28	24.453	7.912	8.901	1.00	0.00	H
ATOM	456	HB3	PRO	28	23.735	8.291	10.546	1.00	0.00	H
ATOM	457	CA	PRO	28	24.914	6.625	10.685	1.00	0.00	C
ATOM	458	HA	PRO	28	25.597	6.008	10.101	1.00	0.00	H
ATOM	459	C	PRO	28	25.871	7.567	11.540	1.00	0.00	C
ATOM	460	O	PRO	28	26.966	7.937	11.113	1.00	0.00	O
ATOM	461	N	LYS	29	25.551	7.940	12.761	1.00	0.00	N
ATOM	462	H	LYS	29	24.685	7.550	13.105	1.00	0.00	H
ATOM	463	CA	LYS	29	26.267	8.762	13.706	1.00	0.00	C
ATOM	464	HA	LYS	29	27.303	8.904	13.395	1.00	0.00	H
ATOM	465	CB	LYS	29	25.412	10.049	13.835	1.00	0.00	C
ATOM	466	HB2	LYS	29	24.395	9.871	14.184	1.00	0.00	H
ATOM	467	HB3	LYS	29	25.398	10.442	12.818	1.00	0.00	H
ATOM	468	CG	LYS	29	25.998	11.139	14.713	1.00	0.00	C
ATOM	469	HG2	LYS	29	27.018	11.399	14.429	1.00	0.00	H
ATOM	470	HG3	LYS	29	25.928	10.777	15.738	1.00	0.00	H
ATOM	471	CD	LYS	29	25.126	12.396	14.608	1.00	0.00	C
ATOM	472	HD2	LYS	29	24.062	12.160	14.592	1.00	0.00	H
ATOM	473	HD3	LYS	29	25.409	12.818	13.644	1.00	0.00	H
ATOM	474	CE	LYS	29	25.247	13.455	15.674	1.00	0.00	C
ATOM	475	HE2	LYS	29	26.293	13.677	15.888	1.00	0.00	H
ATOM	476	HE3	LYS	29	24.762	12.960	16.516	1.00	0.00	H
ATOM	477	NZ	LYS	29	24.458	14.651	15.398	1.00	0.00	N
ATOM	478	HZ1	LYS	29	24.730	15.150	14.563	1.00	0.00	H
ATOM	479	HZ2	LYS	29	23.493	14.450	15.178	1.00	0.00	H
ATOM	480	HZ3	LYS	29	24.528	15.330	16.142	1.00	0.00	H
ATOM	481	C	LYS	29	26.509	8.040	15.094	1.00	0.00	C

ATOM	482	O	LYS	29	25.639	7.265	15.506	1.00	0.00	O
ATOM	483	N	THR	30	27.552	8.335	15.858	1.00	0.00	N
ATOM	484	H	THR	30	28.236	9.011	15.550	1.00	0.00	H
ATOM	485	CA	THR	30	27.901	7.513	17.043	1.00	0.00	C
ATOM	486	HA	THR	30	27.565	6.482	16.932	1.00	0.00	H
ATOM	487	CB	THR	30	29.454	7.424	17.125	1.00	0.00	C
ATOM	488	HB	THR	30	29.693	6.872	18.034	1.00	0.00	H
ATOM	489	CG2	THR	30	30.130	6.785	15.985	1.00	0.00	C
ATOM	490	HG21	THR	30	29.937	7.358	15.078	1.00	0.00	H
ATOM	491	HG22	THR	30	31.170	6.582	16.239	1.00	0.00	H
ATOM	492	HG23	THR	30	29.642	5.814	15.907	1.00	0.00	H
ATOM	493	OG1	THR	30	29.940	8.721	17.317	1.00	0.00	O
ATOM	494	HG1	THR	30	30.784	8.745	16.860	1.00	0.00	H
ATOM	495	C	THR	30	27.118	7.966	18.370	1.00	0.00	C
ATOM	496	O	THR	30	26.954	7.213	19.308	1.00	0.00	O
ATOM	497	N	ASN	31	26.520	9.135	18.387	1.00	0.00	N
ATOM	498	H	ASN	31	26.782	9.747	17.628	1.00	0.00	H
ATOM	499	CA	ASN	31	25.934	9.769	19.654	1.00	0.00	C
ATOM	500	HA	ASN	31	26.209	9.201	20.542	1.00	0.00	H
ATOM	501	CB	ASN	31	26.541	11.231	19.745	1.00	0.00	C
ATOM	502	HB2	ASN	31	26.111	11.885	18.986	1.00	0.00	H
ATOM	503	HB3	ASN	31	27.612	11.068	19.624	1.00	0.00	H
ATOM	504	CG	ASN	31	26.514	11.779	21.137	1.00	0.00	C
ATOM	505	OD1	ASN	31	25.841	12.718	21.531	1.00	0.00	O
ATOM	506	ND2	ASN	31	27.197	11.217	22.063	1.00	0.00	N
ATOM	507	HD21	ASN	31	27.183	11.620	22.989	1.00	0.00	H
ATOM	508	HD22	ASN	31	27.755	10.414	21.811	1.00	0.00	H
ATOM	509	C	ASN	31	24.408	9.684	19.652	1.00	0.00	C
ATOM	510	O	ASN	31	23.652	10.676	19.931	1.00	0.00	O
ATOM	511	N	MET	32	23.893	8.501	19.299	1.00	0.00	N
ATOM	512	H	MET	32	24.579	7.795	19.072	1.00	0.00	H
ATOM	513	CA	MET	32	22.480	8.169	19.021	1.00	0.00	C
ATOM	514	HA	MET	32	21.873	8.962	19.457	1.00	0.00	H
ATOM	515	CB	MET	32	22.210	8.070	17.510	1.00	0.00	C
ATOM	516	HB2	MET	32	21.226	7.665	17.275	1.00	0.00	H
ATOM	517	HB3	MET	32	23.058	7.491	17.145	1.00	0.00	H
ATOM	518	CG	MET	32	22.281	9.355	16.771	1.00	0.00	C
ATOM	519	HG2	MET	32	22.600	9.198	15.740	1.00	0.00	H
ATOM	520	HG3	MET	32	23.085	9.872	17.294	1.00	0.00	H
ATOM	521	SD	MET	32	20.756	10.305	16.828	1.00	0.00	S
ATOM	522	CE	MET	32	19.507	9.151	16.198	1.00	0.00	C
ATOM	523	HE1	MET	32	18.634	9.616	15.739	1.00	0.00	H
ATOM	524	HE2	MET	32	19.970	8.550	15.415	1.00	0.00	H
ATOM	525	HE3	MET	32	19.145	8.438	16.938	1.00	0.00	H
ATOM	526	C	MET	32	22.165	6.848	19.740	1.00	0.00	C
ATOM	527	O	MET	32	23.049	6.001	19.855	1.00	0.00	O
ATOM	528	N	LYS	33	20.867	6.598	20.028	1.00	0.00	N
ATOM	529	H	LYS	33	20.190	7.319	19.829	1.00	0.00	H
ATOM	530	CA	LYS	33	20.283	5.430	20.782	1.00	0.00	C
ATOM	531	HA	LYS	33	21.094	5.186	21.468	1.00	0.00	H
ATOM	532	CB	LYS	33	19.002	6.027	21.405	1.00	0.00	C
ATOM	533	HB2	LYS	33	18.091	5.880	20.825	1.00	0.00	H
ATOM	534	HB3	LYS	33	19.142	7.104	21.496	1.00	0.00	H
ATOM	535	CG	LYS	33	18.720	5.313	22.724	1.00	0.00	C
ATOM	536	HG2	LYS	33	18.842	4.233	22.636	1.00	0.00	H
ATOM	537	HG3	LYS	33	17.668	5.509	22.930	1.00	0.00	H
ATOM	538	CD	LYS	33	19.519	5.893	23.907	1.00	0.00	C
ATOM	539	HD2	LYS	33	19.603	6.980	23.937	1.00	0.00	H
ATOM	540	HD3	LYS	33	20.508	5.442	23.823	1.00	0.00	H
ATOM	541	CE	LYS	33	19.140	5.513	25.317	1.00	0.00	C
ATOM	542	HE2	LYS	33	18.915	4.452	25.432	1.00	0.00	H
ATOM	543	HE3	LYS	33	18.205	6.069	25.266	1.00	0.00	H
ATOM	544	NZ	LYS	33	20.086	5.927	26.400	1.00	0.00	N

```
ATOM    545  HZ1 LYS    33      20.234   6.923  26.325  1.00  0.00           H
ATOM    546  HZ2 LYS    33      19.742   5.714  27.326  1.00  0.00           H
ATOM    547  HZ3 LYS    33      20.981   5.478  26.268  1.00  0.00           H
ATOM    548  C   LYS    33      20.187   4.241  19.828  1.00  0.00           C
ATOM    549  O   LYS    33      19.971   4.366  18.624  1.00  0.00           O
ATOM    550  N   NME    34      20.412   3.098  20.403  1.00  0.00           N
ATOM    551  H   NME    34      20.454   3.124  21.412  1.00  0.00           H
ATOM    552  CH3 NME    34      20.095   1.833  19.779  1.00  0.00           C
ATOM    553 HH31 NME    34      19.015   1.842  19.634  1.00  0.00           H
ATOM    554 HH32 NME    34      20.600   1.599  18.842  1.00  0.00           H
ATOM    555 HH33 NME    34      20.319   1.022  20.472  1.00  0.00           H
TER     556      NME    34
END
```

Cluster 4:

```
ATOM      1 HH31 ACE     1      19.704  19.083   9.497  1.00  0.00           H
ATOM      2  CH3 ACE     1      20.515  18.740   8.855  1.00  0.00           C
ATOM      3 HH32 ACE     1      21.437  18.701   9.434  1.00  0.00           H
ATOM      4 HH33 ACE     1      20.730  19.324   7.959  1.00  0.00           H
ATOM      5  C   ACE     1      20.071  17.363   8.358  1.00  0.00           C
ATOM      6  O   ACE     1      20.193  16.453   9.171  1.00  0.00           O
ATOM      7  N   ASN     2      19.579  17.260   7.108  1.00  0.00           N
ATOM      8  H   ASN     2      19.510  18.120   6.580  1.00  0.00           H
ATOM      9  CA  ASN     2      19.187  16.026   6.378  1.00  0.00           C
ATOM     10  HA  ASN     2      18.580  15.457   7.082  1.00  0.00           H
ATOM     11  CB  ASN     2      18.513  16.434   5.117  1.00  0.00           C
ATOM     12  HB2 ASN     2      19.309  16.819   4.481  1.00  0.00           H
ATOM     13  HB3 ASN     2      17.808  17.233   5.346  1.00  0.00           H
ATOM     14  CG  ASN     2      17.697  15.366   4.338  1.00  0.00           C
ATOM     15  OD1 ASN     2      18.100  14.729   3.337  1.00  0.00           O
ATOM     16  ND2 ASN     2      16.415  15.135   4.715  1.00  0.00           N
ATOM     17 HD21 ASN     2      15.910  14.561   4.056  1.00  0.00           H
ATOM     18 HD22 ASN     2      15.895  15.570   5.463  1.00  0.00           H
ATOM     19  C   ASN     2      20.445  15.244   5.983  1.00  0.00           C
ATOM     20  O   ASN     2      21.532  15.768   5.652  1.00  0.00           O
ATOM     21  N   ASP     3      20.329  13.910   5.943  1.00  0.00           N
ATOM     22  H   ASP     3      19.446  13.603   6.325  1.00  0.00           H
ATOM     23  CA  ASP     3      21.430  13.050   5.576  1.00  0.00           C
ATOM     24  HA  ASP     3      22.021  13.546   4.806  1.00  0.00           H
ATOM     25  CB  ASP     3      22.281  12.711   6.796  1.00  0.00           C
ATOM     26  HB2 ASP     3      21.715  12.143   7.534  1.00  0.00           H
ATOM     27  HB3 ASP     3      22.656  13.665   7.164  1.00  0.00           H
ATOM     28  CG  ASP     3      23.485  11.782   6.460  1.00  0.00           C
ATOM     29  OD1 ASP     3      24.720  12.204   6.511  1.00  0.00           O
ATOM     30  OD2 ASP     3      23.224  10.594   6.132  1.00  0.00           O
ATOM     31  C   ASP     3      20.878  11.901   4.796  1.00  0.00           C
ATOM     32  O   ASP     3      20.365  10.946   5.389  1.00  0.00           O
ATOM     33  N   TYR     4      20.963  11.946   3.440  1.00  0.00           N
ATOM     34  H   TYR     4      21.355  12.738   2.951  1.00  0.00           H
ATOM     35  CA  TYR     4      20.728  10.818   2.578  1.00  0.00           C
ATOM     36  HA  TYR     4      20.850   9.901   3.155  1.00  0.00           H
ATOM     37  CB  TYR     4      19.299  10.949   2.023  1.00  0.00           C
ATOM     38  HB2 TYR     4      19.177  11.889   1.485  1.00  0.00           H
ATOM     39  HB3 TYR     4      18.711  11.055   2.934  1.00  0.00           H
ATOM     40  CG  TYR     4      18.798   9.810   1.084  1.00  0.00           C
ATOM     41  CD1 TYR     4      18.416   8.585   1.657  1.00  0.00           C
ATOM     42  HD1 TYR     4      18.476   8.290   2.693  1.00  0.00           H
ATOM     43  CE1 TYR     4      17.944   7.618   0.768  1.00  0.00           C
ATOM     44  HE1 TYR     4      17.823   6.600   1.106  1.00  0.00           H
ATOM     45  CZ  TYR     4      17.872   7.845  -0.630  1.00  0.00           C
```

ATOM	46	OH	TYR	4	17.513	6.728	-1.344	1.00	0.00	O
ATOM	47	HH	TYR	4	17.524	6.993	-2.267	1.00	0.00	H
ATOM	48	CE2	TYR	4	18.088	9.136	-1.183	1.00	0.00	C
ATOM	49	HE2	TYR	4	17.858	9.300	-2.225	1.00	0.00	H
ATOM	50	CD2	TYR	4	18.428	10.103	-0.244	1.00	0.00	C
ATOM	51	HD2	TYR	4	18.666	11.069	-0.666	1.00	0.00	H
ATOM	52	C	TYR	4	21.712	10.701	1.364	1.00	0.00	C
ATOM	53	O	TYR	4	22.345	11.703	0.963	1.00	0.00	O
ATOM	54	N	GLU	5	21.921	9.447	0.798	1.00	0.00	N
ATOM	55	H	GLU	5	21.562	8.602	1.221	1.00	0.00	H
ATOM	56	CA	GLU	5	22.663	9.315	-0.485	1.00	0.00	C
ATOM	57	HA	GLU	5	22.810	10.300	-0.928	1.00	0.00	H
ATOM	58	CB	GLU	5	24.179	8.915	-0.138	1.00	0.00	C
ATOM	59	HB2	GLU	5	24.283	8.083	0.559	1.00	0.00	H
ATOM	60	HB3	GLU	5	24.527	9.819	0.360	1.00	0.00	H
ATOM	61	CG	GLU	5	25.045	8.579	-1.368	1.00	0.00	C
ATOM	62	HG2	GLU	5	24.518	7.986	-2.116	1.00	0.00	H
ATOM	63	HG3	GLU	5	25.870	7.955	-1.025	1.00	0.00	H
ATOM	64	CD	GLU	5	25.630	9.804	-2.089	1.00	0.00	C
ATOM	65	OE1	GLU	5	24.820	10.624	-2.575	1.00	0.00	O
ATOM	66	OE2	GLU	5	26.870	9.770	-2.368	1.00	0.00	O
ATOM	67	C	GLU	5	22.023	8.279	-1.420	1.00	0.00	C
ATOM	68	O	GLU	5	21.940	7.060	-1.193	1.00	0.00	O
ATOM	69	N	ASP	6	21.409	8.744	-2.524	1.00	0.00	N
ATOM	70	H	ASP	6	21.374	9.722	-2.775	1.00	0.00	H
ATOM	71	CA	ASP	6	20.579	7.821	-3.310	1.00	0.00	C
ATOM	72	HA	ASP	6	19.963	7.194	-2.667	1.00	0.00	H
ATOM	73	CB	ASP	6	19.770	8.562	-4.403	1.00	0.00	C
ATOM	74	HB2	ASP	6	20.369	8.608	-5.311	1.00	0.00	H
ATOM	75	HB3	ASP	6	19.641	9.575	-4.022	1.00	0.00	H
ATOM	76	CG	ASP	6	18.341	7.933	-4.750	1.00	0.00	C
ATOM	77	OD1	ASP	6	17.844	7.223	-3.862	1.00	0.00	O
ATOM	78	OD2	ASP	6	17.700	8.413	-5.760	1.00	0.00	O
ATOM	79	C	ASP	6	21.397	6.771	-4.054	1.00	0.00	C
ATOM	80	O	ASP	6	21.041	5.578	-4.082	1.00	0.00	O
ATOM	81	N	ARG	7	22.670	7.019	-4.318	1.00	0.00	N
ATOM	82	H	ARG	7	23.030	7.944	-4.131	1.00	0.00	H
ATOM	83	CA	ARG	7	23.536	6.053	-5.030	1.00	0.00	C
ATOM	84	HA	ARG	7	23.128	5.737	-5.990	1.00	0.00	H
ATOM	85	CB	ARG	7	24.839	6.792	-5.390	1.00	0.00	C
ATOM	86	HB2	ARG	7	25.505	6.161	-5.978	1.00	0.00	H
ATOM	87	HB3	ARG	7	25.302	7.036	-4.433	1.00	0.00	H
ATOM	88	CG	ARG	7	24.771	8.167	-6.172	1.00	0.00	C
ATOM	89	HG2	ARG	7	24.376	8.925	-5.496	1.00	0.00	H
ATOM	90	HG3	ARG	7	24.243	7.982	-7.109	1.00	0.00	H
ATOM	91	CD	ARG	7	26.158	8.672	-6.506	1.00	0.00	C
ATOM	92	HD2	ARG	7	26.026	9.473	-7.235	1.00	0.00	H
ATOM	93	HD3	ARG	7	26.630	7.870	-7.074	1.00	0.00	H
ATOM	94	NE	ARG	7	27.038	9.047	-5.382	1.00	0.00	N
ATOM	95	HE	ARG	7	26.612	9.123	-4.470	1.00	0.00	H
ATOM	96	CZ	ARG	7	28.254	9.601	-5.452	1.00	0.00	C
ATOM	97	NH1	ARG	7	28.841	9.682	-6.624	1.00	0.00	N
ATOM	98	HH11	ARG	7	28.307	9.318	-7.400	1.00	0.00	H
ATOM	99	HH12	ARG	7	29.753	10.106	-6.717	1.00	0.00	H
ATOM	100	NH2	ARG	7	28.901	10.030	-4.387	1.00	0.00	N
ATOM	101	HH21	ARG	7	28.344	9.960	-3.548	1.00	0.00	H
ATOM	102	HH22	ARG	7	29.819	10.427	-4.253	1.00	0.00	H
ATOM	103	C	ARG	7	23.881	4.798	-4.124	1.00	0.00	C
ATOM	104	O	ARG	7	23.930	4.870	-2.881	1.00	0.00	O
ATOM	105	N	TYR	8	24.022	3.640	-4.817	1.00	0.00	N
ATOM	106	H	TYR	8	23.890	3.750	-5.812	1.00	0.00	H
ATOM	107	CA	TYR	8	24.378	2.371	-4.190	1.00	0.00	C
ATOM	108	HA	TYR	8	23.602	2.125	-3.465	1.00	0.00	H

ATOM	109	CB	TYR	8	24.308	1.335	-5.375	1.00	0.00	C
ATOM	110	HB2	TYR	8	25.073	1.521	-6.128	1.00	0.00	H
ATOM	111	HB3	TYR	8	23.334	1.511	-5.829	1.00	0.00	H
ATOM	112	CG	TYR	8	24.234	-0.161	-5.024	1.00	0.00	C
ATOM	113	CD1	TYR	8	25.061	-1.103	-5.677	1.00	0.00	C
ATOM	114	HD1	TYR	8	25.722	-0.630	-6.389	1.00	0.00	H
ATOM	115	CE1	TYR	8	25.144	-2.426	-5.260	1.00	0.00	C
ATOM	116	HE1	TYR	8	25.810	-3.099	-5.779	1.00	0.00	H
ATOM	117	CZ	TYR	8	24.261	-2.876	-4.219	1.00	0.00	C
ATOM	118	OH	TYR	8	24.187	-4.177	-3.887	1.00	0.00	O
ATOM	119	HH	TYR	8	23.500	-4.313	-3.230	1.00	0.00	H
ATOM	120	CE2	TYR	8	23.335	-1.943	-3.685	1.00	0.00	C
ATOM	121	HE2	TYR	8	22.611	-2.270	-2.953	1.00	0.00	H
ATOM	122	CD2	TYR	8	23.287	-0.570	-4.039	1.00	0.00	C
ATOM	123	HD2	TYR	8	22.612	0.119	-3.553	1.00	0.00	H
ATOM	124	C	TYR	8	25.734	2.350	-3.558	1.00	0.00	C
ATOM	125	O	TYR	8	25.886	1.937	-2.392	1.00	0.00	O
ATOM	126	N	TYR	9	26.663	3.136	-4.222	1.00	0.00	N
ATOM	127	H	TYR	9	26.453	3.452	-5.158	1.00	0.00	H
ATOM	128	CA	TYR	9	28.076	3.265	-3.741	1.00	0.00	C
ATOM	129	HA	TYR	9	28.332	2.368	-3.178	1.00	0.00	H
ATOM	130	CB	TYR	9	29.022	3.372	-4.919	1.00	0.00	C
ATOM	131	HB2	TYR	9	30.016	3.289	-4.479	1.00	0.00	H
ATOM	132	HB3	TYR	9	28.891	4.349	-5.383	1.00	0.00	H
ATOM	133	CG	TYR	9	28.954	2.311	-5.994	1.00	0.00	C
ATOM	134	CD1	TYR	9	28.952	2.751	-7.365	1.00	0.00	C
ATOM	135	HD1	TYR	9	29.001	3.777	-7.698	1.00	0.00	H
ATOM	136	CE1	TYR	9	28.901	1.745	-8.366	1.00	0.00	C
ATOM	137	HE1	TYR	9	28.967	2.037	-9.404	1.00	0.00	H
ATOM	138	CZ	TYR	9	28.646	0.402	-8.131	1.00	0.00	C
ATOM	139	OH	TYR	9	28.636	-0.472	-9.112	1.00	0.00	O
ATOM	140	HH	TYR	9	28.466	-1.395	-8.912	1.00	0.00	H
ATOM	141	CE2	TYR	9	28.600	-0.004	-6.773	1.00	0.00	C
ATOM	142	HE2	TYR	9	28.445	-1.051	-6.556	1.00	0.00	H
ATOM	143	CD2	TYR	9	28.675	0.946	-5.681	1.00	0.00	C
ATOM	144	HD2	TYR	9	28.616	0.700	-4.631	1.00	0.00	H
ATOM	145	C	TYR	9	28.163	4.418	-2.735	1.00	0.00	C
ATOM	146	O	TYR	9	27.269	5.227	-2.530	1.00	0.00	O
ATOM	147	N	ARG	10	29.395	4.587	-2.202	1.00	0.00	N
ATOM	148	H	ARG	10	29.998	3.777	-2.254	1.00	0.00	H
ATOM	149	CA	ARG	10	29.895	5.643	-1.246	1.00	0.00	C
ATOM	150	HA	ARG	10	29.251	6.520	-1.311	1.00	0.00	H
ATOM	151	CB	ARG	10	29.752	4.927	0.108	1.00	0.00	C
ATOM	152	HB2	ARG	10	30.274	5.492	0.881	1.00	0.00	H
ATOM	153	HB3	ARG	10	30.223	3.966	-0.093	1.00	0.00	H
ATOM	154	CG	ARG	10	28.391	4.715	0.794	1.00	0.00	C
ATOM	155	HG2	ARG	10	28.510	4.391	1.828	1.00	0.00	H
ATOM	156	HG3	ARG	10	27.874	3.931	0.241	1.00	0.00	H
ATOM	157	CD	ARG	10	27.616	6.078	0.868	1.00	0.00	C
ATOM	158	HD2	ARG	10	27.591	6.687	-0.035	1.00	0.00	H
ATOM	159	HD3	ARG	10	28.225	6.624	1.589	1.00	0.00	H
ATOM	160	NE	ARG	10	26.264	5.878	1.449	1.00	0.00	N
ATOM	161	HE	ARG	10	26.067	6.060	2.423	1.00	0.00	H
ATOM	162	CZ	ARG	10	25.162	5.584	0.726	1.00	0.00	C
ATOM	163	NH1	ARG	10	25.245	5.315	-0.548	1.00	0.00	N
ATOM	164	HH11	ARG	10	26.136	5.241	-1.018	1.00	0.00	H
ATOM	165	HH12	ARG	10	24.432	5.191	-1.132	1.00	0.00	H
ATOM	166	NH2	ARG	10	23.974	5.460	1.347	1.00	0.00	N
ATOM	167	HH21	ARG	10	23.826	5.572	2.341	1.00	0.00	H
ATOM	168	HH22	ARG	10	23.231	5.031	0.815	1.00	0.00	H
ATOM	169	C	ARG	10	31.317	6.062	-1.573	1.00	0.00	C
ATOM	170	O	ARG	10	31.947	5.427	-2.418	1.00	0.00	O
ATOM	171	N	GLU	11	31.731	7.149	-0.983	1.00	0.00	N

ATOM	172	H	GLU	11	31.141	7.630	-0.319	1.00	0.00	H
ATOM	173	CA	GLU	11	33.125	7.707	-0.927	1.00	0.00	C
ATOM	174	HA	GLU	11	33.493	7.734	-1.952	1.00	0.00	H
ATOM	175	CB	GLU	11	33.138	9.137	-0.429	1.00	0.00	C
ATOM	176	HB2	GLU	11	34.156	9.391	-0.722	1.00	0.00	H
ATOM	177	HB3	GLU	11	32.901	9.094	0.634	1.00	0.00	H
ATOM	178	CG	GLU	11	32.172	10.189	-1.085	1.00	0.00	C
ATOM	179	HG2	GLU	11	32.154	11.096	-0.481	1.00	0.00	H
ATOM	180	HG3	GLU	11	31.205	9.685	-1.044	1.00	0.00	H
ATOM	181	CD	GLU	11	32.449	10.574	-2.486	1.00	0.00	C
ATOM	182	OE1	GLU	11	33.559	11.119	-2.795	1.00	0.00	O
ATOM	183	OE2	GLU	11	31.509	10.503	-3.334	1.00	0.00	O
ATOM	184	C	GLU	11	33.961	6.673	-0.206	1.00	0.00	C
ATOM	185	O	GLU	11	33.652	6.311	0.892	1.00	0.00	O
ATOM	186	N	ASN	12	35.193	6.428	-0.701	1.00	0.00	N
ATOM	187	H	ASN	12	35.581	6.763	-1.571	1.00	0.00	H
ATOM	188	CA	ASN	12	36.137	5.494	-0.055	1.00	0.00	C
ATOM	189	HA	ASN	12	35.485	4.750	0.402	1.00	0.00	H
ATOM	190	CB	ASN	12	36.999	4.720	-1.073	1.00	0.00	C
ATOM	191	HB2	ASN	12	37.682	5.464	-1.482	1.00	0.00	H
ATOM	192	HB3	ASN	12	36.270	4.398	-1.817	1.00	0.00	H
ATOM	193	CG	ASN	12	37.785	3.502	-0.547	1.00	0.00	C
ATOM	194	OD1	ASN	12	37.527	2.872	0.504	1.00	0.00	O
ATOM	195	ND2	ASN	12	38.822	3.080	-1.198	1.00	0.00	N
ATOM	196	HD21	ASN	12	39.222	2.225	-0.840	1.00	0.00	H
ATOM	197	HD22	ASN	12	38.975	3.355	-2.157	1.00	0.00	H
ATOM	198	C	ASN	12	36.986	6.097	1.113	1.00	0.00	C
ATOM	199	O	ASN	12	37.709	7.047	0.818	1.00	0.00	O
ATOM	200	N	MET	13	36.820	5.622	2.326	1.00	0.00	N
ATOM	201	H	MET	13	36.172	4.858	2.454	1.00	0.00	H
ATOM	202	CA	MET	13	37.554	6.048	3.513	1.00	0.00	C
ATOM	203	HA	MET	13	38.134	6.931	3.248	1.00	0.00	H
ATOM	204	CB	MET	13	36.590	6.458	4.674	1.00	0.00	C
ATOM	205	HB2	MET	13	37.162	6.776	5.547	1.00	0.00	H
ATOM	206	HB3	MET	13	35.959	5.635	5.012	1.00	0.00	H
ATOM	207	CG	MET	13	35.598	7.622	4.304	1.00	0.00	C
ATOM	208	HG2	MET	13	35.025	7.434	3.396	1.00	0.00	H
ATOM	209	HG3	MET	13	36.243	8.492	4.180	1.00	0.00	H
ATOM	210	SD	MET	13	34.258	7.964	5.501	1.00	0.00	S
ATOM	211	CE	MET	13	33.444	9.335	4.558	1.00	0.00	C
ATOM	212	HE1	MET	13	32.636	9.703	5.189	1.00	0.00	H
ATOM	213	HE2	MET	13	34.158	10.148	4.432	1.00	0.00	H
ATOM	214	HE3	MET	13	33.154	9.016	3.557	1.00	0.00	H
ATOM	215	C	MET	13	38.553	4.995	4.079	1.00	0.00	C
ATOM	216	O	MET	13	38.375	3.778	3.939	1.00	0.00	O
ATOM	217	N	TYR	14	39.612	5.412	4.818	1.00	0.00	N
ATOM	218	H	TYR	14	39.850	6.381	4.662	1.00	0.00	H
ATOM	219	CA	TYR	14	40.637	4.613	5.602	1.00	0.00	C
ATOM	220	HA	TYR	14	40.862	3.669	5.107	1.00	0.00	H
ATOM	221	CB	TYR	14	41.902	5.466	5.646	1.00	0.00	C
ATOM	222	HB2	TYR	14	41.732	6.461	6.055	1.00	0.00	H
ATOM	223	HB3	TYR	14	42.187	5.673	4.615	1.00	0.00	H
ATOM	224	CG	TYR	14	43.049	4.855	6.508	1.00	0.00	C
ATOM	225	CD1	TYR	14	43.793	3.721	6.000	1.00	0.00	C
ATOM	226	HD1	TYR	14	43.352	3.219	5.150	1.00	0.00	H
ATOM	227	CE1	TYR	14	44.830	3.084	6.753	1.00	0.00	C
ATOM	228	HE1	TYR	14	45.259	2.170	6.371	1.00	0.00	H
ATOM	229	CZ	TYR	14	45.269	3.682	7.980	1.00	0.00	C
ATOM	230	OH	TYR	14	46.358	3.226	8.728	1.00	0.00	O
ATOM	231	HH	TYR	14	46.455	3.807	9.487	1.00	0.00	H
ATOM	232	CE2	TYR	14	44.646	4.952	8.368	1.00	0.00	C
ATOM	233	HE2	TYR	14	45.075	5.456	9.221	1.00	0.00	H
ATOM	234	CD2	TYR	14	43.479	5.507	7.672	1.00	0.00	C

```
ATOM    235  HD2 TYR    14      43.136   6.500   7.921  1.00  0.00           H
ATOM    236  C   TYR    14      40.075   4.279   7.010  1.00  0.00           C
ATOM    237  O   TYR    14      39.311   5.003   7.621  1.00  0.00           O
ATOM    238  N   ARG    15      40.537   3.077   7.443  1.00  0.00           N
ATOM    239  H   ARG    15      41.230   2.650   6.846  1.00  0.00           H
ATOM    240  CA  ARG    15      39.946   2.378   8.577  1.00  0.00           C
ATOM    241  HA  ARG    15      38.863   2.466   8.491  1.00  0.00           H
ATOM    242  CB  ARG    15      40.231   0.853   8.410  1.00  0.00           C
ATOM    243  HB2 ARG    15      39.928   0.311   9.307  1.00  0.00           H
ATOM    244  HB3 ARG    15      41.320   0.893   8.383  1.00  0.00           H
ATOM    245  CG  ARG    15      39.610   0.138   7.218  1.00  0.00           C
ATOM    246  HG2 ARG    15      39.874  -0.900   7.422  1.00  0.00           H
ATOM    247  HG3 ARG    15      40.070   0.426   6.273  1.00  0.00           H
ATOM    248  CD  ARG    15      38.071   0.312   6.989  1.00  0.00           C
ATOM    249  HD2 ARG    15      37.823   1.367   6.877  1.00  0.00           H
ATOM    250  HD3 ARG    15      37.615   0.067   7.948  1.00  0.00           H
ATOM    251  NE  ARG    15      37.466  -0.535   5.876  1.00  0.00           N
ATOM    252  HE  ARG    15      37.160  -1.455   6.160  1.00  0.00           H
ATOM    253  CZ  ARG    15      37.355  -0.235   4.597  1.00  0.00           C
ATOM    254  NH1 ARG    15      37.816   0.876   4.083  1.00  0.00           N
ATOM    255 HH11 ARG    15      38.107   1.594   4.732  1.00  0.00           H
ATOM    256 HH12 ARG    15      37.593   1.075   3.118  1.00  0.00           H
ATOM    257  NH2 ARG    15      36.802  -1.038   3.798  1.00  0.00           N
ATOM    258 HH21 ARG    15      36.306  -1.852   4.130  1.00  0.00           H
ATOM    259 HH22 ARG    15      36.663  -0.794   2.828  1.00  0.00           H
ATOM    260  C   ARG    15      40.428   2.787   9.968  1.00  0.00           C
ATOM    261  O   ARG    15      39.717   2.615  10.947  1.00  0.00           O
ATOM    262  N   TYR    16      41.613   3.551  10.021  1.00  0.00           N
ATOM    263  H   TYR    16      42.185   3.714   9.206  1.00  0.00           H
ATOM    264  CA  TYR    16      42.130   4.142  11.296  1.00  0.00           C
ATOM    265  HA  TYR    16      41.459   3.857  12.108  1.00  0.00           H
ATOM    266  CB  TYR    16      43.436   3.414  11.705  1.00  0.00           C
ATOM    267  HB2 TYR    16      43.715   3.714  12.715  1.00  0.00           H
ATOM    268  HB3 TYR    16      44.117   3.770  10.932  1.00  0.00           H
ATOM    269  CG  TYR    16      43.458   1.899  11.741  1.00  0.00           C
ATOM    270  CD1 TYR    16      43.650   1.243  10.481  1.00  0.00           C
ATOM    271  HD1 TYR    16      43.623   1.701   9.503  1.00  0.00           H
ATOM    272  CE1 TYR    16      43.557  -0.135  10.443  1.00  0.00           C
ATOM    273  HE1 TYR    16      43.615  -0.599   9.469  1.00  0.00           H
ATOM    274  CZ  TYR    16      43.514  -0.871  11.609  1.00  0.00           C
ATOM    275  OH  TYR    16      43.524  -2.247  11.548  1.00  0.00           O
ATOM    276  HH  TYR    16      43.471  -2.501  12.472  1.00  0.00           H
ATOM    277  CE2 TYR    16      43.251  -0.239  12.884  1.00  0.00           C
ATOM    278  HE2 TYR    16      43.175  -0.873  13.754  1.00  0.00           H
ATOM    279  CD2 TYR    16      43.288   1.151  12.932  1.00  0.00           C
ATOM    280  HD2 TYR    16      43.046   1.692  13.835  1.00  0.00           H
ATOM    281  C   TYR    16      42.218   5.695  11.246  1.00  0.00           C
ATOM    282  O   TYR    16      41.692   6.327  10.333  1.00  0.00           O
ATOM    283  N   NME    17      42.850   6.248  12.255  1.00  0.00           N
ATOM    284  H   NME    17      43.350   5.671  12.916  1.00  0.00           H
ATOM    285  CH3 NME    17      42.954   7.635  12.456  1.00  0.00           C
ATOM    286 HH31 NME    17      43.848   7.873  13.031  1.00  0.00           H
ATOM    287 HH32 NME    17      42.910   8.072  11.458  1.00  0.00           H
ATOM    288 HH33 NME    17      42.122   7.934  13.093  1.00  0.00           H
TER     289      NME    17
ATOM    289 HH31 ACE    18      45.682   5.244   5.136  1.00  0.00           H
ATOM    290  CH3 ACE    18      45.882   5.305   4.066  1.00  0.00           C
ATOM    291 HH32 ACE    18      45.267   4.539   3.593  1.00  0.00           H
ATOM    292 HH33 ACE    18      46.875   4.862   3.984  1.00  0.00           H
ATOM    293  C   ACE    18      45.823   6.654   3.384  1.00  0.00           C
ATOM    294  O   ACE    18      46.599   6.903   2.441  1.00  0.00           O
ATOM    295  N   HIE    19      44.848   7.463   3.770  1.00  0.00           N
ATOM    296  H   HIE    19      44.191   7.120   4.455  1.00  0.00           H
```

ATOM	297	CA	HIE	19	44.368	8.556	2.979	1.00	0.00	C
ATOM	298	HA	HIE	19	45.073	8.870	2.209	1.00	0.00	H
ATOM	299	CB	HIE	19	43.108	8.114	2.259	1.00	0.00	C
ATOM	300	HB2	HIE	19	42.321	7.878	2.975	1.00	0.00	H
ATOM	301	HB3	HIE	19	43.313	7.219	1.671	1.00	0.00	H
ATOM	302	CG	HIE	19	42.655	8.966	1.095	1.00	0.00	C
ATOM	303	ND1	HIE	19	41.348	9.439	0.892	1.00	0.00	N
ATOM	304	CE1	HIE	19	41.398	10.274	-0.148	1.00	0.00	C
ATOM	305	HE1	HIE	19	40.531	10.789	-0.536	1.00	0.00	H
ATOM	306	NE2	HIE	19	42.630	10.398	-0.587	1.00	0.00	N
ATOM	307	HE2	HIE	19	42.961	10.935	-1.376	1.00	0.00	H
ATOM	308	CD2	HIE	19	43.443	9.608	0.193	1.00	0.00	C
ATOM	309	HD2	HIE	19	44.522	9.580	0.155	1.00	0.00	H
ATOM	310	C	HIE	19	44.193	9.755	3.938	1.00	0.00	C
ATOM	311	O	HIE	19	44.372	9.643	5.188	1.00	0.00	O
ATOM	312	N	SER	20	43.703	10.875	3.364	1.00	0.00	N
ATOM	313	H	SER	20	43.655	10.821	2.357	1.00	0.00	H
ATOM	314	CA	SER	20	43.151	12.129	4.037	1.00	0.00	C
ATOM	315	HA	SER	20	43.858	12.578	4.735	1.00	0.00	H
ATOM	316	CB	SER	20	43.041	13.275	3.007	1.00	0.00	C
ATOM	317	HB2	SER	20	42.986	14.255	3.482	1.00	0.00	H
ATOM	318	HB3	SER	20	42.057	13.147	2.558	1.00	0.00	H
ATOM	319	OG	SER	20	43.978	13.437	1.984	1.00	0.00	O
ATOM	320	HG	SER	20	43.573	14.022	1.339	1.00	0.00	H
ATOM	321	C	SER	20	41.874	11.919	4.759	1.00	0.00	C
ATOM	322	O	SER	20	41.647	12.571	5.771	1.00	0.00	O
ATOM	323	N	GLN	21	40.979	11.098	4.212	1.00	0.00	N
ATOM	324	H	GLN	21	41.244	10.634	3.354	1.00	0.00	H
ATOM	325	CA	GLN	21	39.612	10.817	4.814	1.00	0.00	C
ATOM	326	HA	GLN	21	39.470	11.527	5.629	1.00	0.00	H
ATOM	327	CB	GLN	21	38.486	11.177	3.761	1.00	0.00	C
ATOM	328	HB2	GLN	21	38.553	12.209	3.414	1.00	0.00	H
ATOM	329	HB3	GLN	21	37.630	11.216	4.435	1.00	0.00	H
ATOM	330	CG	GLN	21	38.232	10.272	2.612	1.00	0.00	C
ATOM	331	HG2	GLN	21	38.056	9.297	3.065	1.00	0.00	H
ATOM	332	HG3	GLN	21	39.111	10.423	1.986	1.00	0.00	H
ATOM	333	CD	GLN	21	36.979	10.620	1.797	1.00	0.00	C
ATOM	334	OE1	GLN	21	36.132	11.463	2.055	1.00	0.00	O
ATOM	335	NE2	GLN	21	36.738	10.005	0.656	1.00	0.00	N
ATOM	336	HE21	GLN	21	35.917	10.323	0.160	1.00	0.00	H
ATOM	337	HE22	GLN	21	37.245	9.158	0.442	1.00	0.00	H
ATOM	338	C	GLN	21	39.542	9.435	5.366	1.00	0.00	C
ATOM	339	O	GLN	21	39.900	8.424	4.697	1.00	0.00	O
ATOM	340	N	TRP	22	39.022	9.306	6.626	1.00	0.00	N
ATOM	341	H	TRP	22	38.591	10.161	6.947	1.00	0.00	H
ATOM	342	CA	TRP	22	38.870	8.098	7.451	1.00	0.00	C
ATOM	343	HA	TRP	22	38.900	7.310	6.699	1.00	0.00	H
ATOM	344	CB	TRP	22	39.894	8.184	8.594	1.00	0.00	C
ATOM	345	HB2	TRP	22	40.942	8.210	8.292	1.00	0.00	H
ATOM	346	HB3	TRP	22	39.718	7.283	9.180	1.00	0.00	H
ATOM	347	CG	TRP	22	39.810	9.420	9.531	1.00	0.00	C
ATOM	348	CD1	TRP	22	40.478	10.571	9.465	1.00	0.00	C
ATOM	349	HD1	TRP	22	41.029	10.908	8.599	1.00	0.00	H
ATOM	350	NE1	TRP	22	40.218	11.372	10.514	1.00	0.00	N
ATOM	351	HE1	TRP	22	40.725	12.232	10.669	1.00	0.00	H
ATOM	352	CE2	TRP	22	39.541	10.580	11.453	1.00	0.00	C
ATOM	353	CZ2	TRP	22	39.276	10.869	12.790	1.00	0.00	C
ATOM	354	HZ2	TRP	22	39.416	11.874	13.162	1.00	0.00	H
ATOM	355	CH2	TRP	22	38.532	9.876	13.550	1.00	0.00	C
ATOM	356	HH2	TRP	22	38.332	10.088	14.590	1.00	0.00	H
ATOM	357	CZ3	TRP	22	37.978	8.714	12.905	1.00	0.00	C
ATOM	358	HZ3	TRP	22	37.297	8.073	13.445	1.00	0.00	H
ATOM	359	CE3	TRP	22	38.447	8.449	11.609	1.00	0.00	C

ATOM	360	HE3	TRP	22	38.138	7.566	11.069	1.00	0.00	H
ATOM	361	CD2	TRP	22	39.219	9.383	10.829	1.00	0.00	C
ATOM	362	C	TRP	22	37.491	8.130	7.987	1.00	0.00	C
ATOM	363	O	TRP	22	36.667	9.076	7.732	1.00	0.00	O
ATOM	364	N	ASN	23	37.026	7.028	8.627	1.00	0.00	N
ATOM	365	H	ASN	23	37.592	6.196	8.533	1.00	0.00	H
ATOM	366	CA	ASN	23	35.646	6.759	9.044	1.00	0.00	C
ATOM	367	HA	ASN	23	34.925	7.418	8.562	1.00	0.00	H
ATOM	368	CB	ASN	23	35.293	5.309	8.648	1.00	0.00	C
ATOM	369	HB2	ASN	23	36.061	4.679	9.096	1.00	0.00	H
ATOM	370	HB3	ASN	23	35.239	5.500	7.576	1.00	0.00	H
ATOM	371	CG	ASN	23	33.894	4.918	9.232	1.00	0.00	C
ATOM	372	OD1	ASN	23	33.057	5.789	9.421	1.00	0.00	O
ATOM	373	ND2	ASN	23	33.628	3.648	9.377	1.00	0.00	N
ATOM	374	HD21	ASN	23	32.773	3.327	9.808	1.00	0.00	H
ATOM	375	HD22	ASN	23	34.388	3.026	9.138	1.00	0.00	H
ATOM	376	C	ASN	23	35.527	7.003	10.549	1.00	0.00	C
ATOM	377	O	ASN	23	36.186	6.254	11.300	1.00	0.00	O
ATOM	378	N	LYS	24	34.712	7.972	10.978	1.00	0.00	N
ATOM	379	H	LYS	24	34.135	8.346	10.239	1.00	0.00	H
ATOM	380	CA	LYS	24	34.312	8.301	12.365	1.00	0.00	C
ATOM	381	HA	LYS	24	34.881	7.598	12.974	1.00	0.00	H
ATOM	382	CB	LYS	24	34.641	9.695	12.928	1.00	0.00	C
ATOM	383	HB2	LYS	24	35.708	9.917	12.908	1.00	0.00	H
ATOM	384	HB3	LYS	24	34.547	9.586	14.008	1.00	0.00	H
ATOM	385	CG	LYS	24	33.919	10.957	12.496	1.00	0.00	C
ATOM	386	HG2	LYS	24	34.247	11.794	13.113	1.00	0.00	H
ATOM	387	HG3	LYS	24	32.847	10.765	12.477	1.00	0.00	H
ATOM	388	CD	LYS	24	34.396	11.415	11.098	1.00	0.00	C
ATOM	389	HD2	LYS	24	33.862	12.322	10.817	1.00	0.00	H
ATOM	390	HD3	LYS	24	34.151	10.599	10.418	1.00	0.00	H
ATOM	391	CE	LYS	24	35.880	11.780	11.062	1.00	0.00	C
ATOM	392	HE2	LYS	24	36.476	10.942	11.423	1.00	0.00	H
ATOM	393	HE3	LYS	24	36.009	12.584	11.785	1.00	0.00	H
ATOM	394	NZ	LYS	24	36.260	12.139	9.628	1.00	0.00	N
ATOM	395	HZ1	LYS	24	37.248	11.951	9.530	1.00	0.00	H
ATOM	396	HZ2	LYS	24	35.648	11.668	8.976	1.00	0.00	H
ATOM	397	HZ3	LYS	24	36.112	13.107	9.381	1.00	0.00	H
ATOM	398	C	LYS	24	32.797	8.018	12.427	1.00	0.00	C
ATOM	399	O	LYS	24	32.153	8.248	11.406	1.00	0.00	O
ATOM	400	N	PRO	25	32.226	7.415	13.491	1.00	0.00	N
ATOM	401	CD	PRO	25	32.858	6.983	14.741	1.00	0.00	C
ATOM	402	HD2	PRO	25	33.478	6.116	14.512	1.00	0.00	H
ATOM	403	HD3	PRO	25	33.435	7.831	15.111	1.00	0.00	H
ATOM	404	CG	PRO	25	31.778	6.670	15.715	1.00	0.00	C
ATOM	405	HG2	PRO	25	32.190	6.052	16.513	1.00	0.00	H
ATOM	406	HG3	PRO	25	31.277	7.591	16.013	1.00	0.00	H
ATOM	407	CB	PRO	25	30.725	5.973	14.823	1.00	0.00	C
ATOM	408	HB2	PRO	25	30.964	4.932	14.602	1.00	0.00	H
ATOM	409	HB3	PRO	25	29.708	6.130	15.181	1.00	0.00	H
ATOM	410	CA	PRO	25	30.925	6.836	13.558	1.00	0.00	C
ATOM	411	HA	PRO	25	30.702	6.202	12.700	1.00	0.00	H
ATOM	412	C	PRO	25	29.780	7.896	13.566	1.00	0.00	C
ATOM	413	O	PRO	25	29.507	8.659	14.522	1.00	0.00	O
ATOM	414	N	SER	26	29.015	7.707	12.509	1.00	0.00	N
ATOM	415	H	SER	26	29.405	7.046	11.852	1.00	0.00	H
ATOM	416	CA	SER	26	27.656	8.307	12.133	1.00	0.00	C
ATOM	417	HA	SER	26	27.651	9.321	12.530	1.00	0.00	H
ATOM	418	CB	SER	26	27.421	8.567	10.620	1.00	0.00	C
ATOM	419	HB2	SER	26	26.409	8.856	10.336	1.00	0.00	H
ATOM	420	HB3	SER	26	27.583	7.599	10.146	1.00	0.00	H
ATOM	421	OG	SER	26	28.447	9.533	10.111	1.00	0.00	O
ATOM	422	HG	SER	26	29.040	9.025	9.552	1.00	0.00	H

ATOM	423	C	SER	26	26.559	7.522	12.854	1.00	0.00	C
ATOM	424	O	SER	26	26.758	6.326	13.218	1.00	0.00	O
ATOM	425	N	LYS	27	25.401	8.165	13.046	1.00	0.00	N
ATOM	426	H	LYS	27	25.314	9.113	12.707	1.00	0.00	H
ATOM	427	CA	LYS	27	24.196	7.557	13.743	1.00	0.00	C
ATOM	428	HA	LYS	27	24.503	6.801	14.465	1.00	0.00	H
ATOM	429	CB	LYS	27	23.537	8.675	14.562	1.00	0.00	C
ATOM	430	HB2	LYS	27	22.642	8.387	15.115	1.00	0.00	H
ATOM	431	HB3	LYS	27	23.334	9.382	13.758	1.00	0.00	H
ATOM	432	CG	LYS	27	24.415	9.306	15.660	1.00	0.00	C
ATOM	433	HG2	LYS	27	25.273	9.776	15.178	1.00	0.00	H
ATOM	434	HG3	LYS	27	24.585	8.464	16.331	1.00	0.00	H
ATOM	435	CD	LYS	27	23.623	10.368	16.432	1.00	0.00	C
ATOM	436	HD2	LYS	27	22.620	10.008	16.665	1.00	0.00	H
ATOM	437	HD3	LYS	27	23.502	11.122	15.655	1.00	0.00	H
ATOM	438	CE	LYS	27	24.263	11.147	17.591	1.00	0.00	C
ATOM	439	HE2	LYS	27	23.658	12.053	17.650	1.00	0.00	H
ATOM	440	HE3	LYS	27	25.267	11.379	17.238	1.00	0.00	H
ATOM	441	NZ	LYS	27	24.268	10.498	18.916	1.00	0.00	N
ATOM	442	HZ1	LYS	27	23.365	10.576	19.362	1.00	0.00	H
ATOM	443	HZ2	LYS	27	24.556	9.532	18.848	1.00	0.00	H
ATOM	444	HZ3	LYS	27	24.949	11.019	19.450	1.00	0.00	H
ATOM	445	C	LYS	27	23.137	6.821	12.875	1.00	0.00	C
ATOM	446	O	LYS	27	23.133	7.123	11.654	1.00	0.00	O
ATOM	447	N	PRO	28	22.294	5.986	13.331	1.00	0.00	N
ATOM	448	CD	PRO	28	22.427	5.345	14.688	1.00	0.00	C
ATOM	449	HD2	PRO	28	23.463	5.076	14.894	1.00	0.00	H
ATOM	450	HD3	PRO	28	22.065	5.977	15.500	1.00	0.00	H
ATOM	451	CG	PRO	28	21.512	4.099	14.756	1.00	0.00	C
ATOM	452	HG2	PRO	28	21.979	3.238	14.279	1.00	0.00	H
ATOM	453	HG3	PRO	28	21.041	3.825	15.700	1.00	0.00	H
ATOM	454	CB	PRO	28	20.496	4.395	13.636	1.00	0.00	C
ATOM	455	HB2	PRO	28	20.033	3.533	13.154	1.00	0.00	H
ATOM	456	HB3	PRO	28	19.853	4.998	14.278	1.00	0.00	H
ATOM	457	CA	PRO	28	21.236	5.303	12.617	1.00	0.00	C
ATOM	458	HA	PRO	28	21.728	4.598	11.946	1.00	0.00	H
ATOM	459	C	PRO	28	20.344	6.254	11.858	1.00	0.00	C
ATOM	460	O	PRO	28	19.845	5.787	10.790	1.00	0.00	O
ATOM	461	N	LYS	29	20.099	7.482	12.372	1.00	0.00	N
ATOM	462	H	LYS	29	20.497	7.833	13.231	1.00	0.00	H
ATOM	463	CA	LYS	29	19.324	8.483	11.633	1.00	0.00	C
ATOM	464	HA	LYS	29	18.373	8.047	11.327	1.00	0.00	H
ATOM	465	CB	LYS	29	18.826	9.643	12.552	1.00	0.00	C
ATOM	466	HB2	LYS	29	18.102	10.187	11.945	1.00	0.00	H
ATOM	467	HB3	LYS	29	19.579	10.306	12.977	1.00	0.00	H
ATOM	468	CG	LYS	29	18.002	9.099	13.724	1.00	0.00	C
ATOM	469	HG2	LYS	29	18.596	8.462	14.381	1.00	0.00	H
ATOM	470	HG3	LYS	29	17.199	8.552	13.231	1.00	0.00	H
ATOM	471	CD	LYS	29	17.494	10.222	14.650	1.00	0.00	C
ATOM	472	HD2	LYS	29	16.880	10.959	14.131	1.00	0.00	H
ATOM	473	HD3	LYS	29	18.376	10.746	15.017	1.00	0.00	H
ATOM	474	CE	LYS	29	16.794	9.553	15.802	1.00	0.00	C
ATOM	475	HE2	LYS	29	17.411	8.902	16.421	1.00	0.00	H
ATOM	476	HE3	LYS	29	15.840	9.086	15.557	1.00	0.00	H
ATOM	477	NZ	LYS	29	16.375	10.498	16.916	1.00	0.00	N
ATOM	478	HZ1	LYS	29	15.640	11.145	16.670	1.00	0.00	H
ATOM	479	HZ2	LYS	29	16.019	10.058	17.753	1.00	0.00	H
ATOM	480	HZ3	LYS	29	17.126	11.117	17.186	1.00	0.00	H
ATOM	481	C	LYS	29	19.929	8.988	10.274	1.00	0.00	C
ATOM	482	O	LYS	29	19.250	9.800	9.588	1.00	0.00	O
ATOM	483	N	THR	30	21.146	8.570	9.945	1.00	0.00	N
ATOM	484	H	THR	30	21.534	7.858	10.548	1.00	0.00	H
ATOM	485	CA	THR	30	21.874	8.855	8.710	1.00	0.00	C

ATOM	486	HA	THR	30	21.436	9.765	8.297	1.00	0.00	H
ATOM	487	CB	THR	30	23.407	9.102	8.926	1.00	0.00	C
ATOM	488	HB	THR	30	23.803	9.485	7.985	1.00	0.00	H
ATOM	489	CG2	THR	30	23.628	10.180	9.943	1.00	0.00	C
ATOM	490	HG21	THR	30	23.630	9.694	10.918	1.00	0.00	H
ATOM	491	HG22	THR	30	24.601	10.649	9.808	1.00	0.00	H
ATOM	492	HG23	THR	30	22.805	10.897	9.924	1.00	0.00	H
ATOM	493	OG1	THR	30	23.954	7.851	9.288	1.00	0.00	O
ATOM	494	HG1	THR	30	23.728	7.771	10.218	1.00	0.00	H
ATOM	495	C	THR	30	21.715	7.814	7.616	1.00	0.00	C
ATOM	496	O	THR	30	20.966	6.848	7.742	1.00	0.00	O
ATOM	497	N	ASN	31	22.419	8.044	6.471	1.00	0.00	N
ATOM	498	H	ASN	31	22.868	8.931	6.295	1.00	0.00	H
ATOM	499	CA	ASN	31	22.373	7.250	5.253	1.00	0.00	C
ATOM	500	HA	ASN	31	21.321	7.080	5.022	1.00	0.00	H
ATOM	501	CB	ASN	31	22.995	8.068	4.112	1.00	0.00	C
ATOM	502	HB2	ASN	31	22.601	9.084	4.101	1.00	0.00	H
ATOM	503	HB3	ASN	31	22.756	7.584	3.164	1.00	0.00	H
ATOM	504	CG	ASN	31	24.470	8.050	4.153	1.00	0.00	C
ATOM	505	OD1	ASN	31	25.055	7.219	3.509	1.00	0.00	O
ATOM	506	ND2	ASN	31	25.132	9.005	4.727	1.00	0.00	N
ATOM	507	HD21	ASN	31	26.130	9.056	4.878	1.00	0.00	H
ATOM	508	HD22	ASN	31	24.601	9.791	5.075	1.00	0.00	H
ATOM	509	C	ASN	31	22.969	5.774	5.407	1.00	0.00	C
ATOM	510	O	ASN	31	22.673	4.827	4.618	1.00	0.00	O
ATOM	511	N	MET	32	23.836	5.645	6.386	1.00	0.00	N
ATOM	512	H	MET	32	24.049	6.458	6.946	1.00	0.00	H
ATOM	513	CA	MET	32	24.631	4.417	6.713	1.00	0.00	C
ATOM	514	HA	MET	32	24.832	3.929	5.760	1.00	0.00	H
ATOM	515	CB	MET	32	26.042	4.762	7.184	1.00	0.00	C
ATOM	516	HB2	MET	32	26.609	3.879	7.480	1.00	0.00	H
ATOM	517	HB3	MET	32	25.918	5.544	7.933	1.00	0.00	H
ATOM	518	CG	MET	32	26.864	5.316	6.049	1.00	0.00	C
ATOM	519	HG2	MET	32	26.186	5.734	5.303	1.00	0.00	H
ATOM	520	HG3	MET	32	27.273	4.386	5.657	1.00	0.00	H
ATOM	521	SD	MET	32	28.235	6.366	6.517	1.00	0.00	S
ATOM	522	CE	MET	32	29.352	5.097	7.251	1.00	0.00	C
ATOM	523	HE1	MET	32	29.394	4.225	6.599	1.00	0.00	H
ATOM	524	HE2	MET	32	30.317	5.588	7.388	1.00	0.00	H
ATOM	525	HE3	MET	32	28.973	4.758	8.215	1.00	0.00	H
ATOM	526	C	MET	32	23.945	3.350	7.635	1.00	0.00	C
ATOM	527	O	MET	32	24.326	2.175	7.712	1.00	0.00	O
ATOM	528	N	LYS	33	22.955	3.832	8.413	1.00	0.00	N
ATOM	529	H	LYS	33	22.683	4.801	8.331	1.00	0.00	H
ATOM	530	CA	LYS	33	22.288	3.025	9.479	1.00	0.00	C
ATOM	531	HA	LYS	33	21.577	3.654	10.013	1.00	0.00	H
ATOM	532	CB	LYS	33	21.486	1.862	8.896	1.00	0.00	C
ATOM	533	HB2	LYS	33	20.914	1.416	9.710	1.00	0.00	H
ATOM	534	HB3	LYS	33	22.163	1.149	8.426	1.00	0.00	H
ATOM	535	CG	LYS	33	20.334	2.312	8.023	1.00	0.00	C
ATOM	536	HG2	LYS	33	20.658	2.927	7.184	1.00	0.00	H
ATOM	537	HG3	LYS	33	19.707	2.891	8.701	1.00	0.00	H
ATOM	538	CD	LYS	33	19.483	1.108	7.583	1.00	0.00	C
ATOM	539	HD2	LYS	33	18.969	0.683	8.446	1.00	0.00	H
ATOM	540	HD3	LYS	33	20.199	0.372	7.218	1.00	0.00	H
ATOM	541	CE	LYS	33	18.453	1.642	6.543	1.00	0.00	C
ATOM	542	HE2	LYS	33	18.046	0.806	5.973	1.00	0.00	H
ATOM	543	HE3	LYS	33	18.958	2.235	5.781	1.00	0.00	H
ATOM	544	NZ	LYS	33	17.304	2.318	7.148	1.00	0.00	N
ATOM	545	HZ1	LYS	33	17.531	3.106	7.736	1.00	0.00	H
ATOM	546	HZ2	LYS	33	16.633	2.653	6.472	1.00	0.00	H
ATOM	547	HZ3	LYS	33	16.719	1.743	7.738	1.00	0.00	H
ATOM	548	C	LYS	33	23.325	2.678	10.606	1.00	0.00	C

```
ATOM    549  O   LYS    33      23.174   1.619  11.187  1.00  0.00           O
ATOM    550  N   NME    34      24.326   3.524  10.865  1.00  0.00           N
ATOM    551  H   NME    34      24.324   4.420  10.398  1.00  0.00           H
ATOM    552  CH3 NME    34      25.408   3.190  11.846  1.00  0.00           C
ATOM    553 HH31 NME    34      25.035   3.226  12.870  1.00  0.00           H
ATOM    554 HH32 NME    34      25.736   2.163  11.684  1.00  0.00           H
ATOM    555 HH33 NME    34      26.287   3.834  11.833  1.00  0.00           H
TER     556      NME    34
END
```

Simulation where the distance between the N- and C-termini is restrained:

```
#Cluster   Frames     Frac  AvgDist    Stdev Centroid AvgCDist
       0   152488    0.762    1.776    0.901   197277    4.314
       1    47401    0.237    2.778    1.011    97715    4.055
       2      111    0.001    0.000    0.000   135831    4.266
```

Cluster 0:

```
ATOM      1 HH31 ACE     1      34.211  17.998   9.349  1.00  0.00           H
ATOM      2  CH3 ACE     1      34.165  18.015   8.260  1.00  0.00           C
ATOM      3 HH32 ACE     1      34.001  19.075   8.065  1.00  0.00           H
ATOM      4 HH33 ACE     1      33.269  17.444   8.017  1.00  0.00           H
ATOM      5  C   ACE     1      35.373  17.636   7.476  1.00  0.00           C
ATOM      6  O   ACE     1      35.924  18.619   6.820  1.00  0.00           O
ATOM      7  N   ASN     2      35.794  16.403   7.532  1.00  0.00           N
ATOM      8  H   ASN     2      35.243  15.871   8.190  1.00  0.00           H
ATOM      9  CA  ASN     2      36.841  15.800   6.653  1.00  0.00           C
ATOM     10  HA  ASN     2      36.925  16.337   5.709  1.00  0.00           H
ATOM     11  CB  ASN     2      38.196  15.946   7.366  1.00  0.00           C
ATOM     12  HB2 ASN     2      38.252  15.286   8.231  1.00  0.00           H
ATOM     13  HB3 ASN     2      38.207  16.985   7.695  1.00  0.00           H
ATOM     14  CG  ASN     2      39.421  15.558   6.522  1.00  0.00           C
ATOM     15  OD1 ASN     2      39.740  16.226   5.577  1.00  0.00           O
ATOM     16  ND2 ASN     2      40.255  14.657   6.943  1.00  0.00           N
ATOM     17 HD21 ASN     2      41.236  14.653   6.705  1.00  0.00           H
ATOM     18 HD22 ASN     2      39.969  14.024   7.676  1.00  0.00           H
ATOM     19  C   ASN     2      36.508  14.384   6.227  1.00  0.00           C
ATOM     20  O   ASN     2      36.904  13.415   6.812  1.00  0.00           O
ATOM     21  N   ASP     3      35.576  14.269   5.281  1.00  0.00           N
ATOM     22  H   ASP     3      35.219  15.120   4.873  1.00  0.00           H
ATOM     23  CA  ASP     3      34.961  13.057   4.699  1.00  0.00           C
ATOM     24  HA  ASP     3      34.765  12.378   5.529  1.00  0.00           H
ATOM     25  CB  ASP     3      33.611  13.406   4.100  1.00  0.00           C
ATOM     26  HB2 ASP     3      33.718  14.169   3.328  1.00  0.00           H
ATOM     27  HB3 ASP     3      33.025  13.731   4.960  1.00  0.00           H
ATOM     28  CG  ASP     3      32.852  12.196   3.457  1.00  0.00           C
ATOM     29  OD1 ASP     3      31.919  11.666   4.163  1.00  0.00           O
ATOM     30  OD2 ASP     3      33.103  11.689   2.342  1.00  0.00           O
ATOM     31  C   ASP     3      35.984  12.367   3.714  1.00  0.00           C
ATOM     32  O   ASP     3      36.747  13.041   3.076  1.00  0.00           O
ATOM     33  N   TYR     4      35.862  11.037   3.481  1.00  0.00           N
ATOM     34  H   TYR     4      35.286  10.530   4.137  1.00  0.00           H
ATOM     35  CA  TYR     4      36.649  10.240   2.548  1.00  0.00           C
ATOM     36  HA  TYR     4      36.806  10.854   1.661  1.00  0.00           H
ATOM     37  CB  TYR     4      37.970   9.793   3.185  1.00  0.00           C
```

```
ATOM     38  HB2 TYR     4      37.753   8.974   3.870  1.00  0.00           H
ATOM     39  HB3 TYR     4      38.460  10.685   3.576  1.00  0.00           H
ATOM     40  CG  TYR     4      38.984   9.213   2.137  1.00  0.00           C
ATOM     41  CD1 TYR     4      39.238   9.935   0.929  1.00  0.00           C
ATOM     42  HD1 TYR     4      38.798  10.911   0.790  1.00  0.00           H
ATOM     43  CE1 TYR     4      40.185   9.434  -0.036  1.00  0.00           C
ATOM     44  HE1 TYR     4      40.384   9.842  -1.016  1.00  0.00           H
ATOM     45  CZ  TYR     4      40.881   8.176   0.319  1.00  0.00           C
ATOM     46  OH  TYR     4      41.992   7.819  -0.429  1.00  0.00           O
ATOM     47  HH  TYR     4      41.986   8.443  -1.159  1.00  0.00           H
ATOM     48  CE2 TYR     4      40.576   7.502   1.504  1.00  0.00           C
ATOM     49  HE2 TYR     4      41.009   6.539   1.734  1.00  0.00           H
ATOM     50  CD2 TYR     4      39.644   8.025   2.392  1.00  0.00           C
ATOM     51  HD2 TYR     4      39.583   7.587   3.378  1.00  0.00           H
ATOM     52  C   TYR     4      35.855   9.046   2.163  1.00  0.00           C
ATOM     53  O   TYR     4      35.352   8.277   3.020  1.00  0.00           O
ATOM     54  N   GLU     5      35.663   8.893   0.842  1.00  0.00           N
ATOM     55  H   GLU     5      36.120   9.484   0.162  1.00  0.00           H
ATOM     56  CA  GLU     5      35.001   7.661   0.324  1.00  0.00           C
ATOM     57  HA  GLU     5      35.152   6.800   0.975  1.00  0.00           H
ATOM     58  CB  GLU     5      33.492   7.914   0.253  1.00  0.00           C
ATOM     59  HB2 GLU     5      33.295   8.695  -0.481  1.00  0.00           H
ATOM     60  HB3 GLU     5      33.267   8.334   1.233  1.00  0.00           H
ATOM     61  CG  GLU     5      32.542   6.695   0.056  1.00  0.00           C
ATOM     62  HG2 GLU     5      32.735   6.208  -0.900  1.00  0.00           H
ATOM     63  HG3 GLU     5      31.570   7.187   0.026  1.00  0.00           H
ATOM     64  CD  GLU     5      32.585   5.562   1.107  1.00  0.00           C
ATOM     65  OE1 GLU     5      31.719   5.397   1.970  1.00  0.00           O
ATOM     66  OE2 GLU     5      33.420   4.659   1.040  1.00  0.00           O
ATOM     67  C   GLU     5      35.662   7.273  -1.043  1.00  0.00           C
ATOM     68  O   GLU     5      35.625   8.096  -1.958  1.00  0.00           O
ATOM     69  N   ASP     6      36.168   6.017  -1.205  1.00  0.00           N
ATOM     70  H   ASP     6      36.278   5.449  -0.377  1.00  0.00           H
ATOM     71  CA  ASP     6      36.716   5.536  -2.475  1.00  0.00           C
ATOM     72  HA  ASP     6      36.884   6.399  -3.119  1.00  0.00           H
ATOM     73  CB  ASP     6      38.104   4.889  -2.201  1.00  0.00           C
ATOM     74  HB2 ASP     6      38.642   5.423  -1.417  1.00  0.00           H
ATOM     75  HB3 ASP     6      38.648   4.870  -3.145  1.00  0.00           H
ATOM     76  CG  ASP     6      38.054   3.515  -1.645  1.00  0.00           C
ATOM     77  OD1 ASP     6      37.273   3.379  -0.651  1.00  0.00           O
ATOM     78  OD2 ASP     6      38.895   2.702  -1.971  1.00  0.00           O
ATOM     79  C   ASP     6      35.735   4.570  -3.248  1.00  0.00           C
ATOM     80  O   ASP     6      36.141   4.052  -4.243  1.00  0.00           O
ATOM     81  N   ARG     7      34.519   4.337  -2.702  1.00  0.00           N
ATOM     82  H   ARG     7      34.350   4.730  -1.787  1.00  0.00           H
ATOM     83  CA  ARG     7      33.425   3.531  -3.199  1.00  0.00           C
ATOM     84  HA  ARG     7      33.882   2.693  -3.725  1.00  0.00           H
ATOM     85  CB  ARG     7      32.548   2.896  -2.164  1.00  0.00           C
ATOM     86  HB2 ARG     7      31.922   2.214  -2.740  1.00  0.00           H
ATOM     87  HB3 ARG     7      31.980   3.649  -1.617  1.00  0.00           H
ATOM     88  CG  ARG     7      33.375   2.005  -1.202  1.00  0.00           C
ATOM     89  HG2 ARG     7      34.168   2.557  -0.697  1.00  0.00           H
ATOM     90  HG3 ARG     7      33.810   1.300  -1.910  1.00  0.00           H
ATOM     91  CD  ARG     7      32.644   1.195  -0.164  1.00  0.00           C
ATOM     92  HD2 ARG     7      33.306   0.458   0.291  1.00  0.00           H
ATOM     93  HD3 ARG     7      31.847   0.709  -0.727  1.00  0.00           H
ATOM     94  NE  ARG     7      31.967   2.079   0.746  1.00  0.00           N
ATOM     95  HE  ARG     7      32.446   2.960   0.860  1.00  0.00           H
ATOM     96  CZ  ARG     7      30.750   2.001   1.247  1.00  0.00           C
ATOM     97  NH1 ARG     7      30.020   0.894   1.242  1.00  0.00           N
ATOM     98 HH11 ARG     7      30.433   0.007   0.991  1.00  0.00           H
ATOM     99 HH12 ARG     7      29.258   0.920   1.905  1.00  0.00           H
ATOM    100  NH2 ARG     7      30.183   3.063   1.811  1.00  0.00           N
```

ATOM	101	HH21	ARG	7	29.288	2.987	2.272	1.00	0.00	H
ATOM	102	HH22	ARG	7	30.779	3.872	1.910	1.00	0.00	H
ATOM	103	C	ARG	7	32.676	4.445	-4.172	1.00	0.00	C
ATOM	104	O	ARG	7	32.514	5.626	-3.851	1.00	0.00	O
ATOM	105	N	TYR	8	32.160	3.879	-5.262	1.00	0.00	N
ATOM	106	H	TYR	8	32.249	2.892	-5.456	1.00	0.00	H
ATOM	107	CA	TYR	8	31.413	4.543	-6.289	1.00	0.00	C
ATOM	108	HA	TYR	8	31.939	5.489	-6.411	1.00	0.00	H
ATOM	109	CB	TYR	8	31.471	3.750	-7.600	1.00	0.00	C
ATOM	110	HB2	TYR	8	32.524	3.765	-7.882	1.00	0.00	H
ATOM	111	HB3	TYR	8	30.858	4.274	-8.334	1.00	0.00	H
ATOM	112	CG	TYR	8	31.128	2.281	-7.536	1.00	0.00	C
ATOM	113	CD1	TYR	8	29.770	1.865	-7.588	1.00	0.00	C
ATOM	114	HD1	TYR	8	29.048	2.667	-7.545	1.00	0.00	H
ATOM	115	CE1	TYR	8	29.334	0.523	-7.551	1.00	0.00	C
ATOM	116	HE1	TYR	8	28.274	0.345	-7.664	1.00	0.00	H
ATOM	117	CZ	TYR	8	30.342	-0.472	-7.564	1.00	0.00	C
ATOM	118	OH	TYR	8	29.988	-1.804	-7.584	1.00	0.00	O
ATOM	119	HH	TYR	8	29.045	-1.925	-7.448	1.00	0.00	H
ATOM	120	CE2	TYR	8	31.667	-0.055	-7.575	1.00	0.00	C
ATOM	121	HE2	TYR	8	32.390	-0.856	-7.615	1.00	0.00	H
ATOM	122	CD2	TYR	8	32.046	1.274	-7.516	1.00	0.00	C
ATOM	123	HD2	TYR	8	33.084	1.555	-7.417	1.00	0.00	H
ATOM	124	C	TYR	8	29.915	4.770	-5.898	1.00	0.00	C
ATOM	125	O	TYR	8	29.160	5.428	-6.615	1.00	0.00	O
ATOM	126	N	TYR	9	29.470	4.186	-4.745	1.00	0.00	N
ATOM	127	H	TYR	9	30.080	3.602	-4.191	1.00	0.00	H
ATOM	128	CA	TYR	9	28.211	4.412	-4.091	1.00	0.00	C
ATOM	129	HA	TYR	9	27.875	5.417	-4.345	1.00	0.00	H
ATOM	130	CB	TYR	9	27.096	3.480	-4.608	1.00	0.00	C
ATOM	131	HB2	TYR	9	27.365	3.082	-5.587	1.00	0.00	H
ATOM	132	HB3	TYR	9	26.243	4.138	-4.444	1.00	0.00	H
ATOM	133	CG	TYR	9	26.893	2.114	-3.842	1.00	0.00	C
ATOM	134	CD1	TYR	9	25.832	2.097	-2.936	1.00	0.00	C
ATOM	135	HD1	TYR	9	25.209	2.972	-2.824	1.00	0.00	H
ATOM	136	CE1	TYR	9	25.666	0.993	-2.151	1.00	0.00	C
ATOM	137	HE1	TYR	9	24.829	0.940	-1.471	1.00	0.00	H
ATOM	138	CZ	TYR	9	26.543	-0.109	-2.209	1.00	0.00	C
ATOM	139	OH	TYR	9	26.364	-1.259	-1.430	1.00	0.00	O
ATOM	140	HH	TYR	9	27.141	-1.814	-1.525	1.00	0.00	H
ATOM	141	CE2	TYR	9	27.619	-0.079	-3.083	1.00	0.00	C
ATOM	142	HE2	TYR	9	28.314	-0.899	-3.182	1.00	0.00	H
ATOM	143	CD2	TYR	9	27.815	1.064	-3.895	1.00	0.00	C
ATOM	144	HD2	TYR	9	28.676	1.136	-4.544	1.00	0.00	H
ATOM	145	C	TYR	9	28.335	4.267	-2.579	1.00	0.00	C
ATOM	146	O	TYR	9	29.182	3.582	-1.981	1.00	0.00	O
ATOM	147	N	ARG	10	27.484	5.061	-1.920	1.00	0.00	N
ATOM	148	H	ARG	10	26.792	5.506	-2.506	1.00	0.00	H
ATOM	149	CA	ARG	10	27.398	5.241	-0.480	1.00	0.00	C
ATOM	150	HA	ARG	10	27.975	4.401	-0.094	1.00	0.00	H
ATOM	151	CB	ARG	10	28.191	6.495	-0.035	1.00	0.00	C
ATOM	152	HB2	ARG	10	29.212	6.493	-0.416	1.00	0.00	H
ATOM	153	HB3	ARG	10	28.230	6.181	1.008	1.00	0.00	H
ATOM	154	CG	ARG	10	27.428	7.834	-0.094	1.00	0.00	C
ATOM	155	HG2	ARG	10	26.572	7.834	0.581	1.00	0.00	H
ATOM	156	HG3	ARG	10	27.107	7.836	-1.136	1.00	0.00	H
ATOM	157	CD	ARG	10	28.411	8.950	0.269	1.00	0.00	C
ATOM	158	HD2	ARG	10	27.887	9.904	0.216	1.00	0.00	H
ATOM	159	HD3	ARG	10	29.164	8.783	-0.501	1.00	0.00	H
ATOM	160	NE	ARG	10	29.022	8.929	1.665	1.00	0.00	N
ATOM	161	HE	ARG	10	28.461	8.628	2.449	1.00	0.00	H
ATOM	162	CZ	ARG	10	30.212	9.499	1.881	1.00	0.00	C
ATOM	163	NH1	ARG	10	30.843	10.107	0.898	1.00	0.00	N

ATOM	164	HH11	ARG	10	30.409	9.918	0.005	1.00	0.00	H
ATOM	165	HH12	ARG	10	31.771	10.465	1.073	1.00	0.00	H
ATOM	166	NH2	ARG	10	30.561	9.609	3.122	1.00	0.00	N
ATOM	167	HH21	ARG	10	29.945	9.331	3.873	1.00	0.00	H
ATOM	168	HH22	ARG	10	31.169	10.389	3.323	1.00	0.00	H
ATOM	169	C	ARG	10	26.043	5.136	0.182	1.00	0.00	C
ATOM	170	O	ARG	10	25.048	5.270	-0.424	1.00	0.00	O
ATOM	171	N	GLU	11	26.020	4.543	1.387	1.00	0.00	N
ATOM	172	H	GLU	11	26.916	4.204	1.707	1.00	0.00	H
ATOM	173	CA	GLU	11	24.794	4.250	2.199	1.00	0.00	C
ATOM	174	HA	GLU	11	23.884	4.354	1.608	1.00	0.00	H
ATOM	175	CB	GLU	11	24.784	2.808	2.694	1.00	0.00	C
ATOM	176	HB2	GLU	11	24.772	2.049	1.911	1.00	0.00	H
ATOM	177	HB3	GLU	11	23.771	2.684	3.074	1.00	0.00	H
ATOM	178	CG	GLU	11	25.757	2.394	3.803	1.00	0.00	C
ATOM	179	HG2	GLU	11	25.504	1.392	4.150	1.00	0.00	H
ATOM	180	HG3	GLU	11	25.653	3.171	4.560	1.00	0.00	H
ATOM	181	CD	GLU	11	27.198	2.315	3.305	1.00	0.00	C
ATOM	182	OE1	GLU	11	27.642	1.257	2.801	1.00	0.00	O
ATOM	183	OE2	GLU	11	27.930	3.315	3.424	1.00	0.00	O
ATOM	184	C	GLU	11	24.704	5.234	3.378	1.00	0.00	C
ATOM	185	O	GLU	11	23.676	5.318	4.005	1.00	0.00	O
ATOM	186	N	ASN	12	25.710	6.010	3.639	1.00	0.00	N
ATOM	187	H	ASN	12	26.527	5.948	3.047	1.00	0.00	H
ATOM	188	CA	ASN	12	25.828	6.871	4.823	1.00	0.00	C
ATOM	189	HA	ASN	12	24.956	6.785	5.471	1.00	0.00	H
ATOM	190	CB	ASN	12	27.134	6.495	5.579	1.00	0.00	C
ATOM	191	HB2	ASN	12	27.099	5.439	5.845	1.00	0.00	H
ATOM	192	HB3	ASN	12	27.065	7.041	6.520	1.00	0.00	H
ATOM	193	CG	ASN	12	28.368	6.754	4.767	1.00	0.00	C
ATOM	194	OD1	ASN	12	28.801	7.947	4.724	1.00	0.00	O
ATOM	195	ND2	ASN	12	28.970	5.839	4.071	1.00	0.00	N
ATOM	196	HD21	ASN	12	29.834	6.063	3.598	1.00	0.00	H
ATOM	197	HD22	ASN	12	28.556	4.919	4.023	1.00	0.00	H
ATOM	198	C	ASN	12	25.999	8.320	4.539	1.00	0.00	C
ATOM	199	O	ASN	12	26.396	8.704	3.454	1.00	0.00	O
ATOM	200	N	MET	13	25.586	9.167	5.502	1.00	0.00	N
ATOM	201	H	MET	13	25.042	8.893	6.308	1.00	0.00	H
ATOM	202	CA	MET	13	25.740	10.615	5.236	1.00	0.00	C
ATOM	203	HA	MET	13	25.298	10.887	4.278	1.00	0.00	H
ATOM	204	CB	MET	13	24.936	11.406	6.295	1.00	0.00	C
ATOM	205	HB2	MET	13	25.371	12.405	6.331	1.00	0.00	H
ATOM	206	HB3	MET	13	24.976	10.846	7.229	1.00	0.00	H
ATOM	207	CG	MET	13	23.496	11.736	5.839	1.00	0.00	C
ATOM	208	HG2	MET	13	23.016	12.266	6.662	1.00	0.00	H
ATOM	209	HG3	MET	13	23.072	10.756	5.623	1.00	0.00	H
ATOM	210	SD	MET	13	23.405	12.850	4.413	1.00	0.00	S
ATOM	211	CE	MET	13	21.620	12.692	4.111	1.00	0.00	C
ATOM	212	HE1	MET	13	21.001	12.836	4.997	1.00	0.00	H
ATOM	213	HE2	MET	13	21.366	13.410	3.331	1.00	0.00	H
ATOM	214	HE3	MET	13	21.343	11.718	3.708	1.00	0.00	H
ATOM	215	C	MET	13	27.205	11.182	5.235	1.00	0.00	C
ATOM	216	O	MET	13	27.501	12.183	4.616	1.00	0.00	O
ATOM	217	N	TYR	14	28.083	10.534	6.043	1.00	0.00	N
ATOM	218	H	TYR	14	27.875	9.600	6.367	1.00	0.00	H
ATOM	219	CA	TYR	14	29.397	11.098	6.400	1.00	0.00	C
ATOM	220	HA	TYR	14	29.909	11.479	5.516	1.00	0.00	H
ATOM	221	CB	TYR	14	29.377	12.290	7.433	1.00	0.00	C
ATOM	222	HB2	TYR	14	28.718	12.224	8.299	1.00	0.00	H
ATOM	223	HB3	TYR	14	28.805	13.061	6.917	1.00	0.00	H
ATOM	224	CG	TYR	14	30.662	12.809	7.889	1.00	0.00	C
ATOM	225	CD1	TYR	14	31.338	13.870	7.265	1.00	0.00	C
ATOM	226	HD1	TYR	14	31.005	14.288	6.327	1.00	0.00	H

```
ATOM    227  CE1 TYR    14      32.549  14.345   7.748  1.00  0.00           C
ATOM    228  HE1 TYR    14      33.015  15.192   7.267  1.00  0.00           H
ATOM    229  CZ  TYR    14      33.108  13.824   8.901  1.00  0.00           C
ATOM    230  OH  TYR    14      34.389  14.288   9.257  1.00  0.00           O
ATOM    231  HH  TYR    14      34.570  13.744  10.027  1.00  0.00           H
ATOM    232  CE2 TYR    14      32.430  12.791   9.584  1.00  0.00           C
ATOM    233  HE2 TYR    14      32.851  12.325  10.463  1.00  0.00           H
ATOM    234  CD2 TYR    14      31.195  12.300   9.090  1.00  0.00           C
ATOM    235  HD2 TYR    14      30.724  11.504   9.646  1.00  0.00           H
ATOM    236  C   TYR    14      30.278   9.929   6.968  1.00  0.00           C
ATOM    237  O   TYR    14      29.838   9.141   7.753  1.00  0.00           O
ATOM    238  N   ARG    15      31.495   9.844   6.409  1.00  0.00           N
ATOM    239  H   ARG    15      31.684  10.463   5.634  1.00  0.00           H
ATOM    240  CA  ARG    15      32.612   8.911   6.783  1.00  0.00           C
ATOM    241  HA  ARG    15      32.476   8.515   7.789  1.00  0.00           H
ATOM    242  CB  ARG    15      32.657   7.727   5.811  1.00  0.00           C
ATOM    243  HB2 ARG    15      32.819   8.130   4.812  1.00  0.00           H
ATOM    244  HB3 ARG    15      31.655   7.308   5.911  1.00  0.00           H
ATOM    245  CG  ARG    15      33.736   6.701   6.094  1.00  0.00           C
ATOM    246  HG2 ARG    15      33.724   6.499   7.165  1.00  0.00           H
ATOM    247  HG3 ARG    15      34.718   7.043   5.765  1.00  0.00           H
ATOM    248  CD  ARG    15      33.379   5.351   5.441  1.00  0.00           C
ATOM    249  HD2 ARG    15      32.324   5.258   5.184  1.00  0.00           H
ATOM    250  HD3 ARG    15      33.766   4.522   6.033  1.00  0.00           H
ATOM    251  NE  ARG    15      34.009   5.121   4.088  1.00  0.00           N
ATOM    252  HE  ARG    15      33.355   5.183   3.320  1.00  0.00           H
ATOM    253  CZ  ARG    15      35.235   4.641   3.870  1.00  0.00           C
ATOM    254  NH1 ARG    15      36.108   4.411   4.801  1.00  0.00           N
ATOM    255 HH11 ARG    15      35.986   4.511   5.798  1.00  0.00           H
ATOM    256 HH12 ARG    15      37.062   4.207   4.541  1.00  0.00           H
ATOM    257  NH2 ARG    15      35.663   4.400   2.698  1.00  0.00           N
ATOM    258 HH21 ARG    15      34.991   4.279   1.954  1.00  0.00           H
ATOM    259 HH22 ARG    15      36.603   4.050   2.587  1.00  0.00           H
ATOM    260  C   ARG    15      33.941   9.703   6.982  1.00  0.00           C
ATOM    261  O   ARG    15      34.394  10.286   6.029  1.00  0.00           O
ATOM    262  N   TYR    16      34.435   9.706   8.180  1.00  0.00           N
ATOM    263  H   TYR    16      33.921   9.111   8.814  1.00  0.00           H
ATOM    264  CA  TYR    16      35.725  10.266   8.573  1.00  0.00           C
ATOM    265  HA  TYR    16      35.732  11.351   8.468  1.00  0.00           H
ATOM    266  CB  TYR    16      35.963   9.946  10.041  1.00  0.00           C
ATOM    267  HB2 TYR    16      36.255   8.908  10.203  1.00  0.00           H
ATOM    268  HB3 TYR    16      35.001  10.026  10.547  1.00  0.00           H
ATOM    269  CG  TYR    16      37.023  10.749  10.773  1.00  0.00           C
ATOM    270  CD1 TYR    16      38.273  10.108  11.160  1.00  0.00           C
ATOM    271  HD1 TYR    16      38.300   9.032  11.069  1.00  0.00           H
ATOM    272  CE1 TYR    16      39.298  10.903  11.636  1.00  0.00           C
ATOM    273  HE1 TYR    16      40.234  10.488  11.977  1.00  0.00           H
ATOM    274  CZ  TYR    16      39.135  12.357  11.667  1.00  0.00           C
ATOM    275  OH  TYR    16      40.153  13.086  12.195  1.00  0.00           O
ATOM    276  HH  TYR    16      40.931  12.547  12.031  1.00  0.00           H
ATOM    277  CE2 TYR    16      37.913  12.943  11.443  1.00  0.00           C
ATOM    278  HE2 TYR    16      37.717  13.995  11.589  1.00  0.00           H
ATOM    279  CD2 TYR    16      36.872  12.143  10.889  1.00  0.00           C
ATOM    280  HD2 TYR    16      35.992  12.613  10.476  1.00  0.00           H
ATOM    281  C   TYR    16      36.859   9.733   7.669  1.00  0.00           C
ATOM    282  O   TYR    16      36.837   8.557   7.215  1.00  0.00           O
ATOM    283  N   NME    17      37.922  10.554   7.584  1.00  0.00           N
ATOM    284  H   NME    17      37.868  11.415   8.108  1.00  0.00           H
ATOM    285  CH3 NME    17      39.146  10.487   6.780  1.00  0.00           C
ATOM    286 HH31 NME    17      39.374   9.439   6.586  1.00  0.00           H
ATOM    287 HH32 NME    17      38.997  10.921   5.791  1.00  0.00           H
ATOM    288 HH33 NME    17      39.997  10.984   7.246  1.00  0.00           H
TER     289      NME    17
```

```
END
```

Cluster 1:

```
ATOM      1 HH31 ACE     1      38.120   6.625   5.776  1.00  0.00           H
ATOM      2  CH3 ACE     1      39.155   6.963   5.823  1.00  0.00           C
ATOM      3 HH32 ACE     1      39.787   6.510   5.060  1.00  0.00           H
ATOM      4 HH33 ACE     1      39.522   6.664   6.804  1.00  0.00           H
ATOM      5  C   ACE     1      39.183   8.490   5.726  1.00  0.00           C
ATOM      6  O   ACE     1      39.435   9.175   6.706  1.00  0.00           O
ATOM      7  N   ASN     2      39.075   9.036   4.522  1.00  0.00           N
ATOM      8  H   ASN     2      38.929   8.383   3.766  1.00  0.00           H
ATOM      9  CA  ASN     2      39.042  10.456   4.038  1.00  0.00           C
ATOM     10  HA  ASN     2      38.782  11.155   4.834  1.00  0.00           H
ATOM     11  CB  ASN     2      40.414  10.873   3.660  1.00  0.00           C
ATOM     12  HB2 ASN     2      40.793  10.759   2.644  1.00  0.00           H
ATOM     13  HB3 ASN     2      41.077  10.265   4.275  1.00  0.00           H
ATOM     14  CG  ASN     2      40.652  12.354   3.996  1.00  0.00           C
ATOM     15  OD1 ASN     2      39.777  13.183   4.235  1.00  0.00           O
ATOM     16  ND2 ASN     2      41.910  12.792   3.976  1.00  0.00           N
ATOM     17 HD21 ASN     2      42.097  13.748   4.243  1.00  0.00           H
ATOM     18 HD22 ASN     2      42.685  12.157   3.850  1.00  0.00           H
ATOM     19  C   ASN     2      38.026  10.682   3.006  1.00  0.00           C
ATOM     20  O   ASN     2      37.479   9.833   2.323  1.00  0.00           O
ATOM     21  N   ASP     3      37.793  11.934   2.670  1.00  0.00           N
ATOM     22  H   ASP     3      38.243  12.590   3.293  1.00  0.00           H
ATOM     23  CA  ASP     3      36.797  12.419   1.760  1.00  0.00           C
ATOM     24  HA  ASP     3      35.847  12.237   2.264  1.00  0.00           H
ATOM     25  CB  ASP     3      36.917  13.974   1.859  1.00  0.00           C
ATOM     26  HB2 ASP     3      37.932  14.263   1.586  1.00  0.00           H
ATOM     27  HB3 ASP     3      36.636  14.315   2.856  1.00  0.00           H
ATOM     28  CG  ASP     3      35.983  14.663   0.810  1.00  0.00           C
ATOM     29  OD1 ASP     3      34.729  14.700   1.014  1.00  0.00           O
ATOM     30  OD2 ASP     3      36.439  15.157  -0.266  1.00  0.00           O
ATOM     31  C   ASP     3      36.893  11.999   0.302  1.00  0.00           C
ATOM     32  O   ASP     3      35.930  11.980  -0.493  1.00  0.00           O
ATOM     33  N   TYR     4      38.075  11.561  -0.234  1.00  0.00           N
ATOM     34  H   TYR     4      38.903  11.474   0.339  1.00  0.00           H
ATOM     35  CA  TYR     4      38.366  11.086  -1.614  1.00  0.00           C
ATOM     36  HA  TYR     4      37.759  11.712  -2.269  1.00  0.00           H
ATOM     37  CB  TYR     4      39.827  11.391  -2.010  1.00  0.00           C
ATOM     38  HB2 TYR     4      39.943  12.465  -1.872  1.00  0.00           H
ATOM     39  HB3 TYR     4      40.013  11.318  -3.082  1.00  0.00           H
ATOM     40  CG  TYR     4      40.955  10.611  -1.310  1.00  0.00           C
ATOM     41  CD1 TYR     4      41.111   9.201  -1.492  1.00  0.00           C
ATOM     42  HD1 TYR     4      40.532   8.761  -2.290  1.00  0.00           H
ATOM     43  CE1 TYR     4      42.104   8.517  -0.806  1.00  0.00           C
ATOM     44  HE1 TYR     4      42.225   7.456  -0.969  1.00  0.00           H
ATOM     45  CZ  TYR     4      42.884   9.222   0.101  1.00  0.00           C
ATOM     46  OH  TYR     4      43.784   8.537   0.860  1.00  0.00           O
ATOM     47  HH  TYR     4      44.293   9.191   1.345  1.00  0.00           H
ATOM     48  CE2 TYR     4      42.711  10.608   0.305  1.00  0.00           C
ATOM     49  HE2 TYR     4      43.329  11.203   0.960  1.00  0.00           H
ATOM     50  CD2 TYR     4      41.832  11.428  -0.452  1.00  0.00           C
ATOM     51  HD2 TYR     4      41.680  12.492  -0.347  1.00  0.00           H
ATOM     52  C   TYR     4      37.983   9.594  -1.951  1.00  0.00           C
ATOM     53  O   TYR     4      37.709   9.381  -3.122  1.00  0.00           O
ATOM     54  N   GLU     5      37.784   8.772  -0.921  1.00  0.00           N
ATOM     55  H   GLU     5      38.210   8.990  -0.031  1.00  0.00           H
ATOM     56  CA  GLU     5      37.269   7.430  -0.952  1.00  0.00           C
ATOM     57  HA  GLU     5      37.198   7.069  -1.978  1.00  0.00           H
```

ATOM	58	CB	GLU	5	38.317	6.481	-0.279	1.00	0.00	C
ATOM	59	HB2	GLU	5	37.990	6.425	0.759	1.00	0.00	H
ATOM	60	HB3	GLU	5	39.329	6.887	-0.289	1.00	0.00	H
ATOM	61	CG	GLU	5	38.316	5.096	-0.906	1.00	0.00	C
ATOM	62	HG2	GLU	5	38.774	5.062	-1.894	1.00	0.00	H
ATOM	63	HG3	GLU	5	37.246	4.918	-1.015	1.00	0.00	H
ATOM	64	CD	GLU	5	39.173	4.009	-0.109	1.00	0.00	C
ATOM	65	OE1	GLU	5	40.381	4.236	0.173	1.00	0.00	O
ATOM	66	OE2	GLU	5	38.610	2.857	0.175	1.00	0.00	O
ATOM	67	C	GLU	5	35.873	7.353	-0.257	1.00	0.00	C
ATOM	68	O	GLU	5	35.247	6.224	-0.288	1.00	0.00	O
ATOM	69	N	ASP	6	35.378	8.468	0.387	1.00	0.00	N
ATOM	70	H	ASP	6	36.079	9.153	0.634	1.00	0.00	H
ATOM	71	CA	ASP	6	34.100	8.495	1.002	1.00	0.00	C
ATOM	72	HA	ASP	6	33.986	7.772	1.810	1.00	0.00	H
ATOM	73	CB	ASP	6	33.850	9.976	1.597	1.00	0.00	C
ATOM	74	HB2	ASP	6	34.016	10.799	0.903	1.00	0.00	H
ATOM	75	HB3	ASP	6	34.562	10.043	2.420	1.00	0.00	H
ATOM	76	CG	ASP	6	32.442	10.112	2.267	1.00	0.00	C
ATOM	77	OD1	ASP	6	31.728	11.019	1.844	1.00	0.00	O
ATOM	78	OD2	ASP	6	32.158	9.349	3.264	1.00	0.00	O
ATOM	79	C	ASP	6	32.997	8.322	-0.118	1.00	0.00	C
ATOM	80	O	ASP	6	31.867	8.105	0.283	1.00	0.00	O
ATOM	81	N	ARG	7	33.236	8.559	-1.458	1.00	0.00	N
ATOM	82	H	ARG	7	34.179	8.702	-1.789	1.00	0.00	H
ATOM	83	CA	ARG	7	32.147	8.679	-2.399	1.00	0.00	C
ATOM	84	HA	ARG	7	31.299	9.154	-1.906	1.00	0.00	H
ATOM	85	CB	ARG	7	32.606	9.603	-3.621	1.00	0.00	C
ATOM	86	HB2	ARG	7	31.697	9.987	-4.083	1.00	0.00	H
ATOM	87	HB3	ARG	7	33.058	8.870	-4.290	1.00	0.00	H
ATOM	88	CG	ARG	7	33.515	10.825	-3.335	1.00	0.00	C
ATOM	89	HG2	ARG	7	33.678	11.322	-4.291	1.00	0.00	H
ATOM	90	HG3	ARG	7	34.406	10.302	-2.989	1.00	0.00	H
ATOM	91	CD	ARG	7	32.971	11.720	-2.221	1.00	0.00	C
ATOM	92	HD2	ARG	7	33.044	11.099	-1.330	1.00	0.00	H
ATOM	93	HD3	ARG	7	31.923	11.932	-2.439	1.00	0.00	H
ATOM	94	NE	ARG	7	33.714	13.003	-2.019	1.00	0.00	N
ATOM	95	HE	ARG	7	34.272	12.986	-1.178	1.00	0.00	H
ATOM	96	CZ	ARG	7	33.874	14.064	-2.745	1.00	0.00	C
ATOM	97	NH1	ARG	7	33.349	14.179	-3.922	1.00	0.00	N
ATOM	98	HH11	ARG	7	33.048	15.064	-4.307	1.00	0.00	H
ATOM	99	HH12	ARG	7	33.045	13.328	-4.373	1.00	0.00	H
ATOM	100	NH2	ARG	7	34.585	15.055	-2.300	1.00	0.00	N
ATOM	101	HH21	ARG	7	35.274	14.980	-1.566	1.00	0.00	H
ATOM	102	HH22	ARG	7	34.663	15.776	-3.004	1.00	0.00	H
ATOM	103	C	ARG	7	31.584	7.336	-2.921	1.00	0.00	C
ATOM	104	O	ARG	7	30.358	7.261	-3.039	1.00	0.00	O
ATOM	105	N	TYR	8	32.439	6.364	-3.092	1.00	0.00	N
ATOM	106	H	TYR	8	33.413	6.629	-3.120	1.00	0.00	H
ATOM	107	CA	TYR	8	32.234	4.944	-3.278	1.00	0.00	C
ATOM	108	HA	TYR	8	31.703	4.762	-4.213	1.00	0.00	H
ATOM	109	CB	TYR	8	33.596	4.256	-3.326	1.00	0.00	C
ATOM	110	HB2	TYR	8	33.479	3.179	-3.443	1.00	0.00	H
ATOM	111	HB3	TYR	8	34.073	4.543	-2.388	1.00	0.00	H
ATOM	112	CG	TYR	8	34.505	4.695	-4.478	1.00	0.00	C
ATOM	113	CD1	TYR	8	35.708	5.338	-4.254	1.00	0.00	C
ATOM	114	HD1	TYR	8	35.951	5.498	-3.213	1.00	0.00	H
ATOM	115	CE1	TYR	8	36.584	5.539	-5.300	1.00	0.00	C
ATOM	116	HE1	TYR	8	37.508	5.954	-4.923	1.00	0.00	H
ATOM	117	CZ	TYR	8	36.242	5.098	-6.637	1.00	0.00	C
ATOM	118	OH	TYR	8	36.978	5.498	-7.693	1.00	0.00	O
ATOM	119	HH	TYR	8	36.416	5.629	-8.462	1.00	0.00	H
ATOM	120	CE2	TYR	8	34.987	4.497	-6.880	1.00	0.00	C

ATOM	121	HE2	TYR	8	34.692	4.229	-7.884	1.00	0.00	H
ATOM	122	CD2	TYR	8	34.122	4.268	-5.757	1.00	0.00	C
ATOM	123	HD2	TYR	8	33.325	3.571	-5.974	1.00	0.00	H
ATOM	124	C	TYR	8	31.331	4.348	-2.252	1.00	0.00	C
ATOM	125	O	TYR	8	30.622	3.399	-2.546	1.00	0.00	O
ATOM	126	N	TYR	9	31.268	4.931	-1.035	1.00	0.00	N
ATOM	127	H	TYR	9	31.911	5.679	-0.814	1.00	0.00	H
ATOM	128	CA	TYR	9	30.493	4.440	0.071	1.00	0.00	C
ATOM	129	HA	TYR	9	30.025	3.528	-0.301	1.00	0.00	H
ATOM	130	CB	TYR	9	31.505	3.990	1.117	1.00	0.00	C
ATOM	131	HB2	TYR	9	31.047	3.274	1.799	1.00	0.00	H
ATOM	132	HB3	TYR	9	31.802	4.854	1.712	1.00	0.00	H
ATOM	133	CG	TYR	9	32.765	3.218	0.690	1.00	0.00	C
ATOM	134	CD1	TYR	9	32.668	2.166	-0.223	1.00	0.00	C
ATOM	135	HD1	TYR	9	31.691	1.858	-0.564	1.00	0.00	H
ATOM	136	CE1	TYR	9	33.816	1.444	-0.623	1.00	0.00	C
ATOM	137	HE1	TYR	9	33.732	0.517	-1.170	1.00	0.00	H
ATOM	138	CZ	TYR	9	35.087	1.812	-0.190	1.00	0.00	C
ATOM	139	OH	TYR	9	36.185	1.210	-0.660	1.00	0.00	O
ATOM	140	HH	TYR	9	36.923	1.591	-0.178	1.00	0.00	H
ATOM	141	CE2	TYR	9	35.215	2.962	0.654	1.00	0.00	C
ATOM	142	HE2	TYR	9	36.189	3.221	1.044	1.00	0.00	H
ATOM	143	CD2	TYR	9	34.022	3.580	1.151	1.00	0.00	C
ATOM	144	HD2	TYR	9	34.044	4.253	1.995	1.00	0.00	H
ATOM	145	C	TYR	9	29.433	5.466	0.570	1.00	0.00	C
ATOM	146	O	TYR	9	28.953	5.266	1.735	1.00	0.00	O
ATOM	147	N	ARG	10	28.995	6.427	-0.246	1.00	0.00	N
ATOM	148	H	ARG	10	29.232	6.332	-1.222	1.00	0.00	H
ATOM	149	CA	ARG	10	27.934	7.385	0.163	1.00	0.00	C
ATOM	150	HA	ARG	10	28.092	7.626	1.214	1.00	0.00	H
ATOM	151	CB	ARG	10	27.937	8.662	-0.670	1.00	0.00	C
ATOM	152	HB2	ARG	10	27.305	8.538	-1.550	1.00	0.00	H
ATOM	153	HB3	ARG	10	28.980	8.721	-0.981	1.00	0.00	H
ATOM	154	CG	ARG	10	27.373	9.983	-0.081	1.00	0.00	C
ATOM	155	HG2	ARG	10	26.476	9.750	0.493	1.00	0.00	H
ATOM	156	HG3	ARG	10	27.005	10.503	-0.965	1.00	0.00	H
ATOM	157	CD	ARG	10	28.122	10.995	0.718	1.00	0.00	C
ATOM	158	HD2	ARG	10	27.520	11.768	1.197	1.00	0.00	H
ATOM	159	HD3	ARG	10	28.811	11.409	-0.018	1.00	0.00	H
ATOM	160	NE	ARG	10	28.975	10.417	1.786	1.00	0.00	N
ATOM	161	HE	ARG	10	29.976	10.499	1.686	1.00	0.00	H
ATOM	162	CZ	ARG	10	28.522	9.807	2.841	1.00	0.00	C
ATOM	163	NH1	ARG	10	27.235	9.756	3.194	1.00	0.00	N
ATOM	164	HH11	ARG	10	27.126	9.206	4.033	1.00	0.00	H
ATOM	165	HH12	ARG	10	26.531	10.361	2.795	1.00	0.00	H
ATOM	166	NH2	ARG	10	29.353	9.143	3.560	1.00	0.00	N
ATOM	167	HH21	ARG	10	30.337	9.126	3.331	1.00	0.00	H
ATOM	168	HH22	ARG	10	29.042	8.774	4.448	1.00	0.00	H
ATOM	169	C	ARG	10	26.511	6.686	0.210	1.00	0.00	C
ATOM	170	O	ARG	10	25.538	7.304	0.671	1.00	0.00	O
ATOM	171	N	GLU	11	26.363	5.377	-0.118	1.00	0.00	N
ATOM	172	H	GLU	11	27.120	4.818	-0.486	1.00	0.00	H
ATOM	173	CA	GLU	11	25.188	4.609	0.202	1.00	0.00	C
ATOM	174	HA	GLU	11	24.351	4.976	-0.392	1.00	0.00	H
ATOM	175	CB	GLU	11	25.478	3.172	-0.094	1.00	0.00	C
ATOM	176	HB2	GLU	11	24.587	2.547	-0.051	1.00	0.00	H
ATOM	177	HB3	GLU	11	26.228	2.832	0.621	1.00	0.00	H
ATOM	178	CG	GLU	11	26.074	2.857	-1.528	1.00	0.00	C
ATOM	179	HG2	GLU	11	26.319	1.806	-1.684	1.00	0.00	H
ATOM	180	HG3	GLU	11	27.048	3.345	-1.561	1.00	0.00	H
ATOM	181	CD	GLU	11	25.262	3.226	-2.749	1.00	0.00	C
ATOM	182	OE1	GLU	11	24.025	3.418	-2.647	1.00	0.00	O
ATOM	183	OE2	GLU	11	25.896	3.316	-3.791	1.00	0.00	O

ATOM	184	C	GLU	11	24.758	4.675	1.715	1.00	0.00	C
ATOM	185	O	GLU	11	23.567	4.681	2.001	1.00	0.00	O
ATOM	186	N	ASN	12	25.661	4.593	2.679	1.00	0.00	N
ATOM	187	H	ASN	12	26.639	4.547	2.432	1.00	0.00	H
ATOM	188	CA	ASN	12	25.388	4.812	4.096	1.00	0.00	C
ATOM	189	HA	ASN	12	24.394	4.450	4.361	1.00	0.00	H
ATOM	190	CB	ASN	12	26.430	3.969	4.839	1.00	0.00	C
ATOM	191	HB2	ASN	12	27.414	4.418	4.704	1.00	0.00	H
ATOM	192	HB3	ASN	12	26.389	3.009	4.323	1.00	0.00	H
ATOM	193	CG	ASN	12	26.283	3.764	6.324	1.00	0.00	C
ATOM	194	OD1	ASN	12	25.567	4.436	7.092	1.00	0.00	O
ATOM	195	ND2	ASN	12	27.034	2.894	6.919	1.00	0.00	N
ATOM	196	HD21	ASN	12	26.602	2.496	7.740	1.00	0.00	H
ATOM	197	HD22	ASN	12	27.717	2.419	6.346	1.00	0.00	H
ATOM	198	C	ASN	12	25.406	6.287	4.482	1.00	0.00	C
ATOM	199	O	ASN	12	26.291	7.079	4.027	1.00	0.00	O
ATOM	200	N	MET	13	24.459	6.636	5.368	1.00	0.00	N
ATOM	201	H	MET	13	23.779	5.955	5.676	1.00	0.00	H
ATOM	202	CA	MET	13	24.230	8.069	5.841	1.00	0.00	C
ATOM	203	HA	MET	13	24.228	8.659	4.925	1.00	0.00	H
ATOM	204	CB	MET	13	22.865	8.050	6.488	1.00	0.00	C
ATOM	205	HB2	MET	13	22.716	9.041	6.917	1.00	0.00	H
ATOM	206	HB3	MET	13	22.864	7.313	7.292	1.00	0.00	H
ATOM	207	CG	MET	13	21.731	7.722	5.530	1.00	0.00	C
ATOM	208	HG2	MET	13	21.708	8.243	4.572	1.00	0.00	H
ATOM	209	HG3	MET	13	20.836	8.042	6.064	1.00	0.00	H
ATOM	210	SD	MET	13	21.426	5.981	5.229	1.00	0.00	S
ATOM	211	CE	MET	13	19.829	5.944	4.553	1.00	0.00	C
ATOM	212	HE1	MET	13	19.570	5.047	3.989	1.00	0.00	H
ATOM	213	HE2	MET	13	19.033	6.176	5.261	1.00	0.00	H
ATOM	214	HE3	MET	13	19.727	6.677	3.753	1.00	0.00	H
ATOM	215	C	MET	13	25.359	8.595	6.711	1.00	0.00	C
ATOM	216	O	MET	13	25.575	9.766	6.717	1.00	0.00	O
ATOM	217	N	TYR	14	26.086	7.759	7.417	1.00	0.00	N
ATOM	218	H	TYR	14	25.687	6.881	7.719	1.00	0.00	H
ATOM	219	CA	TYR	14	27.296	8.203	8.150	1.00	0.00	C
ATOM	220	HA	TYR	14	27.023	8.945	8.902	1.00	0.00	H
ATOM	221	CB	TYR	14	27.890	7.074	8.926	1.00	0.00	C
ATOM	222	HB2	TYR	14	28.065	6.265	8.217	1.00	0.00	H
ATOM	223	HB3	TYR	14	27.181	6.729	9.679	1.00	0.00	H
ATOM	224	CG	TYR	14	29.113	7.347	9.830	1.00	0.00	C
ATOM	225	CD1	TYR	14	30.325	6.764	9.334	1.00	0.00	C
ATOM	226	HD1	TYR	14	30.383	6.247	8.387	1.00	0.00	H
ATOM	227	CE1	TYR	14	31.598	7.217	9.924	1.00	0.00	C
ATOM	228	HE1	TYR	14	32.524	6.840	9.516	1.00	0.00	H
ATOM	229	CZ	TYR	14	31.566	7.929	11.130	1.00	0.00	C
ATOM	230	OH	TYR	14	32.756	8.181	11.829	1.00	0.00	O
ATOM	231	HH	TYR	14	32.573	8.582	12.682	1.00	0.00	H
ATOM	232	CE2	TYR	14	30.290	8.289	11.653	1.00	0.00	C
ATOM	233	HE2	TYR	14	30.246	8.805	12.601	1.00	0.00	H
ATOM	234	CD2	TYR	14	29.107	7.991	11.010	1.00	0.00	C
ATOM	235	HD2	TYR	14	28.143	8.351	11.340	1.00	0.00	H
ATOM	236	C	TYR	14	28.319	8.657	7.072	1.00	0.00	C
ATOM	237	O	TYR	14	28.452	8.095	5.958	1.00	0.00	O
ATOM	238	N	ARG	15	29.086	9.728	7.384	1.00	0.00	N
ATOM	239	H	ARG	15	28.995	10.013	8.349	1.00	0.00	H
ATOM	240	CA	ARG	15	30.285	10.277	6.685	1.00	0.00	C
ATOM	241	HA	ARG	15	30.560	9.562	5.910	1.00	0.00	H
ATOM	242	CB	ARG	15	29.768	11.616	6.081	1.00	0.00	C
ATOM	243	HB2	ARG	15	29.287	12.154	6.897	1.00	0.00	H
ATOM	244	HB3	ARG	15	29.007	11.279	5.378	1.00	0.00	H
ATOM	245	CG	ARG	15	30.931	12.370	5.430	1.00	0.00	C
ATOM	246	HG2	ARG	15	31.600	11.744	4.839	1.00	0.00	H

```
ATOM    247  HG3 ARG    15       31.476  12.808   6.266  1.00  0.00            H
ATOM    248  CD  ARG    15       30.294  13.390   4.495  1.00  0.00            C
ATOM    249  HD2 ARG    15       29.406  13.807   4.969  1.00  0.00            H
ATOM    250  HD3 ARG    15       30.073  12.899   3.547  1.00  0.00            H
ATOM    251  NE  ARG    15       31.173  14.575   4.192  1.00  0.00            N
ATOM    252  HE  ARG    15       31.082  15.295   4.893  1.00  0.00            H
ATOM    253  CZ  ARG    15       32.182  14.648   3.355  1.00  0.00            C
ATOM    254  NH1 ARG    15       32.393  13.755   2.467  1.00  0.00            N
ATOM    255 HH11 ARG    15       31.854  12.901   2.464  1.00  0.00            H
ATOM    256 HH12 ARG    15       33.155  14.026   1.861  1.00  0.00            H
ATOM    257  NH2 ARG    15       32.806  15.754   3.271  1.00  0.00            N
ATOM    258 HH21 ARG    15       32.557  16.460   3.949  1.00  0.00            H
ATOM    259 HH22 ARG    15       33.461  15.838   2.506  1.00  0.00            H
ATOM    260  C   ARG    15       31.472  10.489   7.654  1.00  0.00            C
ATOM    261  O   ARG    15       31.215  10.732   8.851  1.00  0.00            O
ATOM    262  N   TYR    16       32.688  10.544   7.164  1.00  0.00            N
ATOM    263  H   TYR    16       32.781  10.444   6.163  1.00  0.00            H
ATOM    264  CA  TYR    16       33.943  10.725   7.939  1.00  0.00            C
ATOM    265  HA  TYR    16       33.874  10.108   8.834  1.00  0.00            H
ATOM    266  CB  TYR    16       35.191  10.327   7.228  1.00  0.00            C
ATOM    267  HB2 TYR    16       36.045  10.382   7.904  1.00  0.00            H
ATOM    268  HB3 TYR    16       35.373  10.997   6.389  1.00  0.00            H
ATOM    269  CG  TYR    16       35.300   8.901   6.696  1.00  0.00            C
ATOM    270  CD1 TYR    16       35.800   8.782   5.464  1.00  0.00            C
ATOM    271  HD1 TYR    16       36.059   9.668   4.902  1.00  0.00            H
ATOM    272  CE1 TYR    16       35.814   7.531   4.813  1.00  0.00            C
ATOM    273  HE1 TYR    16       36.211   7.475   3.811  1.00  0.00            H
ATOM    274  CZ  TYR    16       35.534   6.307   5.476  1.00  0.00            C
ATOM    275  OH  TYR    16       35.580   5.136   4.778  1.00  0.00            O
ATOM    276  HH  TYR    16       35.840   5.214   3.858  1.00  0.00            H
ATOM    277  CE2 TYR    16       34.956   6.452   6.692  1.00  0.00            C
ATOM    278  HE2 TYR    16       34.648   5.553   7.207  1.00  0.00            H
ATOM    279  CD2 TYR    16       34.787   7.745   7.282  1.00  0.00            C
ATOM    280  HD2 TYR    16       34.322   7.769   8.257  1.00  0.00            H
ATOM    281  C   TYR    16       33.981  12.192   8.399  1.00  0.00            C
ATOM    282  O   TYR    16       33.406  13.082   7.686  1.00  0.00            O
ATOM    283  N   NME    17       34.546  12.463   9.606  1.00  0.00            N
ATOM    284  H   NME    17       34.891  11.690  10.158  1.00  0.00            H
ATOM    285  CH3 NME    17       34.687  13.813  10.166  1.00  0.00            C
ATOM    286 HH31 NME    17       33.728  14.304  10.334  1.00  0.00            H
ATOM    287 HH32 NME    17       35.135  13.532  11.119  1.00  0.00            H
ATOM    288 HH33 NME    17       35.364  14.503   9.662  1.00  0.00            H
TER     289      NME    17
END
```

Cluster 2:

```
ATOM      1 HH31 ACE     1       39.300  18.695   6.491  1.00  0.00            H
ATOM      2  CH3 ACE     1       38.572  17.884   6.530  1.00  0.00            C
ATOM      3 HH32 ACE     1       37.675  18.465   6.746  1.00  0.00            H
ATOM      4 HH33 ACE     1       38.863  17.139   7.271  1.00  0.00            H
ATOM      5  C   ACE     1       38.344  17.208   5.157  1.00  0.00            C
ATOM      6  O   ACE     1       37.839  17.911   4.256  1.00  0.00            O
ATOM      7  N   ASN     2       38.819  15.996   4.863  1.00  0.00            N
ATOM      8  H   ASN     2       39.152  15.364   5.576  1.00  0.00            H
ATOM      9  CA  ASN     2       38.799  15.331   3.548  1.00  0.00            C
ATOM     10  HA  ASN     2       37.930  15.560   2.932  1.00  0.00            H
ATOM     11  CB  ASN     2       40.025  15.921   2.775  1.00  0.00            C
ATOM     12  HB2 ASN     2       40.911  15.597   3.322  1.00  0.00            H
ATOM     13  HB3 ASN     2       39.832  16.993   2.811  1.00  0.00            H
ATOM     14  CG  ASN     2       40.146  15.596   1.286  1.00  0.00            C
```

ATOM	15	OD1	ASN	2	39.216	15.122	0.696	1.00	0.00	O
ATOM	16	ND2	ASN	2	41.334	15.662	0.765	1.00	0.00	N
ATOM	17	HD21	ASN	2	42.125	15.895	1.348	1.00	0.00	H
ATOM	18	HD22	ASN	2	41.487	15.230	-0.135	1.00	0.00	H
ATOM	19	C	ASN	2	38.813	13.787	3.766	1.00	0.00	C
ATOM	20	O	ASN	2	39.306	13.339	4.817	1.00	0.00	O
ATOM	21	N	ASP	3	38.561	13.017	2.752	1.00	0.00	N
ATOM	22	H	ASP	3	38.087	13.393	1.943	1.00	0.00	H
ATOM	23	CA	ASP	3	38.651	11.533	2.816	1.00	0.00	C
ATOM	24	HA	ASP	3	39.428	11.187	3.498	1.00	0.00	H
ATOM	25	CB	ASP	3	37.399	10.944	3.488	1.00	0.00	C
ATOM	26	HB2	ASP	3	36.792	10.742	2.606	1.00	0.00	H
ATOM	27	HB3	ASP	3	36.909	11.792	3.965	1.00	0.00	H
ATOM	28	CG	ASP	3	37.628	9.691	4.424	1.00	0.00	C
ATOM	29	OD1	ASP	3	37.338	9.814	5.626	1.00	0.00	O
ATOM	30	OD2	ASP	3	37.938	8.592	3.949	1.00	0.00	O
ATOM	31	C	ASP	3	39.018	10.805	1.550	1.00	0.00	C
ATOM	32	O	ASP	3	38.577	11.018	0.415	1.00	0.00	O
ATOM	33	N	TYR	4	39.863	9.800	1.822	1.00	0.00	N
ATOM	34	H	TYR	4	40.076	9.590	2.787	1.00	0.00	H
ATOM	35	CA	TYR	4	40.381	8.904	0.719	1.00	0.00	C
ATOM	36	HA	TYR	4	40.103	9.437	-0.190	1.00	0.00	H
ATOM	37	CB	TYR	4	41.874	8.793	0.839	1.00	0.00	C
ATOM	38	HB2	TYR	4	42.326	8.216	0.032	1.00	0.00	H
ATOM	39	HB3	TYR	4	42.013	8.192	1.738	1.00	0.00	H
ATOM	40	CG	TYR	4	42.656	10.132	1.059	1.00	0.00	C
ATOM	41	CD1	TYR	4	42.968	11.038	-0.029	1.00	0.00	C
ATOM	42	HD1	TYR	4	42.685	10.885	-1.060	1.00	0.00	H
ATOM	43	CE1	TYR	4	43.691	12.147	0.204	1.00	0.00	C
ATOM	44	HE1	TYR	4	43.940	12.789	-0.629	1.00	0.00	H
ATOM	45	CZ	TYR	4	44.215	12.398	1.480	1.00	0.00	C
ATOM	46	OH	TYR	4	45.015	13.441	1.731	1.00	0.00	O
ATOM	47	HH	TYR	4	44.964	13.544	2.684	1.00	0.00	H
ATOM	48	CE2	TYR	4	43.965	11.561	2.541	1.00	0.00	C
ATOM	49	HE2	TYR	4	44.325	11.918	3.495	1.00	0.00	H
ATOM	50	CD2	TYR	4	43.131	10.437	2.382	1.00	0.00	C
ATOM	51	HD2	TYR	4	42.769	9.784	3.162	1.00	0.00	H
ATOM	52	C	TYR	4	39.655	7.537	0.481	1.00	0.00	C
ATOM	53	O	TYR	4	39.877	6.844	-0.478	1.00	0.00	O
ATOM	54	N	GLU	5	38.732	7.222	1.394	1.00	0.00	N
ATOM	55	H	GLU	5	38.755	7.786	2.231	1.00	0.00	H
ATOM	56	CA	GLU	5	37.961	5.931	1.426	1.00	0.00	C
ATOM	57	HA	GLU	5	38.334	5.302	0.618	1.00	0.00	H
ATOM	58	CB	GLU	5	38.358	5.322	2.729	1.00	0.00	C
ATOM	59	HB2	GLU	5	38.243	6.115	3.467	1.00	0.00	H
ATOM	60	HB3	GLU	5	39.395	5.027	2.569	1.00	0.00	H
ATOM	61	CG	GLU	5	37.530	4.143	3.246	1.00	0.00	C
ATOM	62	HG2	GLU	5	37.542	3.194	2.710	1.00	0.00	H
ATOM	63	HG3	GLU	5	36.515	4.537	3.195	1.00	0.00	H
ATOM	64	CD	GLU	5	37.807	3.836	4.765	1.00	0.00	C
ATOM	65	OE1	GLU	5	38.932	3.410	5.018	1.00	0.00	O
ATOM	66	OE2	GLU	5	36.879	4.034	5.618	1.00	0.00	O
ATOM	67	C	GLU	5	36.499	6.192	1.309	1.00	0.00	C
ATOM	68	O	GLU	5	35.745	5.596	0.529	1.00	0.00	O
ATOM	69	N	ASP	6	35.980	7.053	2.155	1.00	0.00	N
ATOM	70	H	ASP	6	36.584	7.553	2.791	1.00	0.00	H
ATOM	71	CA	ASP	6	34.490	7.353	2.199	1.00	0.00	C
ATOM	72	HA	ASP	6	34.090	6.354	2.374	1.00	0.00	H
ATOM	73	CB	ASP	6	34.261	8.258	3.409	1.00	0.00	C
ATOM	74	HB2	ASP	6	34.363	9.312	3.151	1.00	0.00	H
ATOM	75	HB3	ASP	6	35.008	7.917	4.126	1.00	0.00	H
ATOM	76	CG	ASP	6	32.896	8.053	4.117	1.00	0.00	C
ATOM	77	OD1	ASP	6	32.641	7.023	4.735	1.00	0.00	O

ATOM	78	OD2	ASP	6	32.023	8.959	4.196	1.00	0.00	O
ATOM	79	C	ASP	6	33.872	7.979	0.894	1.00	0.00	C
ATOM	80	O	ASP	6	32.657	8.244	0.904	1.00	0.00	O
ATOM	81	N	ARG	7	34.635	8.228	-0.197	1.00	0.00	N
ATOM	82	H	ARG	7	35.586	7.887	-0.190	1.00	0.00	H
ATOM	83	CA	ARG	7	34.095	8.733	-1.543	1.00	0.00	C
ATOM	84	HA	ARG	7	33.525	9.640	-1.342	1.00	0.00	H
ATOM	85	CB	ARG	7	35.236	9.305	-2.381	1.00	0.00	C
ATOM	86	HB2	ARG	7	34.851	9.694	-3.323	1.00	0.00	H
ATOM	87	HB3	ARG	7	36.020	8.560	-2.509	1.00	0.00	H
ATOM	88	CG	ARG	7	35.917	10.511	-1.741	1.00	0.00	C
ATOM	89	HG2	ARG	7	36.474	10.204	-0.856	1.00	0.00	H
ATOM	90	HG3	ARG	7	35.123	11.228	-1.527	1.00	0.00	H
ATOM	91	CD	ARG	7	36.925	11.110	-2.758	1.00	0.00	C
ATOM	92	HD2	ARG	7	36.490	11.618	-3.619	1.00	0.00	H
ATOM	93	HD3	ARG	7	37.302	10.204	-3.232	1.00	0.00	H
ATOM	94	NE	ARG	7	37.854	12.032	-2.030	1.00	0.00	N
ATOM	95	HE	ARG	7	37.703	12.001	-1.032	1.00	0.00	H
ATOM	96	CZ	ARG	7	39.111	12.300	-2.411	1.00	0.00	C
ATOM	97	NH1	ARG	7	39.547	11.888	-3.510	1.00	0.00	N
ATOM	98	HH11	ARG	7	40.538	11.970	-3.686	1.00	0.00	H
ATOM	99	HH12	ARG	7	38.979	11.332	-4.133	1.00	0.00	H
ATOM	100	NH2	ARG	7	39.823	13.208	-1.793	1.00	0.00	N
ATOM	101	HH21	ARG	7	39.433	13.650	-0.973	1.00	0.00	H
ATOM	102	HH22	ARG	7	40.595	13.575	-2.332	1.00	0.00	H
ATOM	103	C	ARG	7	33.158	7.815	-2.410	1.00	0.00	C
ATOM	104	O	ARG	7	33.009	7.886	-3.650	1.00	0.00	O
ATOM	105	N	TYR	8	32.529	6.731	-1.837	1.00	0.00	N
ATOM	106	H	TYR	8	32.615	6.586	-0.841	1.00	0.00	H
ATOM	107	CA	TYR	8	31.476	5.922	-2.594	1.00	0.00	C
ATOM	108	HA	TYR	8	31.714	5.772	-3.647	1.00	0.00	H
ATOM	109	CB	TYR	8	31.495	4.529	-1.914	1.00	0.00	C
ATOM	110	HB2	TYR	8	30.632	3.967	-2.273	1.00	0.00	H
ATOM	111	HB3	TYR	8	31.374	4.845	-0.879	1.00	0.00	H
ATOM	112	CG	TYR	8	32.752	3.689	-1.989	1.00	0.00	C
ATOM	113	CD1	TYR	8	33.576	3.565	-0.856	1.00	0.00	C
ATOM	114	HD1	TYR	8	33.333	4.221	-0.033	1.00	0.00	H
ATOM	115	CE1	TYR	8	34.721	2.826	-0.943	1.00	0.00	C
ATOM	116	HE1	TYR	8	35.390	2.644	-0.115	1.00	0.00	H
ATOM	117	CZ	TYR	8	34.981	2.115	-2.127	1.00	0.00	C
ATOM	118	OH	TYR	8	36.198	1.569	-2.158	1.00	0.00	O
ATOM	119	HH	TYR	8	36.432	1.191	-3.008	1.00	0.00	H
ATOM	120	CE2	TYR	8	34.166	2.181	-3.227	1.00	0.00	C
ATOM	121	HE2	TYR	8	34.551	1.735	-4.132	1.00	0.00	H
ATOM	122	CD2	TYR	8	33.005	2.842	-3.088	1.00	0.00	C
ATOM	123	HD2	TYR	8	32.519	2.877	-4.052	1.00	0.00	H
ATOM	124	C	TYR	8	30.003	6.534	-2.547	1.00	0.00	C
ATOM	125	O	TYR	8	29.658	7.126	-1.548	1.00	0.00	O
ATOM	126	N	TYR	9	29.247	6.299	-3.597	1.00	0.00	N
ATOM	127	H	TYR	9	29.459	5.690	-4.374	1.00	0.00	H
ATOM	128	CA	TYR	9	27.917	6.852	-3.666	1.00	0.00	C
ATOM	129	HA	TYR	9	27.804	7.772	-3.093	1.00	0.00	H
ATOM	130	CB	TYR	9	27.688	7.304	-5.173	1.00	0.00	C
ATOM	131	HB2	TYR	9	27.912	6.473	-5.842	1.00	0.00	H
ATOM	132	HB3	TYR	9	28.313	8.188	-5.296	1.00	0.00	H
ATOM	133	CG	TYR	9	26.212	7.847	-5.445	1.00	0.00	C
ATOM	134	CD1	TYR	9	25.820	9.172	-5.067	1.00	0.00	C
ATOM	135	HD1	TYR	9	26.562	9.725	-4.510	1.00	0.00	H
ATOM	136	CE1	TYR	9	24.540	9.658	-5.473	1.00	0.00	C
ATOM	137	HE1	TYR	9	24.182	10.611	-5.111	1.00	0.00	H
ATOM	138	CZ	TYR	9	23.666	8.819	-6.159	1.00	0.00	C
ATOM	139	OH	TYR	9	22.416	9.373	-6.447	1.00	0.00	O
ATOM	140	HH	TYR	9	21.731	8.717	-6.600	1.00	0.00	H

ATOM	141	CE2	TYR	9	24.010	7.574	-6.604	1.00	0.00	C
ATOM	142	HE2	TYR	9	23.321	6.959	-7.163	1.00	0.00	H
ATOM	143	CD2	TYR	9	25.269	7.095	-6.285	1.00	0.00	C
ATOM	144	HD2	TYR	9	25.626	6.144	-6.652	1.00	0.00	H
ATOM	145	C	TYR	9	26.953	5.841	-3.139	1.00	0.00	C
ATOM	146	O	TYR	9	26.562	4.878	-3.818	1.00	0.00	O
ATOM	147	N	ARG	10	26.513	6.086	-1.883	1.00	0.00	N
ATOM	148	H	ARG	10	27.180	6.695	-1.430	1.00	0.00	H
ATOM	149	CA	ARG	10	25.458	5.306	-1.170	1.00	0.00	C
ATOM	150	HA	ARG	10	24.731	5.000	-1.923	1.00	0.00	H
ATOM	151	CB	ARG	10	26.001	3.993	-0.539	1.00	0.00	C
ATOM	152	HB2	ARG	10	26.670	3.438	-1.196	1.00	0.00	H
ATOM	153	HB3	ARG	10	25.134	3.334	-0.480	1.00	0.00	H
ATOM	154	CG	ARG	10	26.728	4.045	0.829	1.00	0.00	C
ATOM	155	HG2	ARG	10	27.155	3.057	0.997	1.00	0.00	H
ATOM	156	HG3	ARG	10	25.933	4.293	1.532	1.00	0.00	H
ATOM	157	CD	ARG	10	27.860	5.175	0.860	1.00	0.00	C
ATOM	158	HD2	ARG	10	27.639	6.195	0.547	1.00	0.00	H
ATOM	159	HD3	ARG	10	28.481	4.871	0.017	1.00	0.00	H
ATOM	160	NE	ARG	10	28.537	5.192	2.189	1.00	0.00	N
ATOM	161	HE	ARG	10	28.059	4.689	2.923	1.00	0.00	H
ATOM	162	CZ	ARG	10	29.689	5.820	2.435	1.00	0.00	C
ATOM	163	NH1	ARG	10	30.238	6.669	1.678	1.00	0.00	N
ATOM	164	HH11	ARG	10	30.833	7.323	2.166	1.00	0.00	H
ATOM	165	HH12	ARG	10	30.010	6.776	0.700	1.00	0.00	H
ATOM	166	NH2	ARG	10	30.400	5.537	3.475	1.00	0.00	N
ATOM	167	HH21	ARG	10	31.186	6.131	3.697	1.00	0.00	H
ATOM	168	HH22	ARG	10	30.194	4.756	4.080	1.00	0.00	H
ATOM	169	C	ARG	10	24.700	6.076	-0.103	1.00	0.00	C
ATOM	170	O	ARG	10	25.241	7.063	0.425	1.00	0.00	O
ATOM	171	N	GLU	11	23.544	5.619	0.359	1.00	0.00	N
ATOM	172	H	GLU	11	23.131	4.855	-0.156	1.00	0.00	H
ATOM	173	CA	GLU	11	22.682	6.246	1.447	1.00	0.00	C
ATOM	174	HA	GLU	11	22.983	7.282	1.605	1.00	0.00	H
ATOM	175	CB	GLU	11	21.109	6.212	1.082	1.00	0.00	C
ATOM	176	HB2	GLU	11	20.516	6.887	1.699	1.00	0.00	H
ATOM	177	HB3	GLU	11	20.882	5.170	1.310	1.00	0.00	H
ATOM	178	CG	GLU	11	20.771	6.626	-0.426	1.00	0.00	C
ATOM	179	HG2	GLU	11	21.143	5.896	-1.145	1.00	0.00	H
ATOM	180	HG3	GLU	11	21.331	7.549	-0.577	1.00	0.00	H
ATOM	181	CD	GLU	11	19.339	6.988	-0.646	1.00	0.00	C
ATOM	182	OE1	GLU	11	18.548	6.031	-0.591	1.00	0.00	O
ATOM	183	OE2	GLU	11	18.970	8.173	-0.766	1.00	0.00	O
ATOM	184	C	GLU	11	22.964	5.695	2.833	1.00	0.00	C
ATOM	185	O	GLU	11	22.551	6.223	3.859	1.00	0.00	O
ATOM	186	N	ASN	12	23.578	4.530	2.947	1.00	0.00	N
ATOM	187	H	ASN	12	23.972	4.142	2.102	1.00	0.00	H
ATOM	188	CA	ASN	12	24.046	3.843	4.199	1.00	0.00	C
ATOM	189	HA	ASN	12	23.305	4.107	4.954	1.00	0.00	H
ATOM	190	CB	ASN	12	23.936	2.336	3.982	1.00	0.00	C
ATOM	191	HB2	ASN	12	24.626	1.909	3.254	1.00	0.00	H
ATOM	192	HB3	ASN	12	22.931	2.043	3.677	1.00	0.00	H
ATOM	193	CG	ASN	12	24.230	1.545	5.228	1.00	0.00	C
ATOM	194	OD1	ASN	12	23.451	1.586	6.152	1.00	0.00	O
ATOM	195	ND2	ASN	12	25.421	0.947	5.357	1.00	0.00	N
ATOM	196	HD21	ASN	12	25.558	0.541	6.272	1.00	0.00	H
ATOM	197	HD22	ASN	12	26.116	0.841	4.632	1.00	0.00	H
ATOM	198	C	ASN	12	25.396	4.247	4.733	1.00	0.00	C
ATOM	199	O	ASN	12	26.433	4.464	4.050	1.00	0.00	O
ATOM	200	N	MET	13	25.501	4.482	6.056	1.00	0.00	N
ATOM	201	H	MET	13	24.630	4.376	6.556	1.00	0.00	H
ATOM	202	CA	MET	13	26.651	4.964	6.897	1.00	0.00	C
ATOM	203	HA	MET	13	26.117	5.581	7.620	1.00	0.00	H

ATOM	204	CB	MET	13	27.165	3.630	7.687	1.00	0.00	C
ATOM	205	HB2	MET	13	27.804	2.967	7.104	1.00	0.00	H
ATOM	206	HB3	MET	13	26.287	3.049	7.970	1.00	0.00	H
ATOM	207	CG	MET	13	28.022	3.995	8.922	1.00	0.00	C
ATOM	208	HG2	MET	13	27.441	4.398	9.752	1.00	0.00	H
ATOM	209	HG3	MET	13	28.665	4.745	8.462	1.00	0.00	H
ATOM	210	SD	MET	13	29.052	2.692	9.577	1.00	0.00	S
ATOM	211	CE	MET	13	29.865	3.658	10.898	1.00	0.00	C
ATOM	212	HE1	MET	13	30.081	2.949	11.698	1.00	0.00	H
ATOM	213	HE2	MET	13	30.796	4.177	10.670	1.00	0.00	H
ATOM	214	HE3	MET	13	29.239	4.427	11.350	1.00	0.00	H
ATOM	215	C	MET	13	27.771	5.749	6.237	1.00	0.00	C
ATOM	216	O	MET	13	28.758	5.194	5.694	1.00	0.00	O
ATOM	217	N	TYR	14	27.698	7.110	6.193	1.00	0.00	N
ATOM	218	H	TYR	14	26.974	7.550	6.742	1.00	0.00	H
ATOM	219	CA	TYR	14	28.766	8.013	5.767	1.00	0.00	C
ATOM	220	HA	TYR	14	29.674	7.432	5.609	1.00	0.00	H
ATOM	221	CB	TYR	14	28.423	8.724	4.484	1.00	0.00	C
ATOM	222	HB2	TYR	14	28.551	8.104	3.597	1.00	0.00	H
ATOM	223	HB3	TYR	14	29.289	9.383	4.422	1.00	0.00	H
ATOM	224	CG	TYR	14	27.040	9.454	4.442	1.00	0.00	C
ATOM	225	CD1	TYR	14	26.922	10.899	4.601	1.00	0.00	C
ATOM	226	HD1	TYR	14	27.765	11.534	4.830	1.00	0.00	H
ATOM	227	CE1	TYR	14	25.701	11.528	4.272	1.00	0.00	C
ATOM	228	HE1	TYR	14	25.575	12.600	4.294	1.00	0.00	H
ATOM	229	CZ	TYR	14	24.555	10.733	3.971	1.00	0.00	C
ATOM	230	OH	TYR	14	23.319	11.333	3.799	1.00	0.00	O
ATOM	231	HH	TYR	14	23.206	12.269	3.976	1.00	0.00	H
ATOM	232	CE2	TYR	14	24.664	9.348	3.861	1.00	0.00	C
ATOM	233	HE2	TYR	14	23.740	8.832	3.648	1.00	0.00	H
ATOM	234	CD2	TYR	14	25.941	8.678	4.054	1.00	0.00	C
ATOM	235	HD2	TYR	14	26.120	7.625	3.898	1.00	0.00	H
ATOM	236	C	TYR	14	29.350	8.973	6.872	1.00	0.00	C
ATOM	237	O	TYR	14	28.724	9.221	7.905	1.00	0.00	O
ATOM	238	N	ARG	15	30.518	9.577	6.622	1.00	0.00	N
ATOM	239	H	ARG	15	31.002	9.262	5.793	1.00	0.00	H
ATOM	240	CA	ARG	15	31.204	10.626	7.450	1.00	0.00	C
ATOM	241	HA	ARG	15	30.487	10.996	8.182	1.00	0.00	H
ATOM	242	CB	ARG	15	32.325	9.913	8.277	1.00	0.00	C
ATOM	243	HB2	ARG	15	31.801	9.033	8.649	1.00	0.00	H
ATOM	244	HB3	ARG	15	32.458	10.613	9.102	1.00	0.00	H
ATOM	245	CG	ARG	15	33.648	9.594	7.505	1.00	0.00	C
ATOM	246	HG2	ARG	15	34.397	10.385	7.456	1.00	0.00	H
ATOM	247	HG3	ARG	15	33.265	9.451	6.494	1.00	0.00	H
ATOM	248	CD	ARG	15	34.378	8.259	7.901	1.00	0.00	C
ATOM	249	HD2	ARG	15	33.695	7.410	7.924	1.00	0.00	H
ATOM	250	HD3	ARG	15	34.579	8.414	8.961	1.00	0.00	H
ATOM	251	NE	ARG	15	35.631	8.018	7.244	1.00	0.00	N
ATOM	252	HE	ARG	15	36.166	8.801	6.896	1.00	0.00	H
ATOM	253	CZ	ARG	15	36.168	6.840	6.984	1.00	0.00	C
ATOM	254	NH1	ARG	15	35.720	5.764	7.536	1.00	0.00	N
ATOM	255	HH11	ARG	15	36.019	4.940	7.035	1.00	0.00	H
ATOM	256	HH12	ARG	15	34.915	5.643	8.134	1.00	0.00	H
ATOM	257	NH2	ARG	15	37.223	6.741	6.273	1.00	0.00	N
ATOM	258	HH21	ARG	15	37.438	5.862	5.826	1.00	0.00	H
ATOM	259	HH22	ARG	15	37.439	7.607	5.799	1.00	0.00	H
ATOM	260	C	ARG	15	31.687	11.763	6.570	1.00	0.00	C
ATOM	261	O	ARG	15	31.806	12.876	7.053	1.00	0.00	O
ATOM	262	N	TYR	16	31.857	11.507	5.329	1.00	0.00	N
ATOM	263	H	TYR	16	31.912	10.552	5.005	1.00	0.00	H
ATOM	264	CA	TYR	16	32.135	12.471	4.208	1.00	0.00	C
ATOM	265	HA	TYR	16	31.988	13.492	4.560	1.00	0.00	H
ATOM	266	CB	TYR	16	33.510	12.245	3.706	1.00	0.00	C

```
ATOM    267  HB2 TYR    16      33.535  11.194   3.421  1.00  0.00           H
ATOM    268  HB3 TYR    16      34.154  12.480   4.554  1.00  0.00           H
ATOM    269  CG  TYR    16      34.067  13.068   2.555  1.00  0.00           C
ATOM    270  CD1 TYR    16      33.775  12.596   1.281  1.00  0.00           C
ATOM    271  HD1 TYR    16      33.188  11.711   1.083  1.00  0.00           H
ATOM    272  CE1 TYR    16      34.175  13.384   0.209  1.00  0.00           C
ATOM    273  HE1 TYR    16      33.800  13.177  -0.782  1.00  0.00           H
ATOM    274  CZ  TYR    16      35.009  14.466   0.402  1.00  0.00           C
ATOM    275  OH  TYR    16      35.267  15.241  -0.659  1.00  0.00           O
ATOM    276  HH  TYR    16      35.659  16.097  -0.469  1.00  0.00           H
ATOM    277  CE2 TYR    16      35.267  14.917   1.696  1.00  0.00           C
ATOM    278  HE2 TYR    16      35.909  15.778   1.808  1.00  0.00           H
ATOM    279  CD2 TYR    16      34.748  14.267   2.834  1.00  0.00           C
ATOM    280  HD2 TYR    16      34.923  14.582   3.852  1.00  0.00           H
ATOM    281  C   TYR    16      31.135  12.353   3.036  1.00  0.00           C
ATOM    282  O   TYR    16      30.748  13.404   2.460  1.00  0.00           O
ATOM    283  N   NME    17      30.742  11.177   2.635  1.00  0.00           N
ATOM    284  H   NME    17      31.096  10.357   3.106  1.00  0.00           H
ATOM    285  CH3 NME    17      30.009  10.952   1.357  1.00  0.00           C
ATOM    286 HH31 NME    17      29.653   9.922   1.326  1.00  0.00           H
ATOM    287 HH32 NME    17      29.196  11.674   1.279  1.00  0.00           H
ATOM    288 HH33 NME    17      30.680  11.233   0.546  1.00  0.00           H
TER     289      NME    17
END
```
